# M6A Modification and Transcription Analysis of LncRNA in Cerebral Ischemia/Reperfusion Injury

**DOI:** 10.1155/2024/4596974

**Published:** 2024-10-05

**Authors:** Jierong Mo, Zhiquan Li, Zhengfei Yang, Zuhua Huang, Pengpeng Guo, Jianfeng Gao, Haiqiong xiao, Ping Ye, Haini Qin, Tianen Zhou, Jun Jiang

**Affiliations:** ^1^Department of Emergency, The First People's Hospital of Foshan, Foshan, Guangdong 528000, China; ^2^Guangdong Medical University, Zhanjiang, Guangdong 524000, China; ^3^Sun Yat-Sen Memorial Hospital, Sun Yat-Sen University, Guangzhou, Guangdong 510000, China

**Keywords:** cerebral ischemia/reperfusion injury, GO and KEGG, high-throughput sequencing, LncRNA, transcription and methylation

## Abstract

LncRNA is a major factor in the occurrence and development of many diseases. However, its mechanism in cerebral ischemia/reperfusion injury (CIRI) is yet unknown. In this study, the transcriptional level and methylation modification level of LncRNAs before and after mechanical thrombectomy were compared by high-throughput sequencing. Venn diagram, Spearman correlation analysis, Gene Ontology (GO), Kyoto Encyclopedia of Genes and Genomes (KEGG) enrichment analysis, TargetScan, and miRanda were used to analyze the experimental data. The results showed that four key LncRNAs changed at both transcription and methylation levels. Specifically, LncRNA FAR2, LINC02431, and AL357060.1 were downregulated and hypomethylated, while LncRNA FOXD2-AS1 was upregulated and hypomethylated. Moreover, positive regulation of angiogenesis, protein domain–specific binding, autophagy pathway, PPAR signaling pathway, and MAPK signaling pathway were co-enriched between LncRNAs with different expression levels and different methylation levels. Finally, a LncRNA-miRNA-mRNA network was constructed. Therefore, this study explored the potential key LncRNAs and regulatory mechanisms of CIRI.

## 1. Introduction

There are two types of stroke: ischemic stroke and hemorrhagic stroke. Ischemic stroke is a major research area because it accounts for 87% of all strokes in the United States [1, 2]. Stroke is a major cause of disability and mortality. Restoring blood flow and oxygen delivery to brain tissue in a timely way is the main goal of treatment for patients suffering from acute ischemic stroke (AIS) [3]. Revascularization therapy, however, may have unexpected consequences in some cases, including the potential cerebral ischemia/reperfusion injury (CIRI) due to inflammatory response, neuronal death, cell death, and other factors [4]. Therefore, it is imperative to explore the mechanism underlying CIRI and identify practical targets for diagnosis and treatment.

LncRNA is a noncoding RNA with more than 200 nucleotides that plays a critical role in the regulation of a number of important biological process (BP) [5]. More and more data indicate that LncRNA plays a critical role in many diseases such as tumor development, immunological response, inflammation, stem cell differentiation, and epigenetic regulation [6]. In addition, LncRNA performs regulatory functions by binding to DNA, RNA, and proteins. On the one hand, LncRNA targets miRNA and releases their target mRNA through sponge adsorption; on the other hand, LncRNA, as competing endogenous RNA (ceRNA), binds to mRNA directly and plays its role [7]. Similarly, the previous studies have reported that LncRNA plays a crucial role in CIRI via regulating apoptosis, inflammation, immune response, and so on [8, 9]. For example, LncRNA activated by DNA damage (NORAD) attenuates oxygen-glucose deprivation/reoxygenation (OGD/R)–induced neuron injury through LncRNA NORAD-miR-30a-5p-YWHAG pathway [10]. LncRNA SNHG4 regulates STAT6 by adsorbing miR-449c-5p to inhibit inflammation of CIRI [11]. LncRNA DHFRL1-4 suppresses CIRI by regulating the levels of angiogenesis-related genes [12]. Therefore, LncRNA is closely related to CIRI and may be a potential critical gene in the diagnosis and treatment of CIRI.

M6A modification refers to the addition or deletion of a methyl group (CH3) at the N6 position of adenine nucleotide. It is the most abundant and evolutionarily conserved RNA modification, which is related to the occurrence and development of many diseases [13, 14]. CIRI is one of the diseases regulated by m6A modification. For instance, microRNA-421-3p prevents inflammatory response in CIRI via regulating m6A reader YTHDF1 to inhibit p65 mRNA translation [15]. FTO inhibits cGAS mRNA stability in an m6A-dependent manner to alleviate cerebral I/R–induced neuroinflammation [16]. In addition, lots of research suggested that m6A modification of LncRNA has an important regulated effect on different diseases. M6A-mediated upregulation of LINC00958 increases lipogenesis and acts as a nanotherapeutic target in hepatocellular carcinoma [17]. LncRNA LINC00942 is involved in gastric cancer by suppressing MSI2 degradation to enhance c-Myc mRNA stability [18]. M6A-mediated LINC01003 regulates cell migration via altering the CAV1/FAK signaling pathway in glioma [19]. In summary, m6A modification combined with LncRNA to involve in the pathogenesis of a variety of diseases. However, few studies have revealed the relationship between m6A modification of LncRNA and CIRI.

In the present study, we aim to explore the potential key LncRNAs with different expression levels and different m6A modification levels and their possible mechanism of action. To accomplish this goal, high-throughput sequencing was used to detect the expression level of LncRNA and m6A modification level. Then, bioinformatics analysis was performed to further screen differentially expressed genes, GO items, and KEGG signaling pathways. Finally, the regulatory network of LncRNA-miRNA-mRNA was constructed. The successful development of this project may provide a new therapeutic target for CIRI.

## 2. Materials and Methods

### 2.1. Patients and Samples

A total of 16 pairs of blood samples (preoperative and postoperative) in patients with AIS were collected from the First People's Hospital of Foshan. Recent studies by Androvic et al. and Ahnstedt et al. have shown that gender and age have important effects on the transcriptional response, inflammatory response, and angiogenesis process in ischemic stroke [20, 21]. In addition, the median age of stroke onset in China in recent years was 66 years old (57.0–75.0), and the ratio of male to female was about 6:4 [22]. Therefore, in order to make the patients more representative, we selected patients between the ages of 57.0 - 75.0 (half male and half female). The diagnosis of patients with AIS were performed by computed tomography angiography (CTA) and confirmed by neurosurgeons. In addition, the effect of mechanical thrombectomy in all the patients was confirmed by senior neurosurgeons. Samples were stored in BD PAXgene Blood RNA Tube (Becton, Dickinson and Company, United States) according to the instruction manual. This study was approved by the Ethics Committee of the First People's Hospital of Foshan.

### 2.2. MeRIP Sequencing and RNA Sequencing of LncRNA

On the one hand, the m6A modification level of LncRNA in samples was detected with Arraystar human m6A-mRNA and lncRNA epitranscriptomic microarray (Arraystar) according to the instruction manual [23]. On the other hand, the expression level of LncRNA and mRNA was measured with Arraystar LncRNA/mRNA expression profile microarray according to the manufacture's proposal [24]. Then, the identification of differentially m6A-methylated RNA and differentially expressed RNA were performed.

### 2.3. Screen for Different LncRNAs

To recognize the key LncRNAs, we filter differentially m6A-methylated LncRNAs and differentially expressed LncRNAs in a threshold of |fold change (FC)| > 1.5 and *p* < 0.05. A volcano plot was used to show the differentially expressed and differentially methylated LncRNAs. Venn diagram was utilized to further confirm the key LncRNAs with differential expression level and differential methylation modification level simultaneously. The paired sample testing and visualization of key LncRNAs were performed using Sangerbox 3.0 (http://www.sangerbox.com/tool) with paired Student's *t*-test analysis, and *p* < 0.05 was considered significant [25].

### 2.4. Correlation Analysis

Although LncRNA with differential expression level and differential methylation modification level simultaneously was identified, it is not clear about the relationship between expression level and methylation modification level. Therefore, to explore the correlation between differential expression and differential methylation levels of LncRNAs from the previous steps, we performed a Spearman correlation analysis in R software using “ggplot2” package to facilitate visualization [26].

### 2.5. Enrichment Analysis of GO and KEGG Pathway

To explore probable BP, molecular function (MF), cellular component (CC), and signaling pathway of LncRNA with differential expression and differential methylation, we evaluated the correlation of differential expression mRNA and LncRNA with differential expression level and differential methylation level, respectively, through Pearson correlation analysis (*p* < 0.01, Tables S1–S4). Furthermore, enrichment analyses of GO (*p* value cutoff: 0.01 and min enrichment: 1.5) and KEGG (*p* value cutoff: 0.01 and min enrichment: 1.5) were performed with Metascape (http://metascape.org) [27], and *p* value < 0.05 was considered significant.

### 2.6. Prediction of Targeted miRNA and Targeted mRNA

It was reported that LncRNA regulates the occurrence and progression of diseases by LncRNA-miRNA-mRNA usually, so we aimed to formulate the possible network of LncRNA-miRNA-mRNA in CIRI. In order to improve the accuracy, we combined TargetScan (context+ score = −0.1) and miRanda (miRanda score = 140 and miRanda energy = 1.0) to predict the targeted miRNAs of key LncRNAs [28]. In addition, the targeted mRNAs of miRNAs were predicted via TargetScan (context+ score = −0.1). Finally, the first 10 miRNAs and the first 10 mRNAs were used to construct the regulatory network according to the correlation coefficient.

### 2.7. qRT-PCR and MeRip-qPCR

The expression level and methylation level of key LncRNAs were detected by qRT-PCR under the conditions of initial denaturation step (95°C for 10 min), 40 cycles of denaturation (95°C for 10 s) and annealing (60°C for 60 s) using 2X PCR master mix (Arraystar) according to the manufacturer's instructions. In addition, the methylation level of key LncRNAs were detected by MeRip-qPCR using affinity-purified anti-m6A rabbit polyclonal antibody (Synaptic Systems, 202003), Dynabeads M-280 Sheep Anti-Rabbit IgG (Invitrogen, 11203D), RNase inhibitor (Enzymatics, Y9240L) and 2X PCR master mix (Arraystar, AS-MR-006-5) according to the manufacturer's instructions. The primers (Table 1) of key LncRNA are as follows:

### 2.8. Statistical Analysis

The data were analyzed with GraphPad Prism 8 (GraphPad Software, La Jolla, California, United States) using paired Student's *t*-test analysis between preoperative and postoperative, and the results were expressed as the mean ± SD. *p* < 0.05 was considered statistically significant.

## 3. Result

### 3.1. Identification of Differential Expression LncRNAs and Differential Methylation LncRNAs

**Figure 1:**
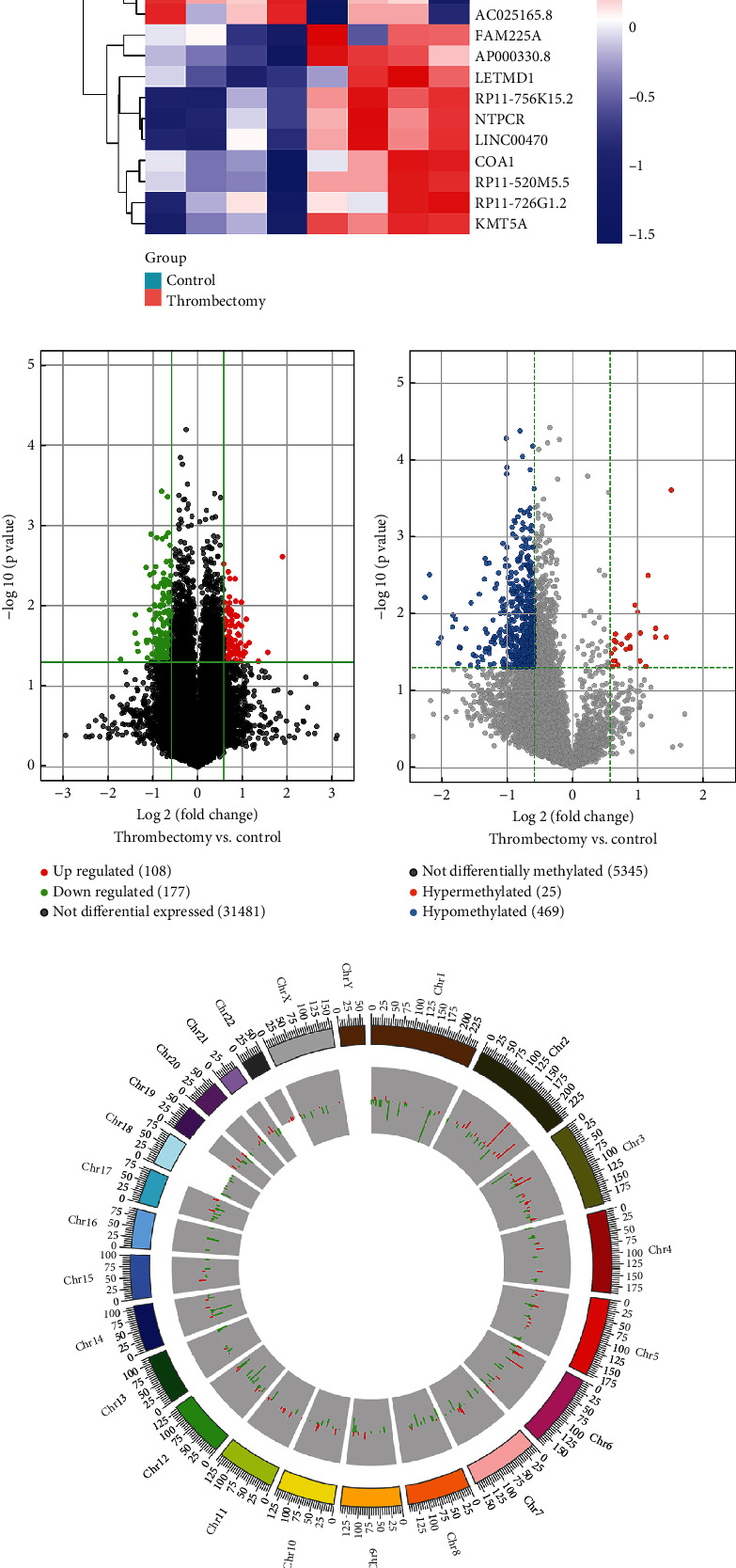
Identification of LncRNAs with differential expression level and differential methylation level between preoperative and postoperative samples. (a) The heat map shows the top 10 LncRNAs with the most significant upregulation and downregulation. (b) The heat map shows the top 10 LncRNAs with the most significant hypermethylation and hypomethylation. (c, d) The volcano plot shows LncRNAs with differential expression level (upregulated and downregulated) and differential methylation level (hypermethylation and hypomethylation), respectively. (e, f) The circos plot shows the chromosomal locations of differentially expressed LncRNAs and differentially methylated LncRNAs and their degree of difference, respectively. Red represents upregulation, and green represents downregulation.

A series of differential expression LncRNAs and differential methylation LncRNAs were detected by high-throughput sequencing between preoperative and postoperative. The top 10 upregulated LncRNAs and top 10 downregulated LncRNAs were shown in the heat map (Figure 1(a)). Similarly, the top 10 hypermethylated LncRNAs and the top 10 hypomethylated LncRNAs were shown in the heat map (Figure 1(b)). In addition, the volcanic plot indicated that there were 108 upregulated LncRNAs and 177 downregulated LncRNAs (Figure 1(c)). The volcano plot also indicated that there were 25 hypermethylated LncRNAs and 469 hypomethylated LncRNAs (Figure 1(d)). The location in chromosomes of differentially expressed LncRNAs and differentially methylated LncRNAs are shown in Figures 1(e) and 1(f).

### 3.2. Identification of Key LncRNAs

**Figure 2:**
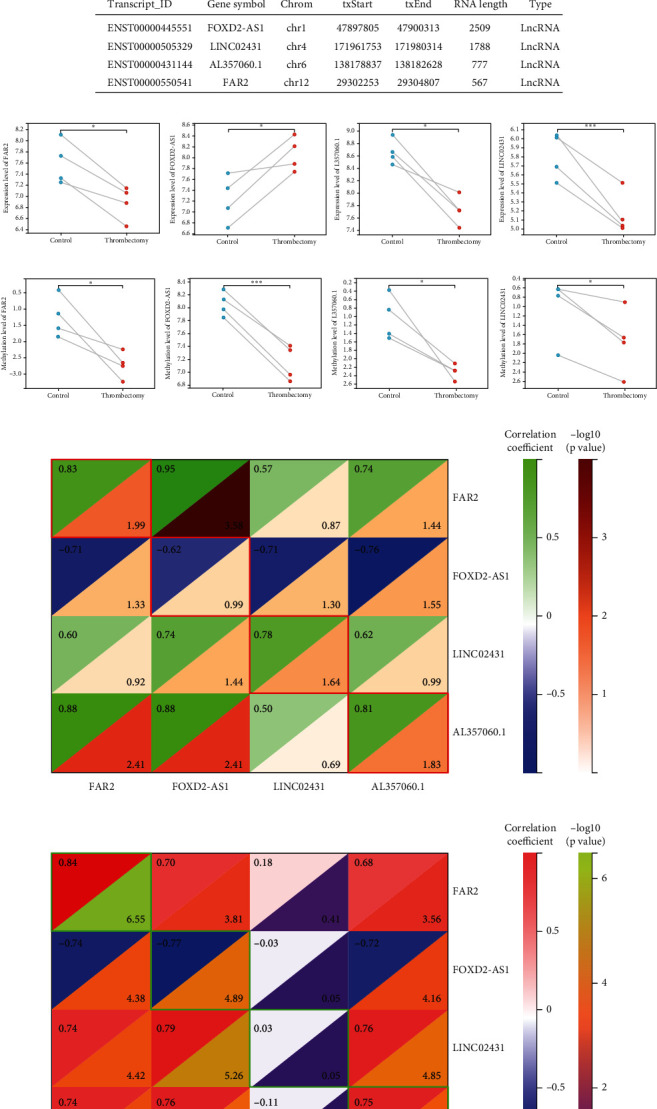
Identification of the key LncRNAs. (a) The Venn diagram shows the LncRNAs with both differential expression level and differential methylation level. (b) The three-line table shows the transcription ID, gene symbol, exact chromosomal location, and gene length of the key LncRNAs. The expression level (c) and methylation level (d) of the key LncRNAs in the four groups of samples are shown by paired sample test and visualization between preoperative and postoperative samples. (e) Correlation analysis shows the correlation between the expression level and the methylation level of the key LncRNAs in the four groups of samples used for sequencing. (f) Correlation analysis shows the correlation between the expression level and the methylation level of the key LncRNAs in the 12 groups of samples used for PCR. ^∗^*p* < 0.05, ^∗∗^*p* < 0.01, and ^∗∗∗^*p* < 0.001.

The intersection of differentially expressed LncRNAs and differentially methylated LncRNAs was performed using the Venn diagram. The results revealed that the methylation level and expression level of the four LncRNAs (LncRNA FOXD2-AS1, LINC02431, AL357060.1, and LncRNA FAR2) were changed simultaneously (Figure 2(a)). The transcript ID, gene symbol, chromosome position, and RNA length are displayed in Figure 2(b). The paired sample test and visualization were performed to show the expression matrix and methylation modification matrix of the four key LncRNAs. The expression of LncRNA FAR2, AL357060.1, and LINC02431 was decreased in the thrombectomy group, accompanied by hypomethylation (Figures 2(c) and 2(d)). Finally, correlation analysis was performed on the four groups of samples used for sequencing, and it was found that the change of expression level was correlated with the change of methylation level in LINC02431, AL357060.1, and LncRNA FAR2. However, there was no significant correlation between the expression level and the methylation level in LncRNA FOXD2-AS1 (Figure 2(e)). In addition, correlation analysis of 12 groups of samples used for PCR revealed that the expression levels of LncRNA FAR2, LncRNA FOXD2-AS1, and AL357060.1 were correlated with methylation levels, but not in LINC02431 (Figure 2(f)).

### 3.3. Enrichment Analysis of GO

**Figure 3:**
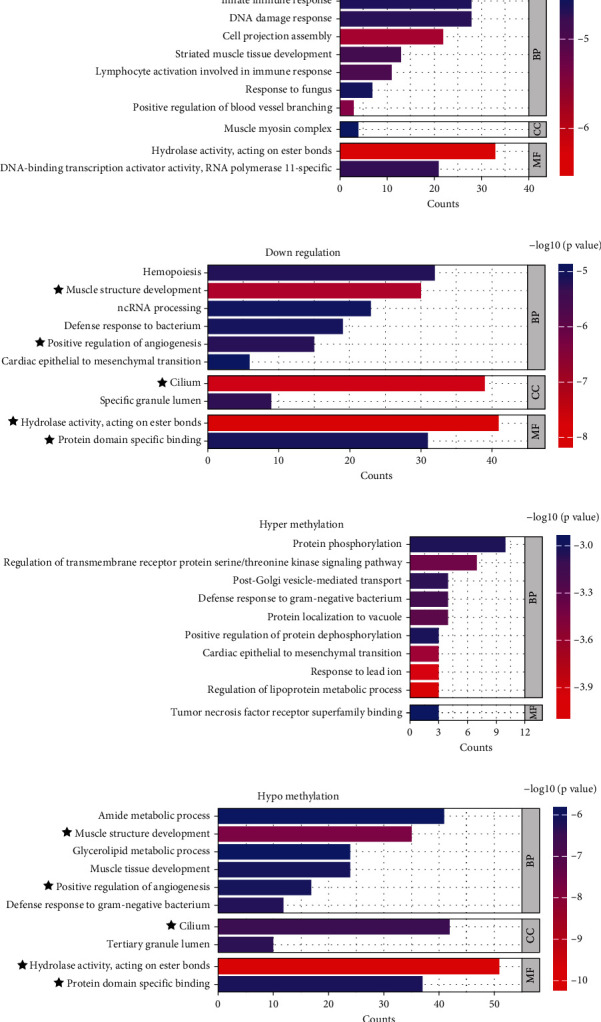
(a, b) The top 10 most significant GO terms enriched by the upregulated LncRNAs and the downregulated LncRNAs, respectively. (c, d) The top 10 most significant GO terms enriched by the hypermethylated LncRNAs and the hypomethylated LncRNAs, respectively. ★ represents coenriched GO terms between downregulated LncRNAs and hypomethylated LncRNAs.

The GO analysis showed that the upregulated LncRNAs–related mRNAs were enriched in positive regulation of blood vessel branching, DNA-binding transcription activator activity, RNA polymerase II specific, DNA damage response, hydrolase activity, and acting on ester bonds (Figure 3(a)). In addition, the GO analysis also showed that downregulated LncRNAs–related mRNAs were enriched in hydrolase activity, acting on ester bonds, cilium, positive regulation of angiogenesis, protein domain–specific binding, and defense response to bacterium (Figure 3(b)). The enrichment of hypermethylated LncRNAs–related mRNAs of GO analysis contained regulation of lipoprotein metabolic process, regulation of transmembrane receptor protein serine/threonine kinase signaling pathway, protein phosphorylation, and positive regulation of protein dephosphorylation (Figure 3(c)). Furthermore, the enrichment of hypomethylated LncRNAs–related mRNAs of GO analysis contains hydrolase activity, acting on ester bonds, cilium, defense response to gram-negative bacterium, protein domain–specific binding, positive regulation of angiogenesis, and glycerolipid metabolic process (Figure 3(d)). Due to the downregulation and hypomethylation of the three key LncRNAs (LINC02431, AL357060.1, and LncRNA FAR2), we found that the defense response to gram-negative bacterium, protein domain–specific binding, and positive regulation of angiogenesis were concurrently enriched in downregulated LncRNAs–related mRNAs and hypomethylated LncRNAs–related mRNAs.

### 3.4. Enrichment Analysis of KEGG

**Figure 4:**
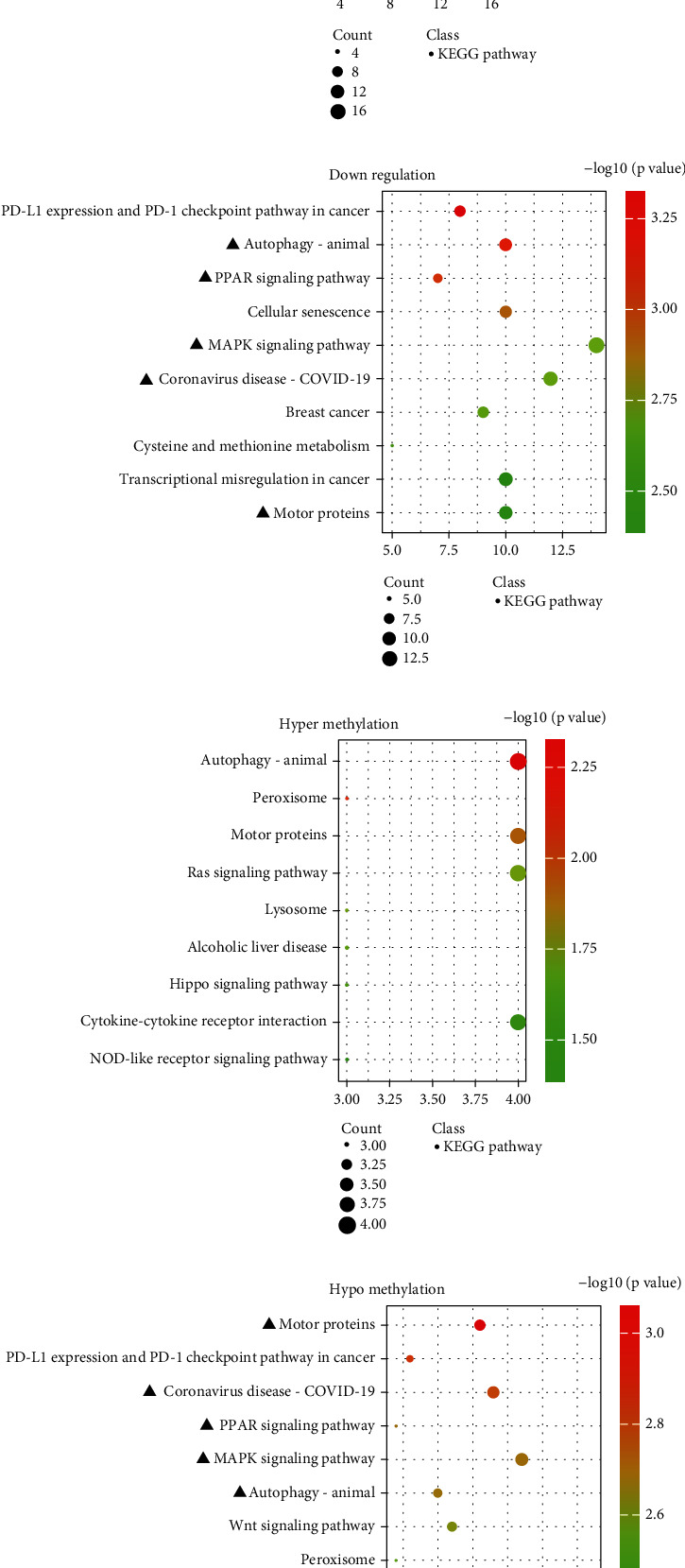
(a, b) The top 10 KEGG signaling pathway enriched by the upregulated LncRNAs and the downregulated LncRNAs, respectively. (c, d) The top 10 KEGG signaling pathway enriched by the hypermethylated LncRNAs and the hypomethylated LncRNAs, respectively. ▲ represents coenriched GO terms between downregulated LncRNAs and hypomethylated LncRNAs.

Upregulated LncRNAs–related mRNAs enriched in KEGG signaling pathways included the insulin signaling pathway, autophagy-animal, MAPK signaling pathway, and B cell receptor signaling pathway (Figure 4(a)). Besides, downregulated LncRNAs–related mRNAs enriched in KEGG signaling pathways included autophagy-animal, PPAR signaling pathway, MAPK signaling pathway, and cellular senescence (Figure 4(b)). Moreover, autophagy-animal, peroxisome, Ras signaling pathway, and lysosome were enriched in hypermethylated LncRNAs–related mRNAs (Figure 4(c)). In addition, the MAPK signaling pathway, PPAR signaling pathway, autophagy-animal, Wnt signaling pathway, and peroxisome were enriched in hypomethylated LncRNAs–related mRNAs (Figure 4(d)). The above results implied that autophagy-animal, MAPK signaling pathway, and PPAR signaling pathway were simultaneously enriched in the downregulated LncRNAs–related mRNAs and hypomethylated LncRNAs–related mRNAs.

### 3.5. Construction of LncRNA-miRNA-mRNA Network

**Figure 5:**
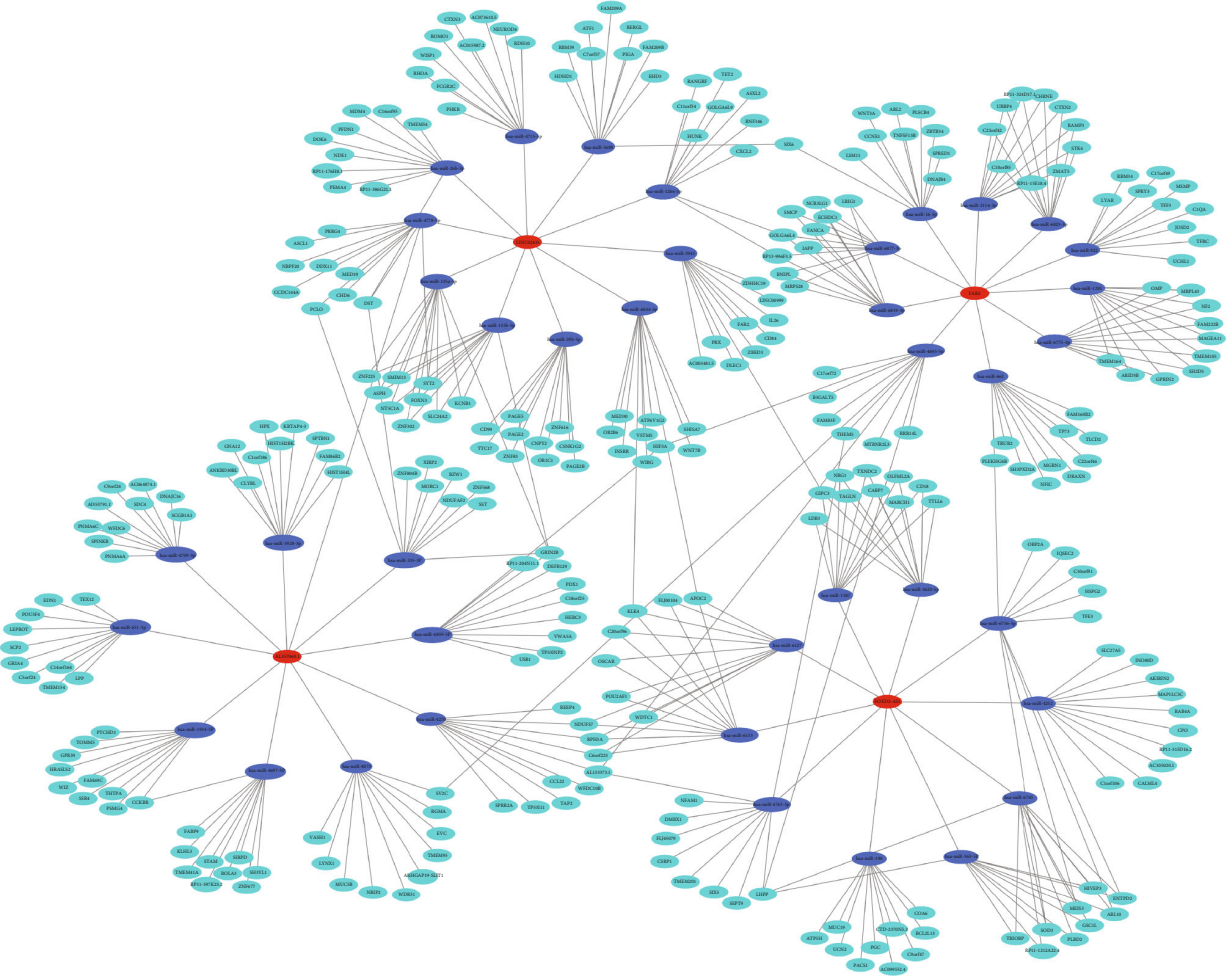
Network of LncRNA-miRNA-mRNA was constructed to demonstrate possible regulatory relationships among lncRNAs, miRNAs, and mRNAs (the top 10 most significant target genes). Red represents LncRNAs, blue represents miRNAs, and green represents mRNAs.

To explore the regulatory mechanism of the key LncRNAs, TargetScan and miRanda were used to predict the targeted miRNAs and mRNAs. The LncRNA-miRNA-mRNA network consisted of the top 10 most relevant miRNAs and mRNAs were shown with Cytoscape (version 3.6.0) (Figure 5).

### 3.6. Verification of the Expression Level and Methylation Level of the Key LncRNAs

**Figure 6:**
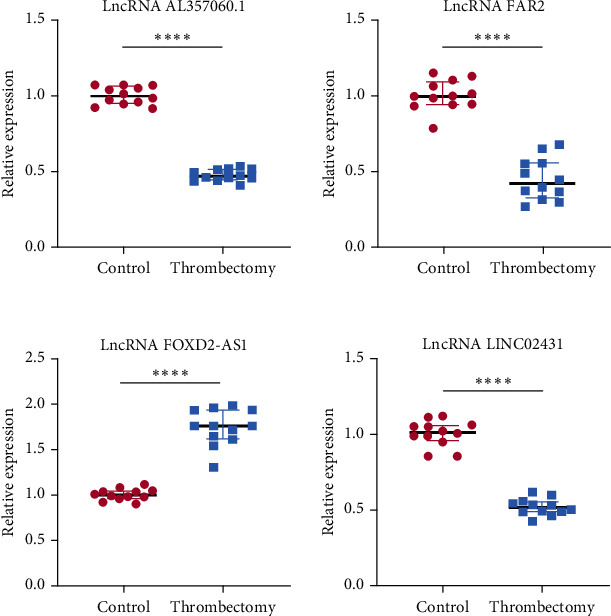
The expression levels of the four key LncRNAs in the 12 pairs of blood were quantified using qRT-PCR. Comparing with the control group, the expression levels of LncRNA FAR2, LncRNA LINC02431, and LncRNA AL357060.1 were downregulated, while the expression level of LncRNA FOXD2-AS1 was upregulated. ^∗∗∗∗^*p* < 0.0001.

**Figure 7:**
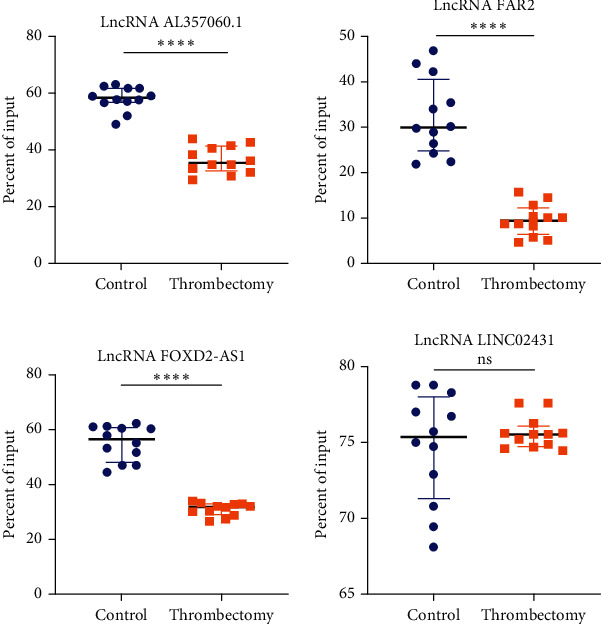
The methylation levels of the four key LncRNAs in the 12 pairs of blood were quantified using MeRip-qPCR. Comparing with control group, the methylation levels of LncRNA FAR2, LncRNA FOXD2-AS1, and LncRNA AL357060.1 were hypomethylated, while the methylation level of LncRNA LINC02431 was not significantly different. ^∗∗∗∗^*p* < 0.0001. ns: nonsignificant.

qRT-PCR and MeRip-qPCR were performed to explore the expression level and methylation level of the four key LncRNAs in another 12 pairs of preoperative and postoperative blood samples, respectively. The results indicated that the expression level of LncRNA FAR2, LINC02431, and AL357060.1 were downregulated, while that of LncRNA FOXD2-AS1 was upregulated (Figure 6). The above results were consistent with the high-throughput sequencing. Besides, the methylation level of LncRNA FAR2, LncRNA FOXD2-AS1, and AL357060.1 were hypomethylated, while there was nonsignificant in LINC02431 (Figure 7).

## 4. Discussion

Ischemic stroke, a disease that often leads to disability and death, has become one of the most challenging problems in the world. Due to the presence of CIRI, the treatment of this disease is not effective [29]. We aimed to explore the potential role of LncRNAs in CIRI. The previous study reported that LncRNA and m6A modification were associated with CIRI. However, the mechanism was still not clear [30, 31]. In this study, high-throughput sequencing was used to filter LncRNAs with differential expression level and differential m6A modification level between preoperative and postoperative. LncRNA FAR2, LINC02431, and AL357060.1 were downregulated and hypomethylated, while LncRNA FOXD2-AS1 was upregulated and hypomethylated. Furthermore, the identification of key LncRNAs and potential mechanisms were performed.

Due to the development of high-throughput technology, more and more LncRNAs have been discovered, and the functions of some LncRNAs were reported. For example, LncRNA FOXD2-AS1 was indicated related to the regulation of cancer, inflammation, apoptosis, and so on [32, 33]. LncRNA FOXD2-AS1 promotes hemangioma progression through the miR-324-3p/PDRG1 pathway [34]. Upregulation of the long noncoding RNA FOXD2-AS1 promotes carcinogenesis by epigenetically silencing EphB3 through EZH2 and LSD1 [35]. In addition, it had been reported that N6-methyladenosine methyltransferase WTAP-stabilized FOXD2-AS1 promoted the osteosarcoma progression through m6A/FOXM1 axis [36]. m6A methyltransferase METTL3-mediated lncRNA FOXD2-AS1 promotes the tumorigenesis of cervical cancer via enhanced itself stability [37]. Our results showed that the expression level of LncRNA FOXD2-AS1 was upregulated while the m6A modification level was downregulated after I/R. Therefore, although the correlation analysis between the expression level and the m6A modification level of LncRNA FOXD2-AS1 was negative in the four groups of samples used for sequencing, these negative results may be due to the small sample size. So we further verified the expression level and the m6A modification level of LncRNA FOXD2-AS1 in another 12 patients with ischemic stroke and found that the results were indeed consistent with the trend of the sequencing, and with the increase of sample size, the expression level of LncRNA FOXD2-AS1 was correlated with methylation level (Figure 2(f)). Therefore, LncRNA FOXD2-AS1 may be an important regulator of CIRI through combining expression level and m6A modification level. In addition, AL357060.1 is associated with liver fibrosis, which can also be aggravated by I/R injury [38, 39]. The results of PCR indicated that the expression level and the methylation level were consistent with sequencing. Besides, twice correlation analyses of AL357060.1 with different sample sizes were statistically significant, so AL357060.1 may participate in the regulation of I/R injury via regulating methylation modification level and expression level. Moreover, there is no report on LncRNA FAR2 as it is a novel LncRNA, but LncRNA FAR2 has similar results with AL357060.1. Hence, it is reasonable to believe that LncRNA FAR2 is involved in the development of CIRI through the mutual regulation of expression level and methylation level. Nevertheless, the results of PCR showed that there was no difference in methylation level of LINC02431 between another 12 groups preoperative and postoperative samples (Figure 2(f)). Moreover, there was no correlation between the expression level and the methylation level of LINC02431 with the increase of sample size. Therefore, LINC02431 perhaps plays a role in CIRI by regulating expression levels alone. In this study, we revealed that LncRNA FOXD2-AS1, LncRNA FAR2, and AL357060.1 may play a key role in CIRI, which may be related to the methylation modification and expression level.

In the present study, the results showed that differentially expressed genes and differentially m6A modified genes were enriched in several GO items before and after mechanical thrombolysis, respectively. Interestingly, both positive regulation of angiogenesis and protein domain–specific binding were enriched in genes with downregulated expression and hypomethylation modification. In mammals, the YTHDF protein is a direct m6A reader containing the YTH domain (a dedicated m6A binding domain). The binding of YTHDF to m6A-modified mRNA induces its translation and is associated with a variety of physiologically relevant processes [40]. This is consistent with our findings which suggested that the three key LncRNAs (LncRNA FAR2, LncRNA FOXD2-AS1, and AL357060.1) may play a regulatory role in CIRI through m6A modification related to YTHDF. In addition, the previous studies reported that angiogenesis is a complex process, and promoting angiogenesis can alleviate CIRI [41]. LncRNA is also involved in the regulation of angiogenesis in CIRI. LncRNA MALAT1 upregulates VEGF-A and ANGPT2 to promote angiogenesis against oxygen-glucose deprivation via targeting miR-145 [42]. LncRNA MALAT1 regulates angiogenesis following OGD/R [43]. LncRNA DHFRL1-4 regulates CIRI by upregulating the levels of angiogenesis [12]. Based on the results of the present study, we speculated that the change of m6A modification level and expression level of LncRNA FAR2, LncRNA FOXD2-AS1, and AL357060.1 inhibited the regulatory process of angiogenesis and promoted CIRI. Besides, a previous study revealed that LncRNA FOXD2-AS1 promotes hemangioma progression via regulating DNA damage regulated 1 (PDRG1) [34]. Our results of GO analysis suggested that the DNA damage response was also enriched. Thus, overexpression of LncRNA FOXD2-AS1 probably promoted CIRI via regulating DNA response and immune response, and hypomethylation of LncRNA FOXD2-AS1 may aggravate CIRI by regulating angiogenesis.

Enrichment analysis of differentially expressed genes and differentially m6A modified genes was performed, and the results showed that the downregulated genes and hypomethylated genes were enriched in the autophagy pathway, PPAR signaling pathway, and MAPK signaling pathway. Autophagy gets close to CIRI. For example, LncRNA H19 induces CIRI via activation of autophagy, and LncRNA MEG3 regulates autophagy after CIRI [44, 45]. Thus, LncRNA FAR2, LncRNA FOXD2-AS1, and AL357060.1 perhaps regulate autophagy to improve CIRI. Furthermore, lots of researches have reported the relationship between LncRNA and PPAR signaling pathway. In detail, LncRNA Blnc1 mediates the permeability and inflammatory response of cerebral hemorrhage by regulating the PPAR-*γ* pathway. Overexpression of LncDBET activates the PPAR signaling pathway and promotes the progression of bladder cancer [46, 47]. PPAR signaling pathway is involved in regulating CIRI by inhibiting of inflammatory responses [48]. Therefore, the above key LncRNAs (LncRNA FAR2, LncRNA FOXD2-AS1, and AL357060.1) may regulate the PPAR pathway to promote CIRI. What's more, some studies revealed that the MAPK signaling pathway has a close regulatory relationship with LncRNA and CIRI. For example, the MAPK signaling pathway is activated by LncRNA PVT1 to regulate CIRI and propofol inhibits the expression of SNHG14 through MAPK signaling pathway to aggravate CIRI [49, 50]. Importantly, a study detecting LncRNA with altered m6A modification level in CIRI rat brain tissue showed that enrichment analysis of the KEGG signaling pathway was enriched to the PPAR signaling pathway and MAPK signaling pathway, and this is highly consistent with our findings in human blood samples of the CIRI. Therefore, we have reason to believe that the autophagy pathway, PPAR signaling pathway, and MAPK signaling pathway are involved in the regulation of CIRI. Similarly, LncRNA FOXD2-AS1 also regulates CIRI by influencing autophagy and MAPK signaling pathway possibly. One study reported that AL357060.1 may affect the recurrence-free survival of patients with hepatocellular carcinoma through the chemokine, cell cycle, Th17 cell differentiation, or thermogenesis [38], and it may be the potential mechanism by which AL357060.1 regulates the above signaling pathways. In addition, methylation of LncRNA FOXD2-AS1 may regulate the above signaling pathways through regulating methyltransferases (METTL3 and WTAP) [36, 37, 51]. Furthermore, it has been reported that LncRNA plays a biological role by regulating demethylase FTO, m6A reader YTHDC2, ALKBH5, mRNA stability, miRNA sponge, and so on [18, 52–54]. Finally, we predicted the top 10 miRNAs and top 10 mRNAs of the four key LncRNAs and constructed a network of LncRNA-miRNA-mRNA.

## 5. Conclusion

In this study, the expression level and the m6A modification level of LncRNAs in preoperative and postoperative samples were compared. Finally, we screened out three key LncRNAs (LncRNA FOXD2-AS1, LncRNA FAR2, and AL357060.1) which may regulate the angiogenesis process through autophagy pathway, PPAR signaling pathway, and MAPK signaling pathway and ultimately promote CIRI. The sample size of this study is limited, and a larger sample size may make the results more credible. In addition, this study has verified the expression level and methylation level of key LncRNAs preliminarily. We will explore the function of the key LncRNAs at the cellular level and in vivo animal level and verify the signaling pathway of bioinformatics analysis in the following research. The results of this project may provide an idea for the diagnosis and treatment of CIRI.

## Figures and Tables

**Table 1:** Primer sequences for key LncRNA and GAPDH.

**Gene**	**Sequence**	
GAPDH	F: 5′GGGAAACTGTGGCGTGAT3′	R: 5′GAGTGGGTGTCGCTGTTGA3′
LncRNA FOXD2-AS1	F: 5′AGTGGGGAATGAGGATGGGT3′	R: 5′CGGCGTGTAATTGGTAGGAG3′
LncRNA FAR2	F: 5′GGAAGGAGCAGGATTTAGAGG3′	R: 5′TAAGCCAGCAGACGGTGATAG3′
LncRNA AL357060.1	F: 5′GGATTGGGAGGAAGAATGAAAA3′	R: 5′CAGGGAGTGAAGAGAGGTGGTT3′
LncRNA LINC02431	F: 5′CCAAACCTGCACCCCTAGTC3′	R: 5′AGGGATTAGGGGCAACATCC3′

## Data Availability

The data that support the findings of this study are available.

## Nomenclature

AIS: acute ischemia stroke
BP: biological process
CC: cellular component
ceRNA: competing endogenous RNA
CIRI: cerebral ischemia/reperfusion injury
CTA: computed tomography angiography
FC: fold change
GO: Gene Ontology
I/R: ischemia/reperfusion
KEGG: Kyoto Encyclopedia of Genes and Genomes
m6A: N6-methyladenosine
MF: molecular function
NORAD: noncoding RNA activated by DNA damage
OGD/R-induced: oxygen-glucose deprivation/reoxygenation induced

## Supporting Information

Supporting Information Additional supporting information can be found online in the Supporting Information section. Table S1. Pearson correlation analysis between upregulated LncRNA and mRNA. Table S2. Pearson correlation analysis between downregulated LncRNA and mRNA. Table S3. Pearson correlation analysis between hypermethylated LncRNA and mRNA. Table S4. Pearson correlation analysis between hypomethylated LncRNA and mRNA.
