# Supplementary material for: M6A Modification and Transcription Analysis of LncRNA in Cerebral Ischemia/Reperfusion Injury
Source: Int J Genomics. 2024 Oct 5;2024:4596974. doi: 10.1155/2024/4596974 (PMC11470819; doi:10.1155/2024/4596974)
Supplement: Supporting Information — Additional supporting information can be found online in the Supporting Information section. Table S1. Pearson correlation analysis between upregulated LncRNA and mRNA. Table S2. Pearson correlation analysis between downregulated LncRNA and mRNA. Table S3. Pearson correlation analysis between hypermethylated LncRNA and mRNA. Table S4. Pearson correlation analysis between hypomethylated LncRNA and mRNA. [file 4596974.f1.zip › Table S1.docx]

**Table S1 Pearson correlation analysis between up-regulated LncRNA and mRNA**

| LncRNA | mRNA | P | R |
| --- | --- | --- | --- |
| ENST00000497004 | TMEM132B | 0.008141 | 0.991859 |
| ENST00000497004 | EEF1D | 5.65E-04 | -0.99944 |
| ENST00000497004 | TBKBP1 | 0.00318 | -0.99682 |
| ENST00000497004 | WT1 | 0.009226 | -0.99077 |
| ENST00000497004 | CATG00000039284.1 | 0.008605 | -0.99139 |
| ENST00000497004 | SCAMP3 | 0.004643 | -0.99536 |
| ENST00000497004 | PDE3B | 0.007847 | -0.99215 |
| ENST00000497004 | TMEM132B | 0.008141 | 0.991859 |
| ENST00000497004 | EEF1D | 5.65E-04 | -0.99944 |
| ENST00000497004 | TBKBP1 | 0.00318 | -0.99682 |
| ENST00000497004 | WT1 | 0.009226 | -0.99077 |
| ENST00000497004 | CATG00000039284.1 | 0.008605 | -0.99139 |
| ENST00000497004 | SCAMP3 | 0.004643 | -0.99536 |
| ENST00000497004 | PDE3B | 0.007847 | -0.99215 |
| ENST00000497004 | TMEM132B | 0.008141 | 0.991859 |
| ENST00000497004 | EEF1D | 5.65E-04 | -0.99944 |
| ENST00000497004 | TBKBP1 | 0.00318 | -0.99682 |
| ENST00000497004 | WT1 | 0.009226 | -0.99077 |
| ENST00000497004 | CATG00000039284.1 | 0.008605 | -0.99139 |
| ENST00000497004 | SCAMP3 | 0.004643 | -0.99536 |
| ENST00000497004 | PDE3B | 0.007847 | -0.99215 |
| ENST00000497004 | TMEM132B | 0.008141 | 0.991859 |
| ENST00000497004 | EEF1D | 5.65E-04 | -0.99944 |
| ENST00000497004 | TBKBP1 | 0.00318 | -0.99682 |
| ENST00000497004 | WT1 | 0.009226 | -0.99077 |
| ENST00000497004 | CATG00000039284.1 | 0.008605 | -0.99139 |
| ENST00000497004 | SCAMP3 | 0.004643 | -0.99536 |
| ENST00000497004 | PDE3B | 0.007847 | -0.99215 |
| ENST00000497004 | TMEM132B | 0.008141 | 0.991859 |
| ENST00000497004 | EEF1D | 5.65E-04 | -0.99944 |
| ENST00000497004 | TBKBP1 | 0.00318 | -0.99682 |
| ENST00000497004 | WT1 | 0.009226 | -0.99077 |
| ENST00000497004 | CATG00000039284.1 | 0.008605 | -0.99139 |
| ENST00000497004 | SCAMP3 | 0.004643 | -0.99536 |
| ENST00000497004 | PDE3B | 0.007847 | -0.99215 |
| ENST00000497004 | TMEM132B | 0.008141 | 0.991859 |
| ENST00000497004 | EEF1D | 5.65E-04 | -0.99944 |
| ENST00000497004 | TBKBP1 | 0.00318 | -0.99682 |
| ENST00000497004 | WT1 | 0.009226 | -0.99077 |
| ENST00000497004 | CATG00000039284.1 | 0.008605 | -0.99139 |
| ENST00000497004 | SCAMP3 | 0.004643 | -0.99536 |
| ENST00000497004 | PDE3B | 0.007847 | -0.99215 |
| ENST00000497004 | TMEM132B | 0.008141 | 0.991859 |
| ENST00000497004 | EEF1D | 5.65E-04 | -0.99944 |
| ENST00000497004 | TBKBP1 | 0.00318 | -0.99682 |
| ENST00000497004 | WT1 | 0.009226 | -0.99077 |
| ENST00000497004 | CATG00000039284.1 | 0.008605 | -0.99139 |
| ENST00000497004 | SCAMP3 | 0.004643 | -0.99536 |
| ENST00000497004 | PDE3B | 0.007847 | -0.99215 |
| ENST00000497004 | TMEM132B | 0.008141 | 0.991859 |
| ENST00000497004 | EEF1D | 5.65E-04 | -0.99944 |
| ENST00000497004 | TBKBP1 | 0.00318 | -0.99682 |
| ENST00000497004 | WT1 | 0.009226 | -0.99077 |
| ENST00000497004 | CATG00000039284.1 | 0.008605 | -0.99139 |
| ENST00000497004 | SCAMP3 | 0.004643 | -0.99536 |
| ENST00000497004 | PDE3B | 0.007847 | -0.99215 |
| ENST00000497004 | TMEM132B | 0.008141 | 0.991859 |
| ENST00000497004 | EEF1D | 5.65E-04 | -0.99944 |
| ENST00000497004 | TBKBP1 | 0.00318 | -0.99682 |
| ENST00000497004 | WT1 | 0.009226 | -0.99077 |
| ENST00000497004 | CATG00000039284.1 | 0.008605 | -0.99139 |
| ENST00000497004 | SCAMP3 | 0.004643 | -0.99536 |
| ENST00000497004 | PDE3B | 0.007847 | -0.99215 |
| ENST00000497004 | TMEM132B | 0.008141 | 0.991859 |
| ENST00000497004 | EEF1D | 5.65E-04 | -0.99944 |
| ENST00000497004 | TBKBP1 | 0.00318 | -0.99682 |
| ENST00000497004 | WT1 | 0.009226 | -0.99077 |
| ENST00000497004 | CATG00000039284.1 | 0.008605 | -0.99139 |
| ENST00000497004 | SCAMP3 | 0.004643 | -0.99536 |
| ENST00000497004 | PDE3B | 0.007847 | -0.99215 |
| ENST00000497004 | TMEM132B | 0.008141 | 0.991859 |
| ENST00000497004 | EEF1D | 5.65E-04 | -0.99944 |
| ENST00000497004 | TBKBP1 | 0.00318 | -0.99682 |
| ENST00000497004 | WT1 | 0.009226 | -0.99077 |
| ENST00000497004 | CATG00000039284.1 | 0.008605 | -0.99139 |
| ENST00000497004 | SCAMP3 | 0.004643 | -0.99536 |
| ENST00000497004 | PDE3B | 0.007847 | -0.99215 |
| ENST00000497004 | TMEM132B | 0.008141 | 0.991859 |
| ENST00000497004 | EEF1D | 5.65E-04 | -0.99944 |
| ENST00000497004 | TBKBP1 | 0.00318 | -0.99682 |
| ENST00000497004 | WT1 | 0.009226 | -0.99077 |
| ENST00000497004 | CATG00000039284.1 | 0.008605 | -0.99139 |
| ENST00000497004 | SCAMP3 | 0.004643 | -0.99536 |
| ENST00000497004 | PDE3B | 0.007847 | -0.99215 |
| ENST00000497004 | TMEM132B | 0.008141 | 0.991859 |
| ENST00000497004 | EEF1D | 5.65E-04 | -0.99944 |
| ENST00000497004 | TBKBP1 | 0.00318 | -0.99682 |
| ENST00000497004 | WT1 | 0.009226 | -0.99077 |
| ENST00000497004 | CATG00000039284.1 | 0.008605 | -0.99139 |
| ENST00000497004 | SCAMP3 | 0.004643 | -0.99536 |
| ENST00000497004 | PDE3B | 0.007847 | -0.99215 |
| ENST00000497004 | TMEM132B | 0.008141 | 0.991859 |
| ENST00000497004 | EEF1D | 5.65E-04 | -0.99944 |
| ENST00000497004 | TBKBP1 | 0.00318 | -0.99682 |
| ENST00000497004 | WT1 | 0.009226 | -0.99077 |
| ENST00000497004 | CATG00000039284.1 | 0.008605 | -0.99139 |
| ENST00000497004 | SCAMP3 | 0.004643 | -0.99536 |
| ENST00000497004 | PDE3B | 0.007847 | -0.99215 |
| T109858 | SLC24A5 | 0.004679 | -0.99532 |
| T109858 | TCOF1 | 0.004775 | 0.995225 |
| T109858 | MYD88 | 3.93E-04 | -0.99961 |
| T109858 | FSCB | 0.002003 | 0.997997 |
| T109858 | SAMD13 | 0.003275 | 0.996725 |
| T109858 | PPP5C | 7.14E-04 | 0.999286 |
| T109858 | IGSF9B | 0.009793 | 0.990207 |
| T109858 | PURG | 0.009476 | 0.990524 |
| T109858 | CENPO | 0.009717 | 0.990283 |
| T109858 | YPEL5 | 0.007598 | 0.992402 |
| TCONS_00028210 | ASAP1 | 0.008395 | -0.9916 |
| TCONS_00028210 | SUPV3L1 | 0.006687 | 0.993313 |
| TCONS_00028210 | GPR156 | 0.00711 | -0.99289 |
| TCONS_00028210 | UBE2V1 | 0.00278 | 0.99722 |
| TCONS_00028210 | FTCD | 0.003454 | -0.99655 |
| TCONS_00028210 | ZNF77 | 0.002858 | 0.997142 |
| ENST00000457964 | ECSIT | 0.005027 | -0.99497 |
| ENST00000457964 | CATG00000056264.1 | 2.91E-04 | -0.99971 |
| ENST00000457964 | CATG00000003494.1 | 0.005419 | -0.99458 |
| ENST00000457964 | SSBP2 | 0.00148 | -0.99852 |
| ENST00000457964 | CHST8 | 0.004318 | -0.99568 |
| ENST00000457964 | CLEC4G | 8.17E-04 | -0.99918 |
| ENST00000457964 | TMC2 | 5.61E-05 | -0.99994 |
| ENST00000457964 | CLK3 | 0.003362 | -0.99664 |
| ENST00000457964 | TPRX1 | 0.001091 | -0.99891 |
| ENST00000457964 | CLCNKB | 0.004329 | -0.99567 |
| ENST00000457964 | INO80B | 0.005855 | -0.99414 |
| ENST00000457964 | PPTC7 | 0.005784 | 0.994216 |
| ENST00000457964 | GABPA | 0.008938 | -0.99106 |
| ENST00000457964 | CIB1 | 0.005465 | 0.994535 |
| ENST00000457964 | ECSIT | 0.005027 | -0.99497 |
| ENST00000457964 | CATG00000056264.1 | 2.91E-04 | -0.99971 |
| ENST00000457964 | CATG00000003494.1 | 0.005419 | -0.99458 |
| ENST00000457964 | SSBP2 | 0.00148 | -0.99852 |
| ENST00000457964 | CHST8 | 0.004318 | -0.99568 |
| ENST00000457964 | CLEC4G | 8.17E-04 | -0.99918 |
| ENST00000457964 | TMC2 | 5.61E-05 | -0.99994 |
| ENST00000457964 | CLK3 | 0.003362 | -0.99664 |
| ENST00000457964 | TPRX1 | 0.001091 | -0.99891 |
| ENST00000457964 | CLCNKB | 0.004329 | -0.99567 |
| ENST00000457964 | INO80B | 0.005855 | -0.99414 |
| ENST00000457964 | PPTC7 | 0.005784 | 0.994216 |
| ENST00000457964 | GABPA | 0.008938 | -0.99106 |
| ENST00000457964 | CIB1 | 0.005465 | 0.994535 |
| ENST00000457964 | ECSIT | 0.005027 | -0.99497 |
| ENST00000457964 | CATG00000056264.1 | 2.91E-04 | -0.99971 |
| ENST00000457964 | CATG00000003494.1 | 0.005419 | -0.99458 |
| ENST00000457964 | SSBP2 | 0.00148 | -0.99852 |
| ENST00000457964 | CHST8 | 0.004318 | -0.99568 |
| ENST00000457964 | CLEC4G | 8.17E-04 | -0.99918 |
| ENST00000457964 | TMC2 | 5.61E-05 | -0.99994 |
| ENST00000457964 | CLK3 | 0.003362 | -0.99664 |
| ENST00000457964 | TPRX1 | 0.001091 | -0.99891 |
| ENST00000457964 | CLCNKB | 0.004329 | -0.99567 |
| ENST00000457964 | INO80B | 0.005855 | -0.99414 |
| ENST00000457964 | PPTC7 | 0.005784 | 0.994216 |
| ENST00000457964 | GABPA | 0.008938 | -0.99106 |
| ENST00000457964 | CIB1 | 0.005465 | 0.994535 |
| ENST00000457964 | ECSIT | 0.005027 | -0.99497 |
| ENST00000457964 | CATG00000056264.1 | 2.91E-04 | -0.99971 |
| ENST00000457964 | CATG00000003494.1 | 0.005419 | -0.99458 |
| ENST00000457964 | SSBP2 | 0.00148 | -0.99852 |
| ENST00000457964 | CHST8 | 0.004318 | -0.99568 |
| ENST00000457964 | CLEC4G | 8.17E-04 | -0.99918 |
| ENST00000457964 | TMC2 | 5.61E-05 | -0.99994 |
| ENST00000457964 | CLK3 | 0.003362 | -0.99664 |
| ENST00000457964 | TPRX1 | 0.001091 | -0.99891 |
| ENST00000457964 | CLCNKB | 0.004329 | -0.99567 |
| ENST00000457964 | INO80B | 0.005855 | -0.99414 |
| ENST00000457964 | PPTC7 | 0.005784 | 0.994216 |
| ENST00000457964 | GABPA | 0.008938 | -0.99106 |
| ENST00000457964 | CIB1 | 0.005465 | 0.994535 |
| ENST00000531024 | MIEN1 | 0.00801 | -0.99199 |
| ENST00000531024 | ZMYM1 | 0.007458 | -0.99254 |
| ENST00000531024 | EPHB6 | 0.007712 | 0.992288 |
| ENST00000531024 | TSPAN32 | 0.001102 | 0.998898 |
| ENST00000531024 | CATG00000021838.1 | 0.003509 | -0.99649 |
| ENST00000531024 | C1QTNF9B | 0.001104 | -0.9989 |
| ENST00000531024 | UPP2 | 6.42E-04 | -0.99936 |
| ENST00000531024 | MIEN1 | 0.00801 | -0.99199 |
| ENST00000531024 | ZMYM1 | 0.007458 | -0.99254 |
| ENST00000531024 | EPHB6 | 0.007712 | 0.992288 |
| ENST00000531024 | TSPAN32 | 0.001102 | 0.998898 |
| ENST00000531024 | CATG00000021838.1 | 0.003509 | -0.99649 |
| ENST00000531024 | C1QTNF9B | 0.001104 | -0.9989 |
| ENST00000531024 | UPP2 | 6.42E-04 | -0.99936 |
| T201643 | INTS3 | 0.002479 | 0.997521 |
| T201643 | VPS41 | 0.002034 | 0.997966 |
| T201643 | TMEM43 | 8.89E-04 | -0.99911 |
| T201643 | INTS3 | 0.002479 | 0.997521 |
| T201643 | VPS41 | 0.002034 | 0.997966 |
| T201643 | TMEM43 | 8.89E-04 | -0.99911 |
| T201643 | INTS3 | 0.002479 | 0.997521 |
| T201643 | VPS41 | 0.002034 | 0.997966 |
| T201643 | TMEM43 | 8.89E-04 | -0.99911 |
| T201643 | INTS3 | 0.002479 | 0.997521 |
| T201643 | VPS41 | 0.002034 | 0.997966 |
| T201643 | TMEM43 | 8.89E-04 | -0.99911 |
| HBMT00001167636 | SLC13A4 | 0.005346 | 0.994654 |
| HBMT00001167636 | CATG00000039284.1 | 0.001546 | -0.99845 |
| T246626 | TNFRSF18 | 0.00953 | 0.99047 |
| T246626 | CATG00000092654.1 | 0.009151 | 0.990849 |
| T246626 | APBB3 | 0.008171 | -0.99183 |
| T246626 | SPATA33 | 0.008156 | 0.991844 |
| T246626 | PLEKHD1 | 0.003102 | 0.996898 |
| T246626 | MLLT1 | 0.004359 | -0.99564 |
| T246626 | DNAH6 | 0.00331 | -0.99669 |
| T246626 | HEATR9 | 0.004341 | -0.99566 |
| T246626 | TNRC6B | 0.009897 | -0.9901 |
| ENST00000556583 | MYH7 | 3.75E-04 | -0.99962 |
| ENST00000556583 | NABP2 | 0.001521 | 0.998479 |
| ENST00000556583 | CATG00000086563.1 | 0.008287 | 0.991713 |
| ENST00000556583 | TCF23 | 0.005834 | -0.99417 |
| ENST00000556583 | FOXR1 | 0.008088 | -0.99191 |
| ENST00000556583 | MYH7 | 3.75E-04 | -0.99962 |
| ENST00000556583 | NABP2 | 0.001521 | 0.998479 |
| ENST00000556583 | CATG00000086563.1 | 0.008287 | 0.991713 |
| ENST00000556583 | TCF23 | 0.005834 | -0.99417 |
| ENST00000556583 | FOXR1 | 0.008088 | -0.99191 |
| ENST00000556583 | MYH7 | 3.75E-04 | -0.99962 |
| ENST00000556583 | NABP2 | 0.001521 | 0.998479 |
| ENST00000556583 | CATG00000086563.1 | 0.008287 | 0.991713 |
| ENST00000556583 | TCF23 | 0.005834 | -0.99417 |
| ENST00000556583 | FOXR1 | 0.008088 | -0.99191 |
| ENST00000556583 | MYH7 | 3.75E-04 | -0.99962 |
| ENST00000556583 | NABP2 | 0.001521 | 0.998479 |
| ENST00000556583 | CATG00000086563.1 | 0.008287 | 0.991713 |
| ENST00000556583 | TCF23 | 0.005834 | -0.99417 |
| ENST00000556583 | FOXR1 | 0.008088 | -0.99191 |
| ENST00000556583 | MYH7 | 3.75E-04 | -0.99962 |
| ENST00000556583 | NABP2 | 0.001521 | 0.998479 |
| ENST00000556583 | CATG00000086563.1 | 0.008287 | 0.991713 |
| ENST00000556583 | TCF23 | 0.005834 | -0.99417 |
| ENST00000556583 | FOXR1 | 0.008088 | -0.99191 |
| ENST00000556583 | MYH7 | 3.75E-04 | -0.99962 |
| ENST00000556583 | NABP2 | 0.001521 | 0.998479 |
| ENST00000556583 | CATG00000086563.1 | 0.008287 | 0.991713 |
| ENST00000556583 | TCF23 | 0.005834 | -0.99417 |
| ENST00000556583 | FOXR1 | 0.008088 | -0.99191 |
| ENST00000556583 | MYH7 | 3.75E-04 | -0.99962 |
| ENST00000556583 | NABP2 | 0.001521 | 0.998479 |
| ENST00000556583 | CATG00000086563.1 | 0.008287 | 0.991713 |
| ENST00000556583 | TCF23 | 0.005834 | -0.99417 |
| ENST00000556583 | FOXR1 | 0.008088 | -0.99191 |
| ENST00000556583 | MYH7 | 3.75E-04 | -0.99962 |
| ENST00000556583 | NABP2 | 0.001521 | 0.998479 |
| ENST00000556583 | CATG00000086563.1 | 0.008287 | 0.991713 |
| ENST00000556583 | TCF23 | 0.005834 | -0.99417 |
| ENST00000556583 | FOXR1 | 0.008088 | -0.99191 |
| ENST00000556583 | MYH7 | 3.75E-04 | -0.99962 |
| ENST00000556583 | NABP2 | 0.001521 | 0.998479 |
| ENST00000556583 | CATG00000086563.1 | 0.008287 | 0.991713 |
| ENST00000556583 | TCF23 | 0.005834 | -0.99417 |
| ENST00000556583 | FOXR1 | 0.008088 | -0.99191 |
| ENST00000557019 | WDR90 | 0.003444 | -0.99656 |
| ENST00000557019 | SORCS2 | 0.004984 | -0.99502 |
| ENST00000557019 | NUP54 | 0.006697 | -0.9933 |
| ENST00000557019 | KIF17 | 1.27E-04 | -0.99987 |
| ENST00000557019 | FTSJ1 | 0.007196 | -0.9928 |
| ENST00000557019 | CLCN5 | 0.009962 | -0.99004 |
| HBMT00001006885 | TAGAP | 0.00759 | -0.99241 |
| HBMT00001006885 | NKG7 | 0.001169 | -0.99883 |
| HBMT00001006885 | TAGAP | 0.00759 | -0.99241 |
| HBMT00001006885 | NKG7 | 0.001169 | -0.99883 |
| HBMT00001006885 | TAGAP | 0.00759 | -0.99241 |
| HBMT00001006885 | NKG7 | 0.001169 | -0.99883 |
| HBMT00001006885 | TAGAP | 0.00759 | -0.99241 |
| HBMT00001006885 | NKG7 | 0.001169 | -0.99883 |
| ENST00000427820 | GNL1 | 0.003636 | 0.996364 |
| ENST00000427820 | DEAF1 | 0.008377 | -0.99162 |
| ENST00000427820 | GPR25 | 0.008203 | -0.9918 |
| ENST00000427820 | VRK3 | 0.008659 | -0.99134 |
| ENST00000427820 | BLCAP | 0.00864 | -0.99136 |
| ENST00000427820 | FANCD2OS | 0.00443 | -0.99557 |
| ENST00000427820 | CATG00000023328.1 | 0.002607 | 0.997393 |
| ENST00000427820 | EFEMP2 | 0.002325 | 0.997675 |
| ENST00000427820 | OR4X1 | 2.03E-04 | -0.9998 |
| ENST00000427820 | MAP3K5 | 0.00195 | -0.99805 |
| ENST00000427820 | HLA-F | 0.001726 | -0.99827 |
| ENST00000427820 | CATG00000024701.1 | 6.65E-04 | -0.99933 |
| ENCT00000221798 | TAS2R42 | 0.004051 | -0.99595 |
| ENCT00000221798 | GRINA | 0.00982 | -0.99018 |
| ENCT00000221798 | PPP3CC | 0.007661 | 0.992339 |
| ENCT00000221798 | PSRC1 | 0.004734 | 0.995266 |
| ENCT00000221798 | ABCB8 | 0.003286 | -0.99671 |
| ENCT00000221798 | LILRB1 | 0.008563 | 0.991437 |
| ENCT00000221798 | RASL10A | 0.006069 | 0.993931 |
| ENCT00000221798 | LTA4H | 1.69E-04 | 0.999831 |
| ENCT00000221798 | TUT4 | 5.79E-04 | 0.999421 |
| ENCT00000221798 | WDR33 | 0.004349 | -0.99565 |
| ENCT00000221798 | CATG00000053512.1 | 0.009449 | 0.990551 |
| ENCT00000221798 | NFX1 | 0.006468 | 0.993532 |
| ENCT00000221798 | CATG00000063823.1 | 0.007832 | -0.99217 |
| ENCT00000221798 | PEA15 | 0.006798 | -0.9932 |
| ENCT00000221798 | PSMD4 | 0.006661 | 0.993339 |
| ENCT00000221798 | SLC12A3 | 0.007679 | 0.992321 |
| ENCT00000221798 | WNT8B | 0.007594 | -0.99241 |
| ENCT00000221798 | ADAM28 | 0.006563 | 0.993437 |
| ENCT00000221798 | UNC5C | 0.006578 | 0.993422 |
| ENCT00000221798 | AGO2 | 0.009952 | -0.99005 |
| ENCT00000221798 | TRIM47 | 0.004673 | -0.99533 |
| ENCT00000221798 | CATG00000087047.1 | 1.56E-04 | -0.99984 |
| ENCT00000221798 | AC109583.1 | 0.004918 | -0.99508 |
| ENCT00000221798 | CFAP410 | 0.009873 | -0.99013 |
| ENCT00000221798 | HPS1 | 0.009979 | -0.99002 |
| ENCT00000221798 | RAD51AP2 | 0.001048 | 0.998952 |
| ENST00000609792 | TNFRSF18 | 9.14E-04 | 0.999086 |
| ENST00000609792 | SPATA33 | 0.001922 | 0.998078 |
| ENST00000609792 | KLHL35 | 6.88E-04 | 0.999312 |
| ENST00000609792 | MT1F | 0.002868 | 0.997132 |
| ENST00000609792 | WWP1 | 0.001747 | 0.998253 |
| ENST00000609792 | APOBR | 0.003534 | 0.996466 |
| ENST00000609792 | TNXB | 0.002378 | -0.99762 |
| ENST00000609792 | KYAT3 | 0.00324 | -0.99676 |
| ENST00000609792 | HEATR9 | 0.006157 | -0.99384 |
| ENST00000609792 | PAQR5 | 0.002405 | -0.99759 |
| ENST00000609792 | TNRC6B | 0.002801 | -0.9972 |
| ENST00000609792 | ATG4C | 0.00421 | -0.99579 |
| TCONS_00011503 | SYBU | 0.008412 | -0.99159 |
| TCONS_00011503 | EXOC2 | 0.005874 | 0.994126 |
| TCONS_00011503 | CHSY3 | 4.95E-04 | 0.999505 |
| TCONS_00011503 | GLIPR1L2 | 0.001821 | 0.998179 |
| TCONS_00011503 | IKBKE | 4.31E-04 | 0.999569 |
| TCONS_00011503 | KIF21B | 0.007089 | -0.99291 |
| TCONS_00011503 | CATG00000113928.1 | 0.00305 | -0.99695 |
| TCONS_00011503 | KCNK16 | 7.18E-04 | -0.99928 |
| TCONS_00011503 | MMP24 | 0.002848 | -0.99715 |
| TCONS_00011503 | FANCE | 0.001781 | -0.99822 |
| TCONS_00011503 | ZNF624 | 0.007032 | 0.992968 |
| TCONS_00013557 | LDLRAD4 | 6.80E-04 | -0.99932 |
| TCONS_00013557 | GSTM5 | 0.009971 | 0.990029 |
| TCONS_00013557 | IFT80 | 0.009508 | -0.99049 |
| TCONS_00013557 | DLEC1 | 0.002628 | 0.997372 |
| ENST00000509201 | ZNF185 | 0.008858 | -0.99114 |
| ENST00000509201 | FAM169B | 0.003445 | -0.99655 |
| ENST00000509201 | GPR82 | 1.95E-04 | -0.9998 |
| ENST00000509201 | NKG7 | 0.002847 | 0.997153 |
| ENST00000456384 | PZP | 0.00257 | -0.99743 |
| ENST00000456384 | CFAP77 | 0.005953 | -0.99405 |
| ENST00000456384 | CLEC4D | 0.007261 | -0.99274 |
| ENST00000456384 | ALS2CR12 | 0.009064 | 0.990936 |
| ENST00000610062 | SCAMP2 | 0.006835 | -0.99317 |
| ENST00000610062 | SLC24A5 | 0.004841 | 0.995159 |
| ENST00000610062 | TCOF1 | 0.009245 | -0.99075 |
| ENST00000610062 | TMC5 | 0.009449 | -0.99055 |
| ENST00000610062 | XYLT2 | 0.009385 | -0.99062 |
| ENST00000610062 | FSCB | 0.004047 | -0.99595 |
| ENST00000610062 | SAMD13 | 0.008992 | -0.99101 |
| ENST00000610062 | IKZF4 | 0.001102 | -0.9989 |
| ENST00000610062 | MYH3 | 0.00234 | 0.99766 |
| ENST00000610062 | KCTD1 | 0.007429 | -0.99257 |
| ENST00000610062 | PURG | 0.002774 | -0.99723 |
| ENST00000610062 | CACNG6 | 0.004037 | -0.99596 |
| ENST00000610062 | PAN3 | 0.0041 | 0.9959 |
| ENST00000610062 | INKA2 | 0.004858 | -0.99514 |
| ENST00000610062 | RAMP1 | 0.007545 | 0.992455 |
| ENST00000610062 | PARVG | 0.005722 | -0.99428 |
| ENST00000610062 | YPEL5 | 3.53E-04 | -0.99965 |
| ENST00000412811 | CATG00000028653.1 | 0.008545 | 0.991455 |
| ENST00000412811 | CCDC114 | 7.36E-04 | 0.999264 |
| ENST00000412811 | C1QTNF8 | 0.004753 | 0.995247 |
| ENST00000412811 | LHFPL6 | 0.002164 | -0.99784 |
| ENST00000412811 | BVES | 0.007719 | -0.99228 |
| ENST00000412811 | ARHGEF33 | 0.001051 | -0.99895 |
| ENST00000412811 | ASPRV1 | 0.008837 | 0.991163 |
| ENST00000625390 | CATG00000061038.1 | 0.008516 | 0.991484 |
| ENST00000625390 | MAPKBP1 | 0.007458 | -0.99254 |
| ENST00000625390 | CD19 | 0.002354 | 0.997646 |
| ENST00000625390 | RELL1 | 0.006534 | 0.993466 |
| ENST00000625390 | AC093157.1 | 0.006174 | 0.993826 |
| ENST00000625390 | ZPR1 | 0.001134 | -0.99887 |
| ENST00000625390 | OR10R2 | 0.001383 | -0.99862 |
| ENST00000625390 | DDIT4 | 0.003569 | 0.996431 |
| ENST00000625390 | CATG00000061038.1 | 0.008516 | 0.991484 |
| ENST00000625390 | MAPKBP1 | 0.007458 | -0.99254 |
| ENST00000625390 | CD19 | 0.002354 | 0.997646 |
| ENST00000625390 | RELL1 | 0.006534 | 0.993466 |
| ENST00000625390 | AC093157.1 | 0.006174 | 0.993826 |
| ENST00000625390 | ZPR1 | 0.001134 | -0.99887 |
| ENST00000625390 | OR10R2 | 0.001383 | -0.99862 |
| ENST00000625390 | DDIT4 | 0.003569 | 0.996431 |
| ENST00000625390 | CATG00000061038.1 | 0.008516 | 0.991484 |
| ENST00000625390 | MAPKBP1 | 0.007458 | -0.99254 |
| ENST00000625390 | CD19 | 0.002354 | 0.997646 |
| ENST00000625390 | RELL1 | 0.006534 | 0.993466 |
| ENST00000625390 | AC093157.1 | 0.006174 | 0.993826 |
| ENST00000625390 | ZPR1 | 0.001134 | -0.99887 |
| ENST00000625390 | OR10R2 | 0.001383 | -0.99862 |
| ENST00000625390 | DDIT4 | 0.003569 | 0.996431 |
| ENST00000625390 | CATG00000061038.1 | 0.008516 | 0.991484 |
| ENST00000625390 | MAPKBP1 | 0.007458 | -0.99254 |
| ENST00000625390 | CD19 | 0.002354 | 0.997646 |
| ENST00000625390 | RELL1 | 0.006534 | 0.993466 |
| ENST00000625390 | AC093157.1 | 0.006174 | 0.993826 |
| ENST00000625390 | ZPR1 | 0.001134 | -0.99887 |
| ENST00000625390 | OR10R2 | 0.001383 | -0.99862 |
| ENST00000625390 | DDIT4 | 0.003569 | 0.996431 |
| ENST00000625390 | CATG00000061038.1 | 0.008516 | 0.991484 |
| ENST00000625390 | MAPKBP1 | 0.007458 | -0.99254 |
| ENST00000625390 | CD19 | 0.002354 | 0.997646 |
| ENST00000625390 | RELL1 | 0.006534 | 0.993466 |
| ENST00000625390 | AC093157.1 | 0.006174 | 0.993826 |
| ENST00000625390 | ZPR1 | 0.001134 | -0.99887 |
| ENST00000625390 | OR10R2 | 0.001383 | -0.99862 |
| ENST00000625390 | DDIT4 | 0.003569 | 0.996431 |
| ENST00000533945 | TRMT11 | 0.00709 | 0.99291 |
| ENST00000533945 | MYLK3 | 0.002146 | 0.997854 |
| ENST00000533945 | FBLN2 | 0.006128 | 0.993872 |
| ENST00000533945 | CYP21A2 | 0.008885 | -0.99111 |
| ENST00000533945 | SLC39A12 | 0.001594 | -0.99841 |
| ENST00000533945 | SYNGR3 | 0.004854 | -0.99515 |
| ENST00000533945 | EDDM3A | 0.006409 | -0.99359 |
| ENST00000521863 | ZNF185 | 0.005733 | 0.994267 |
| ENST00000521863 | NFATC2 | 0.009492 | -0.99051 |
| ENST00000521863 | FAM169B | 0.00576 | 0.99424 |
| ENST00000521863 | IFNA6 | 0.009947 | 0.990053 |
| ENST00000620208 | PPP1CB | 0.001517 | -0.99848 |
| ENST00000620208 | PGM5 | 0.001094 | 0.998906 |
| ENST00000620208 | CATG00000107158.1 | 2.68E-05 | -0.99997 |
| ENST00000620208 | CATG00000110054.1 | 0.008357 | -0.99164 |
| ENST00000620208 | SPRR1A | 0.007634 | -0.99237 |
| ENST00000620208 | RCSD1 | 0.001297 | 0.998703 |
| ENST00000620208 | LY6G6C | 0.002503 | 0.997497 |
| ENST00000620208 | AOAH | 0.003798 | -0.9962 |
| ENST00000620208 | MOK | 0.00472 | 0.99528 |
| ENST00000620208 | CHI3L1 | 0.002667 | -0.99733 |
| ENST00000620208 | CATG00000020281.1 | 0.004549 | 0.995451 |
| ENST00000620208 | PPP1CB | 0.001517 | -0.99848 |
| ENST00000620208 | PGM5 | 0.001094 | 0.998906 |
| ENST00000620208 | CATG00000107158.1 | 2.68E-05 | -0.99997 |
| ENST00000620208 | CATG00000110054.1 | 0.008357 | -0.99164 |
| ENST00000620208 | SPRR1A | 0.007634 | -0.99237 |
| ENST00000620208 | RCSD1 | 0.001297 | 0.998703 |
| ENST00000620208 | LY6G6C | 0.002503 | 0.997497 |
| ENST00000620208 | AOAH | 0.003798 | -0.9962 |
| ENST00000620208 | MOK | 0.00472 | 0.99528 |
| ENST00000620208 | CHI3L1 | 0.002667 | -0.99733 |
| ENST00000620208 | CATG00000020281.1 | 0.004549 | 0.995451 |
| ENST00000620208 | PPP1CB | 0.001517 | -0.99848 |
| ENST00000620208 | PGM5 | 0.001094 | 0.998906 |
| ENST00000620208 | CATG00000107158.1 | 2.68E-05 | -0.99997 |
| ENST00000620208 | CATG00000110054.1 | 0.008357 | -0.99164 |
| ENST00000620208 | SPRR1A | 0.007634 | -0.99237 |
| ENST00000620208 | RCSD1 | 0.001297 | 0.998703 |
| ENST00000620208 | LY6G6C | 0.002503 | 0.997497 |
| ENST00000620208 | AOAH | 0.003798 | -0.9962 |
| ENST00000620208 | MOK | 0.00472 | 0.99528 |
| ENST00000620208 | CHI3L1 | 0.002667 | -0.99733 |
| ENST00000620208 | CATG00000020281.1 | 0.004549 | 0.995451 |
| ENST00000620208 | PPP1CB | 0.001517 | -0.99848 |
| ENST00000620208 | PGM5 | 0.001094 | 0.998906 |
| ENST00000620208 | CATG00000107158.1 | 2.68E-05 | -0.99997 |
| ENST00000620208 | CATG00000110054.1 | 0.008357 | -0.99164 |
| ENST00000620208 | SPRR1A | 0.007634 | -0.99237 |
| ENST00000620208 | RCSD1 | 0.001297 | 0.998703 |
| ENST00000620208 | LY6G6C | 0.002503 | 0.997497 |
| ENST00000620208 | AOAH | 0.003798 | -0.9962 |
| ENST00000620208 | MOK | 0.00472 | 0.99528 |
| ENST00000620208 | CHI3L1 | 0.002667 | -0.99733 |
| ENST00000620208 | CATG00000020281.1 | 0.004549 | 0.995451 |
| ENST00000556033 | DNAJC6 | 2.42E-04 | -0.99976 |
| ENST00000556033 | MFAP3L | 8.93E-04 | -0.99911 |
| ENST00000556033 | CATG00000086946.1 | 0.005066 | -0.99493 |
| ENST00000556033 | LGALS1 | 0.007694 | 0.992306 |
| ENST00000556033 | SPACA9 | 4.28E-04 | -0.99957 |
| ENST00000556033 | CMBL | 0.006492 | -0.99351 |
| ENST00000556033 | ORM1 | 0.003805 | -0.9962 |
| ENST00000433121 | AX748369 | 0.004874 | -0.99513 |
| ENST00000433121 | PNPLA2 | 0.003832 | -0.99617 |
| ENST00000433121 | PDCD2L | 0.002948 | 0.997052 |
| ENST00000433121 | DRC1 | 0.00903 | -0.99097 |
| ENST00000433121 | FGD4 | 0.008229 | -0.99177 |
| ENST00000433121 | CATG00000108269.1 | 2.32E-04 | -0.99977 |
| ENST00000433121 | CYP27C1 | 0.004609 | -0.99539 |
| T146776 | CLEC17A | 0.004272 | -0.99573 |
| T146776 | SELENOP | 0.006508 | 0.993492 |
| T146776 | CATG00000023328.1 | 0.00834 | 0.99166 |
| T146776 | MAP3K5 | 0.002661 | -0.99734 |
| T146776 | CATG00000024701.1 | 0.006122 | -0.99388 |
| ENST00000629441 | E4F1 | 0.003234 | -0.99677 |
| ENST00000629441 | ZNF736 | 0.006974 | -0.99303 |
| ENST00000629441 | RNASEH2A | 0.001573 | -0.99843 |
| ENST00000629441 | NDUFC1 | 0.007995 | -0.99201 |
| ENST00000629441 | E4F1 | 0.003234 | -0.99677 |
| ENST00000629441 | ZNF736 | 0.006974 | -0.99303 |
| ENST00000629441 | RNASEH2A | 0.001573 | -0.99843 |
| ENST00000629441 | NDUFC1 | 0.007995 | -0.99201 |
| ENST00000629441 | E4F1 | 0.003234 | -0.99677 |
| ENST00000629441 | ZNF736 | 0.006974 | -0.99303 |
| ENST00000629441 | RNASEH2A | 0.001573 | -0.99843 |
| ENST00000629441 | NDUFC1 | 0.007995 | -0.99201 |
| ENST00000558755 | HIGD2A | 0.003403 | -0.9966 |
| ENST00000558755 | SYBU | 0.007096 | 0.992904 |
| ENST00000558755 | RFC2 | 0.009019 | -0.99098 |
| ENST00000558755 | CATG00000101330.1 | 0.001933 | -0.99807 |
| ENST00000558755 | ACAP3 | 0.005745 | 0.994255 |
| ENST00000558755 | KRTAP10-6 | 0.007387 | 0.992613 |
| ENST00000558755 | AP002990.1 | 0.007115 | -0.99289 |
| ENST00000558755 | SH3D21 | 0.006162 | 0.993838 |
| ENST00000558755 | PIKFYVE | 0.004178 | -0.99582 |
| ENST00000558755 | KIF21B | 0.008413 | 0.991587 |
| ENST00000558755 | TPRKB | 0.007554 | -0.99245 |
| ENST00000558755 | ARHGEF26 | 8.19E-04 | 0.999181 |
| ENST00000558755 | PHKA1 | 0.004181 | 0.995819 |
| ENST00000558755 | CATG00000026557.1 | 0.00231 | 0.99769 |
| ENST00000558755 | ELSPBP1 | 0.007094 | 0.992906 |
| ENST00000558755 | PSME1 | 8.18E-04 | -0.99918 |
| ENST00000558755 | CLRN1 | 0.006397 | 0.993603 |
| ENST00000558755 | DOCK1 | 0.005147 | 0.994853 |
| ENST00000558755 | DOHH | 0.001059 | 0.998941 |
| ENST00000558755 | HIGD2A | 0.003403 | -0.9966 |
| ENST00000558755 | SYBU | 0.007096 | 0.992904 |
| ENST00000558755 | RFC2 | 0.009019 | -0.99098 |
| ENST00000558755 | CATG00000101330.1 | 0.001933 | -0.99807 |
| ENST00000558755 | ACAP3 | 0.005745 | 0.994255 |
| ENST00000558755 | KRTAP10-6 | 0.007387 | 0.992613 |
| ENST00000558755 | AP002990.1 | 0.007115 | -0.99289 |
| ENST00000558755 | SH3D21 | 0.006162 | 0.993838 |
| ENST00000558755 | PIKFYVE | 0.004178 | -0.99582 |
| ENST00000558755 | KIF21B | 0.008413 | 0.991587 |
| ENST00000558755 | TPRKB | 0.007554 | -0.99245 |
| ENST00000558755 | ARHGEF26 | 8.19E-04 | 0.999181 |
| ENST00000558755 | PHKA1 | 0.004181 | 0.995819 |
| ENST00000558755 | CATG00000026557.1 | 0.00231 | 0.99769 |
| ENST00000558755 | ELSPBP1 | 0.007094 | 0.992906 |
| ENST00000558755 | PSME1 | 8.18E-04 | -0.99918 |
| ENST00000558755 | CLRN1 | 0.006397 | 0.993603 |
| ENST00000558755 | DOCK1 | 0.005147 | 0.994853 |
| ENST00000558755 | DOHH | 0.001059 | 0.998941 |
| ENST00000558755 | HIGD2A | 0.003403 | -0.9966 |
| ENST00000558755 | SYBU | 0.007096 | 0.992904 |
| ENST00000558755 | RFC2 | 0.009019 | -0.99098 |
| ENST00000558755 | CATG00000101330.1 | 0.001933 | -0.99807 |
| ENST00000558755 | ACAP3 | 0.005745 | 0.994255 |
| ENST00000558755 | KRTAP10-6 | 0.007387 | 0.992613 |
| ENST00000558755 | AP002990.1 | 0.007115 | -0.99289 |
| ENST00000558755 | SH3D21 | 0.006162 | 0.993838 |
| ENST00000558755 | PIKFYVE | 0.004178 | -0.99582 |
| ENST00000558755 | KIF21B | 0.008413 | 0.991587 |
| ENST00000558755 | TPRKB | 0.007554 | -0.99245 |
| ENST00000558755 | ARHGEF26 | 8.19E-04 | 0.999181 |
| ENST00000558755 | PHKA1 | 0.004181 | 0.995819 |
| ENST00000558755 | CATG00000026557.1 | 0.00231 | 0.99769 |
| ENST00000558755 | ELSPBP1 | 0.007094 | 0.992906 |
| ENST00000558755 | PSME1 | 8.18E-04 | -0.99918 |
| ENST00000558755 | CLRN1 | 0.006397 | 0.993603 |
| ENST00000558755 | DOCK1 | 0.005147 | 0.994853 |
| ENST00000558755 | DOHH | 0.001059 | 0.998941 |
| ENST00000558755 | HIGD2A | 0.003403 | -0.9966 |
| ENST00000558755 | SYBU | 0.007096 | 0.992904 |
| ENST00000558755 | RFC2 | 0.009019 | -0.99098 |
| ENST00000558755 | CATG00000101330.1 | 0.001933 | -0.99807 |
| ENST00000558755 | ACAP3 | 0.005745 | 0.994255 |
| ENST00000558755 | KRTAP10-6 | 0.007387 | 0.992613 |
| ENST00000558755 | AP002990.1 | 0.007115 | -0.99289 |
| ENST00000558755 | SH3D21 | 0.006162 | 0.993838 |
| ENST00000558755 | PIKFYVE | 0.004178 | -0.99582 |
| ENST00000558755 | KIF21B | 0.008413 | 0.991587 |
| ENST00000558755 | TPRKB | 0.007554 | -0.99245 |
| ENST00000558755 | ARHGEF26 | 8.19E-04 | 0.999181 |
| ENST00000558755 | PHKA1 | 0.004181 | 0.995819 |
| ENST00000558755 | CATG00000026557.1 | 0.00231 | 0.99769 |
| ENST00000558755 | ELSPBP1 | 0.007094 | 0.992906 |
| ENST00000558755 | PSME1 | 8.18E-04 | -0.99918 |
| ENST00000558755 | CLRN1 | 0.006397 | 0.993603 |
| ENST00000558755 | DOCK1 | 0.005147 | 0.994853 |
| ENST00000558755 | DOHH | 0.001059 | 0.998941 |
| ENST00000558755 | HIGD2A | 0.003403 | -0.9966 |
| ENST00000558755 | SYBU | 0.007096 | 0.992904 |
| ENST00000558755 | RFC2 | 0.009019 | -0.99098 |
| ENST00000558755 | CATG00000101330.1 | 0.001933 | -0.99807 |
| ENST00000558755 | ACAP3 | 0.005745 | 0.994255 |
| ENST00000558755 | KRTAP10-6 | 0.007387 | 0.992613 |
| ENST00000558755 | AP002990.1 | 0.007115 | -0.99289 |
| ENST00000558755 | SH3D21 | 0.006162 | 0.993838 |
| ENST00000558755 | PIKFYVE | 0.004178 | -0.99582 |
| ENST00000558755 | KIF21B | 0.008413 | 0.991587 |
| ENST00000558755 | TPRKB | 0.007554 | -0.99245 |
| ENST00000558755 | ARHGEF26 | 8.19E-04 | 0.999181 |
| ENST00000558755 | PHKA1 | 0.004181 | 0.995819 |
| ENST00000558755 | CATG00000026557.1 | 0.00231 | 0.99769 |
| ENST00000558755 | ELSPBP1 | 0.007094 | 0.992906 |
| ENST00000558755 | PSME1 | 8.18E-04 | -0.99918 |
| ENST00000558755 | CLRN1 | 0.006397 | 0.993603 |
| ENST00000558755 | DOCK1 | 0.005147 | 0.994853 |
| ENST00000558755 | DOHH | 0.001059 | 0.998941 |
| ENST00000558755 | HIGD2A | 0.003403 | -0.9966 |
| ENST00000558755 | SYBU | 0.007096 | 0.992904 |
| ENST00000558755 | RFC2 | 0.009019 | -0.99098 |
| ENST00000558755 | CATG00000101330.1 | 0.001933 | -0.99807 |
| ENST00000558755 | ACAP3 | 0.005745 | 0.994255 |
| ENST00000558755 | KRTAP10-6 | 0.007387 | 0.992613 |
| ENST00000558755 | AP002990.1 | 0.007115 | -0.99289 |
| ENST00000558755 | SH3D21 | 0.006162 | 0.993838 |
| ENST00000558755 | PIKFYVE | 0.004178 | -0.99582 |
| ENST00000558755 | KIF21B | 0.008413 | 0.991587 |
| ENST00000558755 | TPRKB | 0.007554 | -0.99245 |
| ENST00000558755 | ARHGEF26 | 8.19E-04 | 0.999181 |
| ENST00000558755 | PHKA1 | 0.004181 | 0.995819 |
| ENST00000558755 | CATG00000026557.1 | 0.00231 | 0.99769 |
| ENST00000558755 | ELSPBP1 | 0.007094 | 0.992906 |
| ENST00000558755 | PSME1 | 8.18E-04 | -0.99918 |
| ENST00000558755 | CLRN1 | 0.006397 | 0.993603 |
| ENST00000558755 | DOCK1 | 0.005147 | 0.994853 |
| ENST00000558755 | DOHH | 0.001059 | 0.998941 |
| ENST00000623418 | SIRPG | 0.007477 | 0.992523 |
| ENST00000623418 | AHSP | 0.00307 | 0.99693 |
| ENST00000623418 | AC092073.1 | 4.57E-04 | 0.999543 |
| ENST00000623418 | TGFBR3 | 4.82E-05 | 0.999952 |
| ENST00000623418 | SLC22A6 | 0.003737 | -0.99626 |
| ENST00000623418 | COA1 | 0.008895 | -0.9911 |
| ENST00000623418 | CATG00000034210.1 | 0.006808 | 0.993192 |
| ENST00000623418 | COA5 | 0.002228 | -0.99777 |
| ENST00000623418 | SLC6A5 | 0.008034 | 0.991966 |
| ENST00000623418 | RRBP1 | 4.44E-04 | -0.99956 |
| ENST00000623418 | MORN3 | 1.71E-04 | -0.99983 |
| ENST00000623418 | VPS13A | 0.002238 | 0.997762 |
| ENST00000623418 | FGF9 | 0.005311 | -0.99469 |
| ENST00000623418 | CAPN15 | 0.002062 | 0.997938 |
| ENST00000623418 | SIRPG | 0.007477 | 0.992523 |
| ENST00000623418 | AHSP | 0.00307 | 0.99693 |
| ENST00000623418 | AC092073.1 | 4.57E-04 | 0.999543 |
| ENST00000623418 | TGFBR3 | 4.82E-05 | 0.999952 |
| ENST00000623418 | SLC22A6 | 0.003737 | -0.99626 |
| ENST00000623418 | COA1 | 0.008895 | -0.9911 |
| ENST00000623418 | CATG00000034210.1 | 0.006808 | 0.993192 |
| ENST00000623418 | COA5 | 0.002228 | -0.99777 |
| ENST00000623418 | SLC6A5 | 0.008034 | 0.991966 |
| ENST00000623418 | RRBP1 | 4.44E-04 | -0.99956 |
| ENST00000623418 | MORN3 | 1.71E-04 | -0.99983 |
| ENST00000623418 | VPS13A | 0.002238 | 0.997762 |
| ENST00000623418 | FGF9 | 0.005311 | -0.99469 |
| ENST00000623418 | CAPN15 | 0.002062 | 0.997938 |
| TCONS_00020238 | ZC3H7B | 0.004577 | 0.995423 |
| TCONS_00020238 | CATG00000061038.1 | 0.001174 | -0.99883 |
| TCONS_00020238 | CD19 | 0.009943 | -0.99006 |
| TCONS_00020238 | OR10R2 | 0.006039 | 0.993961 |
| TCONS_00020238 | DDIT4 | 0.003011 | -0.99699 |
| ENST00000510302 | RPS7 | 0.007052 | 0.992948 |
| ENST00000510302 | ACOT12 | 0.004257 | 0.995743 |
| ENST00000510302 | PALM3 | 4.41E-04 | -0.99956 |
| ENST00000510302 | BCAS4 | 0.00125 | 0.99875 |
| ENST00000510302 | PELP1 | 0.002466 | 0.997534 |
| ENST00000510302 | CATG00000063823.1 | 0.007496 | 0.992504 |
| ENST00000510302 | PSMD4 | 0.007472 | -0.99253 |
| ENST00000510302 | OR8B4 | 0.007753 | -0.99225 |
| ENST00000510302 | MYH4 | 0.006735 | 0.993265 |
| ENST00000510302 | ADAM28 | 0.007247 | -0.99275 |
| ENST00000510302 | KNCN | 1.52E-04 | 0.999848 |
| ENST00000510302 | FANCL | 0.008705 | 0.991295 |
| ENST00000510302 | AC109583.1 | 0.009234 | 0.990766 |
| ENST00000510302 | CAMK2N2 | 8.39E-04 | 0.999161 |
| HSALNT0289196 | TAS2R42 | 0.003667 | 0.996333 |
| HSALNT0289196 | GRINA | 0.005042 | 0.994958 |
| HSALNT0289196 | PSRC1 | 0.006118 | -0.99388 |
| HSALNT0289196 | ABCB8 | 0.001428 | 0.998572 |
| HSALNT0289196 | LILRB1 | 0.00431 | -0.99569 |
| HSALNT0289196 | RASL10A | 0.002422 | -0.99758 |
| HSALNT0289196 | LTA4H | 0.001761 | -0.99824 |
| HSALNT0289196 | HERPUD2 | 0.005771 | -0.99423 |
| HSALNT0289196 | TUT4 | 0.00147 | -0.99853 |
| HSALNT0289196 | NFX1 | 0.003139 | -0.99686 |
| HSALNT0289196 | PEA15 | 0.003355 | 0.996645 |
| HSALNT0289196 | ZNF579 | 0.009183 | 0.990817 |
| HSALNT0289196 | SLC12A3 | 0.008937 | -0.99106 |
| HSALNT0289196 | WNT8B | 0.004802 | 0.995198 |
| HSALNT0289196 | PTPN23 | 0.008665 | 0.991335 |
| HSALNT0289196 | UNC5C | 0.003986 | -0.99601 |
| HSALNT0289196 | AGO2 | 0.003917 | 0.996083 |
| HSALNT0289196 | TRIM47 | 0.002753 | 0.997247 |
| HSALNT0289196 | CATG00000087047.1 | 8.09E-04 | 0.999191 |
| HSALNT0289196 | AC109583.1 | 0.007066 | 0.992934 |
| HSALNT0289196 | HPS1 | 0.006829 | 0.993171 |
| HSALNT0289196 | RAD51AP2 | 0.004823 | -0.99518 |
| HSALNT0289196 | TAS2R42 | 0.003667 | 0.996333 |
| HSALNT0289196 | GRINA | 0.005042 | 0.994958 |
| HSALNT0289196 | PSRC1 | 0.006118 | -0.99388 |
| HSALNT0289196 | ABCB8 | 0.001428 | 0.998572 |
| HSALNT0289196 | LILRB1 | 0.00431 | -0.99569 |
| HSALNT0289196 | RASL10A | 0.002422 | -0.99758 |
| HSALNT0289196 | LTA4H | 0.001761 | -0.99824 |
| HSALNT0289196 | HERPUD2 | 0.005771 | -0.99423 |
| HSALNT0289196 | TUT4 | 0.00147 | -0.99853 |
| HSALNT0289196 | NFX1 | 0.003139 | -0.99686 |
| HSALNT0289196 | PEA15 | 0.003355 | 0.996645 |
| HSALNT0289196 | ZNF579 | 0.009183 | 0.990817 |
| HSALNT0289196 | SLC12A3 | 0.008937 | -0.99106 |
| HSALNT0289196 | WNT8B | 0.004802 | 0.995198 |
| HSALNT0289196 | PTPN23 | 0.008665 | 0.991335 |
| HSALNT0289196 | UNC5C | 0.003986 | -0.99601 |
| HSALNT0289196 | AGO2 | 0.003917 | 0.996083 |
| HSALNT0289196 | TRIM47 | 0.002753 | 0.997247 |
| HSALNT0289196 | CATG00000087047.1 | 8.09E-04 | 0.999191 |
| HSALNT0289196 | AC109583.1 | 0.007066 | 0.992934 |
| HSALNT0289196 | HPS1 | 0.006829 | 0.993171 |
| HSALNT0289196 | RAD51AP2 | 0.004823 | -0.99518 |
| HSALNT0289196 | TAS2R42 | 0.003667 | 0.996333 |
| HSALNT0289196 | GRINA | 0.005042 | 0.994958 |
| HSALNT0289196 | PSRC1 | 0.006118 | -0.99388 |
| HSALNT0289196 | ABCB8 | 0.001428 | 0.998572 |
| HSALNT0289196 | LILRB1 | 0.00431 | -0.99569 |
| HSALNT0289196 | RASL10A | 0.002422 | -0.99758 |
| HSALNT0289196 | LTA4H | 0.001761 | -0.99824 |
| HSALNT0289196 | HERPUD2 | 0.005771 | -0.99423 |
| HSALNT0289196 | TUT4 | 0.00147 | -0.99853 |
| HSALNT0289196 | NFX1 | 0.003139 | -0.99686 |
| HSALNT0289196 | PEA15 | 0.003355 | 0.996645 |
| HSALNT0289196 | ZNF579 | 0.009183 | 0.990817 |
| HSALNT0289196 | SLC12A3 | 0.008937 | -0.99106 |
| HSALNT0289196 | WNT8B | 0.004802 | 0.995198 |
| HSALNT0289196 | PTPN23 | 0.008665 | 0.991335 |
| HSALNT0289196 | UNC5C | 0.003986 | -0.99601 |
| HSALNT0289196 | AGO2 | 0.003917 | 0.996083 |
| HSALNT0289196 | TRIM47 | 0.002753 | 0.997247 |
| HSALNT0289196 | CATG00000087047.1 | 8.09E-04 | 0.999191 |
| HSALNT0289196 | AC109583.1 | 0.007066 | 0.992934 |
| HSALNT0289196 | HPS1 | 0.006829 | 0.993171 |
| HSALNT0289196 | RAD51AP2 | 0.004823 | -0.99518 |
| ENST00000587049 | GSTM5 | 0.009159 | -0.99084 |
| ENST00000587049 | SUPV3L1 | 0.006511 | 0.993489 |
| ENST00000587049 | UBE2V1 | 0.006172 | 0.993828 |
| ENST00000587049 | FAM186A | 0.002296 | -0.9977 |
| ENST00000587049 | PGAM5 | 0.001485 | -0.99851 |
| ENST00000587049 | GSTM5 | 0.009159 | -0.99084 |
| ENST00000587049 | SUPV3L1 | 0.006511 | 0.993489 |
| ENST00000587049 | UBE2V1 | 0.006172 | 0.993828 |
| ENST00000587049 | FAM186A | 0.002296 | -0.9977 |
| ENST00000587049 | PGAM5 | 0.001485 | -0.99851 |
| ENST00000529766 | IL31RA | 0.004834 | -0.99517 |
| ENST00000529766 | EHD4 | 0.009165 | 0.990835 |
| ENST00000529766 | SHISA6 | 0.00617 | 0.99383 |
| ENST00000529766 | SMAP2 | 0.003131 | 0.996869 |
| ENST00000529766 | ISG15 | 0.00166 | -0.99834 |
| ENST00000529766 | IL31RA | 0.004834 | -0.99517 |
| ENST00000529766 | EHD4 | 0.009165 | 0.990835 |
| ENST00000529766 | SHISA6 | 0.00617 | 0.99383 |
| ENST00000529766 | SMAP2 | 0.003131 | 0.996869 |
| ENST00000529766 | ISG15 | 0.00166 | -0.99834 |
| ENST00000529766 | IL31RA | 0.004834 | -0.99517 |
| ENST00000529766 | EHD4 | 0.009165 | 0.990835 |
| ENST00000529766 | SHISA6 | 0.00617 | 0.99383 |
| ENST00000529766 | SMAP2 | 0.003131 | 0.996869 |
| ENST00000529766 | ISG15 | 0.00166 | -0.99834 |
| ENST00000529766 | IL31RA | 0.004834 | -0.99517 |
| ENST00000529766 | EHD4 | 0.009165 | 0.990835 |
| ENST00000529766 | SHISA6 | 0.00617 | 0.99383 |
| ENST00000529766 | SMAP2 | 0.003131 | 0.996869 |
| ENST00000529766 | ISG15 | 0.00166 | -0.99834 |
| ENST00000529766 | IL31RA | 0.004834 | -0.99517 |
| ENST00000529766 | EHD4 | 0.009165 | 0.990835 |
| ENST00000529766 | SHISA6 | 0.00617 | 0.99383 |
| ENST00000529766 | SMAP2 | 0.003131 | 0.996869 |
| ENST00000529766 | ISG15 | 0.00166 | -0.99834 |
| ENST00000529766 | IL31RA | 0.004834 | -0.99517 |
| ENST00000529766 | EHD4 | 0.009165 | 0.990835 |
| ENST00000529766 | SHISA6 | 0.00617 | 0.99383 |
| ENST00000529766 | SMAP2 | 0.003131 | 0.996869 |
| ENST00000529766 | ISG15 | 0.00166 | -0.99834 |
| ENST00000529766 | IL31RA | 0.004834 | -0.99517 |
| ENST00000529766 | EHD4 | 0.009165 | 0.990835 |
| ENST00000529766 | SHISA6 | 0.00617 | 0.99383 |
| ENST00000529766 | SMAP2 | 0.003131 | 0.996869 |
| ENST00000529766 | ISG15 | 0.00166 | -0.99834 |
| ENST00000529766 | IL31RA | 0.004834 | -0.99517 |
| ENST00000529766 | EHD4 | 0.009165 | 0.990835 |
| ENST00000529766 | SHISA6 | 0.00617 | 0.99383 |
| ENST00000529766 | SMAP2 | 0.003131 | 0.996869 |
| ENST00000529766 | ISG15 | 0.00166 | -0.99834 |
| ENST00000529766 | IL31RA | 0.004834 | -0.99517 |
| ENST00000529766 | EHD4 | 0.009165 | 0.990835 |
| ENST00000529766 | SHISA6 | 0.00617 | 0.99383 |
| ENST00000529766 | SMAP2 | 0.003131 | 0.996869 |
| ENST00000529766 | ISG15 | 0.00166 | -0.99834 |
| ENST00000529766 | IL31RA | 0.004834 | -0.99517 |
| ENST00000529766 | EHD4 | 0.009165 | 0.990835 |
| ENST00000529766 | SHISA6 | 0.00617 | 0.99383 |
| ENST00000529766 | SMAP2 | 0.003131 | 0.996869 |
| ENST00000529766 | ISG15 | 0.00166 | -0.99834 |
| ENST00000433133 | INTS3 | 9.44E-04 | -0.99906 |
| ENST00000433133 | VPS41 | 0.007124 | -0.99288 |
| ENST00000433133 | SPEF1 | 0.009748 | -0.99025 |
| ENST00000433133 | TCF4 | 0.005392 | -0.99461 |
| ENST00000433133 | TMEM43 | 0.007778 | 0.992222 |
| ENCT00000403049 | HIGD2A | 0.005782 | 0.994218 |
| ENCT00000403049 | SYNDIG1L | 0.002551 | 0.997449 |
| ENCT00000403049 | CATG00000101330.1 | 0.003248 | 0.996752 |
| ENCT00000403049 | IL17RA | 0.009561 | -0.99044 |
| ENCT00000403049 | KRTAP10-6 | 0.005883 | -0.99412 |
| ENCT00000403049 | AP002990.1 | 0.006083 | 0.993917 |
| ENCT00000403049 | AC092073.1 | 0.009292 | -0.99071 |
| ENCT00000403049 | TGFBR3 | 0.007756 | -0.99224 |
| ENCT00000403049 | ARHGEF26 | 0.006595 | -0.9934 |
| ENCT00000403049 | PHKA1 | 0.005617 | -0.99438 |
| ENCT00000403049 | LYPD1 | 0.009042 | -0.99096 |
| ENCT00000403049 | COA1 | 0.005057 | 0.994943 |
| ENCT00000403049 | ELSPBP1 | 0.002787 | -0.99721 |
| ENCT00000403049 | SLC6A5 | 0.002059 | -0.99794 |
| ENCT00000403049 | RRBP1 | 0.009679 | 0.990321 |
| ENCT00000403049 | PSME1 | 0.004595 | 0.995405 |
| ENCT00000403049 | DOHH | 0.006465 | -0.99353 |
| ENCT00000403049 | VPS13A | 0.002217 | -0.99778 |
| ENST00000511693 | MRPL24 | 0.005785 | 0.994215 |
| ENST00000511693 | DCLRE1B | 0.006459 | 0.993541 |
| ENST00000511693 | CLEC4D | 0.006324 | -0.99368 |
| ENST00000511693 | CLRN2 | 0.005501 | -0.9945 |
| ENST00000511693 | SLC11A1 | 7.86E-06 | -0.99999 |
| ENST00000531882 | MRPL24 | 0.002529 | 0.997471 |
| ENST00000531882 | DCLRE1B | 0.003649 | 0.996351 |
| ENST00000531882 | CLRN2 | 0.009096 | -0.9909 |
| ENST00000531882 | SLC11A1 | 5.96E-04 | -0.9994 |
| ENST00000414886 | MIEN1 | 0.00788 | -0.99212 |
| ENST00000414886 | UBE2V1 | 0.00871 | 0.99129 |
| ENST00000414886 | CEP44 | 0.008031 | 0.991969 |
| ENST00000414886 | OR5M8 | 0.002939 | 0.997061 |
| ENST00000414886 | KLRG2 | 0.009157 | 0.990843 |
| T140007 | EDC3 | 0.005704 | 0.994296 |
| T140007 | MYL3 | 0.008449 | 0.991551 |
| T140007 | PCP4 | 0.004523 | 0.995477 |
| T140007 | DHODH | 0.007154 | -0.99285 |
| T140007 | PRPF8 | 0.00436 | -0.99564 |
| T140007 | NPDC1 | 0.002326 | 0.997674 |
| T140007 | CCDC149 | 0.001692 | 0.998308 |
| T140007 | OSBPL5 | 0.005268 | 0.994732 |
| T140007 | CHCHD6 | 0.008248 | 0.991752 |
| T140007 | TMEM51 | 0.002884 | 0.997116 |
| T140007 | CEP290 | 0.0067 | 0.9933 |
| T140007 | PCNX3 | 0.001908 | -0.99809 |
| T140007 | TIGD3 | 0.004754 | -0.99525 |
| T140007 | ZNF107 | 0.001564 | -0.99844 |
| T140007 | BICRA | 0.005444 | 0.994556 |
| T140007 | CEP63 | 0.004646 | -0.99535 |
| T140007 | ACMSD | 0.008235 | -0.99177 |
| T140007 | EDC3 | 0.005704 | 0.994296 |
| T140007 | MYL3 | 0.008449 | 0.991551 |
| T140007 | PCP4 | 0.004523 | 0.995477 |
| T140007 | DHODH | 0.007154 | -0.99285 |
| T140007 | PRPF8 | 0.00436 | -0.99564 |
| T140007 | NPDC1 | 0.002326 | 0.997674 |
| T140007 | CCDC149 | 0.001692 | 0.998308 |
| T140007 | OSBPL5 | 0.005268 | 0.994732 |
| T140007 | CHCHD6 | 0.008248 | 0.991752 |
| T140007 | TMEM51 | 0.002884 | 0.997116 |
| T140007 | CEP290 | 0.0067 | 0.9933 |
| T140007 | PCNX3 | 0.001908 | -0.99809 |
| T140007 | TIGD3 | 0.004754 | -0.99525 |
| T140007 | ZNF107 | 0.001564 | -0.99844 |
| T140007 | BICRA | 0.005444 | 0.994556 |
| T140007 | CEP63 | 0.004646 | -0.99535 |
| T140007 | ACMSD | 0.008235 | -0.99177 |
| ENST00000609349 | SYNDIG1L | 0.008402 | 0.991598 |
| ENST00000609349 | IL17RA | 0.007846 | -0.99215 |
| ENST00000609349 | AHSP | 0.003983 | -0.99602 |
| ENST00000609349 | AC092073.1 | 8.19E-04 | -0.99918 |
| ENST00000609349 | TGFBR3 | 6.16E-04 | -0.99938 |
| ENST00000609349 | SLC22A6 | 0.00678 | 0.99322 |
| ENST00000609349 | COA1 | 0.007547 | 0.992453 |
| ENST00000609349 | COA5 | 0.00436 | 0.99564 |
| ENST00000609349 | SLC6A5 | 0.003704 | -0.9963 |
| ENST00000609349 | RRBP1 | 9.29E-04 | 0.999071 |
| ENST00000609349 | MORN3 | 0.001772 | 0.998228 |
| ENST00000609349 | VPS13A | 5.27E-04 | -0.99947 |
| ENST00000609349 | CAPN15 | 0.005237 | -0.99476 |
| ENST00000637411 | FBXL16 | 0.00978 | 0.99022 |
| ENST00000637411 | ZDBF2 | 0.008838 | -0.99116 |
| ENST00000637411 | EMP1 | 0.007346 | 0.992654 |
| ENST00000637411 | IGFL2 | 3.43E-04 | 0.999657 |
| ENST00000637411 | S100P | 8.62E-04 | -0.99914 |
| ENST00000468480 | VGLL3 | 0.009527 | -0.99047 |
| ENST00000468480 | ASB11 | 0.007919 | 0.992081 |
| ENST00000468480 | MMP2 | 0.007597 | -0.9924 |
| ENST00000468480 | FAM186A | 0.001808 | -0.99819 |
| ENST00000468480 | PGAM5 | 0.007715 | -0.99229 |
| ENST00000468480 | VGLL3 | 0.009527 | -0.99047 |
| ENST00000468480 | ASB11 | 0.007919 | 0.992081 |
| ENST00000468480 | MMP2 | 0.007597 | -0.9924 |
| ENST00000468480 | FAM186A | 0.001808 | -0.99819 |
| ENST00000468480 | PGAM5 | 0.007715 | -0.99229 |
| ENST00000468480 | VGLL3 | 0.009527 | -0.99047 |
| ENST00000468480 | ASB11 | 0.007919 | 0.992081 |
| ENST00000468480 | MMP2 | 0.007597 | -0.9924 |
| ENST00000468480 | FAM186A | 0.001808 | -0.99819 |
| ENST00000468480 | PGAM5 | 0.007715 | -0.99229 |
| ENST00000468480 | VGLL3 | 0.009527 | -0.99047 |
| ENST00000468480 | ASB11 | 0.007919 | 0.992081 |
| ENST00000468480 | MMP2 | 0.007597 | -0.9924 |
| ENST00000468480 | FAM186A | 0.001808 | -0.99819 |
| ENST00000468480 | PGAM5 | 0.007715 | -0.99229 |
| T295609 | ZC3H7B | 0.009227 | 0.990773 |
| T295609 | CATG00000061038.1 | 2.68E-04 | -0.99973 |
| T295609 | OR10R2 | 0.0052 | 0.9948 |
| T295609 | DDIT4 | 0.002874 | -0.99713 |
| ENST00000503242 | FBXL16 | 0.008669 | 0.991331 |
| ENST00000503242 | ZDBF2 | 0.002638 | -0.99736 |
| ENST00000503242 | GABRG1 | 0.002555 | -0.99745 |
| ENST00000503242 | FBXL16 | 0.008669 | 0.991331 |
| ENST00000503242 | ZDBF2 | 0.002638 | -0.99736 |
| ENST00000503242 | GABRG1 | 0.002555 | -0.99745 |
| ENST00000503242 | FBXL16 | 0.008669 | 0.991331 |
| ENST00000503242 | ZDBF2 | 0.002638 | -0.99736 |
| ENST00000503242 | GABRG1 | 0.002555 | -0.99745 |
| ENST00000583012 | WDR90 | 0.004255 | 0.995745 |
| ENST00000583012 | SORCS2 | 0.006279 | 0.993721 |
| ENST00000583012 | NUP54 | 0.005298 | 0.994702 |
| ENST00000583012 | KIF17 | 2.36E-05 | 0.999976 |
| ENST00000583012 | TDRD12 | 0.008178 | 0.991822 |
| ENST00000583012 | FTSJ1 | 0.006109 | 0.993891 |
| ENST00000583012 | WDR90 | 0.004255 | 0.995745 |
| ENST00000583012 | SORCS2 | 0.006279 | 0.993721 |
| ENST00000583012 | NUP54 | 0.005298 | 0.994702 |
| ENST00000583012 | KIF17 | 2.36E-05 | 0.999976 |
| ENST00000583012 | TDRD12 | 0.008178 | 0.991822 |
| ENST00000583012 | FTSJ1 | 0.006109 | 0.993891 |
| ENST00000583012 | WDR90 | 0.004255 | 0.995745 |
| ENST00000583012 | SORCS2 | 0.006279 | 0.993721 |
| ENST00000583012 | NUP54 | 0.005298 | 0.994702 |
| ENST00000583012 | KIF17 | 2.36E-05 | 0.999976 |
| ENST00000583012 | TDRD12 | 0.008178 | 0.991822 |
| ENST00000583012 | FTSJ1 | 0.006109 | 0.993891 |
| ENST00000583012 | WDR90 | 0.004255 | 0.995745 |
| ENST00000583012 | SORCS2 | 0.006279 | 0.993721 |
| ENST00000583012 | NUP54 | 0.005298 | 0.994702 |
| ENST00000583012 | KIF17 | 2.36E-05 | 0.999976 |
| ENST00000583012 | TDRD12 | 0.008178 | 0.991822 |
| ENST00000583012 | FTSJ1 | 0.006109 | 0.993891 |
| T315462 | ACOT12 | 0.004217 | -0.99578 |
| T315462 | SELENOP | 0.009441 | 0.990559 |
| T315462 | BCAS4 | 0.003952 | -0.99605 |
| T315462 | PROKR1 | 0.005992 | 0.994008 |
| T315462 | OR8B4 | 0.001394 | 0.998606 |
| T315462 | MYH4 | 0.006649 | -0.99335 |
| T315462 | FANCL | 0.001144 | -0.99886 |
| ENST00000467309 | RFC2 | 0.002733 | -0.99727 |
| ENST00000467309 | CATG00000101330.1 | 0.004705 | -0.99529 |
| ENST00000467309 | CNEP1R1 | 0.001348 | -0.99865 |
| ENST00000467309 | SH3D21 | 0.005918 | 0.994082 |
| ENST00000467309 | TP73 | 0.004058 | 0.995942 |
| ENST00000467309 | ARHGEF26 | 0.003308 | 0.996692 |
| ENST00000467309 | PHKA1 | 0.001369 | 0.998631 |
| ENST00000467309 | CATG00000026557.1 | 0.001358 | 0.998642 |
| ENST00000467309 | COA1 | 0.007204 | -0.9928 |
| ENST00000467309 | PSME1 | 0.006881 | -0.99312 |
| ENST00000467309 | DOCK1 | 7.38E-04 | 0.999262 |
| ENST00000467309 | BMPER | 0.00902 | 0.99098 |
| ENST00000467309 | RFC2 | 0.002733 | -0.99727 |
| ENST00000467309 | CATG00000101330.1 | 0.004705 | -0.99529 |
| ENST00000467309 | CNEP1R1 | 0.001348 | -0.99865 |
| ENST00000467309 | SH3D21 | 0.005918 | 0.994082 |
| ENST00000467309 | TP73 | 0.004058 | 0.995942 |
| ENST00000467309 | ARHGEF26 | 0.003308 | 0.996692 |
| ENST00000467309 | PHKA1 | 0.001369 | 0.998631 |
| ENST00000467309 | CATG00000026557.1 | 0.001358 | 0.998642 |
| ENST00000467309 | COA1 | 0.007204 | -0.9928 |
| ENST00000467309 | PSME1 | 0.006881 | -0.99312 |
| ENST00000467309 | DOCK1 | 7.38E-04 | 0.999262 |
| ENST00000467309 | BMPER | 0.00902 | 0.99098 |
| ENST00000467309 | RFC2 | 0.002733 | -0.99727 |
| ENST00000467309 | CATG00000101330.1 | 0.004705 | -0.99529 |
| ENST00000467309 | CNEP1R1 | 0.001348 | -0.99865 |
| ENST00000467309 | SH3D21 | 0.005918 | 0.994082 |
| ENST00000467309 | TP73 | 0.004058 | 0.995942 |
| ENST00000467309 | ARHGEF26 | 0.003308 | 0.996692 |
| ENST00000467309 | PHKA1 | 0.001369 | 0.998631 |
| ENST00000467309 | CATG00000026557.1 | 0.001358 | 0.998642 |
| ENST00000467309 | COA1 | 0.007204 | -0.9928 |
| ENST00000467309 | PSME1 | 0.006881 | -0.99312 |
| ENST00000467309 | DOCK1 | 7.38E-04 | 0.999262 |
| ENST00000467309 | BMPER | 0.00902 | 0.99098 |
| ENST00000467309 | RFC2 | 0.002733 | -0.99727 |
| ENST00000467309 | CATG00000101330.1 | 0.004705 | -0.99529 |
| ENST00000467309 | CNEP1R1 | 0.001348 | -0.99865 |
| ENST00000467309 | SH3D21 | 0.005918 | 0.994082 |
| ENST00000467309 | TP73 | 0.004058 | 0.995942 |
| ENST00000467309 | ARHGEF26 | 0.003308 | 0.996692 |
| ENST00000467309 | PHKA1 | 0.001369 | 0.998631 |
| ENST00000467309 | CATG00000026557.1 | 0.001358 | 0.998642 |
| ENST00000467309 | COA1 | 0.007204 | -0.9928 |
| ENST00000467309 | PSME1 | 0.006881 | -0.99312 |
| ENST00000467309 | DOCK1 | 7.38E-04 | 0.999262 |
| ENST00000467309 | BMPER | 0.00902 | 0.99098 |
| ENST00000467309 | RFC2 | 0.002733 | -0.99727 |
| ENST00000467309 | CATG00000101330.1 | 0.004705 | -0.99529 |
| ENST00000467309 | CNEP1R1 | 0.001348 | -0.99865 |
| ENST00000467309 | SH3D21 | 0.005918 | 0.994082 |
| ENST00000467309 | TP73 | 0.004058 | 0.995942 |
| ENST00000467309 | ARHGEF26 | 0.003308 | 0.996692 |
| ENST00000467309 | PHKA1 | 0.001369 | 0.998631 |
| ENST00000467309 | CATG00000026557.1 | 0.001358 | 0.998642 |
| ENST00000467309 | COA1 | 0.007204 | -0.9928 |
| ENST00000467309 | PSME1 | 0.006881 | -0.99312 |
| ENST00000467309 | DOCK1 | 7.38E-04 | 0.999262 |
| ENST00000467309 | BMPER | 0.00902 | 0.99098 |
| ENST00000467309 | RFC2 | 0.002733 | -0.99727 |
| ENST00000467309 | CATG00000101330.1 | 0.004705 | -0.99529 |
| ENST00000467309 | CNEP1R1 | 0.001348 | -0.99865 |
| ENST00000467309 | SH3D21 | 0.005918 | 0.994082 |
| ENST00000467309 | TP73 | 0.004058 | 0.995942 |
| ENST00000467309 | ARHGEF26 | 0.003308 | 0.996692 |
| ENST00000467309 | PHKA1 | 0.001369 | 0.998631 |
| ENST00000467309 | CATG00000026557.1 | 0.001358 | 0.998642 |
| ENST00000467309 | COA1 | 0.007204 | -0.9928 |
| ENST00000467309 | PSME1 | 0.006881 | -0.99312 |
| ENST00000467309 | DOCK1 | 7.38E-04 | 0.999262 |
| ENST00000467309 | BMPER | 0.00902 | 0.99098 |
| NR_038202 | BARHL1 | 0.003967 | 0.996033 |
| NR_038202 | B4GALNT1 | 0.009579 | 0.990421 |
| NR_038202 | S1PR5 | 0.004315 | 0.995685 |
| NR_038202 | RPL29 | 0.001885 | -0.99812 |
| NR_038202 | RP1L1 | 0.007032 | 0.992968 |
| NR_038202 | CNOT1 | 0.007583 | -0.99242 |
| NR_038202 | DEFA1 | 5.12E-04 | -0.99949 |
| NR_038202 | PLEKHG3 | 0.008454 | 0.991546 |
| NR_038202 | BARHL1 | 0.003967 | 0.996033 |
| NR_038202 | B4GALNT1 | 0.009579 | 0.990421 |
| NR_038202 | S1PR5 | 0.004315 | 0.995685 |
| NR_038202 | RPL29 | 0.001885 | -0.99812 |
| NR_038202 | RP1L1 | 0.007032 | 0.992968 |
| NR_038202 | CNOT1 | 0.007583 | -0.99242 |
| NR_038202 | DEFA1 | 5.12E-04 | -0.99949 |
| NR_038202 | PLEKHG3 | 0.008454 | 0.991546 |
| ENST00000529719 | B4GALNT1 | 0.006718 | 0.993282 |
| ENST00000529719 | ZFY | 0.001074 | 0.998926 |
| ENST00000529719 | S1PR5 | 0.009182 | 0.990818 |
| ENST00000529719 | PAK5 | 0.009531 | 0.990469 |
| ENST00000529719 | RAB8A | 0.008336 | 0.991664 |
| ENST00000529719 | SSX1 | 0.001033 | -0.99897 |
| ENST00000529719 | WSCD2 | 0.00516 | -0.99484 |
| ENST00000529719 | GRIN3B | 0.001714 | 0.998286 |
| ENST00000529719 | PFKFB4 | 0.009837 | 0.990163 |
| ENST00000529719 | CATG00000068089.1 | 0.009282 | -0.99072 |
| ENST00000529719 | PTPRK | 0.00767 | -0.99233 |
| ENST00000529719 | DEFA4 | 9.59E-04 | -0.99904 |
| ENST00000600704 | ZNF227 | 0.00122 | 0.99878 |
| ENST00000600704 | DKK4 | 0.008333 | 0.991667 |
| ENST00000600704 | NAT8 | 0.003528 | 0.996472 |
| ENST00000600704 | TLR2 | 0.006215 | 0.993785 |
| ENST00000600704 | PLA2G4F | 8.00E-04 | 0.9992 |
| ENST00000600704 | ZNF227 | 0.00122 | 0.99878 |
| ENST00000600704 | DKK4 | 0.008333 | 0.991667 |
| ENST00000600704 | NAT8 | 0.003528 | 0.996472 |
| ENST00000600704 | TLR2 | 0.006215 | 0.993785 |
| ENST00000600704 | PLA2G4F | 8.00E-04 | 0.9992 |
| ENST00000600704 | ZNF227 | 0.00122 | 0.99878 |
| ENST00000600704 | DKK4 | 0.008333 | 0.991667 |
| ENST00000600704 | NAT8 | 0.003528 | 0.996472 |
| ENST00000600704 | TLR2 | 0.006215 | 0.993785 |
| ENST00000600704 | PLA2G4F | 8.00E-04 | 0.9992 |
| ENST00000600704 | ZNF227 | 0.00122 | 0.99878 |
| ENST00000600704 | DKK4 | 0.008333 | 0.991667 |
| ENST00000600704 | NAT8 | 0.003528 | 0.996472 |
| ENST00000600704 | TLR2 | 0.006215 | 0.993785 |
| ENST00000600704 | PLA2G4F | 8.00E-04 | 0.9992 |
| ENST00000600704 | ZNF227 | 0.00122 | 0.99878 |
| ENST00000600704 | DKK4 | 0.008333 | 0.991667 |
| ENST00000600704 | NAT8 | 0.003528 | 0.996472 |
| ENST00000600704 | TLR2 | 0.006215 | 0.993785 |
| ENST00000600704 | PLA2G4F | 8.00E-04 | 0.9992 |
| ENST00000600704 | ZNF227 | 0.00122 | 0.99878 |
| ENST00000600704 | DKK4 | 0.008333 | 0.991667 |
| ENST00000600704 | NAT8 | 0.003528 | 0.996472 |
| ENST00000600704 | TLR2 | 0.006215 | 0.993785 |
| ENST00000600704 | PLA2G4F | 8.00E-04 | 0.9992 |
| ENST00000600704 | ZNF227 | 0.00122 | 0.99878 |
| ENST00000600704 | DKK4 | 0.008333 | 0.991667 |
| ENST00000600704 | NAT8 | 0.003528 | 0.996472 |
| ENST00000600704 | TLR2 | 0.006215 | 0.993785 |
| ENST00000600704 | PLA2G4F | 8.00E-04 | 0.9992 |
| ENST00000600704 | ZNF227 | 0.00122 | 0.99878 |
| ENST00000600704 | DKK4 | 0.008333 | 0.991667 |
| ENST00000600704 | NAT8 | 0.003528 | 0.996472 |
| ENST00000600704 | TLR2 | 0.006215 | 0.993785 |
| ENST00000600704 | PLA2G4F | 8.00E-04 | 0.9992 |
| ENST00000600704 | ZNF227 | 0.00122 | 0.99878 |
| ENST00000600704 | DKK4 | 0.008333 | 0.991667 |
| ENST00000600704 | NAT8 | 0.003528 | 0.996472 |
| ENST00000600704 | TLR2 | 0.006215 | 0.993785 |
| ENST00000600704 | PLA2G4F | 8.00E-04 | 0.9992 |
| ENST00000484368 | TRMT11 | 0.002551 | 0.997449 |
| ENST00000484368 | ADGRE1 | 0.00707 | 0.99293 |
| ENST00000484368 | MYLK3 | 0.001558 | 0.998442 |
| ENST00000484368 | SIRT6 | 0.006925 | 0.993075 |
| ENST00000484368 | SLC39A12 | 0.002705 | -0.99729 |
| ENST00000484368 | TRMT11 | 0.002551 | 0.997449 |
| ENST00000484368 | ADGRE1 | 0.00707 | 0.99293 |
| ENST00000484368 | MYLK3 | 0.001558 | 0.998442 |
| ENST00000484368 | SIRT6 | 0.006925 | 0.993075 |
| ENST00000484368 | SLC39A12 | 0.002705 | -0.99729 |
| ENST00000484368 | TRMT11 | 0.002551 | 0.997449 |
| ENST00000484368 | ADGRE1 | 0.00707 | 0.99293 |
| ENST00000484368 | MYLK3 | 0.001558 | 0.998442 |
| ENST00000484368 | SIRT6 | 0.006925 | 0.993075 |
| ENST00000484368 | SLC39A12 | 0.002705 | -0.99729 |
| ENST00000484368 | TRMT11 | 0.002551 | 0.997449 |
| ENST00000484368 | ADGRE1 | 0.00707 | 0.99293 |
| ENST00000484368 | MYLK3 | 0.001558 | 0.998442 |
| ENST00000484368 | SIRT6 | 0.006925 | 0.993075 |
| ENST00000484368 | SLC39A12 | 0.002705 | -0.99729 |
| ENST00000484368 | TRMT11 | 0.002551 | 0.997449 |
| ENST00000484368 | ADGRE1 | 0.00707 | 0.99293 |
| ENST00000484368 | MYLK3 | 0.001558 | 0.998442 |
| ENST00000484368 | SIRT6 | 0.006925 | 0.993075 |
| ENST00000484368 | SLC39A12 | 0.002705 | -0.99729 |
| ENST00000484368 | TRMT11 | 0.002551 | 0.997449 |
| ENST00000484368 | ADGRE1 | 0.00707 | 0.99293 |
| ENST00000484368 | MYLK3 | 0.001558 | 0.998442 |
| ENST00000484368 | SIRT6 | 0.006925 | 0.993075 |
| ENST00000484368 | SLC39A12 | 0.002705 | -0.99729 |
| ENST00000484368 | TRMT11 | 0.002551 | 0.997449 |
| ENST00000484368 | ADGRE1 | 0.00707 | 0.99293 |
| ENST00000484368 | MYLK3 | 0.001558 | 0.998442 |
| ENST00000484368 | SIRT6 | 0.006925 | 0.993075 |
| ENST00000484368 | SLC39A12 | 0.002705 | -0.99729 |
| ENST00000484368 | TRMT11 | 0.002551 | 0.997449 |
| ENST00000484368 | ADGRE1 | 0.00707 | 0.99293 |
| ENST00000484368 | MYLK3 | 0.001558 | 0.998442 |
| ENST00000484368 | SIRT6 | 0.006925 | 0.993075 |
| ENST00000484368 | SLC39A12 | 0.002705 | -0.99729 |
| ENST00000484368 | TRMT11 | 0.002551 | 0.997449 |
| ENST00000484368 | ADGRE1 | 0.00707 | 0.99293 |
| ENST00000484368 | MYLK3 | 0.001558 | 0.998442 |
| ENST00000484368 | SIRT6 | 0.006925 | 0.993075 |
| ENST00000484368 | SLC39A12 | 0.002705 | -0.99729 |
| ENST00000484368 | TRMT11 | 0.002551 | 0.997449 |
| ENST00000484368 | ADGRE1 | 0.00707 | 0.99293 |
| ENST00000484368 | MYLK3 | 0.001558 | 0.998442 |
| ENST00000484368 | SIRT6 | 0.006925 | 0.993075 |
| ENST00000484368 | SLC39A12 | 0.002705 | -0.99729 |
| NR_136320 | CMYA5 | 0.002005 | 0.997995 |
| NR_136320 | CCDC172 | 0.003571 | 0.996429 |
| NR_136320 | CNOT1 | 0.009572 | -0.99043 |
| NR_136320 | RCSD1 | 0.007784 | -0.99222 |
| NR_136320 | MOK | 0.004868 | -0.99513 |
| ENST00000432621 | MYH7 | 0.00921 | -0.99079 |
| ENST00000432621 | NABP2 | 0.001438 | 0.998562 |
| ENST00000432621 | PPP1R16B | 0.009458 | 0.990542 |
| ENST00000432621 | FOXR1 | 0.001944 | -0.99806 |
| ENST00000432621 | MYH7 | 0.00921 | -0.99079 |
| ENST00000432621 | NABP2 | 0.001438 | 0.998562 |
| ENST00000432621 | PPP1R16B | 0.009458 | 0.990542 |
| ENST00000432621 | FOXR1 | 0.001944 | -0.99806 |
| ENST00000432621 | MYH7 | 0.00921 | -0.99079 |
| ENST00000432621 | NABP2 | 0.001438 | 0.998562 |
| ENST00000432621 | PPP1R16B | 0.009458 | 0.990542 |
| ENST00000432621 | FOXR1 | 0.001944 | -0.99806 |
| ENST00000432621 | MYH7 | 0.00921 | -0.99079 |
| ENST00000432621 | NABP2 | 0.001438 | 0.998562 |
| ENST00000432621 | PPP1R16B | 0.009458 | 0.990542 |
| ENST00000432621 | FOXR1 | 0.001944 | -0.99806 |
| ENST00000432621 | MYH7 | 0.00921 | -0.99079 |
| ENST00000432621 | NABP2 | 0.001438 | 0.998562 |
| ENST00000432621 | PPP1R16B | 0.009458 | 0.990542 |
| ENST00000432621 | FOXR1 | 0.001944 | -0.99806 |
| ENST00000432621 | MYH7 | 0.00921 | -0.99079 |
| ENST00000432621 | NABP2 | 0.001438 | 0.998562 |
| ENST00000432621 | PPP1R16B | 0.009458 | 0.990542 |
| ENST00000432621 | FOXR1 | 0.001944 | -0.99806 |
| ENST00000432621 | MYH7 | 0.00921 | -0.99079 |
| ENST00000432621 | NABP2 | 0.001438 | 0.998562 |
| ENST00000432621 | PPP1R16B | 0.009458 | 0.990542 |
| ENST00000432621 | FOXR1 | 0.001944 | -0.99806 |
| ENST00000650103 | TAS2R42 | 0.003298 | -0.9967 |
| ENST00000650103 | PPP3CC | 0.003633 | 0.996367 |
| ENST00000650103 | PSRC1 | 0.002662 | 0.997338 |
| ENST00000650103 | ABCB8 | 0.007316 | -0.99268 |
| ENST00000650103 | RASL10A | 0.007846 | 0.992154 |
| ENST00000650103 | LTA4H | 3.37E-04 | 0.999663 |
| ENST00000650103 | TUT4 | 0.002605 | 0.997395 |
| ENST00000650103 | WDR33 | 0.002112 | -0.99789 |
| ENST00000650103 | CATG00000053512.1 | 0.005275 | 0.994725 |
| ENST00000650103 | HLF | 0.008364 | -0.99164 |
| ENST00000650103 | NFX1 | 0.007627 | 0.992373 |
| ENST00000650103 | CATG00000063823.1 | 0.007388 | -0.99261 |
| ENST00000650103 | ZNF607 | 0.009513 | -0.99049 |
| ENST00000650103 | PEA15 | 0.007961 | -0.99204 |
| ENST00000650103 | PSMD4 | 0.006621 | 0.993379 |
| ENST00000650103 | SLC12A3 | 0.004884 | 0.995116 |
| ENST00000650103 | WNT8B | 0.007761 | -0.99224 |
| ENST00000650103 | OR6F1 | 0.006969 | -0.99303 |
| ENST00000650103 | ADAM28 | 0.006668 | 0.993332 |
| ENST00000650103 | UNC5C | 0.006879 | 0.993121 |
| ENST00000650103 | TRIM47 | 0.009348 | -0.99065 |
| ENST00000650103 | CATG00000087047.1 | 0.001019 | -0.99898 |
| ENST00000650103 | AC109583.1 | 0.008219 | -0.99178 |
| ENST00000650103 | CFAP410 | 0.006745 | -0.99325 |
| ENST00000650103 | HPS1 | 0.009809 | -0.99019 |
| ENST00000650103 | RAD51AP2 | 4.56E-04 | 0.999544 |
| ENST00000650103 | TAS2R42 | 0.003298 | -0.9967 |
| ENST00000650103 | PPP3CC | 0.003633 | 0.996367 |
| ENST00000650103 | PSRC1 | 0.002662 | 0.997338 |
| ENST00000650103 | ABCB8 | 0.007316 | -0.99268 |
| ENST00000650103 | RASL10A | 0.007846 | 0.992154 |
| ENST00000650103 | LTA4H | 3.37E-04 | 0.999663 |
| ENST00000650103 | TUT4 | 0.002605 | 0.997395 |
| ENST00000650103 | WDR33 | 0.002112 | -0.99789 |
| ENST00000650103 | CATG00000053512.1 | 0.005275 | 0.994725 |
| ENST00000650103 | HLF | 0.008364 | -0.99164 |
| ENST00000650103 | NFX1 | 0.007627 | 0.992373 |
| ENST00000650103 | CATG00000063823.1 | 0.007388 | -0.99261 |
| ENST00000650103 | ZNF607 | 0.009513 | -0.99049 |
| ENST00000650103 | PEA15 | 0.007961 | -0.99204 |
| ENST00000650103 | PSMD4 | 0.006621 | 0.993379 |
| ENST00000650103 | SLC12A3 | 0.004884 | 0.995116 |
| ENST00000650103 | WNT8B | 0.007761 | -0.99224 |
| ENST00000650103 | OR6F1 | 0.006969 | -0.99303 |
| ENST00000650103 | ADAM28 | 0.006668 | 0.993332 |
| ENST00000650103 | UNC5C | 0.006879 | 0.993121 |
| ENST00000650103 | TRIM47 | 0.009348 | -0.99065 |
| ENST00000650103 | CATG00000087047.1 | 0.001019 | -0.99898 |
| ENST00000650103 | AC109583.1 | 0.008219 | -0.99178 |
| ENST00000650103 | CFAP410 | 0.006745 | -0.99325 |
| ENST00000650103 | HPS1 | 0.009809 | -0.99019 |
| ENST00000650103 | RAD51AP2 | 4.56E-04 | 0.999544 |
| ENST00000650103 | TAS2R42 | 0.003298 | -0.9967 |
| ENST00000650103 | PPP3CC | 0.003633 | 0.996367 |
| ENST00000650103 | PSRC1 | 0.002662 | 0.997338 |
| ENST00000650103 | ABCB8 | 0.007316 | -0.99268 |
| ENST00000650103 | RASL10A | 0.007846 | 0.992154 |
| ENST00000650103 | LTA4H | 3.37E-04 | 0.999663 |
| ENST00000650103 | TUT4 | 0.002605 | 0.997395 |
| ENST00000650103 | WDR33 | 0.002112 | -0.99789 |
| ENST00000650103 | CATG00000053512.1 | 0.005275 | 0.994725 |
| ENST00000650103 | HLF | 0.008364 | -0.99164 |
| ENST00000650103 | NFX1 | 0.007627 | 0.992373 |
| ENST00000650103 | CATG00000063823.1 | 0.007388 | -0.99261 |
| ENST00000650103 | ZNF607 | 0.009513 | -0.99049 |
| ENST00000650103 | PEA15 | 0.007961 | -0.99204 |
| ENST00000650103 | PSMD4 | 0.006621 | 0.993379 |
| ENST00000650103 | SLC12A3 | 0.004884 | 0.995116 |
| ENST00000650103 | WNT8B | 0.007761 | -0.99224 |
| ENST00000650103 | OR6F1 | 0.006969 | -0.99303 |
| ENST00000650103 | ADAM28 | 0.006668 | 0.993332 |
| ENST00000650103 | UNC5C | 0.006879 | 0.993121 |
| ENST00000650103 | TRIM47 | 0.009348 | -0.99065 |
| ENST00000650103 | CATG00000087047.1 | 0.001019 | -0.99898 |
| ENST00000650103 | AC109583.1 | 0.008219 | -0.99178 |
| ENST00000650103 | CFAP410 | 0.006745 | -0.99325 |
| ENST00000650103 | HPS1 | 0.009809 | -0.99019 |
| ENST00000650103 | RAD51AP2 | 4.56E-04 | 0.999544 |
| ENST00000601205 | PTPN6 | 0.003009 | 0.996991 |
| ENST00000601205 | FILIP1 | 0.004046 | 0.995954 |
| ENST00000601205 | NOL9 | 0.001222 | 0.998778 |
| ENST00000601205 | PMPCA | 0.008785 | -0.99121 |
| ENST00000601205 | IRX3 | 0.005354 | -0.99465 |
| ENST00000601205 | CD180 | 0.002947 | 0.997053 |
| ENST00000601205 | CAPNS1 | 0.005195 | 0.994805 |
| ENST00000601205 | SPATA13 | 0.002977 | -0.99702 |
| ENST00000601205 | COBL | 0.007393 | -0.99261 |
| ENST00000601205 | KRTAP4-7 | 0.004685 | -0.99531 |
| ENST00000601205 | SPSB2 | 0.009691 | -0.99031 |
| ENST00000601205 | SIPA1L2 | 0.002176 | -0.99782 |
| ENST00000626649 | BECN1 | 0.007452 | 0.992548 |
| ENST00000626649 | ZNF736 | 0.00282 | 0.99718 |
| ENST00000626649 | CCDC42 | 0.004141 | 0.995859 |
| ENST00000626649 | BECN1 | 0.007452 | 0.992548 |
| ENST00000626649 | ZNF736 | 0.00282 | 0.99718 |
| ENST00000626649 | CCDC42 | 0.004141 | 0.995859 |
| ENST00000626649 | BECN1 | 0.007452 | 0.992548 |
| ENST00000626649 | ZNF736 | 0.00282 | 0.99718 |
| ENST00000626649 | CCDC42 | 0.004141 | 0.995859 |
| ENST00000626649 | BECN1 | 0.007452 | 0.992548 |
| ENST00000626649 | ZNF736 | 0.00282 | 0.99718 |
| ENST00000626649 | CCDC42 | 0.004141 | 0.995859 |
| ENST00000626649 | BECN1 | 0.007452 | 0.992548 |
| ENST00000626649 | ZNF736 | 0.00282 | 0.99718 |
| ENST00000626649 | CCDC42 | 0.004141 | 0.995859 |
| ENST00000626649 | BECN1 | 0.007452 | 0.992548 |
| ENST00000626649 | ZNF736 | 0.00282 | 0.99718 |
| ENST00000626649 | CCDC42 | 0.004141 | 0.995859 |
| ENST00000626649 | BECN1 | 0.007452 | 0.992548 |
| ENST00000626649 | ZNF736 | 0.00282 | 0.99718 |
| ENST00000626649 | CCDC42 | 0.004141 | 0.995859 |
| ENST00000535324 | ECSIT | 1.46E-04 | -0.99985 |
| ENST00000535324 | CATG00000056264.1 | 0.005499 | -0.9945 |
| ENST00000535324 | SIRPG | 0.002942 | -0.99706 |
| ENST00000535324 | SSBP2 | 0.001527 | -0.99847 |
| ENST00000535324 | CHST8 | 0.001396 | -0.9986 |
| ENST00000535324 | TMC2 | 0.005482 | -0.99452 |
| ENST00000535324 | FRG1 | 0.005685 | 0.994315 |
| ENST00000535324 | CATG00000022188.1 | 0.006149 | -0.99385 |
| ENST00000535324 | CLK3 | 0.008859 | -0.99114 |
| ENST00000535324 | TGFBR3 | 0.009204 | -0.9908 |
| ENST00000535324 | SLC22A6 | 0.001345 | 0.998655 |
| ENST00000535324 | SPATA31D1 | 0.007502 | -0.9925 |
| ENST00000535324 | CATG00000038058.1 | 0.004803 | -0.9952 |
| ENST00000535324 | COA5 | 0.002868 | 0.997132 |
| ENST00000535324 | ZC3H12D | 0.009105 | 0.990895 |
| ENST00000535324 | MORN3 | 0.00766 | 0.99234 |
| ENST00000535324 | FGF9 | 0.004858 | 0.995142 |
| ENST00000535324 | CAPN15 | 0.008247 | -0.99175 |
| ENST00000606282 | LGALS1 | 0.004444 | 0.995556 |
| ENST00000606282 | DUSP2 | 0.006585 | 0.993415 |
| ENST00000609706 | WDR90 | 0.005719 | -0.99428 |
| ENST00000609706 | SORCS2 | 0.0059 | -0.9941 |
| ENST00000609706 | KIF17 | 0.002728 | -0.99727 |
| ENST00000609706 | MDK | 0.006837 | -0.99316 |
| ENST00000609706 | FTSJ1 | 0.009109 | -0.99089 |
| ENST00000609706 | CLCN5 | 0.00423 | -0.99577 |
| ENST00000609706 | WDR90 | 0.005719 | -0.99428 |
| ENST00000609706 | SORCS2 | 0.0059 | -0.9941 |
| ENST00000609706 | KIF17 | 0.002728 | -0.99727 |
| ENST00000609706 | MDK | 0.006837 | -0.99316 |
| ENST00000609706 | FTSJ1 | 0.009109 | -0.99089 |
| ENST00000609706 | CLCN5 | 0.00423 | -0.99577 |
| ENST00000609706 | WDR90 | 0.005719 | -0.99428 |
| ENST00000609706 | SORCS2 | 0.0059 | -0.9941 |
| ENST00000609706 | KIF17 | 0.002728 | -0.99727 |
| ENST00000609706 | MDK | 0.006837 | -0.99316 |
| ENST00000609706 | FTSJ1 | 0.009109 | -0.99089 |
| ENST00000609706 | CLCN5 | 0.00423 | -0.99577 |
| NR_110863 | HIGD2A | 0.008538 | -0.99146 |
| NR_110863 | RSL1D1 | 0.006242 | -0.99376 |
| NR_110863 | SLC2A4 | 0.0037 | 0.9963 |
| NR_110863 | SYNDIG1L | 0.001615 | -0.99839 |
| NR_110863 | HIRA | 0.003589 | -0.99641 |
| NR_110863 | TMEM155 | 0.006107 | 0.993893 |
| NR_110863 | AQP1 | 0.00609 | -0.99391 |
| NR_110863 | IL17RA | 9.59E-04 | 0.999041 |
| NR_110863 | KRTAP10-6 | 0.004569 | 0.995431 |
| NR_110863 | PLPPR4 | 0.005004 | -0.995 |
| NR_110863 | HLA-A | 0.004968 | -0.99503 |
| NR_110863 | AP002990.1 | 0.0049 | -0.9951 |
| NR_110863 | KCNK10 | 0.004556 | 0.995444 |
| NR_110863 | LYPD1 | 2.04E-04 | 0.999796 |
| NR_110863 | ELSPBP1 | 0.003747 | 0.996253 |
| NR_110863 | SLC6A5 | 0.001788 | 0.998212 |
| NR_110863 | VPS13A | 0.008794 | 0.991206 |
| ENST00000528650 | IL17RA | 0.0083 | -0.9917 |
| ENST00000528650 | TBX3 | 0.006241 | -0.99376 |
| ENST00000528650 | AHSP | 1.93E-04 | -0.99981 |
| ENST00000528650 | KRT83 | 0.009704 | -0.9903 |
| ENST00000528650 | AC092073.1 | 0.0025 | -0.9975 |
| ENST00000528650 | CATG00000012021.1 | 0.004298 | 0.995702 |
| ENST00000528650 | TGFBR3 | 0.005514 | -0.99449 |
| ENST00000528650 | ESRRB | 0.007626 | -0.99237 |
| ENST00000528650 | IFT122 | 0.009452 | -0.99055 |
| ENST00000528650 | SIGLEC7 | 0.004839 | -0.99516 |
| ENST00000528650 | CATG00000034210.1 | 0.00403 | -0.99597 |
| ENST00000528650 | RRBP1 | 0.002429 | 0.997571 |
| ENST00000528650 | MORN3 | 0.00476 | 0.99524 |
| ENST00000528650 | VPS13A | 0.00935 | -0.99065 |
| ENST00000528650 | FGF9 | 0.009423 | 0.990577 |
| ENST00000528650 | CAPN15 | 0.003956 | -0.99604 |
| ENST00000528650 | FAM151A | 0.007235 | -0.99277 |
| T045868 | B4GALNT1 | 0.006494 | -0.99351 |
| T045868 | ZFY | 0.002552 | -0.99745 |
| T045868 | S1PR5 | 0.007031 | -0.99297 |
| T045868 | RAB8A | 0.006543 | -0.99346 |
| T045868 | EEF1B2 | 0.009922 | 0.990078 |
| T045868 | SSX1 | 0.00231 | 0.99769 |
| T045868 | WSCD2 | 0.005413 | 0.994587 |
| T045868 | GRIN3B | 0.001029 | -0.99897 |
| T045868 | PTPRK | 0.005893 | 0.994107 |
| T045868 | DEFA4 | 3.62E-04 | 0.999638 |
| FTMT23800001993 | ACTL7B | 0.008096 | -0.9919 |
| FTMT23800001993 | FBXL16 | 0.002185 | 0.997815 |
| FTMT23800001993 | MED12L | 0.008667 | -0.99133 |
| FTMT23800001993 | ZDBF2 | 0.009079 | -0.99092 |
| FTMT23800001993 | IGFL2 | 0.005037 | 0.994963 |
| FTMT23800001993 | S100P | 0.003602 | -0.9964 |
| ENST00000479612 | TMEM255B | 0.004403 | 0.995597 |
| ENST00000479612 | POMT2 | 0.003333 | -0.99667 |
| ENST00000479612 | ZNF77 | 0.008107 | 0.991893 |
| ENST00000479612 | INHBA | 0.002607 | -0.99739 |
| ENST00000479612 | CATG00000063086.1 | 0.001792 | -0.99821 |
| ENST00000479612 | PPP3R2 | 0.007022 | -0.99298 |
| ENST00000479612 | CATG00000068640.1 | 0.005718 | -0.99428 |
| T314159 | PHF13 | 0.005078 | -0.99492 |
| T314159 | CATG00000107403.1 | 0.009067 | -0.99093 |
| T314159 | OR10H1 | 0.0037 | -0.9963 |
| T239183 | HEYL | 0.003248 | -0.99675 |
| T239183 | 11-Mar | 0.0043 | 0.9957 |
| T239183 | TMEM236 | 0.002736 | 0.997264 |
| T239183 | CPLX1 | 0.005258 | 0.994742 |
| T239183 | LILRB5 | 0.007557 | -0.99244 |
| T239183 | GMEB2 | 0.002676 | 0.997324 |
| T239183 | APOBR | 0.00808 | 0.99192 |
| T239183 | OR13A1 | 0.008433 | 0.991567 |
| T239183 | ANKHD1 | 0.004138 | 0.995862 |
| T239183 | SMUG1 | 0.004706 | 0.995294 |
| T239183 | PAQR5 | 0.009918 | -0.99008 |
| T239183 | C3orf84 | 0.00437 | -0.99563 |
| T239183 | HEYL | 0.003248 | -0.99675 |
| T239183 | 11-Mar | 0.0043 | 0.9957 |
| T239183 | TMEM236 | 0.002736 | 0.997264 |
| T239183 | CPLX1 | 0.005258 | 0.994742 |
| T239183 | LILRB5 | 0.007557 | -0.99244 |
| T239183 | GMEB2 | 0.002676 | 0.997324 |
| T239183 | APOBR | 0.00808 | 0.99192 |
| T239183 | OR13A1 | 0.008433 | 0.991567 |
| T239183 | ANKHD1 | 0.004138 | 0.995862 |
| T239183 | SMUG1 | 0.004706 | 0.995294 |
| T239183 | PAQR5 | 0.009918 | -0.99008 |
| T239183 | C3orf84 | 0.00437 | -0.99563 |
| T239183 | HEYL | 0.003248 | -0.99675 |
| T239183 | 11-Mar | 0.0043 | 0.9957 |
| T239183 | TMEM236 | 0.002736 | 0.997264 |
| T239183 | CPLX1 | 0.005258 | 0.994742 |
| T239183 | LILRB5 | 0.007557 | -0.99244 |
| T239183 | GMEB2 | 0.002676 | 0.997324 |
| T239183 | APOBR | 0.00808 | 0.99192 |
| T239183 | OR13A1 | 0.008433 | 0.991567 |
| T239183 | ANKHD1 | 0.004138 | 0.995862 |
| T239183 | SMUG1 | 0.004706 | 0.995294 |
| T239183 | PAQR5 | 0.009918 | -0.99008 |
| T239183 | C3orf84 | 0.00437 | -0.99563 |
| T239183 | HEYL | 0.003248 | -0.99675 |
| T239183 | 11-Mar | 0.0043 | 0.9957 |
| T239183 | TMEM236 | 0.002736 | 0.997264 |
| T239183 | CPLX1 | 0.005258 | 0.994742 |
| T239183 | LILRB5 | 0.007557 | -0.99244 |
| T239183 | GMEB2 | 0.002676 | 0.997324 |
| T239183 | APOBR | 0.00808 | 0.99192 |
| T239183 | OR13A1 | 0.008433 | 0.991567 |
| T239183 | ANKHD1 | 0.004138 | 0.995862 |
| T239183 | SMUG1 | 0.004706 | 0.995294 |
| T239183 | PAQR5 | 0.009918 | -0.99008 |
| T239183 | C3orf84 | 0.00437 | -0.99563 |
| T239183 | HEYL | 0.003248 | -0.99675 |
| T239183 | 11-Mar | 0.0043 | 0.9957 |
| T239183 | TMEM236 | 0.002736 | 0.997264 |
| T239183 | CPLX1 | 0.005258 | 0.994742 |
| T239183 | LILRB5 | 0.007557 | -0.99244 |
| T239183 | GMEB2 | 0.002676 | 0.997324 |
| T239183 | APOBR | 0.00808 | 0.99192 |
| T239183 | OR13A1 | 0.008433 | 0.991567 |
| T239183 | ANKHD1 | 0.004138 | 0.995862 |
| T239183 | SMUG1 | 0.004706 | 0.995294 |
| T239183 | PAQR5 | 0.009918 | -0.99008 |
| T239183 | C3orf84 | 0.00437 | -0.99563 |
| ENST00000589967 | PZP | 4.25E-04 | 0.999575 |
| ENST00000589967 | CLEC4D | 0.004425 | 0.995575 |
| ENST00000589967 | ALS2CR12 | 0.00661 | -0.99339 |
| ENST00000557475 | ZNF185 | 0.00969 | -0.99031 |
| ENST00000557475 | NFATC2 | 0.00437 | 0.99563 |
| ENST00000557475 | IFNA6 | 0.008741 | -0.99126 |
| ENST00000557475 | AK4 | 0.009992 | 0.990008 |
| ENST00000557475 | LYPD3 | 0.009826 | 0.990174 |
| ENST00000557475 | ACER1 | 0.007513 | -0.99249 |
| ENST00000557475 | WDR60 | 0.006239 | 0.993761 |
| ENST00000458463 | RNF207 | 0.009983 | -0.99002 |
| ENST00000458463 | PLEKHA2 | 0.003315 | 0.996685 |
| ENST00000458463 | PLAG1 | 0.007996 | 0.992004 |
| ENST00000458463 | VNN3 | 0.009578 | -0.99042 |
| ENST00000458463 | DYNC2H1 | 0.004736 | -0.99526 |
| ENST00000458463 | RNF207 | 0.009983 | -0.99002 |
| ENST00000458463 | PLEKHA2 | 0.003315 | 0.996685 |
| ENST00000458463 | PLAG1 | 0.007996 | 0.992004 |
| ENST00000458463 | VNN3 | 0.009578 | -0.99042 |
| ENST00000458463 | DYNC2H1 | 0.004736 | -0.99526 |
| ENST00000458463 | RNF207 | 0.009983 | -0.99002 |
| ENST00000458463 | PLEKHA2 | 0.003315 | 0.996685 |
| ENST00000458463 | PLAG1 | 0.007996 | 0.992004 |
| ENST00000458463 | VNN3 | 0.009578 | -0.99042 |
| ENST00000458463 | DYNC2H1 | 0.004736 | -0.99526 |
| ENST00000608605 | GNL1 | 0.007156 | 0.992844 |
| ENST00000608605 | DEAF1 | 0.001206 | -0.99879 |
| ENST00000608605 | CLEC1A | 0.006597 | 0.993403 |
| ENST00000608605 | HMGXB4 | 0.008814 | 0.991186 |
| ENST00000608605 | FNDC10 | 0.005654 | -0.99435 |
| ENST00000608605 | VRK3 | 0.006552 | -0.99345 |
| ENST00000608605 | RPL11 | 0.007072 | 0.992928 |
| ENST00000608605 | BLCAP | 0.007647 | -0.99235 |
| ENST00000608605 | FANCD2OS | 0.003499 | -0.9965 |
| ENST00000608605 | PNPLA1 | 9.40E-04 | -0.99906 |
| ENST00000608605 | IDH1 | 0.002814 | 0.997186 |
| ENST00000608605 | TAF8 | 0.009483 | 0.990517 |
| ENST00000608605 | ACSL6 | 0.006982 | -0.99302 |
| ENST00000608605 | USP9X | 0.004049 | -0.99595 |
| ENST00000608605 | ZNF431 | 6.69E-04 | -0.99933 |
| ENST00000419640 | TRIM73 | 0.005261 | -0.99474 |
| ENST00000419640 | POMT2 | 0.009341 | 0.990659 |
| ENST00000419640 | ZNF77 | 0.0095 | -0.9905 |
| ENST00000419640 | INHBA | 0.008396 | 0.991604 |
| ENST00000419640 | CATG00000063086.1 | 2.67E-05 | 0.999973 |
| ENST00000419640 | LTF | 0.002851 | 0.997149 |
| ENST00000419640 | WFDC1 | 0.005351 | 0.994649 |
| ENST00000419640 | CATG00000068640.1 | 0.00314 | 0.99686 |
| ENST00000419640 | TRIM73 | 0.005261 | -0.99474 |
| ENST00000419640 | POMT2 | 0.009341 | 0.990659 |
| ENST00000419640 | ZNF77 | 0.0095 | -0.9905 |
| ENST00000419640 | INHBA | 0.008396 | 0.991604 |
| ENST00000419640 | CATG00000063086.1 | 2.67E-05 | 0.999973 |
| ENST00000419640 | LTF | 0.002851 | 0.997149 |
| ENST00000419640 | WFDC1 | 0.005351 | 0.994649 |
| ENST00000419640 | CATG00000068640.1 | 0.00314 | 0.99686 |
| ENST00000419640 | TRIM73 | 0.005261 | -0.99474 |
| ENST00000419640 | POMT2 | 0.009341 | 0.990659 |
| ENST00000419640 | ZNF77 | 0.0095 | -0.9905 |
| ENST00000419640 | INHBA | 0.008396 | 0.991604 |
| ENST00000419640 | CATG00000063086.1 | 2.67E-05 | 0.999973 |
| ENST00000419640 | LTF | 0.002851 | 0.997149 |
| ENST00000419640 | WFDC1 | 0.005351 | 0.994649 |
| ENST00000419640 | CATG00000068640.1 | 0.00314 | 0.99686 |
| ENST00000419640 | TRIM73 | 0.005261 | -0.99474 |
| ENST00000419640 | POMT2 | 0.009341 | 0.990659 |
| ENST00000419640 | ZNF77 | 0.0095 | -0.9905 |
| ENST00000419640 | INHBA | 0.008396 | 0.991604 |
| ENST00000419640 | CATG00000063086.1 | 2.67E-05 | 0.999973 |
| ENST00000419640 | LTF | 0.002851 | 0.997149 |
| ENST00000419640 | WFDC1 | 0.005351 | 0.994649 |
| ENST00000419640 | CATG00000068640.1 | 0.00314 | 0.99686 |
| ENST00000419640 | TRIM73 | 0.005261 | -0.99474 |
| ENST00000419640 | POMT2 | 0.009341 | 0.990659 |
| ENST00000419640 | ZNF77 | 0.0095 | -0.9905 |
| ENST00000419640 | INHBA | 0.008396 | 0.991604 |
| ENST00000419640 | CATG00000063086.1 | 2.67E-05 | 0.999973 |
| ENST00000419640 | LTF | 0.002851 | 0.997149 |
| ENST00000419640 | WFDC1 | 0.005351 | 0.994649 |
| ENST00000419640 | CATG00000068640.1 | 0.00314 | 0.99686 |
| ENST00000419640 | TRIM73 | 0.005261 | -0.99474 |
| ENST00000419640 | POMT2 | 0.009341 | 0.990659 |
| ENST00000419640 | ZNF77 | 0.0095 | -0.9905 |
| ENST00000419640 | INHBA | 0.008396 | 0.991604 |
| ENST00000419640 | CATG00000063086.1 | 2.67E-05 | 0.999973 |
| ENST00000419640 | LTF | 0.002851 | 0.997149 |
| ENST00000419640 | WFDC1 | 0.005351 | 0.994649 |
| ENST00000419640 | CATG00000068640.1 | 0.00314 | 0.99686 |
| ENST00000460721 | DNASE1L2 | 0.009801 | 0.990199 |
| ENST00000460721 | ZC3H7B | 0.00312 | 0.99688 |
| ENST00000460721 | CRYM | 0.001209 | -0.99879 |
| ENST00000445551 | IL31RA | 0.008283 | -0.99172 |
| ENST00000445551 | NHEJ1 | 0.008957 | -0.99104 |
| ENST00000445551 | EHD4 | 0.009368 | 0.990632 |
| ENST00000445551 | PRKDC | 0.007931 | 0.992069 |
| ENST00000445551 | USF2 | 0.006413 | 0.993587 |
| ENST00000445551 | SMAP2 | 5.38E-05 | 0.999946 |
| ENST00000445551 | ISG15 | 0.00337 | -0.99663 |
| ENST00000532839 | AX748369 | 0.005794 | -0.99421 |
| ENST00000532839 | PNPLA2 | 0.004657 | -0.99534 |
| ENST00000532839 | PDCD2L | 0.003252 | 0.996748 |
| ENST00000532839 | FGD4 | 0.008255 | -0.99175 |
| ENST00000532839 | CATG00000108269.1 | 4.71E-04 | -0.99953 |
| ENST00000532839 | CYP27C1 | 0.005256 | -0.99474 |
| ENST00000532839 | AX748369 | 0.005794 | -0.99421 |
| ENST00000532839 | PNPLA2 | 0.004657 | -0.99534 |
| ENST00000532839 | PDCD2L | 0.003252 | 0.996748 |
| ENST00000532839 | FGD4 | 0.008255 | -0.99175 |
| ENST00000532839 | CATG00000108269.1 | 4.71E-04 | -0.99953 |
| ENST00000532839 | CYP27C1 | 0.005256 | -0.99474 |
| ENST00000532839 | AX748369 | 0.005794 | -0.99421 |
| ENST00000532839 | PNPLA2 | 0.004657 | -0.99534 |
| ENST00000532839 | PDCD2L | 0.003252 | 0.996748 |
| ENST00000532839 | FGD4 | 0.008255 | -0.99175 |
| ENST00000532839 | CATG00000108269.1 | 4.71E-04 | -0.99953 |
| ENST00000532839 | CYP27C1 | 0.005256 | -0.99474 |
| ENST00000532839 | AX748369 | 0.005794 | -0.99421 |
| ENST00000532839 | PNPLA2 | 0.004657 | -0.99534 |
| ENST00000532839 | PDCD2L | 0.003252 | 0.996748 |
| ENST00000532839 | FGD4 | 0.008255 | -0.99175 |
| ENST00000532839 | CATG00000108269.1 | 4.71E-04 | -0.99953 |
| ENST00000532839 | CYP27C1 | 0.005256 | -0.99474 |
| ENST00000532839 | AX748369 | 0.005794 | -0.99421 |
| ENST00000532839 | PNPLA2 | 0.004657 | -0.99534 |
| ENST00000532839 | PDCD2L | 0.003252 | 0.996748 |
| ENST00000532839 | FGD4 | 0.008255 | -0.99175 |
| ENST00000532839 | CATG00000108269.1 | 4.71E-04 | -0.99953 |
| ENST00000532839 | CYP27C1 | 0.005256 | -0.99474 |
| ENST00000532839 | AX748369 | 0.005794 | -0.99421 |
| ENST00000532839 | PNPLA2 | 0.004657 | -0.99534 |
| ENST00000532839 | PDCD2L | 0.003252 | 0.996748 |
| ENST00000532839 | FGD4 | 0.008255 | -0.99175 |
| ENST00000532839 | CATG00000108269.1 | 4.71E-04 | -0.99953 |
| ENST00000532839 | CYP27C1 | 0.005256 | -0.99474 |
| ENST00000532839 | AX748369 | 0.005794 | -0.99421 |
| ENST00000532839 | PNPLA2 | 0.004657 | -0.99534 |
| ENST00000532839 | PDCD2L | 0.003252 | 0.996748 |
| ENST00000532839 | FGD4 | 0.008255 | -0.99175 |
| ENST00000532839 | CATG00000108269.1 | 4.71E-04 | -0.99953 |
| ENST00000532839 | CYP27C1 | 0.005256 | -0.99474 |
| ENST00000532839 | AX748369 | 0.005794 | -0.99421 |
| ENST00000532839 | PNPLA2 | 0.004657 | -0.99534 |
| ENST00000532839 | PDCD2L | 0.003252 | 0.996748 |
| ENST00000532839 | FGD4 | 0.008255 | -0.99175 |
| ENST00000532839 | CATG00000108269.1 | 4.71E-04 | -0.99953 |
| ENST00000532839 | CYP27C1 | 0.005256 | -0.99474 |
| ENST00000532839 | AX748369 | 0.005794 | -0.99421 |
| ENST00000532839 | PNPLA2 | 0.004657 | -0.99534 |
| ENST00000532839 | PDCD2L | 0.003252 | 0.996748 |
| ENST00000532839 | FGD4 | 0.008255 | -0.99175 |
| ENST00000532839 | CATG00000108269.1 | 4.71E-04 | -0.99953 |
| ENST00000532839 | CYP27C1 | 0.005256 | -0.99474 |
| ENST00000532839 | AX748369 | 0.005794 | -0.99421 |
| ENST00000532839 | PNPLA2 | 0.004657 | -0.99534 |
| ENST00000532839 | PDCD2L | 0.003252 | 0.996748 |
| ENST00000532839 | FGD4 | 0.008255 | -0.99175 |
| ENST00000532839 | CATG00000108269.1 | 4.71E-04 | -0.99953 |
| ENST00000532839 | CYP27C1 | 0.005256 | -0.99474 |
| ENST00000532839 | AX748369 | 0.005794 | -0.99421 |
| ENST00000532839 | PNPLA2 | 0.004657 | -0.99534 |
| ENST00000532839 | PDCD2L | 0.003252 | 0.996748 |
| ENST00000532839 | FGD4 | 0.008255 | -0.99175 |
| ENST00000532839 | CATG00000108269.1 | 4.71E-04 | -0.99953 |
| ENST00000532839 | CYP27C1 | 0.005256 | -0.99474 |
| ENST00000532839 | AX748369 | 0.005794 | -0.99421 |
| ENST00000532839 | PNPLA2 | 0.004657 | -0.99534 |
| ENST00000532839 | PDCD2L | 0.003252 | 0.996748 |
| ENST00000532839 | FGD4 | 0.008255 | -0.99175 |
| ENST00000532839 | CATG00000108269.1 | 4.71E-04 | -0.99953 |
| ENST00000532839 | CYP27C1 | 0.005256 | -0.99474 |
| ENST00000532839 | AX748369 | 0.005794 | -0.99421 |
| ENST00000532839 | PNPLA2 | 0.004657 | -0.99534 |
| ENST00000532839 | PDCD2L | 0.003252 | 0.996748 |
| ENST00000532839 | FGD4 | 0.008255 | -0.99175 |
| ENST00000532839 | CATG00000108269.1 | 4.71E-04 | -0.99953 |
| ENST00000532839 | CYP27C1 | 0.005256 | -0.99474 |
| ENST00000532839 | AX748369 | 0.005794 | -0.99421 |
| ENST00000532839 | PNPLA2 | 0.004657 | -0.99534 |
| ENST00000532839 | PDCD2L | 0.003252 | 0.996748 |
| ENST00000532839 | FGD4 | 0.008255 | -0.99175 |
| ENST00000532839 | CATG00000108269.1 | 4.71E-04 | -0.99953 |
| ENST00000532839 | CYP27C1 | 0.005256 | -0.99474 |
| ENST00000532839 | AX748369 | 0.005794 | -0.99421 |
| ENST00000532839 | PNPLA2 | 0.004657 | -0.99534 |
| ENST00000532839 | PDCD2L | 0.003252 | 0.996748 |
| ENST00000532839 | FGD4 | 0.008255 | -0.99175 |
| ENST00000532839 | CATG00000108269.1 | 4.71E-04 | -0.99953 |
| ENST00000532839 | CYP27C1 | 0.005256 | -0.99474 |
| ENST00000532839 | AX748369 | 0.005794 | -0.99421 |
| ENST00000532839 | PNPLA2 | 0.004657 | -0.99534 |
| ENST00000532839 | PDCD2L | 0.003252 | 0.996748 |
| ENST00000532839 | FGD4 | 0.008255 | -0.99175 |
| ENST00000532839 | CATG00000108269.1 | 4.71E-04 | -0.99953 |
| ENST00000532839 | CYP27C1 | 0.005256 | -0.99474 |
| ENST00000532839 | AX748369 | 0.005794 | -0.99421 |
| ENST00000532839 | PNPLA2 | 0.004657 | -0.99534 |
| ENST00000532839 | PDCD2L | 0.003252 | 0.996748 |
| ENST00000532839 | FGD4 | 0.008255 | -0.99175 |
| ENST00000532839 | CATG00000108269.1 | 4.71E-04 | -0.99953 |
| ENST00000532839 | CYP27C1 | 0.005256 | -0.99474 |
| T167158 | VWA5A | 5.75E-04 | -0.99943 |
| T167158 | PLA1A | 0.009952 | 0.990048 |
| T167158 | TLR2 | 0.002922 | -0.99708 |
| T167158 | CATG00000107403.1 | 0.003493 | 0.996507 |
| NR_036608 | AL358113.1 | 0.001032 | 0.998968 |
| NR_036608 | AL358113.1 | 0.001032 | 0.998968 |
| NR_036608 | AL358113.1 | 0.001032 | 0.998968 |
| NR_036608 | AL358113.1 | 0.001032 | 0.998968 |
| NR_036608 | AL358113.1 | 0.001032 | 0.998968 |
| NR_036608 | AL358113.1 | 0.001032 | 0.998968 |
| NR_036608 | AL358113.1 | 0.001032 | 0.998968 |
| NR_036608 | AL358113.1 | 0.001032 | 0.998968 |
| NR_036608 | AL358113.1 | 0.001032 | 0.998968 |
| NR_036608 | AL358113.1 | 0.001032 | 0.998968 |
| NR_036608 | AL358113.1 | 0.001032 | 0.998968 |
| NR_036608 | AL358113.1 | 0.001032 | 0.998968 |
| ENST00000444178 | GBA | 0.002932 | 0.997068 |
| ENST00000444178 | SLC7A4 | 0.00204 | 0.99796 |
| ENST00000444178 | SMPD1 | 0.005833 | 0.994167 |
| ENST00000444178 | SAMD1 | 0.004013 | -0.99599 |
| ENST00000444178 | ZMYND15 | 0.006662 | 0.993338 |
| ENST00000444178 | CATG00000057824.1 | 8.95E-05 | 0.99991 |
| ENST00000444178 | CDK11B | 0.005378 | -0.99462 |
| ENST00000444178 | IQCF5 | 0.006217 | 0.993783 |
| ENST00000444178 | KLF6 | 0.007044 | -0.99296 |
| ENST00000444178 | MAP1LC3A | 0.002811 | 0.997189 |
| ENST00000444178 | LBP | 0.005835 | 0.994165 |
| ENST00000444178 | DNAL4 | 0.009493 | -0.99051 |
| ENST00000444178 | FAM53B | 0.002591 | -0.99741 |
| ENST00000444178 | SH3RF3 | 0.009775 | -0.99022 |
| ENST00000444178 | LDHB | 4.14E-04 | -0.99959 |
| FTMT21000001953 | TAS2R42 | 0.003138 | 0.996862 |
| FTMT21000001953 | B3GNT3 | 0.008946 | -0.99105 |
| FTMT21000001953 | GRINA | 0.001588 | 0.998412 |
| FTMT21000001953 | HMGB2 | 0.008283 | -0.99172 |
| FTMT21000001953 | PSRC1 | 0.005302 | -0.9947 |
| FTMT21000001953 | PLIN4 | 7.45E-04 | 0.999255 |
| FTMT21000001953 | LILRB1 | 0.001543 | -0.99846 |
| FTMT21000001953 | RASL10A | 0.002828 | -0.99717 |
| FTMT21000001953 | CXXC1 | 0.003093 | -0.99691 |
| FTMT21000001953 | CATG00000053512.1 | 0.009504 | -0.9905 |
| FTMT21000001953 | HLF | 0.00629 | 0.99371 |
| FTMT21000001953 | SRRD | 0.009791 | 0.990209 |
| FTMT21000001953 | TPTE | 0.009844 | 0.990156 |
| FTMT21000001953 | NFX1 | 0.001825 | -0.99818 |
| FTMT21000001953 | CATG00000012021.1 | 0.007266 | -0.99273 |
| FTMT21000001953 | FYB2 | 0.005194 | 0.994806 |
| FTMT21000001953 | PEA15 | 0.001698 | 0.998302 |
| FTMT21000001953 | ESRRB | 0.004153 | 0.995847 |
| FTMT21000001953 | IFT122 | 0.002231 | 0.997769 |
| FTMT21000001953 | SLC12A3 | 0.004554 | -0.99545 |
| FTMT21000001953 | SIGLEC7 | 0.005422 | 0.994578 |
| FTMT21000001953 | WNT8B | 8.06E-04 | 0.999194 |
| FTMT21000001953 | UNC5C | 0.001201 | -0.9988 |
| FTMT21000001953 | AGO2 | 0.009116 | 0.990884 |
| FTMT21000001953 | OXT | 0.008813 | 0.991187 |
| FTMT21000001953 | FAM174A | 0.005545 | -0.99445 |
| FTMT21000001953 | CFAP410 | 0.004073 | 0.995927 |
| FTMT21000001953 | HPS1 | 2.29E-04 | 0.999771 |
| FTMT21000001953 | CCDC174 | 0.006588 | -0.99341 |
| FTMT21000001953 | FAM151A | 0.003942 | 0.996058 |
| FTMT21000002507 | OR5T2 | 0.006623 | -0.99338 |
| ENST00000415655 | PZP | 0.003544 | -0.99646 |
| ENST00000415655 | CLEC4D | 0.00172 | -0.99828 |
| ENST00000415655 | CLRN2 | 0.003044 | -0.99696 |
| ENST00000415655 | SLC11A1 | 0.008505 | -0.9915 |
| TCONS_00008604 | MMP1 | 0.004384 | -0.99562 |
| TCONS_00008604 | PARP6 | 0.008757 | 0.991243 |
| TCONS_00008604 | TTC38 | 0.003345 | -0.99666 |
| ENST00000596071 | LDLRAD4 | 0.004006 | 0.995994 |
| ENST00000596071 | NAAA | 0.005706 | 0.994294 |
| ENST00000596071 | RFC5 | 0.007461 | -0.99254 |
| ENST00000596071 | DLEC1 | 0.001823 | -0.99818 |
| ENST00000596071 | LDLRAD4 | 0.004006 | 0.995994 |
| ENST00000596071 | NAAA | 0.005706 | 0.994294 |
| ENST00000596071 | RFC5 | 0.007461 | -0.99254 |
| ENST00000596071 | DLEC1 | 0.001823 | -0.99818 |
| ENST00000609365 | EPB41L1 | 0.005281 | -0.99472 |
| ENST00000609365 | CACHD1 | 0.007247 | -0.99275 |
| ENST00000609365 | INSC | 2.49E-04 | -0.99975 |
| ENST00000609365 | EIF1AD | 0.006996 | -0.993 |
| ENST00000609365 | CATG00000047316.1 | 0.009122 | -0.99088 |
| ENST00000609365 | SAMD1 | 0.00898 | 0.99102 |
| ENST00000609365 | CAPRIN2 | 0.007914 | -0.99209 |
| ENST00000609365 | CATG00000089121.1 | 0.005701 | 0.994299 |
| ENST00000609365 | OAS1 | 0.002503 | -0.9975 |
| ENST00000609365 | ZMYND15 | 0.008705 | -0.9913 |
| ENST00000609365 | IQCF5 | 0.005525 | -0.99448 |
| ENST00000609365 | CSNK1A1 | 0.002585 | 0.997415 |
| ENST00000609365 | DNAL4 | 0.005408 | 0.994592 |
| ENST00000609365 | ZNF587B | 0.002284 | 0.997716 |
| ENST00000609365 | RPL17 | 0.003508 | 0.996492 |
| ENST00000609365 | ZNF37A | 0.008037 | 0.991963 |
| ENST00000609365 | WAPL | 9.84E-04 | 0.999016 |
| ENST00000609365 | PLCL1 | 0.008496 | 0.991504 |
| ENST00000609365 | AL627171.2 | 0.004505 | 0.995495 |
| TCONS_00016461 | RHEB | 0.007284 | 0.992716 |
| TCONS_00016461 | CATG00000060074.1 | 0.004978 | 0.995022 |
| TCONS_00016461 | VWA5A | 0.008624 | 0.991376 |
| TCONS_00016461 | UFC1 | 0.009338 | 0.990662 |
| TCONS_00016461 | CATG00000051841.1 | 0.007792 | -0.99221 |
| TCONS_00016461 | ANKS4B | 0.007678 | -0.99232 |
| ENST00000444488 | CATG00000086946.1 | 0.009218 | -0.99078 |
| ENST00000444488 | GATA4 | 0.005952 | -0.99405 |
| ENST00000444488 | WT1 | 0.002941 | 0.997059 |
| ENST00000444488 | CATG00000039284.1 | 0.004326 | 0.995674 |
| ENST00000444488 | SCAMP3 | 0.005413 | 0.994587 |
| ENST00000574681 | HEYL | 0.006171 | -0.99383 |
| ENST00000574681 | RPS7 | 0.002606 | -0.99739 |
| ENST00000574681 | 11-Mar | 0.001205 | 0.998795 |
| ENST00000574681 | LILRB5 | 0.002499 | -0.9975 |
| ENST00000574681 | PELP1 | 0.007245 | -0.99275 |
| ENST00000574681 | GMEB2 | 0.002179 | 0.997821 |
| ENST00000574681 | GRTP1 | 0.004853 | -0.99515 |
| ENST00000574681 | SMUG1 | 0.007988 | 0.992012 |
| ENST00000574681 | CLEC4E | 0.007058 | -0.99294 |
| ENST00000574681 | HEYL | 0.006171 | -0.99383 |
| ENST00000574681 | RPS7 | 0.002606 | -0.99739 |
| ENST00000574681 | 11-Mar | 0.001205 | 0.998795 |
| ENST00000574681 | LILRB5 | 0.002499 | -0.9975 |
| ENST00000574681 | PELP1 | 0.007245 | -0.99275 |
| ENST00000574681 | GMEB2 | 0.002179 | 0.997821 |
| ENST00000574681 | GRTP1 | 0.004853 | -0.99515 |
| ENST00000574681 | SMUG1 | 0.007988 | 0.992012 |
| ENST00000574681 | CLEC4E | 0.007058 | -0.99294 |
| ENST00000574681 | HEYL | 0.006171 | -0.99383 |
| ENST00000574681 | RPS7 | 0.002606 | -0.99739 |
| ENST00000574681 | 11-Mar | 0.001205 | 0.998795 |
| ENST00000574681 | LILRB5 | 0.002499 | -0.9975 |
| ENST00000574681 | PELP1 | 0.007245 | -0.99275 |
| ENST00000574681 | GMEB2 | 0.002179 | 0.997821 |
| ENST00000574681 | GRTP1 | 0.004853 | -0.99515 |
| ENST00000574681 | SMUG1 | 0.007988 | 0.992012 |
| ENST00000574681 | CLEC4E | 0.007058 | -0.99294 |
| ENST00000574681 | HEYL | 0.006171 | -0.99383 |
| ENST00000574681 | RPS7 | 0.002606 | -0.99739 |
| ENST00000574681 | 11-Mar | 0.001205 | 0.998795 |
| ENST00000574681 | LILRB5 | 0.002499 | -0.9975 |
| ENST00000574681 | PELP1 | 0.007245 | -0.99275 |
| ENST00000574681 | GMEB2 | 0.002179 | 0.997821 |
| ENST00000574681 | GRTP1 | 0.004853 | -0.99515 |
| ENST00000574681 | SMUG1 | 0.007988 | 0.992012 |
| ENST00000574681 | CLEC4E | 0.007058 | -0.99294 |
| T155718 | GSTM5 | 0.006634 | 0.993366 |
| T155718 | ASB11 | 0.004805 | -0.9952 |
| T155718 | FAM186A | 0.003317 | 0.996683 |
| T155718 | PGAM5 | 0.005675 | 0.994325 |
| ENST00000413745 | DMAC2 | 0.002446 | -0.99755 |
| ENST00000413745 | RSL1D1 | 0.009207 | 0.990793 |
| ENST00000413745 | GORASP1 | 0.002277 | 0.997723 |
| ENST00000413745 | ABCB8 | 0.006729 | -0.99327 |
| ENST00000413745 | HERPUD2 | 0.003281 | 0.996719 |
| ENST00000413745 | CPLX1 | 0.009204 | 0.990796 |
| ENST00000413745 | SLC22A12 | 0.005087 | -0.99491 |
| ENST00000413745 | FCHSD2 | 0.002604 | 0.997396 |
| ENST00000413745 | ZNF579 | 0.002013 | -0.99799 |
| ENST00000413745 | OR13A1 | 0.006937 | 0.993063 |
| ENST00000413745 | PTPN23 | 0.006461 | -0.99354 |
| ENST00000413745 | TRIM47 | 0.004833 | -0.99517 |
| ENST00000413745 | DHDH | 0.003419 | -0.99658 |
| ENST00000413745 | DCUN1D1 | 0.004441 | 0.995559 |
| ENST00000413745 | ATG4C | 0.009148 | -0.99085 |
| ENST00000413745 | DMAC2 | 0.002446 | -0.99755 |
| ENST00000413745 | RSL1D1 | 0.009207 | 0.990793 |
| ENST00000413745 | GORASP1 | 0.002277 | 0.997723 |
| ENST00000413745 | ABCB8 | 0.006729 | -0.99327 |
| ENST00000413745 | HERPUD2 | 0.003281 | 0.996719 |
| ENST00000413745 | CPLX1 | 0.009204 | 0.990796 |
| ENST00000413745 | SLC22A12 | 0.005087 | -0.99491 |
| ENST00000413745 | FCHSD2 | 0.002604 | 0.997396 |
| ENST00000413745 | ZNF579 | 0.002013 | -0.99799 |
| ENST00000413745 | OR13A1 | 0.006937 | 0.993063 |
| ENST00000413745 | PTPN23 | 0.006461 | -0.99354 |
| ENST00000413745 | TRIM47 | 0.004833 | -0.99517 |
| ENST00000413745 | DHDH | 0.003419 | -0.99658 |
| ENST00000413745 | DCUN1D1 | 0.004441 | 0.995559 |
| ENST00000413745 | ATG4C | 0.009148 | -0.99085 |
| T266344 | TMED3 | 0.008741 | -0.99126 |
| T266344 | ACOT12 | 0.006036 | 0.993964 |
| T266344 | VRK3 | 0.00826 | 0.99174 |
| T266344 | PALM3 | 0.005402 | -0.9946 |
| T266344 | BLCAP | 0.007284 | 0.992716 |
| T266344 | BCAS4 | 0.002354 | 0.997646 |
| T266344 | CATG00000063823.1 | 0.009857 | 0.990143 |
| T266344 | OR8B4 | 0.00455 | -0.99545 |
| T266344 | ACSL6 | 0.007128 | 0.992872 |
| T266344 | MYH4 | 0.009957 | 0.990043 |
| T266344 | KNCN | 0.007361 | 0.992639 |
| T266344 | FANCL | 0.004586 | 0.995414 |
| T266344 | CAMK2N2 | 0.00974 | 0.99026 |
| ENST00000606991 | PTPN6 | 0.00815 | 0.99185 |
| ENST00000606991 | HMGXB4 | 0.007377 | 0.992623 |
| ENST00000606991 | DNAJA4 | 0.002655 | -0.99734 |
| ENST00000606991 | GPR25 | 0.009332 | -0.99067 |
| ENST00000606991 | RPL11 | 0.009821 | 0.990179 |
| ENST00000606991 | PMPCA | 0.001356 | -0.99864 |
| ENST00000606991 | CAPNS1 | 0.002531 | 0.997469 |
| ENST00000606991 | SHC2 | 0.006065 | -0.99393 |
| ENST00000606991 | KRTAP4-7 | 0.001118 | -0.99888 |
| ENST00000606991 | PTPN6 | 0.00815 | 0.99185 |
| ENST00000606991 | HMGXB4 | 0.007377 | 0.992623 |
| ENST00000606991 | DNAJA4 | 0.002655 | -0.99734 |
| ENST00000606991 | GPR25 | 0.009332 | -0.99067 |
| ENST00000606991 | RPL11 | 0.009821 | 0.990179 |
| ENST00000606991 | PMPCA | 0.001356 | -0.99864 |
| ENST00000606991 | CAPNS1 | 0.002531 | 0.997469 |
| ENST00000606991 | SHC2 | 0.006065 | -0.99393 |
| ENST00000606991 | KRTAP4-7 | 0.001118 | -0.99888 |
| ENST00000606991 | PTPN6 | 0.00815 | 0.99185 |
| ENST00000606991 | HMGXB4 | 0.007377 | 0.992623 |
| ENST00000606991 | DNAJA4 | 0.002655 | -0.99734 |
| ENST00000606991 | GPR25 | 0.009332 | -0.99067 |
| ENST00000606991 | RPL11 | 0.009821 | 0.990179 |
| ENST00000606991 | PMPCA | 0.001356 | -0.99864 |
| ENST00000606991 | CAPNS1 | 0.002531 | 0.997469 |
| ENST00000606991 | SHC2 | 0.006065 | -0.99393 |
| ENST00000606991 | KRTAP4-7 | 0.001118 | -0.99888 |
| T032329 | RRAS2 | 0.005471 | -0.99453 |
| FTMT24000005423 | DGAT2 | 0.00486 | -0.99514 |
| FTMT24000005423 | PTPRN | 0.003198 | 0.996802 |
| FTMT24000005423 | EHD4 | 0.001109 | -0.99889 |
| FTMT24000005423 | PRKDC | 0.009376 | -0.99062 |
| ENST00000447423 | IL17RA | 0.005494 | -0.99451 |
| ENST00000447423 | AHSP | 6.46E-04 | -0.99935 |
| ENST00000447423 | AC092073.1 | 6.55E-04 | -0.99934 |
| ENST00000447423 | CATG00000012021.1 | 0.009771 | 0.990229 |
| ENST00000447423 | TGFBR3 | 0.002479 | -0.99752 |
| ENST00000447423 | LYPD1 | 0.008598 | -0.9914 |
| ENST00000447423 | CATG00000034210.1 | 0.00685 | -0.99315 |
| ENST00000447423 | COA5 | 0.008846 | 0.991154 |
| ENST00000447423 | SLC6A5 | 0.005727 | -0.99427 |
| ENST00000447423 | RRBP1 | 6.92E-04 | 0.999308 |
| ENST00000447423 | MORN3 | 0.002778 | 0.997222 |
| ENST00000447423 | VPS13A | 0.004107 | -0.99589 |
| ENST00000447423 | CAPN15 | 0.00411 | -0.99589 |
| ENST00000447423 | IL17RA | 0.005494 | -0.99451 |
| ENST00000447423 | AHSP | 6.46E-04 | -0.99935 |
| ENST00000447423 | AC092073.1 | 6.55E-04 | -0.99934 |
| ENST00000447423 | CATG00000012021.1 | 0.009771 | 0.990229 |
| ENST00000447423 | TGFBR3 | 0.002479 | -0.99752 |
| ENST00000447423 | LYPD1 | 0.008598 | -0.9914 |
| ENST00000447423 | CATG00000034210.1 | 0.00685 | -0.99315 |
| ENST00000447423 | COA5 | 0.008846 | 0.991154 |
| ENST00000447423 | SLC6A5 | 0.005727 | -0.99427 |
| ENST00000447423 | RRBP1 | 6.92E-04 | 0.999308 |
| ENST00000447423 | MORN3 | 0.002778 | 0.997222 |
| ENST00000447423 | VPS13A | 0.004107 | -0.99589 |
| ENST00000447423 | CAPN15 | 0.00411 | -0.99589 |
| ENST00000447423 | IL17RA | 0.005494 | -0.99451 |
| ENST00000447423 | AHSP | 6.46E-04 | -0.99935 |
| ENST00000447423 | AC092073.1 | 6.55E-04 | -0.99934 |
| ENST00000447423 | CATG00000012021.1 | 0.009771 | 0.990229 |
| ENST00000447423 | TGFBR3 | 0.002479 | -0.99752 |
| ENST00000447423 | LYPD1 | 0.008598 | -0.9914 |
| ENST00000447423 | CATG00000034210.1 | 0.00685 | -0.99315 |
| ENST00000447423 | COA5 | 0.008846 | 0.991154 |
| ENST00000447423 | SLC6A5 | 0.005727 | -0.99427 |
| ENST00000447423 | RRBP1 | 6.92E-04 | 0.999308 |
| ENST00000447423 | MORN3 | 0.002778 | 0.997222 |
| ENST00000447423 | VPS13A | 0.004107 | -0.99589 |
| ENST00000447423 | CAPN15 | 0.00411 | -0.99589 |
| ENST00000447423 | IL17RA | 0.005494 | -0.99451 |
| ENST00000447423 | AHSP | 6.46E-04 | -0.99935 |
| ENST00000447423 | AC092073.1 | 6.55E-04 | -0.99934 |
| ENST00000447423 | CATG00000012021.1 | 0.009771 | 0.990229 |
| ENST00000447423 | TGFBR3 | 0.002479 | -0.99752 |
| ENST00000447423 | LYPD1 | 0.008598 | -0.9914 |
| ENST00000447423 | CATG00000034210.1 | 0.00685 | -0.99315 |
| ENST00000447423 | COA5 | 0.008846 | 0.991154 |
| ENST00000447423 | SLC6A5 | 0.005727 | -0.99427 |
| ENST00000447423 | RRBP1 | 6.92E-04 | 0.999308 |
| ENST00000447423 | MORN3 | 0.002778 | 0.997222 |
| ENST00000447423 | VPS13A | 0.004107 | -0.99589 |
| ENST00000447423 | CAPN15 | 0.00411 | -0.99589 |
| ENST00000447423 | IL17RA | 0.005494 | -0.99451 |
| ENST00000447423 | AHSP | 6.46E-04 | -0.99935 |
| ENST00000447423 | AC092073.1 | 6.55E-04 | -0.99934 |
| ENST00000447423 | CATG00000012021.1 | 0.009771 | 0.990229 |
| ENST00000447423 | TGFBR3 | 0.002479 | -0.99752 |
| ENST00000447423 | LYPD1 | 0.008598 | -0.9914 |
| ENST00000447423 | CATG00000034210.1 | 0.00685 | -0.99315 |
| ENST00000447423 | COA5 | 0.008846 | 0.991154 |
| ENST00000447423 | SLC6A5 | 0.005727 | -0.99427 |
| ENST00000447423 | RRBP1 | 6.92E-04 | 0.999308 |
| ENST00000447423 | MORN3 | 0.002778 | 0.997222 |
| ENST00000447423 | VPS13A | 0.004107 | -0.99589 |
| ENST00000447423 | CAPN15 | 0.00411 | -0.99589 |
| ENST00000447423 | IL17RA | 0.005494 | -0.99451 |
| ENST00000447423 | AHSP | 6.46E-04 | -0.99935 |
| ENST00000447423 | AC092073.1 | 6.55E-04 | -0.99934 |
| ENST00000447423 | CATG00000012021.1 | 0.009771 | 0.990229 |
| ENST00000447423 | TGFBR3 | 0.002479 | -0.99752 |
| ENST00000447423 | LYPD1 | 0.008598 | -0.9914 |
| ENST00000447423 | CATG00000034210.1 | 0.00685 | -0.99315 |
| ENST00000447423 | COA5 | 0.008846 | 0.991154 |
| ENST00000447423 | SLC6A5 | 0.005727 | -0.99427 |
| ENST00000447423 | RRBP1 | 6.92E-04 | 0.999308 |
| ENST00000447423 | MORN3 | 0.002778 | 0.997222 |
| ENST00000447423 | VPS13A | 0.004107 | -0.99589 |
| ENST00000447423 | CAPN15 | 0.00411 | -0.99589 |
| MICT00000361968 | TMED3 | 0.003805 | 0.996195 |
| MICT00000361968 | DEAF1 | 0.006136 | -0.99386 |
| MICT00000361968 | CLEC1A | 0.009106 | 0.990894 |
| MICT00000361968 | FNDC10 | 0.004224 | -0.99578 |
| MICT00000361968 | VRK3 | 0.004228 | -0.99577 |
| MICT00000361968 | BLCAP | 0.004863 | -0.99514 |
| MICT00000361968 | FANCD2OS | 0.00863 | -0.99137 |
| MICT00000361968 | PNPLA1 | 0.002102 | -0.9979 |
| MICT00000361968 | IDH1 | 0.00225 | 0.99775 |
| MICT00000361968 | ZNF607 | 0.00307 | -0.99693 |
| MICT00000361968 | OR6F1 | 0.005489 | -0.99451 |
| MICT00000361968 | ACSL6 | 0.003549 | -0.99645 |
| MICT00000361968 | ZNF431 | 0.00714 | -0.99286 |
| ENST00000602357 | GRINA | 0.006523 | -0.99348 |
| ENST00000602357 | HMGB2 | 0.003952 | 0.996048 |
| ENST00000602357 | PLIN4 | 0.001414 | -0.99859 |
| ENST00000602357 | LILRB1 | 0.007321 | 0.992679 |
| ENST00000602357 | GSTA4 | 0.004222 | -0.99578 |
| ENST00000602357 | CXXC1 | 3.50E-04 | 0.99965 |
| ENST00000602357 | TBX3 | 0.002258 | -0.99774 |
| ENST00000602357 | KRT83 | 0.001694 | -0.99831 |
| ENST00000602357 | SRRD | 0.009879 | -0.99012 |
| ENST00000602357 | TPTE | 0.006949 | -0.99305 |
| ENST00000602357 | NFX1 | 0.00917 | 0.99083 |
| ENST00000602357 | CATG00000012021.1 | 0.001845 | 0.998155 |
| ENST00000602357 | FYB2 | 0.006635 | -0.99337 |
| ENST00000602357 | CLEC5A | 0.008951 | -0.99105 |
| ENST00000602357 | PEA15 | 0.008792 | -0.99121 |
| ENST00000602357 | ESRRB | 6.07E-04 | -0.99939 |
| ENST00000602357 | GSG1L | 0.007059 | -0.99294 |
| ENST00000602357 | IFT122 | 0.002888 | -0.99711 |
| ENST00000602357 | PAK3 | 0.008106 | -0.99189 |
| ENST00000602357 | SIGLEC7 | 0.002649 | -0.99735 |
| ENST00000602357 | WNT8B | 0.007806 | -0.99219 |
| ENST00000602357 | CATG00000034210.1 | 0.008754 | -0.99125 |
| ENST00000602357 | UNC5C | 0.008859 | 0.991141 |
| ENST00000602357 | HPS1 | 0.005891 | -0.99411 |
| ENST00000602357 | CCDC174 | 5.28E-04 | 0.999472 |
| ENST00000602357 | FAM151A | 9.30E-04 | -0.99907 |
| ENST00000602357 | GRINA | 0.006523 | -0.99348 |
| ENST00000602357 | HMGB2 | 0.003952 | 0.996048 |
| ENST00000602357 | PLIN4 | 0.001414 | -0.99859 |
| ENST00000602357 | LILRB1 | 0.007321 | 0.992679 |
| ENST00000602357 | GSTA4 | 0.004222 | -0.99578 |
| ENST00000602357 | CXXC1 | 3.50E-04 | 0.99965 |
| ENST00000602357 | TBX3 | 0.002258 | -0.99774 |
| ENST00000602357 | KRT83 | 0.001694 | -0.99831 |
| ENST00000602357 | SRRD | 0.009879 | -0.99012 |
| ENST00000602357 | TPTE | 0.006949 | -0.99305 |
| ENST00000602357 | NFX1 | 0.00917 | 0.99083 |
| ENST00000602357 | CATG00000012021.1 | 0.001845 | 0.998155 |
| ENST00000602357 | FYB2 | 0.006635 | -0.99337 |
| ENST00000602357 | CLEC5A | 0.008951 | -0.99105 |
| ENST00000602357 | PEA15 | 0.008792 | -0.99121 |
| ENST00000602357 | ESRRB | 6.07E-04 | -0.99939 |
| ENST00000602357 | GSG1L | 0.007059 | -0.99294 |
| ENST00000602357 | IFT122 | 0.002888 | -0.99711 |
| ENST00000602357 | PAK3 | 0.008106 | -0.99189 |
| ENST00000602357 | SIGLEC7 | 0.002649 | -0.99735 |
| ENST00000602357 | WNT8B | 0.007806 | -0.99219 |
| ENST00000602357 | CATG00000034210.1 | 0.008754 | -0.99125 |
| ENST00000602357 | UNC5C | 0.008859 | 0.991141 |
| ENST00000602357 | HPS1 | 0.005891 | -0.99411 |
| ENST00000602357 | CCDC174 | 5.28E-04 | 0.999472 |
| ENST00000602357 | FAM151A | 9.30E-04 | -0.99907 |
| ENST00000602357 | GRINA | 0.006523 | -0.99348 |
| ENST00000602357 | HMGB2 | 0.003952 | 0.996048 |
| ENST00000602357 | PLIN4 | 0.001414 | -0.99859 |
| ENST00000602357 | LILRB1 | 0.007321 | 0.992679 |
| ENST00000602357 | GSTA4 | 0.004222 | -0.99578 |
| ENST00000602357 | CXXC1 | 3.50E-04 | 0.99965 |
| ENST00000602357 | TBX3 | 0.002258 | -0.99774 |
| ENST00000602357 | KRT83 | 0.001694 | -0.99831 |
| ENST00000602357 | SRRD | 0.009879 | -0.99012 |
| ENST00000602357 | TPTE | 0.006949 | -0.99305 |
| ENST00000602357 | NFX1 | 0.00917 | 0.99083 |
| ENST00000602357 | CATG00000012021.1 | 0.001845 | 0.998155 |
| ENST00000602357 | FYB2 | 0.006635 | -0.99337 |
| ENST00000602357 | CLEC5A | 0.008951 | -0.99105 |
| ENST00000602357 | PEA15 | 0.008792 | -0.99121 |
| ENST00000602357 | ESRRB | 6.07E-04 | -0.99939 |
| ENST00000602357 | GSG1L | 0.007059 | -0.99294 |
| ENST00000602357 | IFT122 | 0.002888 | -0.99711 |
| ENST00000602357 | PAK3 | 0.008106 | -0.99189 |
| ENST00000602357 | SIGLEC7 | 0.002649 | -0.99735 |
| ENST00000602357 | WNT8B | 0.007806 | -0.99219 |
| ENST00000602357 | CATG00000034210.1 | 0.008754 | -0.99125 |
| ENST00000602357 | UNC5C | 0.008859 | 0.991141 |
| ENST00000602357 | HPS1 | 0.005891 | -0.99411 |
| ENST00000602357 | CCDC174 | 5.28E-04 | 0.999472 |
| ENST00000602357 | FAM151A | 9.30E-04 | -0.99907 |
| ENST00000602357 | GRINA | 0.006523 | -0.99348 |
| ENST00000602357 | HMGB2 | 0.003952 | 0.996048 |
| ENST00000602357 | PLIN4 | 0.001414 | -0.99859 |
| ENST00000602357 | LILRB1 | 0.007321 | 0.992679 |
| ENST00000602357 | GSTA4 | 0.004222 | -0.99578 |
| ENST00000602357 | CXXC1 | 3.50E-04 | 0.99965 |
| ENST00000602357 | TBX3 | 0.002258 | -0.99774 |
| ENST00000602357 | KRT83 | 0.001694 | -0.99831 |
| ENST00000602357 | SRRD | 0.009879 | -0.99012 |
| ENST00000602357 | TPTE | 0.006949 | -0.99305 |
| ENST00000602357 | NFX1 | 0.00917 | 0.99083 |
| ENST00000602357 | CATG00000012021.1 | 0.001845 | 0.998155 |
| ENST00000602357 | FYB2 | 0.006635 | -0.99337 |
| ENST00000602357 | CLEC5A | 0.008951 | -0.99105 |
| ENST00000602357 | PEA15 | 0.008792 | -0.99121 |
| ENST00000602357 | ESRRB | 6.07E-04 | -0.99939 |
| ENST00000602357 | GSG1L | 0.007059 | -0.99294 |
| ENST00000602357 | IFT122 | 0.002888 | -0.99711 |
| ENST00000602357 | PAK3 | 0.008106 | -0.99189 |
| ENST00000602357 | SIGLEC7 | 0.002649 | -0.99735 |
| ENST00000602357 | WNT8B | 0.007806 | -0.99219 |
| ENST00000602357 | CATG00000034210.1 | 0.008754 | -0.99125 |
| ENST00000602357 | UNC5C | 0.008859 | 0.991141 |
| ENST00000602357 | HPS1 | 0.005891 | -0.99411 |
| ENST00000602357 | CCDC174 | 5.28E-04 | 0.999472 |
| ENST00000602357 | FAM151A | 9.30E-04 | -0.99907 |
| ENST00000602357 | GRINA | 0.006523 | -0.99348 |
| ENST00000602357 | HMGB2 | 0.003952 | 0.996048 |
| ENST00000602357 | PLIN4 | 0.001414 | -0.99859 |
| ENST00000602357 | LILRB1 | 0.007321 | 0.992679 |
| ENST00000602357 | GSTA4 | 0.004222 | -0.99578 |
| ENST00000602357 | CXXC1 | 3.50E-04 | 0.99965 |
| ENST00000602357 | TBX3 | 0.002258 | -0.99774 |
| ENST00000602357 | KRT83 | 0.001694 | -0.99831 |
| ENST00000602357 | SRRD | 0.009879 | -0.99012 |
| ENST00000602357 | TPTE | 0.006949 | -0.99305 |
| ENST00000602357 | NFX1 | 0.00917 | 0.99083 |
| ENST00000602357 | CATG00000012021.1 | 0.001845 | 0.998155 |
| ENST00000602357 | FYB2 | 0.006635 | -0.99337 |
| ENST00000602357 | CLEC5A | 0.008951 | -0.99105 |
| ENST00000602357 | PEA15 | 0.008792 | -0.99121 |
| ENST00000602357 | ESRRB | 6.07E-04 | -0.99939 |
| ENST00000602357 | GSG1L | 0.007059 | -0.99294 |
| ENST00000602357 | IFT122 | 0.002888 | -0.99711 |
| ENST00000602357 | PAK3 | 0.008106 | -0.99189 |
| ENST00000602357 | SIGLEC7 | 0.002649 | -0.99735 |
| ENST00000602357 | WNT8B | 0.007806 | -0.99219 |
| ENST00000602357 | CATG00000034210.1 | 0.008754 | -0.99125 |
| ENST00000602357 | UNC5C | 0.008859 | 0.991141 |
| ENST00000602357 | HPS1 | 0.005891 | -0.99411 |
| ENST00000602357 | CCDC174 | 5.28E-04 | 0.999472 |
| ENST00000602357 | FAM151A | 9.30E-04 | -0.99907 |
| ENST00000602357 | GRINA | 0.006523 | -0.99348 |
| ENST00000602357 | HMGB2 | 0.003952 | 0.996048 |
| ENST00000602357 | PLIN4 | 0.001414 | -0.99859 |
| ENST00000602357 | LILRB1 | 0.007321 | 0.992679 |
| ENST00000602357 | GSTA4 | 0.004222 | -0.99578 |
| ENST00000602357 | CXXC1 | 3.50E-04 | 0.99965 |
| ENST00000602357 | TBX3 | 0.002258 | -0.99774 |
| ENST00000602357 | KRT83 | 0.001694 | -0.99831 |
| ENST00000602357 | SRRD | 0.009879 | -0.99012 |
| ENST00000602357 | TPTE | 0.006949 | -0.99305 |
| ENST00000602357 | NFX1 | 0.00917 | 0.99083 |
| ENST00000602357 | CATG00000012021.1 | 0.001845 | 0.998155 |
| ENST00000602357 | FYB2 | 0.006635 | -0.99337 |
| ENST00000602357 | CLEC5A | 0.008951 | -0.99105 |
| ENST00000602357 | PEA15 | 0.008792 | -0.99121 |
| ENST00000602357 | ESRRB | 6.07E-04 | -0.99939 |
| ENST00000602357 | GSG1L | 0.007059 | -0.99294 |
| ENST00000602357 | IFT122 | 0.002888 | -0.99711 |
| ENST00000602357 | PAK3 | 0.008106 | -0.99189 |
| ENST00000602357 | SIGLEC7 | 0.002649 | -0.99735 |
| ENST00000602357 | WNT8B | 0.007806 | -0.99219 |
| ENST00000602357 | CATG00000034210.1 | 0.008754 | -0.99125 |
| ENST00000602357 | UNC5C | 0.008859 | 0.991141 |
| ENST00000602357 | HPS1 | 0.005891 | -0.99411 |
| ENST00000602357 | CCDC174 | 5.28E-04 | 0.999472 |
| ENST00000602357 | FAM151A | 9.30E-04 | -0.99907 |
| NR_135574 | LDLRAD4 | 0.007972 | 0.992028 |
| NR_135574 | GSTM5 | 0.002966 | -0.99703 |
| NR_135574 | DLEC1 | 0.005708 | -0.99429 |
| NR_135574 | ASB11 | 0.005102 | 0.994898 |
| NR_135574 | HSDL2 | 0.008509 | 0.991491 |
| NR_135574 | ZNRD1 | 0.004727 | -0.99527 |
| TCONS_00022456 | INTS3 | 0.002795 | 0.997205 |
| TCONS_00022456 | GYG2 | 0.007619 | 0.992381 |
| TCONS_00022456 | SPEF1 | 0.006472 | 0.993528 |
| TCONS_00022456 | TCF4 | 0.007258 | 0.992742 |
| TCONS_00022456 | AVPI1 | 0.009348 | 0.990652 |
| TCONS_00022456 | TMEM43 | 0.009381 | -0.99062 |
| T070259 | PZP | 0.003478 | -0.99652 |
| T070259 | CFAP77 | 0.0078 | -0.9922 |
| T070259 | ALS2CR12 | 0.005646 | 0.994354 |
| TCONS_00027944 | RPS7 | 0.008315 | 0.991685 |
| TCONS_00027944 | ACOT12 | 0.001264 | 0.998736 |
| TCONS_00027944 | SELENOP | 0.009691 | -0.99031 |
| TCONS_00027944 | BCAS4 | 0.0056 | 0.9944 |
| TCONS_00027944 | PROKR1 | 0.001426 | -0.99857 |
| TCONS_00027944 | PELP1 | 0.008441 | 0.991559 |
| TCONS_00027944 | OR8B4 | 0.001082 | -0.99892 |
| TCONS_00027944 | MYH4 | 7.18E-04 | 0.999282 |
| TCONS_00027944 | CLEC4E | 0.002755 | 0.997245 |
| TCONS_00027944 | FANCL | 0.001268 | 0.998732 |
| TCONS_00027944 | CAMK2N2 | 0.009171 | 0.990829 |
| ENST00000416696 | FTCD | 0.008182 | -0.99182 |
| ENST00000416696 | ZNF77 | 1.43E-04 | 0.999857 |
| ENST00000416696 | INHBA | 0.006823 | -0.99318 |
| ENST00000416696 | CATG00000063086.1 | 0.009503 | -0.9905 |
| ENST00000437331 | LDLRAD4 | 0.008764 | 0.991236 |
| ENST00000437331 | NAAA | 0.003046 | 0.996954 |
| ENST00000437331 | RFC5 | 0.004368 | -0.99563 |
| ENST00000437331 | DLEC1 | 0.00282 | -0.99718 |
| ENST00000437331 | DARS | 0.005265 | 0.994735 |
| ENST00000437331 | CATG00000038628.1 | 0.009771 | -0.99023 |
| ENST00000437331 | LDLRAD4 | 0.008764 | 0.991236 |
| ENST00000437331 | NAAA | 0.003046 | 0.996954 |
| ENST00000437331 | RFC5 | 0.004368 | -0.99563 |
| ENST00000437331 | DLEC1 | 0.00282 | -0.99718 |
| ENST00000437331 | DARS | 0.005265 | 0.994735 |
| ENST00000437331 | CATG00000038628.1 | 0.009771 | -0.99023 |
| T206895 | SPTY2D1OS | 0.002048 | 0.997952 |
| T206895 | KYAT1 | 0.001843 | 0.998157 |
| T206895 | LYL1 | 0.002023 | 0.997977 |
| T206895 | STEAP3 | 4.37E-04 | 0.999563 |
| T206895 | SLC13A4 | 0.006045 | 0.993955 |
| T206895 | TP53I13 | 0.00767 | 0.99233 |
| T206895 | HIC1 | 0.003858 | 0.996142 |
| T206895 | TFF2 | 0.001987 | 0.998013 |
| T206895 | ORC3 | 1.02E-04 | 0.999898 |
| T206895 | UGT2B28 | 0.004911 | 0.995089 |
| T206895 | ZNF664 | 0.007548 | -0.99245 |
| T206895 | ABL1 | 0.004639 | -0.99536 |
| T206895 | SPTY2D1OS | 0.002048 | 0.997952 |
| T206895 | KYAT1 | 0.001843 | 0.998157 |
| T206895 | LYL1 | 0.002023 | 0.997977 |
| T206895 | STEAP3 | 4.37E-04 | 0.999563 |
| T206895 | SLC13A4 | 0.006045 | 0.993955 |
| T206895 | TP53I13 | 0.00767 | 0.99233 |
| T206895 | HIC1 | 0.003858 | 0.996142 |
| T206895 | TFF2 | 0.001987 | 0.998013 |
| T206895 | ORC3 | 1.02E-04 | 0.999898 |
| T206895 | UGT2B28 | 0.004911 | 0.995089 |
| T206895 | ZNF664 | 0.007548 | -0.99245 |
| T206895 | ABL1 | 0.004639 | -0.99536 |
| T206895 | SPTY2D1OS | 0.002048 | 0.997952 |
| T206895 | KYAT1 | 0.001843 | 0.998157 |
| T206895 | LYL1 | 0.002023 | 0.997977 |
| T206895 | STEAP3 | 4.37E-04 | 0.999563 |
| T206895 | SLC13A4 | 0.006045 | 0.993955 |
| T206895 | TP53I13 | 0.00767 | 0.99233 |
| T206895 | HIC1 | 0.003858 | 0.996142 |
| T206895 | TFF2 | 0.001987 | 0.998013 |
| T206895 | ORC3 | 1.02E-04 | 0.999898 |
| T206895 | UGT2B28 | 0.004911 | 0.995089 |
| T206895 | ZNF664 | 0.007548 | -0.99245 |
| T206895 | ABL1 | 0.004639 | -0.99536 |
| T206895 | SPTY2D1OS | 0.002048 | 0.997952 |
| T206895 | KYAT1 | 0.001843 | 0.998157 |
| T206895 | LYL1 | 0.002023 | 0.997977 |
| T206895 | STEAP3 | 4.37E-04 | 0.999563 |
| T206895 | SLC13A4 | 0.006045 | 0.993955 |
| T206895 | TP53I13 | 0.00767 | 0.99233 |
| T206895 | HIC1 | 0.003858 | 0.996142 |
| T206895 | TFF2 | 0.001987 | 0.998013 |
| T206895 | ORC3 | 1.02E-04 | 0.999898 |
| T206895 | UGT2B28 | 0.004911 | 0.995089 |
| T206895 | ZNF664 | 0.007548 | -0.99245 |
| T206895 | ABL1 | 0.004639 | -0.99536 |
| T206895 | SPTY2D1OS | 0.002048 | 0.997952 |
| T206895 | KYAT1 | 0.001843 | 0.998157 |
| T206895 | LYL1 | 0.002023 | 0.997977 |
| T206895 | STEAP3 | 4.37E-04 | 0.999563 |
| T206895 | SLC13A4 | 0.006045 | 0.993955 |
| T206895 | TP53I13 | 0.00767 | 0.99233 |
| T206895 | HIC1 | 0.003858 | 0.996142 |
| T206895 | TFF2 | 0.001987 | 0.998013 |
| T206895 | ORC3 | 1.02E-04 | 0.999898 |
| T206895 | UGT2B28 | 0.004911 | 0.995089 |
| T206895 | ZNF664 | 0.007548 | -0.99245 |
| T206895 | ABL1 | 0.004639 | -0.99536 |
| ENST00000511634 | SCAMP2 | 0.007514 | 0.992486 |
| ENST00000511634 | EPB41L1 | 1.17E-04 | 0.999883 |
| ENST00000511634 | INSC | 0.004277 | 0.995723 |
| ENST00000511634 | PEX14 | 0.005962 | 0.994038 |
| ENST00000511634 | XYLT2 | 0.006802 | 0.993198 |
| ENST00000511634 | ZNF713 | 0.005193 | 0.994807 |
| ENST00000511634 | MYH3 | 0.006584 | -0.99342 |
| ENST00000511634 | KCTD1 | 0.005208 | 0.994792 |
| ENST00000511634 | CATG00000047316.1 | 2.83E-04 | 0.999717 |
| ENST00000511634 | ZMYND15 | 0.002801 | 0.997199 |
| ENST00000511634 | IQCF5 | 0.009317 | 0.990683 |
| ENST00000511634 | PAN3 | 0.008669 | -0.99133 |
| ENST00000511634 | DNAL4 | 0.001595 | -0.99841 |
| ENST00000511634 | ZNF587B | 0.006736 | -0.99326 |
| ENST00000511634 | INKA2 | 0.002723 | 0.997277 |
| ENST00000511634 | ZNF37A | 3.27E-04 | -0.99967 |
| ENST00000511634 | WAPL | 0.002791 | -0.99721 |
| ENST00000511634 | DICER1 | 0.001006 | -0.99899 |
| ENST00000438293 | NFATC2 | 0.003317 | 0.996683 |
| ENST00000438293 | MINK1 | 0.006326 | -0.99367 |
| ENST00000438293 | CFAP77 | 0.006634 | 0.993366 |
| ENST00000438293 | ST3GAL2 | 0.008065 | -0.99193 |
| ENST00000438293 | ACER1 | 0.00103 | -0.99897 |
| ENST00000438293 | CATG00000096017.1 | 0.008646 | 0.991354 |
| ENST00000359720 | PTPN6 | 0.003714 | 0.996286 |
| ENST00000359720 | DNAJA4 | 0.006687 | -0.99331 |
| ENST00000359720 | PXDC1 | 0.006977 | -0.99302 |
| ENST00000359720 | NOL9 | 0.008831 | 0.991169 |
| ENST00000359720 | EVC2 | 0.009012 | -0.99099 |
| ENST00000359720 | SHC2 | 0.006154 | -0.99385 |
| ENST00000359720 | MYCN | 0.009333 | -0.99067 |
| ENST00000359720 | SPSB2 | 0.005685 | -0.99432 |
| ENST00000359720 | PPP1R3E | 0.005755 | 0.994245 |
| ENST00000464144 | PHF20 | 0.006968 | -0.99303 |
| ENST00000464144 | CLEC17A | 0.00544 | 0.99456 |
| ENST00000464144 | CDO1 | 0.00326 | -0.99674 |
| ENST00000464144 | SELENOP | 0.004207 | -0.99579 |
| ENST00000464144 | PROKR1 | 0.006416 | -0.99358 |
| ENST00000464144 | DEFB112 | 0.002121 | -0.99788 |
| ENST00000464144 | GPR153 | 3.22E-04 | 0.999678 |
| ENST00000464144 | VNN3 | 0.001924 | 0.998076 |
| ENST00000464144 | PHF20 | 0.006968 | -0.99303 |
| ENST00000464144 | CLEC17A | 0.00544 | 0.99456 |
| ENST00000464144 | CDO1 | 0.00326 | -0.99674 |
| ENST00000464144 | SELENOP | 0.004207 | -0.99579 |
| ENST00000464144 | PROKR1 | 0.006416 | -0.99358 |
| ENST00000464144 | DEFB112 | 0.002121 | -0.99788 |
| ENST00000464144 | GPR153 | 3.22E-04 | 0.999678 |
| ENST00000464144 | VNN3 | 0.001924 | 0.998076 |
| ENST00000464144 | PHF20 | 0.006968 | -0.99303 |
| ENST00000464144 | CLEC17A | 0.00544 | 0.99456 |
| ENST00000464144 | CDO1 | 0.00326 | -0.99674 |
| ENST00000464144 | SELENOP | 0.004207 | -0.99579 |
| ENST00000464144 | PROKR1 | 0.006416 | -0.99358 |
| ENST00000464144 | DEFB112 | 0.002121 | -0.99788 |
| ENST00000464144 | GPR153 | 3.22E-04 | 0.999678 |
| ENST00000464144 | VNN3 | 0.001924 | 0.998076 |
| ENST00000464144 | PHF20 | 0.006968 | -0.99303 |
| ENST00000464144 | CLEC17A | 0.00544 | 0.99456 |
| ENST00000464144 | CDO1 | 0.00326 | -0.99674 |
| ENST00000464144 | SELENOP | 0.004207 | -0.99579 |
| ENST00000464144 | PROKR1 | 0.006416 | -0.99358 |
| ENST00000464144 | DEFB112 | 0.002121 | -0.99788 |
| ENST00000464144 | GPR153 | 3.22E-04 | 0.999678 |
| ENST00000464144 | VNN3 | 0.001924 | 0.998076 |
| ENST00000464144 | PHF20 | 0.006968 | -0.99303 |
| ENST00000464144 | CLEC17A | 0.00544 | 0.99456 |
| ENST00000464144 | CDO1 | 0.00326 | -0.99674 |
| ENST00000464144 | SELENOP | 0.004207 | -0.99579 |
| ENST00000464144 | PROKR1 | 0.006416 | -0.99358 |
| ENST00000464144 | DEFB112 | 0.002121 | -0.99788 |
| ENST00000464144 | GPR153 | 3.22E-04 | 0.999678 |
| ENST00000464144 | VNN3 | 0.001924 | 0.998076 |
| ENST00000464144 | PHF20 | 0.006968 | -0.99303 |
| ENST00000464144 | CLEC17A | 0.00544 | 0.99456 |
| ENST00000464144 | CDO1 | 0.00326 | -0.99674 |
| ENST00000464144 | SELENOP | 0.004207 | -0.99579 |
| ENST00000464144 | PROKR1 | 0.006416 | -0.99358 |
| ENST00000464144 | DEFB112 | 0.002121 | -0.99788 |
| ENST00000464144 | GPR153 | 3.22E-04 | 0.999678 |
| ENST00000464144 | VNN3 | 0.001924 | 0.998076 |
| ENST00000464144 | PHF20 | 0.006968 | -0.99303 |
| ENST00000464144 | CLEC17A | 0.00544 | 0.99456 |
| ENST00000464144 | CDO1 | 0.00326 | -0.99674 |
| ENST00000464144 | SELENOP | 0.004207 | -0.99579 |
| ENST00000464144 | PROKR1 | 0.006416 | -0.99358 |
| ENST00000464144 | DEFB112 | 0.002121 | -0.99788 |
| ENST00000464144 | GPR153 | 3.22E-04 | 0.999678 |
| ENST00000464144 | VNN3 | 0.001924 | 0.998076 |
