# Supplementary material for: M6A Modification and Transcription Analysis of LncRNA in Cerebral Ischemia/Reperfusion Injury
Source: Int J Genomics. 2024 Oct 5;2024:4596974. doi: 10.1155/2024/4596974 (PMC11470819; doi:10.1155/2024/4596974)
Supplement: Supporting Information — Additional supporting information can be found online in the Supporting Information section. Table S1. Pearson correlation analysis between upregulated LncRNA and mRNA. Table S2. Pearson correlation analysis between downregulated LncRNA and mRNA. Table S3. Pearson correlation analysis between hypermethylated LncRNA and mRNA. Table S4. Pearson correlation analysis between hypomethylated LncRNA and mRNA. [file 4596974.f1.zip › Table S2.docx]

**Table S2 Pearson correlation analysis between down-regulated LncRNA and mRNA**

| LncRNA | mRNA | P | R |
| --- | --- | --- | --- |
| ENST00000625027 | CATG00000056264.1 | 0.007181 | -0.99282 |
| ENST00000625027 | CATG00000003494.1 | 0.005266 | -0.99473 |
| ENST00000625027 | CLEC4G | 0.00313 | -0.99687 |
| ENST00000625027 | CD27 | 0.001884 | -0.99812 |
| ENST00000625027 | TMC2 | 0.007058 | -0.99294 |
| ENST00000625027 | FAM90A1 | 0.002663 | 0.997337 |
| ENST00000625027 | TPRX1 | 0.002804 | -0.9972 |
| ENST00000625027 | CLCNKB | 3.10E-04 | -0.99969 |
| ENST00000625027 | DCD | 0.008876 | -0.99112 |
| ENST00000625027 | ELF2 | 0.003622 | 0.996378 |
| ENST00000625027 | INO80B | 0.005585 | -0.99442 |
| ENST00000625027 | LCE2A | 0.007389 | -0.99261 |
| ENST00000625027 | ACP6 | 0.00412 | 0.99588 |
| ENST00000625027 | PPTC7 | 0.002301 | 0.997699 |
| ENST00000625027 | GABPA | 0.006419 | -0.99358 |
| ENST00000625027 | CIB1 | 0.004087 | 0.995913 |
| ENST00000625027 | CATG00000056264.1 | 0.007181 | -0.99282 |
| ENST00000625027 | CATG00000003494.1 | 0.005266 | -0.99473 |
| ENST00000625027 | CLEC4G | 0.00313 | -0.99687 |
| ENST00000625027 | CD27 | 0.001884 | -0.99812 |
| ENST00000625027 | TMC2 | 0.007058 | -0.99294 |
| ENST00000625027 | FAM90A1 | 0.002663 | 0.997337 |
| ENST00000625027 | TPRX1 | 0.002804 | -0.9972 |
| ENST00000625027 | CLCNKB | 3.10E-04 | -0.99969 |
| ENST00000625027 | DCD | 0.008876 | -0.99112 |
| ENST00000625027 | ELF2 | 0.003622 | 0.996378 |
| ENST00000625027 | INO80B | 0.005585 | -0.99442 |
| ENST00000625027 | LCE2A | 0.007389 | -0.99261 |
| ENST00000625027 | ACP6 | 0.00412 | 0.99588 |
| ENST00000625027 | PPTC7 | 0.002301 | 0.997699 |
| ENST00000625027 | GABPA | 0.006419 | -0.99358 |
| ENST00000625027 | CIB1 | 0.004087 | 0.995913 |
| ENST00000625027 | CATG00000056264.1 | 0.007181 | -0.99282 |
| ENST00000625027 | CATG00000003494.1 | 0.005266 | -0.99473 |
| ENST00000625027 | CLEC4G | 0.00313 | -0.99687 |
| ENST00000625027 | CD27 | 0.001884 | -0.99812 |
| ENST00000625027 | TMC2 | 0.007058 | -0.99294 |
| ENST00000625027 | FAM90A1 | 0.002663 | 0.997337 |
| ENST00000625027 | TPRX1 | 0.002804 | -0.9972 |
| ENST00000625027 | CLCNKB | 3.10E-04 | -0.99969 |
| ENST00000625027 | DCD | 0.008876 | -0.99112 |
| ENST00000625027 | ELF2 | 0.003622 | 0.996378 |
| ENST00000625027 | INO80B | 0.005585 | -0.99442 |
| ENST00000625027 | LCE2A | 0.007389 | -0.99261 |
| ENST00000625027 | ACP6 | 0.00412 | 0.99588 |
| ENST00000625027 | PPTC7 | 0.002301 | 0.997699 |
| ENST00000625027 | GABPA | 0.006419 | -0.99358 |
| ENST00000625027 | CIB1 | 0.004087 | 0.995913 |
| ENST00000625027 | CATG00000056264.1 | 0.007181 | -0.99282 |
| ENST00000625027 | CATG00000003494.1 | 0.005266 | -0.99473 |
| ENST00000625027 | CLEC4G | 0.00313 | -0.99687 |
| ENST00000625027 | CD27 | 0.001884 | -0.99812 |
| ENST00000625027 | TMC2 | 0.007058 | -0.99294 |
| ENST00000625027 | FAM90A1 | 0.002663 | 0.997337 |
| ENST00000625027 | TPRX1 | 0.002804 | -0.9972 |
| ENST00000625027 | CLCNKB | 3.10E-04 | -0.99969 |
| ENST00000625027 | DCD | 0.008876 | -0.99112 |
| ENST00000625027 | ELF2 | 0.003622 | 0.996378 |
| ENST00000625027 | INO80B | 0.005585 | -0.99442 |
| ENST00000625027 | LCE2A | 0.007389 | -0.99261 |
| ENST00000625027 | ACP6 | 0.00412 | 0.99588 |
| ENST00000625027 | PPTC7 | 0.002301 | 0.997699 |
| ENST00000625027 | GABPA | 0.006419 | -0.99358 |
| ENST00000625027 | CIB1 | 0.004087 | 0.995913 |
| ENST00000625027 | CATG00000056264.1 | 0.007181 | -0.99282 |
| ENST00000625027 | CATG00000003494.1 | 0.005266 | -0.99473 |
| ENST00000625027 | CLEC4G | 0.00313 | -0.99687 |
| ENST00000625027 | CD27 | 0.001884 | -0.99812 |
| ENST00000625027 | TMC2 | 0.007058 | -0.99294 |
| ENST00000625027 | FAM90A1 | 0.002663 | 0.997337 |
| ENST00000625027 | TPRX1 | 0.002804 | -0.9972 |
| ENST00000625027 | CLCNKB | 3.10E-04 | -0.99969 |
| ENST00000625027 | DCD | 0.008876 | -0.99112 |
| ENST00000625027 | ELF2 | 0.003622 | 0.996378 |
| ENST00000625027 | INO80B | 0.005585 | -0.99442 |
| ENST00000625027 | LCE2A | 0.007389 | -0.99261 |
| ENST00000625027 | ACP6 | 0.00412 | 0.99588 |
| ENST00000625027 | PPTC7 | 0.002301 | 0.997699 |
| ENST00000625027 | GABPA | 0.006419 | -0.99358 |
| ENST00000625027 | CIB1 | 0.004087 | 0.995913 |
| ENST00000625027 | CATG00000056264.1 | 0.007181 | -0.99282 |
| ENST00000625027 | CATG00000003494.1 | 0.005266 | -0.99473 |
| ENST00000625027 | CLEC4G | 0.00313 | -0.99687 |
| ENST00000625027 | CD27 | 0.001884 | -0.99812 |
| ENST00000625027 | TMC2 | 0.007058 | -0.99294 |
| ENST00000625027 | FAM90A1 | 0.002663 | 0.997337 |
| ENST00000625027 | TPRX1 | 0.002804 | -0.9972 |
| ENST00000625027 | CLCNKB | 3.10E-04 | -0.99969 |
| ENST00000625027 | DCD | 0.008876 | -0.99112 |
| ENST00000625027 | ELF2 | 0.003622 | 0.996378 |
| ENST00000625027 | INO80B | 0.005585 | -0.99442 |
| ENST00000625027 | LCE2A | 0.007389 | -0.99261 |
| ENST00000625027 | ACP6 | 0.00412 | 0.99588 |
| ENST00000625027 | PPTC7 | 0.002301 | 0.997699 |
| ENST00000625027 | GABPA | 0.006419 | -0.99358 |
| ENST00000625027 | CIB1 | 0.004087 | 0.995913 |
| ENST00000625027 | CATG00000056264.1 | 0.007181 | -0.99282 |
| ENST00000625027 | CATG00000003494.1 | 0.005266 | -0.99473 |
| ENST00000625027 | CLEC4G | 0.00313 | -0.99687 |
| ENST00000625027 | CD27 | 0.001884 | -0.99812 |
| ENST00000625027 | TMC2 | 0.007058 | -0.99294 |
| ENST00000625027 | FAM90A1 | 0.002663 | 0.997337 |
| ENST00000625027 | TPRX1 | 0.002804 | -0.9972 |
| ENST00000625027 | CLCNKB | 3.10E-04 | -0.99969 |
| ENST00000625027 | DCD | 0.008876 | -0.99112 |
| ENST00000625027 | ELF2 | 0.003622 | 0.996378 |
| ENST00000625027 | INO80B | 0.005585 | -0.99442 |
| ENST00000625027 | LCE2A | 0.007389 | -0.99261 |
| ENST00000625027 | ACP6 | 0.00412 | 0.99588 |
| ENST00000625027 | PPTC7 | 0.002301 | 0.997699 |
| ENST00000625027 | GABPA | 0.006419 | -0.99358 |
| ENST00000625027 | CIB1 | 0.004087 | 0.995913 |
| ENST00000625027 | CATG00000056264.1 | 0.007181 | -0.99282 |
| ENST00000625027 | CATG00000003494.1 | 0.005266 | -0.99473 |
| ENST00000625027 | CLEC4G | 0.00313 | -0.99687 |
| ENST00000625027 | CD27 | 0.001884 | -0.99812 |
| ENST00000625027 | TMC2 | 0.007058 | -0.99294 |
| ENST00000625027 | FAM90A1 | 0.002663 | 0.997337 |
| ENST00000625027 | TPRX1 | 0.002804 | -0.9972 |
| ENST00000625027 | CLCNKB | 3.10E-04 | -0.99969 |
| ENST00000625027 | DCD | 0.008876 | -0.99112 |
| ENST00000625027 | ELF2 | 0.003622 | 0.996378 |
| ENST00000625027 | INO80B | 0.005585 | -0.99442 |
| ENST00000625027 | LCE2A | 0.007389 | -0.99261 |
| ENST00000625027 | ACP6 | 0.00412 | 0.99588 |
| ENST00000625027 | PPTC7 | 0.002301 | 0.997699 |
| ENST00000625027 | GABPA | 0.006419 | -0.99358 |
| ENST00000625027 | CIB1 | 0.004087 | 0.995913 |
| ENST00000444219 | RNF207 | 0.005753 | -0.99425 |
| ENST00000444219 | PLEKHA2 | 0.002861 | 0.997139 |
| ENST00000444219 | PLAG1 | 0.009444 | 0.990556 |
| ENST00000444219 | DYNC2H1 | 0.003538 | -0.99646 |
| T371632 | MMP1 | 0.006137 | -0.99386 |
| T371632 | TTC38 | 0.003174 | -0.99683 |
| T371632 | IGFL2 | 0.009307 | -0.99069 |
| ENST00000505329 | BECN1 | 0.002963 | -0.99704 |
| ENST00000505329 | ACTL7B | 6.23E-04 | 0.999377 |
| ENST00000505329 | C15orf65 | 0.007953 | 0.992047 |
| ENST00000505329 | NDUFAF8 | 0.001524 | -0.99848 |
| ENST00000505329 | MED12L | 0.001893 | 0.998107 |
| ENST00000505329 | PMEPA1 | 0.005357 | 0.994643 |
| ENST00000505329 | FCRL1 | 0.009119 | 0.990881 |
| ENST00000591503 | NOC4L | 0.006501 | 0.993499 |
| ENST00000591503 | CATG00000080699.1 | 0.002301 | 0.997699 |
| ENST00000591503 | SLC24A5 | 0.009133 | -0.99087 |
| ENST00000591503 | TCOF1 | 0.004763 | 0.995237 |
| ENST00000591503 | SAMD13 | 0.005253 | 0.994747 |
| ENST00000591503 | ZDHHC16 | 2.84E-04 | -0.99972 |
| ENST00000591503 | CDKL2 | 0.008566 | 0.991434 |
| ENST00000591503 | MACROD2 | 0.001153 | 0.998847 |
| ENST00000591503 | CHST12 | 0 | 1 |
| ENST00000591503 | HIPK2 | 0.004794 | -0.99521 |
| ENST00000591503 | PCDHB8 | 0 | 1 |
| ENST00000443714 | SZRD1 | 0.009159 | -0.99084 |
| ENST00000443714 | FAM84A | 2.72E-04 | -0.99973 |
| ENST00000443714 | C20orf203 | 0.00568 | -0.99432 |
| ENST00000443714 | DUSP15 | 0.006871 | -0.99313 |
| ENST00000443714 | GMPPA | 0.008192 | -0.99181 |
| ENST00000443714 | AASDHPPT | 0.004376 | -0.99562 |
| ENST00000443714 | G6PC3 | 9.21E-05 | 0.999908 |
| ENST00000443714 | RTN4 | 0.008958 | 0.991042 |
| ENST00000443714 | FAM110C | 0.005153 | 0.994847 |
| ENST00000443714 | C16orf78 | 0.006839 | 0.993161 |
| ENST00000443714 | OR5D18 | 0.004406 | 0.995594 |
| T109219 | SUPV3L1 | 0.003812 | -0.99619 |
| T109219 | GPR156 | 0.008887 | 0.991113 |
| T109219 | UBE2V1 | 0.001289 | -0.99871 |
| T109219 | FTCD | 0.009392 | 0.990608 |
| T109219 | VGLL3 | 0.006226 | 0.993774 |
| T109219 | FAM186A | 0.00314 | 0.99686 |
| T109219 | PGAM5 | 0.005833 | 0.994167 |
| T109219 | SUPV3L1 | 0.003812 | -0.99619 |
| T109219 | GPR156 | 0.008887 | 0.991113 |
| T109219 | UBE2V1 | 0.001289 | -0.99871 |
| T109219 | FTCD | 0.009392 | 0.990608 |
| T109219 | VGLL3 | 0.006226 | 0.993774 |
| T109219 | FAM186A | 0.00314 | 0.99686 |
| T109219 | PGAM5 | 0.005833 | 0.994167 |
| T109219 | SUPV3L1 | 0.003812 | -0.99619 |
| T109219 | GPR156 | 0.008887 | 0.991113 |
| T109219 | UBE2V1 | 0.001289 | -0.99871 |
| T109219 | FTCD | 0.009392 | 0.990608 |
| T109219 | VGLL3 | 0.006226 | 0.993774 |
| T109219 | FAM186A | 0.00314 | 0.99686 |
| T109219 | PGAM5 | 0.005833 | 0.994167 |
| T109219 | SUPV3L1 | 0.003812 | -0.99619 |
| T109219 | GPR156 | 0.008887 | 0.991113 |
| T109219 | UBE2V1 | 0.001289 | -0.99871 |
| T109219 | FTCD | 0.009392 | 0.990608 |
| T109219 | VGLL3 | 0.006226 | 0.993774 |
| T109219 | FAM186A | 0.00314 | 0.99686 |
| T109219 | PGAM5 | 0.005833 | 0.994167 |
| T094824 | RPUSD1 | 0.008662 | -0.99134 |
| T094824 | AIFM3 | 0.007007 | -0.99299 |
| T094824 | ALB | 0.007957 | 0.992043 |
| T094824 | HILPDA | 0.006482 | 0.993518 |
| T094824 | RPUSD1 | 0.008662 | -0.99134 |
| T094824 | AIFM3 | 0.007007 | -0.99299 |
| T094824 | ALB | 0.007957 | 0.992043 |
| T094824 | HILPDA | 0.006482 | 0.993518 |
| T094824 | RPUSD1 | 0.008662 | -0.99134 |
| T094824 | AIFM3 | 0.007007 | -0.99299 |
| T094824 | ALB | 0.007957 | 0.992043 |
| T094824 | HILPDA | 0.006482 | 0.993518 |
| T094824 | RPUSD1 | 0.008662 | -0.99134 |
| T094824 | AIFM3 | 0.007007 | -0.99299 |
| T094824 | ALB | 0.007957 | 0.992043 |
| T094824 | HILPDA | 0.006482 | 0.993518 |
| ENST00000512527 | TOB1 | 0.001082 | -0.99892 |
| ENST00000512527 | MED20 | 0.002767 | 0.997233 |
| ENST00000512527 | PPARD | 0.003709 | 0.996291 |
| ENST00000512527 | PRKDC | 0.00661 | -0.99339 |
| ENST00000512527 | METTL3 | 0.00736 | -0.99264 |
| ENST00000512527 | ACO1 | 0.00505 | 0.99495 |
| ENST00000512527 | TSFM | 0.009711 | -0.99029 |
| ENST00000512527 | USF2 | 0.009104 | -0.9909 |
| T249007 | EXOC3L2 | 0.007888 | 0.992112 |
| T249007 | POP1 | 0.009939 | -0.99006 |
| T249007 | PLD3 | 0.001255 | -0.99874 |
| T249007 | RAB8A | 0.001266 | -0.99873 |
| T249007 | EEF1B2 | 0.006808 | 0.993192 |
| T249007 | SSX1 | 0.007746 | 0.992254 |
| T249007 | WSCD2 | 8.91E-04 | 0.999109 |
| T249007 | GRIN3B | 0.003121 | -0.99688 |
| T249007 | TNFAIP6 | 0.004043 | -0.99596 |
| T249007 | PTPRK | 0.001414 | 0.998586 |
| T249007 | CATG00000039609.1 | 0.004336 | 0.995664 |
| ENST00000520562 | HEXIM2 | 0.004933 | 0.995067 |
| ENST00000520562 | OCLN | 9.73E-04 | 0.999027 |
| ENST00000520562 | FAR1 | 6.12E-05 | 0.999939 |
| ENST00000520562 | TRIM73 | 0.009492 | 0.990508 |
| ENST00000520562 | KIF27 | 0.00232 | 0.99768 |
| ENST00000520562 | HEXIM2 | 0.004933 | 0.995067 |
| ENST00000520562 | OCLN | 9.73E-04 | 0.999027 |
| ENST00000520562 | FAR1 | 6.12E-05 | 0.999939 |
| ENST00000520562 | TRIM73 | 0.009492 | 0.990508 |
| ENST00000520562 | KIF27 | 0.00232 | 0.99768 |
| ENST00000520562 | HEXIM2 | 0.004933 | 0.995067 |
| ENST00000520562 | OCLN | 9.73E-04 | 0.999027 |
| ENST00000520562 | FAR1 | 6.12E-05 | 0.999939 |
| ENST00000520562 | TRIM73 | 0.009492 | 0.990508 |
| ENST00000520562 | KIF27 | 0.00232 | 0.99768 |
| ENST00000520562 | HEXIM2 | 0.004933 | 0.995067 |
| ENST00000520562 | OCLN | 9.73E-04 | 0.999027 |
| ENST00000520562 | FAR1 | 6.12E-05 | 0.999939 |
| ENST00000520562 | TRIM73 | 0.009492 | 0.990508 |
| ENST00000520562 | KIF27 | 0.00232 | 0.99768 |
| ENST00000520562 | HEXIM2 | 0.004933 | 0.995067 |
| ENST00000520562 | OCLN | 9.73E-04 | 0.999027 |
| ENST00000520562 | FAR1 | 6.12E-05 | 0.999939 |
| ENST00000520562 | TRIM73 | 0.009492 | 0.990508 |
| ENST00000520562 | KIF27 | 0.00232 | 0.99768 |
| ENST00000520562 | HEXIM2 | 0.004933 | 0.995067 |
| ENST00000520562 | OCLN | 9.73E-04 | 0.999027 |
| ENST00000520562 | FAR1 | 6.12E-05 | 0.999939 |
| ENST00000520562 | TRIM73 | 0.009492 | 0.990508 |
| ENST00000520562 | KIF27 | 0.00232 | 0.99768 |
| ENST00000520562 | HEXIM2 | 0.004933 | 0.995067 |
| ENST00000520562 | OCLN | 9.73E-04 | 0.999027 |
| ENST00000520562 | FAR1 | 6.12E-05 | 0.999939 |
| ENST00000520562 | TRIM73 | 0.009492 | 0.990508 |
| ENST00000520562 | KIF27 | 0.00232 | 0.99768 |
| ENST00000520562 | HEXIM2 | 0.004933 | 0.995067 |
| ENST00000520562 | OCLN | 9.73E-04 | 0.999027 |
| ENST00000520562 | FAR1 | 6.12E-05 | 0.999939 |
| ENST00000520562 | TRIM73 | 0.009492 | 0.990508 |
| ENST00000520562 | KIF27 | 0.00232 | 0.99768 |
| ENST00000520562 | HEXIM2 | 0.004933 | 0.995067 |
| ENST00000520562 | OCLN | 9.73E-04 | 0.999027 |
| ENST00000520562 | FAR1 | 6.12E-05 | 0.999939 |
| ENST00000520562 | TRIM73 | 0.009492 | 0.990508 |
| ENST00000520562 | KIF27 | 0.00232 | 0.99768 |
| ENST00000542819 | SPTY2D1OS | 0.004598 | 0.995402 |
| ENST00000542819 | LYL1 | 0.009359 | 0.990641 |
| ENST00000542819 | STEAP3 | 0.001736 | 0.998264 |
| ENST00000542819 | SLC13A4 | 0.007005 | 0.992995 |
| ENST00000542819 | TFF2 | 0.005026 | 0.994974 |
| ENST00000542819 | ORC3 | 0.004781 | 0.995219 |
| ENST00000542819 | UGT2B28 | 4.53E-04 | 0.999547 |
| ENST00000466168 | ACTR3B | 0.006986 | 0.993014 |
| ENST00000466168 | TTC38 | 0.001555 | -0.99845 |
| ENST00000466168 | PLAG1 | 0.00833 | -0.99167 |
| ENST00000466168 | ACTR3B | 0.006986 | 0.993014 |
| ENST00000466168 | TTC38 | 0.001555 | -0.99845 |
| ENST00000466168 | PLAG1 | 0.00833 | -0.99167 |
| ENST00000612739 | ACTL7B | 0.005474 | 0.994526 |
| ENST00000612739 | FBXL16 | 0.003773 | -0.99623 |
| ENST00000612739 | MED12L | 0.001322 | 0.998678 |
| ENST00000554409 | RPS15 | 0.007265 | -0.99273 |
| ENST00000554409 | ARHGAP8 | 7.55E-04 | -0.99925 |
| ENST00000554409 | PLA2G15 | 0.003931 | -0.99607 |
| ENST00000554409 | JCAD | 0.003353 | 0.996647 |
| ENST00000554409 | TREM1 | 0.005225 | 0.994775 |
| ENST00000554409 | CTNNBIP1 | 0.007054 | -0.99295 |
| ENST00000584677 | BECN1 | 0.008696 | -0.9913 |
| ENST00000584677 | ACTL7B | 0.003115 | 0.996885 |
| ENST00000584677 | NDUFAF8 | 0.009541 | -0.99046 |
| ENST00000584677 | FBXL16 | 0.005074 | -0.99493 |
| ENST00000584677 | MED12L | 2.86E-04 | 0.999714 |
| T018050 | AHNAK2 | 0.002865 | 0.997135 |
| T018050 | CHSY3 | 0.008572 | 0.991428 |
| T018050 | HOMER3 | 0.003101 | -0.9969 |
| T018050 | ARPC4-TTLL3 | 0.005948 | -0.99405 |
| T018050 | CATG00000113928.1 | 0.006115 | -0.99389 |
| T018050 | MMP24 | 0.005311 | -0.99469 |
| T018050 | ZNF624 | 0.004769 | 0.995231 |
| MICT00000282226 | RRAS2 | 6.58E-04 | 0.999342 |
| MICT00000282226 | ZP1 | 0.007687 | 0.992313 |
| T151854 | IL31RA | 0.008088 | -0.99191 |
| T151854 | EHD4 | 0.006354 | 0.993646 |
| T151854 | SHISA6 | 0.006778 | 0.993222 |
| T151854 | CAMP | 0.00985 | 0.99015 |
| T151854 | SMAP2 | 0.005185 | 0.994815 |
| T151854 | ISG15 | 0.004151 | -0.99585 |
| T212465 | FILIP1 | 0.005084 | -0.99492 |
| T212465 | CLEC1A | 0.008936 | -0.99106 |
| T212465 | HMGXB4 | 6.27E-04 | -0.99937 |
| T212465 | RPL11 | 0.00102 | -0.99898 |
| T212465 | PMPCA | 0.003341 | 0.996659 |
| T212465 | UNC45A | 0.004108 | 0.995892 |
| T212465 | CAPNS1 | 0.002641 | -0.99736 |
| T212465 | TUBGCP4 | 0.008722 | 0.991278 |
| T212465 | RACK1 | 0.005172 | 0.994828 |
| T212465 | KRTAP4-7 | 0.004643 | 0.995357 |
| T212465 | CDKN3 | 2.12E-04 | -0.99979 |
| T212465 | RPUSD2 | 0.004596 | -0.9954 |
| T212465 | USP9X | 0.003088 | 0.996912 |
| T212465 | ZNF431 | 0.008871 | 0.991129 |
| T212465 | FILIP1 | 0.005084 | -0.99492 |
| T212465 | CLEC1A | 0.008936 | -0.99106 |
| T212465 | HMGXB4 | 6.27E-04 | -0.99937 |
| T212465 | RPL11 | 0.00102 | -0.99898 |
| T212465 | PMPCA | 0.003341 | 0.996659 |
| T212465 | UNC45A | 0.004108 | 0.995892 |
| T212465 | CAPNS1 | 0.002641 | -0.99736 |
| T212465 | TUBGCP4 | 0.008722 | 0.991278 |
| T212465 | RACK1 | 0.005172 | 0.994828 |
| T212465 | KRTAP4-7 | 0.004643 | 0.995357 |
| T212465 | CDKN3 | 2.12E-04 | -0.99979 |
| T212465 | RPUSD2 | 0.004596 | -0.9954 |
| T212465 | USP9X | 0.003088 | 0.996912 |
| T212465 | ZNF431 | 0.008871 | 0.991129 |
| T212465 | FILIP1 | 0.005084 | -0.99492 |
| T212465 | CLEC1A | 0.008936 | -0.99106 |
| T212465 | HMGXB4 | 6.27E-04 | -0.99937 |
| T212465 | RPL11 | 0.00102 | -0.99898 |
| T212465 | PMPCA | 0.003341 | 0.996659 |
| T212465 | UNC45A | 0.004108 | 0.995892 |
| T212465 | CAPNS1 | 0.002641 | -0.99736 |
| T212465 | TUBGCP4 | 0.008722 | 0.991278 |
| T212465 | RACK1 | 0.005172 | 0.994828 |
| T212465 | KRTAP4-7 | 0.004643 | 0.995357 |
| T212465 | CDKN3 | 2.12E-04 | -0.99979 |
| T212465 | RPUSD2 | 0.004596 | -0.9954 |
| T212465 | USP9X | 0.003088 | 0.996912 |
| T212465 | ZNF431 | 0.008871 | 0.991129 |
| TCONS_00001047 | PDE4C | 0.009302 | -0.9907 |
| TCONS_00001047 | CATG00000029374.1 | 0.009406 | 0.990594 |
| ENST00000556520 | CATG00000038628.1 | 0.008461 | -0.99154 |
| ENST00000556520 | OR5T2 | 0.003827 | 0.996173 |
| ENST00000556520 | CATG00000038628.1 | 0.008461 | -0.99154 |
| ENST00000556520 | OR5T2 | 0.003827 | 0.996173 |
| ENST00000556520 | CATG00000038628.1 | 0.008461 | -0.99154 |
| ENST00000556520 | OR5T2 | 0.003827 | 0.996173 |
| ENST00000556520 | CATG00000038628.1 | 0.008461 | -0.99154 |
| ENST00000556520 | OR5T2 | 0.003827 | 0.996173 |
| ENST00000604999 | AHNAK2 | 0.003686 | 0.996314 |
| ENST00000604999 | CHSY3 | 0.00798 | 0.99202 |
| ENST00000604999 | HOMER3 | 0.003969 | -0.99603 |
| ENST00000604999 | ARPC4-TTLL3 | 0.008289 | -0.99171 |
| ENST00000604999 | CATG00000113928.1 | 0.006437 | -0.99356 |
| ENST00000604999 | MMP24 | 0.005503 | -0.9945 |
| ENST00000604999 | ZNF624 | 0.005809 | 0.994191 |
| ENST00000513419 | RRAS2 | 0.004097 | 0.995903 |
| ENST00000513419 | CATG00000067666.1 | 0.007299 | 0.992701 |
| ENST00000513419 | ZP1 | 0.004279 | 0.995721 |
| T367347 | LGALS1 | 0.005974 | 0.994026 |
| NR_033370 | DNAJC6 | 0.003412 | -0.99659 |
| NR_033370 | MFAP3L | 0.008413 | -0.99159 |
| NR_033370 | LGALS1 | 0.00286 | 0.99714 |
| NR_033370 | SPACA9 | 0.002087 | -0.99791 |
| NR_033370 | OR5T2 | 0.009715 | 0.990285 |
| GSE61474_TCONS_00271756 | RPUSD1 | 9.91E-04 | -0.99901 |
| GSE61474_TCONS_00271756 | ALB | 0.001709 | 0.998291 |
| GSE61474_TCONS_00271756 | TNFSF13B | 0.002402 | -0.9976 |
| GSE61474_TCONS_00271756 | AGAP1 | 0.002651 | 0.997349 |
| GSE61474_TCONS_00271756 | CATG00000051841.1 | 0.0091 | 0.9909 |
| GSE61474_TCONS_00271756 | HILPDA | 0.002646 | 0.997354 |
| T293890 | DNASE1L2 | 0.007108 | -0.99289 |
| T293890 | TUBB2A | 0.00406 | 0.99594 |
| T293890 | EGF | 0.004676 | -0.99532 |
| T293890 | CATG00000099282.1 | 0.004465 | 0.995535 |
| ENST00000431144 | ZFAND5 | 0.008863 | -0.99114 |
| ENST00000431144 | CATG00000056264.1 | 0.006969 | -0.99303 |
| ENST00000431144 | SSBP2 | 0.007531 | -0.99247 |
| ENST00000431144 | CLEC4G | 0.006593 | -0.99341 |
| ENST00000431144 | CD27 | 0.008741 | -0.99126 |
| ENST00000431144 | TMC2 | 0.003645 | -0.99636 |
| ENST00000431144 | NAGLU | 0.002411 | -0.99759 |
| ENST00000431144 | CLK3 | 2.52E-04 | -0.99975 |
| ENST00000431144 | TPRX1 | 0.003188 | -0.99681 |
| ENST00000431144 | CLCNKB | 0.009189 | -0.99081 |
| ENST00000431144 | PPTC7 | 0.003609 | 0.996391 |
| ENST00000431144 | CIB1 | 0.001946 | 0.998054 |
| ENST00000423466 | PHF20 | 0.001496 | -0.9985 |
| ENST00000423466 | MMP1 | 0.008061 | -0.99194 |
| ENST00000423466 | CDO1 | 1.36E-04 | -0.99986 |
| ENST00000423466 | PARP6 | 0.00409 | 0.99591 |
| ENST00000423466 | ABCA4 | 0.009698 | 0.990302 |
| ENST00000423466 | DEFB112 | 0.001334 | -0.99867 |
| ENST00000423466 | GPR153 | 0.003163 | 0.996837 |
| ENST00000423466 | PMIS2 | 0.005156 | 0.994844 |
| ENST00000423466 | KCTD18 | 0.006693 | 0.993307 |
| ENST00000423466 | VNN3 | 0.005906 | 0.994094 |
| ENST00000437156 | CATG00000092654.1 | 0.003244 | -0.99676 |
| ENST00000437156 | APBB3 | 0.002952 | 0.997048 |
| ENST00000437156 | PLEKHD1 | 0.0045 | -0.9955 |
| ENST00000437156 | MLLT1 | 0.005047 | 0.994953 |
| ENST00000437156 | DNAH6 | 0.00133 | 0.99867 |
| ENST00000437156 | MAP7 | 0.006017 | 0.993983 |
| ENST00000449197 | BECN1 | 0.002733 | -0.99727 |
| ENST00000449197 | ZNF736 | 0.009579 | -0.99042 |
| ENST00000449197 | ACTL7B | 0.00128 | 0.99872 |
| ENST00000449197 | C15orf65 | 0.005137 | 0.994863 |
| ENST00000449197 | NTNG1 | 0.009321 | -0.99068 |
| ENST00000449197 | NDUFAF8 | 5.52E-04 | -0.99945 |
| ENST00000449197 | MED12L | 0.003823 | 0.996177 |
| ENST00000449197 | PMEPA1 | 0.003011 | 0.996989 |
| ENST00000449197 | FCRL1 | 0.005961 | 0.994039 |
| ENST00000449197 | BECN1 | 0.002733 | -0.99727 |
| ENST00000449197 | ZNF736 | 0.009579 | -0.99042 |
| ENST00000449197 | ACTL7B | 0.00128 | 0.99872 |
| ENST00000449197 | C15orf65 | 0.005137 | 0.994863 |
| ENST00000449197 | NTNG1 | 0.009321 | -0.99068 |
| ENST00000449197 | NDUFAF8 | 5.52E-04 | -0.99945 |
| ENST00000449197 | MED12L | 0.003823 | 0.996177 |
| ENST00000449197 | PMEPA1 | 0.003011 | 0.996989 |
| ENST00000449197 | FCRL1 | 0.005961 | 0.994039 |
| ENST00000449197 | BECN1 | 0.002733 | -0.99727 |
| ENST00000449197 | ZNF736 | 0.009579 | -0.99042 |
| ENST00000449197 | ACTL7B | 0.00128 | 0.99872 |
| ENST00000449197 | C15orf65 | 0.005137 | 0.994863 |
| ENST00000449197 | NTNG1 | 0.009321 | -0.99068 |
| ENST00000449197 | NDUFAF8 | 5.52E-04 | -0.99945 |
| ENST00000449197 | MED12L | 0.003823 | 0.996177 |
| ENST00000449197 | PMEPA1 | 0.003011 | 0.996989 |
| ENST00000449197 | FCRL1 | 0.005961 | 0.994039 |
| ENST00000449197 | BECN1 | 0.002733 | -0.99727 |
| ENST00000449197 | ZNF736 | 0.009579 | -0.99042 |
| ENST00000449197 | ACTL7B | 0.00128 | 0.99872 |
| ENST00000449197 | C15orf65 | 0.005137 | 0.994863 |
| ENST00000449197 | NTNG1 | 0.009321 | -0.99068 |
| ENST00000449197 | NDUFAF8 | 5.52E-04 | -0.99945 |
| ENST00000449197 | MED12L | 0.003823 | 0.996177 |
| ENST00000449197 | PMEPA1 | 0.003011 | 0.996989 |
| ENST00000449197 | FCRL1 | 0.005961 | 0.994039 |
| ENST00000449197 | BECN1 | 0.002733 | -0.99727 |
| ENST00000449197 | ZNF736 | 0.009579 | -0.99042 |
| ENST00000449197 | ACTL7B | 0.00128 | 0.99872 |
| ENST00000449197 | C15orf65 | 0.005137 | 0.994863 |
| ENST00000449197 | NTNG1 | 0.009321 | -0.99068 |
| ENST00000449197 | NDUFAF8 | 5.52E-04 | -0.99945 |
| ENST00000449197 | MED12L | 0.003823 | 0.996177 |
| ENST00000449197 | PMEPA1 | 0.003011 | 0.996989 |
| ENST00000449197 | FCRL1 | 0.005961 | 0.994039 |
| ENST00000590912 | AHNAK2 | 0.002704 | 0.997296 |
| ENST00000590912 | NCCRP1 | 0.007382 | 0.992618 |
| ENST00000590912 | DUSP15 | 0.006888 | 0.993112 |
| ENST00000590912 | HOMER3 | 0.002817 | -0.99718 |
| ENST00000590912 | AASDHPPT | 0.00962 | 0.99038 |
| ENST00000590912 | ARPC4-TTLL3 | 0.001841 | -0.99816 |
| ENST00000590912 | MAMLD1 | 0.006886 | -0.99311 |
| ENST00000590912 | CATG00000113928.1 | 0.007237 | -0.99276 |
| ENST00000590912 | ZNF214 | 0.007356 | -0.99264 |
| ENST00000590912 | MMP24 | 0.006771 | -0.99323 |
| ENST00000590912 | ZNF624 | 0.004034 | 0.995966 |
| ENST00000590912 | OR5D18 | 0.009116 | -0.99088 |
| ENST00000445861 | ZNF77 | 0.004258 | -0.99574 |
| ENST00000445861 | ZNF77 | 0.004258 | -0.99574 |
| NR_136304 | RHEB | 0.006618 | -0.99338 |
| NR_136304 | RIOX2 | 0.007203 | -0.9928 |
| NR_136304 | ASGR2 | 0.00919 | -0.99081 |
| NR_136304 | VWA5A | 0.003418 | -0.99658 |
| NR_136304 | NAT8 | 0.002285 | -0.99772 |
| NR_136304 | PLA1A | 0.001653 | 0.998347 |
| NR_136304 | TLR2 | 0.002339 | -0.99766 |
| NR_136304 | PLA2G4F | 0.008841 | -0.99116 |
| NR_136304 | RHEB | 0.006618 | -0.99338 |
| NR_136304 | RIOX2 | 0.007203 | -0.9928 |
| NR_136304 | ASGR2 | 0.00919 | -0.99081 |
| NR_136304 | VWA5A | 0.003418 | -0.99658 |
| NR_136304 | NAT8 | 0.002285 | -0.99772 |
| NR_136304 | PLA1A | 0.001653 | 0.998347 |
| NR_136304 | TLR2 | 0.002339 | -0.99766 |
| NR_136304 | PLA2G4F | 0.008841 | -0.99116 |
| NR_136304 | RHEB | 0.006618 | -0.99338 |
| NR_136304 | RIOX2 | 0.007203 | -0.9928 |
| NR_136304 | ASGR2 | 0.00919 | -0.99081 |
| NR_136304 | VWA5A | 0.003418 | -0.99658 |
| NR_136304 | NAT8 | 0.002285 | -0.99772 |
| NR_136304 | PLA1A | 0.001653 | 0.998347 |
| NR_136304 | TLR2 | 0.002339 | -0.99766 |
| NR_136304 | PLA2G4F | 0.008841 | -0.99116 |
| NR_136304 | RHEB | 0.006618 | -0.99338 |
| NR_136304 | RIOX2 | 0.007203 | -0.9928 |
| NR_136304 | ASGR2 | 0.00919 | -0.99081 |
| NR_136304 | VWA5A | 0.003418 | -0.99658 |
| NR_136304 | NAT8 | 0.002285 | -0.99772 |
| NR_136304 | PLA1A | 0.001653 | 0.998347 |
| NR_136304 | TLR2 | 0.002339 | -0.99766 |
| NR_136304 | PLA2G4F | 0.008841 | -0.99116 |
| NR_136304 | RHEB | 0.006618 | -0.99338 |
| NR_136304 | RIOX2 | 0.007203 | -0.9928 |
| NR_136304 | ASGR2 | 0.00919 | -0.99081 |
| NR_136304 | VWA5A | 0.003418 | -0.99658 |
| NR_136304 | NAT8 | 0.002285 | -0.99772 |
| NR_136304 | PLA1A | 0.001653 | 0.998347 |
| NR_136304 | TLR2 | 0.002339 | -0.99766 |
| NR_136304 | PLA2G4F | 0.008841 | -0.99116 |
| NR_136304 | RHEB | 0.006618 | -0.99338 |
| NR_136304 | RIOX2 | 0.007203 | -0.9928 |
| NR_136304 | ASGR2 | 0.00919 | -0.99081 |
| NR_136304 | VWA5A | 0.003418 | -0.99658 |
| NR_136304 | NAT8 | 0.002285 | -0.99772 |
| NR_136304 | PLA1A | 0.001653 | 0.998347 |
| NR_136304 | TLR2 | 0.002339 | -0.99766 |
| NR_136304 | PLA2G4F | 0.008841 | -0.99116 |
| NR_136304 | RHEB | 0.006618 | -0.99338 |
| NR_136304 | RIOX2 | 0.007203 | -0.9928 |
| NR_136304 | ASGR2 | 0.00919 | -0.99081 |
| NR_136304 | VWA5A | 0.003418 | -0.99658 |
| NR_136304 | NAT8 | 0.002285 | -0.99772 |
| NR_136304 | PLA1A | 0.001653 | 0.998347 |
| NR_136304 | TLR2 | 0.002339 | -0.99766 |
| NR_136304 | PLA2G4F | 0.008841 | -0.99116 |
| NR_136304 | RHEB | 0.006618 | -0.99338 |
| NR_136304 | RIOX2 | 0.007203 | -0.9928 |
| NR_136304 | ASGR2 | 0.00919 | -0.99081 |
| NR_136304 | VWA5A | 0.003418 | -0.99658 |
| NR_136304 | NAT8 | 0.002285 | -0.99772 |
| NR_136304 | PLA1A | 0.001653 | 0.998347 |
| NR_136304 | TLR2 | 0.002339 | -0.99766 |
| NR_136304 | PLA2G4F | 0.008841 | -0.99116 |
| NR_136304 | RHEB | 0.006618 | -0.99338 |
| NR_136304 | RIOX2 | 0.007203 | -0.9928 |
| NR_136304 | ASGR2 | 0.00919 | -0.99081 |
| NR_136304 | VWA5A | 0.003418 | -0.99658 |
| NR_136304 | NAT8 | 0.002285 | -0.99772 |
| NR_136304 | PLA1A | 0.001653 | 0.998347 |
| NR_136304 | TLR2 | 0.002339 | -0.99766 |
| NR_136304 | PLA2G4F | 0.008841 | -0.99116 |
| NR_136304 | RHEB | 0.006618 | -0.99338 |
| NR_136304 | RIOX2 | 0.007203 | -0.9928 |
| NR_136304 | ASGR2 | 0.00919 | -0.99081 |
| NR_136304 | VWA5A | 0.003418 | -0.99658 |
| NR_136304 | NAT8 | 0.002285 | -0.99772 |
| NR_136304 | PLA1A | 0.001653 | 0.998347 |
| NR_136304 | TLR2 | 0.002339 | -0.99766 |
| NR_136304 | PLA2G4F | 0.008841 | -0.99116 |
| NR_136304 | RHEB | 0.006618 | -0.99338 |
| NR_136304 | RIOX2 | 0.007203 | -0.9928 |
| NR_136304 | ASGR2 | 0.00919 | -0.99081 |
| NR_136304 | VWA5A | 0.003418 | -0.99658 |
| NR_136304 | NAT8 | 0.002285 | -0.99772 |
| NR_136304 | PLA1A | 0.001653 | 0.998347 |
| NR_136304 | TLR2 | 0.002339 | -0.99766 |
| NR_136304 | PLA2G4F | 0.008841 | -0.99116 |
| NR_136304 | RHEB | 0.006618 | -0.99338 |
| NR_136304 | RIOX2 | 0.007203 | -0.9928 |
| NR_136304 | ASGR2 | 0.00919 | -0.99081 |
| NR_136304 | VWA5A | 0.003418 | -0.99658 |
| NR_136304 | NAT8 | 0.002285 | -0.99772 |
| NR_136304 | PLA1A | 0.001653 | 0.998347 |
| NR_136304 | TLR2 | 0.002339 | -0.99766 |
| NR_136304 | PLA2G4F | 0.008841 | -0.99116 |
| NR_136304 | RHEB | 0.006618 | -0.99338 |
| NR_136304 | RIOX2 | 0.007203 | -0.9928 |
| NR_136304 | ASGR2 | 0.00919 | -0.99081 |
| NR_136304 | VWA5A | 0.003418 | -0.99658 |
| NR_136304 | NAT8 | 0.002285 | -0.99772 |
| NR_136304 | PLA1A | 0.001653 | 0.998347 |
| NR_136304 | TLR2 | 0.002339 | -0.99766 |
| NR_136304 | PLA2G4F | 0.008841 | -0.99116 |
| NR_136304 | RHEB | 0.006618 | -0.99338 |
| NR_136304 | RIOX2 | 0.007203 | -0.9928 |
| NR_136304 | ASGR2 | 0.00919 | -0.99081 |
| NR_136304 | VWA5A | 0.003418 | -0.99658 |
| NR_136304 | NAT8 | 0.002285 | -0.99772 |
| NR_136304 | PLA1A | 0.001653 | 0.998347 |
| NR_136304 | TLR2 | 0.002339 | -0.99766 |
| NR_136304 | PLA2G4F | 0.008841 | -0.99116 |
| NR_136304 | RHEB | 0.006618 | -0.99338 |
| NR_136304 | RIOX2 | 0.007203 | -0.9928 |
| NR_136304 | ASGR2 | 0.00919 | -0.99081 |
| NR_136304 | VWA5A | 0.003418 | -0.99658 |
| NR_136304 | NAT8 | 0.002285 | -0.99772 |
| NR_136304 | PLA1A | 0.001653 | 0.998347 |
| NR_136304 | TLR2 | 0.002339 | -0.99766 |
| NR_136304 | PLA2G4F | 0.008841 | -0.99116 |
| ENST00000451099 | FILIP1 | 0.003958 | -0.99604 |
| ENST00000451099 | HMGXB4 | 0.005709 | -0.99429 |
| ENST00000451099 | GGCT | 0.008304 | -0.9917 |
| ENST00000451099 | RPL11 | 0.005921 | -0.99408 |
| ENST00000451099 | TMEM242 | 0.004322 | -0.99568 |
| ENST00000451099 | NLRP9 | 0.009885 | 0.990115 |
| ENST00000451099 | UNC45A | 6.55E-04 | 0.999345 |
| ENST00000451099 | CAPNS1 | 0.007549 | -0.99245 |
| ENST00000451099 | THOC5 | 0.005387 | 0.994613 |
| ENST00000451099 | TUBGCP4 | 0.004579 | 0.995421 |
| ENST00000451099 | RACK1 | 0.00112 | 0.99888 |
| ENST00000451099 | CHCHD6 | 0.005842 | 0.994158 |
| ENST00000451099 | MCM3AP | 0.007075 | 0.992925 |
| ENST00000451099 | CDKN3 | 0.00195 | -0.99805 |
| ENST00000451099 | RPUSD2 | 4.97E-04 | -0.9995 |
| ENST00000451099 | CEP63 | 0.006082 | -0.99392 |
| ENST00000451099 | USP9X | 0.008564 | 0.991436 |
| ENST00000451099 | FILIP1 | 0.003958 | -0.99604 |
| ENST00000451099 | HMGXB4 | 0.005709 | -0.99429 |
| ENST00000451099 | GGCT | 0.008304 | -0.9917 |
| ENST00000451099 | RPL11 | 0.005921 | -0.99408 |
| ENST00000451099 | TMEM242 | 0.004322 | -0.99568 |
| ENST00000451099 | NLRP9 | 0.009885 | 0.990115 |
| ENST00000451099 | UNC45A | 6.55E-04 | 0.999345 |
| ENST00000451099 | CAPNS1 | 0.007549 | -0.99245 |
| ENST00000451099 | THOC5 | 0.005387 | 0.994613 |
| ENST00000451099 | TUBGCP4 | 0.004579 | 0.995421 |
| ENST00000451099 | RACK1 | 0.00112 | 0.99888 |
| ENST00000451099 | CHCHD6 | 0.005842 | 0.994158 |
| ENST00000451099 | MCM3AP | 0.007075 | 0.992925 |
| ENST00000451099 | CDKN3 | 0.00195 | -0.99805 |
| ENST00000451099 | RPUSD2 | 4.97E-04 | -0.9995 |
| ENST00000451099 | CEP63 | 0.006082 | -0.99392 |
| ENST00000451099 | USP9X | 0.008564 | 0.991436 |
| ENST00000451099 | FILIP1 | 0.003958 | -0.99604 |
| ENST00000451099 | HMGXB4 | 0.005709 | -0.99429 |
| ENST00000451099 | GGCT | 0.008304 | -0.9917 |
| ENST00000451099 | RPL11 | 0.005921 | -0.99408 |
| ENST00000451099 | TMEM242 | 0.004322 | -0.99568 |
| ENST00000451099 | NLRP9 | 0.009885 | 0.990115 |
| ENST00000451099 | UNC45A | 6.55E-04 | 0.999345 |
| ENST00000451099 | CAPNS1 | 0.007549 | -0.99245 |
| ENST00000451099 | THOC5 | 0.005387 | 0.994613 |
| ENST00000451099 | TUBGCP4 | 0.004579 | 0.995421 |
| ENST00000451099 | RACK1 | 0.00112 | 0.99888 |
| ENST00000451099 | CHCHD6 | 0.005842 | 0.994158 |
| ENST00000451099 | MCM3AP | 0.007075 | 0.992925 |
| ENST00000451099 | CDKN3 | 0.00195 | -0.99805 |
| ENST00000451099 | RPUSD2 | 4.97E-04 | -0.9995 |
| ENST00000451099 | CEP63 | 0.006082 | -0.99392 |
| ENST00000451099 | USP9X | 0.008564 | 0.991436 |
| ENST00000451099 | FILIP1 | 0.003958 | -0.99604 |
| ENST00000451099 | HMGXB4 | 0.005709 | -0.99429 |
| ENST00000451099 | GGCT | 0.008304 | -0.9917 |
| ENST00000451099 | RPL11 | 0.005921 | -0.99408 |
| ENST00000451099 | TMEM242 | 0.004322 | -0.99568 |
| ENST00000451099 | NLRP9 | 0.009885 | 0.990115 |
| ENST00000451099 | UNC45A | 6.55E-04 | 0.999345 |
| ENST00000451099 | CAPNS1 | 0.007549 | -0.99245 |
| ENST00000451099 | THOC5 | 0.005387 | 0.994613 |
| ENST00000451099 | TUBGCP4 | 0.004579 | 0.995421 |
| ENST00000451099 | RACK1 | 0.00112 | 0.99888 |
| ENST00000451099 | CHCHD6 | 0.005842 | 0.994158 |
| ENST00000451099 | MCM3AP | 0.007075 | 0.992925 |
| ENST00000451099 | CDKN3 | 0.00195 | -0.99805 |
| ENST00000451099 | RPUSD2 | 4.97E-04 | -0.9995 |
| ENST00000451099 | CEP63 | 0.006082 | -0.99392 |
| ENST00000451099 | USP9X | 0.008564 | 0.991436 |
| ENST00000451099 | FILIP1 | 0.003958 | -0.99604 |
| ENST00000451099 | HMGXB4 | 0.005709 | -0.99429 |
| ENST00000451099 | GGCT | 0.008304 | -0.9917 |
| ENST00000451099 | RPL11 | 0.005921 | -0.99408 |
| ENST00000451099 | TMEM242 | 0.004322 | -0.99568 |
| ENST00000451099 | NLRP9 | 0.009885 | 0.990115 |
| ENST00000451099 | UNC45A | 6.55E-04 | 0.999345 |
| ENST00000451099 | CAPNS1 | 0.007549 | -0.99245 |
| ENST00000451099 | THOC5 | 0.005387 | 0.994613 |
| ENST00000451099 | TUBGCP4 | 0.004579 | 0.995421 |
| ENST00000451099 | RACK1 | 0.00112 | 0.99888 |
| ENST00000451099 | CHCHD6 | 0.005842 | 0.994158 |
| ENST00000451099 | MCM3AP | 0.007075 | 0.992925 |
| ENST00000451099 | CDKN3 | 0.00195 | -0.99805 |
| ENST00000451099 | RPUSD2 | 4.97E-04 | -0.9995 |
| ENST00000451099 | CEP63 | 0.006082 | -0.99392 |
| ENST00000451099 | USP9X | 0.008564 | 0.991436 |
| ENST00000451099 | FILIP1 | 0.003958 | -0.99604 |
| ENST00000451099 | HMGXB4 | 0.005709 | -0.99429 |
| ENST00000451099 | GGCT | 0.008304 | -0.9917 |
| ENST00000451099 | RPL11 | 0.005921 | -0.99408 |
| ENST00000451099 | TMEM242 | 0.004322 | -0.99568 |
| ENST00000451099 | NLRP9 | 0.009885 | 0.990115 |
| ENST00000451099 | UNC45A | 6.55E-04 | 0.999345 |
| ENST00000451099 | CAPNS1 | 0.007549 | -0.99245 |
| ENST00000451099 | THOC5 | 0.005387 | 0.994613 |
| ENST00000451099 | TUBGCP4 | 0.004579 | 0.995421 |
| ENST00000451099 | RACK1 | 0.00112 | 0.99888 |
| ENST00000451099 | CHCHD6 | 0.005842 | 0.994158 |
| ENST00000451099 | MCM3AP | 0.007075 | 0.992925 |
| ENST00000451099 | CDKN3 | 0.00195 | -0.99805 |
| ENST00000451099 | RPUSD2 | 4.97E-04 | -0.9995 |
| ENST00000451099 | CEP63 | 0.006082 | -0.99392 |
| ENST00000451099 | USP9X | 0.008564 | 0.991436 |
| ENST00000451099 | FILIP1 | 0.003958 | -0.99604 |
| ENST00000451099 | HMGXB4 | 0.005709 | -0.99429 |
| ENST00000451099 | GGCT | 0.008304 | -0.9917 |
| ENST00000451099 | RPL11 | 0.005921 | -0.99408 |
| ENST00000451099 | TMEM242 | 0.004322 | -0.99568 |
| ENST00000451099 | NLRP9 | 0.009885 | 0.990115 |
| ENST00000451099 | UNC45A | 6.55E-04 | 0.999345 |
| ENST00000451099 | CAPNS1 | 0.007549 | -0.99245 |
| ENST00000451099 | THOC5 | 0.005387 | 0.994613 |
| ENST00000451099 | TUBGCP4 | 0.004579 | 0.995421 |
| ENST00000451099 | RACK1 | 0.00112 | 0.99888 |
| ENST00000451099 | CHCHD6 | 0.005842 | 0.994158 |
| ENST00000451099 | MCM3AP | 0.007075 | 0.992925 |
| ENST00000451099 | CDKN3 | 0.00195 | -0.99805 |
| ENST00000451099 | RPUSD2 | 4.97E-04 | -0.9995 |
| ENST00000451099 | CEP63 | 0.006082 | -0.99392 |
| ENST00000451099 | USP9X | 0.008564 | 0.991436 |
| ENST00000451099 | FILIP1 | 0.003958 | -0.99604 |
| ENST00000451099 | HMGXB4 | 0.005709 | -0.99429 |
| ENST00000451099 | GGCT | 0.008304 | -0.9917 |
| ENST00000451099 | RPL11 | 0.005921 | -0.99408 |
| ENST00000451099 | TMEM242 | 0.004322 | -0.99568 |
| ENST00000451099 | NLRP9 | 0.009885 | 0.990115 |
| ENST00000451099 | UNC45A | 6.55E-04 | 0.999345 |
| ENST00000451099 | CAPNS1 | 0.007549 | -0.99245 |
| ENST00000451099 | THOC5 | 0.005387 | 0.994613 |
| ENST00000451099 | TUBGCP4 | 0.004579 | 0.995421 |
| ENST00000451099 | RACK1 | 0.00112 | 0.99888 |
| ENST00000451099 | CHCHD6 | 0.005842 | 0.994158 |
| ENST00000451099 | MCM3AP | 0.007075 | 0.992925 |
| ENST00000451099 | CDKN3 | 0.00195 | -0.99805 |
| ENST00000451099 | RPUSD2 | 4.97E-04 | -0.9995 |
| ENST00000451099 | CEP63 | 0.006082 | -0.99392 |
| ENST00000451099 | USP9X | 0.008564 | 0.991436 |
| ENST00000451099 | FILIP1 | 0.003958 | -0.99604 |
| ENST00000451099 | HMGXB4 | 0.005709 | -0.99429 |
| ENST00000451099 | GGCT | 0.008304 | -0.9917 |
| ENST00000451099 | RPL11 | 0.005921 | -0.99408 |
| ENST00000451099 | TMEM242 | 0.004322 | -0.99568 |
| ENST00000451099 | NLRP9 | 0.009885 | 0.990115 |
| ENST00000451099 | UNC45A | 6.55E-04 | 0.999345 |
| ENST00000451099 | CAPNS1 | 0.007549 | -0.99245 |
| ENST00000451099 | THOC5 | 0.005387 | 0.994613 |
| ENST00000451099 | TUBGCP4 | 0.004579 | 0.995421 |
| ENST00000451099 | RACK1 | 0.00112 | 0.99888 |
| ENST00000451099 | CHCHD6 | 0.005842 | 0.994158 |
| ENST00000451099 | MCM3AP | 0.007075 | 0.992925 |
| ENST00000451099 | CDKN3 | 0.00195 | -0.99805 |
| ENST00000451099 | RPUSD2 | 4.97E-04 | -0.9995 |
| ENST00000451099 | CEP63 | 0.006082 | -0.99392 |
| ENST00000451099 | USP9X | 0.008564 | 0.991436 |
| ENST00000451099 | FILIP1 | 0.003958 | -0.99604 |
| ENST00000451099 | HMGXB4 | 0.005709 | -0.99429 |
| ENST00000451099 | GGCT | 0.008304 | -0.9917 |
| ENST00000451099 | RPL11 | 0.005921 | -0.99408 |
| ENST00000451099 | TMEM242 | 0.004322 | -0.99568 |
| ENST00000451099 | NLRP9 | 0.009885 | 0.990115 |
| ENST00000451099 | UNC45A | 6.55E-04 | 0.999345 |
| ENST00000451099 | CAPNS1 | 0.007549 | -0.99245 |
| ENST00000451099 | THOC5 | 0.005387 | 0.994613 |
| ENST00000451099 | TUBGCP4 | 0.004579 | 0.995421 |
| ENST00000451099 | RACK1 | 0.00112 | 0.99888 |
| ENST00000451099 | CHCHD6 | 0.005842 | 0.994158 |
| ENST00000451099 | MCM3AP | 0.007075 | 0.992925 |
| ENST00000451099 | CDKN3 | 0.00195 | -0.99805 |
| ENST00000451099 | RPUSD2 | 4.97E-04 | -0.9995 |
| ENST00000451099 | CEP63 | 0.006082 | -0.99392 |
| ENST00000451099 | USP9X | 0.008564 | 0.991436 |
| ENST00000577694 | RRAS2 | 0.00569 | 0.99431 |
| ENST00000577694 | RRAS2 | 0.00569 | 0.99431 |
| ENST00000441053 | TMED3 | 0.00824 | -0.99176 |
| ENST00000441053 | DEAF1 | 0.008862 | 0.991138 |
| ENST00000441053 | VRK3 | 6.56E-04 | 0.999344 |
| ENST00000441053 | BLCAP | 3.73E-04 | 0.999627 |
| ENST00000441053 | FANCD2OS | 0.007342 | 0.992658 |
| ENST00000441053 | CATG00000023328.1 | 0.003056 | -0.99694 |
| ENST00000441053 | ACSL6 | 5.59E-04 | 0.999441 |
| ENST00000441053 | CATG00000024701.1 | 0.007214 | 0.992786 |
| ENST00000585890 | NOC4L | 0.006501 | 0.993499 |
| ENST00000585890 | CATG00000080699.1 | 0.002301 | 0.997699 |
| ENST00000585890 | SLC24A5 | 0.009133 | -0.99087 |
| ENST00000585890 | TCOF1 | 0.004763 | 0.995237 |
| ENST00000585890 | SAMD13 | 0.005253 | 0.994747 |
| ENST00000585890 | ZDHHC16 | 2.84E-04 | -0.99972 |
| ENST00000585890 | CDKL2 | 0.008566 | 0.991434 |
| ENST00000585890 | MACROD2 | 0.001153 | 0.998847 |
| ENST00000585890 | CHST12 | 0 | 1 |
| ENST00000585890 | HIPK2 | 0.004794 | -0.99521 |
| ENST00000585890 | PCDHB8 | 0 | 1 |
| ENST00000547357 | AHNAK2 | 0.003649 | 0.996351 |
| ENST00000547357 | CHSY3 | 0.007994 | 0.992006 |
| ENST00000547357 | HOMER3 | 0.00393 | -0.99607 |
| ENST00000547357 | ARPC4-TTLL3 | 0.008196 | -0.9918 |
| ENST00000547357 | CATG00000113928.1 | 0.006418 | -0.99358 |
| ENST00000547357 | MMP24 | 0.005489 | -0.99451 |
| ENST00000547357 | ZNF624 | 0.005764 | 0.994236 |
| ENST00000547357 | AHNAK2 | 0.003649 | 0.996351 |
| ENST00000547357 | CHSY3 | 0.007994 | 0.992006 |
| ENST00000547357 | HOMER3 | 0.00393 | -0.99607 |
| ENST00000547357 | ARPC4-TTLL3 | 0.008196 | -0.9918 |
| ENST00000547357 | CATG00000113928.1 | 0.006418 | -0.99358 |
| ENST00000547357 | MMP24 | 0.005489 | -0.99451 |
| ENST00000547357 | ZNF624 | 0.005764 | 0.994236 |
| ENST00000547357 | AHNAK2 | 0.003649 | 0.996351 |
| ENST00000547357 | CHSY3 | 0.007994 | 0.992006 |
| ENST00000547357 | HOMER3 | 0.00393 | -0.99607 |
| ENST00000547357 | ARPC4-TTLL3 | 0.008196 | -0.9918 |
| ENST00000547357 | CATG00000113928.1 | 0.006418 | -0.99358 |
| ENST00000547357 | MMP24 | 0.005489 | -0.99451 |
| ENST00000547357 | ZNF624 | 0.005764 | 0.994236 |
| ENST00000547357 | AHNAK2 | 0.003649 | 0.996351 |
| ENST00000547357 | CHSY3 | 0.007994 | 0.992006 |
| ENST00000547357 | HOMER3 | 0.00393 | -0.99607 |
| ENST00000547357 | ARPC4-TTLL3 | 0.008196 | -0.9918 |
| ENST00000547357 | CATG00000113928.1 | 0.006418 | -0.99358 |
| ENST00000547357 | MMP24 | 0.005489 | -0.99451 |
| ENST00000547357 | ZNF624 | 0.005764 | 0.994236 |
| ENST00000547357 | AHNAK2 | 0.003649 | 0.996351 |
| ENST00000547357 | CHSY3 | 0.007994 | 0.992006 |
| ENST00000547357 | HOMER3 | 0.00393 | -0.99607 |
| ENST00000547357 | ARPC4-TTLL3 | 0.008196 | -0.9918 |
| ENST00000547357 | CATG00000113928.1 | 0.006418 | -0.99358 |
| ENST00000547357 | MMP24 | 0.005489 | -0.99451 |
| ENST00000547357 | ZNF624 | 0.005764 | 0.994236 |
| ENST00000547357 | AHNAK2 | 0.003649 | 0.996351 |
| ENST00000547357 | CHSY3 | 0.007994 | 0.992006 |
| ENST00000547357 | HOMER3 | 0.00393 | -0.99607 |
| ENST00000547357 | ARPC4-TTLL3 | 0.008196 | -0.9918 |
| ENST00000547357 | CATG00000113928.1 | 0.006418 | -0.99358 |
| ENST00000547357 | MMP24 | 0.005489 | -0.99451 |
| ENST00000547357 | ZNF624 | 0.005764 | 0.994236 |
| ENST00000547357 | AHNAK2 | 0.003649 | 0.996351 |
| ENST00000547357 | CHSY3 | 0.007994 | 0.992006 |
| ENST00000547357 | HOMER3 | 0.00393 | -0.99607 |
| ENST00000547357 | ARPC4-TTLL3 | 0.008196 | -0.9918 |
| ENST00000547357 | CATG00000113928.1 | 0.006418 | -0.99358 |
| ENST00000547357 | MMP24 | 0.005489 | -0.99451 |
| ENST00000547357 | ZNF624 | 0.005764 | 0.994236 |
| T081143 | RRAS2 | 0.001108 | 0.998892 |
| T081143 | ZP1 | 0.007554 | 0.992446 |
| T081143 | RRAS2 | 0.001108 | 0.998892 |
| T081143 | ZP1 | 0.007554 | 0.992446 |
| T081143 | RRAS2 | 0.001108 | 0.998892 |
| T081143 | ZP1 | 0.007554 | 0.992446 |
| T081143 | RRAS2 | 0.001108 | 0.998892 |
| T081143 | ZP1 | 0.007554 | 0.992446 |
| T081143 | RRAS2 | 0.001108 | 0.998892 |
| T081143 | ZP1 | 0.007554 | 0.992446 |
| T081143 | RRAS2 | 0.001108 | 0.998892 |
| T081143 | ZP1 | 0.007554 | 0.992446 |
| T081143 | RRAS2 | 0.001108 | 0.998892 |
| T081143 | ZP1 | 0.007554 | 0.992446 |
| T081143 | RRAS2 | 0.001108 | 0.998892 |
| T081143 | ZP1 | 0.007554 | 0.992446 |
| T081143 | RRAS2 | 0.001108 | 0.998892 |
| T081143 | ZP1 | 0.007554 | 0.992446 |
| T081143 | RRAS2 | 0.001108 | 0.998892 |
| T081143 | ZP1 | 0.007554 | 0.992446 |
| T081143 | RRAS2 | 0.001108 | 0.998892 |
| T081143 | ZP1 | 0.007554 | 0.992446 |
| T081143 | RRAS2 | 0.001108 | 0.998892 |
| T081143 | ZP1 | 0.007554 | 0.992446 |
| T081143 | RRAS2 | 0.001108 | 0.998892 |
| T081143 | ZP1 | 0.007554 | 0.992446 |
| ENST00000604994 | LGALS1 | 0.005517 | 0.994483 |
| ENST00000515617 | GNL1 | 0.003065 | -0.99694 |
| ENST00000515617 | DEAF1 | 0.009014 | 0.990986 |
| ENST00000515617 | DNAJA4 | 0.0029 | 0.9971 |
| ENST00000515617 | GPR25 | 5.00E-04 | 0.9995 |
| ENST00000515617 | FANCD2OS | 0.008008 | 0.991992 |
| ENST00000515617 | PMPCA | 0.007926 | 0.992074 |
| ENST00000515617 | SHC2 | 0.004254 | 0.995746 |
| ENST00000515617 | EFEMP2 | 0.003665 | -0.99634 |
| ENST00000515617 | OR4X1 | 0.008114 | 0.991886 |
| ENST00000515617 | HLA-F | 0.004131 | 0.995869 |
| T274512 | AHNAK2 | 0.005814 | 0.994186 |
| T274512 | NCCRP1 | 0.004902 | 0.995098 |
| T274512 | C20orf203 | 0.006883 | 0.993117 |
| T274512 | DUSP15 | 0.004878 | 0.995122 |
| T274512 | HOMER3 | 0.005793 | -0.99421 |
| T274512 | AASDHPPT | 0.004613 | 0.995387 |
| T274512 | ARPC4-TTLL3 | 6.05E-04 | -0.9994 |
| T274512 | MAMLD1 | 0.00379 | -0.99621 |
| T274512 | ZNF214 | 0.007589 | -0.99241 |
| T274512 | ZNF624 | 0.006508 | 0.993492 |
| T274512 | OR5D18 | 0.005526 | -0.99447 |
| ENST00000422480 | PNCK | 0.007558 | -0.99244 |
| ENST00000422480 | E4F1 | 0.00303 | -0.99697 |
| ENST00000422480 | C15orf65 | 0.00404 | 0.99596 |
| ENST00000422480 | NTNG1 | 0.003045 | -0.99695 |
| ENST00000422480 | NDUFAF8 | 0.008374 | -0.99163 |
| ENST00000422480 | MROH9 | 0.004261 | 0.995739 |
| ENST00000422480 | PMEPA1 | 0.003439 | 0.996561 |
| ENST00000422480 | NDUFC1 | 1.50E-04 | -0.99985 |
| ENST00000422480 | FCRL1 | 0.001843 | 0.998157 |
| T000463 | NOC4L | 0.006501 | 0.993499 |
| T000463 | CATG00000080699.1 | 0.002301 | 0.997699 |
| T000463 | SLC24A5 | 0.009133 | -0.99087 |
| T000463 | TCOF1 | 0.004763 | 0.995237 |
| T000463 | SAMD13 | 0.005253 | 0.994747 |
| T000463 | ZDHHC16 | 2.84E-04 | -0.99972 |
| T000463 | CDKL2 | 0.008566 | 0.991434 |
| T000463 | MACROD2 | 0.001153 | 0.998847 |
| T000463 | CHST12 | 0 | 1 |
| T000463 | HIPK2 | 0.004794 | -0.99521 |
| T000463 | PCDHB8 | 0 | 1 |
| TCONS_00026396 | CATG00000086946.1 | 0.008261 | -0.99174 |
| TCONS_00026396 | WT1 | 0.007425 | 0.992575 |
| TCONS_00026396 | CATG00000039284.1 | 0.001651 | 0.998349 |
| TCONS_00026396 | SCAMP3 | 0.008868 | 0.991132 |
| TCONS_00026396 | ORM1 | 0.008183 | -0.99182 |
| MICT00000092884 | PLAGL1 | 0.001646 | -0.99835 |
| MICT00000092884 | RHBDD3 | 0.001364 | -0.99864 |
| MICT00000092884 | AIFM3 | 0.007155 | -0.99285 |
| MICT00000092884 | VGLL3 | 0.003531 | 0.996469 |
| MICT00000092884 | MMP2 | 0.001309 | 0.998691 |
| MICT00000092884 | FAM186A | 0.006703 | 0.993297 |
| MICT00000092884 | IL1R2 | 0.007681 | 0.992319 |
| MICT00000092884 | PLAGL1 | 0.001646 | -0.99835 |
| MICT00000092884 | RHBDD3 | 0.001364 | -0.99864 |
| MICT00000092884 | AIFM3 | 0.007155 | -0.99285 |
| MICT00000092884 | VGLL3 | 0.003531 | 0.996469 |
| MICT00000092884 | MMP2 | 0.001309 | 0.998691 |
| MICT00000092884 | FAM186A | 0.006703 | 0.993297 |
| MICT00000092884 | IL1R2 | 0.007681 | 0.992319 |
| MICT00000092884 | PLAGL1 | 0.001646 | -0.99835 |
| MICT00000092884 | RHBDD3 | 0.001364 | -0.99864 |
| MICT00000092884 | AIFM3 | 0.007155 | -0.99285 |
| MICT00000092884 | VGLL3 | 0.003531 | 0.996469 |
| MICT00000092884 | MMP2 | 0.001309 | 0.998691 |
| MICT00000092884 | FAM186A | 0.006703 | 0.993297 |
| MICT00000092884 | IL1R2 | 0.007681 | 0.992319 |
| MICT00000092884 | PLAGL1 | 0.001646 | -0.99835 |
| MICT00000092884 | RHBDD3 | 0.001364 | -0.99864 |
| MICT00000092884 | AIFM3 | 0.007155 | -0.99285 |
| MICT00000092884 | VGLL3 | 0.003531 | 0.996469 |
| MICT00000092884 | MMP2 | 0.001309 | 0.998691 |
| MICT00000092884 | FAM186A | 0.006703 | 0.993297 |
| MICT00000092884 | IL1R2 | 0.007681 | 0.992319 |
| MICT00000092884 | PLAGL1 | 0.001646 | -0.99835 |
| MICT00000092884 | RHBDD3 | 0.001364 | -0.99864 |
| MICT00000092884 | AIFM3 | 0.007155 | -0.99285 |
| MICT00000092884 | VGLL3 | 0.003531 | 0.996469 |
| MICT00000092884 | MMP2 | 0.001309 | 0.998691 |
| MICT00000092884 | FAM186A | 0.006703 | 0.993297 |
| MICT00000092884 | IL1R2 | 0.007681 | 0.992319 |
| T340632 | FILIP1 | 8.88E-04 | -0.99911 |
| T340632 | WNT16 | 0.009081 | 0.990919 |
| T340632 | HMGXB4 | 0.004443 | -0.99556 |
| T340632 | RPL11 | 0.005665 | -0.99434 |
| T340632 | NLRP9 | 0.009189 | 0.990811 |
| T340632 | PMPCA | 0.005657 | 0.994343 |
| T340632 | UNC45A | 0.004161 | 0.995839 |
| T340632 | CD180 | 0.005445 | -0.99455 |
| T340632 | CAPNS1 | 0.002741 | -0.99726 |
| T340632 | RACK1 | 0.001317 | 0.998683 |
| T340632 | KRTAP4-7 | 0.004845 | 0.995155 |
| T340632 | CDKN3 | 0.002305 | -0.99769 |
| T340632 | RPUSD2 | 0.001567 | -0.99843 |
| T340632 | USP9X | 0.009663 | 0.990337 |
| T198098 | SLC38A10 | 0.009423 | 0.990577 |
| T198098 | ZNF30 | 0.005924 | -0.99408 |
| T198098 | SZRD1 | 0.003162 | -0.99684 |
| T198098 | FAM84A | 7.39E-04 | -0.99926 |
| T198098 | GMPPA | 0.005593 | -0.99441 |
| T198098 | G6PC3 | 0.001303 | 0.998697 |
| T198098 | MAP7 | 0.004518 | 0.995482 |
| T198098 | C16orf78 | 0.009031 | 0.990969 |
| T198098 | EFCAB8 | 0.006386 | 0.993614 |
| T198098 | OR5D18 | 0.009333 | 0.990667 |
| ENST00000508851 | RPS7 | 0.00755 | 0.99245 |
| ENST00000508851 | ACOT12 | 0.00296 | 0.99704 |
| ENST00000508851 | BCAS4 | 0.009237 | 0.990763 |
| ENST00000508851 | PROKR1 | 0.001796 | -0.9982 |
| ENST00000508851 | PELP1 | 0.009488 | 0.990512 |
| ENST00000508851 | TSC22D4 | 0.008786 | -0.99121 |
| ENST00000508851 | ABCA4 | 0.008916 | 0.991084 |
| ENST00000508851 | OR8B4 | 0.003457 | -0.99654 |
| ENST00000508851 | MYH4 | 0.001247 | 0.998753 |
| ENST00000508851 | CLEC4E | 8.60E-04 | 0.99914 |
| ENST00000508851 | FANCL | 0.003747 | 0.996253 |
| ENST00000508851 | ARG1 | 0.009143 | 0.990857 |
| compmerge.2359.pooled.chr8 | ZFY | 0.008507 | -0.99149 |
| compmerge.2359.pooled.chr8 | EXOC3L2 | 0.008003 | 0.991997 |
| compmerge.2359.pooled.chr8 | PLD3 | 0.001659 | -0.99834 |
| compmerge.2359.pooled.chr8 | RAB8A | 0.001925 | -0.99808 |
| compmerge.2359.pooled.chr8 | EEF1B2 | 0.008226 | 0.991774 |
| compmerge.2359.pooled.chr8 | SSX1 | 0.005837 | 0.994163 |
| compmerge.2359.pooled.chr8 | WSCD2 | 2.92E-04 | 0.999708 |
| compmerge.2359.pooled.chr8 | GRIN3B | 0.002602 | -0.9974 |
| compmerge.2359.pooled.chr8 | TNFAIP6 | 0.005427 | -0.99457 |
| compmerge.2359.pooled.chr8 | PTPRK | 0.002007 | 0.997993 |
| compmerge.2359.pooled.chr8 | CATG00000039609.1 | 0.005354 | 0.994646 |
| compmerge.2359.pooled.chr8 | DEFA4 | 0.009549 | 0.990451 |
| T339955 | SPANXN4 | 0.009674 | 0.990326 |
| T339955 | MYT1L | 8.81E-04 | 0.999119 |
| T339955 | CATG00000074949.1 | 0.002468 | 0.997532 |
| T339955 | OR8G1 | 0.009606 | 0.990394 |
| T339955 | FRAT1 | 0.006215 | -0.99379 |
| T339955 | KCNE4 | 0.00129 | -0.99871 |
| T099310 | RRAS2 | 6.85E-04 | 0.999315 |
| T099310 | ZP1 | 0.008911 | 0.991089 |
| T241883 | NOC4L | 0.006501 | 0.993499 |
| T241883 | CATG00000080699.1 | 0.002301 | 0.997699 |
| T241883 | SLC24A5 | 0.009133 | -0.99087 |
| T241883 | TCOF1 | 0.004763 | 0.995237 |
| T241883 | SAMD13 | 0.005253 | 0.994747 |
| T241883 | ZDHHC16 | 2.84E-04 | -0.99972 |
| T241883 | CDKL2 | 0.008566 | 0.991434 |
| T241883 | MACROD2 | 0.001153 | 0.998847 |
| T241883 | CHST12 | 0 | 1 |
| T241883 | HIPK2 | 0.004794 | -0.99521 |
| T241883 | PCDHB8 | 0 | 1 |
| T241883 | NOC4L | 0.006501 | 0.993499 |
| T241883 | CATG00000080699.1 | 0.002301 | 0.997699 |
| T241883 | SLC24A5 | 0.009133 | -0.99087 |
| T241883 | TCOF1 | 0.004763 | 0.995237 |
| T241883 | SAMD13 | 0.005253 | 0.994747 |
| T241883 | ZDHHC16 | 2.84E-04 | -0.99972 |
| T241883 | CDKL2 | 0.008566 | 0.991434 |
| T241883 | MACROD2 | 0.001153 | 0.998847 |
| T241883 | CHST12 | 0 | 1 |
| T241883 | HIPK2 | 0.004794 | -0.99521 |
| T241883 | PCDHB8 | 0 | 1 |
| T241883 | NOC4L | 0.006501 | 0.993499 |
| T241883 | CATG00000080699.1 | 0.002301 | 0.997699 |
| T241883 | SLC24A5 | 0.009133 | -0.99087 |
| T241883 | TCOF1 | 0.004763 | 0.995237 |
| T241883 | SAMD13 | 0.005253 | 0.994747 |
| T241883 | ZDHHC16 | 2.84E-04 | -0.99972 |
| T241883 | CDKL2 | 0.008566 | 0.991434 |
| T241883 | MACROD2 | 0.001153 | 0.998847 |
| T241883 | CHST12 | 0 | 1 |
| T241883 | HIPK2 | 0.004794 | -0.99521 |
| T241883 | PCDHB8 | 0 | 1 |
| T241883 | NOC4L | 0.006501 | 0.993499 |
| T241883 | CATG00000080699.1 | 0.002301 | 0.997699 |
| T241883 | SLC24A5 | 0.009133 | -0.99087 |
| T241883 | TCOF1 | 0.004763 | 0.995237 |
| T241883 | SAMD13 | 0.005253 | 0.994747 |
| T241883 | ZDHHC16 | 2.84E-04 | -0.99972 |
| T241883 | CDKL2 | 0.008566 | 0.991434 |
| T241883 | MACROD2 | 0.001153 | 0.998847 |
| T241883 | CHST12 | 0 | 1 |
| T241883 | HIPK2 | 0.004794 | -0.99521 |
| T241883 | PCDHB8 | 0 | 1 |
| ENST00000557465 | LGALS1 | 0.003437 | 0.996563 |
| ENST00000557465 | DUSP2 | 0.002164 | 0.997836 |
| ENST00000549725 | UGT2B28 | 0.005246 | -0.99475 |
| ENST00000549725 | ORM1 | 0.007542 | -0.99246 |
| ENST00000549725 | UGT2B28 | 0.005246 | -0.99475 |
| ENST00000549725 | ORM1 | 0.007542 | -0.99246 |
| ENST00000549725 | UGT2B28 | 0.005246 | -0.99475 |
| ENST00000549725 | ORM1 | 0.007542 | -0.99246 |
| T370928 | CATG00000038628.1 | 0.005536 | -0.99446 |
| T370928 | RAB5B | 0.007119 | 0.992881 |
| NR_146730 | PDE4C | 8.61E-04 | -0.99914 |
| NR_146730 | RUSC2 | 0.002684 | 0.997316 |
| NR_146730 | CATG00000067666.1 | 0.001279 | 0.998721 |
| NR_146730 | CDA | 0.004424 | 0.995576 |
| NR_146730 | ZP1 | 0.004492 | 0.995508 |
| TCONS_00018525 | NOC4L | 1.07E-04 | -0.99989 |
| TCONS_00018525 | TUBB2A | 0.005945 | 0.994055 |
| TCONS_00018525 | ZDHHC16 | 0.005023 | 0.994977 |
| TCONS_00018525 | CDKL2 | 0.001388 | -0.99861 |
| TCONS_00018525 | MACROD2 | 0.005068 | -0.99493 |
| TCONS_00018525 | CR749689 | 0.007253 | 0.992747 |
| TCONS_00018525 | CHST12 | 0.00757 | -0.99243 |
| TCONS_00018525 | PCDHB8 | 0.00757 | -0.99243 |
| ENST00000411728 | NOC4L | 0.006501 | 0.993499 |
| ENST00000411728 | CATG00000080699.1 | 0.002301 | 0.997699 |
| ENST00000411728 | SLC24A5 | 0.009133 | -0.99087 |
| ENST00000411728 | TCOF1 | 0.004763 | 0.995237 |
| ENST00000411728 | SAMD13 | 0.005253 | 0.994747 |
| ENST00000411728 | ZDHHC16 | 2.84E-04 | -0.99972 |
| ENST00000411728 | CDKL2 | 0.008566 | 0.991434 |
| ENST00000411728 | MACROD2 | 0.001153 | 0.998847 |
| ENST00000411728 | CHST12 | 0 | 1 |
| ENST00000411728 | HIPK2 | 0.004794 | -0.99521 |
| ENST00000411728 | PCDHB8 | 0 | 1 |
| NR_125875 | FCN2 | 0.003175 | 0.996825 |
| NR_125875 | GBA | 0.006362 | 0.993638 |
| NR_125875 | NFE2L2 | 0.008138 | -0.99186 |
| NR_125875 | NPDC1 | 0.009928 | 0.990072 |
| NR_125875 | SIVA1 | 0.002569 | -0.99743 |
| NR_125875 | PCNX3 | 0.004987 | -0.99501 |
| NR_125875 | CDK11B | 0.003544 | -0.99646 |
| NR_125875 | TCTEX1D4 | 7.04E-04 | -0.9993 |
| NR_125875 | TIGD3 | 0.009454 | -0.99055 |
| NR_125875 | MAP1LC3A | 0.007087 | 0.992913 |
| NR_125875 | SMIM6 | 0.006861 | 0.993139 |
| NR_125875 | SH3RF3 | 0.001864 | -0.99814 |
| NR_125875 | ACMSD | 0.001347 | -0.99865 |
| ENST00000427615 | TMED3 | 0.00676 | -0.99324 |
| ENST00000427615 | TAS2R42 | 0.006578 | 0.993422 |
| ENST00000427615 | PPP3CC | 0.003225 | -0.99677 |
| ENST00000427615 | PSRC1 | 0.004634 | -0.99537 |
| ENST00000427615 | LTA4H | 0.002024 | -0.99798 |
| ENST00000427615 | TUT4 | 0.004727 | -0.99527 |
| ENST00000427615 | WDR33 | 3.38E-04 | 0.999662 |
| ENST00000427615 | CATG00000053512.1 | 0.005701 | -0.9943 |
| ENST00000427615 | HLF | 0.009987 | 0.990013 |
| ENST00000427615 | CATG00000063823.1 | 0.00472 | 0.99528 |
| ENST00000427615 | ZNF607 | 0.004836 | 0.995164 |
| ENST00000427615 | PSMD4 | 0.004445 | -0.99556 |
| ENST00000427615 | SLC12A3 | 0.006991 | -0.99301 |
| ENST00000427615 | OR6F1 | 0.003292 | 0.996708 |
| ENST00000427615 | ADAM28 | 0.004591 | -0.99541 |
| ENST00000427615 | CATG00000087047.1 | 0.003399 | 0.996601 |
| ENST00000427615 | AC109583.1 | 0.009265 | 0.990735 |
| ENST00000427615 | CFAP410 | 0.009095 | 0.990905 |
| ENST00000427615 | RAD51AP2 | 4.75E-04 | -0.99952 |
| ENST00000650264 | ZNF490 | 0.005772 | -0.99423 |
| ENST00000650264 | HEYL | 9.07E-04 | 0.999093 |
| ENST00000650264 | TMEM236 | 0.002211 | -0.99779 |
| ENST00000650264 | LILRB5 | 0.008655 | 0.991345 |
| ENST00000650264 | SCYGR6 | 0.004914 | -0.99509 |
| ENST00000650264 | BRSK2 | 0.003327 | -0.99667 |
| ENST00000650264 | LCE4A | 0.004984 | 0.995016 |
| ENST00000650264 | LYPD8 | 0.008745 | 0.991255 |
| ENST00000650264 | SCRG1 | 0.00201 | 0.99799 |
| GSE61474_TCONS_00184163 | ZDBF2 | 0.009699 | 0.990301 |
| GSE61474_TCONS_00184163 | CATG00000029374.1 | 0.001547 | 0.998453 |
| T149270 | RIPK1 | 0.007282 | 0.992718 |
| T149270 | NAPA | 0.003163 | -0.99684 |
| T149270 | PPP1CB | 4.11E-04 | -0.99959 |
| T149270 | P3H3 | 0.007875 | 0.992125 |
| T149270 | PTGER1 | 0.004133 | 0.995867 |
| T149270 | PGM5 | 0.006067 | 0.993933 |
| T149270 | CATG00000107158.1 | 0.002666 | -0.99733 |
| T149270 | CATG00000110054.1 | 0.001334 | -0.99867 |
| T149270 | YY2 | 0.003395 | 0.996605 |
| T149270 | RCSD1 | 0.004928 | 0.995072 |
| T149270 | KIAA1211L | 0.008982 | 0.991018 |
| T149270 | CHI3L1 | 0.005546 | -0.99445 |
| T149270 | CATG00000020281.1 | 1.90E-04 | 0.99981 |
| ENST00000561336 | RPS7 | 0.004997 | 0.995003 |
| ENST00000561336 | ACOT12 | 5.10E-04 | 0.99949 |
| ENST00000561336 | BCAS4 | 0.004151 | 0.995849 |
| ENST00000561336 | PROKR1 | 0.003459 | -0.99654 |
| ENST00000561336 | PELP1 | 0.005013 | 0.994987 |
| ENST00000561336 | OR8B4 | 0.001682 | -0.99832 |
| ENST00000561336 | MYH4 | 3.03E-05 | 0.99997 |
| ENST00000561336 | CLEC4E | 0.002341 | 0.997659 |
| ENST00000561336 | KNCN | 0.008866 | 0.991134 |
| ENST00000561336 | FANCL | 0.002127 | 0.997873 |
| ENST00000561336 | CAMK2N2 | 0.005804 | 0.994196 |
| T308234 | TRMT11 | 0.004627 | 0.995373 |
| T308234 | ADGRE1 | 0.00923 | 0.99077 |
| T308234 | CPSF3 | 0.007961 | 0.992039 |
| T308234 | C11orf71 | 0.00857 | 0.99143 |
| T308234 | MYLK3 | 0.001478 | 0.998522 |
| T308234 | SIRT6 | 0.003425 | 0.996575 |
| T308234 | SLC39A12 | 0.00739 | -0.99261 |
| ENST00000414452 | TAS2R42 | 0.00768 | -0.99232 |
| ENST00000414452 | GRINA | 0.004577 | -0.99542 |
| ENST00000414452 | RSL1D1 | 0.008954 | 0.991046 |
| ENST00000414452 | ABCB8 | 0.00153 | -0.99847 |
| ENST00000414452 | LILRB1 | 0.004335 | 0.995665 |
| ENST00000414452 | RASL10A | 0.002686 | 0.997314 |
| ENST00000414452 | LTA4H | 0.006557 | 0.993443 |
| ENST00000414452 | HERPUD2 | 0.001781 | 0.998219 |
| ENST00000414452 | TUT4 | 0.004657 | 0.995343 |
| ENST00000414452 | NFX1 | 0.003992 | 0.996008 |
| ENST00000414452 | PEA15 | 0.004129 | -0.99587 |
| ENST00000414452 | ZNF579 | 0.007761 | -0.99224 |
| ENST00000414452 | IFT122 | 0.008576 | -0.99142 |
| ENST00000414452 | WNT8B | 0.006619 | -0.99338 |
| ENST00000414452 | PTPN23 | 0.002933 | -0.99707 |
| ENST00000414452 | UNC5C | 0.005859 | 0.994141 |
| ENST00000414452 | AGO2 | 8.55E-04 | -0.99915 |
| ENST00000414452 | TRIM47 | 0.002415 | -0.99758 |
| ENST00000414452 | DHDH | 0.005482 | -0.99452 |
| ENST00000414452 | CATG00000087047.1 | 0.004548 | -0.99545 |
| ENST00000414452 | HPS1 | 0.008601 | -0.9914 |
| ENST00000607327 | GATA4 | 0.00335 | -0.99665 |
| ENST00000607327 | CYP21A2 | 0.00544 | -0.99456 |
| ENST00000607327 | IL4I1 | 0.007745 | 0.992255 |
| ENST00000607327 | SLC39A12 | 0.007759 | -0.99224 |
| ENST00000607327 | HSDL2 | 0.008161 | 0.991839 |
| ENST00000607327 | SYNGR3 | 0.004287 | -0.99571 |
| ENST00000448858 | POP1 | 0.005852 | 0.994148 |
| ENST00000448858 | PLD3 | 0.007637 | 0.992363 |
| ENST00000448858 | TMEM230 | 0.00177 | 0.99823 |
| ENST00000448858 | RAB8A | 0.001757 | 0.998243 |
| ENST00000448858 | EEF1B2 | 0.001005 | -0.99899 |
| ENST00000448858 | WSCD2 | 0.009547 | -0.99045 |
| ENST00000448858 | GRIN3B | 0.007802 | 0.992198 |
| ENST00000448858 | KIAA1211L | 0.005198 | -0.9948 |
| ENST00000448858 | TNFAIP6 | 1.94E-04 | 0.999806 |
| ENST00000448858 | PTPRK | 0.00194 | -0.99806 |
| ENST00000448858 | CATG00000039609.1 | 0.009413 | -0.99059 |
| ENST00000596289 | PLEKHD1 | 0.005058 | -0.99494 |
| ENST00000596289 | MLLT1 | 0.004973 | 0.995027 |
| ENST00000596289 | GPCPD1 | 0.004299 | -0.9957 |
| ENST00000596289 | STMND1 | 0.005219 | 0.994781 |
| ENST00000596289 | LYPD8 | 0.002365 | 0.997635 |
| ENST00000596289 | PLEKHD1 | 0.005058 | -0.99494 |
| ENST00000596289 | MLLT1 | 0.004973 | 0.995027 |
| ENST00000596289 | GPCPD1 | 0.004299 | -0.9957 |
| ENST00000596289 | STMND1 | 0.005219 | 0.994781 |
| ENST00000596289 | LYPD8 | 0.002365 | 0.997635 |
| TCONS_00018840 | CHSY3 | 0.007627 | -0.99237 |
| TCONS_00018840 | SIRT6 | 0.004352 | -0.99565 |
| TCONS_00018840 | MMP24 | 0.008937 | 0.991063 |
| ENST00000606528 | AHNAK2 | 0.002432 | 0.997568 |
| ENST00000606528 | NCCRP1 | 0.009715 | 0.990285 |
| ENST00000606528 | CHSY3 | 0.009916 | 0.990084 |
| ENST00000606528 | DUSP15 | 0.009001 | 0.990999 |
| ENST00000606528 | HOMER3 | 0.002609 | -0.99739 |
| ENST00000606528 | ARPC4-TTLL3 | 0.003599 | -0.9964 |
| ENST00000606528 | MAMLD1 | 0.009506 | -0.99049 |
| ENST00000606528 | CATG00000113928.1 | 0.006307 | -0.99369 |
| ENST00000606528 | ZNF214 | 0.008421 | -0.99158 |
| ENST00000606528 | MMP24 | 0.005667 | -0.99433 |
| ENST00000606528 | ZNF624 | 0.004058 | 0.995942 |
| ENST00000606528 | AHNAK2 | 0.002432 | 0.997568 |
| ENST00000606528 | NCCRP1 | 0.009715 | 0.990285 |
| ENST00000606528 | CHSY3 | 0.009916 | 0.990084 |
| ENST00000606528 | DUSP15 | 0.009001 | 0.990999 |
| ENST00000606528 | HOMER3 | 0.002609 | -0.99739 |
| ENST00000606528 | ARPC4-TTLL3 | 0.003599 | -0.9964 |
| ENST00000606528 | MAMLD1 | 0.009506 | -0.99049 |
| ENST00000606528 | CATG00000113928.1 | 0.006307 | -0.99369 |
| ENST00000606528 | ZNF214 | 0.008421 | -0.99158 |
| ENST00000606528 | MMP24 | 0.005667 | -0.99433 |
| ENST00000606528 | ZNF624 | 0.004058 | 0.995942 |
| ENST00000606528 | AHNAK2 | 0.002432 | 0.997568 |
| ENST00000606528 | NCCRP1 | 0.009715 | 0.990285 |
| ENST00000606528 | CHSY3 | 0.009916 | 0.990084 |
| ENST00000606528 | DUSP15 | 0.009001 | 0.990999 |
| ENST00000606528 | HOMER3 | 0.002609 | -0.99739 |
| ENST00000606528 | ARPC4-TTLL3 | 0.003599 | -0.9964 |
| ENST00000606528 | MAMLD1 | 0.009506 | -0.99049 |
| ENST00000606528 | CATG00000113928.1 | 0.006307 | -0.99369 |
| ENST00000606528 | ZNF214 | 0.008421 | -0.99158 |
| ENST00000606528 | MMP24 | 0.005667 | -0.99433 |
| ENST00000606528 | ZNF624 | 0.004058 | 0.995942 |
| ENST00000606528 | AHNAK2 | 0.002432 | 0.997568 |
| ENST00000606528 | NCCRP1 | 0.009715 | 0.990285 |
| ENST00000606528 | CHSY3 | 0.009916 | 0.990084 |
| ENST00000606528 | DUSP15 | 0.009001 | 0.990999 |
| ENST00000606528 | HOMER3 | 0.002609 | -0.99739 |
| ENST00000606528 | ARPC4-TTLL3 | 0.003599 | -0.9964 |
| ENST00000606528 | MAMLD1 | 0.009506 | -0.99049 |
| ENST00000606528 | CATG00000113928.1 | 0.006307 | -0.99369 |
| ENST00000606528 | ZNF214 | 0.008421 | -0.99158 |
| ENST00000606528 | MMP24 | 0.005667 | -0.99433 |
| ENST00000606528 | ZNF624 | 0.004058 | 0.995942 |
| ENST00000606528 | AHNAK2 | 0.002432 | 0.997568 |
| ENST00000606528 | NCCRP1 | 0.009715 | 0.990285 |
| ENST00000606528 | CHSY3 | 0.009916 | 0.990084 |
| ENST00000606528 | DUSP15 | 0.009001 | 0.990999 |
| ENST00000606528 | HOMER3 | 0.002609 | -0.99739 |
| ENST00000606528 | ARPC4-TTLL3 | 0.003599 | -0.9964 |
| ENST00000606528 | MAMLD1 | 0.009506 | -0.99049 |
| ENST00000606528 | CATG00000113928.1 | 0.006307 | -0.99369 |
| ENST00000606528 | ZNF214 | 0.008421 | -0.99158 |
| ENST00000606528 | MMP24 | 0.005667 | -0.99433 |
| ENST00000606528 | ZNF624 | 0.004058 | 0.995942 |
| ENST00000596088 | CCDC114 | 0.004865 | -0.99513 |
| ENST00000596088 | SPANXN4 | 0.006387 | -0.99361 |
| ENST00000596088 | C1orf198 | 0.006664 | -0.99334 |
| ENST00000596088 | LHFPL6 | 0.007843 | 0.992157 |
| ENST00000596088 | BVES | 0.004468 | 0.995532 |
| ENST00000596088 | COMMD2 | 0.002634 | -0.99737 |
| ENST00000596088 | RELL1 | 0.007603 | 0.992397 |
| ENST00000596088 | ANKS3 | 2.34E-04 | 0.999766 |
| ENST00000596088 | ATXN7 | 0.0011 | 0.9989 |
| ENST00000596088 | ASPRV1 | 3.08E-04 | -0.99969 |
| ENST00000596088 | CCDC114 | 0.004865 | -0.99513 |
| ENST00000596088 | SPANXN4 | 0.006387 | -0.99361 |
| ENST00000596088 | C1orf198 | 0.006664 | -0.99334 |
| ENST00000596088 | LHFPL6 | 0.007843 | 0.992157 |
| ENST00000596088 | BVES | 0.004468 | 0.995532 |
| ENST00000596088 | COMMD2 | 0.002634 | -0.99737 |
| ENST00000596088 | RELL1 | 0.007603 | 0.992397 |
| ENST00000596088 | ANKS3 | 2.34E-04 | 0.999766 |
| ENST00000596088 | ATXN7 | 0.0011 | 0.9989 |
| ENST00000596088 | ASPRV1 | 3.08E-04 | -0.99969 |
| ENST00000596088 | CCDC114 | 0.004865 | -0.99513 |
| ENST00000596088 | SPANXN4 | 0.006387 | -0.99361 |
| ENST00000596088 | C1orf198 | 0.006664 | -0.99334 |
| ENST00000596088 | LHFPL6 | 0.007843 | 0.992157 |
| ENST00000596088 | BVES | 0.004468 | 0.995532 |
| ENST00000596088 | COMMD2 | 0.002634 | -0.99737 |
| ENST00000596088 | RELL1 | 0.007603 | 0.992397 |
| ENST00000596088 | ANKS3 | 2.34E-04 | 0.999766 |
| ENST00000596088 | ATXN7 | 0.0011 | 0.9989 |
| ENST00000596088 | ASPRV1 | 3.08E-04 | -0.99969 |
| ENST00000584273 | ZFY | 0.00487 | -0.99513 |
| ENST00000584273 | EXOC3L2 | 0.009311 | 0.990689 |
| ENST00000584273 | PLD3 | 0.003584 | -0.99642 |
| ENST00000584273 | RAB8A | 0.004391 | -0.99561 |
| ENST00000584273 | SSX1 | 0.002864 | 0.997136 |
| ENST00000584273 | WSCD2 | 9.83E-05 | 0.999902 |
| ENST00000584273 | GRIN3B | 0.002574 | -0.99743 |
| ENST00000584273 | TNFAIP6 | 0.009422 | -0.99058 |
| ENST00000584273 | PTPRK | 0.004333 | 0.995667 |
| ENST00000584273 | CATG00000039609.1 | 0.008577 | 0.991423 |
| ENST00000584273 | DEFA4 | 0.008165 | 0.991835 |
| FTMT23600008245 | CATG00000021869.1 | 0.005232 | 0.994768 |
| FTMT23600008245 | KCNJ11 | 0.0028 | 0.9972 |
| FTMT23600008245 | TSPAN32 | 0.006608 | -0.99339 |
| FTMT23600008245 | NHEJ1 | 0.005347 | -0.99465 |
| FTMT23600008245 | METTL3 | 0.001557 | 0.998443 |
| FTMT23600008245 | C1QTNF9B | 0.009002 | 0.990998 |
| FTMT23600008245 | USF2 | 0.008284 | 0.991716 |
| FTMT23600008245 | UPP2 | 0.008564 | 0.991436 |
| FTMT23600008245 | CATG00000021869.1 | 0.005232 | 0.994768 |
| FTMT23600008245 | KCNJ11 | 0.0028 | 0.9972 |
| FTMT23600008245 | TSPAN32 | 0.006608 | -0.99339 |
| FTMT23600008245 | NHEJ1 | 0.005347 | -0.99465 |
| FTMT23600008245 | METTL3 | 0.001557 | 0.998443 |
| FTMT23600008245 | C1QTNF9B | 0.009002 | 0.990998 |
| FTMT23600008245 | USF2 | 0.008284 | 0.991716 |
| FTMT23600008245 | UPP2 | 0.008564 | 0.991436 |
| FTMT23600008245 | CATG00000021869.1 | 0.005232 | 0.994768 |
| FTMT23600008245 | KCNJ11 | 0.0028 | 0.9972 |
| FTMT23600008245 | TSPAN32 | 0.006608 | -0.99339 |
| FTMT23600008245 | NHEJ1 | 0.005347 | -0.99465 |
| FTMT23600008245 | METTL3 | 0.001557 | 0.998443 |
| FTMT23600008245 | C1QTNF9B | 0.009002 | 0.990998 |
| FTMT23600008245 | USF2 | 0.008284 | 0.991716 |
| FTMT23600008245 | UPP2 | 0.008564 | 0.991436 |
| FTMT23600008245 | CATG00000021869.1 | 0.005232 | 0.994768 |
| FTMT23600008245 | KCNJ11 | 0.0028 | 0.9972 |
| FTMT23600008245 | TSPAN32 | 0.006608 | -0.99339 |
| FTMT23600008245 | NHEJ1 | 0.005347 | -0.99465 |
| FTMT23600008245 | METTL3 | 0.001557 | 0.998443 |
| FTMT23600008245 | C1QTNF9B | 0.009002 | 0.990998 |
| FTMT23600008245 | USF2 | 0.008284 | 0.991716 |
| FTMT23600008245 | UPP2 | 0.008564 | 0.991436 |
| FTMT23600008245 | CATG00000021869.1 | 0.005232 | 0.994768 |
| FTMT23600008245 | KCNJ11 | 0.0028 | 0.9972 |
| FTMT23600008245 | TSPAN32 | 0.006608 | -0.99339 |
| FTMT23600008245 | NHEJ1 | 0.005347 | -0.99465 |
| FTMT23600008245 | METTL3 | 0.001557 | 0.998443 |
| FTMT23600008245 | C1QTNF9B | 0.009002 | 0.990998 |
| FTMT23600008245 | USF2 | 0.008284 | 0.991716 |
| FTMT23600008245 | UPP2 | 0.008564 | 0.991436 |
| FTMT23600008245 | CATG00000021869.1 | 0.005232 | 0.994768 |
| FTMT23600008245 | KCNJ11 | 0.0028 | 0.9972 |
| FTMT23600008245 | TSPAN32 | 0.006608 | -0.99339 |
| FTMT23600008245 | NHEJ1 | 0.005347 | -0.99465 |
| FTMT23600008245 | METTL3 | 0.001557 | 0.998443 |
| FTMT23600008245 | C1QTNF9B | 0.009002 | 0.990998 |
| FTMT23600008245 | USF2 | 0.008284 | 0.991716 |
| FTMT23600008245 | UPP2 | 0.008564 | 0.991436 |
| ENST00000561912 | NOC4L | 0.006501 | 0.993499 |
| ENST00000561912 | CATG00000080699.1 | 0.002301 | 0.997699 |
| ENST00000561912 | SLC24A5 | 0.009133 | -0.99087 |
| ENST00000561912 | TCOF1 | 0.004763 | 0.995237 |
| ENST00000561912 | SAMD13 | 0.005253 | 0.994747 |
| ENST00000561912 | ZDHHC16 | 2.84E-04 | -0.99972 |
| ENST00000561912 | CDKL2 | 0.008566 | 0.991434 |
| ENST00000561912 | MACROD2 | 0.001153 | 0.998847 |
| ENST00000561912 | CHST12 | 0 | 1 |
| ENST00000561912 | HIPK2 | 0.004794 | -0.99521 |
| ENST00000561912 | PCDHB8 | 0 | 1 |
| ENST00000561912 | NOC4L | 0.006501 | 0.993499 |
| ENST00000561912 | CATG00000080699.1 | 0.002301 | 0.997699 |
| ENST00000561912 | SLC24A5 | 0.009133 | -0.99087 |
| ENST00000561912 | TCOF1 | 0.004763 | 0.995237 |
| ENST00000561912 | SAMD13 | 0.005253 | 0.994747 |
| ENST00000561912 | ZDHHC16 | 2.84E-04 | -0.99972 |
| ENST00000561912 | CDKL2 | 0.008566 | 0.991434 |
| ENST00000561912 | MACROD2 | 0.001153 | 0.998847 |
| ENST00000561912 | CHST12 | 0 | 1 |
| ENST00000561912 | HIPK2 | 0.004794 | -0.99521 |
| ENST00000561912 | PCDHB8 | 0 | 1 |
| ENST00000561912 | NOC4L | 0.006501 | 0.993499 |
| ENST00000561912 | CATG00000080699.1 | 0.002301 | 0.997699 |
| ENST00000561912 | SLC24A5 | 0.009133 | -0.99087 |
| ENST00000561912 | TCOF1 | 0.004763 | 0.995237 |
| ENST00000561912 | SAMD13 | 0.005253 | 0.994747 |
| ENST00000561912 | ZDHHC16 | 2.84E-04 | -0.99972 |
| ENST00000561912 | CDKL2 | 0.008566 | 0.991434 |
| ENST00000561912 | MACROD2 | 0.001153 | 0.998847 |
| ENST00000561912 | CHST12 | 0 | 1 |
| ENST00000561912 | HIPK2 | 0.004794 | -0.99521 |
| ENST00000561912 | PCDHB8 | 0 | 1 |
| ENST00000561912 | NOC4L | 0.006501 | 0.993499 |
| ENST00000561912 | CATG00000080699.1 | 0.002301 | 0.997699 |
| ENST00000561912 | SLC24A5 | 0.009133 | -0.99087 |
| ENST00000561912 | TCOF1 | 0.004763 | 0.995237 |
| ENST00000561912 | SAMD13 | 0.005253 | 0.994747 |
| ENST00000561912 | ZDHHC16 | 2.84E-04 | -0.99972 |
| ENST00000561912 | CDKL2 | 0.008566 | 0.991434 |
| ENST00000561912 | MACROD2 | 0.001153 | 0.998847 |
| ENST00000561912 | CHST12 | 0 | 1 |
| ENST00000561912 | HIPK2 | 0.004794 | -0.99521 |
| ENST00000561912 | PCDHB8 | 0 | 1 |
| ENST00000608197 | NR2F2 | 0.003727 | -0.99627 |
| ENST00000608197 | RPL23A | 0.006121 | 0.993879 |
| ENST00000608197 | SMPD1 | 0.006987 | -0.99301 |
| ENST00000608197 | DHODH | 0.001137 | 0.998863 |
| ENST00000608197 | PRPF8 | 0.002221 | 0.997779 |
| ENST00000608197 | CCDC149 | 0.002877 | -0.99712 |
| ENST00000608197 | CHCHD6 | 0.008124 | -0.99188 |
| ENST00000608197 | TMEM51 | 0.004603 | -0.9954 |
| ENST00000608197 | CEP290 | 0.001571 | -0.99843 |
| ENST00000608197 | PCNX3 | 0.001723 | 0.998277 |
| ENST00000608197 | WNT2 | 0.009749 | -0.99025 |
| ENST00000608197 | TCTEX1D4 | 0.006018 | 0.993982 |
| ENST00000608197 | TIGD3 | 8.20E-06 | 0.999992 |
| ENST00000608197 | LBP | 0.009871 | -0.99013 |
| ENST00000608197 | ZNF107 | 0.001231 | 0.998769 |
| ENST00000608197 | CEP63 | 0.008524 | 0.991476 |
| ENST00000608197 | ACMSD | 0.003824 | 0.996176 |
| ENST00000608197 | NR2F2 | 0.003727 | -0.99627 |
| ENST00000608197 | RPL23A | 0.006121 | 0.993879 |
| ENST00000608197 | SMPD1 | 0.006987 | -0.99301 |
| ENST00000608197 | DHODH | 0.001137 | 0.998863 |
| ENST00000608197 | PRPF8 | 0.002221 | 0.997779 |
| ENST00000608197 | CCDC149 | 0.002877 | -0.99712 |
| ENST00000608197 | CHCHD6 | 0.008124 | -0.99188 |
| ENST00000608197 | TMEM51 | 0.004603 | -0.9954 |
| ENST00000608197 | CEP290 | 0.001571 | -0.99843 |
| ENST00000608197 | PCNX3 | 0.001723 | 0.998277 |
| ENST00000608197 | WNT2 | 0.009749 | -0.99025 |
| ENST00000608197 | TCTEX1D4 | 0.006018 | 0.993982 |
| ENST00000608197 | TIGD3 | 8.20E-06 | 0.999992 |
| ENST00000608197 | LBP | 0.009871 | -0.99013 |
| ENST00000608197 | ZNF107 | 0.001231 | 0.998769 |
| ENST00000608197 | CEP63 | 0.008524 | 0.991476 |
| ENST00000608197 | ACMSD | 0.003824 | 0.996176 |
| ENST00000608197 | NR2F2 | 0.003727 | -0.99627 |
| ENST00000608197 | RPL23A | 0.006121 | 0.993879 |
| ENST00000608197 | SMPD1 | 0.006987 | -0.99301 |
| ENST00000608197 | DHODH | 0.001137 | 0.998863 |
| ENST00000608197 | PRPF8 | 0.002221 | 0.997779 |
| ENST00000608197 | CCDC149 | 0.002877 | -0.99712 |
| ENST00000608197 | CHCHD6 | 0.008124 | -0.99188 |
| ENST00000608197 | TMEM51 | 0.004603 | -0.9954 |
| ENST00000608197 | CEP290 | 0.001571 | -0.99843 |
| ENST00000608197 | PCNX3 | 0.001723 | 0.998277 |
| ENST00000608197 | WNT2 | 0.009749 | -0.99025 |
| ENST00000608197 | TCTEX1D4 | 0.006018 | 0.993982 |
| ENST00000608197 | TIGD3 | 8.20E-06 | 0.999992 |
| ENST00000608197 | LBP | 0.009871 | -0.99013 |
| ENST00000608197 | ZNF107 | 0.001231 | 0.998769 |
| ENST00000608197 | CEP63 | 0.008524 | 0.991476 |
| ENST00000608197 | ACMSD | 0.003824 | 0.996176 |
| ENST00000424421 | PTGER1 | 0.008559 | 0.991441 |
| ENST00000424421 | CMYA5 | 0.003452 | -0.99655 |
| ENST00000424421 | TMEM230 | 0.001242 | -0.99876 |
| ENST00000424421 | EEF1B2 | 0.00325 | 0.99675 |
| ENST00000424421 | KIAA1211L | 0.006379 | 0.993621 |
| ENST00000424421 | TNFAIP6 | 0.005327 | -0.99467 |
| ENST00000424421 | PTGER1 | 0.008559 | 0.991441 |
| ENST00000424421 | CMYA5 | 0.003452 | -0.99655 |
| ENST00000424421 | TMEM230 | 0.001242 | -0.99876 |
| ENST00000424421 | EEF1B2 | 0.00325 | 0.99675 |
| ENST00000424421 | KIAA1211L | 0.006379 | 0.993621 |
| ENST00000424421 | TNFAIP6 | 0.005327 | -0.99467 |
| ENST00000424421 | PTGER1 | 0.008559 | 0.991441 |
| ENST00000424421 | CMYA5 | 0.003452 | -0.99655 |
| ENST00000424421 | TMEM230 | 0.001242 | -0.99876 |
| ENST00000424421 | EEF1B2 | 0.00325 | 0.99675 |
| ENST00000424421 | KIAA1211L | 0.006379 | 0.993621 |
| ENST00000424421 | TNFAIP6 | 0.005327 | -0.99467 |
| T227479 | BARHL1 | 0.006015 | -0.99398 |
| T227479 | B4GALNT1 | 0.001656 | -0.99834 |
| T227479 | ZFY | 0.009277 | -0.99072 |
| T227479 | S1PR5 | 7.11E-04 | -0.99929 |
| T227479 | RP1L1 | 0.007387 | -0.99261 |
| T227479 | DEFA1 | 0.004817 | 0.995183 |
| T227479 | GRIN3B | 0.007372 | -0.99263 |
| T227479 | PLEKHG3 | 0.004226 | -0.99577 |
| T227479 | DEFA4 | 0.001734 | 0.998266 |
| T227479 | BARHL1 | 0.006015 | -0.99398 |
| T227479 | B4GALNT1 | 0.001656 | -0.99834 |
| T227479 | ZFY | 0.009277 | -0.99072 |
| T227479 | S1PR5 | 7.11E-04 | -0.99929 |
| T227479 | RP1L1 | 0.007387 | -0.99261 |
| T227479 | DEFA1 | 0.004817 | 0.995183 |
| T227479 | GRIN3B | 0.007372 | -0.99263 |
| T227479 | PLEKHG3 | 0.004226 | -0.99577 |
| T227479 | DEFA4 | 0.001734 | 0.998266 |
| T227479 | BARHL1 | 0.006015 | -0.99398 |
| T227479 | B4GALNT1 | 0.001656 | -0.99834 |
| T227479 | ZFY | 0.009277 | -0.99072 |
| T227479 | S1PR5 | 7.11E-04 | -0.99929 |
| T227479 | RP1L1 | 0.007387 | -0.99261 |
| T227479 | DEFA1 | 0.004817 | 0.995183 |
| T227479 | GRIN3B | 0.007372 | -0.99263 |
| T227479 | PLEKHG3 | 0.004226 | -0.99577 |
| T227479 | DEFA4 | 0.001734 | 0.998266 |
| T227479 | BARHL1 | 0.006015 | -0.99398 |
| T227479 | B4GALNT1 | 0.001656 | -0.99834 |
| T227479 | ZFY | 0.009277 | -0.99072 |
| T227479 | S1PR5 | 7.11E-04 | -0.99929 |
| T227479 | RP1L1 | 0.007387 | -0.99261 |
| T227479 | DEFA1 | 0.004817 | 0.995183 |
| T227479 | GRIN3B | 0.007372 | -0.99263 |
| T227479 | PLEKHG3 | 0.004226 | -0.99577 |
| T227479 | DEFA4 | 0.001734 | 0.998266 |
| ENST00000592125 | INTS2 | 0.002892 | -0.99711 |
| ENST00000592125 | CEP170 | 0.001596 | -0.9984 |
| ENST00000592125 | RFC2 | 0.006167 | -0.99383 |
| ENST00000592125 | EXOC4 | 0.004029 | 0.995971 |
| ENST00000592125 | ADGRE1 | 0.005677 | -0.99432 |
| ENST00000592125 | CDIP1 | 0.001348 | -0.99865 |
| ENST00000592125 | SH3D21 | 0.009131 | 0.990869 |
| ENST00000592125 | DBI | 0.006389 | -0.99361 |
| ENST00000592125 | TP73 | 0.003724 | 0.996276 |
| ENST00000592125 | SEC14L6 | 0.007277 | 0.992723 |
| ENST00000592125 | NEMP2 | 0.00161 | 0.99839 |
| ENST00000592125 | NEFM | 0.003471 | -0.99653 |
| ENST00000592125 | CATG00000074344.1 | 0.004405 | 0.995595 |
| ENST00000592125 | DEFA6 | 0.002614 | 0.997386 |
| ENST00000592125 | CATG00000027020.1 | 1.33E-04 | 0.999867 |
| ENST00000432148 | GNL1 | 0.001802 | 0.998198 |
| ENST00000432148 | DEAF1 | 0.007874 | -0.99213 |
| ENST00000432148 | DNAJA4 | 0.004853 | -0.99515 |
| ENST00000432148 | GPR25 | 2.18E-04 | -0.99978 |
| ENST00000432148 | FANCD2OS | 0.006216 | -0.99378 |
| ENST00000432148 | SHC2 | 0.005807 | -0.99419 |
| ENST00000432148 | EFEMP2 | 0.001787 | 0.998213 |
| ENST00000432148 | OR4X1 | 0.005227 | -0.99477 |
| ENST00000432148 | HLA-F | 0.002101 | -0.9979 |
| T002642 | FAM84A | 0.004892 | -0.99511 |
| T002642 | C20orf203 | 0.00346 | -0.99654 |
| T002642 | DUSP15 | 9.57E-04 | -0.99904 |
| T002642 | AASDHPPT | 5.03E-04 | -0.9995 |
| T002642 | G6PC3 | 0.002647 | 0.997353 |
| T002642 | RTN4 | 0.004157 | 0.995843 |
| T002642 | FAM110C | 0.006417 | 0.993583 |
| T002642 | ZNF624 | 0.007247 | -0.99275 |
| T002642 | OR5D18 | 1.59E-04 | 0.999841 |
| T002642 | FAM84A | 0.004892 | -0.99511 |
| T002642 | C20orf203 | 0.00346 | -0.99654 |
| T002642 | DUSP15 | 9.57E-04 | -0.99904 |
| T002642 | AASDHPPT | 5.03E-04 | -0.9995 |
| T002642 | G6PC3 | 0.002647 | 0.997353 |
| T002642 | RTN4 | 0.004157 | 0.995843 |
| T002642 | FAM110C | 0.006417 | 0.993583 |
| T002642 | ZNF624 | 0.007247 | -0.99275 |
| T002642 | OR5D18 | 1.59E-04 | 0.999841 |
| ENST00000548497 | CATG00000061038.1 | 0.002442 | 0.997558 |
| ENST00000548497 | MAPKBP1 | 0.005719 | -0.99428 |
| ENST00000548497 | AC093157.1 | 0.007803 | 0.992197 |
| ENST00000548497 | CATG00000026669.1 | 0.003381 | 0.996619 |
| ENST00000548497 | OR10R2 | 0.006561 | -0.99344 |
| ENST00000548497 | DDIT4 | 0.005775 | 0.994225 |
| ENST00000548497 | CARNS1 | 0.003486 | 0.996514 |
| ENST00000432735 | ANXA3 | 0.00303 | -0.99697 |
| ENST00000432735 | CHIC2 | 0.002108 | 0.997892 |
| ENST00000432735 | RNF149 | 0.006432 | 0.993568 |
| ENST00000432735 | ANXA3 | 0.00303 | -0.99697 |
| ENST00000432735 | CHIC2 | 0.002108 | 0.997892 |
| ENST00000432735 | RNF149 | 0.006432 | 0.993568 |
| ENST00000432735 | ANXA3 | 0.00303 | -0.99697 |
| ENST00000432735 | CHIC2 | 0.002108 | 0.997892 |
| ENST00000432735 | RNF149 | 0.006432 | 0.993568 |
| ENST00000432735 | ANXA3 | 0.00303 | -0.99697 |
| ENST00000432735 | CHIC2 | 0.002108 | 0.997892 |
| ENST00000432735 | RNF149 | 0.006432 | 0.993568 |
| ENST00000564259 | TNFRSF18 | 3.67E-04 | 0.999633 |
| ENST00000564259 | SPATA33 | 7.62E-04 | 0.999238 |
| ENST00000564259 | KLHL35 | 0.002909 | 0.997091 |
| ENST00000564259 | MT1F | 0.006001 | 0.993999 |
| ENST00000564259 | WWP1 | 0.004815 | 0.995185 |
| ENST00000564259 | APOBR | 0.00648 | 0.99352 |
| ENST00000564259 | TNXB | 0.002256 | -0.99774 |
| ENST00000564259 | KYAT3 | 0.004437 | -0.99556 |
| ENST00000564259 | HEATR9 | 0.003117 | -0.99688 |
| ENST00000564259 | PAQR5 | 0.004859 | -0.99514 |
| ENST00000564259 | TNRC6B | 0.001939 | -0.99806 |
| ENST00000564259 | ATG4C | 0.008108 | -0.99189 |
| ENST00000564259 | TNFRSF18 | 3.67E-04 | 0.999633 |
| ENST00000564259 | SPATA33 | 7.62E-04 | 0.999238 |
| ENST00000564259 | KLHL35 | 0.002909 | 0.997091 |
| ENST00000564259 | MT1F | 0.006001 | 0.993999 |
| ENST00000564259 | WWP1 | 0.004815 | 0.995185 |
| ENST00000564259 | APOBR | 0.00648 | 0.99352 |
| ENST00000564259 | TNXB | 0.002256 | -0.99774 |
| ENST00000564259 | KYAT3 | 0.004437 | -0.99556 |
| ENST00000564259 | HEATR9 | 0.003117 | -0.99688 |
| ENST00000564259 | PAQR5 | 0.004859 | -0.99514 |
| ENST00000564259 | TNRC6B | 0.001939 | -0.99806 |
| ENST00000564259 | ATG4C | 0.008108 | -0.99189 |
| ENST00000564259 | TNFRSF18 | 3.67E-04 | 0.999633 |
| ENST00000564259 | SPATA33 | 7.62E-04 | 0.999238 |
| ENST00000564259 | KLHL35 | 0.002909 | 0.997091 |
| ENST00000564259 | MT1F | 0.006001 | 0.993999 |
| ENST00000564259 | WWP1 | 0.004815 | 0.995185 |
| ENST00000564259 | APOBR | 0.00648 | 0.99352 |
| ENST00000564259 | TNXB | 0.002256 | -0.99774 |
| ENST00000564259 | KYAT3 | 0.004437 | -0.99556 |
| ENST00000564259 | HEATR9 | 0.003117 | -0.99688 |
| ENST00000564259 | PAQR5 | 0.004859 | -0.99514 |
| ENST00000564259 | TNRC6B | 0.001939 | -0.99806 |
| ENST00000564259 | ATG4C | 0.008108 | -0.99189 |
| ENST00000564259 | TNFRSF18 | 3.67E-04 | 0.999633 |
| ENST00000564259 | SPATA33 | 7.62E-04 | 0.999238 |
| ENST00000564259 | KLHL35 | 0.002909 | 0.997091 |
| ENST00000564259 | MT1F | 0.006001 | 0.993999 |
| ENST00000564259 | WWP1 | 0.004815 | 0.995185 |
| ENST00000564259 | APOBR | 0.00648 | 0.99352 |
| ENST00000564259 | TNXB | 0.002256 | -0.99774 |
| ENST00000564259 | KYAT3 | 0.004437 | -0.99556 |
| ENST00000564259 | HEATR9 | 0.003117 | -0.99688 |
| ENST00000564259 | PAQR5 | 0.004859 | -0.99514 |
| ENST00000564259 | TNRC6B | 0.001939 | -0.99806 |
| ENST00000564259 | ATG4C | 0.008108 | -0.99189 |
| ENST00000564259 | TNFRSF18 | 3.67E-04 | 0.999633 |
| ENST00000564259 | SPATA33 | 7.62E-04 | 0.999238 |
| ENST00000564259 | KLHL35 | 0.002909 | 0.997091 |
| ENST00000564259 | MT1F | 0.006001 | 0.993999 |
| ENST00000564259 | WWP1 | 0.004815 | 0.995185 |
| ENST00000564259 | APOBR | 0.00648 | 0.99352 |
| ENST00000564259 | TNXB | 0.002256 | -0.99774 |
| ENST00000564259 | KYAT3 | 0.004437 | -0.99556 |
| ENST00000564259 | HEATR9 | 0.003117 | -0.99688 |
| ENST00000564259 | PAQR5 | 0.004859 | -0.99514 |
| ENST00000564259 | TNRC6B | 0.001939 | -0.99806 |
| ENST00000564259 | ATG4C | 0.008108 | -0.99189 |
| ENST00000564259 | TNFRSF18 | 3.67E-04 | 0.999633 |
| ENST00000564259 | SPATA33 | 7.62E-04 | 0.999238 |
| ENST00000564259 | KLHL35 | 0.002909 | 0.997091 |
| ENST00000564259 | MT1F | 0.006001 | 0.993999 |
| ENST00000564259 | WWP1 | 0.004815 | 0.995185 |
| ENST00000564259 | APOBR | 0.00648 | 0.99352 |
| ENST00000564259 | TNXB | 0.002256 | -0.99774 |
| ENST00000564259 | KYAT3 | 0.004437 | -0.99556 |
| ENST00000564259 | HEATR9 | 0.003117 | -0.99688 |
| ENST00000564259 | PAQR5 | 0.004859 | -0.99514 |
| ENST00000564259 | TNRC6B | 0.001939 | -0.99806 |
| ENST00000564259 | ATG4C | 0.008108 | -0.99189 |
| ENST00000564259 | TNFRSF18 | 3.67E-04 | 0.999633 |
| ENST00000564259 | SPATA33 | 7.62E-04 | 0.999238 |
| ENST00000564259 | KLHL35 | 0.002909 | 0.997091 |
| ENST00000564259 | MT1F | 0.006001 | 0.993999 |
| ENST00000564259 | WWP1 | 0.004815 | 0.995185 |
| ENST00000564259 | APOBR | 0.00648 | 0.99352 |
| ENST00000564259 | TNXB | 0.002256 | -0.99774 |
| ENST00000564259 | KYAT3 | 0.004437 | -0.99556 |
| ENST00000564259 | HEATR9 | 0.003117 | -0.99688 |
| ENST00000564259 | PAQR5 | 0.004859 | -0.99514 |
| ENST00000564259 | TNRC6B | 0.001939 | -0.99806 |
| ENST00000564259 | ATG4C | 0.008108 | -0.99189 |
| HSALNT0289206 | NOC4L | 0.006501 | 0.993499 |
| HSALNT0289206 | CATG00000080699.1 | 0.002301 | 0.997699 |
| HSALNT0289206 | SLC24A5 | 0.009133 | -0.99087 |
| HSALNT0289206 | TCOF1 | 0.004763 | 0.995237 |
| HSALNT0289206 | SAMD13 | 0.005253 | 0.994747 |
| HSALNT0289206 | ZDHHC16 | 2.84E-04 | -0.99972 |
| HSALNT0289206 | CDKL2 | 0.008566 | 0.991434 |
| HSALNT0289206 | MACROD2 | 0.001153 | 0.998847 |
| HSALNT0289206 | CHST12 | 0 | 1 |
| HSALNT0289206 | HIPK2 | 0.004794 | -0.99521 |
| HSALNT0289206 | PCDHB8 | 0 | 1 |
| ENST00000426083 | ZDBF2 | 0.005392 | 0.994608 |
| ENST00000426083 | CATG00000029374.1 | 0.00774 | 0.99226 |
| ENST00000426083 | ZDBF2 | 0.005392 | 0.994608 |
| ENST00000426083 | CATG00000029374.1 | 0.00774 | 0.99226 |
| ENST00000426083 | ZDBF2 | 0.005392 | 0.994608 |
| ENST00000426083 | CATG00000029374.1 | 0.00774 | 0.99226 |
| ENST00000471993 | SCAMP2 | 0.007037 | -0.99296 |
| ENST00000471993 | SLC24A5 | 0.003862 | 0.996138 |
| ENST00000471993 | TCOF1 | 0.007859 | -0.99214 |
| ENST00000471993 | TMC5 | 0.007902 | -0.9921 |
| ENST00000471993 | XYLT2 | 0.009705 | -0.9903 |
| ENST00000471993 | FSCB | 0.004147 | -0.99585 |
| ENST00000471993 | SAMD13 | 0.00778 | -0.99222 |
| ENST00000471993 | IKZF4 | 0.001008 | -0.99899 |
| ENST00000471993 | MYH3 | 0.003362 | 0.996638 |
| ENST00000471993 | KCTD1 | 0.007919 | -0.99208 |
| ENST00000471993 | PURG | 0.001854 | -0.99815 |
| ENST00000471993 | CACNG6 | 0.002945 | -0.99706 |
| ENST00000471993 | PAN3 | 0.00544 | 0.99456 |
| ENST00000471993 | SHROOM3 | 0.009045 | -0.99096 |
| ENST00000471993 | INKA2 | 0.005915 | -0.99409 |
| ENST00000471993 | RAMP1 | 0.006291 | 0.993709 |
| ENST00000471993 | PARVG | 0.004609 | -0.99539 |
| ENST00000471993 | ABCA13 | 0.009404 | -0.9906 |
| ENST00000471993 | YPEL5 | 4.80E-04 | -0.99952 |
| ENST00000600155 | BECN1 | 0.003029 | -0.99697 |
| ENST00000600155 | ACTL7B | 5.93E-04 | 0.999407 |
| ENST00000600155 | C15orf65 | 0.007985 | 0.992015 |
| ENST00000600155 | NDUFAF8 | 0.001564 | -0.99844 |
| ENST00000600155 | MED12L | 0.001863 | 0.998137 |
| ENST00000600155 | PMEPA1 | 0.005407 | 0.994593 |
| ENST00000600155 | FCRL1 | 0.009178 | 0.990822 |
| ENCT00000424714 | BECN1 | 0.006284 | -0.99372 |
| ENCT00000424714 | ACTL7B | 0.001625 | 0.998375 |
| ENCT00000424714 | NDUFAF8 | 0.00645 | -0.99355 |
| ENCT00000424714 | FBXL16 | 0.006908 | -0.99309 |
| ENCT00000424714 | MED12L | 5.63E-07 | 0.999999 |
| uc003wjz.1 | LDLRAD4 | 0.001814 | 0.998186 |
| uc003wjz.1 | GSTM5 | 0.003518 | -0.99648 |
| uc003wjz.1 | DLEC1 | 0.008071 | -0.99193 |
| uc003wjz.1 | LDLRAD4 | 0.001814 | 0.998186 |
| uc003wjz.1 | GSTM5 | 0.003518 | -0.99648 |
| uc003wjz.1 | DLEC1 | 0.008071 | -0.99193 |
| uc003wjz.1 | LDLRAD4 | 0.001814 | 0.998186 |
| uc003wjz.1 | GSTM5 | 0.003518 | -0.99648 |
| uc003wjz.1 | DLEC1 | 0.008071 | -0.99193 |
| TCONS_00004332 | SPTY2D1OS | 0.007376 | -0.99262 |
| TCONS_00004332 | KYAT1 | 0.00941 | -0.99059 |
| TCONS_00004332 | LYL1 | 0.005635 | -0.99436 |
| TCONS_00004332 | STEAP3 | 0.002413 | -0.99759 |
| TCONS_00004332 | SLC13A4 | 0.002124 | -0.99788 |
| TCONS_00004332 | HIC1 | 0.00743 | -0.99257 |
| TCONS_00004332 | TFF2 | 0.001638 | -0.99836 |
| TCONS_00004332 | ORC3 | 0.003674 | -0.99633 |
| TCONS_00004332 | UGT2B28 | 0.003466 | -0.99653 |
| T376751 | BECN1 | 0.006546 | -0.99345 |
| T376751 | ACTL7B | 0.001774 | 0.998226 |
| T376751 | NDUFAF8 | 0.006795 | -0.9932 |
| T376751 | FBXL16 | 0.00665 | -0.99335 |
| T376751 | MED12L | 2.00E-06 | 0.999998 |
| ENST00000484721 | BTBD2 | 0.005197 | 0.994803 |
| ENST00000484721 | UBB | 2.43E-04 | 0.999757 |
| ENST00000484721 | PXDC1 | 0.002602 | 0.997398 |
| ENST00000484721 | EVC2 | 3.09E-04 | 0.999691 |
| ENST00000484721 | TMOD1 | 0.001296 | 0.998704 |
| ENST00000484721 | MYCN | 9.70E-04 | 0.99903 |
| ENST00000484721 | HMOX2 | 0.00607 | 0.99393 |
| ENST00000484721 | CCNK | 0.008169 | 0.991831 |
| ENST00000484721 | UBN2 | 0.009761 | -0.99024 |
| ENST00000484721 | CATG00000020284.1 | 0.008115 | -0.99188 |
| ENST00000484721 | BTBD2 | 0.005197 | 0.994803 |
| ENST00000484721 | UBB | 2.43E-04 | 0.999757 |
| ENST00000484721 | PXDC1 | 0.002602 | 0.997398 |
| ENST00000484721 | EVC2 | 3.09E-04 | 0.999691 |
| ENST00000484721 | TMOD1 | 0.001296 | 0.998704 |
| ENST00000484721 | MYCN | 9.70E-04 | 0.99903 |
| ENST00000484721 | HMOX2 | 0.00607 | 0.99393 |
| ENST00000484721 | CCNK | 0.008169 | 0.991831 |
| ENST00000484721 | UBN2 | 0.009761 | -0.99024 |
| ENST00000484721 | CATG00000020284.1 | 0.008115 | -0.99188 |
| ENST00000484721 | BTBD2 | 0.005197 | 0.994803 |
| ENST00000484721 | UBB | 2.43E-04 | 0.999757 |
| ENST00000484721 | PXDC1 | 0.002602 | 0.997398 |
| ENST00000484721 | EVC2 | 3.09E-04 | 0.999691 |
| ENST00000484721 | TMOD1 | 0.001296 | 0.998704 |
| ENST00000484721 | MYCN | 9.70E-04 | 0.99903 |
| ENST00000484721 | HMOX2 | 0.00607 | 0.99393 |
| ENST00000484721 | CCNK | 0.008169 | 0.991831 |
| ENST00000484721 | UBN2 | 0.009761 | -0.99024 |
| ENST00000484721 | CATG00000020284.1 | 0.008115 | -0.99188 |
| ENST00000484721 | BTBD2 | 0.005197 | 0.994803 |
| ENST00000484721 | UBB | 2.43E-04 | 0.999757 |
| ENST00000484721 | PXDC1 | 0.002602 | 0.997398 |
| ENST00000484721 | EVC2 | 3.09E-04 | 0.999691 |
| ENST00000484721 | TMOD1 | 0.001296 | 0.998704 |
| ENST00000484721 | MYCN | 9.70E-04 | 0.99903 |
| ENST00000484721 | HMOX2 | 0.00607 | 0.99393 |
| ENST00000484721 | CCNK | 0.008169 | 0.991831 |
| ENST00000484721 | UBN2 | 0.009761 | -0.99024 |
| ENST00000484721 | CATG00000020284.1 | 0.008115 | -0.99188 |
| ENST00000484721 | BTBD2 | 0.005197 | 0.994803 |
| ENST00000484721 | UBB | 2.43E-04 | 0.999757 |
| ENST00000484721 | PXDC1 | 0.002602 | 0.997398 |
| ENST00000484721 | EVC2 | 3.09E-04 | 0.999691 |
| ENST00000484721 | TMOD1 | 0.001296 | 0.998704 |
| ENST00000484721 | MYCN | 9.70E-04 | 0.99903 |
| ENST00000484721 | HMOX2 | 0.00607 | 0.99393 |
| ENST00000484721 | CCNK | 0.008169 | 0.991831 |
| ENST00000484721 | UBN2 | 0.009761 | -0.99024 |
| ENST00000484721 | CATG00000020284.1 | 0.008115 | -0.99188 |
| ENST00000484721 | BTBD2 | 0.005197 | 0.994803 |
| ENST00000484721 | UBB | 2.43E-04 | 0.999757 |
| ENST00000484721 | PXDC1 | 0.002602 | 0.997398 |
| ENST00000484721 | EVC2 | 3.09E-04 | 0.999691 |
| ENST00000484721 | TMOD1 | 0.001296 | 0.998704 |
| ENST00000484721 | MYCN | 9.70E-04 | 0.99903 |
| ENST00000484721 | HMOX2 | 0.00607 | 0.99393 |
| ENST00000484721 | CCNK | 0.008169 | 0.991831 |
| ENST00000484721 | UBN2 | 0.009761 | -0.99024 |
| ENST00000484721 | CATG00000020284.1 | 0.008115 | -0.99188 |
| ENST00000484721 | BTBD2 | 0.005197 | 0.994803 |
| ENST00000484721 | UBB | 2.43E-04 | 0.999757 |
| ENST00000484721 | PXDC1 | 0.002602 | 0.997398 |
| ENST00000484721 | EVC2 | 3.09E-04 | 0.999691 |
| ENST00000484721 | TMOD1 | 0.001296 | 0.998704 |
| ENST00000484721 | MYCN | 9.70E-04 | 0.99903 |
| ENST00000484721 | HMOX2 | 0.00607 | 0.99393 |
| ENST00000484721 | CCNK | 0.008169 | 0.991831 |
| ENST00000484721 | UBN2 | 0.009761 | -0.99024 |
| ENST00000484721 | CATG00000020284.1 | 0.008115 | -0.99188 |
| ENST00000510332 | RIPK1 | 0.001397 | -0.9986 |
| ENST00000510332 | NAPA | 0.004866 | 0.995134 |
| ENST00000510332 | BTBD2 | 0.004002 | 0.995998 |
| ENST00000510332 | HDHD5 | 8.91E-04 | -0.99911 |
| ENST00000510332 | PTGER1 | 0.004171 | -0.99583 |
| ENST00000510332 | POP1 | 0.001104 | 0.998896 |
| ENST00000510332 | CATG00000110054.1 | 0.007656 | 0.992344 |
| ENST00000510332 | TMOD1 | 0.007313 | 0.992687 |
| ENST00000510332 | TMEM230 | 0.008779 | 0.991221 |
| ENST00000510332 | YY2 | 0.00619 | -0.99381 |
| ENST00000510332 | KIAA1211L | 0.002524 | -0.99748 |
| ENST00000510332 | CCNK | 0.007113 | 0.992887 |
| ENST00000510332 | CATG00000039609.1 | 0.009481 | -0.99052 |
| ENST00000510332 | UBN2 | 0.006744 | -0.99326 |
| ENST00000510332 | CATG00000020284.1 | 0.001655 | -0.99835 |
| T040563 | PIK3R6 | 0.009881 | -0.99012 |
| T040563 | MDK | 0.008638 | -0.99136 |
| T040563 | ANXA3 | 0.004719 | -0.99528 |
| T040563 | MAP3K12 | 0.002405 | -0.99759 |
| T040563 | CLCN5 | 0.009141 | -0.99086 |
| T040563 | PIK3R6 | 0.009881 | -0.99012 |
| T040563 | MDK | 0.008638 | -0.99136 |
| T040563 | ANXA3 | 0.004719 | -0.99528 |
| T040563 | MAP3K12 | 0.002405 | -0.99759 |
| T040563 | CLCN5 | 0.009141 | -0.99086 |
| T040563 | PIK3R6 | 0.009881 | -0.99012 |
| T040563 | MDK | 0.008638 | -0.99136 |
| T040563 | ANXA3 | 0.004719 | -0.99528 |
| T040563 | MAP3K12 | 0.002405 | -0.99759 |
| T040563 | CLCN5 | 0.009141 | -0.99086 |
| T040563 | PIK3R6 | 0.009881 | -0.99012 |
| T040563 | MDK | 0.008638 | -0.99136 |
| T040563 | ANXA3 | 0.004719 | -0.99528 |
| T040563 | MAP3K12 | 0.002405 | -0.99759 |
| T040563 | CLCN5 | 0.009141 | -0.99086 |
| T040563 | PIK3R6 | 0.009881 | -0.99012 |
| T040563 | MDK | 0.008638 | -0.99136 |
| T040563 | ANXA3 | 0.004719 | -0.99528 |
| T040563 | MAP3K12 | 0.002405 | -0.99759 |
| T040563 | CLCN5 | 0.009141 | -0.99086 |
| T040563 | PIK3R6 | 0.009881 | -0.99012 |
| T040563 | MDK | 0.008638 | -0.99136 |
| T040563 | ANXA3 | 0.004719 | -0.99528 |
| T040563 | MAP3K12 | 0.002405 | -0.99759 |
| T040563 | CLCN5 | 0.009141 | -0.99086 |
| T040563 | PIK3R6 | 0.009881 | -0.99012 |
| T040563 | MDK | 0.008638 | -0.99136 |
| T040563 | ANXA3 | 0.004719 | -0.99528 |
| T040563 | MAP3K12 | 0.002405 | -0.99759 |
| T040563 | CLCN5 | 0.009141 | -0.99086 |
| T040563 | PIK3R6 | 0.009881 | -0.99012 |
| T040563 | MDK | 0.008638 | -0.99136 |
| T040563 | ANXA3 | 0.004719 | -0.99528 |
| T040563 | MAP3K12 | 0.002405 | -0.99759 |
| T040563 | CLCN5 | 0.009141 | -0.99086 |
| T040563 | PIK3R6 | 0.009881 | -0.99012 |
| T040563 | MDK | 0.008638 | -0.99136 |
| T040563 | ANXA3 | 0.004719 | -0.99528 |
| T040563 | MAP3K12 | 0.002405 | -0.99759 |
| T040563 | CLCN5 | 0.009141 | -0.99086 |
| T040563 | PIK3R6 | 0.009881 | -0.99012 |
| T040563 | MDK | 0.008638 | -0.99136 |
| T040563 | ANXA3 | 0.004719 | -0.99528 |
| T040563 | MAP3K12 | 0.002405 | -0.99759 |
| T040563 | CLCN5 | 0.009141 | -0.99086 |
| T040563 | PIK3R6 | 0.009881 | -0.99012 |
| T040563 | MDK | 0.008638 | -0.99136 |
| T040563 | ANXA3 | 0.004719 | -0.99528 |
| T040563 | MAP3K12 | 0.002405 | -0.99759 |
| T040563 | CLCN5 | 0.009141 | -0.99086 |
| T040563 | PIK3R6 | 0.009881 | -0.99012 |
| T040563 | MDK | 0.008638 | -0.99136 |
| T040563 | ANXA3 | 0.004719 | -0.99528 |
| T040563 | MAP3K12 | 0.002405 | -0.99759 |
| T040563 | CLCN5 | 0.009141 | -0.99086 |
| T040563 | PIK3R6 | 0.009881 | -0.99012 |
| T040563 | MDK | 0.008638 | -0.99136 |
| T040563 | ANXA3 | 0.004719 | -0.99528 |
| T040563 | MAP3K12 | 0.002405 | -0.99759 |
| T040563 | CLCN5 | 0.009141 | -0.99086 |
| ENST00000470902 | SHANK2 | 0.007055 | 0.992945 |
| ENST00000470902 | ASAP1 | 0.003993 | 0.996007 |
| ENST00000470902 | SUPV3L1 | 0.004776 | -0.99522 |
| ENST00000470902 | GPR156 | 0.003767 | 0.996233 |
| ENST00000470902 | FTCD | 0.008522 | 0.991478 |
| ENST00000470902 | KHDRBS3 | 0.004029 | -0.99597 |
| ENST00000470902 | SHANK2 | 0.007055 | 0.992945 |
| ENST00000470902 | ASAP1 | 0.003993 | 0.996007 |
| ENST00000470902 | SUPV3L1 | 0.004776 | -0.99522 |
| ENST00000470902 | GPR156 | 0.003767 | 0.996233 |
| ENST00000470902 | FTCD | 0.008522 | 0.991478 |
| ENST00000470902 | KHDRBS3 | 0.004029 | -0.99597 |
| ENST00000470902 | SHANK2 | 0.007055 | 0.992945 |
| ENST00000470902 | ASAP1 | 0.003993 | 0.996007 |
| ENST00000470902 | SUPV3L1 | 0.004776 | -0.99522 |
| ENST00000470902 | GPR156 | 0.003767 | 0.996233 |
| ENST00000470902 | FTCD | 0.008522 | 0.991478 |
| ENST00000470902 | KHDRBS3 | 0.004029 | -0.99597 |
| ENST00000470902 | SHANK2 | 0.007055 | 0.992945 |
| ENST00000470902 | ASAP1 | 0.003993 | 0.996007 |
| ENST00000470902 | SUPV3L1 | 0.004776 | -0.99522 |
| ENST00000470902 | GPR156 | 0.003767 | 0.996233 |
| ENST00000470902 | FTCD | 0.008522 | 0.991478 |
| ENST00000470902 | KHDRBS3 | 0.004029 | -0.99597 |
| ENST00000470902 | SHANK2 | 0.007055 | 0.992945 |
| ENST00000470902 | ASAP1 | 0.003993 | 0.996007 |
| ENST00000470902 | SUPV3L1 | 0.004776 | -0.99522 |
| ENST00000470902 | GPR156 | 0.003767 | 0.996233 |
| ENST00000470902 | FTCD | 0.008522 | 0.991478 |
| ENST00000470902 | KHDRBS3 | 0.004029 | -0.99597 |
| ENST00000426999 | PTPN6 | 0.008325 | -0.99167 |
| ENST00000426999 | LCK | 0.008112 | -0.99189 |
| ENST00000426999 | AGPS | 0.005367 | -0.99463 |
| ENST00000426999 | TEX13D | 0.002146 | 0.997854 |
| ENST00000426999 | NOL9 | 0.004162 | -0.99584 |
| ENST00000426999 | IRX3 | 4.06E-04 | 0.999594 |
| ENST00000426999 | SNX16 | 0.003726 | -0.99627 |
| ENST00000426999 | CD180 | 0.008241 | -0.99176 |
| ENST00000426999 | SPATA13 | 0.001088 | 0.998912 |
| ENST00000426999 | BHLHB9 | 0.00677 | 0.99323 |
| ENST00000426999 | PLTP | 0.009402 | 0.990598 |
| ENST00000426999 | PLCH2 | 0.006515 | 0.993485 |
| ENST00000426999 | COBL | 0.002102 | 0.997898 |
| ENST00000426999 | RRP9 | 0.006018 | 0.993982 |
| ENST00000426999 | CATG00000038465.1 | 0.009711 | 0.990289 |
| ENST00000426999 | SPSB2 | 0.007 | 0.993 |
| ENST00000426999 | SIPA1L2 | 0.001869 | 0.998131 |
| ENST00000426999 | PIPOX | 0.004093 | -0.99591 |
| ENST00000426999 | PTPN6 | 0.008325 | -0.99167 |
| ENST00000426999 | LCK | 0.008112 | -0.99189 |
| ENST00000426999 | AGPS | 0.005367 | -0.99463 |
| ENST00000426999 | TEX13D | 0.002146 | 0.997854 |
| ENST00000426999 | NOL9 | 0.004162 | -0.99584 |
| ENST00000426999 | IRX3 | 4.06E-04 | 0.999594 |
| ENST00000426999 | SNX16 | 0.003726 | -0.99627 |
| ENST00000426999 | CD180 | 0.008241 | -0.99176 |
| ENST00000426999 | SPATA13 | 0.001088 | 0.998912 |
| ENST00000426999 | BHLHB9 | 0.00677 | 0.99323 |
| ENST00000426999 | PLTP | 0.009402 | 0.990598 |
| ENST00000426999 | PLCH2 | 0.006515 | 0.993485 |
| ENST00000426999 | COBL | 0.002102 | 0.997898 |
| ENST00000426999 | RRP9 | 0.006018 | 0.993982 |
| ENST00000426999 | CATG00000038465.1 | 0.009711 | 0.990289 |
| ENST00000426999 | SPSB2 | 0.007 | 0.993 |
| ENST00000426999 | SIPA1L2 | 0.001869 | 0.998131 |
| ENST00000426999 | PIPOX | 0.004093 | -0.99591 |
| ENST00000426999 | PTPN6 | 0.008325 | -0.99167 |
| ENST00000426999 | LCK | 0.008112 | -0.99189 |
| ENST00000426999 | AGPS | 0.005367 | -0.99463 |
| ENST00000426999 | TEX13D | 0.002146 | 0.997854 |
| ENST00000426999 | NOL9 | 0.004162 | -0.99584 |
| ENST00000426999 | IRX3 | 4.06E-04 | 0.999594 |
| ENST00000426999 | SNX16 | 0.003726 | -0.99627 |
| ENST00000426999 | CD180 | 0.008241 | -0.99176 |
| ENST00000426999 | SPATA13 | 0.001088 | 0.998912 |
| ENST00000426999 | BHLHB9 | 0.00677 | 0.99323 |
| ENST00000426999 | PLTP | 0.009402 | 0.990598 |
| ENST00000426999 | PLCH2 | 0.006515 | 0.993485 |
| ENST00000426999 | COBL | 0.002102 | 0.997898 |
| ENST00000426999 | RRP9 | 0.006018 | 0.993982 |
| ENST00000426999 | CATG00000038465.1 | 0.009711 | 0.990289 |
| ENST00000426999 | SPSB2 | 0.007 | 0.993 |
| ENST00000426999 | SIPA1L2 | 0.001869 | 0.998131 |
| ENST00000426999 | PIPOX | 0.004093 | -0.99591 |
| ENST00000610089 | EPB41L1 | 0.003168 | -0.99683 |
| ENST00000610089 | RASSF6 | 0.003464 | -0.99654 |
| ENST00000610089 | PEX14 | 0.00681 | -0.99319 |
| ENST00000610089 | XYLT2 | 0.008484 | -0.99152 |
| ENST00000610089 | ZNF713 | 0.001082 | -0.99892 |
| ENST00000610089 | ABR | 0.006077 | -0.99392 |
| ENST00000610089 | KCTD1 | 0.008296 | -0.9917 |
| ENST00000610089 | CATG00000047316.1 | 0.002039 | -0.99796 |
| ENST00000610089 | SAMD1 | 0.009596 | 0.990404 |
| ENST00000610089 | ZMYND15 | 8.77E-04 | -0.99912 |
| ENST00000610089 | CATG00000057824.1 | 0.00957 | -0.99043 |
| ENST00000610089 | IQCF5 | 0.00849 | -0.99151 |
| ENST00000610089 | NTF3 | 0.004966 | -0.99503 |
| ENST00000610089 | DNAL4 | 0.002056 | 0.997944 |
| ENST00000610089 | INKA2 | 0.009636 | -0.99036 |
| ENST00000610089 | FAM53B | 0.008336 | 0.991664 |
| ENST00000610089 | LDHB | 0.005241 | 0.994759 |
| ENST00000610089 | ZNF37A | 0.004976 | 0.995024 |
| ENST00000610089 | DICER1 | 0.002555 | 0.997445 |
| ENST00000466291 | CMYA5 | 0.004324 | -0.99568 |
| ENST00000466291 | CCDC172 | 0.005209 | -0.99479 |
| ENST00000466291 | RCSD1 | 0.004448 | 0.995552 |
| ENST00000466291 | MOK | 0.002318 | 0.997682 |
| TCONS_00022739 | RHEB | 0.00606 | -0.99394 |
| TCONS_00022739 | ARHGAP8 | 0.00656 | -0.99344 |
| TCONS_00022739 | PLA2G15 | 0.008055 | -0.99194 |
| TCONS_00022739 | UFC1 | 0.002542 | -0.99746 |
| TCONS_00022739 | JCAD | 1.89E-04 | 0.999811 |
| TCONS_00022739 | TREM1 | 0.001318 | 0.998682 |
| TCONS_00022739 | PLA1A | 0.009831 | 0.990169 |
| TCONS_00022739 | ANKS4B | 0.006732 | 0.993268 |
| ENST00000523005 | TNFRSF18 | 0.009274 | -0.99073 |
| ENST00000523005 | SLC2A4 | 0.006259 | 0.993741 |
| ENST00000523005 | HIRA | 0.007226 | -0.99277 |
| ENST00000523005 | TMEM155 | 0.003218 | 0.996782 |
| ENST00000523005 | SPATA33 | 0.004235 | -0.99577 |
| ENST00000523005 | AQP1 | 0.004451 | -0.99555 |
| ENST00000523005 | KLHL35 | 0.005604 | -0.9944 |
| ENST00000523005 | MRNIP | 0.009382 | 0.990618 |
| ENST00000523005 | SLC22A12 | 0.005835 | 0.994165 |
| ENST00000523005 | PLPPR4 | 0.004842 | -0.99516 |
| ENST00000523005 | HLA-A | 0.002576 | -0.99742 |
| ENST00000523005 | MT1F | 0.002184 | -0.99782 |
| ENST00000523005 | WWP1 | 0.006691 | -0.99331 |
| ENST00000523005 | ARSD | 0.006929 | -0.99307 |
| ENST00000523005 | KCNK10 | 0.004531 | 0.995469 |
| ENST00000523005 | KYAT3 | 2.25E-04 | 0.999775 |
| ENST00000523005 | HEATR9 | 0.007784 | 0.992216 |
| ENST00000523005 | ATG4C | 0.003166 | 0.996834 |
| ENST00000523005 | TNFRSF18 | 0.009274 | -0.99073 |
| ENST00000523005 | SLC2A4 | 0.006259 | 0.993741 |
| ENST00000523005 | HIRA | 0.007226 | -0.99277 |
| ENST00000523005 | TMEM155 | 0.003218 | 0.996782 |
| ENST00000523005 | SPATA33 | 0.004235 | -0.99577 |
| ENST00000523005 | AQP1 | 0.004451 | -0.99555 |
| ENST00000523005 | KLHL35 | 0.005604 | -0.9944 |
| ENST00000523005 | MRNIP | 0.009382 | 0.990618 |
| ENST00000523005 | SLC22A12 | 0.005835 | 0.994165 |
| ENST00000523005 | PLPPR4 | 0.004842 | -0.99516 |
| ENST00000523005 | HLA-A | 0.002576 | -0.99742 |
| ENST00000523005 | MT1F | 0.002184 | -0.99782 |
| ENST00000523005 | WWP1 | 0.006691 | -0.99331 |
| ENST00000523005 | ARSD | 0.006929 | -0.99307 |
| ENST00000523005 | KCNK10 | 0.004531 | 0.995469 |
| ENST00000523005 | KYAT3 | 2.25E-04 | 0.999775 |
| ENST00000523005 | HEATR9 | 0.007784 | 0.992216 |
| ENST00000523005 | ATG4C | 0.003166 | 0.996834 |
| ENST00000448597 | RRAS2 | 0.007264 | 0.992736 |
| ENST00000448597 | WDR60 | 0.007019 | 0.992981 |
| ENST00000448597 | WBP2NL | 0.002684 | 0.997316 |
| ENST00000448597 | GSC | 0.009222 | 0.990778 |
| ENST00000448597 | ZP1 | 0.00608 | 0.99392 |
| MICT00000027555 | TRMT11 | 0.005177 | 0.994823 |
| MICT00000027555 | ADGRE1 | 0.009804 | 0.990196 |
| MICT00000027555 | CPSF3 | 0.00768 | 0.99232 |
| MICT00000027555 | C11orf71 | 0.008203 | 0.991797 |
| MICT00000027555 | MYLK3 | 0.001662 | 0.998338 |
| MICT00000027555 | SIRT6 | 0.003102 | 0.996898 |
| MICT00000027555 | SLC39A12 | 0.00833 | -0.99167 |
| ENST00000518837 | LRMDA | 0.001881 | -0.99812 |
| ENST00000518837 | RETSAT | 0.002788 | -0.99721 |
| ENST00000518837 | CAPS | 0.009207 | -0.99079 |
| ENST00000518837 | PHF13 | 0.00748 | 0.99252 |
| ENST00000518837 | YES1 | 2.93E-04 | 0.999707 |
| ENST00000518837 | CTSL | 0.003742 | -0.99626 |
| ENST00000518837 | VMP1 | 0.004533 | 0.995467 |
| ENST00000518837 | GDF2 | 0.001155 | 0.998845 |
| ENST00000518837 | LRMDA | 0.001881 | -0.99812 |
| ENST00000518837 | RETSAT | 0.002788 | -0.99721 |
| ENST00000518837 | CAPS | 0.009207 | -0.99079 |
| ENST00000518837 | PHF13 | 0.00748 | 0.99252 |
| ENST00000518837 | YES1 | 2.93E-04 | 0.999707 |
| ENST00000518837 | CTSL | 0.003742 | -0.99626 |
| ENST00000518837 | VMP1 | 0.004533 | 0.995467 |
| ENST00000518837 | GDF2 | 0.001155 | 0.998845 |
| ENST00000518837 | LRMDA | 0.001881 | -0.99812 |
| ENST00000518837 | RETSAT | 0.002788 | -0.99721 |
| ENST00000518837 | CAPS | 0.009207 | -0.99079 |
| ENST00000518837 | PHF13 | 0.00748 | 0.99252 |
| ENST00000518837 | YES1 | 2.93E-04 | 0.999707 |
| ENST00000518837 | CTSL | 0.003742 | -0.99626 |
| ENST00000518837 | VMP1 | 0.004533 | 0.995467 |
| ENST00000518837 | GDF2 | 0.001155 | 0.998845 |
| ENST00000518837 | LRMDA | 0.001881 | -0.99812 |
| ENST00000518837 | RETSAT | 0.002788 | -0.99721 |
| ENST00000518837 | CAPS | 0.009207 | -0.99079 |
| ENST00000518837 | PHF13 | 0.00748 | 0.99252 |
| ENST00000518837 | YES1 | 2.93E-04 | 0.999707 |
| ENST00000518837 | CTSL | 0.003742 | -0.99626 |
| ENST00000518837 | VMP1 | 0.004533 | 0.995467 |
| ENST00000518837 | GDF2 | 0.001155 | 0.998845 |
| ENST00000518837 | LRMDA | 0.001881 | -0.99812 |
| ENST00000518837 | RETSAT | 0.002788 | -0.99721 |
| ENST00000518837 | CAPS | 0.009207 | -0.99079 |
| ENST00000518837 | PHF13 | 0.00748 | 0.99252 |
| ENST00000518837 | YES1 | 2.93E-04 | 0.999707 |
| ENST00000518837 | CTSL | 0.003742 | -0.99626 |
| ENST00000518837 | VMP1 | 0.004533 | 0.995467 |
| ENST00000518837 | GDF2 | 0.001155 | 0.998845 |
| ENST00000425150 | DNAJC6 | 9.69E-05 | -0.9999 |
| ENST00000425150 | MFAP3L | 0.001721 | -0.99828 |
| ENST00000425150 | CATG00000086946.1 | 0.006464 | -0.99354 |
| ENST00000425150 | LGALS1 | 0.006414 | 0.993586 |
| ENST00000425150 | SPACA9 | 2.56E-05 | -0.99997 |
| ENST00000425150 | CMBL | 0.005687 | -0.99431 |
| ENST00000425150 | ORM1 | 0.006092 | -0.99391 |
| ENST00000616366 | ZFY | 0.006264 | -0.99374 |
| ENST00000616366 | EXOC3L2 | 0.008568 | 0.991432 |
| ENST00000616366 | PLD3 | 0.002573 | -0.99743 |
| ENST00000616366 | RAB8A | 0.003146 | -0.99685 |
| ENST00000616366 | SSX1 | 0.003971 | 0.996029 |
| ENST00000616366 | WSCD2 | 3.10E-06 | 0.999997 |
| ENST00000616366 | GRIN3B | 0.002407 | -0.99759 |
| ENST00000616366 | TNFAIP6 | 0.007517 | -0.99248 |
| ENST00000616366 | PTPRK | 0.003149 | 0.996851 |
| ENST00000616366 | CATG00000039609.1 | 0.007006 | 0.992994 |
| ENST00000616366 | DEFA4 | 0.008586 | 0.991414 |
| TCONS_00026547 | ZNF185 | 0.001311 | -0.99869 |
| TCONS_00026547 | FAM169B | 3.16E-05 | -0.99997 |
| TCONS_00026547 | IFNA6 | 0.008649 | -0.99135 |
| TCONS_00026547 | GPR82 | 0.002761 | -0.99724 |
| ENST00000512559 | NOC4L | 0.006501 | 0.993499 |
| ENST00000512559 | CATG00000080699.1 | 0.002301 | 0.997699 |
| ENST00000512559 | SLC24A5 | 0.009133 | -0.99087 |
| ENST00000512559 | TCOF1 | 0.004763 | 0.995237 |
| ENST00000512559 | SAMD13 | 0.005253 | 0.994747 |
| ENST00000512559 | ZDHHC16 | 2.84E-04 | -0.99972 |
| ENST00000512559 | CDKL2 | 0.008566 | 0.991434 |
| ENST00000512559 | MACROD2 | 0.001153 | 0.998847 |
| ENST00000512559 | CHST12 | 0 | 1 |
| ENST00000512559 | HIPK2 | 0.004794 | -0.99521 |
| ENST00000512559 | PCDHB8 | 0 | 1 |
| ENST00000536131 | TMED3 | 0.004565 | -0.99544 |
| ENST00000536131 | PPP3CC | 0.008563 | -0.99144 |
| ENST00000536131 | LTA4H | 0.003474 | -0.99653 |
| ENST00000536131 | TUT4 | 0.003789 | -0.99621 |
| ENST00000536131 | WDR33 | 9.49E-04 | 0.999051 |
| ENST00000536131 | PALM3 | 0.009249 | -0.99075 |
| ENST00000536131 | CATG00000063823.1 | 0.001242 | 0.998758 |
| ENST00000536131 | ZNF607 | 0.004777 | 0.995223 |
| ENST00000536131 | PSMD4 | 9.86E-04 | -0.99901 |
| ENST00000536131 | OR6F1 | 0.005411 | 0.994589 |
| ENST00000536131 | ADAM28 | 0.001035 | -0.99896 |
| ENST00000536131 | CATG00000087047.1 | 0.004678 | 0.995322 |
| ENST00000536131 | AC109583.1 | 0.004826 | 0.995174 |
| ENST00000536131 | RAD51AP2 | 8.81E-04 | -0.99912 |
| ENST00000440522 | RPS7 | 0.003372 | -0.99663 |
| ENST00000440522 | 11-Mar | 0.007661 | 0.992339 |
| ENST00000440522 | ACOT12 | 0.002342 | -0.99766 |
| ENST00000440522 | PALM3 | 0.002351 | 0.997649 |
| ENST00000440522 | BCAS4 | 0.00159 | -0.99841 |
| ENST00000440522 | PELP1 | 7.11E-04 | -0.99929 |
| ENST00000440522 | OR8B4 | 0.006137 | 0.993863 |
| ENST00000440522 | MYH4 | 0.003579 | -0.99642 |
| ENST00000440522 | CLEC4E | 0.009781 | -0.99022 |
| ENST00000440522 | KNCN | 0.001071 | -0.99893 |
| ENST00000440522 | FANCL | 0.007118 | -0.99288 |
| ENST00000440522 | CAMK2N2 | 2.13E-04 | -0.99979 |
| ENST00000434993 | PDE4C | 0.009717 | -0.99028 |
| ENST00000434993 | PDE4C | 0.009717 | -0.99028 |
| ENST00000434993 | PDE4C | 0.009717 | -0.99028 |
| ENST00000434993 | PDE4C | 0.009717 | -0.99028 |
| ENST00000434993 | PDE4C | 0.009717 | -0.99028 |
| ENST00000434993 | PDE4C | 0.009717 | -0.99028 |
| ENST00000434993 | PDE4C | 0.009717 | -0.99028 |
| ENST00000434993 | PDE4C | 0.009717 | -0.99028 |
| ENST00000434993 | PDE4C | 0.009717 | -0.99028 |
| ENST00000434993 | PDE4C | 0.009717 | -0.99028 |
| ENST00000434993 | PDE4C | 0.009717 | -0.99028 |
| ENST00000434993 | PDE4C | 0.009717 | -0.99028 |
| ENST00000434993 | PDE4C | 0.009717 | -0.99028 |
| ENST00000434993 | PDE4C | 0.009717 | -0.99028 |
| ENST00000434993 | PDE4C | 0.009717 | -0.99028 |
| ENST00000434993 | PDE4C | 0.009717 | -0.99028 |
| ENST00000434993 | PDE4C | 0.009717 | -0.99028 |
| ENST00000434993 | PDE4C | 0.009717 | -0.99028 |
| ENST00000434993 | PDE4C | 0.009717 | -0.99028 |
| ENST00000614972 | ACTL7B | 0.00457 | 0.99543 |
| ENST00000614972 | FBXL16 | 0.00414 | -0.99586 |
| ENST00000614972 | MED12L | 8.77E-04 | 0.999123 |
| ENST00000452481 | AX748369 | 0.004193 | 0.995807 |
| ENST00000452481 | SLC38A10 | 0.001773 | 0.998227 |
| ENST00000452481 | ZNF30 | 0.003104 | -0.9969 |
| ENST00000452481 | SZRD1 | 0.006004 | -0.994 |
| ENST00000452481 | MLLT1 | 0.008414 | 0.991586 |
| ENST00000452481 | PNPLA2 | 0.007728 | 0.992272 |
| ENST00000452481 | STMND1 | 0.001424 | 0.998576 |
| ENST00000452481 | MAP7 | 0.007485 | 0.992515 |
| ENST00000452481 | DRC1 | 0.001584 | 0.998416 |
| ENST00000452481 | EFCAB8 | 0.003146 | 0.996854 |
| ENST00000452481 | MSI1 | 0.00969 | 0.99031 |
| ENST00000452481 | AX748369 | 0.004193 | 0.995807 |
| ENST00000452481 | SLC38A10 | 0.001773 | 0.998227 |
| ENST00000452481 | ZNF30 | 0.003104 | -0.9969 |
| ENST00000452481 | SZRD1 | 0.006004 | -0.994 |
| ENST00000452481 | MLLT1 | 0.008414 | 0.991586 |
| ENST00000452481 | PNPLA2 | 0.007728 | 0.992272 |
| ENST00000452481 | STMND1 | 0.001424 | 0.998576 |
| ENST00000452481 | MAP7 | 0.007485 | 0.992515 |
| ENST00000452481 | DRC1 | 0.001584 | 0.998416 |
| ENST00000452481 | EFCAB8 | 0.003146 | 0.996854 |
| ENST00000452481 | MSI1 | 0.00969 | 0.99031 |
| ENST00000452481 | AX748369 | 0.004193 | 0.995807 |
| ENST00000452481 | SLC38A10 | 0.001773 | 0.998227 |
| ENST00000452481 | ZNF30 | 0.003104 | -0.9969 |
| ENST00000452481 | SZRD1 | 0.006004 | -0.994 |
| ENST00000452481 | MLLT1 | 0.008414 | 0.991586 |
| ENST00000452481 | PNPLA2 | 0.007728 | 0.992272 |
| ENST00000452481 | STMND1 | 0.001424 | 0.998576 |
| ENST00000452481 | MAP7 | 0.007485 | 0.992515 |
| ENST00000452481 | DRC1 | 0.001584 | 0.998416 |
| ENST00000452481 | EFCAB8 | 0.003146 | 0.996854 |
| ENST00000452481 | MSI1 | 0.00969 | 0.99031 |
| ENST00000452481 | AX748369 | 0.004193 | 0.995807 |
| ENST00000452481 | SLC38A10 | 0.001773 | 0.998227 |
| ENST00000452481 | ZNF30 | 0.003104 | -0.9969 |
| ENST00000452481 | SZRD1 | 0.006004 | -0.994 |
| ENST00000452481 | MLLT1 | 0.008414 | 0.991586 |
| ENST00000452481 | PNPLA2 | 0.007728 | 0.992272 |
| ENST00000452481 | STMND1 | 0.001424 | 0.998576 |
| ENST00000452481 | MAP7 | 0.007485 | 0.992515 |
| ENST00000452481 | DRC1 | 0.001584 | 0.998416 |
| ENST00000452481 | EFCAB8 | 0.003146 | 0.996854 |
| ENST00000452481 | MSI1 | 0.00969 | 0.99031 |
| ENST00000452481 | AX748369 | 0.004193 | 0.995807 |
| ENST00000452481 | SLC38A10 | 0.001773 | 0.998227 |
| ENST00000452481 | ZNF30 | 0.003104 | -0.9969 |
| ENST00000452481 | SZRD1 | 0.006004 | -0.994 |
| ENST00000452481 | MLLT1 | 0.008414 | 0.991586 |
| ENST00000452481 | PNPLA2 | 0.007728 | 0.992272 |
| ENST00000452481 | STMND1 | 0.001424 | 0.998576 |
| ENST00000452481 | MAP7 | 0.007485 | 0.992515 |
| ENST00000452481 | DRC1 | 0.001584 | 0.998416 |
| ENST00000452481 | EFCAB8 | 0.003146 | 0.996854 |
| ENST00000452481 | MSI1 | 0.00969 | 0.99031 |
| ENST00000452481 | AX748369 | 0.004193 | 0.995807 |
| ENST00000452481 | SLC38A10 | 0.001773 | 0.998227 |
| ENST00000452481 | ZNF30 | 0.003104 | -0.9969 |
| ENST00000452481 | SZRD1 | 0.006004 | -0.994 |
| ENST00000452481 | MLLT1 | 0.008414 | 0.991586 |
| ENST00000452481 | PNPLA2 | 0.007728 | 0.992272 |
| ENST00000452481 | STMND1 | 0.001424 | 0.998576 |
| ENST00000452481 | MAP7 | 0.007485 | 0.992515 |
| ENST00000452481 | DRC1 | 0.001584 | 0.998416 |
| ENST00000452481 | EFCAB8 | 0.003146 | 0.996854 |
| ENST00000452481 | MSI1 | 0.00969 | 0.99031 |
| ENST00000452481 | AX748369 | 0.004193 | 0.995807 |
| ENST00000452481 | SLC38A10 | 0.001773 | 0.998227 |
| ENST00000452481 | ZNF30 | 0.003104 | -0.9969 |
| ENST00000452481 | SZRD1 | 0.006004 | -0.994 |
| ENST00000452481 | MLLT1 | 0.008414 | 0.991586 |
| ENST00000452481 | PNPLA2 | 0.007728 | 0.992272 |
| ENST00000452481 | STMND1 | 0.001424 | 0.998576 |
| ENST00000452481 | MAP7 | 0.007485 | 0.992515 |
| ENST00000452481 | DRC1 | 0.001584 | 0.998416 |
| ENST00000452481 | EFCAB8 | 0.003146 | 0.996854 |
| ENST00000452481 | MSI1 | 0.00969 | 0.99031 |
| ENST00000439621 | NOC4L | 0.006501 | 0.993499 |
| ENST00000439621 | CATG00000080699.1 | 0.002301 | 0.997699 |
| ENST00000439621 | SLC24A5 | 0.009133 | -0.99087 |
| ENST00000439621 | TCOF1 | 0.004763 | 0.995237 |
| ENST00000439621 | SAMD13 | 0.005253 | 0.994747 |
| ENST00000439621 | ZDHHC16 | 2.84E-04 | -0.99972 |
| ENST00000439621 | CDKL2 | 0.008566 | 0.991434 |
| ENST00000439621 | MACROD2 | 0.001153 | 0.998847 |
| ENST00000439621 | CHST12 | 0 | 1 |
| ENST00000439621 | HIPK2 | 0.004794 | -0.99521 |
| ENST00000439621 | PCDHB8 | 0 | 1 |
| ENST00000439621 | NOC4L | 0.006501 | 0.993499 |
| ENST00000439621 | CATG00000080699.1 | 0.002301 | 0.997699 |
| ENST00000439621 | SLC24A5 | 0.009133 | -0.99087 |
| ENST00000439621 | TCOF1 | 0.004763 | 0.995237 |
| ENST00000439621 | SAMD13 | 0.005253 | 0.994747 |
| ENST00000439621 | ZDHHC16 | 2.84E-04 | -0.99972 |
| ENST00000439621 | CDKL2 | 0.008566 | 0.991434 |
| ENST00000439621 | MACROD2 | 0.001153 | 0.998847 |
| ENST00000439621 | CHST12 | 0 | 1 |
| ENST00000439621 | HIPK2 | 0.004794 | -0.99521 |
| ENST00000439621 | PCDHB8 | 0 | 1 |
| ENST00000439621 | NOC4L | 0.006501 | 0.993499 |
| ENST00000439621 | CATG00000080699.1 | 0.002301 | 0.997699 |
| ENST00000439621 | SLC24A5 | 0.009133 | -0.99087 |
| ENST00000439621 | TCOF1 | 0.004763 | 0.995237 |
| ENST00000439621 | SAMD13 | 0.005253 | 0.994747 |
| ENST00000439621 | ZDHHC16 | 2.84E-04 | -0.99972 |
| ENST00000439621 | CDKL2 | 0.008566 | 0.991434 |
| ENST00000439621 | MACROD2 | 0.001153 | 0.998847 |
| ENST00000439621 | CHST12 | 0 | 1 |
| ENST00000439621 | HIPK2 | 0.004794 | -0.99521 |
| ENST00000439621 | PCDHB8 | 0 | 1 |
| ENST00000439621 | NOC4L | 0.006501 | 0.993499 |
| ENST00000439621 | CATG00000080699.1 | 0.002301 | 0.997699 |
| ENST00000439621 | SLC24A5 | 0.009133 | -0.99087 |
| ENST00000439621 | TCOF1 | 0.004763 | 0.995237 |
| ENST00000439621 | SAMD13 | 0.005253 | 0.994747 |
| ENST00000439621 | ZDHHC16 | 2.84E-04 | -0.99972 |
| ENST00000439621 | CDKL2 | 0.008566 | 0.991434 |
| ENST00000439621 | MACROD2 | 0.001153 | 0.998847 |
| ENST00000439621 | CHST12 | 0 | 1 |
| ENST00000439621 | HIPK2 | 0.004794 | -0.99521 |
| ENST00000439621 | PCDHB8 | 0 | 1 |
| ENST00000596410 | CATG00000003494.1 | 0.007404 | -0.9926 |
| ENST00000596410 | CACHD1 | 0.004106 | -0.99589 |
| ENST00000596410 | INSC | 0.002852 | -0.99715 |
| ENST00000596410 | PCDHGA3 | 0.006302 | -0.9937 |
| ENST00000596410 | CATG00000089121.1 | 0.007248 | 0.992752 |
| ENST00000596410 | OAS1 | 1.30E-04 | -0.99987 |
| ENST00000596410 | DCD | 0.007331 | -0.99267 |
| ENST00000596410 | CSNK1A1 | 1.91E-04 | 0.999809 |
| ENST00000596410 | ZNF587B | 0.00186 | 0.99814 |
| ENST00000596410 | INO80B | 0.006918 | -0.99308 |
| ENST00000596410 | RPL17 | 4.14E-04 | 0.999586 |
| ENST00000596410 | WAPL | 0.003401 | 0.996599 |
| ENST00000596410 | PLCL1 | 0.005473 | 0.994527 |
| ENST00000596410 | AL627171.2 | 0.008637 | 0.991363 |
| ENST00000596410 | GABPA | 0.004252 | -0.99575 |
| ENST00000497885 | TAS2R42 | 2.81E-04 | -0.99972 |
| ENST00000497885 | B3GNT3 | 0.009813 | 0.990187 |
| ENST00000497885 | GRINA | 0.002857 | -0.99714 |
| ENST00000497885 | PPP3CC | 0.006438 | 0.993562 |
| ENST00000497885 | PSRC1 | 0.001561 | 0.998439 |
| ENST00000497885 | PLIN4 | 0.007927 | -0.99207 |
| ENST00000497885 | ABCB8 | 0.006716 | -0.99328 |
| ENST00000497885 | LILRB1 | 0.002141 | 0.997859 |
| ENST00000497885 | RASL10A | 0.001402 | 0.998598 |
| ENST00000497885 | LTA4H | 0.002068 | 0.997932 |
| ENST00000497885 | TUT4 | 0.005031 | 0.994969 |
| ENST00000497885 | WDR33 | 0.009927 | -0.99007 |
| ENST00000497885 | CATG00000053512.1 | 0.005912 | 0.994088 |
| ENST00000497885 | HLF | 0.006097 | -0.9939 |
| ENST00000497885 | NFX1 | 0.001114 | 0.998886 |
| ENST00000497885 | PEA15 | 0.001235 | -0.99877 |
| ENST00000497885 | IFT122 | 0.007445 | -0.99255 |
| ENST00000497885 | SLC12A3 | 0.002762 | 0.997238 |
| ENST00000497885 | WNT8B | 0.001187 | -0.99881 |
| ENST00000497885 | UNC5C | 8.27E-04 | 0.999173 |
| ENST00000497885 | AGO2 | 0.006597 | -0.9934 |
| ENST00000497885 | TRIM47 | 0.009335 | -0.99066 |
| ENST00000497885 | CATG00000087047.1 | 0.001724 | -0.99828 |
| ENST00000497885 | FAM174A | 0.00946 | 0.99054 |
| ENST00000497885 | CFAP410 | 0.003707 | -0.99629 |
| ENST00000497885 | HPS1 | 0.00219 | -0.99781 |
| ENST00000497885 | RAD51AP2 | 0.0055 | 0.9945 |
| ENST00000497885 | TAS2R42 | 2.81E-04 | -0.99972 |
| ENST00000497885 | B3GNT3 | 0.009813 | 0.990187 |
| ENST00000497885 | GRINA | 0.002857 | -0.99714 |
| ENST00000497885 | PPP3CC | 0.006438 | 0.993562 |
| ENST00000497885 | PSRC1 | 0.001561 | 0.998439 |
| ENST00000497885 | PLIN4 | 0.007927 | -0.99207 |
| ENST00000497885 | ABCB8 | 0.006716 | -0.99328 |
| ENST00000497885 | LILRB1 | 0.002141 | 0.997859 |
| ENST00000497885 | RASL10A | 0.001402 | 0.998598 |
| ENST00000497885 | LTA4H | 0.002068 | 0.997932 |
| ENST00000497885 | TUT4 | 0.005031 | 0.994969 |
| ENST00000497885 | WDR33 | 0.009927 | -0.99007 |
| ENST00000497885 | CATG00000053512.1 | 0.005912 | 0.994088 |
| ENST00000497885 | HLF | 0.006097 | -0.9939 |
| ENST00000497885 | NFX1 | 0.001114 | 0.998886 |
| ENST00000497885 | PEA15 | 0.001235 | -0.99877 |
| ENST00000497885 | IFT122 | 0.007445 | -0.99255 |
| ENST00000497885 | SLC12A3 | 0.002762 | 0.997238 |
| ENST00000497885 | WNT8B | 0.001187 | -0.99881 |
| ENST00000497885 | UNC5C | 8.27E-04 | 0.999173 |
| ENST00000497885 | AGO2 | 0.006597 | -0.9934 |
| ENST00000497885 | TRIM47 | 0.009335 | -0.99066 |
| ENST00000497885 | CATG00000087047.1 | 0.001724 | -0.99828 |
| ENST00000497885 | FAM174A | 0.00946 | 0.99054 |
| ENST00000497885 | CFAP410 | 0.003707 | -0.99629 |
| ENST00000497885 | HPS1 | 0.00219 | -0.99781 |
| ENST00000497885 | RAD51AP2 | 0.0055 | 0.9945 |
| ENST00000497885 | TAS2R42 | 2.81E-04 | -0.99972 |
| ENST00000497885 | B3GNT3 | 0.009813 | 0.990187 |
| ENST00000497885 | GRINA | 0.002857 | -0.99714 |
| ENST00000497885 | PPP3CC | 0.006438 | 0.993562 |
| ENST00000497885 | PSRC1 | 0.001561 | 0.998439 |
| ENST00000497885 | PLIN4 | 0.007927 | -0.99207 |
| ENST00000497885 | ABCB8 | 0.006716 | -0.99328 |
| ENST00000497885 | LILRB1 | 0.002141 | 0.997859 |
| ENST00000497885 | RASL10A | 0.001402 | 0.998598 |
| ENST00000497885 | LTA4H | 0.002068 | 0.997932 |
| ENST00000497885 | TUT4 | 0.005031 | 0.994969 |
| ENST00000497885 | WDR33 | 0.009927 | -0.99007 |
| ENST00000497885 | CATG00000053512.1 | 0.005912 | 0.994088 |
| ENST00000497885 | HLF | 0.006097 | -0.9939 |
| ENST00000497885 | NFX1 | 0.001114 | 0.998886 |
| ENST00000497885 | PEA15 | 0.001235 | -0.99877 |
| ENST00000497885 | IFT122 | 0.007445 | -0.99255 |
| ENST00000497885 | SLC12A3 | 0.002762 | 0.997238 |
| ENST00000497885 | WNT8B | 0.001187 | -0.99881 |
| ENST00000497885 | UNC5C | 8.27E-04 | 0.999173 |
| ENST00000497885 | AGO2 | 0.006597 | -0.9934 |
| ENST00000497885 | TRIM47 | 0.009335 | -0.99066 |
| ENST00000497885 | CATG00000087047.1 | 0.001724 | -0.99828 |
| ENST00000497885 | FAM174A | 0.00946 | 0.99054 |
| ENST00000497885 | CFAP410 | 0.003707 | -0.99629 |
| ENST00000497885 | HPS1 | 0.00219 | -0.99781 |
| ENST00000497885 | RAD51AP2 | 0.0055 | 0.9945 |
| T268536 | AL358113.1 | 0.004373 | -0.99563 |
| NR_073171 | TOB1 | 0.00555 | -0.99445 |
| NR_073171 | MED20 | 0.00791 | 0.99209 |
| NR_073171 | PLTP | 0.009568 | 0.990432 |
| NR_073171 | CATG00000038465.1 | 0.008586 | 0.991414 |
| NR_073171 | CFLAR | 0.006203 | -0.9938 |
| NR_073171 | C17orf47 | 0.003965 | 0.996035 |
| NR_073171 | TSFM | 0.007588 | -0.99241 |
| NR_073171 | TOB1 | 0.00555 | -0.99445 |
| NR_073171 | MED20 | 0.00791 | 0.99209 |
| NR_073171 | PLTP | 0.009568 | 0.990432 |
| NR_073171 | CATG00000038465.1 | 0.008586 | 0.991414 |
| NR_073171 | CFLAR | 0.006203 | -0.9938 |
| NR_073171 | C17orf47 | 0.003965 | 0.996035 |
| NR_073171 | TSFM | 0.007588 | -0.99241 |
| NR_073171 | TOB1 | 0.00555 | -0.99445 |
| NR_073171 | MED20 | 0.00791 | 0.99209 |
| NR_073171 | PLTP | 0.009568 | 0.990432 |
| NR_073171 | CATG00000038465.1 | 0.008586 | 0.991414 |
| NR_073171 | CFLAR | 0.006203 | -0.9938 |
| NR_073171 | C17orf47 | 0.003965 | 0.996035 |
| NR_073171 | TSFM | 0.007588 | -0.99241 |
| ENST00000505326 | TMED3 | 0.009093 | 0.990907 |
| ENST00000505326 | ACOT12 | 0.005031 | -0.99497 |
| ENST00000505326 | PALM3 | 5.08E-04 | 0.999492 |
| ENST00000505326 | BCAS4 | 8.85E-04 | -0.99912 |
| ENST00000505326 | PELP1 | 0.005675 | -0.99433 |
| ENST00000505326 | CATG00000063823.1 | 0.005322 | -0.99468 |
| ENST00000505326 | PSMD4 | 0.005703 | 0.994297 |
| ENST00000505326 | OR8B4 | 0.006994 | 0.993006 |
| ENST00000505326 | MYH4 | 0.008439 | -0.99156 |
| ENST00000505326 | ADAM28 | 0.005617 | 0.994383 |
| ENST00000505326 | KNCN | 0.00127 | -0.99873 |
| ENST00000505326 | FANCL | 0.007675 | -0.99233 |
| ENST00000505326 | CAMK2N2 | 0.003084 | -0.99692 |
| ENST00000585999 | TOB1 | 0.007645 | 0.992355 |
| ENST00000585999 | ANKRD13D | 0.002101 | 0.997899 |
| ENST00000585999 | MED20 | 0.004712 | -0.99529 |
| ENST00000585999 | CATG00000087963.1 | 0.002907 | -0.99709 |
| ENST00000585999 | EPHB6 | 0.001501 | -0.9985 |
| ENST00000585999 | CATG00000021838.1 | 0.003913 | 0.996087 |
| ENST00000585999 | TSFM | 4.18E-04 | 0.999582 |
| ENST00000585999 | TOB1 | 0.007645 | 0.992355 |
| ENST00000585999 | ANKRD13D | 0.002101 | 0.997899 |
| ENST00000585999 | MED20 | 0.004712 | -0.99529 |
| ENST00000585999 | CATG00000087963.1 | 0.002907 | -0.99709 |
| ENST00000585999 | EPHB6 | 0.001501 | -0.9985 |
| ENST00000585999 | CATG00000021838.1 | 0.003913 | 0.996087 |
| ENST00000585999 | TSFM | 4.18E-04 | 0.999582 |
| ENST00000585999 | TOB1 | 0.007645 | 0.992355 |
| ENST00000585999 | ANKRD13D | 0.002101 | 0.997899 |
| ENST00000585999 | MED20 | 0.004712 | -0.99529 |
| ENST00000585999 | CATG00000087963.1 | 0.002907 | -0.99709 |
| ENST00000585999 | EPHB6 | 0.001501 | -0.9985 |
| ENST00000585999 | CATG00000021838.1 | 0.003913 | 0.996087 |
| ENST00000585999 | TSFM | 4.18E-04 | 0.999582 |
| ENST00000585999 | TOB1 | 0.007645 | 0.992355 |
| ENST00000585999 | ANKRD13D | 0.002101 | 0.997899 |
| ENST00000585999 | MED20 | 0.004712 | -0.99529 |
| ENST00000585999 | CATG00000087963.1 | 0.002907 | -0.99709 |
| ENST00000585999 | EPHB6 | 0.001501 | -0.9985 |
| ENST00000585999 | CATG00000021838.1 | 0.003913 | 0.996087 |
| ENST00000585999 | TSFM | 4.18E-04 | 0.999582 |
| ENST00000585999 | TOB1 | 0.007645 | 0.992355 |
| ENST00000585999 | ANKRD13D | 0.002101 | 0.997899 |
| ENST00000585999 | MED20 | 0.004712 | -0.99529 |
| ENST00000585999 | CATG00000087963.1 | 0.002907 | -0.99709 |
| ENST00000585999 | EPHB6 | 0.001501 | -0.9985 |
| ENST00000585999 | CATG00000021838.1 | 0.003913 | 0.996087 |
| ENST00000585999 | TSFM | 4.18E-04 | 0.999582 |
| ENST00000562904 | EPB41L1 | 0.008751 | -0.99125 |
| ENST00000562904 | CACHD1 | 0.009122 | -0.99088 |
| ENST00000562904 | INSC | 0.00319 | -0.99681 |
| ENST00000562904 | MYH3 | 0.008868 | 0.991132 |
| ENST00000562904 | PCDHGA3 | 0.002795 | -0.9972 |
| ENST00000562904 | OAS1 | 0.001482 | -0.99852 |
| ENST00000562904 | DCD | 0.004383 | -0.99562 |
| ENST00000562904 | PAN3 | 0.005866 | 0.994134 |
| ENST00000562904 | CSNK1A1 | 7.19E-04 | 0.999281 |
| ENST00000562904 | ZNF587B | 2.29E-04 | 0.999771 |
| ENST00000562904 | ACP6 | 0.00882 | 0.99118 |
| ENST00000562904 | RPL17 | 0.001828 | 0.998172 |
| ENST00000562904 | ZNF37A | 0.009253 | 0.990747 |
| ENST00000562904 | WAPL | 0.002382 | 0.997618 |
| ENST00000562904 | GABPA | 0.007206 | -0.99279 |
| ENST00000454555 | ACTL7B | 0.008121 | 0.991879 |
| ENST00000454555 | TTC38 | 0.008595 | -0.9914 |
| ENST00000454555 | FBXL16 | 0.009432 | -0.99057 |
| ENST00000454555 | IGFL2 | 0.003953 | -0.99605 |
| ENST00000454555 | S100P | 0.007439 | 0.992561 |
| ENST00000608044 | C16orf45 | 0.007841 | -0.99216 |
| ENST00000608044 | PLAGL1 | 9.66E-04 | -0.99903 |
| ENST00000608044 | RHBDD3 | 0.003759 | -0.99624 |
| ENST00000608044 | AIFM3 | 0.00535 | -0.99465 |
| ENST00000608044 | VGLL3 | 0.007318 | 0.992682 |
| ENST00000608044 | MMP2 | 0.005471 | 0.994529 |
| ENST00000608044 | OCSTAMP | 0.003914 | 0.996086 |
| ENST00000608044 | IL1R2 | 0.001223 | 0.998777 |
| ENST00000608044 | CROT | 0.002277 | -0.99772 |
| ENST00000522127 | FBXL7 | 0.00525 | -0.99475 |
| ENST00000522127 | NUP54 | 0.005704 | -0.9943 |
| ENST00000522127 | HERC3 | 0.0012 | -0.9988 |
| ENST00000522127 | TDRD12 | 0.003602 | -0.9964 |
| ENST00000522127 | FTSJ1 | 0.009332 | -0.99067 |
| ENST00000522127 | FABP2 | 0.009418 | 0.990582 |
| ENST00000330551 | NR2F2 | 0.003867 | -0.99613 |
| ENST00000330551 | GBA | 0.001443 | -0.99856 |
| ENST00000330551 | RPL23A | 0.009207 | 0.990793 |
| ENST00000330551 | SLC7A4 | 0.003301 | -0.9967 |
| ENST00000330551 | SMPD1 | 9.07E-04 | -0.99909 |
| ENST00000330551 | SAMD1 | 0.009813 | 0.990187 |
| ENST00000330551 | CATG00000057824.1 | 0.001426 | -0.99857 |
| ENST00000330551 | WNT2 | 0.007725 | -0.99227 |
| ENST00000330551 | CDK11B | 0.003492 | 0.996508 |
| ENST00000330551 | TCTEX1D4 | 0.007641 | 0.992359 |
| ENST00000330551 | KLF6 | 0.00506 | 0.99494 |
| ENST00000330551 | MAP1LC3A | 8.86E-04 | -0.99911 |
| ENST00000330551 | LBP | 0.001303 | -0.9987 |
| ENST00000330551 | FAM53B | 0.00783 | 0.99217 |
| ENST00000330551 | SH3RF3 | 0.008172 | 0.991828 |
| ENST00000330551 | LDHB | 0.003894 | 0.996106 |
| ENST00000330551 | REG4 | 0.004852 | 0.995148 |
| ENST00000330551 | ACMSD | 0.00821 | 0.99179 |
| ENST00000330551 | NR2F2 | 0.003867 | -0.99613 |
| ENST00000330551 | GBA | 0.001443 | -0.99856 |
| ENST00000330551 | RPL23A | 0.009207 | 0.990793 |
| ENST00000330551 | SLC7A4 | 0.003301 | -0.9967 |
| ENST00000330551 | SMPD1 | 9.07E-04 | -0.99909 |
| ENST00000330551 | SAMD1 | 0.009813 | 0.990187 |
| ENST00000330551 | CATG00000057824.1 | 0.001426 | -0.99857 |
| ENST00000330551 | WNT2 | 0.007725 | -0.99227 |
| ENST00000330551 | CDK11B | 0.003492 | 0.996508 |
| ENST00000330551 | TCTEX1D4 | 0.007641 | 0.992359 |
| ENST00000330551 | KLF6 | 0.00506 | 0.99494 |
| ENST00000330551 | MAP1LC3A | 8.86E-04 | -0.99911 |
| ENST00000330551 | LBP | 0.001303 | -0.9987 |
| ENST00000330551 | FAM53B | 0.00783 | 0.99217 |
| ENST00000330551 | SH3RF3 | 0.008172 | 0.991828 |
| ENST00000330551 | LDHB | 0.003894 | 0.996106 |
| ENST00000330551 | REG4 | 0.004852 | 0.995148 |
| ENST00000330551 | ACMSD | 0.00821 | 0.99179 |
| ENCT00000137497 | WDR90 | 0.009128 | -0.99087 |
| ENCT00000137497 | SORCS2 | 0.005809 | -0.99419 |
| ENCT00000137497 | LHFPL6 | 0.009644 | 0.990356 |
| ENCT00000137497 | ARHGEF33 | 0.008654 | 0.991346 |
| T238382 | MYT1L | 0.005614 | 0.994386 |
| T238382 | MAPKBP1 | 0.008311 | 0.991689 |
| T238382 | TREML2 | 0.005583 | 0.994417 |
| T238382 | AC093157.1 | 0.00576 | -0.99424 |
| T238382 | ZPR1 | 0.001849 | 0.998151 |
| T238382 | FRAT1 | 0.005793 | -0.99421 |
| T238382 | OR10R2 | 0.009497 | 0.990503 |
| T018424 | SCAMP2 | 0.001943 | -0.99806 |
| T018424 | SLC24A5 | 0.009095 | 0.990905 |
| T018424 | TMC5 | 0.00436 | -0.99564 |
| T018424 | PEX14 | 0.004714 | -0.99529 |
| T018424 | XYLT2 | 0.003472 | -0.99653 |
| T018424 | IKZF4 | 2.42E-04 | -0.99976 |
| T018424 | MYH3 | 0.005338 | 0.994662 |
| T018424 | KCTD1 | 0.002938 | -0.99706 |
| T018424 | STRADB | 0.00689 | -0.99311 |
| T018424 | PURG | 0.004192 | -0.99581 |
| T018424 | CACNG6 | 0.002475 | -0.99753 |
| T018424 | PAN3 | 0.009577 | 0.990423 |
| T018424 | SHROOM3 | 0.003647 | -0.99635 |
| T018424 | INKA2 | 0.004452 | -0.99555 |
| T018424 | RAMP1 | 0.00266 | 0.99734 |
| T018424 | PARVG | 0.001915 | -0.99809 |
| T018424 | ABCA13 | 0.003715 | -0.99628 |
| T018424 | ZNF37A | 0.009593 | 0.990407 |
| T018424 | DICER1 | 0.009412 | 0.990588 |
| T018424 | YPEL5 | 0.004435 | -0.99557 |
| ENST00000452629 | INTS3 | 7.86E-04 | -0.99921 |
| ENST00000452629 | VPS41 | 0.003641 | -0.99636 |
| ENST00000452629 | TMEM43 | 0.00342 | 0.99658 |
| ENST00000552996 | FCN2 | 0.007708 | -0.99229 |
| ENST00000552996 | TRMT6 | 0.001947 | -0.99805 |
| ENST00000552996 | RECK | 0.003541 | -0.99646 |
| ENST00000552996 | EDC3 | 0.008613 | -0.99139 |
| ENST00000552996 | CATG00000035925.1 | 0.002701 | -0.9973 |
| ENST00000552996 | MYL3 | 0.006631 | -0.99337 |
| ENST00000552996 | NFE2L2 | 5.24E-04 | 0.999476 |
| ENST00000552996 | OSBPL5 | 0.008413 | -0.99159 |
| ENST00000552996 | SIVA1 | 0.005076 | 0.994924 |
| ENST00000552996 | CORO7-PAM16 | 0.001821 | 0.998179 |
| ENST00000552996 | SMIM6 | 0.002105 | -0.9979 |
| ENST00000559022 | CATG00000021869.1 | 0.001931 | -0.99807 |
| ENST00000559022 | PLEKHG2 | 0.002868 | 0.997132 |
| ENST00000559022 | KCNJ11 | 0.004198 | -0.9958 |
| ENST00000559022 | TSPAN32 | 0.006974 | 0.993026 |
| ENST00000559022 | IQANK1 | 0.001967 | -0.99803 |
| ENST00000559022 | SLPI | 0.002366 | -0.99763 |
| ENST00000559022 | C1QTNF9B | 0.007937 | -0.99206 |
| ENST00000559022 | KLRG2 | 0.004211 | 0.995789 |
| ENST00000559022 | UPP2 | 0.008664 | -0.99134 |
| ENST00000559022 | CATG00000021869.1 | 0.001931 | -0.99807 |
| ENST00000559022 | PLEKHG2 | 0.002868 | 0.997132 |
| ENST00000559022 | KCNJ11 | 0.004198 | -0.9958 |
| ENST00000559022 | TSPAN32 | 0.006974 | 0.993026 |
| ENST00000559022 | IQANK1 | 0.001967 | -0.99803 |
| ENST00000559022 | SLPI | 0.002366 | -0.99763 |
| ENST00000559022 | C1QTNF9B | 0.007937 | -0.99206 |
| ENST00000559022 | KLRG2 | 0.004211 | 0.995789 |
| ENST00000559022 | UPP2 | 0.008664 | -0.99134 |
| T305289 | NOC4L | 0.006501 | 0.993499 |
| T305289 | CATG00000080699.1 | 0.002301 | 0.997699 |
| T305289 | SLC24A5 | 0.009133 | -0.99087 |
| T305289 | TCOF1 | 0.004763 | 0.995237 |
| T305289 | SAMD13 | 0.005253 | 0.994747 |
| T305289 | ZDHHC16 | 2.84E-04 | -0.99972 |
| T305289 | CDKL2 | 0.008566 | 0.991434 |
| T305289 | MACROD2 | 0.001153 | 0.998847 |
| T305289 | CHST12 | 0 | 1 |
| T305289 | HIPK2 | 0.004794 | -0.99521 |
| T305289 | PCDHB8 | 0 | 1 |
| ENST00000521498 | NOC4L | 0.006501 | 0.993499 |
| ENST00000521498 | CATG00000080699.1 | 0.002301 | 0.997699 |
| ENST00000521498 | SLC24A5 | 0.009133 | -0.99087 |
| ENST00000521498 | TCOF1 | 0.004763 | 0.995237 |
| ENST00000521498 | SAMD13 | 0.005253 | 0.994747 |
| ENST00000521498 | ZDHHC16 | 2.84E-04 | -0.99972 |
| ENST00000521498 | CDKL2 | 0.008566 | 0.991434 |
| ENST00000521498 | MACROD2 | 0.001153 | 0.998847 |
| ENST00000521498 | CHST12 | 0 | 1 |
| ENST00000521498 | HIPK2 | 0.004794 | -0.99521 |
| ENST00000521498 | PCDHB8 | 0 | 1 |
| ENST00000432707 | RPS15 | 0.002066 | -0.99793 |
| ENST00000432707 | IL31RA | 0.006728 | -0.99327 |
| ENST00000432707 | SHISA6 | 0.009741 | 0.990259 |
| ENST00000432707 | FRAT1 | 0.008521 | 0.991479 |
| ENST00000432707 | RPS15 | 0.002066 | -0.99793 |
| ENST00000432707 | IL31RA | 0.006728 | -0.99327 |
| ENST00000432707 | SHISA6 | 0.009741 | 0.990259 |
| ENST00000432707 | FRAT1 | 0.008521 | 0.991479 |
| ENST00000432707 | RPS15 | 0.002066 | -0.99793 |
| ENST00000432707 | IL31RA | 0.006728 | -0.99327 |
| ENST00000432707 | SHISA6 | 0.009741 | 0.990259 |
| ENST00000432707 | FRAT1 | 0.008521 | 0.991479 |
| ENST00000432707 | RPS15 | 0.002066 | -0.99793 |
| ENST00000432707 | IL31RA | 0.006728 | -0.99327 |
| ENST00000432707 | SHISA6 | 0.009741 | 0.990259 |
| ENST00000432707 | FRAT1 | 0.008521 | 0.991479 |
| ENST00000432707 | RPS15 | 0.002066 | -0.99793 |
| ENST00000432707 | IL31RA | 0.006728 | -0.99327 |
| ENST00000432707 | SHISA6 | 0.009741 | 0.990259 |
| ENST00000432707 | FRAT1 | 0.008521 | 0.991479 |
| ENST00000432707 | RPS15 | 0.002066 | -0.99793 |
| ENST00000432707 | IL31RA | 0.006728 | -0.99327 |
| ENST00000432707 | SHISA6 | 0.009741 | 0.990259 |
| ENST00000432707 | FRAT1 | 0.008521 | 0.991479 |
| ENST00000569993 | ZC3H7B | 0.008697 | -0.9913 |
| ENST00000569993 | C1QTNF8 | 0.008856 | -0.99114 |
| ENST00000569993 | CRYM | 0.006007 | 0.993993 |
| T038065 | CATG00000067666.1 | 0.009562 | 0.990438 |
| T038065 | FABP2 | 0.005987 | 0.994013 |
| TCONS_00009990 | ECSIT | 0.002751 | -0.99725 |
| TCONS_00009990 | CATG00000056264.1 | 0.002207 | -0.99779 |
| TCONS_00009990 | SIRPG | 0.002668 | -0.99733 |
| TCONS_00009990 | CATG00000003494.1 | 0.005359 | -0.99464 |
| TCONS_00009990 | SSBP2 | 0.002013 | -0.99799 |
| TCONS_00009990 | CACHD1 | 0.007782 | -0.99222 |
| TCONS_00009990 | CHST8 | 7.74E-04 | -0.99923 |
| TCONS_00009990 | CLEC4G | 0.005017 | -0.99498 |
| TCONS_00009990 | TMC2 | 0.004441 | -0.99556 |
| TCONS_00009990 | CATG00000022188.1 | 0.001448 | -0.99855 |
| TCONS_00009990 | TPRX1 | 0.008476 | -0.99152 |
| TCONS_00009990 | RBM7 | 0.00404 | -0.99596 |
| TCONS_00009990 | SLC22A6 | 0.008552 | 0.991448 |
| TCONS_00009990 | SPATA31D1 | 0.003918 | -0.99608 |
| TCONS_00009990 | CATG00000038058.1 | 0.002161 | -0.99784 |
| TCONS_00009990 | ZC3H12D | 0.004178 | 0.995822 |
| TCONS_00009990 | INO80B | 0.005519 | -0.99448 |
| TCONS_00009990 | PLCL1 | 0.007034 | 0.992966 |
| TCONS_00009990 | TNFRSF13B | 0.004525 | 0.995475 |
| TCONS_00009990 | GABPA | 0.008172 | -0.99183 |
| TCONS_00009990 | FGF9 | 0.005216 | 0.994784 |
| ENST00000559148 | INPP5J | 6.72E-04 | -0.99933 |
| ENST00000559148 | RTN1 | 0.004913 | 0.995087 |
| ENST00000559148 | CNEP1R1 | 0.002663 | 0.997337 |
| ENST00000559148 | NAGLU | 0.009485 | -0.99051 |
| ENST00000559148 | FRG1 | 0.002617 | 0.997383 |
| ENST00000559148 | AP3M2 | 0.009544 | 0.990456 |
| ENST00000559148 | PHKA1 | 0.009829 | -0.99017 |
| ENST00000559148 | COA1 | 0.005976 | 0.994024 |
| ENST00000559148 | BMPER | 2.43E-04 | -0.99976 |
| ENST00000428573 | CATG00000021869.1 | 0.001845 | 0.998155 |
| ENST00000428573 | PLEKHG2 | 0.004536 | -0.99546 |
| ENST00000428573 | KCNJ11 | 0.002948 | 0.997052 |
| ENST00000428573 | TSPAN32 | 0.006458 | -0.99354 |
| ENST00000428573 | IQANK1 | 0.003568 | 0.996432 |
| ENST00000428573 | SLPI | 0.004006 | 0.995994 |
| ENST00000428573 | METTL3 | 0.007498 | 0.992502 |
| ENST00000428573 | C1QTNF9B | 0.007808 | 0.992192 |
| ENST00000428573 | KLRG2 | 0.006151 | -0.99385 |
| ENST00000428573 | UPP2 | 0.008257 | 0.991743 |
| ENST00000428573 | CATG00000021869.1 | 0.001845 | 0.998155 |
| ENST00000428573 | PLEKHG2 | 0.004536 | -0.99546 |
| ENST00000428573 | KCNJ11 | 0.002948 | 0.997052 |
| ENST00000428573 | TSPAN32 | 0.006458 | -0.99354 |
| ENST00000428573 | IQANK1 | 0.003568 | 0.996432 |
| ENST00000428573 | SLPI | 0.004006 | 0.995994 |
| ENST00000428573 | METTL3 | 0.007498 | 0.992502 |
| ENST00000428573 | C1QTNF9B | 0.007808 | 0.992192 |
| ENST00000428573 | KLRG2 | 0.006151 | -0.99385 |
| ENST00000428573 | UPP2 | 0.008257 | 0.991743 |
| ENST00000428573 | CATG00000021869.1 | 0.001845 | 0.998155 |
| ENST00000428573 | PLEKHG2 | 0.004536 | -0.99546 |
| ENST00000428573 | KCNJ11 | 0.002948 | 0.997052 |
| ENST00000428573 | TSPAN32 | 0.006458 | -0.99354 |
| ENST00000428573 | IQANK1 | 0.003568 | 0.996432 |
| ENST00000428573 | SLPI | 0.004006 | 0.995994 |
| ENST00000428573 | METTL3 | 0.007498 | 0.992502 |
| ENST00000428573 | C1QTNF9B | 0.007808 | 0.992192 |
| ENST00000428573 | KLRG2 | 0.006151 | -0.99385 |
| ENST00000428573 | UPP2 | 0.008257 | 0.991743 |
| ENST00000502435 | NOC4L | 0.006501 | 0.993499 |
| ENST00000502435 | CATG00000080699.1 | 0.002301 | 0.997699 |
| ENST00000502435 | SLC24A5 | 0.009133 | -0.99087 |
| ENST00000502435 | TCOF1 | 0.004763 | 0.995237 |
| ENST00000502435 | SAMD13 | 0.005253 | 0.994747 |
| ENST00000502435 | ZDHHC16 | 2.84E-04 | -0.99972 |
| ENST00000502435 | CDKL2 | 0.008566 | 0.991434 |
| ENST00000502435 | MACROD2 | 0.001153 | 0.998847 |
| ENST00000502435 | CHST12 | 0 | 1 |
| ENST00000502435 | HIPK2 | 0.004794 | -0.99521 |
| ENST00000502435 | PCDHB8 | 0 | 1 |
| ENST00000502435 | NOC4L | 0.006501 | 0.993499 |
| ENST00000502435 | CATG00000080699.1 | 0.002301 | 0.997699 |
| ENST00000502435 | SLC24A5 | 0.009133 | -0.99087 |
| ENST00000502435 | TCOF1 | 0.004763 | 0.995237 |
| ENST00000502435 | SAMD13 | 0.005253 | 0.994747 |
| ENST00000502435 | ZDHHC16 | 2.84E-04 | -0.99972 |
| ENST00000502435 | CDKL2 | 0.008566 | 0.991434 |
| ENST00000502435 | MACROD2 | 0.001153 | 0.998847 |
| ENST00000502435 | CHST12 | 0 | 1 |
| ENST00000502435 | HIPK2 | 0.004794 | -0.99521 |
| ENST00000502435 | PCDHB8 | 0 | 1 |
| ENST00000502435 | NOC4L | 0.006501 | 0.993499 |
| ENST00000502435 | CATG00000080699.1 | 0.002301 | 0.997699 |
| ENST00000502435 | SLC24A5 | 0.009133 | -0.99087 |
| ENST00000502435 | TCOF1 | 0.004763 | 0.995237 |
| ENST00000502435 | SAMD13 | 0.005253 | 0.994747 |
| ENST00000502435 | ZDHHC16 | 2.84E-04 | -0.99972 |
| ENST00000502435 | CDKL2 | 0.008566 | 0.991434 |
| ENST00000502435 | MACROD2 | 0.001153 | 0.998847 |
| ENST00000502435 | CHST12 | 0 | 1 |
| ENST00000502435 | HIPK2 | 0.004794 | -0.99521 |
| ENST00000502435 | PCDHB8 | 0 | 1 |
| ENST00000502435 | NOC4L | 0.006501 | 0.993499 |
| ENST00000502435 | CATG00000080699.1 | 0.002301 | 0.997699 |
| ENST00000502435 | SLC24A5 | 0.009133 | -0.99087 |
| ENST00000502435 | TCOF1 | 0.004763 | 0.995237 |
| ENST00000502435 | SAMD13 | 0.005253 | 0.994747 |
| ENST00000502435 | ZDHHC16 | 2.84E-04 | -0.99972 |
| ENST00000502435 | CDKL2 | 0.008566 | 0.991434 |
| ENST00000502435 | MACROD2 | 0.001153 | 0.998847 |
| ENST00000502435 | CHST12 | 0 | 1 |
| ENST00000502435 | HIPK2 | 0.004794 | -0.99521 |
| ENST00000502435 | PCDHB8 | 0 | 1 |
| ENST00000502435 | NOC4L | 0.006501 | 0.993499 |
| ENST00000502435 | CATG00000080699.1 | 0.002301 | 0.997699 |
| ENST00000502435 | SLC24A5 | 0.009133 | -0.99087 |
| ENST00000502435 | TCOF1 | 0.004763 | 0.995237 |
| ENST00000502435 | SAMD13 | 0.005253 | 0.994747 |
| ENST00000502435 | ZDHHC16 | 2.84E-04 | -0.99972 |
| ENST00000502435 | CDKL2 | 0.008566 | 0.991434 |
| ENST00000502435 | MACROD2 | 0.001153 | 0.998847 |
| ENST00000502435 | CHST12 | 0 | 1 |
| ENST00000502435 | HIPK2 | 0.004794 | -0.99521 |
| ENST00000502435 | PCDHB8 | 0 | 1 |
| ENST00000502435 | NOC4L | 0.006501 | 0.993499 |
| ENST00000502435 | CATG00000080699.1 | 0.002301 | 0.997699 |
| ENST00000502435 | SLC24A5 | 0.009133 | -0.99087 |
| ENST00000502435 | TCOF1 | 0.004763 | 0.995237 |
| ENST00000502435 | SAMD13 | 0.005253 | 0.994747 |
| ENST00000502435 | ZDHHC16 | 2.84E-04 | -0.99972 |
| ENST00000502435 | CDKL2 | 0.008566 | 0.991434 |
| ENST00000502435 | MACROD2 | 0.001153 | 0.998847 |
| ENST00000502435 | CHST12 | 0 | 1 |
| ENST00000502435 | HIPK2 | 0.004794 | -0.99521 |
| ENST00000502435 | PCDHB8 | 0 | 1 |
| ENST00000502435 | NOC4L | 0.006501 | 0.993499 |
| ENST00000502435 | CATG00000080699.1 | 0.002301 | 0.997699 |
| ENST00000502435 | SLC24A5 | 0.009133 | -0.99087 |
| ENST00000502435 | TCOF1 | 0.004763 | 0.995237 |
| ENST00000502435 | SAMD13 | 0.005253 | 0.994747 |
| ENST00000502435 | ZDHHC16 | 2.84E-04 | -0.99972 |
| ENST00000502435 | CDKL2 | 0.008566 | 0.991434 |
| ENST00000502435 | MACROD2 | 0.001153 | 0.998847 |
| ENST00000502435 | CHST12 | 0 | 1 |
| ENST00000502435 | HIPK2 | 0.004794 | -0.99521 |
| ENST00000502435 | PCDHB8 | 0 | 1 |
| ENST00000502435 | NOC4L | 0.006501 | 0.993499 |
| ENST00000502435 | CATG00000080699.1 | 0.002301 | 0.997699 |
| ENST00000502435 | SLC24A5 | 0.009133 | -0.99087 |
| ENST00000502435 | TCOF1 | 0.004763 | 0.995237 |
| ENST00000502435 | SAMD13 | 0.005253 | 0.994747 |
| ENST00000502435 | ZDHHC16 | 2.84E-04 | -0.99972 |
| ENST00000502435 | CDKL2 | 0.008566 | 0.991434 |
| ENST00000502435 | MACROD2 | 0.001153 | 0.998847 |
| ENST00000502435 | CHST12 | 0 | 1 |
| ENST00000502435 | HIPK2 | 0.004794 | -0.99521 |
| ENST00000502435 | PCDHB8 | 0 | 1 |
| ENST00000502435 | NOC4L | 0.006501 | 0.993499 |
| ENST00000502435 | CATG00000080699.1 | 0.002301 | 0.997699 |
| ENST00000502435 | SLC24A5 | 0.009133 | -0.99087 |
| ENST00000502435 | TCOF1 | 0.004763 | 0.995237 |
| ENST00000502435 | SAMD13 | 0.005253 | 0.994747 |
| ENST00000502435 | ZDHHC16 | 2.84E-04 | -0.99972 |
| ENST00000502435 | CDKL2 | 0.008566 | 0.991434 |
| ENST00000502435 | MACROD2 | 0.001153 | 0.998847 |
| ENST00000502435 | CHST12 | 0 | 1 |
| ENST00000502435 | HIPK2 | 0.004794 | -0.99521 |
| ENST00000502435 | PCDHB8 | 0 | 1 |
| ENST00000502435 | NOC4L | 0.006501 | 0.993499 |
| ENST00000502435 | CATG00000080699.1 | 0.002301 | 0.997699 |
| ENST00000502435 | SLC24A5 | 0.009133 | -0.99087 |
| ENST00000502435 | TCOF1 | 0.004763 | 0.995237 |
| ENST00000502435 | SAMD13 | 0.005253 | 0.994747 |
| ENST00000502435 | ZDHHC16 | 2.84E-04 | -0.99972 |
| ENST00000502435 | CDKL2 | 0.008566 | 0.991434 |
| ENST00000502435 | MACROD2 | 0.001153 | 0.998847 |
| ENST00000502435 | CHST12 | 0 | 1 |
| ENST00000502435 | HIPK2 | 0.004794 | -0.99521 |
| ENST00000502435 | PCDHB8 | 0 | 1 |
| HSALNT0288995 | GABRG1 | 0.009648 | -0.99035 |
| HSALNT0288995 | CEBPD | 0.003562 | -0.99644 |
| HSALNT0288995 | FOXR1 | 0.006339 | -0.99366 |
| HSALNT0289269 | HEXIM2 | 0.006793 | 0.993207 |
| HSALNT0289269 | FAR1 | 0.006459 | 0.993541 |
| HSALNT0289269 | MAPK12 | 0.003548 | -0.99645 |
| HSALNT0289269 | CAMP | 0.004597 | -0.9954 |
| HSALNT0289269 | TCN1 | 0.001747 | -0.99825 |
| HSALNT0289269 | CTNNBIP1 | 0.00899 | 0.99101 |
| HSALNT0289269 | HEXIM2 | 0.006793 | 0.993207 |
| HSALNT0289269 | FAR1 | 0.006459 | 0.993541 |
| HSALNT0289269 | MAPK12 | 0.003548 | -0.99645 |
| HSALNT0289269 | CAMP | 0.004597 | -0.9954 |
| HSALNT0289269 | TCN1 | 0.001747 | -0.99825 |
| HSALNT0289269 | CTNNBIP1 | 0.00899 | 0.99101 |
| HSALNT0289269 | HEXIM2 | 0.006793 | 0.993207 |
| HSALNT0289269 | FAR1 | 0.006459 | 0.993541 |
| HSALNT0289269 | MAPK12 | 0.003548 | -0.99645 |
| HSALNT0289269 | CAMP | 0.004597 | -0.9954 |
| HSALNT0289269 | TCN1 | 0.001747 | -0.99825 |
| HSALNT0289269 | CTNNBIP1 | 0.00899 | 0.99101 |
| HSALNT0289269 | HEXIM2 | 0.006793 | 0.993207 |
| HSALNT0289269 | FAR1 | 0.006459 | 0.993541 |
| HSALNT0289269 | MAPK12 | 0.003548 | -0.99645 |
| HSALNT0289269 | CAMP | 0.004597 | -0.9954 |
| HSALNT0289269 | TCN1 | 0.001747 | -0.99825 |
| HSALNT0289269 | CTNNBIP1 | 0.00899 | 0.99101 |
| HSALNT0289269 | HEXIM2 | 0.006793 | 0.993207 |
| HSALNT0289269 | FAR1 | 0.006459 | 0.993541 |
| HSALNT0289269 | MAPK12 | 0.003548 | -0.99645 |
| HSALNT0289269 | CAMP | 0.004597 | -0.9954 |
| HSALNT0289269 | TCN1 | 0.001747 | -0.99825 |
| HSALNT0289269 | CTNNBIP1 | 0.00899 | 0.99101 |
| ENST00000550541 | C16orf45 | 0.007508 | -0.99249 |
| ENST00000550541 | PLAGL1 | 0.008811 | -0.99119 |
| ENST00000550541 | MYO7A | 0.001673 | 0.998327 |
| ENST00000550541 | ABHD5 | 0.00474 | 0.99526 |
| ENST00000550541 | OCSTAMP | 0.004982 | 0.995018 |
| ENST00000550541 | SP140 | 0.003701 | -0.9963 |
| ENST00000550541 | IL1R2 | 0.001624 | 0.998376 |
| ENST00000550541 | CROT | 6.71E-04 | -0.99933 |
| ENST00000550541 | C16orf45 | 0.007508 | -0.99249 |
| ENST00000550541 | PLAGL1 | 0.008811 | -0.99119 |
| ENST00000550541 | MYO7A | 0.001673 | 0.998327 |
| ENST00000550541 | ABHD5 | 0.00474 | 0.99526 |
| ENST00000550541 | OCSTAMP | 0.004982 | 0.995018 |
| ENST00000550541 | SP140 | 0.003701 | -0.9963 |
| ENST00000550541 | IL1R2 | 0.001624 | 0.998376 |
| ENST00000550541 | CROT | 6.71E-04 | -0.99933 |
| GSE61474_TCONS_00017548 | EHD4 | 0.001894 | 0.998106 |
| GSE61474_TCONS_00017548 | MAPK12 | 0.008017 | 0.991983 |
| GSE61474_TCONS_00017548 | SLCO2A1 | 0.005647 | 0.994353 |
| GSE61474_TCONS_00017548 | CAMP | 0.00504 | 0.99496 |
| GSE61474_TCONS_00017548 | CEACAM8 | 0.001883 | 0.998117 |
| TCONS_00025559 | ZC3H7B | 0.007677 | -0.99232 |
| TCONS_00025559 | CATG00000061038.1 | 8.49E-04 | 0.999151 |
| TCONS_00025559 | CD19 | 0.004576 | 0.995424 |
| TCONS_00025559 | OR10R2 | 0.001957 | -0.99804 |
| TCONS_00025559 | DDIT4 | 4.39E-04 | 0.999561 |
| T348586 | DNAJC22 | 0.008612 | -0.99139 |
| T348586 | SPANXN4 | 0.003833 | -0.99617 |
| T348586 | MYT1L | 0.005446 | -0.99455 |
| T348586 | CATG00000074949.1 | 3.32E-04 | -0.99967 |
| T348586 | ATXN7 | 0.007838 | 0.992162 |
| T348586 | KCNE4 | 0.002102 | 0.997898 |
| NR_029410 | C11orf71 | 0.009732 | 0.990268 |
| NR_029410 | MYLK3 | 0.007685 | 0.992315 |
| NR_029410 | SIRT6 | 0.00205 | 0.99795 |
| ENST00000564127 | RHEB | 2.02E-04 | -0.9998 |
| ENST00000564127 | RIOX2 | 0.009932 | -0.99007 |
| ENST00000564127 | VWA5A | 0.005221 | -0.99478 |
| ENST00000564127 | UFC1 | 0.002845 | -0.99715 |
| ENST00000564127 | JCAD | 0.009616 | 0.990384 |
| ENST00000564127 | PLA1A | 0.001396 | 0.998604 |
| ENST00000564127 | TLR2 | 0.009751 | -0.99025 |
| ENST00000564127 | ANKS4B | 0.008389 | 0.991611 |
| ENST00000601250 | TMEM255B | 0.005207 | -0.99479 |
| ENST00000601250 | ASAP1 | 0.001062 | 0.998938 |
| ENST00000601250 | SUPV3L1 | 0.005605 | -0.99439 |
| ENST00000601250 | GPR156 | 0.00191 | 0.99809 |
| ENST00000601250 | FTCD | 8.59E-04 | 0.999141 |
| ENST00000601250 | ZNF77 | 0.007734 | -0.99227 |
| ENST00000601250 | INHBA | 0.006065 | 0.993935 |
| NR_147832 | DMAC2 | 0.007465 | -0.99254 |
| NR_147832 | GORASP1 | 0.004833 | 0.995167 |
| NR_147832 | TMEM155 | 0.008477 | -0.99152 |
| NR_147832 | CPLX1 | 0.004122 | 0.995878 |
| NR_147832 | KLHL35 | 0.00539 | 0.99461 |
| NR_147832 | SLC22A12 | 0.003625 | -0.99637 |
| NR_147832 | PLPPR4 | 0.009122 | 0.990878 |
| NR_147832 | FCHSD2 | 0.005279 | 0.994721 |
| NR_147832 | MT1F | 0.003876 | 0.996124 |
| NR_147832 | WWP1 | 0.003485 | 0.996515 |
| NR_147832 | APOBR | 0.006139 | 0.993861 |
| NR_147832 | ZNF579 | 0.008342 | -0.99166 |
| NR_147832 | OR13A1 | 0.005351 | 0.994649 |
| NR_147832 | KCNK10 | 0.009935 | -0.99006 |
| NR_147832 | ANKHD1 | 0.00452 | 0.99548 |
| NR_147832 | SMUG1 | 0.007899 | 0.992101 |
| NR_147832 | DHDH | 0.009645 | -0.99036 |
| NR_147832 | DCUN1D1 | 0.007514 | 0.992486 |
| NR_147832 | PAQR5 | 0.006686 | -0.99331 |
| NR_147832 | C3orf84 | 0.005347 | -0.99465 |
| NR_147832 | ATG4C | 0.002505 | -0.99749 |
| NR_147832 | DMAC2 | 0.007465 | -0.99254 |
| NR_147832 | GORASP1 | 0.004833 | 0.995167 |
| NR_147832 | TMEM155 | 0.008477 | -0.99152 |
| NR_147832 | CPLX1 | 0.004122 | 0.995878 |
| NR_147832 | KLHL35 | 0.00539 | 0.99461 |
| NR_147832 | SLC22A12 | 0.003625 | -0.99637 |
| NR_147832 | PLPPR4 | 0.009122 | 0.990878 |
| NR_147832 | FCHSD2 | 0.005279 | 0.994721 |
| NR_147832 | MT1F | 0.003876 | 0.996124 |
| NR_147832 | WWP1 | 0.003485 | 0.996515 |
| NR_147832 | APOBR | 0.006139 | 0.993861 |
| NR_147832 | ZNF579 | 0.008342 | -0.99166 |
| NR_147832 | OR13A1 | 0.005351 | 0.994649 |
| NR_147832 | KCNK10 | 0.009935 | -0.99006 |
| NR_147832 | ANKHD1 | 0.00452 | 0.99548 |
| NR_147832 | SMUG1 | 0.007899 | 0.992101 |
| NR_147832 | DHDH | 0.009645 | -0.99036 |
| NR_147832 | DCUN1D1 | 0.007514 | 0.992486 |
| NR_147832 | PAQR5 | 0.006686 | -0.99331 |
| NR_147832 | C3orf84 | 0.005347 | -0.99465 |
| NR_147832 | ATG4C | 0.002505 | -0.99749 |
| NR_147832 | DMAC2 | 0.007465 | -0.99254 |
| NR_147832 | GORASP1 | 0.004833 | 0.995167 |
| NR_147832 | TMEM155 | 0.008477 | -0.99152 |
| NR_147832 | CPLX1 | 0.004122 | 0.995878 |
| NR_147832 | KLHL35 | 0.00539 | 0.99461 |
| NR_147832 | SLC22A12 | 0.003625 | -0.99637 |
| NR_147832 | PLPPR4 | 0.009122 | 0.990878 |
| NR_147832 | FCHSD2 | 0.005279 | 0.994721 |
| NR_147832 | MT1F | 0.003876 | 0.996124 |
| NR_147832 | WWP1 | 0.003485 | 0.996515 |
| NR_147832 | APOBR | 0.006139 | 0.993861 |
| NR_147832 | ZNF579 | 0.008342 | -0.99166 |
| NR_147832 | OR13A1 | 0.005351 | 0.994649 |
| NR_147832 | KCNK10 | 0.009935 | -0.99006 |
| NR_147832 | ANKHD1 | 0.00452 | 0.99548 |
| NR_147832 | SMUG1 | 0.007899 | 0.992101 |
| NR_147832 | DHDH | 0.009645 | -0.99036 |
| NR_147832 | DCUN1D1 | 0.007514 | 0.992486 |
| NR_147832 | PAQR5 | 0.006686 | -0.99331 |
| NR_147832 | C3orf84 | 0.005347 | -0.99465 |
| NR_147832 | ATG4C | 0.002505 | -0.99749 |
| NR_147832 | DMAC2 | 0.007465 | -0.99254 |
| NR_147832 | GORASP1 | 0.004833 | 0.995167 |
| NR_147832 | TMEM155 | 0.008477 | -0.99152 |
| NR_147832 | CPLX1 | 0.004122 | 0.995878 |
| NR_147832 | KLHL35 | 0.00539 | 0.99461 |
| NR_147832 | SLC22A12 | 0.003625 | -0.99637 |
| NR_147832 | PLPPR4 | 0.009122 | 0.990878 |
| NR_147832 | FCHSD2 | 0.005279 | 0.994721 |
| NR_147832 | MT1F | 0.003876 | 0.996124 |
| NR_147832 | WWP1 | 0.003485 | 0.996515 |
| NR_147832 | APOBR | 0.006139 | 0.993861 |
| NR_147832 | ZNF579 | 0.008342 | -0.99166 |
| NR_147832 | OR13A1 | 0.005351 | 0.994649 |
| NR_147832 | KCNK10 | 0.009935 | -0.99006 |
| NR_147832 | ANKHD1 | 0.00452 | 0.99548 |
| NR_147832 | SMUG1 | 0.007899 | 0.992101 |
| NR_147832 | DHDH | 0.009645 | -0.99036 |
| NR_147832 | DCUN1D1 | 0.007514 | 0.992486 |
| NR_147832 | PAQR5 | 0.006686 | -0.99331 |
| NR_147832 | C3orf84 | 0.005347 | -0.99465 |
| NR_147832 | ATG4C | 0.002505 | -0.99749 |
| NR_147832 | DMAC2 | 0.007465 | -0.99254 |
| NR_147832 | GORASP1 | 0.004833 | 0.995167 |
| NR_147832 | TMEM155 | 0.008477 | -0.99152 |
| NR_147832 | CPLX1 | 0.004122 | 0.995878 |
| NR_147832 | KLHL35 | 0.00539 | 0.99461 |
| NR_147832 | SLC22A12 | 0.003625 | -0.99637 |
| NR_147832 | PLPPR4 | 0.009122 | 0.990878 |
| NR_147832 | FCHSD2 | 0.005279 | 0.994721 |
| NR_147832 | MT1F | 0.003876 | 0.996124 |
| NR_147832 | WWP1 | 0.003485 | 0.996515 |
| NR_147832 | APOBR | 0.006139 | 0.993861 |
| NR_147832 | ZNF579 | 0.008342 | -0.99166 |
| NR_147832 | OR13A1 | 0.005351 | 0.994649 |
| NR_147832 | KCNK10 | 0.009935 | -0.99006 |
| NR_147832 | ANKHD1 | 0.00452 | 0.99548 |
| NR_147832 | SMUG1 | 0.007899 | 0.992101 |
| NR_147832 | DHDH | 0.009645 | -0.99036 |
| NR_147832 | DCUN1D1 | 0.007514 | 0.992486 |
| NR_147832 | PAQR5 | 0.006686 | -0.99331 |
| NR_147832 | C3orf84 | 0.005347 | -0.99465 |
| NR_147832 | ATG4C | 0.002505 | -0.99749 |
| NR_147832 | DMAC2 | 0.007465 | -0.99254 |
| NR_147832 | GORASP1 | 0.004833 | 0.995167 |
| NR_147832 | TMEM155 | 0.008477 | -0.99152 |
| NR_147832 | CPLX1 | 0.004122 | 0.995878 |
| NR_147832 | KLHL35 | 0.00539 | 0.99461 |
| NR_147832 | SLC22A12 | 0.003625 | -0.99637 |
| NR_147832 | PLPPR4 | 0.009122 | 0.990878 |
| NR_147832 | FCHSD2 | 0.005279 | 0.994721 |
| NR_147832 | MT1F | 0.003876 | 0.996124 |
| NR_147832 | WWP1 | 0.003485 | 0.996515 |
| NR_147832 | APOBR | 0.006139 | 0.993861 |
| NR_147832 | ZNF579 | 0.008342 | -0.99166 |
| NR_147832 | OR13A1 | 0.005351 | 0.994649 |
| NR_147832 | KCNK10 | 0.009935 | -0.99006 |
| NR_147832 | ANKHD1 | 0.00452 | 0.99548 |
| NR_147832 | SMUG1 | 0.007899 | 0.992101 |
| NR_147832 | DHDH | 0.009645 | -0.99036 |
| NR_147832 | DCUN1D1 | 0.007514 | 0.992486 |
| NR_147832 | PAQR5 | 0.006686 | -0.99331 |
| NR_147832 | C3orf84 | 0.005347 | -0.99465 |
| NR_147832 | ATG4C | 0.002505 | -0.99749 |
| NR_147832 | DMAC2 | 0.007465 | -0.99254 |
| NR_147832 | GORASP1 | 0.004833 | 0.995167 |
| NR_147832 | TMEM155 | 0.008477 | -0.99152 |
| NR_147832 | CPLX1 | 0.004122 | 0.995878 |
| NR_147832 | KLHL35 | 0.00539 | 0.99461 |
| NR_147832 | SLC22A12 | 0.003625 | -0.99637 |
| NR_147832 | PLPPR4 | 0.009122 | 0.990878 |
| NR_147832 | FCHSD2 | 0.005279 | 0.994721 |
| NR_147832 | MT1F | 0.003876 | 0.996124 |
| NR_147832 | WWP1 | 0.003485 | 0.996515 |
| NR_147832 | APOBR | 0.006139 | 0.993861 |
| NR_147832 | ZNF579 | 0.008342 | -0.99166 |
| NR_147832 | OR13A1 | 0.005351 | 0.994649 |
| NR_147832 | KCNK10 | 0.009935 | -0.99006 |
| NR_147832 | ANKHD1 | 0.00452 | 0.99548 |
| NR_147832 | SMUG1 | 0.007899 | 0.992101 |
| NR_147832 | DHDH | 0.009645 | -0.99036 |
| NR_147832 | DCUN1D1 | 0.007514 | 0.992486 |
| NR_147832 | PAQR5 | 0.006686 | -0.99331 |
| NR_147832 | C3orf84 | 0.005347 | -0.99465 |
| NR_147832 | ATG4C | 0.002505 | -0.99749 |
| NR_147832 | DMAC2 | 0.007465 | -0.99254 |
| NR_147832 | GORASP1 | 0.004833 | 0.995167 |
| NR_147832 | TMEM155 | 0.008477 | -0.99152 |
| NR_147832 | CPLX1 | 0.004122 | 0.995878 |
| NR_147832 | KLHL35 | 0.00539 | 0.99461 |
| NR_147832 | SLC22A12 | 0.003625 | -0.99637 |
| NR_147832 | PLPPR4 | 0.009122 | 0.990878 |
| NR_147832 | FCHSD2 | 0.005279 | 0.994721 |
| NR_147832 | MT1F | 0.003876 | 0.996124 |
| NR_147832 | WWP1 | 0.003485 | 0.996515 |
| NR_147832 | APOBR | 0.006139 | 0.993861 |
| NR_147832 | ZNF579 | 0.008342 | -0.99166 |
| NR_147832 | OR13A1 | 0.005351 | 0.994649 |
| NR_147832 | KCNK10 | 0.009935 | -0.99006 |
| NR_147832 | ANKHD1 | 0.00452 | 0.99548 |
| NR_147832 | SMUG1 | 0.007899 | 0.992101 |
| NR_147832 | DHDH | 0.009645 | -0.99036 |
| NR_147832 | DCUN1D1 | 0.007514 | 0.992486 |
| NR_147832 | PAQR5 | 0.006686 | -0.99331 |
| NR_147832 | C3orf84 | 0.005347 | -0.99465 |
| NR_147832 | ATG4C | 0.002505 | -0.99749 |
| NR_147832 | DMAC2 | 0.007465 | -0.99254 |
| NR_147832 | GORASP1 | 0.004833 | 0.995167 |
| NR_147832 | TMEM155 | 0.008477 | -0.99152 |
| NR_147832 | CPLX1 | 0.004122 | 0.995878 |
| NR_147832 | KLHL35 | 0.00539 | 0.99461 |
| NR_147832 | SLC22A12 | 0.003625 | -0.99637 |
| NR_147832 | PLPPR4 | 0.009122 | 0.990878 |
| NR_147832 | FCHSD2 | 0.005279 | 0.994721 |
| NR_147832 | MT1F | 0.003876 | 0.996124 |
| NR_147832 | WWP1 | 0.003485 | 0.996515 |
| NR_147832 | APOBR | 0.006139 | 0.993861 |
| NR_147832 | ZNF579 | 0.008342 | -0.99166 |
| NR_147832 | OR13A1 | 0.005351 | 0.994649 |
| NR_147832 | KCNK10 | 0.009935 | -0.99006 |
| NR_147832 | ANKHD1 | 0.00452 | 0.99548 |
| NR_147832 | SMUG1 | 0.007899 | 0.992101 |
| NR_147832 | DHDH | 0.009645 | -0.99036 |
| NR_147832 | DCUN1D1 | 0.007514 | 0.992486 |
| NR_147832 | PAQR5 | 0.006686 | -0.99331 |
| NR_147832 | C3orf84 | 0.005347 | -0.99465 |
| NR_147832 | ATG4C | 0.002505 | -0.99749 |
| NR_147832 | DMAC2 | 0.007465 | -0.99254 |
| NR_147832 | GORASP1 | 0.004833 | 0.995167 |
| NR_147832 | TMEM155 | 0.008477 | -0.99152 |
| NR_147832 | CPLX1 | 0.004122 | 0.995878 |
| NR_147832 | KLHL35 | 0.00539 | 0.99461 |
| NR_147832 | SLC22A12 | 0.003625 | -0.99637 |
| NR_147832 | PLPPR4 | 0.009122 | 0.990878 |
| NR_147832 | FCHSD2 | 0.005279 | 0.994721 |
| NR_147832 | MT1F | 0.003876 | 0.996124 |
| NR_147832 | WWP1 | 0.003485 | 0.996515 |
| NR_147832 | APOBR | 0.006139 | 0.993861 |
| NR_147832 | ZNF579 | 0.008342 | -0.99166 |
| NR_147832 | OR13A1 | 0.005351 | 0.994649 |
| NR_147832 | KCNK10 | 0.009935 | -0.99006 |
| NR_147832 | ANKHD1 | 0.00452 | 0.99548 |
| NR_147832 | SMUG1 | 0.007899 | 0.992101 |
| NR_147832 | DHDH | 0.009645 | -0.99036 |
| NR_147832 | DCUN1D1 | 0.007514 | 0.992486 |
| NR_147832 | PAQR5 | 0.006686 | -0.99331 |
| NR_147832 | C3orf84 | 0.005347 | -0.99465 |
| NR_147832 | ATG4C | 0.002505 | -0.99749 |
| NR_147832 | DMAC2 | 0.007465 | -0.99254 |
| NR_147832 | GORASP1 | 0.004833 | 0.995167 |
| NR_147832 | TMEM155 | 0.008477 | -0.99152 |
| NR_147832 | CPLX1 | 0.004122 | 0.995878 |
| NR_147832 | KLHL35 | 0.00539 | 0.99461 |
| NR_147832 | SLC22A12 | 0.003625 | -0.99637 |
| NR_147832 | PLPPR4 | 0.009122 | 0.990878 |
| NR_147832 | FCHSD2 | 0.005279 | 0.994721 |
| NR_147832 | MT1F | 0.003876 | 0.996124 |
| NR_147832 | WWP1 | 0.003485 | 0.996515 |
| NR_147832 | APOBR | 0.006139 | 0.993861 |
| NR_147832 | ZNF579 | 0.008342 | -0.99166 |
| NR_147832 | OR13A1 | 0.005351 | 0.994649 |
| NR_147832 | KCNK10 | 0.009935 | -0.99006 |
| NR_147832 | ANKHD1 | 0.00452 | 0.99548 |
| NR_147832 | SMUG1 | 0.007899 | 0.992101 |
| NR_147832 | DHDH | 0.009645 | -0.99036 |
| NR_147832 | DCUN1D1 | 0.007514 | 0.992486 |
| NR_147832 | PAQR5 | 0.006686 | -0.99331 |
| NR_147832 | C3orf84 | 0.005347 | -0.99465 |
| NR_147832 | ATG4C | 0.002505 | -0.99749 |
| NR_147832 | DMAC2 | 0.007465 | -0.99254 |
| NR_147832 | GORASP1 | 0.004833 | 0.995167 |
| NR_147832 | TMEM155 | 0.008477 | -0.99152 |
| NR_147832 | CPLX1 | 0.004122 | 0.995878 |
| NR_147832 | KLHL35 | 0.00539 | 0.99461 |
| NR_147832 | SLC22A12 | 0.003625 | -0.99637 |
| NR_147832 | PLPPR4 | 0.009122 | 0.990878 |
| NR_147832 | FCHSD2 | 0.005279 | 0.994721 |
| NR_147832 | MT1F | 0.003876 | 0.996124 |
| NR_147832 | WWP1 | 0.003485 | 0.996515 |
| NR_147832 | APOBR | 0.006139 | 0.993861 |
| NR_147832 | ZNF579 | 0.008342 | -0.99166 |
| NR_147832 | OR13A1 | 0.005351 | 0.994649 |
| NR_147832 | KCNK10 | 0.009935 | -0.99006 |
| NR_147832 | ANKHD1 | 0.00452 | 0.99548 |
| NR_147832 | SMUG1 | 0.007899 | 0.992101 |
| NR_147832 | DHDH | 0.009645 | -0.99036 |
| NR_147832 | DCUN1D1 | 0.007514 | 0.992486 |
| NR_147832 | PAQR5 | 0.006686 | -0.99331 |
| NR_147832 | C3orf84 | 0.005347 | -0.99465 |
| NR_147832 | ATG4C | 0.002505 | -0.99749 |
| NR_147832 | DMAC2 | 0.007465 | -0.99254 |
| NR_147832 | GORASP1 | 0.004833 | 0.995167 |
| NR_147832 | TMEM155 | 0.008477 | -0.99152 |
| NR_147832 | CPLX1 | 0.004122 | 0.995878 |
| NR_147832 | KLHL35 | 0.00539 | 0.99461 |
| NR_147832 | SLC22A12 | 0.003625 | -0.99637 |
| NR_147832 | PLPPR4 | 0.009122 | 0.990878 |
| NR_147832 | FCHSD2 | 0.005279 | 0.994721 |
| NR_147832 | MT1F | 0.003876 | 0.996124 |
| NR_147832 | WWP1 | 0.003485 | 0.996515 |
| NR_147832 | APOBR | 0.006139 | 0.993861 |
| NR_147832 | ZNF579 | 0.008342 | -0.99166 |
| NR_147832 | OR13A1 | 0.005351 | 0.994649 |
| NR_147832 | KCNK10 | 0.009935 | -0.99006 |
| NR_147832 | ANKHD1 | 0.00452 | 0.99548 |
| NR_147832 | SMUG1 | 0.007899 | 0.992101 |
| NR_147832 | DHDH | 0.009645 | -0.99036 |
| NR_147832 | DCUN1D1 | 0.007514 | 0.992486 |
| NR_147832 | PAQR5 | 0.006686 | -0.99331 |
| NR_147832 | C3orf84 | 0.005347 | -0.99465 |
| NR_147832 | ATG4C | 0.002505 | -0.99749 |
| NR_147832 | DMAC2 | 0.007465 | -0.99254 |
| NR_147832 | GORASP1 | 0.004833 | 0.995167 |
| NR_147832 | TMEM155 | 0.008477 | -0.99152 |
| NR_147832 | CPLX1 | 0.004122 | 0.995878 |
| NR_147832 | KLHL35 | 0.00539 | 0.99461 |
| NR_147832 | SLC22A12 | 0.003625 | -0.99637 |
| NR_147832 | PLPPR4 | 0.009122 | 0.990878 |
| NR_147832 | FCHSD2 | 0.005279 | 0.994721 |
| NR_147832 | MT1F | 0.003876 | 0.996124 |
| NR_147832 | WWP1 | 0.003485 | 0.996515 |
| NR_147832 | APOBR | 0.006139 | 0.993861 |
| NR_147832 | ZNF579 | 0.008342 | -0.99166 |
| NR_147832 | OR13A1 | 0.005351 | 0.994649 |
| NR_147832 | KCNK10 | 0.009935 | -0.99006 |
| NR_147832 | ANKHD1 | 0.00452 | 0.99548 |
| NR_147832 | SMUG1 | 0.007899 | 0.992101 |
| NR_147832 | DHDH | 0.009645 | -0.99036 |
| NR_147832 | DCUN1D1 | 0.007514 | 0.992486 |
| NR_147832 | PAQR5 | 0.006686 | -0.99331 |
| NR_147832 | C3orf84 | 0.005347 | -0.99465 |
| NR_147832 | ATG4C | 0.002505 | -0.99749 |
| NR_147832 | DMAC2 | 0.007465 | -0.99254 |
| NR_147832 | GORASP1 | 0.004833 | 0.995167 |
| NR_147832 | TMEM155 | 0.008477 | -0.99152 |
| NR_147832 | CPLX1 | 0.004122 | 0.995878 |
| NR_147832 | KLHL35 | 0.00539 | 0.99461 |
| NR_147832 | SLC22A12 | 0.003625 | -0.99637 |
| NR_147832 | PLPPR4 | 0.009122 | 0.990878 |
| NR_147832 | FCHSD2 | 0.005279 | 0.994721 |
| NR_147832 | MT1F | 0.003876 | 0.996124 |
| NR_147832 | WWP1 | 0.003485 | 0.996515 |
| NR_147832 | APOBR | 0.006139 | 0.993861 |
| NR_147832 | ZNF579 | 0.008342 | -0.99166 |
| NR_147832 | OR13A1 | 0.005351 | 0.994649 |
| NR_147832 | KCNK10 | 0.009935 | -0.99006 |
| NR_147832 | ANKHD1 | 0.00452 | 0.99548 |
| NR_147832 | SMUG1 | 0.007899 | 0.992101 |
| NR_147832 | DHDH | 0.009645 | -0.99036 |
| NR_147832 | DCUN1D1 | 0.007514 | 0.992486 |
| NR_147832 | PAQR5 | 0.006686 | -0.99331 |
| NR_147832 | C3orf84 | 0.005347 | -0.99465 |
| NR_147832 | ATG4C | 0.002505 | -0.99749 |
| ENST00000520032 | DUSP9 | 0.009704 | -0.9903 |
| ENST00000520032 | PZP | 0.003072 | 0.996928 |
| ENST00000520032 | CFAP77 | 0.009082 | 0.990918 |
| ENST00000520032 | ALS2CR12 | 0.004732 | -0.99527 |
| T087531 | PTPN6 | 0.005641 | -0.99436 |
| T087531 | UBB | 0.007483 | 0.992517 |
| T087531 | PXDC1 | 0.00208 | 0.99792 |
| T087531 | EVC2 | 0.004053 | 0.995947 |
| T087531 | MYCN | 0.00356 | 0.99644 |
| T087531 | SPSB2 | 0.003579 | 0.996421 |
| T087531 | PPP1R3E | 0.007113 | -0.99289 |
| ENST00000399196 | CEP170 | 0.003928 | 0.996072 |
| ENST00000399196 | EXOC4 | 0.004242 | -0.99576 |
| ENST00000399196 | TRMT11 | 0.002675 | 0.997325 |
| ENST00000399196 | ADGRE1 | 5.85E-04 | 0.999415 |
| ENST00000399196 | TMEM132B | 0.006079 | -0.99392 |
| ENST00000399196 | CDIP1 | 0.006738 | 0.993262 |
| ENST00000399196 | DBI | 0.007677 | 0.992323 |
| ENST00000399196 | SEC14L6 | 0.002168 | -0.99783 |
| ENST00000399196 | IL4I1 | 0.009613 | 0.990387 |
| ENST00000399196 | NEMP2 | 0.002094 | -0.99791 |
| ENST00000399196 | NEFM | 0.001129 | 0.998871 |
| ENST00000399196 | SLC39A12 | 0.009787 | -0.99021 |
| ENST00000399196 | PDE3B | 0.006489 | 0.993511 |
| ENST00000399196 | DEFA6 | 0.009022 | -0.99098 |
| ENST00000399196 | CATG00000027020.1 | 0.004713 | -0.99529 |
| ENST00000462717 | DNAJC6 | 0.006007 | -0.99399 |
| ENST00000462717 | LGALS1 | 3.73E-04 | 0.999627 |
| ENST00000462717 | SPACA9 | 0.00412 | -0.99588 |
| ENST00000462717 | DUSP2 | 0.002628 | 0.997372 |
| ENST00000462717 | DNAJC6 | 0.006007 | -0.99399 |
| ENST00000462717 | LGALS1 | 3.73E-04 | 0.999627 |
| ENST00000462717 | SPACA9 | 0.00412 | -0.99588 |
| ENST00000462717 | DUSP2 | 0.002628 | 0.997372 |
| ENST00000462717 | DNAJC6 | 0.006007 | -0.99399 |
| ENST00000462717 | LGALS1 | 3.73E-04 | 0.999627 |
| ENST00000462717 | SPACA9 | 0.00412 | -0.99588 |
| ENST00000462717 | DUSP2 | 0.002628 | 0.997372 |
| ENST00000462717 | DNAJC6 | 0.006007 | -0.99399 |
| ENST00000462717 | LGALS1 | 3.73E-04 | 0.999627 |
| ENST00000462717 | SPACA9 | 0.00412 | -0.99588 |
| ENST00000462717 | DUSP2 | 0.002628 | 0.997372 |
| ENST00000462717 | DNAJC6 | 0.006007 | -0.99399 |
| ENST00000462717 | LGALS1 | 3.73E-04 | 0.999627 |
| ENST00000462717 | SPACA9 | 0.00412 | -0.99588 |
| ENST00000462717 | DUSP2 | 0.002628 | 0.997372 |
| ENST00000608173 | GRINA | 0.003736 | -0.99626 |
| ENST00000608173 | RSL1D1 | 0.008349 | 0.991651 |
| ENST00000608173 | PLIN4 | 0.007723 | -0.99228 |
| ENST00000608173 | LILRB1 | 0.004614 | 0.995386 |
| ENST00000608173 | RASL10A | 0.005728 | 0.994272 |
| ENST00000608173 | CXXC1 | 0.009267 | 0.990733 |
| ENST00000608173 | TBX3 | 0.007638 | -0.99236 |
| ENST00000608173 | AHSP | 0.006458 | -0.99354 |
| ENST00000608173 | NFX1 | 0.00639 | 0.99361 |
| ENST00000608173 | CATG00000012021.1 | 0.002731 | 0.997269 |
| ENST00000608173 | PEA15 | 0.006164 | -0.99384 |
| ENST00000608173 | ESRRB | 0.00344 | -0.99656 |
| ENST00000608173 | IFT122 | 0.001734 | -0.99827 |
| ENST00000608173 | SIGLEC7 | 0.00104 | -0.99896 |
| ENST00000608173 | WNT8B | 0.008062 | -0.99194 |
| ENST00000608173 | PTPN23 | 0.006434 | -0.99357 |
| ENST00000608173 | UNC5C | 0.008317 | 0.991683 |
| ENST00000608173 | AGO2 | 0.003688 | -0.99631 |
| ENST00000608173 | HPS1 | 0.007955 | -0.99204 |
| ENST00000608173 | FAM151A | 0.002742 | -0.99726 |
| ENST00000534492 | NFATC2 | 0.003672 | 0.996328 |
| ENST00000534492 | ACER1 | 0.00603 | -0.99397 |
| ENST00000534492 | WDR60 | 0.007343 | 0.992657 |
| T160264 | ELOF1 | 0.008322 | 0.991678 |
| T160264 | WNT16 | 0.009953 | 0.990047 |
| T160264 | GGCT | 0.00532 | -0.99468 |
| T160264 | PCP4 | 0.007766 | 0.992234 |
| T160264 | TMEM242 | 0.001069 | -0.99893 |
| T160264 | NLRP9 | 0.006947 | 0.993053 |
| T160264 | DHODH | 0.008328 | -0.99167 |
| T160264 | PRPF8 | 0.003669 | -0.99633 |
| T160264 | UNC45A | 0.003289 | 0.996711 |
| T160264 | CCDC149 | 0.003064 | 0.996936 |
| T160264 | THOC5 | 0.00245 | 0.99755 |
| T160264 | TUBGCP4 | 0.005937 | 0.994063 |
| T160264 | RACK1 | 0.003608 | 0.996392 |
| T160264 | CHCHD6 | 8.49E-04 | 0.999151 |
| T160264 | TMEM51 | 0.001485 | 0.998515 |
| T160264 | CEP290 | 0.006599 | 0.993401 |
| T160264 | JDP2 | 0.009989 | 0.990011 |
| T160264 | MCM3AP | 0.005312 | 0.994688 |
| T160264 | RPUSD2 | 0.002797 | -0.9972 |
| T160264 | ZNF107 | 0.005562 | -0.99444 |
| T160264 | CEP63 | 2.95E-04 | -0.99971 |
| ENCT00000402323 | VWA5A | 5.43E-04 | -0.99946 |
| ENCT00000402323 | PLA1A | 0.008762 | 0.991238 |
| ENCT00000402323 | TLR2 | 0.001043 | -0.99896 |
| ENCT00000402323 | CATG00000107403.1 | 0.002179 | 0.997821 |
| ENST00000609942 | TAS2R42 | 0.008971 | -0.99103 |
| ENST00000609942 | GRINA | 0.001269 | -0.99873 |
| ENST00000609942 | PLIN4 | 0.004226 | -0.99577 |
| ENST00000609942 | LILRB1 | 0.001827 | 0.998173 |
| ENST00000609942 | RASL10A | 0.002855 | 0.997145 |
| ENST00000609942 | CXXC1 | 0.006253 | 0.993747 |
| ENST00000609942 | TBX3 | 0.00791 | -0.99209 |
| ENST00000609942 | KRT83 | 0.009505 | -0.9905 |
| ENST00000609942 | NFX1 | 0.00307 | 0.99693 |
| ENST00000609942 | CATG00000012021.1 | 0.002907 | 0.997093 |
| ENST00000609942 | PEA15 | 0.00289 | -0.99711 |
| ENST00000609942 | ESRRB | 0.002406 | -0.99759 |
| ENST00000609942 | IFT122 | 3.24E-04 | -0.99968 |
| ENST00000609942 | SIGLEC7 | 9.73E-04 | -0.99903 |
| ENST00000609942 | WNT8B | 0.004016 | -0.99598 |
| ENST00000609942 | PTPN23 | 0.00794 | -0.99206 |
| ENST00000609942 | UNC5C | 0.004262 | 0.995738 |
| ENST00000609942 | AGO2 | 0.003224 | -0.99678 |
| ENST00000609942 | HPS1 | 0.003877 | -0.99612 |
| ENST00000609942 | CCDC174 | 0.008489 | 0.991511 |
| ENST00000609942 | FAM151A | 0.001805 | -0.9982 |
| ENST00000609942 | TAS2R42 | 0.008971 | -0.99103 |
| ENST00000609942 | GRINA | 0.001269 | -0.99873 |
| ENST00000609942 | PLIN4 | 0.004226 | -0.99577 |
| ENST00000609942 | LILRB1 | 0.001827 | 0.998173 |
| ENST00000609942 | RASL10A | 0.002855 | 0.997145 |
| ENST00000609942 | CXXC1 | 0.006253 | 0.993747 |
| ENST00000609942 | TBX3 | 0.00791 | -0.99209 |
| ENST00000609942 | KRT83 | 0.009505 | -0.9905 |
| ENST00000609942 | NFX1 | 0.00307 | 0.99693 |
| ENST00000609942 | CATG00000012021.1 | 0.002907 | 0.997093 |
| ENST00000609942 | PEA15 | 0.00289 | -0.99711 |
| ENST00000609942 | ESRRB | 0.002406 | -0.99759 |
| ENST00000609942 | IFT122 | 3.24E-04 | -0.99968 |
| ENST00000609942 | SIGLEC7 | 9.73E-04 | -0.99903 |
| ENST00000609942 | WNT8B | 0.004016 | -0.99598 |
| ENST00000609942 | PTPN23 | 0.00794 | -0.99206 |
| ENST00000609942 | UNC5C | 0.004262 | 0.995738 |
| ENST00000609942 | AGO2 | 0.003224 | -0.99678 |
| ENST00000609942 | HPS1 | 0.003877 | -0.99612 |
| ENST00000609942 | CCDC174 | 0.008489 | 0.991511 |
| ENST00000609942 | FAM151A | 0.001805 | -0.9982 |
| ENST00000609942 | TAS2R42 | 0.008971 | -0.99103 |
| ENST00000609942 | GRINA | 0.001269 | -0.99873 |
| ENST00000609942 | PLIN4 | 0.004226 | -0.99577 |
| ENST00000609942 | LILRB1 | 0.001827 | 0.998173 |
| ENST00000609942 | RASL10A | 0.002855 | 0.997145 |
| ENST00000609942 | CXXC1 | 0.006253 | 0.993747 |
| ENST00000609942 | TBX3 | 0.00791 | -0.99209 |
| ENST00000609942 | KRT83 | 0.009505 | -0.9905 |
| ENST00000609942 | NFX1 | 0.00307 | 0.99693 |
| ENST00000609942 | CATG00000012021.1 | 0.002907 | 0.997093 |
| ENST00000609942 | PEA15 | 0.00289 | -0.99711 |
| ENST00000609942 | ESRRB | 0.002406 | -0.99759 |
| ENST00000609942 | IFT122 | 3.24E-04 | -0.99968 |
| ENST00000609942 | SIGLEC7 | 9.73E-04 | -0.99903 |
| ENST00000609942 | WNT8B | 0.004016 | -0.99598 |
| ENST00000609942 | PTPN23 | 0.00794 | -0.99206 |
| ENST00000609942 | UNC5C | 0.004262 | 0.995738 |
| ENST00000609942 | AGO2 | 0.003224 | -0.99678 |
| ENST00000609942 | HPS1 | 0.003877 | -0.99612 |
| ENST00000609942 | CCDC174 | 0.008489 | 0.991511 |
| ENST00000609942 | FAM151A | 0.001805 | -0.9982 |
| ENST00000613652 | RIPK1 | 0.006379 | 0.993621 |
| ENST00000613652 | NAPA | 0.005012 | -0.99499 |
| ENST00000613652 | P3H3 | 0.001202 | 0.998798 |
| ENST00000613652 | BTBD2 | 0.009587 | -0.99041 |
| ENST00000613652 | HDHD5 | 0.003371 | 0.996629 |
| ENST00000613652 | UBB | 0.006206 | -0.99379 |
| ENST00000613652 | PXDC1 | 0.006111 | -0.99389 |
| ENST00000613652 | CATG00000110054.1 | 0.007265 | -0.99274 |
| ENST00000613652 | TMOD1 | 0.006277 | -0.99372 |
| ENST00000613652 | BHLHB9 | 0.008054 | -0.99195 |
| ENST00000613652 | MYCN | 0.005773 | -0.99423 |
| ENST00000613652 | YY2 | 0.003504 | 0.996496 |
| ENST00000613652 | ODF3B | 0.004635 | -0.99536 |
| ENST00000613652 | RRP9 | 0.008931 | -0.99107 |
| ENST00000613652 | UBN2 | 7.60E-05 | 0.999924 |
| ENST00000613652 | CATG00000020284.1 | 0.002494 | 0.997506 |
| ENST00000447307 | SUPV3L1 | 0.004273 | -0.99573 |
| ENST00000447307 | GPR156 | 0.009537 | 0.990463 |
| ENST00000447307 | UBE2V1 | 0.001246 | -0.99875 |
| ENST00000447307 | FTCD | 0.009913 | 0.990087 |
| ENST00000447307 | VGLL3 | 0.00565 | 0.99435 |
| ENST00000447307 | FAM186A | 0.00284 | 0.99716 |
| ENST00000447307 | PGAM5 | 0.006119 | 0.993881 |
| T170641 | ECSIT | 0.008532 | -0.99147 |
| T170641 | SIRPG | 0.008308 | -0.99169 |
| T170641 | AHSP | 0.006424 | -0.99358 |
| T170641 | AC092073.1 | 0.001972 | -0.99803 |
| T170641 | FRG1 | 0.009271 | 0.990729 |
| T170641 | TGFBR3 | 3.81E-04 | -0.99962 |
| T170641 | SLC22A6 | 0.00236 | 0.99764 |
| T170641 | COA1 | 0.004925 | 0.995075 |
| T170641 | COA5 | 0.001014 | 0.998986 |
| T170641 | SLC6A5 | 0.008313 | -0.99169 |
| T170641 | RRBP1 | 0.002 | 0.998 |
| T170641 | MORN3 | 9.92E-04 | 0.999008 |
| T170641 | VPS13A | 0.001591 | -0.99841 |
| T170641 | FGF9 | 0.006882 | 0.993118 |
| T170641 | CAPN15 | 0.004106 | -0.99589 |
| ENST00000442716 | SPANXN4 | 0.003587 | -0.99641 |
| ENST00000442716 | CD19 | 0.004027 | 0.995973 |
| ENST00000442716 | RELL1 | 3.16E-04 | 0.999684 |
| ENST00000442716 | ANKS3 | 0.007504 | 0.992496 |
| ENST00000442716 | ATXN7 | 0.006932 | 0.993068 |
| ENST00000442716 | ASPRV1 | 0.008148 | -0.99185 |
| ENST00000422022 | NOC4L | 0.008827 | -0.99117 |
| ENST00000422022 | CATG00000080699.1 | 0.006593 | -0.99341 |
| ENST00000422022 | SLC24A5 | 0.001729 | 0.998271 |
| ENST00000422022 | TCOF1 | 2.25E-04 | -0.99978 |
| ENST00000422022 | MYD88 | 0.008653 | 0.991347 |
| ENST00000422022 | FSCB | 0.007747 | -0.99225 |
| ENST00000422022 | SAMD13 | 4.50E-04 | -0.99955 |
| ENST00000422022 | ZDHHC16 | 0.002789 | 0.997211 |
| ENST00000422022 | MACROD2 | 0.001261 | -0.99874 |
| ENST00000422022 | PURG | 0.004658 | -0.99534 |
| ENST00000422022 | CACNG6 | 0.008386 | -0.99161 |
| ENST00000422022 | CHST12 | 0.00292 | -0.99708 |
| ENST00000422022 | PCDHB8 | 0.00292 | -0.99708 |
| NR_109780 | DNAJC6 | 0.00517 | 0.99483 |
| NR_109780 | MFAP3L | 0.009231 | 0.990769 |
| NR_109780 | LGALS1 | 6.27E-04 | -0.99937 |
| NR_109780 | SPACA9 | 0.003463 | 0.996537 |
| NR_109780 | DUSP2 | 0.003057 | -0.99694 |
| ENST00000630229 | LGALS1 | 0.00867 | 0.99133 |
| ENST00000630229 | DUSP2 | 0.008677 | 0.991323 |
| ENST00000630229 | LGALS1 | 0.00867 | 0.99133 |
| ENST00000630229 | DUSP2 | 0.008677 | 0.991323 |
| ENST00000630229 | LGALS1 | 0.00867 | 0.99133 |
| ENST00000630229 | DUSP2 | 0.008677 | 0.991323 |
| ENST00000630229 | LGALS1 | 0.00867 | 0.99133 |
| ENST00000630229 | DUSP2 | 0.008677 | 0.991323 |
| ENST00000630229 | LGALS1 | 0.00867 | 0.99133 |
| ENST00000630229 | DUSP2 | 0.008677 | 0.991323 |
| ENST00000630229 | LGALS1 | 0.00867 | 0.99133 |
| ENST00000630229 | DUSP2 | 0.008677 | 0.991323 |
| ENST00000428335 | SIRPG | 0.006678 | 0.993322 |
| ENST00000428335 | GSTA4 | 0.009209 | 0.990791 |
| ENST00000428335 | TBX3 | 0.002834 | 0.997166 |
| ENST00000428335 | AHSP | 0.001623 | 0.998377 |
| ENST00000428335 | KRT83 | 0.005081 | 0.994919 |
| ENST00000428335 | AC092073.1 | 0.003864 | 0.996136 |
| ENST00000428335 | CATG00000022188.1 | 0.009404 | 0.990596 |
| ENST00000428335 | CATG00000012021.1 | 0.003927 | -0.99607 |
| ENST00000428335 | TGFBR3 | 0.005973 | 0.994027 |
| ENST00000428335 | ESRRB | 0.007043 | 0.992957 |
| ENST00000428335 | GSG1L | 0.004299 | 0.995701 |
| ENST00000428335 | SNRPD2 | 0.007422 | 0.992578 |
| ENST00000428335 | SIGLEC7 | 0.00632 | 0.99368 |
| ENST00000428335 | SPATA31D1 | 0.005101 | 0.994899 |
| ENST00000428335 | CATG00000038058.1 | 0.007002 | 0.992998 |
| ENST00000428335 | CATG00000034210.1 | 3.92E-04 | 0.999608 |
| ENST00000428335 | RRBP1 | 0.003625 | -0.99638 |
| ENST00000428335 | ZC3H12D | 0.005778 | -0.99422 |
| ENST00000428335 | MORN3 | 0.003878 | -0.99612 |
| ENST00000428335 | FGF9 | 0.003604 | -0.9964 |
| ENST00000428335 | CAPN15 | 0.001268 | 0.998732 |
| ENST00000428335 | CCDC174 | 0.009716 | -0.99028 |
| ENST00000428335 | FAM151A | 0.007237 | 0.992763 |
| ENST00000428335 | SIRPG | 0.006678 | 0.993322 |
| ENST00000428335 | GSTA4 | 0.009209 | 0.990791 |
| ENST00000428335 | TBX3 | 0.002834 | 0.997166 |
| ENST00000428335 | AHSP | 0.001623 | 0.998377 |
| ENST00000428335 | KRT83 | 0.005081 | 0.994919 |
| ENST00000428335 | AC092073.1 | 0.003864 | 0.996136 |
| ENST00000428335 | CATG00000022188.1 | 0.009404 | 0.990596 |
| ENST00000428335 | CATG00000012021.1 | 0.003927 | -0.99607 |
| ENST00000428335 | TGFBR3 | 0.005973 | 0.994027 |
| ENST00000428335 | ESRRB | 0.007043 | 0.992957 |
| ENST00000428335 | GSG1L | 0.004299 | 0.995701 |
| ENST00000428335 | SNRPD2 | 0.007422 | 0.992578 |
| ENST00000428335 | SIGLEC7 | 0.00632 | 0.99368 |
| ENST00000428335 | SPATA31D1 | 0.005101 | 0.994899 |
| ENST00000428335 | CATG00000038058.1 | 0.007002 | 0.992998 |
| ENST00000428335 | CATG00000034210.1 | 3.92E-04 | 0.999608 |
| ENST00000428335 | RRBP1 | 0.003625 | -0.99638 |
| ENST00000428335 | ZC3H12D | 0.005778 | -0.99422 |
| ENST00000428335 | MORN3 | 0.003878 | -0.99612 |
| ENST00000428335 | FGF9 | 0.003604 | -0.9964 |
| ENST00000428335 | CAPN15 | 0.001268 | 0.998732 |
| ENST00000428335 | CCDC174 | 0.009716 | -0.99028 |
| ENST00000428335 | FAM151A | 0.007237 | 0.992763 |
| ENST00000487624 | TAS2R42 | 0.002606 | -0.99739 |
| ENST00000487624 | GRINA | 0.009308 | -0.99069 |
| ENST00000487624 | PPP3CC | 0.004734 | 0.995266 |
| ENST00000487624 | PSRC1 | 0.002631 | 0.997369 |
| ENST00000487624 | ABCB8 | 0.00555 | -0.99445 |
| ENST00000487624 | LILRB1 | 0.008005 | 0.991995 |
| ENST00000487624 | RASL10A | 0.005922 | 0.994078 |
| ENST00000487624 | LTA4H | 4.14E-05 | 0.999959 |
| ENST00000487624 | TUT4 | 0.001858 | 0.998142 |
| ENST00000487624 | WDR33 | 0.003389 | -0.99661 |
| ENST00000487624 | CATG00000053512.1 | 0.006114 | 0.993886 |
| ENST00000487624 | HLF | 0.00879 | -0.99121 |
| ENST00000487624 | NFX1 | 0.005876 | 0.994124 |
| ENST00000487624 | CATG00000063823.1 | 0.008679 | -0.99132 |
| ENST00000487624 | PEA15 | 0.006179 | -0.99382 |
| ENST00000487624 | PSMD4 | 0.007678 | 0.992322 |
| ENST00000487624 | SLC12A3 | 0.004924 | 0.995076 |
| ENST00000487624 | WNT8B | 0.006296 | -0.9937 |
| ENST00000487624 | OR6F1 | 0.009311 | -0.99069 |
| ENST00000487624 | ADAM28 | 0.007668 | 0.992332 |
| ENST00000487624 | UNC5C | 0.005447 | 0.994553 |
| ENST00000487624 | TRIM47 | 0.007475 | -0.99253 |
| ENST00000487624 | CATG00000087047.1 | 3.72E-04 | -0.99963 |
| ENST00000487624 | AC109583.1 | 0.007631 | -0.99237 |
| ENST00000487624 | CFAP410 | 0.006755 | -0.99324 |
| ENST00000487624 | HPS1 | 0.008297 | -0.9917 |
| ENST00000487624 | RAD51AP2 | 8.53E-04 | 0.999147 |
| ENST00000470156 | NUP54 | 0.006327 | -0.99367 |
| ENST00000470156 | TDRD12 | 0.008266 | -0.99173 |
| ENST00000470156 | FTSJ1 | 0.003514 | -0.99649 |
| ENST00000470156 | ANXA3 | 1.23E-04 | -0.99988 |
| ENST00000470156 | MAP3K12 | 0.006581 | -0.99342 |
| ENST00000470156 | CLCN5 | 0.008399 | -0.9916 |
| ENST00000470156 | CHIC2 | 0.006012 | 0.993988 |
| ENST00000470156 | RNF149 | 0.007184 | 0.992816 |
| ENST00000470156 | NUP54 | 0.006327 | -0.99367 |
| ENST00000470156 | TDRD12 | 0.008266 | -0.99173 |
| ENST00000470156 | FTSJ1 | 0.003514 | -0.99649 |
| ENST00000470156 | ANXA3 | 1.23E-04 | -0.99988 |
| ENST00000470156 | MAP3K12 | 0.006581 | -0.99342 |
| ENST00000470156 | CLCN5 | 0.008399 | -0.9916 |
| ENST00000470156 | CHIC2 | 0.006012 | 0.993988 |
| ENST00000470156 | RNF149 | 0.007184 | 0.992816 |
| ENST00000470156 | NUP54 | 0.006327 | -0.99367 |
| ENST00000470156 | TDRD12 | 0.008266 | -0.99173 |
| ENST00000470156 | FTSJ1 | 0.003514 | -0.99649 |
| ENST00000470156 | ANXA3 | 1.23E-04 | -0.99988 |
| ENST00000470156 | MAP3K12 | 0.006581 | -0.99342 |
| ENST00000470156 | CLCN5 | 0.008399 | -0.9916 |
| ENST00000470156 | CHIC2 | 0.006012 | 0.993988 |
| ENST00000470156 | RNF149 | 0.007184 | 0.992816 |
| ENST00000470156 | NUP54 | 0.006327 | -0.99367 |
| ENST00000470156 | TDRD12 | 0.008266 | -0.99173 |
| ENST00000470156 | FTSJ1 | 0.003514 | -0.99649 |
| ENST00000470156 | ANXA3 | 1.23E-04 | -0.99988 |
| ENST00000470156 | MAP3K12 | 0.006581 | -0.99342 |
| ENST00000470156 | CLCN5 | 0.008399 | -0.9916 |
| ENST00000470156 | CHIC2 | 0.006012 | 0.993988 |
| ENST00000470156 | RNF149 | 0.007184 | 0.992816 |
| ENST00000470156 | NUP54 | 0.006327 | -0.99367 |
| ENST00000470156 | TDRD12 | 0.008266 | -0.99173 |
| ENST00000470156 | FTSJ1 | 0.003514 | -0.99649 |
| ENST00000470156 | ANXA3 | 1.23E-04 | -0.99988 |
| ENST00000470156 | MAP3K12 | 0.006581 | -0.99342 |
| ENST00000470156 | CLCN5 | 0.008399 | -0.9916 |
| ENST00000470156 | CHIC2 | 0.006012 | 0.993988 |
| ENST00000470156 | RNF149 | 0.007184 | 0.992816 |
| ENST00000470156 | NUP54 | 0.006327 | -0.99367 |
| ENST00000470156 | TDRD12 | 0.008266 | -0.99173 |
| ENST00000470156 | FTSJ1 | 0.003514 | -0.99649 |
| ENST00000470156 | ANXA3 | 1.23E-04 | -0.99988 |
| ENST00000470156 | MAP3K12 | 0.006581 | -0.99342 |
| ENST00000470156 | CLCN5 | 0.008399 | -0.9916 |
| ENST00000470156 | CHIC2 | 0.006012 | 0.993988 |
| ENST00000470156 | RNF149 | 0.007184 | 0.992816 |
| ENST00000470156 | NUP54 | 0.006327 | -0.99367 |
| ENST00000470156 | TDRD12 | 0.008266 | -0.99173 |
| ENST00000470156 | FTSJ1 | 0.003514 | -0.99649 |
| ENST00000470156 | ANXA3 | 1.23E-04 | -0.99988 |
| ENST00000470156 | MAP3K12 | 0.006581 | -0.99342 |
| ENST00000470156 | CLCN5 | 0.008399 | -0.9916 |
| ENST00000470156 | CHIC2 | 0.006012 | 0.993988 |
| ENST00000470156 | RNF149 | 0.007184 | 0.992816 |
| ENST00000470156 | NUP54 | 0.006327 | -0.99367 |
| ENST00000470156 | TDRD12 | 0.008266 | -0.99173 |
| ENST00000470156 | FTSJ1 | 0.003514 | -0.99649 |
| ENST00000470156 | ANXA3 | 1.23E-04 | -0.99988 |
| ENST00000470156 | MAP3K12 | 0.006581 | -0.99342 |
| ENST00000470156 | CLCN5 | 0.008399 | -0.9916 |
| ENST00000470156 | CHIC2 | 0.006012 | 0.993988 |
| ENST00000470156 | RNF149 | 0.007184 | 0.992816 |
| ENST00000470156 | NUP54 | 0.006327 | -0.99367 |
| ENST00000470156 | TDRD12 | 0.008266 | -0.99173 |
| ENST00000470156 | FTSJ1 | 0.003514 | -0.99649 |
| ENST00000470156 | ANXA3 | 1.23E-04 | -0.99988 |
| ENST00000470156 | MAP3K12 | 0.006581 | -0.99342 |
| ENST00000470156 | CLCN5 | 0.008399 | -0.9916 |
| ENST00000470156 | CHIC2 | 0.006012 | 0.993988 |
| ENST00000470156 | RNF149 | 0.007184 | 0.992816 |
| ENST00000470156 | NUP54 | 0.006327 | -0.99367 |
| ENST00000470156 | TDRD12 | 0.008266 | -0.99173 |
| ENST00000470156 | FTSJ1 | 0.003514 | -0.99649 |
| ENST00000470156 | ANXA3 | 1.23E-04 | -0.99988 |
| ENST00000470156 | MAP3K12 | 0.006581 | -0.99342 |
| ENST00000470156 | CLCN5 | 0.008399 | -0.9916 |
| ENST00000470156 | CHIC2 | 0.006012 | 0.993988 |
| ENST00000470156 | RNF149 | 0.007184 | 0.992816 |
| ENST00000470156 | NUP54 | 0.006327 | -0.99367 |
| ENST00000470156 | TDRD12 | 0.008266 | -0.99173 |
| ENST00000470156 | FTSJ1 | 0.003514 | -0.99649 |
| ENST00000470156 | ANXA3 | 1.23E-04 | -0.99988 |
| ENST00000470156 | MAP3K12 | 0.006581 | -0.99342 |
| ENST00000470156 | CLCN5 | 0.008399 | -0.9916 |
| ENST00000470156 | CHIC2 | 0.006012 | 0.993988 |
| ENST00000470156 | RNF149 | 0.007184 | 0.992816 |
| ENST00000470156 | NUP54 | 0.006327 | -0.99367 |
| ENST00000470156 | TDRD12 | 0.008266 | -0.99173 |
| ENST00000470156 | FTSJ1 | 0.003514 | -0.99649 |
| ENST00000470156 | ANXA3 | 1.23E-04 | -0.99988 |
| ENST00000470156 | MAP3K12 | 0.006581 | -0.99342 |
| ENST00000470156 | CLCN5 | 0.008399 | -0.9916 |
| ENST00000470156 | CHIC2 | 0.006012 | 0.993988 |
| ENST00000470156 | RNF149 | 0.007184 | 0.992816 |
| ENST00000470156 | NUP54 | 0.006327 | -0.99367 |
| ENST00000470156 | TDRD12 | 0.008266 | -0.99173 |
| ENST00000470156 | FTSJ1 | 0.003514 | -0.99649 |
| ENST00000470156 | ANXA3 | 1.23E-04 | -0.99988 |
| ENST00000470156 | MAP3K12 | 0.006581 | -0.99342 |
| ENST00000470156 | CLCN5 | 0.008399 | -0.9916 |
| ENST00000470156 | CHIC2 | 0.006012 | 0.993988 |
| ENST00000470156 | RNF149 | 0.007184 | 0.992816 |
| ENST00000470156 | NUP54 | 0.006327 | -0.99367 |
| ENST00000470156 | TDRD12 | 0.008266 | -0.99173 |
| ENST00000470156 | FTSJ1 | 0.003514 | -0.99649 |
| ENST00000470156 | ANXA3 | 1.23E-04 | -0.99988 |
| ENST00000470156 | MAP3K12 | 0.006581 | -0.99342 |
| ENST00000470156 | CLCN5 | 0.008399 | -0.9916 |
| ENST00000470156 | CHIC2 | 0.006012 | 0.993988 |
| ENST00000470156 | RNF149 | 0.007184 | 0.992816 |
| ENST00000470156 | NUP54 | 0.006327 | -0.99367 |
| ENST00000470156 | TDRD12 | 0.008266 | -0.99173 |
| ENST00000470156 | FTSJ1 | 0.003514 | -0.99649 |
| ENST00000470156 | ANXA3 | 1.23E-04 | -0.99988 |
| ENST00000470156 | MAP3K12 | 0.006581 | -0.99342 |
| ENST00000470156 | CLCN5 | 0.008399 | -0.9916 |
| ENST00000470156 | CHIC2 | 0.006012 | 0.993988 |
| ENST00000470156 | RNF149 | 0.007184 | 0.992816 |
| ENST00000470156 | NUP54 | 0.006327 | -0.99367 |
| ENST00000470156 | TDRD12 | 0.008266 | -0.99173 |
| ENST00000470156 | FTSJ1 | 0.003514 | -0.99649 |
| ENST00000470156 | ANXA3 | 1.23E-04 | -0.99988 |
| ENST00000470156 | MAP3K12 | 0.006581 | -0.99342 |
| ENST00000470156 | CLCN5 | 0.008399 | -0.9916 |
| ENST00000470156 | CHIC2 | 0.006012 | 0.993988 |
| ENST00000470156 | RNF149 | 0.007184 | 0.992816 |
| ENST00000470156 | NUP54 | 0.006327 | -0.99367 |
| ENST00000470156 | TDRD12 | 0.008266 | -0.99173 |
| ENST00000470156 | FTSJ1 | 0.003514 | -0.99649 |
| ENST00000470156 | ANXA3 | 1.23E-04 | -0.99988 |
| ENST00000470156 | MAP3K12 | 0.006581 | -0.99342 |
| ENST00000470156 | CLCN5 | 0.008399 | -0.9916 |
| ENST00000470156 | CHIC2 | 0.006012 | 0.993988 |
| ENST00000470156 | RNF149 | 0.007184 | 0.992816 |
| compmerge.4887.pooled.chr3 | ACOT12 | 0.008625 | -0.99138 |
| compmerge.4887.pooled.chr3 | VRK3 | 0.009451 | -0.99055 |
| compmerge.4887.pooled.chr3 | CLEC17A | 0.00842 | -0.99158 |
| compmerge.4887.pooled.chr3 | BLCAP | 0.008239 | -0.99176 |
| compmerge.4887.pooled.chr3 | SELENOP | 0.007611 | 0.992389 |
| compmerge.4887.pooled.chr3 | BCAS4 | 0.008177 | -0.99182 |
| compmerge.4887.pooled.chr3 | PROKR1 | 0.007814 | 0.992186 |
| compmerge.4887.pooled.chr3 | CATG00000023328.1 | 0.005672 | 0.994328 |
| compmerge.4887.pooled.chr3 | OR8B4 | 0.003882 | 0.996118 |
| compmerge.4887.pooled.chr3 | ACSL6 | 0.009438 | -0.99056 |
| compmerge.4887.pooled.chr3 | MAP3K5 | 0.009345 | -0.99065 |
| compmerge.4887.pooled.chr3 | FANCL | 0.003278 | -0.99672 |
| compmerge.4887.pooled.chr3 | CATG00000024701.1 | 0.009452 | -0.99055 |
| ENST00000569407 | DMAC2 | 0.006303 | 0.993697 |
| ENST00000569407 | RSL1D1 | 0.004342 | -0.99566 |
| ENST00000569407 | GORASP1 | 3.40E-04 | -0.99966 |
| ENST00000569407 | SLC2A4 | 0.006001 | 0.993999 |
| ENST00000569407 | HIRA | 0.005559 | -0.99444 |
| ENST00000569407 | TMEM155 | 0.006743 | 0.993257 |
| ENST00000569407 | ABCB8 | 0.008326 | 0.991674 |
| ENST00000569407 | HERPUD2 | 0.002324 | -0.99768 |
| ENST00000569407 | SLC22A12 | 0.002637 | 0.997363 |
| ENST00000569407 | PLPPR4 | 0.005723 | -0.99428 |
| ENST00000569407 | FCHSD2 | 4.45E-04 | -0.99955 |
| ENST00000569407 | HLA-A | 0.009852 | -0.99015 |
| ENST00000569407 | ZNF579 | 0.005158 | 0.994842 |
| ENST00000569407 | KCNK10 | 0.00655 | 0.99345 |
| ENST00000569407 | PTPN23 | 0.003464 | 0.996536 |
| ENST00000569407 | AGO2 | 0.009077 | 0.990923 |
| ENST00000569407 | TRIM47 | 0.00678 | 0.99322 |
| ENST00000569407 | DHDH | 9.85E-04 | 0.999015 |
| ENST00000569407 | DCUN1D1 | 0.009576 | -0.99042 |
| ENST00000569407 | ATG4C | 0.007951 | 0.992049 |
| ENST00000569407 | DMAC2 | 0.006303 | 0.993697 |
| ENST00000569407 | RSL1D1 | 0.004342 | -0.99566 |
| ENST00000569407 | GORASP1 | 3.40E-04 | -0.99966 |
| ENST00000569407 | SLC2A4 | 0.006001 | 0.993999 |
| ENST00000569407 | HIRA | 0.005559 | -0.99444 |
| ENST00000569407 | TMEM155 | 0.006743 | 0.993257 |
| ENST00000569407 | ABCB8 | 0.008326 | 0.991674 |
| ENST00000569407 | HERPUD2 | 0.002324 | -0.99768 |
| ENST00000569407 | SLC22A12 | 0.002637 | 0.997363 |
| ENST00000569407 | PLPPR4 | 0.005723 | -0.99428 |
| ENST00000569407 | FCHSD2 | 4.45E-04 | -0.99955 |
| ENST00000569407 | HLA-A | 0.009852 | -0.99015 |
| ENST00000569407 | ZNF579 | 0.005158 | 0.994842 |
| ENST00000569407 | KCNK10 | 0.00655 | 0.99345 |
| ENST00000569407 | PTPN23 | 0.003464 | 0.996536 |
| ENST00000569407 | AGO2 | 0.009077 | 0.990923 |
| ENST00000569407 | TRIM47 | 0.00678 | 0.99322 |
| ENST00000569407 | DHDH | 9.85E-04 | 0.999015 |
| ENST00000569407 | DCUN1D1 | 0.009576 | -0.99042 |
| ENST00000569407 | ATG4C | 0.007951 | 0.992049 |
| ENST00000569407 | DMAC2 | 0.006303 | 0.993697 |
| ENST00000569407 | RSL1D1 | 0.004342 | -0.99566 |
| ENST00000569407 | GORASP1 | 3.40E-04 | -0.99966 |
| ENST00000569407 | SLC2A4 | 0.006001 | 0.993999 |
| ENST00000569407 | HIRA | 0.005559 | -0.99444 |
| ENST00000569407 | TMEM155 | 0.006743 | 0.993257 |
| ENST00000569407 | ABCB8 | 0.008326 | 0.991674 |
| ENST00000569407 | HERPUD2 | 0.002324 | -0.99768 |
| ENST00000569407 | SLC22A12 | 0.002637 | 0.997363 |
| ENST00000569407 | PLPPR4 | 0.005723 | -0.99428 |
| ENST00000569407 | FCHSD2 | 4.45E-04 | -0.99955 |
| ENST00000569407 | HLA-A | 0.009852 | -0.99015 |
| ENST00000569407 | ZNF579 | 0.005158 | 0.994842 |
| ENST00000569407 | KCNK10 | 0.00655 | 0.99345 |
| ENST00000569407 | PTPN23 | 0.003464 | 0.996536 |
| ENST00000569407 | AGO2 | 0.009077 | 0.990923 |
| ENST00000569407 | TRIM47 | 0.00678 | 0.99322 |
| ENST00000569407 | DHDH | 9.85E-04 | 0.999015 |
| ENST00000569407 | DCUN1D1 | 0.009576 | -0.99042 |
| ENST00000569407 | ATG4C | 0.007951 | 0.992049 |
| ENST00000514802 | FCN2 | 0.006231 | 0.993769 |
| ENST00000514802 | GBA | 0.002924 | 0.997076 |
| ENST00000514802 | RASSF6 | 0.00401 | 0.99599 |
| ENST00000514802 | ZNF713 | 0.009849 | 0.990151 |
| ENST00000514802 | ABR | 0.004476 | 0.995524 |
| ENST00000514802 | SIVA1 | 0.008901 | -0.9911 |
| ENST00000514802 | CATG00000057824.1 | 0.006009 | 0.993991 |
| ENST00000514802 | CDK11B | 0.001785 | -0.99821 |
| ENST00000514802 | TCTEX1D4 | 0.005876 | -0.99412 |
| ENST00000514802 | MAP1LC3A | 0.003969 | 0.996031 |
| ENST00000514802 | NTF3 | 0.002987 | 0.997013 |
| ENST00000514802 | SH3RF3 | 0.001177 | -0.99882 |
| ENST00000514802 | LDHB | 0.0049 | -0.9951 |
| ENST00000514802 | ACMSD | 0.008514 | -0.99149 |
| ENST00000584219 | RNF207 | 7.18E-04 | 0.999282 |
| ENST00000584219 | POLR2F | 0.004247 | -0.99575 |
| ENST00000584219 | PLEKHA2 | 0.003108 | -0.99689 |
| ENST00000584219 | CFAP161 | 0.006268 | -0.99373 |
| ENST00000584219 | DYNC2H1 | 0.002011 | 0.997989 |
| ENST00000584219 | RNF207 | 7.18E-04 | 0.999282 |
| ENST00000584219 | POLR2F | 0.004247 | -0.99575 |
| ENST00000584219 | PLEKHA2 | 0.003108 | -0.99689 |
| ENST00000584219 | CFAP161 | 0.006268 | -0.99373 |
| ENST00000584219 | DYNC2H1 | 0.002011 | 0.997989 |
