# Supplementary material for: M6A Modification and Transcription Analysis of LncRNA in Cerebral Ischemia/Reperfusion Injury
Source: Int J Genomics. 2024 Oct 5;2024:4596974. doi: 10.1155/2024/4596974 (PMC11470819; doi:10.1155/2024/4596974)
Supplement: Supporting Information — Additional supporting information can be found online in the Supporting Information section. Table S1. Pearson correlation analysis between upregulated LncRNA and mRNA. Table S2. Pearson correlation analysis between downregulated LncRNA and mRNA. Table S3. Pearson correlation analysis between hypermethylated LncRNA and mRNA. Table S4. Pearson correlation analysis between hypomethylated LncRNA and mRNA. [file 4596974.f1.zip › Table S3.docx]

**Table S3** **Pearson correlation analysis between hyper-methylated LncRNA and mRNA**

| LncRNA | mRNA | P | R |
| --- | --- | --- | --- |
| ENST00000442166 | SLC38A10 | 0.007823 | -0.99218 |
| ENST00000442166 | PLEKHD1 | 0.001041 | 0.998959 |
| ENST00000442166 | MLLT1 | 0.001131 | -0.99887 |
| ENST00000442166 | STMND1 | 0.006802 | -0.9932 |
| ENST00000442166 | DNAH6 | 0.004211 | -0.99579 |
| ENST00000442166 | LYPD8 | 0.007075 | -0.99292 |
| ENST00000442166 | SLC38A10 | 0.007823 | -0.99218 |
| ENST00000442166 | PLEKHD1 | 0.001041 | 0.998959 |
| ENST00000442166 | MLLT1 | 0.001131 | -0.99887 |
| ENST00000442166 | STMND1 | 0.006802 | -0.9932 |
| ENST00000442166 | DNAH6 | 0.004211 | -0.99579 |
| ENST00000442166 | LYPD8 | 0.007075 | -0.99292 |
| ENST00000530897 | DNAJC6 | 0.001597 | 0.998403 |
| ENST00000530897 | MFAP3L | 0.001004 | 0.998996 |
| ENST00000530897 | CATG00000086946.1 | 0.001692 | 0.998308 |
| ENST00000530897 | SPACA9 | 0.002883 | 0.997117 |
| ENST00000530897 | CMBL | 0.001464 | 0.998536 |
| ENST00000530897 | ORM1 | 0.007079 | 0.992921 |
| ENST00000520025 | BECN1 | 0.008729 | -0.99127 |
| ENST00000520025 | CCDC42 | 0.004269 | -0.99573 |
| ENST00000520025 | MED12L | 0.004147 | 0.995853 |
| ENST00000520025 | CEBPD | 0.008685 | 0.991315 |
| NR_002570 | PGM5 | 0.00718 | 0.99282 |
| NR_002570 | DGAT2 | 0.002414 | 0.997586 |
| NR_002570 | SPRR1A | 0.004198 | -0.9958 |
| NR_002570 | LY6G6C | 0.002961 | 0.997039 |
| NR_002570 | AOAH | 0.00215 | -0.99785 |
| NR_002570 | MOK | 0.009071 | 0.990929 |
| NR_002570 | CD38 | 5.77E-04 | 0.999423 |
| NR_120450 | TOB1 | 0.00478 | -0.99522 |
| NR_120450 | ANKRD13D | 0.004952 | -0.99505 |
| NR_120450 | MED20 | 0.002167 | 0.997833 |
| NR_120450 | CATG00000087963.1 | 0.004766 | 0.995234 |
| NR_120450 | EPHB6 | 0.001874 | 0.998126 |
| NR_120450 | CATG00000021838.1 | 0.003111 | -0.99689 |
| NR_120450 | TSFM | 1.18E-04 | -0.99988 |
| ENST00000447972 | RRAS2 | 0.005481 | 0.994519 |
| ENST00000580278 | SCAMP2 | 0.001561 | 0.998439 |
| ENST00000580278 | TMC5 | 0.008578 | 0.991422 |
| ENST00000580278 | PEX14 | 0.003706 | 0.996294 |
| ENST00000580278 | XYLT2 | 0.002826 | 0.997174 |
| ENST00000580278 | IKZF4 | 4.72E-04 | 0.999528 |
| ENST00000580278 | MYH3 | 0.00248 | -0.99752 |
| ENST00000580278 | KCTD1 | 0.001784 | 0.998216 |
| ENST00000580278 | CATG00000047316.1 | 0.008015 | 0.991985 |
| ENST00000580278 | PURG | 0.006494 | 0.993506 |
| ENST00000580278 | CACNG6 | 0.005355 | 0.994645 |
| ENST00000580278 | PAN3 | 0.005781 | -0.99422 |
| ENST00000580278 | SHROOM3 | 0.007311 | 0.992689 |
| ENST00000580278 | INKA2 | 0.001645 | 0.998355 |
| ENST00000580278 | RAMP1 | 0.0061 | -0.9939 |
| ENST00000580278 | PARVG | 0.004941 | 0.995059 |
| ENST00000580278 | ABCA13 | 0.007326 | 0.992674 |
| ENST00000580278 | ZNF37A | 0.005343 | -0.99466 |
| ENST00000580278 | DICER1 | 0.005918 | -0.99408 |
| ENST00000580278 | YPEL5 | 0.00388 | 0.99612 |
| NR_037177 | TNFRSF18 | 0.005172 | 0.994828 |
| NR_037177 | SPATA33 | 0.004288 | 0.995712 |
| NR_037177 | PLEKHD1 | 0.005617 | 0.994383 |
| NR_037177 | MLLT1 | 0.007285 | -0.99271 |
| NR_037177 | TNXB | 0.00795 | -0.99205 |
| NR_037177 | DNAH6 | 0.006896 | -0.9931 |
| NR_037177 | HEATR9 | 0.002236 | -0.99776 |
| NR_037177 | TNRC6B | 0.005895 | -0.9941 |
| ENST00000507571 | IL17RA | 0.00862 | 0.99138 |
| ENST00000507571 | AHSP | 0.001537 | 0.998463 |
| ENST00000507571 | AC092073.1 | 7.30E-06 | 0.999993 |
| ENST00000507571 | TGFBR3 | 5.60E-04 | 0.99944 |
| ENST00000507571 | SLC22A6 | 0.006671 | -0.99333 |
| ENST00000507571 | CATG00000034210.1 | 0.006523 | 0.993477 |
| ENST00000507571 | COA5 | 0.004611 | -0.99539 |
| ENST00000507571 | SLC6A5 | 0.006257 | 0.993743 |
| ENST00000507571 | RRBP1 | 2.22E-05 | -0.99998 |
| ENST00000507571 | MORN3 | 8.16E-04 | -0.99918 |
| ENST00000507571 | VPS13A | 0.002383 | 0.997617 |
| ENST00000507571 | FGF9 | 0.007048 | -0.99295 |
| ENST00000507571 | CAPN15 | 0.002559 | 0.997441 |
| ENST00000468219 | TBC1D29 | 0.009406 | 0.990594 |
| ENST00000468219 | TCF23 | 0.003576 | -0.99642 |
| ENST00000468219 | HTRA4 | 0.009306 | -0.99069 |
| NR_104428 | LRMDA | 0.001839 | -0.99816 |
| NR_104428 | PHF13 | 0.007324 | 0.992676 |
| NR_104428 | YES1 | 0.00527 | 0.99473 |
| NR_104428 | CTSL | 4.75E-04 | -0.99953 |
| NR_104428 | TNFSF13B | 0.004162 | -0.99584 |
| NR_104428 | AGAP1 | 0.009252 | 0.990748 |
| NR_104428 | CATG00000051841.1 | 0.001431 | 0.998569 |
| NR_104428 | GDF2 | 0.008579 | 0.991421 |
| ENST00000304233 | FCN2 | 0.005648 | 0.994352 |
| ENST00000304233 | GBA | 0.004007 | 0.995993 |
| ENST00000304233 | RASSF6 | 0.0033 | 0.9967 |
| ENST00000304233 | PCBP4 | 0.008436 | -0.99156 |
| ENST00000304233 | ZNF713 | 0.009083 | 0.990917 |
| ENST00000304233 | ABR | 0.003488 | 0.996512 |
| ENST00000304233 | SIVA1 | 0.008463 | -0.99154 |
| ENST00000304233 | CATG00000057824.1 | 0.007301 | 0.992699 |
| ENST00000304233 | CDK11B | 0.002578 | -0.99742 |
| ENST00000304233 | TCTEX1D4 | 0.006771 | -0.99323 |
| ENST00000304233 | MAP1LC3A | 0.005218 | 0.994782 |
| ENST00000304233 | NTF3 | 0.002274 | 0.997726 |
| ENST00000304233 | SH3RF3 | 0.001456 | -0.99854 |
| ENST00000304233 | LDHB | 0.005788 | -0.99421 |
| ENST00000304233 | ACMSD | 0.009646 | -0.99035 |
| ENST00000437416 | GBA | 0.005808 | 0.994192 |
| ENST00000437416 | SLC7A4 | 7.78E-04 | 0.999222 |
| ENST00000437416 | EIF1AD | 0.009088 | 0.990912 |
| ENST00000437416 | SMPD1 | 0.007476 | 0.992524 |
| ENST00000437416 | SAMD1 | 0.001722 | -0.99828 |
| ENST00000437416 | ZMYND15 | 0.006333 | 0.993667 |
| ENST00000437416 | CPOX | 0.006588 | 0.993412 |
| ENST00000437416 | CATG00000057824.1 | 9.58E-04 | 0.999042 |
| ENST00000437416 | CDK11B | 0.009295 | -0.9907 |
| ENST00000437416 | IQCF5 | 0.003531 | 0.996469 |
| ENST00000437416 | KLF6 | 0.005128 | -0.99487 |
| ENST00000437416 | MAP1LC3A | 0.005418 | 0.994582 |
| ENST00000437416 | LBP | 0.006415 | 0.993585 |
| ENST00000437416 | DNAL4 | 0.008304 | -0.9917 |
| ENST00000437416 | FAM53B | 8.97E-04 | -0.9991 |
| ENST00000437416 | LDHB | 0.001403 | -0.9986 |
| ENST00000437416 | AL627171.2 | 0.00773 | -0.99227 |
| NR_073490 | CATG00000092654.1 | 4.68E-04 | 0.999532 |
| NR_073490 | APBB3 | 7.21E-04 | -0.99928 |
| NR_073490 | EXOC2 | 0.0095 | 0.9905 |
| NR_073490 | CHSY3 | 0.007753 | 0.992247 |
| NR_073490 | GLIPR1L2 | 0.003366 | 0.996634 |
| NR_073490 | IKBKE | 0.005557 | 0.994443 |
| NR_073490 | CATG00000113928.1 | 0.008749 | -0.99125 |
| NR_073490 | RTN4 | 0.006604 | -0.9934 |
| NR_073490 | KCNK16 | 0.004601 | -0.9954 |
| NR_073490 | MMP24 | 0.009751 | -0.99025 |
| NR_073490 | FANCE | 0.004913 | -0.99509 |
| ENST00000423187 | INPP5J | 0.006384 | 0.993616 |
| ENST00000423187 | RFC2 | 0.007547 | -0.99245 |
| ENST00000423187 | RTN1 | 0.007455 | -0.99255 |
| ENST00000423187 | EXOC4 | 0.003694 | 0.996306 |
| ENST00000423187 | CNEP1R1 | 0.009268 | -0.99073 |
| ENST00000423187 | DBI | 0.002273 | -0.99773 |
| ENST00000423187 | TP73 | 0.003662 | 0.996338 |
| ENST00000423187 | SEC14L6 | 0.008941 | 0.991059 |
| ENST00000423187 | NEMP2 | 0.006327 | 0.993673 |
| ENST00000423187 | NEFM | 0.007988 | -0.99201 |
| ENST00000423187 | BMPER | 0.006881 | 0.993119 |
| ENST00000423187 | DEFA6 | 9.52E-04 | 0.999048 |
| ENST00000423187 | CATG00000027020.1 | 0.005719 | 0.994281 |
| ENST00000457816 | WNT16 | 0.005598 | 0.994402 |
| ENST00000457816 | AGPS | 0.006533 | -0.99347 |
| ENST00000457816 | TEX13D | 0.003008 | 0.996992 |
| ENST00000457816 | NOL9 | 0.005387 | -0.99461 |
| ENST00000457816 | NLRP9 | 0.00885 | 0.99115 |
| ENST00000457816 | IRX3 | 4.47E-04 | 0.999553 |
| ENST00000457816 | SNX16 | 0.004901 | -0.9951 |
| ENST00000457816 | CD180 | 0.00363 | -0.99637 |
| ENST00000457816 | SPATA13 | 0.002123 | 0.997877 |
| ENST00000457816 | PLTP | 0.009954 | 0.990046 |
| ENST00000457816 | PLCH2 | 0.008257 | 0.991743 |
| ENST00000457816 | COBL | 9.47E-05 | 0.999905 |
| ENST00000457816 | SIPA1L2 | 0.002968 | 0.997032 |
| ENST00000457816 | PIPOX | 0.001026 | -0.99897 |
