# Supplementary material for: M6A Modification and Transcription Analysis of LncRNA in Cerebral Ischemia/Reperfusion Injury
Source: Int J Genomics. 2024 Oct 5;2024:4596974. doi: 10.1155/2024/4596974 (PMC11470819; doi:10.1155/2024/4596974)
Supplement: Supporting Information — Additional supporting information can be found online in the Supporting Information section. Table S1. Pearson correlation analysis between upregulated LncRNA and mRNA. Table S2. Pearson correlation analysis between downregulated LncRNA and mRNA. Table S3. Pearson correlation analysis between hypermethylated LncRNA and mRNA. Table S4. Pearson correlation analysis between hypomethylated LncRNA and mRNA. [file 4596974.f1.zip › Table S4.docx]

**Table S4** **Pearson correlation analysis between hypo-methylated LncRNA and mRNA**

| LncRNA | mRNA | P | R |
| --- | --- | --- | --- |
| ENST00000575542 | AX748369 | 0.003441 | -0.99656 |
| ENST00000575542 | GMPPA | 0.008387 | 0.991613 |
| ENST00000575542 | PNPLA2 | 0.001057 | -0.99894 |
| ENST00000575542 | PDCD2L | 0.003602 | 0.996398 |
| ENST00000575542 | DRC1 | 0.006064 | -0.99394 |
| ENST00000575542 | CATG00000108269.1 | 0.005822 | -0.99418 |
| ENST00000575542 | EFCAB8 | 0.008128 | -0.99187 |
| ENST00000575542 | FOXJ3 | 0.008924 | -0.99108 |
| ENST00000575542 | CYP27C1 | 0.001151 | -0.99885 |
| ENST00000575542 | MSI1 | 6.63E-04 | -0.99934 |
| ENST00000535127 | B3GNT3 | 0.00359 | -0.99641 |
| ENST00000535127 | HMGB2 | 0.005154 | -0.99485 |
| ENST00000535127 | PLIN4 | 0.008719 | 0.991281 |
| ENST00000535127 | FOXO6 | 5.16E-04 | 0.999484 |
| ENST00000535127 | CXXC1 | 0.009267 | -0.99073 |
| ENST00000535127 | DGAT2L6 | 0.005706 | 0.994294 |
| ENST00000535127 | CATG00000053512.1 | 0.009431 | -0.99057 |
| ENST00000535127 | HLF | 0.005073 | 0.994927 |
| ENST00000535127 | SRRD | 7.56E-04 | 0.999244 |
| ENST00000535127 | TPTE | 0.002828 | 0.997172 |
| ENST00000535127 | CSTL1 | 0.002263 | 0.997737 |
| ENST00000535127 | FYB2 | 0.001127 | 0.998873 |
| ENST00000535127 | SLC12A3 | 0.008388 | -0.99161 |
| ENST00000535127 | WNT2 | 0.007896 | 0.992104 |
| ENST00000535127 | PAK3 | 0.005365 | 0.994635 |
| ENST00000535127 | PPP5D1 | 0.008878 | 0.991122 |
| ENST00000535127 | OXT | 2.04E-04 | 0.999796 |
| ENST00000535127 | FAM174A | 0.001547 | -0.99845 |
| ENST00000535127 | REG4 | 0.009496 | -0.9905 |
| ENST00000535127 | CFAP410 | 0.006315 | 0.993685 |
| ENST00000586544 | INPP5J | 0.007937 | 0.992063 |
| ENST00000586544 | RFC2 | 0.005246 | -0.99475 |
| ENST00000586544 | CATG00000101330.1 | 0.004874 | -0.99513 |
| ENST00000586544 | CNEP1R1 | 2.95E-04 | -0.9997 |
| ENST00000586544 | FRG1 | 0.009429 | -0.99057 |
| ENST00000586544 | SH3D21 | 0.009683 | 0.990317 |
| ENST00000586544 | TP73 | 0.00629 | 0.99371 |
| ENST00000586544 | ARHGEF26 | 0.004375 | 0.995625 |
| ENST00000586544 | PHKA1 | 0.001199 | 0.998801 |
| ENST00000586544 | CATG00000026557.1 | 0.002943 | 0.997057 |
| ENST00000586544 | COA1 | 0.004229 | -0.99577 |
| ENST00000586544 | PSME1 | 0.00777 | -0.99223 |
| ENST00000586544 | DOCK1 | 0.00237 | 0.99763 |
| ENST00000586544 | BMPER | 0.006454 | 0.993546 |
| NR_027889 | HEXIM2 | 0.002317 | -0.99768 |
| NR_027889 | OCLN | 9.67E-04 | -0.99903 |
| NR_027889 | FAR1 | 0.001455 | -0.99855 |
| NR_027889 | TRIM73 | 0.004604 | -0.9954 |
| NR_027889 | LTF | 0.004088 | 0.995912 |
| NR_027889 | KIF27 | 0.001022 | -0.99898 |
| ENST00000537710 | ACSBG1 | 0.002101 | 0.997899 |
| ENST00000537710 | USP28 | 0.006132 | 0.993868 |
| ENST00000537710 | PDCD2L | 0.009294 | 0.990706 |
| ENST00000537710 | PRR15L | 0.001317 | -0.99868 |
| ENST00000537710 | FOXJ3 | 0.009635 | -0.99036 |
| ENST00000380466 | SYNDIG1L | 0.008281 | -0.99172 |
| ENST00000380466 | IL17RA | 0.003961 | 0.996039 |
| ENST00000380466 | AHSP | 0.001768 | 0.998232 |
| ENST00000380466 | AC092073.1 | 9.59E-04 | 0.999041 |
| ENST00000380466 | TGFBR3 | 0.002516 | 0.997484 |
| ENST00000380466 | LYPD1 | 0.006376 | 0.993624 |
| ENST00000380466 | CATG00000034210.1 | 0.009751 | 0.990249 |
| ENST00000380466 | COA5 | 0.009134 | -0.99087 |
| ENST00000380466 | SLC6A5 | 0.003486 | 0.996514 |
| ENST00000380466 | RRBP1 | 0.001065 | -0.99894 |
| ENST00000380466 | MORN3 | 0.003442 | -0.99656 |
| ENST00000380466 | VPS13A | 0.002792 | 0.997208 |
| ENST00000380466 | CAPN15 | 0.005876 | 0.994124 |
| NR_104596 | RNF207 | 0.00517 | 0.99483 |
| NR_104596 | PLEKHA2 | 8.75E-04 | -0.99912 |
| NR_104596 | DYNC2H1 | 0.001644 | 0.998356 |
| ENST00000467155 | GNL1 | 0.004923 | -0.99508 |
| ENST00000467155 | DEAF1 | 0.005318 | 0.994682 |
| ENST00000467155 | VRK3 | 0.002576 | 0.997424 |
| ENST00000467155 | BLCAP | 0.002513 | 0.997487 |
| ENST00000467155 | FANCD2OS | 0.002629 | 0.997371 |
| ENST00000467155 | CATG00000023328.1 | 5.43E-04 | -0.99946 |
| ENST00000467155 | EFEMP2 | 0.006874 | -0.99313 |
| ENST00000467155 | ACSL6 | 0.00369 | 0.99631 |
| ENST00000467155 | OR4X1 | 0.001976 | 0.998024 |
| ENST00000467155 | MAP3K5 | 0.003762 | 0.996238 |
| ENST00000467155 | HLA-F | 0.005682 | 0.994318 |
| ENST00000467155 | CATG00000024701.1 | 0.001208 | 0.998792 |
| ENST00000586544 | INPP5J | 0.007937 | 0.992063 |
| ENST00000586544 | RFC2 | 0.005246 | -0.99475 |
| ENST00000586544 | CATG00000101330.1 | 0.004874 | -0.99513 |
| ENST00000586544 | CNEP1R1 | 2.95E-04 | -0.9997 |
| ENST00000586544 | FRG1 | 0.009429 | -0.99057 |
| ENST00000586544 | SH3D21 | 0.009683 | 0.990317 |
| ENST00000586544 | TP73 | 0.00629 | 0.99371 |
| ENST00000586544 | ARHGEF26 | 0.004375 | 0.995625 |
| ENST00000586544 | PHKA1 | 0.001199 | 0.998801 |
| ENST00000586544 | CATG00000026557.1 | 0.002943 | 0.997057 |
| ENST00000586544 | COA1 | 0.004229 | -0.99577 |
| ENST00000586544 | PSME1 | 0.00777 | -0.99223 |
| ENST00000586544 | DOCK1 | 0.00237 | 0.99763 |
| ENST00000586544 | BMPER | 0.006454 | 0.993546 |
| ENST00000586544 | INPP5J | 0.007937 | 0.992063 |
| ENST00000586544 | RFC2 | 0.005246 | -0.99475 |
| ENST00000586544 | CATG00000101330.1 | 0.004874 | -0.99513 |
| ENST00000586544 | CNEP1R1 | 2.95E-04 | -0.9997 |
| ENST00000586544 | FRG1 | 0.009429 | -0.99057 |
| ENST00000586544 | SH3D21 | 0.009683 | 0.990317 |
| ENST00000586544 | TP73 | 0.00629 | 0.99371 |
| ENST00000586544 | ARHGEF26 | 0.004375 | 0.995625 |
| ENST00000586544 | PHKA1 | 0.001199 | 0.998801 |
| ENST00000586544 | CATG00000026557.1 | 0.002943 | 0.997057 |
| ENST00000586544 | COA1 | 0.004229 | -0.99577 |
| ENST00000586544 | PSME1 | 0.00777 | -0.99223 |
| ENST00000586544 | DOCK1 | 0.00237 | 0.99763 |
| ENST00000586544 | BMPER | 0.006454 | 0.993546 |
| ENST00000584911 | MFAP3L | 0.008468 | 0.991532 |
| ENST00000584911 | CATG00000086946.1 | 0.009597 | 0.990403 |
| ENST00000584911 | CATG00000039284.1 | 0.007754 | -0.99225 |
| ENST00000584911 | ORM1 | 0.001751 | 0.998249 |
| ENST00000584911 | MFAP3L | 0.008468 | 0.991532 |
| ENST00000584911 | CATG00000086946.1 | 0.009597 | 0.990403 |
| ENST00000584911 | CATG00000039284.1 | 0.007754 | -0.99225 |
| ENST00000584911 | ORM1 | 0.001751 | 0.998249 |
| ENST00000326677 | ZNF490 | 0.001892 | 0.998108 |
| ENST00000326677 | HEYL | 0.003354 | -0.99665 |
| ENST00000326677 | TMEM236 | 0.007759 | 0.992241 |
| ENST00000326677 | SCYGR6 | 0.001906 | 0.998094 |
| ENST00000326677 | BRSK2 | 1.96E-04 | 0.999804 |
| ENST00000326677 | GPCPD1 | 0.009027 | 0.990973 |
| ENST00000326677 | LCE4A | 0.002411 | -0.99759 |
| ENST00000326677 | LYPD8 | 0.007023 | -0.99298 |
| ENST00000326677 | SCRG1 | 7.52E-05 | -0.99992 |
| ENST00000326677 | ZFYVE26 | 0.003954 | -0.99605 |
| NR_145459 | CATG00000080699.1 | 8.43E-04 | 0.999157 |
| NR_145459 | TCOF1 | 0.007129 | 0.992871 |
| NR_145459 | SLIRP | 0.009794 | 0.990206 |
| NR_145459 | MYD88 | 0.008484 | -0.99152 |
| NR_145459 | SAMD13 | 0.006112 | 0.993888 |
| NR_145459 | PPP5C | 0.008134 | 0.991866 |
| NR_145459 | ZDHHC16 | 0.00625 | -0.99375 |
| NR_145459 | MACROD2 | 0.00728 | 0.99272 |
| NR_145459 | CENPO | 0.007331 | 0.992669 |
| NR_145459 | CHST12 | 0.004011 | 0.995989 |
| NR_145459 | HIPK2 | 0.005244 | -0.99476 |
| NR_145459 | PCDHB8 | 0.004011 | 0.995989 |
| NR_145459 | CATG00000080699.1 | 8.43E-04 | 0.999157 |
| NR_145459 | TCOF1 | 0.007129 | 0.992871 |
| NR_145459 | SLIRP | 0.009794 | 0.990206 |
| NR_145459 | MYD88 | 0.008484 | -0.99152 |
| NR_145459 | SAMD13 | 0.006112 | 0.993888 |
| NR_145459 | PPP5C | 0.008134 | 0.991866 |
| NR_145459 | ZDHHC16 | 0.00625 | -0.99375 |
| NR_145459 | MACROD2 | 0.00728 | 0.99272 |
| NR_145459 | CENPO | 0.007331 | 0.992669 |
| NR_145459 | CHST12 | 0.004011 | 0.995989 |
| NR_145459 | HIPK2 | 0.005244 | -0.99476 |
| NR_145459 | PCDHB8 | 0.004011 | 0.995989 |
| NR_145459 | CATG00000080699.1 | 8.43E-04 | 0.999157 |
| NR_145459 | TCOF1 | 0.007129 | 0.992871 |
| NR_145459 | SLIRP | 0.009794 | 0.990206 |
| NR_145459 | MYD88 | 0.008484 | -0.99152 |
| NR_145459 | SAMD13 | 0.006112 | 0.993888 |
| NR_145459 | PPP5C | 0.008134 | 0.991866 |
| NR_145459 | ZDHHC16 | 0.00625 | -0.99375 |
| NR_145459 | MACROD2 | 0.00728 | 0.99272 |
| NR_145459 | CENPO | 0.007331 | 0.992669 |
| NR_145459 | CHST12 | 0.004011 | 0.995989 |
| NR_145459 | HIPK2 | 0.005244 | -0.99476 |
| NR_145459 | PCDHB8 | 0.004011 | 0.995989 |
| NR_045020 | ZFAND5 | 0.003575 | 0.996425 |
| NR_045020 | RTN1 | 0.006185 | -0.99382 |
| NR_045020 | NAGLU | 0.001738 | 0.998262 |
| NR_045020 | TP53I13 | 0.008995 | 0.991005 |
| NR_045020 | KRBA2 | 0.001471 | 0.998529 |
| NR_045020 | CLK3 | 0.005915 | 0.994085 |
| NR_045020 | AP3M2 | 0.003556 | -0.99644 |
| NR_045020 | CREBRF | 0.008145 | -0.99185 |
| NR_045020 | CATG00000054083.1 | 0.009915 | -0.99009 |
| NR_045020 | LCE2A | 0.009325 | 0.990675 |
| NR_045020 | PPTC7 | 0.008509 | -0.99149 |
| NR_045020 | CIB1 | 0.005906 | -0.99409 |
| ENST00000458653 | AHNAK2 | 0.003238 | 0.996762 |
| ENST00000458653 | CHSY3 | 0.008125 | 0.991875 |
| ENST00000458653 | HOMER3 | 0.003496 | -0.9965 |
| ENST00000458653 | ARPC4-TTLL3 | 0.006902 | -0.9931 |
| ENST00000458653 | CATG00000113928.1 | 0.006062 | -0.99394 |
| ENST00000458653 | MMP24 | 0.005213 | -0.99479 |
| ENST00000458653 | ZNF624 | 0.005037 | 0.994963 |
| ENST00000495673 | CATG00000063531.1 | 6.77E-04 | 0.999323 |
| ENST00000495673 | UBE2V1 | 5.12E-04 | 0.999488 |
| ENST00000495673 | CEP44 | 7.35E-04 | 0.999265 |
| ENST00000495673 | RASSF6 | 0.009977 | 0.990023 |
| ENST00000495673 | PCBP4 | 7.93E-04 | -0.99921 |
| ENST00000495673 | ABR | 0.00642 | 0.99358 |
| ENST00000495673 | OR5M8 | 0.008599 | 0.991401 |
| ENST00000495673 | NTF3 | 0.008714 | 0.991286 |
| ENST00000495673 | RTL8B | 0.00315 | -0.99685 |
| ENST00000495673 | ERVW-1 | 2.84E-04 | -0.99972 |
| ENST00000495673 | CATG00000117842.1 | 0.004411 | 0.995589 |
| ENST00000495673 | C16orf86 | 0.003514 | -0.99649 |
| ENST00000584911 | MFAP3L | 0.008468 | 0.991532 |
| ENST00000584911 | CATG00000086946.1 | 0.009597 | 0.990403 |
| ENST00000584911 | CATG00000039284.1 | 0.007754 | -0.99225 |
| ENST00000584911 | ORM1 | 0.001751 | 0.998249 |
| ENST00000584911 | MFAP3L | 0.008468 | 0.991532 |
| ENST00000584911 | CATG00000086946.1 | 0.009597 | 0.990403 |
| ENST00000584911 | CATG00000039284.1 | 0.007754 | -0.99225 |
| ENST00000584911 | ORM1 | 0.001751 | 0.998249 |
| ENST00000584911 | MFAP3L | 0.008468 | 0.991532 |
| ENST00000584911 | CATG00000086946.1 | 0.009597 | 0.990403 |
| ENST00000584911 | CATG00000039284.1 | 0.007754 | -0.99225 |
| ENST00000584911 | ORM1 | 0.001751 | 0.998249 |
| ENST00000582348 | TBC1D29 | 4.82E-04 | -0.99952 |
| ENST00000582348 | USP28 | 0.008852 | -0.99115 |
| ENST00000582348 | CCDC42 | 0.007812 | -0.99219 |
| ENST00000582348 | PRR15L | 0.008628 | 0.991372 |
| ENST00000582348 | HTRA4 | 0.007049 | 0.992951 |
| ENST00000582348 | TBC1D29 | 4.82E-04 | -0.99952 |
| ENST00000582348 | USP28 | 0.008852 | -0.99115 |
| ENST00000582348 | CCDC42 | 0.007812 | -0.99219 |
| ENST00000582348 | PRR15L | 0.008628 | 0.991372 |
| ENST00000582348 | HTRA4 | 0.007049 | 0.992951 |
| ENST00000416453 | NR2F2 | 0.004526 | 0.995474 |
| ENST00000416453 | ELOF1 | 0.008535 | 0.991465 |
| ENST00000416453 | RPL23A | 0.001151 | -0.99885 |
| ENST00000416453 | FOXO6 | 0.003541 | 0.996459 |
| ENST00000416453 | DGAT2L6 | 0.003224 | 0.996776 |
| ENST00000416453 | SMPD1 | 0.007259 | 0.992741 |
| ENST00000416453 | DHODH | 0.008253 | -0.99175 |
| ENST00000416453 | SRRD | 0.006305 | 0.993695 |
| ENST00000416453 | TPTE | 0.009815 | 0.990185 |
| ENST00000416453 | CSTL1 | 0.001457 | 0.998543 |
| ENST00000416453 | CEP290 | 0.009319 | 0.990681 |
| ENST00000416453 | WNT2 | 4.18E-04 | 0.999582 |
| ENST00000416453 | JDP2 | 0.009865 | 0.990135 |
| ENST00000416453 | KLF6 | 0.006471 | -0.99353 |
| ENST00000416453 | LBP | 0.005102 | 0.994898 |
| ENST00000416453 | OXT | 0.006063 | 0.993937 |
| ENST00000416453 | REG4 | 0.001482 | -0.99852 |
| ENST00000537710 | ACSBG1 | 0.002101 | 0.997899 |
| ENST00000537710 | USP28 | 0.006132 | 0.993868 |
| ENST00000537710 | PDCD2L | 0.009294 | 0.990706 |
| ENST00000537710 | PRR15L | 0.001317 | -0.99868 |
| ENST00000537710 | FOXJ3 | 0.009635 | -0.99036 |
| ENST00000537710 | ACSBG1 | 0.002101 | 0.997899 |
| ENST00000537710 | USP28 | 0.006132 | 0.993868 |
| ENST00000537710 | PDCD2L | 0.009294 | 0.990706 |
| ENST00000537710 | PRR15L | 0.001317 | -0.99868 |
| ENST00000537710 | FOXJ3 | 0.009635 | -0.99036 |
| ENST00000537710 | ACSBG1 | 0.002101 | 0.997899 |
| ENST00000537710 | USP28 | 0.006132 | 0.993868 |
| ENST00000537710 | PDCD2L | 0.009294 | 0.990706 |
| ENST00000537710 | PRR15L | 0.001317 | -0.99868 |
| ENST00000537710 | FOXJ3 | 0.009635 | -0.99036 |
| ENST00000467519 | BECN1 | 0.003697 | -0.9963 |
| ENST00000467519 | ACTL7B | 0.003912 | 0.996088 |
| ENST00000467519 | NDUFAF8 | 0.00611 | -0.99389 |
| ENST00000467519 | CCDC42 | 0.007582 | -0.99242 |
| ENST00000467519 | MED12L | 0.001016 | 0.998984 |
| ENST00000578334 | PTPN6 | 0.009876 | -0.99012 |
| ENST00000578334 | DNAJA4 | 2.46E-04 | 0.999754 |
| ENST00000578334 | GPR25 | 0.005192 | 0.994808 |
| ENST00000578334 | PMPCA | 0.00473 | 0.99527 |
| ENST00000578334 | CAPNS1 | 0.007416 | -0.99258 |
| ENST00000578334 | SHC2 | 0.001772 | 0.998228 |
| ENST00000578334 | KRTAP4-7 | 0.004804 | 0.995196 |
| ENST00000578334 | PTPN6 | 0.009876 | -0.99012 |
| ENST00000578334 | DNAJA4 | 2.46E-04 | 0.999754 |
| ENST00000578334 | GPR25 | 0.005192 | 0.994808 |
| ENST00000578334 | PMPCA | 0.00473 | 0.99527 |
| ENST00000578334 | CAPNS1 | 0.007416 | -0.99258 |
| ENST00000578334 | SHC2 | 0.001772 | 0.998228 |
| ENST00000578334 | KRTAP4-7 | 0.004804 | 0.995196 |
| NR_146540 | AX748369 | 0.008711 | 0.991289 |
| NR_146540 | ACSBG1 | 0.007814 | -0.99219 |
| NR_146540 | PNPLA2 | 0.005186 | 0.994814 |
| NR_146540 | PDCD2L | 9.19E-04 | -0.99908 |
| NR_146540 | CATG00000108269.1 | 0.002046 | 0.997954 |
| NR_146540 | FOXJ3 | 0.006335 | 0.993665 |
| NR_146540 | CYP27C1 | 0.00306 | 0.99694 |
| NR_147989 | AX748369 | 0.003747 | -0.99625 |
| NR_147989 | PNPLA2 | 0.002484 | -0.99752 |
| NR_147989 | PDCD2L | 0.002042 | 0.997958 |
| NR_147989 | DRC1 | 0.007713 | -0.99229 |
| NR_147989 | FGD4 | 0.00953 | -0.99047 |
| NR_147989 | CATG00000108269.1 | 6.79E-05 | -0.99993 |
| NR_147989 | CYP27C1 | 0.003129 | -0.99687 |
| NR_147989 | AX748369 | 0.003747 | -0.99625 |
| NR_147989 | PNPLA2 | 0.002484 | -0.99752 |
| NR_147989 | PDCD2L | 0.002042 | 0.997958 |
| NR_147989 | DRC1 | 0.007713 | -0.99229 |
| NR_147989 | FGD4 | 0.00953 | -0.99047 |
| NR_147989 | CATG00000108269.1 | 6.79E-05 | -0.99993 |
| NR_147989 | CYP27C1 | 0.003129 | -0.99687 |
| NR_145459 | CATG00000080699.1 | 8.43E-04 | 0.999157 |
| NR_145459 | TCOF1 | 0.007129 | 0.992871 |
| NR_145459 | SLIRP | 0.009794 | 0.990206 |
| NR_145459 | MYD88 | 0.008484 | -0.99152 |
| NR_145459 | SAMD13 | 0.006112 | 0.993888 |
| NR_145459 | PPP5C | 0.008134 | 0.991866 |
| NR_145459 | ZDHHC16 | 0.00625 | -0.99375 |
| NR_145459 | MACROD2 | 0.00728 | 0.99272 |
| NR_145459 | CENPO | 0.007331 | 0.992669 |
| NR_145459 | CHST12 | 0.004011 | 0.995989 |
| NR_145459 | HIPK2 | 0.005244 | -0.99476 |
| NR_145459 | PCDHB8 | 0.004011 | 0.995989 |
| NR_145459 | CATG00000080699.1 | 8.43E-04 | 0.999157 |
| NR_145459 | TCOF1 | 0.007129 | 0.992871 |
| NR_145459 | SLIRP | 0.009794 | 0.990206 |
| NR_145459 | MYD88 | 0.008484 | -0.99152 |
| NR_145459 | SAMD13 | 0.006112 | 0.993888 |
| NR_145459 | PPP5C | 0.008134 | 0.991866 |
| NR_145459 | ZDHHC16 | 0.00625 | -0.99375 |
| NR_145459 | MACROD2 | 0.00728 | 0.99272 |
| NR_145459 | CENPO | 0.007331 | 0.992669 |
| NR_145459 | CHST12 | 0.004011 | 0.995989 |
| NR_145459 | HIPK2 | 0.005244 | -0.99476 |
| NR_145459 | PCDHB8 | 0.004011 | 0.995989 |
| NR_145459 | CATG00000080699.1 | 8.43E-04 | 0.999157 |
| NR_145459 | TCOF1 | 0.007129 | 0.992871 |
| NR_145459 | SLIRP | 0.009794 | 0.990206 |
| NR_145459 | MYD88 | 0.008484 | -0.99152 |
| NR_145459 | SAMD13 | 0.006112 | 0.993888 |
| NR_145459 | PPP5C | 0.008134 | 0.991866 |
| NR_145459 | ZDHHC16 | 0.00625 | -0.99375 |
| NR_145459 | MACROD2 | 0.00728 | 0.99272 |
| NR_145459 | CENPO | 0.007331 | 0.992669 |
| NR_145459 | CHST12 | 0.004011 | 0.995989 |
| NR_145459 | HIPK2 | 0.005244 | -0.99476 |
| NR_145459 | PCDHB8 | 0.004011 | 0.995989 |
| NR_145459 | CATG00000080699.1 | 8.43E-04 | 0.999157 |
| NR_145459 | TCOF1 | 0.007129 | 0.992871 |
| NR_145459 | SLIRP | 0.009794 | 0.990206 |
| NR_145459 | MYD88 | 0.008484 | -0.99152 |
| NR_145459 | SAMD13 | 0.006112 | 0.993888 |
| NR_145459 | PPP5C | 0.008134 | 0.991866 |
| NR_145459 | ZDHHC16 | 0.00625 | -0.99375 |
| NR_145459 | MACROD2 | 0.00728 | 0.99272 |
| NR_145459 | CENPO | 0.007331 | 0.992669 |
| NR_145459 | CHST12 | 0.004011 | 0.995989 |
| NR_145459 | HIPK2 | 0.005244 | -0.99476 |
| NR_145459 | PCDHB8 | 0.004011 | 0.995989 |
| NR_145459 | CATG00000080699.1 | 8.43E-04 | 0.999157 |
| NR_145459 | TCOF1 | 0.007129 | 0.992871 |
| NR_145459 | SLIRP | 0.009794 | 0.990206 |
| NR_145459 | MYD88 | 0.008484 | -0.99152 |
| NR_145459 | SAMD13 | 0.006112 | 0.993888 |
| NR_145459 | PPP5C | 0.008134 | 0.991866 |
| NR_145459 | ZDHHC16 | 0.00625 | -0.99375 |
| NR_145459 | MACROD2 | 0.00728 | 0.99272 |
| NR_145459 | CENPO | 0.007331 | 0.992669 |
| NR_145459 | CHST12 | 0.004011 | 0.995989 |
| NR_145459 | HIPK2 | 0.005244 | -0.99476 |
| NR_145459 | PCDHB8 | 0.004011 | 0.995989 |
| NR_145459 | CATG00000080699.1 | 8.43E-04 | 0.999157 |
| NR_145459 | TCOF1 | 0.007129 | 0.992871 |
| NR_145459 | SLIRP | 0.009794 | 0.990206 |
| NR_145459 | MYD88 | 0.008484 | -0.99152 |
| NR_145459 | SAMD13 | 0.006112 | 0.993888 |
| NR_145459 | PPP5C | 0.008134 | 0.991866 |
| NR_145459 | ZDHHC16 | 0.00625 | -0.99375 |
| NR_145459 | MACROD2 | 0.00728 | 0.99272 |
| NR_145459 | CENPO | 0.007331 | 0.992669 |
| NR_145459 | CHST12 | 0.004011 | 0.995989 |
| NR_145459 | HIPK2 | 0.005244 | -0.99476 |
| NR_145459 | PCDHB8 | 0.004011 | 0.995989 |
| uc001vdn.1 | PLEKHG2 | 0.009685 | 0.990315 |
| uc001vdn.1 | MAPKBP1 | 0.009858 | 0.990142 |
| uc001vdn.1 | CATG00000026669.1 | 0.001072 | -0.99893 |
| uc001vdn.1 | CARNS1 | 7.08E-04 | -0.99929 |
| uc001vdn.1 | PLEKHG2 | 0.009685 | 0.990315 |
| uc001vdn.1 | MAPKBP1 | 0.009858 | 0.990142 |
| uc001vdn.1 | CATG00000026669.1 | 0.001072 | -0.99893 |
| uc001vdn.1 | CARNS1 | 7.08E-04 | -0.99929 |
| ENST00000586544 | INPP5J | 0.007937 | 0.992063 |
| ENST00000586544 | RFC2 | 0.005246 | -0.99475 |
| ENST00000586544 | CATG00000101330.1 | 0.004874 | -0.99513 |
| ENST00000586544 | CNEP1R1 | 2.95E-04 | -0.9997 |
| ENST00000586544 | FRG1 | 0.009429 | -0.99057 |
| ENST00000586544 | SH3D21 | 0.009683 | 0.990317 |
| ENST00000586544 | TP73 | 0.00629 | 0.99371 |
| ENST00000586544 | ARHGEF26 | 0.004375 | 0.995625 |
| ENST00000586544 | PHKA1 | 0.001199 | 0.998801 |
| ENST00000586544 | CATG00000026557.1 | 0.002943 | 0.997057 |
| ENST00000586544 | COA1 | 0.004229 | -0.99577 |
| ENST00000586544 | PSME1 | 0.00777 | -0.99223 |
| ENST00000586544 | DOCK1 | 0.00237 | 0.99763 |
| ENST00000586544 | BMPER | 0.006454 | 0.993546 |
| ENST00000586544 | INPP5J | 0.007937 | 0.992063 |
| ENST00000586544 | RFC2 | 0.005246 | -0.99475 |
| ENST00000586544 | CATG00000101330.1 | 0.004874 | -0.99513 |
| ENST00000586544 | CNEP1R1 | 2.95E-04 | -0.9997 |
| ENST00000586544 | FRG1 | 0.009429 | -0.99057 |
| ENST00000586544 | SH3D21 | 0.009683 | 0.990317 |
| ENST00000586544 | TP73 | 0.00629 | 0.99371 |
| ENST00000586544 | ARHGEF26 | 0.004375 | 0.995625 |
| ENST00000586544 | PHKA1 | 0.001199 | 0.998801 |
| ENST00000586544 | CATG00000026557.1 | 0.002943 | 0.997057 |
| ENST00000586544 | COA1 | 0.004229 | -0.99577 |
| ENST00000586544 | PSME1 | 0.00777 | -0.99223 |
| ENST00000586544 | DOCK1 | 0.00237 | 0.99763 |
| ENST00000586544 | BMPER | 0.006454 | 0.993546 |
| ENST00000586544 | INPP5J | 0.007937 | 0.992063 |
| ENST00000586544 | RFC2 | 0.005246 | -0.99475 |
| ENST00000586544 | CATG00000101330.1 | 0.004874 | -0.99513 |
| ENST00000586544 | CNEP1R1 | 2.95E-04 | -0.9997 |
| ENST00000586544 | FRG1 | 0.009429 | -0.99057 |
| ENST00000586544 | SH3D21 | 0.009683 | 0.990317 |
| ENST00000586544 | TP73 | 0.00629 | 0.99371 |
| ENST00000586544 | ARHGEF26 | 0.004375 | 0.995625 |
| ENST00000586544 | PHKA1 | 0.001199 | 0.998801 |
| ENST00000586544 | CATG00000026557.1 | 0.002943 | 0.997057 |
| ENST00000586544 | COA1 | 0.004229 | -0.99577 |
| ENST00000586544 | PSME1 | 0.00777 | -0.99223 |
| ENST00000586544 | DOCK1 | 0.00237 | 0.99763 |
| ENST00000586544 | BMPER | 0.006454 | 0.993546 |
| ENST00000586544 | INPP5J | 0.007937 | 0.992063 |
| ENST00000586544 | RFC2 | 0.005246 | -0.99475 |
| ENST00000586544 | CATG00000101330.1 | 0.004874 | -0.99513 |
| ENST00000586544 | CNEP1R1 | 2.95E-04 | -0.9997 |
| ENST00000586544 | FRG1 | 0.009429 | -0.99057 |
| ENST00000586544 | SH3D21 | 0.009683 | 0.990317 |
| ENST00000586544 | TP73 | 0.00629 | 0.99371 |
| ENST00000586544 | ARHGEF26 | 0.004375 | 0.995625 |
| ENST00000586544 | PHKA1 | 0.001199 | 0.998801 |
| ENST00000586544 | CATG00000026557.1 | 0.002943 | 0.997057 |
| ENST00000586544 | COA1 | 0.004229 | -0.99577 |
| ENST00000586544 | PSME1 | 0.00777 | -0.99223 |
| ENST00000586544 | DOCK1 | 0.00237 | 0.99763 |
| ENST00000586544 | BMPER | 0.006454 | 0.993546 |
| ENST00000586544 | INPP5J | 0.007937 | 0.992063 |
| ENST00000586544 | RFC2 | 0.005246 | -0.99475 |
| ENST00000586544 | CATG00000101330.1 | 0.004874 | -0.99513 |
| ENST00000586544 | CNEP1R1 | 2.95E-04 | -0.9997 |
| ENST00000586544 | FRG1 | 0.009429 | -0.99057 |
| ENST00000586544 | SH3D21 | 0.009683 | 0.990317 |
| ENST00000586544 | TP73 | 0.00629 | 0.99371 |
| ENST00000586544 | ARHGEF26 | 0.004375 | 0.995625 |
| ENST00000586544 | PHKA1 | 0.001199 | 0.998801 |
| ENST00000586544 | CATG00000026557.1 | 0.002943 | 0.997057 |
| ENST00000586544 | COA1 | 0.004229 | -0.99577 |
| ENST00000586544 | PSME1 | 0.00777 | -0.99223 |
| ENST00000586544 | DOCK1 | 0.00237 | 0.99763 |
| ENST00000586544 | BMPER | 0.006454 | 0.993546 |
| ENST00000586544 | INPP5J | 0.007937 | 0.992063 |
| ENST00000586544 | RFC2 | 0.005246 | -0.99475 |
| ENST00000586544 | CATG00000101330.1 | 0.004874 | -0.99513 |
| ENST00000586544 | CNEP1R1 | 2.95E-04 | -0.9997 |
| ENST00000586544 | FRG1 | 0.009429 | -0.99057 |
| ENST00000586544 | SH3D21 | 0.009683 | 0.990317 |
| ENST00000586544 | TP73 | 0.00629 | 0.99371 |
| ENST00000586544 | ARHGEF26 | 0.004375 | 0.995625 |
| ENST00000586544 | PHKA1 | 0.001199 | 0.998801 |
| ENST00000586544 | CATG00000026557.1 | 0.002943 | 0.997057 |
| ENST00000586544 | COA1 | 0.004229 | -0.99577 |
| ENST00000586544 | PSME1 | 0.00777 | -0.99223 |
| ENST00000586544 | DOCK1 | 0.00237 | 0.99763 |
| ENST00000586544 | BMPER | 0.006454 | 0.993546 |
| NR_145459 | CATG00000080699.1 | 8.43E-04 | 0.999157 |
| NR_145459 | TCOF1 | 0.007129 | 0.992871 |
| NR_145459 | SLIRP | 0.009794 | 0.990206 |
| NR_145459 | MYD88 | 0.008484 | -0.99152 |
| NR_145459 | SAMD13 | 0.006112 | 0.993888 |
| NR_145459 | PPP5C | 0.008134 | 0.991866 |
| NR_145459 | ZDHHC16 | 0.00625 | -0.99375 |
| NR_145459 | MACROD2 | 0.00728 | 0.99272 |
| NR_145459 | CENPO | 0.007331 | 0.992669 |
| NR_145459 | CHST12 | 0.004011 | 0.995989 |
| NR_145459 | HIPK2 | 0.005244 | -0.99476 |
| NR_145459 | PCDHB8 | 0.004011 | 0.995989 |
| ENST00000495673 | CATG00000063531.1 | 6.77E-04 | 0.999323 |
| ENST00000495673 | UBE2V1 | 5.12E-04 | 0.999488 |
| ENST00000495673 | CEP44 | 7.35E-04 | 0.999265 |
| ENST00000495673 | RASSF6 | 0.009977 | 0.990023 |
| ENST00000495673 | PCBP4 | 7.93E-04 | -0.99921 |
| ENST00000495673 | ABR | 0.00642 | 0.99358 |
| ENST00000495673 | OR5M8 | 0.008599 | 0.991401 |
| ENST00000495673 | NTF3 | 0.008714 | 0.991286 |
| ENST00000495673 | RTL8B | 0.00315 | -0.99685 |
| ENST00000495673 | ERVW-1 | 2.84E-04 | -0.99972 |
| ENST00000495673 | CATG00000117842.1 | 0.004411 | 0.995589 |
| ENST00000495673 | C16orf86 | 0.003514 | -0.99649 |
| ENST00000495673 | CATG00000063531.1 | 6.77E-04 | 0.999323 |
| ENST00000495673 | UBE2V1 | 5.12E-04 | 0.999488 |
| ENST00000495673 | CEP44 | 7.35E-04 | 0.999265 |
| ENST00000495673 | RASSF6 | 0.009977 | 0.990023 |
| ENST00000495673 | PCBP4 | 7.93E-04 | -0.99921 |
| ENST00000495673 | ABR | 0.00642 | 0.99358 |
| ENST00000495673 | OR5M8 | 0.008599 | 0.991401 |
| ENST00000495673 | NTF3 | 0.008714 | 0.991286 |
| ENST00000495673 | RTL8B | 0.00315 | -0.99685 |
| ENST00000495673 | ERVW-1 | 2.84E-04 | -0.99972 |
| ENST00000495673 | CATG00000117842.1 | 0.004411 | 0.995589 |
| ENST00000495673 | C16orf86 | 0.003514 | -0.99649 |
| ENST00000584911 | MFAP3L | 0.008468 | 0.991532 |
| ENST00000584911 | CATG00000086946.1 | 0.009597 | 0.990403 |
| ENST00000584911 | CATG00000039284.1 | 0.007754 | -0.99225 |
| ENST00000584911 | ORM1 | 0.001751 | 0.998249 |
| ENST00000584911 | MFAP3L | 0.008468 | 0.991532 |
| ENST00000584911 | CATG00000086946.1 | 0.009597 | 0.990403 |
| ENST00000584911 | CATG00000039284.1 | 0.007754 | -0.99225 |
| ENST00000584911 | ORM1 | 0.001751 | 0.998249 |
| ENST00000584911 | MFAP3L | 0.008468 | 0.991532 |
| ENST00000584911 | CATG00000086946.1 | 0.009597 | 0.990403 |
| ENST00000584911 | CATG00000039284.1 | 0.007754 | -0.99225 |
| ENST00000584911 | ORM1 | 0.001751 | 0.998249 |
| ENST00000584911 | MFAP3L | 0.008468 | 0.991532 |
| ENST00000584911 | CATG00000086946.1 | 0.009597 | 0.990403 |
| ENST00000584911 | CATG00000039284.1 | 0.007754 | -0.99225 |
| ENST00000584911 | ORM1 | 0.001751 | 0.998249 |
| ENST00000584911 | MFAP3L | 0.008468 | 0.991532 |
| ENST00000584911 | CATG00000086946.1 | 0.009597 | 0.990403 |
| ENST00000584911 | CATG00000039284.1 | 0.007754 | -0.99225 |
| ENST00000584911 | ORM1 | 0.001751 | 0.998249 |
| ENST00000584911 | MFAP3L | 0.008468 | 0.991532 |
| ENST00000584911 | CATG00000086946.1 | 0.009597 | 0.990403 |
| ENST00000584911 | CATG00000039284.1 | 0.007754 | -0.99225 |
| ENST00000584911 | ORM1 | 0.001751 | 0.998249 |
| ENST00000584911 | MFAP3L | 0.008468 | 0.991532 |
| ENST00000584911 | CATG00000086946.1 | 0.009597 | 0.990403 |
| ENST00000584911 | CATG00000039284.1 | 0.007754 | -0.99225 |
| ENST00000584911 | ORM1 | 0.001751 | 0.998249 |
| ENST00000584911 | MFAP3L | 0.008468 | 0.991532 |
| ENST00000584911 | CATG00000086946.1 | 0.009597 | 0.990403 |
| ENST00000584911 | CATG00000039284.1 | 0.007754 | -0.99225 |
| ENST00000584911 | ORM1 | 0.001751 | 0.998249 |
| NR_027889 | HEXIM2 | 0.002317 | -0.99768 |
| NR_027889 | OCLN | 9.67E-04 | -0.99903 |
| NR_027889 | FAR1 | 0.001455 | -0.99855 |
| NR_027889 | TRIM73 | 0.004604 | -0.9954 |
| NR_027889 | LTF | 0.004088 | 0.995912 |
| NR_027889 | KIF27 | 0.001022 | -0.99898 |
| NR_027889 | HEXIM2 | 0.002317 | -0.99768 |
| NR_027889 | OCLN | 9.67E-04 | -0.99903 |
| NR_027889 | FAR1 | 0.001455 | -0.99855 |
| NR_027889 | TRIM73 | 0.004604 | -0.9954 |
| NR_027889 | LTF | 0.004088 | 0.995912 |
| NR_027889 | KIF27 | 0.001022 | -0.99898 |
| NR_027889 | HEXIM2 | 0.002317 | -0.99768 |
| NR_027889 | OCLN | 9.67E-04 | -0.99903 |
| NR_027889 | FAR1 | 0.001455 | -0.99855 |
| NR_027889 | TRIM73 | 0.004604 | -0.9954 |
| NR_027889 | LTF | 0.004088 | 0.995912 |
| NR_027889 | KIF27 | 0.001022 | -0.99898 |
| NR_027889 | HEXIM2 | 0.002317 | -0.99768 |
| NR_027889 | OCLN | 9.67E-04 | -0.99903 |
| NR_027889 | FAR1 | 0.001455 | -0.99855 |
| NR_027889 | TRIM73 | 0.004604 | -0.9954 |
| NR_027889 | LTF | 0.004088 | 0.995912 |
| NR_027889 | KIF27 | 0.001022 | -0.99898 |
| NR_027889 | HEXIM2 | 0.002317 | -0.99768 |
| NR_027889 | OCLN | 9.67E-04 | -0.99903 |
| NR_027889 | FAR1 | 0.001455 | -0.99855 |
| NR_027889 | TRIM73 | 0.004604 | -0.9954 |
| NR_027889 | LTF | 0.004088 | 0.995912 |
| NR_027889 | KIF27 | 0.001022 | -0.99898 |
| ENST00000524319 | NAPA | 0.008368 | 0.991632 |
| ENST00000524319 | PPP1CB | 0.002223 | 0.997777 |
| ENST00000524319 | PGM5 | 0.003171 | -0.99683 |
| ENST00000524319 | CATG00000107158.1 | 0.002747 | 0.997253 |
| ENST00000524319 | CATG00000110054.1 | 0.005415 | 0.994585 |
| ENST00000524319 | SPRR1A | 0.009492 | 0.990508 |
| ENST00000524319 | YY2 | 0.007592 | -0.99241 |
| ENST00000524319 | RCSD1 | 0.007533 | -0.99247 |
| ENST00000524319 | LY6G6C | 0.007774 | -0.99223 |
| ENST00000524319 | CFLAR | 0.005659 | -0.99434 |
| ENST00000524319 | CHI3L1 | 0.00152 | 0.99848 |
| ENST00000524319 | C17orf47 | 0.007758 | 0.992242 |
| ENST00000524319 | CATG00000020281.1 | 0.00309 | -0.99691 |
| ENST00000575542 | AX748369 | 0.003441 | -0.99656 |
| ENST00000575542 | GMPPA | 0.008387 | 0.991613 |
| ENST00000575542 | PNPLA2 | 0.001057 | -0.99894 |
| ENST00000575542 | PDCD2L | 0.003602 | 0.996398 |
| ENST00000575542 | DRC1 | 0.006064 | -0.99394 |
| ENST00000575542 | CATG00000108269.1 | 0.005822 | -0.99418 |
| ENST00000575542 | EFCAB8 | 0.008128 | -0.99187 |
| ENST00000575542 | FOXJ3 | 0.008924 | -0.99108 |
| ENST00000575542 | CYP27C1 | 0.001151 | -0.99885 |
| ENST00000575542 | MSI1 | 6.63E-04 | -0.99934 |
| ENST00000575542 | AX748369 | 0.003441 | -0.99656 |
| ENST00000575542 | GMPPA | 0.008387 | 0.991613 |
| ENST00000575542 | PNPLA2 | 0.001057 | -0.99894 |
| ENST00000575542 | PDCD2L | 0.003602 | 0.996398 |
| ENST00000575542 | DRC1 | 0.006064 | -0.99394 |
| ENST00000575542 | CATG00000108269.1 | 0.005822 | -0.99418 |
| ENST00000575542 | EFCAB8 | 0.008128 | -0.99187 |
| ENST00000575542 | FOXJ3 | 0.008924 | -0.99108 |
| ENST00000575542 | CYP27C1 | 0.001151 | -0.99885 |
| ENST00000575542 | MSI1 | 6.63E-04 | -0.99934 |
| ENST00000582348 | TBC1D29 | 4.82E-04 | -0.99952 |
| ENST00000582348 | USP28 | 0.008852 | -0.99115 |
| ENST00000582348 | CCDC42 | 0.007812 | -0.99219 |
| ENST00000582348 | PRR15L | 0.008628 | 0.991372 |
| ENST00000582348 | HTRA4 | 0.007049 | 0.992951 |
| ENST00000416453 | NR2F2 | 0.004526 | 0.995474 |
| ENST00000416453 | ELOF1 | 0.008535 | 0.991465 |
| ENST00000416453 | RPL23A | 0.001151 | -0.99885 |
| ENST00000416453 | FOXO6 | 0.003541 | 0.996459 |
| ENST00000416453 | DGAT2L6 | 0.003224 | 0.996776 |
| ENST00000416453 | SMPD1 | 0.007259 | 0.992741 |
| ENST00000416453 | DHODH | 0.008253 | -0.99175 |
| ENST00000416453 | SRRD | 0.006305 | 0.993695 |
| ENST00000416453 | TPTE | 0.009815 | 0.990185 |
| ENST00000416453 | CSTL1 | 0.001457 | 0.998543 |
| ENST00000416453 | CEP290 | 0.009319 | 0.990681 |
| ENST00000416453 | WNT2 | 4.18E-04 | 0.999582 |
| ENST00000416453 | JDP2 | 0.009865 | 0.990135 |
| ENST00000416453 | KLF6 | 0.006471 | -0.99353 |
| ENST00000416453 | LBP | 0.005102 | 0.994898 |
| ENST00000416453 | OXT | 0.006063 | 0.993937 |
| ENST00000416453 | REG4 | 0.001482 | -0.99852 |
| ENST00000537710 | ACSBG1 | 0.002101 | 0.997899 |
| ENST00000537710 | USP28 | 0.006132 | 0.993868 |
| ENST00000537710 | PDCD2L | 0.009294 | 0.990706 |
| ENST00000537710 | PRR15L | 0.001317 | -0.99868 |
| ENST00000537710 | FOXJ3 | 0.009635 | -0.99036 |
| ENST00000537710 | ACSBG1 | 0.002101 | 0.997899 |
| ENST00000537710 | USP28 | 0.006132 | 0.993868 |
| ENST00000537710 | PDCD2L | 0.009294 | 0.990706 |
| ENST00000537710 | PRR15L | 0.001317 | -0.99868 |
| ENST00000537710 | FOXJ3 | 0.009635 | -0.99036 |
| ENST00000537710 | ACSBG1 | 0.002101 | 0.997899 |
| ENST00000537710 | USP28 | 0.006132 | 0.993868 |
| ENST00000537710 | PDCD2L | 0.009294 | 0.990706 |
| ENST00000537710 | PRR15L | 0.001317 | -0.99868 |
| ENST00000537710 | FOXJ3 | 0.009635 | -0.99036 |
| ENST00000535127 | B3GNT3 | 0.00359 | -0.99641 |
| ENST00000535127 | HMGB2 | 0.005154 | -0.99485 |
| ENST00000535127 | PLIN4 | 0.008719 | 0.991281 |
| ENST00000535127 | FOXO6 | 5.16E-04 | 0.999484 |
| ENST00000535127 | CXXC1 | 0.009267 | -0.99073 |
| ENST00000535127 | DGAT2L6 | 0.005706 | 0.994294 |
| ENST00000535127 | CATG00000053512.1 | 0.009431 | -0.99057 |
| ENST00000535127 | HLF | 0.005073 | 0.994927 |
| ENST00000535127 | SRRD | 7.56E-04 | 0.999244 |
| ENST00000535127 | TPTE | 0.002828 | 0.997172 |
| ENST00000535127 | CSTL1 | 0.002263 | 0.997737 |
| ENST00000535127 | FYB2 | 0.001127 | 0.998873 |
| ENST00000535127 | SLC12A3 | 0.008388 | -0.99161 |
| ENST00000535127 | WNT2 | 0.007896 | 0.992104 |
| ENST00000535127 | PAK3 | 0.005365 | 0.994635 |
| ENST00000535127 | PPP5D1 | 0.008878 | 0.991122 |
| ENST00000535127 | OXT | 2.04E-04 | 0.999796 |
| ENST00000535127 | FAM174A | 0.001547 | -0.99845 |
| ENST00000535127 | REG4 | 0.009496 | -0.9905 |
| ENST00000535127 | CFAP410 | 0.006315 | 0.993685 |
| ENST00000467519 | BECN1 | 0.003697 | -0.9963 |
| ENST00000467519 | ACTL7B | 0.003912 | 0.996088 |
| ENST00000467519 | NDUFAF8 | 0.00611 | -0.99389 |
| ENST00000467519 | CCDC42 | 0.007582 | -0.99242 |
| ENST00000467519 | MED12L | 0.001016 | 0.998984 |
| ENST00000498176 | GNL1 | 0.007991 | -0.99201 |
| ENST00000498176 | HMGXB4 | 0.008579 | -0.99142 |
| ENST00000498176 | DNAJA4 | 0.001322 | 0.998678 |
| ENST00000498176 | GPR25 | 0.003733 | 0.996267 |
| ENST00000498176 | PMPCA | 0.002888 | 0.997112 |
| ENST00000498176 | CAPNS1 | 0.006047 | -0.99395 |
| ENST00000498176 | SHC2 | 0.003805 | 0.996195 |
| ENST00000498176 | KRTAP4-7 | 0.004203 | 0.995797 |
| NR_146084 | C1orf198 | 6.10E-04 | -0.99939 |
| NR_146084 | TATDN3 | 0.002584 | -0.99742 |
| NR_146084 | BVES | 0.00398 | 0.99602 |
| NR_146084 | COMMD2 | 0.002373 | -0.99763 |
| NR_146084 | MRPL10 | 0.003031 | 0.996969 |
| NR_146084 | ANKS3 | 0.00718 | 0.99282 |
| NR_146084 | ATXN7 | 0.009109 | 0.990891 |
| NR_146084 | ASPRV1 | 0.006818 | -0.99318 |
| NR_146084 | SMIM33 | 0.005447 | -0.99455 |
| NR_146084 | C1orf198 | 6.10E-04 | -0.99939 |
| NR_146084 | TATDN3 | 0.002584 | -0.99742 |
| NR_146084 | BVES | 0.00398 | 0.99602 |
| NR_146084 | COMMD2 | 0.002373 | -0.99763 |
| NR_146084 | MRPL10 | 0.003031 | 0.996969 |
| NR_146084 | ANKS3 | 0.00718 | 0.99282 |
| NR_146084 | ATXN7 | 0.009109 | 0.990891 |
| NR_146084 | ASPRV1 | 0.006818 | -0.99318 |
| NR_146084 | SMIM33 | 0.005447 | -0.99455 |
| NR_146084 | C1orf198 | 6.10E-04 | -0.99939 |
| NR_146084 | TATDN3 | 0.002584 | -0.99742 |
| NR_146084 | BVES | 0.00398 | 0.99602 |
| NR_146084 | COMMD2 | 0.002373 | -0.99763 |
| NR_146084 | MRPL10 | 0.003031 | 0.996969 |
| NR_146084 | ANKS3 | 0.00718 | 0.99282 |
| NR_146084 | ATXN7 | 0.009109 | 0.990891 |
| NR_146084 | ASPRV1 | 0.006818 | -0.99318 |
| NR_146084 | SMIM33 | 0.005447 | -0.99455 |
| ENST00000380466 | SYNDIG1L | 0.008281 | -0.99172 |
| ENST00000380466 | IL17RA | 0.003961 | 0.996039 |
| ENST00000380466 | AHSP | 0.001768 | 0.998232 |
| ENST00000380466 | AC092073.1 | 9.59E-04 | 0.999041 |
| ENST00000380466 | TGFBR3 | 0.002516 | 0.997484 |
| ENST00000380466 | LYPD1 | 0.006376 | 0.993624 |
| ENST00000380466 | CATG00000034210.1 | 0.009751 | 0.990249 |
| ENST00000380466 | COA5 | 0.009134 | -0.99087 |
| ENST00000380466 | SLC6A5 | 0.003486 | 0.996514 |
| ENST00000380466 | RRBP1 | 0.001065 | -0.99894 |
| ENST00000380466 | MORN3 | 0.003442 | -0.99656 |
| ENST00000380466 | VPS13A | 0.002792 | 0.997208 |
| ENST00000380466 | CAPN15 | 0.005876 | 0.994124 |
| ENST00000578334 | PTPN6 | 0.009876 | -0.99012 |
| ENST00000578334 | DNAJA4 | 2.46E-04 | 0.999754 |
| ENST00000578334 | GPR25 | 0.005192 | 0.994808 |
| ENST00000578334 | PMPCA | 0.00473 | 0.99527 |
| ENST00000578334 | CAPNS1 | 0.007416 | -0.99258 |
| ENST00000578334 | SHC2 | 0.001772 | 0.998228 |
| ENST00000578334 | KRTAP4-7 | 0.004804 | 0.995196 |
| ENST00000326677 | ZNF490 | 0.001892 | 0.998108 |
| ENST00000326677 | HEYL | 0.003354 | -0.99665 |
| ENST00000326677 | TMEM236 | 0.007759 | 0.992241 |
| ENST00000326677 | SCYGR6 | 0.001906 | 0.998094 |
| ENST00000326677 | BRSK2 | 1.96E-04 | 0.999804 |
| ENST00000326677 | GPCPD1 | 0.009027 | 0.990973 |
| ENST00000326677 | LCE4A | 0.002411 | -0.99759 |
| ENST00000326677 | LYPD8 | 0.007023 | -0.99298 |
| ENST00000326677 | SCRG1 | 7.52E-05 | -0.99992 |
| ENST00000326677 | ZFYVE26 | 0.003954 | -0.99605 |
| ENST00000534317 | CATG00000056264.1 | 0.002078 | -0.99792 |
| ENST00000534317 | CATG00000003494.1 | 4.86E-04 | -0.99951 |
| ENST00000534317 | SSBP2 | 0.006396 | -0.9936 |
| ENST00000534317 | CACHD1 | 0.006493 | -0.99351 |
| ENST00000534317 | CHST8 | 0.008256 | -0.99174 |
| ENST00000534317 | CLEC4G | 8.52E-04 | -0.99915 |
| ENST00000534317 | CD27 | 0.009898 | -0.9901 |
| ENST00000534317 | TMC2 | 0.003745 | -0.99625 |
| ENST00000534317 | TPRX1 | 0.003024 | -0.99698 |
| ENST00000534317 | OAS1 | 0.009063 | -0.99094 |
| ENST00000534317 | CLCNKB | 0.001738 | -0.99826 |
| ENST00000534317 | INO80B | 6.32E-04 | -0.99937 |
| ENST00000534317 | ACP6 | 0.009474 | 0.990526 |
| ENST00000534317 | RPL17 | 0.007495 | 0.992505 |
| ENST00000534317 | PPTC7 | 0.00766 | 0.99234 |
| ENST00000534317 | PLCL1 | 0.007266 | 0.992734 |
| ENST00000534317 | GABPA | 0.001724 | -0.99828 |
| ENST00000534317 | CIB1 | 0.009198 | 0.990802 |
| ENST00000422366 | UBE2V1 | 0.005952 | -0.99405 |
| ENST00000422366 | FTCD | 0.005498 | 0.994502 |
| ENST00000422366 | ZNF77 | 8.85E-04 | -0.99911 |
| ENST00000422366 | INHBA | 0.009272 | 0.990728 |
| NR_146540 | AX748369 | 0.008711 | 0.991289 |
| NR_146540 | ACSBG1 | 0.007814 | -0.99219 |
| NR_146540 | PNPLA2 | 0.005186 | 0.994814 |
| NR_146540 | PDCD2L | 9.19E-04 | -0.99908 |
| NR_146540 | CATG00000108269.1 | 0.002046 | 0.997954 |
| NR_146540 | FOXJ3 | 0.006335 | 0.993665 |
| NR_146540 | CYP27C1 | 0.00306 | 0.99694 |
| NR_045406 | DNAJC6 | 0.008527 | -0.99147 |
| NR_045406 | NAAA | 0.007037 | 0.992963 |
| NR_045406 | RFC5 | 0.006306 | -0.99369 |
| NR_045406 | SPACA9 | 0.009568 | -0.99043 |
| NR_045406 | CMBL | 0.001479 | -0.99852 |
| NR_045406 | DARS | 0.001761 | 0.998239 |
| NR_045406 | CATG00000038628.1 | 0.00617 | -0.99383 |
| NR_045406 | OR5T2 | 0.003901 | 0.996099 |
| NR_145459 | CATG00000080699.1 | 8.43E-04 | 0.999157 |
| NR_145459 | TCOF1 | 0.007129 | 0.992871 |
| NR_145459 | SLIRP | 0.009794 | 0.990206 |
| NR_145459 | MYD88 | 0.008484 | -0.99152 |
| NR_145459 | SAMD13 | 0.006112 | 0.993888 |
| NR_145459 | PPP5C | 0.008134 | 0.991866 |
| NR_145459 | ZDHHC16 | 0.00625 | -0.99375 |
| NR_145459 | MACROD2 | 0.00728 | 0.99272 |
| NR_145459 | CENPO | 0.007331 | 0.992669 |
| NR_145459 | CHST12 | 0.004011 | 0.995989 |
| NR_145459 | HIPK2 | 0.005244 | -0.99476 |
| NR_145459 | PCDHB8 | 0.004011 | 0.995989 |
| NR_145459 | CATG00000080699.1 | 8.43E-04 | 0.999157 |
| NR_145459 | TCOF1 | 0.007129 | 0.992871 |
| NR_145459 | SLIRP | 0.009794 | 0.990206 |
| NR_145459 | MYD88 | 0.008484 | -0.99152 |
| NR_145459 | SAMD13 | 0.006112 | 0.993888 |
| NR_145459 | PPP5C | 0.008134 | 0.991866 |
| NR_145459 | ZDHHC16 | 0.00625 | -0.99375 |
| NR_145459 | MACROD2 | 0.00728 | 0.99272 |
| NR_145459 | CENPO | 0.007331 | 0.992669 |
| NR_145459 | CHST12 | 0.004011 | 0.995989 |
| NR_145459 | HIPK2 | 0.005244 | -0.99476 |
| NR_145459 | PCDHB8 | 0.004011 | 0.995989 |
| NR_145459 | CATG00000080699.1 | 8.43E-04 | 0.999157 |
| NR_145459 | TCOF1 | 0.007129 | 0.992871 |
| NR_145459 | SLIRP | 0.009794 | 0.990206 |
| NR_145459 | MYD88 | 0.008484 | -0.99152 |
| NR_145459 | SAMD13 | 0.006112 | 0.993888 |
| NR_145459 | PPP5C | 0.008134 | 0.991866 |
| NR_145459 | ZDHHC16 | 0.00625 | -0.99375 |
| NR_145459 | MACROD2 | 0.00728 | 0.99272 |
| NR_145459 | CENPO | 0.007331 | 0.992669 |
| NR_145459 | CHST12 | 0.004011 | 0.995989 |
| NR_145459 | HIPK2 | 0.005244 | -0.99476 |
| NR_145459 | PCDHB8 | 0.004011 | 0.995989 |
| NR_145459 | CATG00000080699.1 | 8.43E-04 | 0.999157 |
| NR_145459 | TCOF1 | 0.007129 | 0.992871 |
| NR_145459 | SLIRP | 0.009794 | 0.990206 |
| NR_145459 | MYD88 | 0.008484 | -0.99152 |
| NR_145459 | SAMD13 | 0.006112 | 0.993888 |
| NR_145459 | PPP5C | 0.008134 | 0.991866 |
| NR_145459 | ZDHHC16 | 0.00625 | -0.99375 |
| NR_145459 | MACROD2 | 0.00728 | 0.99272 |
| NR_145459 | CENPO | 0.007331 | 0.992669 |
| NR_145459 | CHST12 | 0.004011 | 0.995989 |
| NR_145459 | HIPK2 | 0.005244 | -0.99476 |
| NR_145459 | PCDHB8 | 0.004011 | 0.995989 |
| NR_145459 | CATG00000080699.1 | 8.43E-04 | 0.999157 |
| NR_145459 | TCOF1 | 0.007129 | 0.992871 |
| NR_145459 | SLIRP | 0.009794 | 0.990206 |
| NR_145459 | MYD88 | 0.008484 | -0.99152 |
| NR_145459 | SAMD13 | 0.006112 | 0.993888 |
| NR_145459 | PPP5C | 0.008134 | 0.991866 |
| NR_145459 | ZDHHC16 | 0.00625 | -0.99375 |
| NR_145459 | MACROD2 | 0.00728 | 0.99272 |
| NR_145459 | CENPO | 0.007331 | 0.992669 |
| NR_145459 | CHST12 | 0.004011 | 0.995989 |
| NR_145459 | HIPK2 | 0.005244 | -0.99476 |
| NR_145459 | PCDHB8 | 0.004011 | 0.995989 |
| NR_145459 | CATG00000080699.1 | 8.43E-04 | 0.999157 |
| NR_145459 | TCOF1 | 0.007129 | 0.992871 |
| NR_145459 | SLIRP | 0.009794 | 0.990206 |
| NR_145459 | MYD88 | 0.008484 | -0.99152 |
| NR_145459 | SAMD13 | 0.006112 | 0.993888 |
| NR_145459 | PPP5C | 0.008134 | 0.991866 |
| NR_145459 | ZDHHC16 | 0.00625 | -0.99375 |
| NR_145459 | MACROD2 | 0.00728 | 0.99272 |
| NR_145459 | CENPO | 0.007331 | 0.992669 |
| NR_145459 | CHST12 | 0.004011 | 0.995989 |
| NR_145459 | HIPK2 | 0.005244 | -0.99476 |
| NR_145459 | PCDHB8 | 0.004011 | 0.995989 |
| NR_145459 | CATG00000080699.1 | 8.43E-04 | 0.999157 |
| NR_145459 | TCOF1 | 0.007129 | 0.992871 |
| NR_145459 | SLIRP | 0.009794 | 0.990206 |
| NR_145459 | MYD88 | 0.008484 | -0.99152 |
| NR_145459 | SAMD13 | 0.006112 | 0.993888 |
| NR_145459 | PPP5C | 0.008134 | 0.991866 |
| NR_145459 | ZDHHC16 | 0.00625 | -0.99375 |
| NR_145459 | MACROD2 | 0.00728 | 0.99272 |
| NR_145459 | CENPO | 0.007331 | 0.992669 |
| NR_145459 | CHST12 | 0.004011 | 0.995989 |
| NR_145459 | HIPK2 | 0.005244 | -0.99476 |
| NR_145459 | PCDHB8 | 0.004011 | 0.995989 |
| NR_145459 | CATG00000080699.1 | 8.43E-04 | 0.999157 |
| NR_145459 | TCOF1 | 0.007129 | 0.992871 |
| NR_145459 | SLIRP | 0.009794 | 0.990206 |
| NR_145459 | MYD88 | 0.008484 | -0.99152 |
| NR_145459 | SAMD13 | 0.006112 | 0.993888 |
| NR_145459 | PPP5C | 0.008134 | 0.991866 |
| NR_145459 | ZDHHC16 | 0.00625 | -0.99375 |
| NR_145459 | MACROD2 | 0.00728 | 0.99272 |
| NR_145459 | CENPO | 0.007331 | 0.992669 |
| NR_145459 | CHST12 | 0.004011 | 0.995989 |
| NR_145459 | HIPK2 | 0.005244 | -0.99476 |
| NR_145459 | PCDHB8 | 0.004011 | 0.995989 |
| NR_145459 | CATG00000080699.1 | 8.43E-04 | 0.999157 |
| NR_145459 | TCOF1 | 0.007129 | 0.992871 |
| NR_145459 | SLIRP | 0.009794 | 0.990206 |
| NR_145459 | MYD88 | 0.008484 | -0.99152 |
| NR_145459 | SAMD13 | 0.006112 | 0.993888 |
| NR_145459 | PPP5C | 0.008134 | 0.991866 |
| NR_145459 | ZDHHC16 | 0.00625 | -0.99375 |
| NR_145459 | MACROD2 | 0.00728 | 0.99272 |
| NR_145459 | CENPO | 0.007331 | 0.992669 |
| NR_145459 | CHST12 | 0.004011 | 0.995989 |
| NR_145459 | HIPK2 | 0.005244 | -0.99476 |
| NR_145459 | PCDHB8 | 0.004011 | 0.995989 |
| NR_145459 | CATG00000080699.1 | 8.43E-04 | 0.999157 |
| NR_145459 | TCOF1 | 0.007129 | 0.992871 |
| NR_145459 | SLIRP | 0.009794 | 0.990206 |
| NR_145459 | MYD88 | 0.008484 | -0.99152 |
| NR_145459 | SAMD13 | 0.006112 | 0.993888 |
| NR_145459 | PPP5C | 0.008134 | 0.991866 |
| NR_145459 | ZDHHC16 | 0.00625 | -0.99375 |
| NR_145459 | MACROD2 | 0.00728 | 0.99272 |
| NR_145459 | CENPO | 0.007331 | 0.992669 |
| NR_145459 | CHST12 | 0.004011 | 0.995989 |
| NR_145459 | HIPK2 | 0.005244 | -0.99476 |
| NR_145459 | PCDHB8 | 0.004011 | 0.995989 |
| NR_145459 | CATG00000080699.1 | 8.43E-04 | 0.999157 |
| NR_145459 | TCOF1 | 0.007129 | 0.992871 |
| NR_145459 | SLIRP | 0.009794 | 0.990206 |
| NR_145459 | MYD88 | 0.008484 | -0.99152 |
| NR_145459 | SAMD13 | 0.006112 | 0.993888 |
| NR_145459 | PPP5C | 0.008134 | 0.991866 |
| NR_145459 | ZDHHC16 | 0.00625 | -0.99375 |
| NR_145459 | MACROD2 | 0.00728 | 0.99272 |
| NR_145459 | CENPO | 0.007331 | 0.992669 |
| NR_145459 | CHST12 | 0.004011 | 0.995989 |
| NR_145459 | HIPK2 | 0.005244 | -0.99476 |
| NR_145459 | PCDHB8 | 0.004011 | 0.995989 |
| NR_145459 | CATG00000080699.1 | 8.43E-04 | 0.999157 |
| NR_145459 | TCOF1 | 0.007129 | 0.992871 |
| NR_145459 | SLIRP | 0.009794 | 0.990206 |
| NR_145459 | MYD88 | 0.008484 | -0.99152 |
| NR_145459 | SAMD13 | 0.006112 | 0.993888 |
| NR_145459 | PPP5C | 0.008134 | 0.991866 |
| NR_145459 | ZDHHC16 | 0.00625 | -0.99375 |
| NR_145459 | MACROD2 | 0.00728 | 0.99272 |
| NR_145459 | CENPO | 0.007331 | 0.992669 |
| NR_145459 | CHST12 | 0.004011 | 0.995989 |
| NR_145459 | HIPK2 | 0.005244 | -0.99476 |
| NR_145459 | PCDHB8 | 0.004011 | 0.995989 |
| NR_145459 | CATG00000080699.1 | 8.43E-04 | 0.999157 |
| NR_145459 | TCOF1 | 0.007129 | 0.992871 |
| NR_145459 | SLIRP | 0.009794 | 0.990206 |
| NR_145459 | MYD88 | 0.008484 | -0.99152 |
| NR_145459 | SAMD13 | 0.006112 | 0.993888 |
| NR_145459 | PPP5C | 0.008134 | 0.991866 |
| NR_145459 | ZDHHC16 | 0.00625 | -0.99375 |
| NR_145459 | MACROD2 | 0.00728 | 0.99272 |
| NR_145459 | CENPO | 0.007331 | 0.992669 |
| NR_145459 | CHST12 | 0.004011 | 0.995989 |
| NR_145459 | HIPK2 | 0.005244 | -0.99476 |
| NR_145459 | PCDHB8 | 0.004011 | 0.995989 |
| NR_145459 | CATG00000080699.1 | 8.43E-04 | 0.999157 |
| NR_145459 | TCOF1 | 0.007129 | 0.992871 |
| NR_145459 | SLIRP | 0.009794 | 0.990206 |
| NR_145459 | MYD88 | 0.008484 | -0.99152 |
| NR_145459 | SAMD13 | 0.006112 | 0.993888 |
| NR_145459 | PPP5C | 0.008134 | 0.991866 |
| NR_145459 | ZDHHC16 | 0.00625 | -0.99375 |
| NR_145459 | MACROD2 | 0.00728 | 0.99272 |
| NR_145459 | CENPO | 0.007331 | 0.992669 |
| NR_145459 | CHST12 | 0.004011 | 0.995989 |
| NR_145459 | HIPK2 | 0.005244 | -0.99476 |
| NR_145459 | PCDHB8 | 0.004011 | 0.995989 |
| ENST00000555769 | SPTY2D1OS | 6.39E-04 | 0.999361 |
| ENST00000555769 | KYAT1 | 0.004158 | 0.995842 |
| ENST00000555769 | CXCL17 | 0.007555 | 0.992445 |
| ENST00000555769 | LYL1 | 0.006152 | 0.993848 |
| ENST00000555769 | STEAP3 | 2.57E-04 | 0.999743 |
| ENST00000555769 | SLC13A4 | 0.009709 | 0.990291 |
| ENST00000555769 | STAR | 0.008909 | 0.991091 |
| ENST00000555769 | HIC1 | 0.009125 | 0.990875 |
| ENST00000555769 | TFF2 | 0.005017 | 0.994983 |
| ENST00000555769 | ORC3 | 0.001855 | 0.998145 |
| ENST00000555769 | UGT2B28 | 0.001931 | 0.998069 |
| ENST00000555769 | AVPI1 | 0.009985 | 0.990015 |
| ENST00000555769 | ABL1 | 0.005706 | -0.99429 |
| ENST00000555769 | SPTY2D1OS | 6.39E-04 | 0.999361 |
| ENST00000555769 | KYAT1 | 0.004158 | 0.995842 |
| ENST00000555769 | CXCL17 | 0.007555 | 0.992445 |
| ENST00000555769 | LYL1 | 0.006152 | 0.993848 |
| ENST00000555769 | STEAP3 | 2.57E-04 | 0.999743 |
| ENST00000555769 | SLC13A4 | 0.009709 | 0.990291 |
| ENST00000555769 | STAR | 0.008909 | 0.991091 |
| ENST00000555769 | HIC1 | 0.009125 | 0.990875 |
| ENST00000555769 | TFF2 | 0.005017 | 0.994983 |
| ENST00000555769 | ORC3 | 0.001855 | 0.998145 |
| ENST00000555769 | UGT2B28 | 0.001931 | 0.998069 |
| ENST00000555769 | AVPI1 | 0.009985 | 0.990015 |
| ENST00000555769 | ABL1 | 0.005706 | -0.99429 |
| ENST00000420253 | AHNAK2 | 0.007278 | -0.99272 |
| ENST00000420253 | C16orf45 | 0.008716 | -0.99128 |
| ENST00000420253 | NCCRP1 | 0.003479 | -0.99652 |
| ENST00000420253 | C20orf203 | 0.005388 | -0.99461 |
| ENST00000420253 | DUSP15 | 0.00675 | -0.99325 |
| ENST00000420253 | HOMER3 | 0.007161 | 0.992839 |
| ENST00000420253 | AASDHPPT | 0.004856 | -0.99514 |
| ENST00000420253 | ARPC4-TTLL3 | 7.41E-04 | 0.999259 |
| ENST00000420253 | MAMLD1 | 0.002325 | 0.997675 |
| ENST00000420253 | ZNF214 | 0.007028 | 0.992972 |
| ENST00000420253 | OR5D18 | 0.006732 | 0.993268 |
| ENST00000467155 | GNL1 | 0.004923 | -0.99508 |
| ENST00000467155 | DEAF1 | 0.005318 | 0.994682 |
| ENST00000467155 | VRK3 | 0.002576 | 0.997424 |
| ENST00000467155 | BLCAP | 0.002513 | 0.997487 |
| ENST00000467155 | FANCD2OS | 0.002629 | 0.997371 |
| ENST00000467155 | CATG00000023328.1 | 5.43E-04 | -0.99946 |
| ENST00000467155 | EFEMP2 | 0.006874 | -0.99313 |
| ENST00000467155 | ACSL6 | 0.00369 | 0.99631 |
| ENST00000467155 | OR4X1 | 0.001976 | 0.998024 |
| ENST00000467155 | MAP3K5 | 0.003762 | 0.996238 |
| ENST00000467155 | HLA-F | 0.005682 | 0.994318 |
| ENST00000467155 | CATG00000024701.1 | 0.001208 | 0.998792 |
| ENST00000467155 | GNL1 | 0.004923 | -0.99508 |
| ENST00000467155 | DEAF1 | 0.005318 | 0.994682 |
| ENST00000467155 | VRK3 | 0.002576 | 0.997424 |
| ENST00000467155 | BLCAP | 0.002513 | 0.997487 |
| ENST00000467155 | FANCD2OS | 0.002629 | 0.997371 |
| ENST00000467155 | CATG00000023328.1 | 5.43E-04 | -0.99946 |
| ENST00000467155 | EFEMP2 | 0.006874 | -0.99313 |
| ENST00000467155 | ACSL6 | 0.00369 | 0.99631 |
| ENST00000467155 | OR4X1 | 0.001976 | 0.998024 |
| ENST00000467155 | MAP3K5 | 0.003762 | 0.996238 |
| ENST00000467155 | HLA-F | 0.005682 | 0.994318 |
| ENST00000467155 | CATG00000024701.1 | 0.001208 | 0.998792 |
| ENST00000467155 | GNL1 | 0.004923 | -0.99508 |
| ENST00000467155 | DEAF1 | 0.005318 | 0.994682 |
| ENST00000467155 | VRK3 | 0.002576 | 0.997424 |
| ENST00000467155 | BLCAP | 0.002513 | 0.997487 |
| ENST00000467155 | FANCD2OS | 0.002629 | 0.997371 |
| ENST00000467155 | CATG00000023328.1 | 5.43E-04 | -0.99946 |
| ENST00000467155 | EFEMP2 | 0.006874 | -0.99313 |
| ENST00000467155 | ACSL6 | 0.00369 | 0.99631 |
| ENST00000467155 | OR4X1 | 0.001976 | 0.998024 |
| ENST00000467155 | MAP3K5 | 0.003762 | 0.996238 |
| ENST00000467155 | HLA-F | 0.005682 | 0.994318 |
| ENST00000467155 | CATG00000024701.1 | 0.001208 | 0.998792 |
| NR_045020 | ZFAND5 | 0.003575 | 0.996425 |
| NR_045020 | RTN1 | 0.006185 | -0.99382 |
| NR_045020 | NAGLU | 0.001738 | 0.998262 |
| NR_045020 | TP53I13 | 0.008995 | 0.991005 |
| NR_045020 | KRBA2 | 0.001471 | 0.998529 |
| NR_045020 | CLK3 | 0.005915 | 0.994085 |
| NR_045020 | AP3M2 | 0.003556 | -0.99644 |
| NR_045020 | CREBRF | 0.008145 | -0.99185 |
| NR_045020 | CATG00000054083.1 | 0.009915 | -0.99009 |
| NR_045020 | LCE2A | 0.009325 | 0.990675 |
| NR_045020 | PPTC7 | 0.008509 | -0.99149 |
| NR_045020 | CIB1 | 0.005906 | -0.99409 |
| ENST00000412178 | AHNAK2 | 0.009183 | -0.99082 |
| ENST00000412178 | HOMER3 | 0.009592 | 0.990408 |
| ENST00000412178 | SIRT6 | 0.003542 | -0.99646 |
| ENST00000412178 | AHNAK2 | 0.009183 | -0.99082 |
| ENST00000412178 | HOMER3 | 0.009592 | 0.990408 |
| ENST00000412178 | SIRT6 | 0.003542 | -0.99646 |
| ENST00000412178 | AHNAK2 | 0.009183 | -0.99082 |
| ENST00000412178 | HOMER3 | 0.009592 | 0.990408 |
| ENST00000412178 | SIRT6 | 0.003542 | -0.99646 |
| ENST00000412178 | AHNAK2 | 0.009183 | -0.99082 |
| ENST00000412178 | HOMER3 | 0.009592 | 0.990408 |
| ENST00000412178 | SIRT6 | 0.003542 | -0.99646 |
| NR_103476 | INTS2 | 0.009204 | 0.990796 |
| NR_103476 | SYBU | 3.15E-05 | -0.99997 |
| NR_103476 | EXOC2 | 0.001331 | 0.998669 |
| NR_103476 | GLIPR1L2 | 0.005962 | 0.994038 |
| NR_103476 | SH3D21 | 0.004794 | -0.99521 |
| NR_103476 | IKBKE | 0.005792 | 0.994208 |
| NR_103476 | KIF21B | 4.17E-04 | -0.99958 |
| NR_103476 | CATG00000074344.1 | 0.007412 | -0.99259 |
| NR_103476 | KCNK16 | 0.006079 | -0.99392 |
| NR_103476 | FANCE | 0.004176 | -0.99582 |
| NR_103476 | CLRN1 | 0.001171 | -0.99883 |
| uc001vdn.1 | PLEKHG2 | 0.009685 | 0.990315 |
| uc001vdn.1 | MAPKBP1 | 0.009858 | 0.990142 |
| uc001vdn.1 | CATG00000026669.1 | 0.001072 | -0.99893 |
| uc001vdn.1 | CARNS1 | 7.08E-04 | -0.99929 |
| ENST00000499137 | INTS2 | 0.002545 | -0.99745 |
| ENST00000499137 | SYBU | 0.009271 | 0.990729 |
| ENST00000499137 | RFC2 | 1.08E-04 | -0.99989 |
| ENST00000499137 | CNEP1R1 | 0.008271 | -0.99173 |
| ENST00000499137 | CDIP1 | 0.008841 | -0.99116 |
| ENST00000499137 | SH3D21 | 8.49E-04 | 0.999151 |
| ENST00000499137 | TP73 | 0.001341 | 0.998659 |
| ENST00000499137 | ARHGEF26 | 0.006743 | 0.993257 |
| ENST00000499137 | PHKA1 | 0.007344 | 0.992656 |
| ENST00000499137 | CATG00000074344.1 | 0.004021 | 0.995979 |
| ENST00000499137 | CATG00000026557.1 | 0.002568 | 0.997432 |
| ENST00000499137 | CLRN1 | 0.004148 | 0.995852 |
| ENST00000499137 | DOCK1 | 0.0011 | 0.9989 |
| ENST00000499137 | DEFA6 | 0.008122 | 0.991878 |
| ENST00000499137 | CATG00000027020.1 | 0.008039 | 0.991961 |
| ENST00000458653 | AHNAK2 | 0.003238 | 0.996762 |
| ENST00000458653 | CHSY3 | 0.008125 | 0.991875 |
| ENST00000458653 | HOMER3 | 0.003496 | -0.9965 |
| ENST00000458653 | ARPC4-TTLL3 | 0.006902 | -0.9931 |
| ENST00000458653 | CATG00000113928.1 | 0.006062 | -0.99394 |
| ENST00000458653 | MMP24 | 0.005213 | -0.99479 |
| ENST00000458653 | ZNF624 | 0.005037 | 0.994963 |
| ENST00000586544 | INPP5J | 0.007937 | 0.992063 |
| ENST00000586544 | RFC2 | 0.005246 | -0.99475 |
| ENST00000586544 | CATG00000101330.1 | 0.004874 | -0.99513 |
| ENST00000586544 | CNEP1R1 | 2.95E-04 | -0.9997 |
| ENST00000586544 | FRG1 | 0.009429 | -0.99057 |
| ENST00000586544 | SH3D21 | 0.009683 | 0.990317 |
| ENST00000586544 | TP73 | 0.00629 | 0.99371 |
| ENST00000586544 | ARHGEF26 | 0.004375 | 0.995625 |
| ENST00000586544 | PHKA1 | 0.001199 | 0.998801 |
| ENST00000586544 | CATG00000026557.1 | 0.002943 | 0.997057 |
| ENST00000586544 | COA1 | 0.004229 | -0.99577 |
| ENST00000586544 | PSME1 | 0.00777 | -0.99223 |
| ENST00000586544 | DOCK1 | 0.00237 | 0.99763 |
| ENST00000586544 | BMPER | 0.006454 | 0.993546 |
| ENST00000586544 | INPP5J | 0.007937 | 0.992063 |
| ENST00000586544 | RFC2 | 0.005246 | -0.99475 |
| ENST00000586544 | CATG00000101330.1 | 0.004874 | -0.99513 |
| ENST00000586544 | CNEP1R1 | 2.95E-04 | -0.9997 |
| ENST00000586544 | FRG1 | 0.009429 | -0.99057 |
| ENST00000586544 | SH3D21 | 0.009683 | 0.990317 |
| ENST00000586544 | TP73 | 0.00629 | 0.99371 |
| ENST00000586544 | ARHGEF26 | 0.004375 | 0.995625 |
| ENST00000586544 | PHKA1 | 0.001199 | 0.998801 |
| ENST00000586544 | CATG00000026557.1 | 0.002943 | 0.997057 |
| ENST00000586544 | COA1 | 0.004229 | -0.99577 |
| ENST00000586544 | PSME1 | 0.00777 | -0.99223 |
| ENST00000586544 | DOCK1 | 0.00237 | 0.99763 |
| ENST00000586544 | BMPER | 0.006454 | 0.993546 |
| ENST00000586544 | INPP5J | 0.007937 | 0.992063 |
| ENST00000586544 | RFC2 | 0.005246 | -0.99475 |
| ENST00000586544 | CATG00000101330.1 | 0.004874 | -0.99513 |
| ENST00000586544 | CNEP1R1 | 2.95E-04 | -0.9997 |
| ENST00000586544 | FRG1 | 0.009429 | -0.99057 |
| ENST00000586544 | SH3D21 | 0.009683 | 0.990317 |
| ENST00000586544 | TP73 | 0.00629 | 0.99371 |
| ENST00000586544 | ARHGEF26 | 0.004375 | 0.995625 |
| ENST00000586544 | PHKA1 | 0.001199 | 0.998801 |
| ENST00000586544 | CATG00000026557.1 | 0.002943 | 0.997057 |
| ENST00000586544 | COA1 | 0.004229 | -0.99577 |
| ENST00000586544 | PSME1 | 0.00777 | -0.99223 |
| ENST00000586544 | DOCK1 | 0.00237 | 0.99763 |
| ENST00000586544 | BMPER | 0.006454 | 0.993546 |
| ENST00000586544 | INPP5J | 0.007937 | 0.992063 |
| ENST00000586544 | RFC2 | 0.005246 | -0.99475 |
| ENST00000586544 | CATG00000101330.1 | 0.004874 | -0.99513 |
| ENST00000586544 | CNEP1R1 | 2.95E-04 | -0.9997 |
| ENST00000586544 | FRG1 | 0.009429 | -0.99057 |
| ENST00000586544 | SH3D21 | 0.009683 | 0.990317 |
| ENST00000586544 | TP73 | 0.00629 | 0.99371 |
| ENST00000586544 | ARHGEF26 | 0.004375 | 0.995625 |
| ENST00000586544 | PHKA1 | 0.001199 | 0.998801 |
| ENST00000586544 | CATG00000026557.1 | 0.002943 | 0.997057 |
| ENST00000586544 | COA1 | 0.004229 | -0.99577 |
| ENST00000586544 | PSME1 | 0.00777 | -0.99223 |
| ENST00000586544 | DOCK1 | 0.00237 | 0.99763 |
| ENST00000586544 | BMPER | 0.006454 | 0.993546 |
| ENST00000418747 | SPTY2D1OS | 0.004966 | 0.995034 |
| ENST00000418747 | KYAT1 | 0.009283 | 0.990717 |
| ENST00000418747 | LYL1 | 0.007531 | 0.992469 |
| ENST00000418747 | STEAP3 | 0.001494 | 0.998506 |
| ENST00000418747 | SLC13A4 | 0.005045 | 0.994955 |
| ENST00000418747 | TFF2 | 0.003456 | 0.996544 |
| ENST00000418747 | ORC3 | 0.003862 | 0.996138 |
| ENST00000418747 | UGT2B28 | 0.001146 | 0.998854 |
| ENST00000531077 | EPB41L1 | 0.007594 | 0.992406 |
| ENST00000531077 | INSC | 0.00687 | 0.99313 |
| ENST00000531077 | SLC7A4 | 0.003409 | 0.996591 |
| ENST00000531077 | GPR161 | 0.008336 | 0.991664 |
| ENST00000531077 | EIF1AD | 0.005098 | 0.994902 |
| ENST00000531077 | CATG00000047316.1 | 0.009737 | 0.990263 |
| ENST00000531077 | SAMD1 | 3.16E-04 | -0.99968 |
| ENST00000531077 | CAPRIN2 | 0.007298 | 0.992702 |
| ENST00000531077 | CATG00000089121.1 | 0.008827 | -0.99117 |
| ENST00000531077 | ZMYND15 | 0.002933 | 0.997067 |
| ENST00000531077 | CPOX | 0.005126 | 0.994874 |
| ENST00000531077 | CATG00000057824.1 | 0.004615 | 0.995385 |
| ENST00000531077 | IQCF5 | 4.42E-04 | 0.999558 |
| ENST00000531077 | KLF6 | 0.009915 | -0.99008 |
| ENST00000531077 | DNAL4 | 0.003308 | -0.99669 |
| ENST00000531077 | FAM53B | 2.33E-04 | -0.99977 |
| ENST00000531077 | LDHB | 0.003463 | -0.99654 |
| ENST00000531077 | WAPL | 0.009441 | -0.99056 |
| ENST00000531077 | AL627171.2 | 0.003239 | -0.99676 |
| NR_148338 | ARHGAP8 | 0.001943 | -0.99806 |
| NR_148338 | PLA2G15 | 2.14E-04 | -0.99979 |
| NR_148338 | RIOX2 | 0.003789 | -0.99621 |
| NR_148338 | ASGR2 | 0.004646 | -0.99535 |
| NR_148338 | OR8G1 | 0.006802 | -0.9932 |
| ENST00000500118 | BARHL1 | 0.008848 | 0.991152 |
| ENST00000500118 | B4GALNT1 | 0.001089 | 0.998911 |
| ENST00000500118 | S1PR5 | 0.002362 | 0.997638 |
| ENST00000500118 | RP1L1 | 0.001353 | 0.998647 |
| ENST00000500118 | DEFA1 | 0.003431 | -0.99657 |
| ENST00000500118 | PLEKHG3 | 7.08E-04 | 0.999292 |
| ENST00000500118 | DEFA4 | 0.007643 | -0.99236 |
| ENST00000482358 | NOC4L | 0.007912 | 0.992088 |
| ENST00000482358 | CATG00000080699.1 | 0.00291 | 0.99709 |
| ENST00000482358 | ZDHHC16 | 0.001859 | -0.99814 |
| ENST00000482358 | CDKL2 | 0.006639 | 0.993361 |
| ENST00000482358 | MACROD2 | 0.004335 | 0.995665 |
| ENST00000482358 | CHST12 | 0.00136 | 0.99864 |
| ENST00000482358 | HIPK2 | 0.002102 | -0.9979 |
| ENST00000482358 | PCDHB8 | 0.00136 | 0.99864 |
| ENST00000446201 | PTPN6 | 6.89E-04 | -0.99931 |
| ENST00000446201 | DNAJA4 | 0.009218 | 0.990782 |
| ENST00000446201 | PXDC1 | 0.008275 | 0.991725 |
| ENST00000446201 | NOL9 | 0.003495 | -0.9965 |
| ENST00000446201 | SPATA13 | 0.007395 | 0.992605 |
| ENST00000446201 | SPSB2 | 0.002823 | 0.997177 |
| ENST00000446201 | SIPA1L2 | 0.005888 | 0.994112 |
| NR_105008 | EGF | 0.001574 | 0.998426 |
| NR_105008 | CATG00000099282.1 | 0.001729 | -0.99827 |
| NR_105008 | ASB14 | 0.008914 | -0.99109 |
| NR_105008 | RHD | 0.005272 | 0.994728 |
| NR_105008 | CATG00000117842.1 | 0.008271 | 0.991729 |
| NR_105008 | C16orf86 | 0.007948 | -0.99205 |
| ENST00000430128 | INPP5J | 0.004216 | 0.995784 |
| ENST00000430128 | RFC2 | 0.007773 | -0.99223 |
| ENST00000430128 | RTN1 | 0.005747 | -0.99425 |
| ENST00000430128 | EXOC4 | 0.005721 | 0.994279 |
| ENST00000430128 | CNEP1R1 | 0.007097 | -0.9929 |
| ENST00000430128 | DBI | 0.003666 | -0.99633 |
| ENST00000430128 | TP73 | 0.004119 | 0.995881 |
| ENST00000430128 | AP3M2 | 0.00908 | -0.99092 |
| ENST00000430128 | NEMP2 | 0.009005 | 0.990995 |
| ENST00000430128 | DOCK1 | 0.009678 | 0.990322 |
| ENST00000430128 | BMPER | 0.004574 | 0.995426 |
| ENST00000430128 | DEFA6 | 0.00209 | 0.99791 |
| ENST00000430128 | CATG00000027020.1 | 0.008043 | 0.991957 |
| NR_135324 | C16orf45 | 0.008475 | 0.991525 |
| NR_135324 | PLAGL1 | 0.001237 | 0.998763 |
| NR_135324 | TNK2 | 0.009283 | 0.990717 |
| NR_135324 | RHBDD3 | 0.001477 | 0.998523 |
| NR_135324 | AIFM3 | 0.007239 | 0.992761 |
| NR_135324 | VGLL3 | 0.006886 | -0.99311 |
| NR_135324 | MMP2 | 0.00261 | -0.99739 |
| NR_135324 | OCSTAMP | 0.004607 | -0.99539 |
| NR_135324 | IL1R2 | 0.003341 | -0.99666 |
| NR_135324 | CROT | 0.004893 | 0.995107 |
| NR_104414 | DMAC2 | 0.001521 | 0.998479 |
| NR_104414 | 11-Mar | 0.008606 | -0.99139 |
| NR_104414 | CPLX1 | 0.004675 | -0.99532 |
| NR_104414 | PELP1 | 0.007667 | 0.992333 |
| NR_104414 | GMEB2 | 0.007052 | -0.99295 |
| NR_104414 | ZNF579 | 0.002933 | 0.997067 |
| NR_104414 | OR13A1 | 0.001359 | -0.99864 |
| NR_104414 | ANKHD1 | 0.00731 | -0.99269 |
| NR_104414 | SMUG1 | 0.002944 | -0.99706 |
| NR_104414 | TRIM47 | 0.008855 | 0.991145 |
| NR_104414 | KNCN | 0.008023 | 0.991977 |
| NR_104414 | DCUN1D1 | 2.00E-04 | -0.9998 |
| NR_104414 | C3orf84 | 0.0045 | 0.9955 |
| NR_104414 | AC109583.1 | 0.005788 | 0.994212 |
| NR_104414 | CAMK2N2 | 0.007578 | 0.992422 |
| ENST00000480632 | DNAJC22 | 0.002869 | -0.99713 |
| ENST00000480632 | MYT1L | 0.007301 | -0.9927 |
| ENST00000480632 | CATG00000074949.1 | 0.003653 | -0.99635 |
| ENST00000480632 | ASGR2 | 0.009209 | -0.99079 |
| ENST00000480632 | OR8G1 | 0.001448 | -0.99855 |
| ENST00000480632 | KCNE4 | 0.001016 | 0.998984 |
| ENST00000417473 | AHNAK2 | 0.008988 | 0.991012 |
| ENST00000417473 | C16orf45 | 0.001985 | 0.998015 |
| ENST00000417473 | NCCRP1 | 6.27E-04 | 0.999373 |
| ENST00000417473 | HOMER3 | 0.008621 | -0.99138 |
| ENST00000417473 | MYO7A | 0.003527 | -0.99647 |
| ENST00000417473 | ARPC4-TTLL3 | 0.003876 | -0.99612 |
| ENST00000417473 | MAMLD1 | 4.59E-04 | -0.99954 |
| ENST00000417473 | ABHD5 | 0.003291 | -0.99671 |
| ENST00000417473 | ZNF214 | 0.003462 | -0.99654 |
| ENST00000417473 | OCSTAMP | 0.003883 | -0.99612 |
| ENST00000417473 | SP140 | 0.003264 | 0.996736 |
| ENST00000417473 | CROT | 0.009812 | 0.990188 |
| ENST00000623943 | YWHAB | 0.003718 | 0.996282 |
| ENST00000623943 | CACHD1 | 0.001688 | 0.998312 |
| ENST00000623943 | INSC | 0.006984 | 0.993016 |
| ENST00000623943 | GPR161 | 0.002488 | 0.997512 |
| ENST00000623943 | EIF1AD | 0.002386 | 0.997614 |
| ENST00000623943 | CAPRIN2 | 0.001691 | 0.998309 |
| ENST00000623943 | CLEC5A | 0.007725 | 0.992275 |
| ENST00000623943 | CATG00000089121.1 | 4.23E-04 | -0.99958 |
| ENST00000623943 | OAS1 | 0.005089 | 0.994911 |
| ENST00000623943 | RBM7 | 0.006029 | 0.993971 |
| ENST00000623943 | CPOX | 0.006006 | 0.993994 |
| ENST00000623943 | ERFE | 0.003096 | -0.9969 |
| ENST00000623943 | SNRPD2 | 0.009385 | 0.990615 |
| ENST00000623943 | IQCF5 | 0.007884 | 0.992116 |
| ENST00000623943 | CSNK1A1 | 0.006915 | -0.99308 |
| ENST00000623943 | LIM2 | 0.005732 | 0.994268 |
| ENST00000623943 | INO80B | 0.009586 | 0.990414 |
| ENST00000623943 | RPL17 | 0.005667 | -0.99433 |
| ENST00000623943 | PLCL1 | 0.001514 | -0.99849 |
| ENST00000623943 | AL627171.2 | 0.002849 | -0.99715 |
| ENST00000623943 | TNFRSF13B | 0.003835 | -0.99616 |
| ENST00000623943 | GABPA | 0.008054 | 0.991946 |
| NR_103825 | ZNF490 | 7.22E-04 | -0.99928 |
| NR_103825 | HEYL | 0.009225 | 0.990775 |
| NR_103825 | PNCK | 0.006645 | -0.99335 |
| NR_103825 | SCYGR6 | 0.00135 | -0.99865 |
| NR_103825 | BRSK2 | 0.003277 | -0.99672 |
| NR_103825 | LCE4A | 0.001825 | 0.998175 |
| NR_103825 | SCRG1 | 0.00312 | 0.99688 |
| NR_103825 | ZFYVE26 | 0.005158 | 0.994842 |
| ENST00000434411 | TOB1 | 0.00308 | -0.99692 |
| ENST00000434411 | MED20 | 0.006332 | 0.993668 |
| ENST00000434411 | CFLAR | 0.009851 | -0.99015 |
| ENST00000434411 | ACO1 | 0.007474 | 0.992526 |
| ENST00000434411 | C17orf47 | 0.006104 | 0.993896 |
| ENST00000434411 | TSFM | 0.009775 | -0.99023 |
| ENST00000595826 | CATG00000021869.1 | 0.008525 | -0.99148 |
| ENST00000595826 | KCNJ11 | 0.00737 | -0.99263 |
| ENST00000595826 | TSPAN32 | 0.00184 | 0.99816 |
| ENST00000595826 | SLPI | 0.008986 | -0.99101 |
| ENST00000595826 | METTL3 | 0.003288 | -0.99671 |
| ENST00000595826 | C1QTNF9B | 0.003235 | -0.99676 |
| ENST00000595826 | UPP2 | 0.002928 | -0.99707 |
| ENST00000481950 | ECSIT | 0.007935 | 0.992065 |
| ENST00000481950 | CNEP1R1 | 0.009346 | -0.99065 |
| ENST00000481950 | AC092073.1 | 0.00639 | 0.99361 |
| ENST00000481950 | FRG1 | 0.004015 | -0.99599 |
| ENST00000481950 | TGFBR3 | 0.00306 | 0.99694 |
| ENST00000481950 | SLC22A6 | 0.002241 | -0.99776 |
| ENST00000481950 | COA1 | 0.001576 | -0.99842 |
| ENST00000481950 | COA5 | 0.001249 | -0.99875 |
| ENST00000481950 | RRBP1 | 0.006464 | -0.99354 |
| ENST00000481950 | MORN3 | 0.004251 | -0.99575 |
| ENST00000481950 | VPS13A | 0.003124 | 0.996876 |
| ENST00000481950 | CAPN15 | 0.008942 | 0.991058 |
| ENST00000402631 | CATG00000021869.1 | 0.002375 | 0.997625 |
| ENST00000402631 | PLEKHG2 | 0.007536 | -0.99246 |
| ENST00000402631 | KCNJ11 | 0.001867 | 0.998133 |
| ENST00000402631 | TSPAN32 | 0.006777 | -0.99322 |
| ENST00000402631 | NHEJ1 | 0.007134 | -0.99287 |
| ENST00000402631 | IQANK1 | 0.006551 | 0.993449 |
| ENST00000402631 | SLPI | 0.007109 | 0.992891 |
| ENST00000402631 | METTL3 | 0.004453 | 0.995547 |
| ENST00000402631 | C1QTNF9B | 0.008689 | 0.991311 |
| ENST00000402631 | KLRG2 | 0.00972 | -0.99028 |
| ENST00000402631 | UPP2 | 0.008759 | 0.991241 |
| ENST00000457315 | CPSF3 | 0.007105 | 0.992895 |
| ENST00000457315 | C11orf71 | 0.008529 | 0.991471 |
| ENST00000457315 | MYLK3 | 0.003909 | 0.996091 |
| ENST00000457315 | FBLN2 | 0.001244 | 0.998756 |
| ENST00000457315 | CYP21A2 | 0.006155 | -0.99385 |
| ENST00000457315 | SLC39A12 | 0.006974 | -0.99303 |
| ENST00000457315 | SYNGR3 | 0.004061 | -0.99594 |
| ENST00000457315 | EDDM3A | 0.002267 | -0.99773 |
| ENST00000517697 | ZNF490 | 0.004163 | -0.99584 |
| ENST00000517697 | HEYL | 0.002581 | 0.997419 |
| ENST00000517697 | TMEM236 | 0.004236 | -0.99576 |
| ENST00000517697 | SCYGR6 | 0.003871 | -0.99613 |
| ENST00000517697 | BRSK2 | 0.00102 | -0.99898 |
| ENST00000517697 | LCE4A | 0.00433 | 0.99567 |
| ENST00000517697 | LYPD8 | 0.00545 | 0.99455 |
| ENST00000517697 | SCRG1 | 7.60E-04 | 0.99924 |
| ENST00000517697 | ZFYVE26 | 0.005844 | 0.994156 |
| ENST00000447413 | MRPL24 | 0.005593 | 0.994407 |
| ENST00000447413 | BTBD2 | 0.004885 | -0.99512 |
| ENST00000447413 | UBB | 0.009843 | -0.99016 |
| ENST00000447413 | EXOC3L2 | 0.006955 | 0.993045 |
| ENST00000447413 | TMOD1 | 0.007247 | -0.99275 |
| ENST00000447413 | HMOX2 | 0.00105 | -0.99895 |
| ENST00000447413 | CCNK | 0.001932 | -0.99807 |
| ENST00000447413 | CATG00000039609.1 | 0.008088 | 0.991912 |
| ENST00000509886 | SLC13A4 | 0.00747 | 0.99253 |
| ENST00000509886 | CATG00000039284.1 | 0.002366 | -0.99763 |
| ENST00000509886 | ORM1 | 0.008188 | 0.991812 |
| NR_144339 | BTBD2 | 4.62E-04 | 0.999538 |
| NR_144339 | HDHD5 | 0.007106 | -0.99289 |
| NR_144339 | UBB | 0.005402 | 0.994598 |
| NR_144339 | EXOC3L2 | 0.00942 | -0.99058 |
| NR_144339 | POP1 | 0.005019 | 0.994981 |
| NR_144339 | EVC2 | 0.009099 | 0.990901 |
| NR_144339 | TMOD1 | 0.00261 | 0.99739 |
| NR_144339 | HMOX2 | 0.004099 | 0.995901 |
| NR_144339 | CCNK | 1.64E-04 | 0.999836 |
| NR_144339 | CATG00000039609.1 | 0.005879 | -0.99412 |
| NR_144339 | CATG00000020284.1 | 0.006273 | -0.99373 |
| ENST00000515194 | CATG00000003494.1 | 0.003834 | -0.99617 |
| ENST00000515194 | CACHD1 | 0.001953 | -0.99805 |
| ENST00000515194 | INSC | 0.005759 | -0.99424 |
| ENST00000515194 | PCDHGA3 | 0.008077 | -0.99192 |
| ENST00000515194 | CATG00000089121.1 | 0.006874 | 0.993126 |
| ENST00000515194 | OAS1 | 2.22E-04 | -0.99978 |
| ENST00000515194 | DCD | 0.00798 | -0.99202 |
| ENST00000515194 | CSNK1A1 | 6.95E-04 | 0.999305 |
| ENST00000515194 | ZNF587B | 0.004381 | 0.995619 |
| ENST00000515194 | INO80B | 0.003474 | -0.99653 |
| ENST00000515194 | RPL17 | 1.40E-04 | 0.99986 |
| ENST00000515194 | WAPL | 0.006918 | 0.993082 |
| ENST00000515194 | PLCL1 | 0.00302 | 0.99698 |
| ENST00000515194 | TNFRSF13B | 0.008911 | 0.991089 |
| ENST00000515194 | GABPA | 0.001744 | -0.99826 |
| NR_105000 | RPS7 | 0.00295 | 0.99705 |
| NR_105000 | 11-Mar | 0.008053 | -0.99195 |
| NR_105000 | ACOT12 | 7.26E-04 | 0.999274 |
| NR_105000 | PALM3 | 0.00964 | -0.99036 |
| NR_105000 | BCAS4 | 0.004146 | 0.995854 |
| NR_105000 | PROKR1 | 0.005528 | -0.99447 |
| NR_105000 | PELP1 | 0.003158 | 0.996842 |
| NR_105000 | OR8B4 | 0.002998 | -0.997 |
| NR_105000 | MYH4 | 1.20E-04 | 0.99988 |
| NR_105000 | CLEC4E | 0.002264 | 0.997736 |
| NR_105000 | KNCN | 0.007354 | 0.992646 |
| NR_105000 | FANCL | 0.00365 | 0.99635 |
| NR_105000 | CAMK2N2 | 0.00416 | 0.99584 |
| NR_120634 | AX748369 | 0.007864 | -0.99214 |
| NR_120634 | GPCPD1 | 0.001412 | 0.998588 |
| NR_120634 | STMND1 | 0.001603 | -0.9984 |
| NR_120634 | LYPD8 | 0.007883 | -0.99212 |
| NR_120634 | DRC1 | 0.004577 | -0.99542 |
| NR_120634 | FGD4 | 0.007067 | -0.99293 |
| NR_120634 | ZFYVE26 | 0.005528 | -0.99447 |
| ENST00000611052 | ZFAND5 | 5.90E-04 | -0.99941 |
| ENST00000611052 | LYL1 | 0.005844 | -0.99416 |
| ENST00000611052 | TP53I13 | 0.00113 | -0.99887 |
| ENST00000611052 | KRBA2 | 0.001581 | -0.99842 |
| ENST00000611052 | HIC1 | 0.003507 | -0.99649 |
| ENST00000611052 | TFF2 | 0.009016 | -0.99098 |
| ENST00000611052 | CREBRF | 0.002617 | 0.997383 |
| ENST00000611052 | CATG00000054083.1 | 0.003504 | 0.996496 |
| ENST00000611052 | ZNF664 | 0.002243 | 0.997757 |
| ENST00000611052 | LCE2A | 0.004684 | -0.99532 |
| ENST00000611052 | CIB1 | 0.009132 | 0.990868 |
| ENST00000505410 | RPS7 | 0.009135 | 0.990865 |
| ENST00000505410 | ACOT12 | 0.001094 | 0.998906 |
| ENST00000505410 | SELENOP | 0.009168 | -0.99083 |
| ENST00000505410 | BCAS4 | 0.004844 | 0.995156 |
| ENST00000505410 | PROKR1 | 0.001485 | -0.99851 |
| ENST00000505410 | PELP1 | 0.008678 | 0.991322 |
| ENST00000505410 | OR8B4 | 5.89E-04 | -0.99941 |
| ENST00000505410 | MYH4 | 9.24E-04 | 0.999076 |
| ENST00000505410 | CLEC4E | 0.003735 | 0.996265 |
| ENST00000505410 | FANCL | 7.23E-04 | 0.999277 |
| ENST00000505410 | CAMK2N2 | 0.009044 | 0.990956 |
| ENST00000505329 | BECN1 | 0.002963 | -0.99704 |
| ENST00000505329 | ACTL7B | 6.23E-04 | 0.999377 |
| ENST00000505329 | C15orf65 | 0.007953 | 0.992047 |
| ENST00000505329 | NDUFAF8 | 0.001524 | -0.99848 |
| ENST00000505329 | MED12L | 0.001893 | 0.998107 |
| ENST00000505329 | PMEPA1 | 0.005357 | 0.994643 |
| ENST00000505329 | FCRL1 | 0.009119 | 0.990881 |
| ENST00000562710 | INTS3 | 0.0054 | -0.9946 |
| ENST00000562710 | VPS41 | 0.003481 | -0.99652 |
| ENST00000562710 | CHIC2 | 0.007009 | 0.992991 |
| ENST00000562710 | TMEM43 | 8.36E-04 | 0.999164 |
| ENST00000563304 | TAS2R42 | 0.002607 | 0.997393 |
| ENST00000563304 | GRINA | 1.53E-04 | 0.999847 |
| ENST00000563304 | PSRC1 | 0.005585 | -0.99442 |
| ENST00000563304 | PLIN4 | 0.002519 | 0.997481 |
| ENST00000563304 | LILRB1 | 1.12E-04 | -0.99989 |
| ENST00000563304 | RASL10A | 6.73E-04 | -0.99933 |
| ENST00000563304 | LTA4H | 0.008588 | -0.99141 |
| ENST00000563304 | CXXC1 | 0.005848 | -0.99415 |
| ENST00000563304 | HLF | 0.009075 | 0.990925 |
| ENST00000563304 | NFX1 | 3.28E-04 | -0.99967 |
| ENST00000563304 | CATG00000012021.1 | 0.007363 | -0.99264 |
| ENST00000563304 | PEA15 | 2.59E-04 | 0.999741 |
| ENST00000563304 | ESRRB | 0.00486 | 0.99514 |
| ENST00000563304 | IFT122 | 0.001468 | 0.998532 |
| ENST00000563304 | SLC12A3 | 0.005879 | -0.99412 |
| ENST00000563304 | SIGLEC7 | 0.004598 | 0.995402 |
| ENST00000563304 | WNT8B | 3.51E-04 | 0.999649 |
| ENST00000563304 | UNC5C | 4.56E-04 | -0.99954 |
| ENST00000563304 | AGO2 | 0.004517 | 0.995483 |
| ENST00000563304 | CATG00000087047.1 | 0.007334 | 0.992666 |
| ENST00000563304 | FAM174A | 0.009655 | -0.99035 |
| ENST00000563304 | CFAP410 | 0.006008 | 0.993992 |
| ENST00000563304 | HPS1 | 4.25E-04 | 0.999575 |
| ENST00000563304 | CCDC174 | 0.00985 | -0.99015 |
| ENST00000563304 | FAM151A | 0.004288 | 0.995712 |
| ENST00000420403 | ACTL7B | 0.006918 | 0.993082 |
| ENST00000420403 | FBXL16 | 0.00659 | -0.99341 |
| ENST00000420403 | CCDC42 | 0.009807 | -0.99019 |
| ENST00000420403 | MED12L | 0.001791 | 0.998209 |
| ENST00000420403 | CEBPD | 0.008612 | 0.991388 |
| ENST00000617352 | FAM84A | 0.00744 | -0.99256 |
| ENST00000617352 | C20orf203 | 0.001952 | -0.99805 |
| ENST00000617352 | DUSP15 | 0.001253 | -0.99875 |
| ENST00000617352 | AASDHPPT | 2.15E-05 | -0.99998 |
| ENST00000617352 | ARPC4-TTLL3 | 0.007716 | 0.992284 |
| ENST00000617352 | G6PC3 | 0.004862 | 0.995138 |
| ENST00000617352 | RTN4 | 0.006557 | 0.993443 |
| ENST00000617352 | FAM110C | 0.005324 | 0.994676 |
| ENST00000617352 | ZNF624 | 0.0074 | -0.9926 |
| ENST00000617352 | OR5D18 | 4.48E-04 | 0.999552 |
| ENST00000493841 | FILIP1 | 0.00589 | -0.99411 |
| ENST00000493841 | CLEC1A | 0.007473 | -0.99253 |
| ENST00000493841 | HMGXB4 | 7.25E-04 | -0.99927 |
| ENST00000493841 | RPL11 | 8.72E-04 | -0.99913 |
| ENST00000493841 | PMPCA | 0.004317 | 0.995683 |
| ENST00000493841 | UNC45A | 0.003359 | 0.996641 |
| ENST00000493841 | CAPNS1 | 0.003635 | -0.99636 |
| ENST00000493841 | TUBGCP4 | 0.007248 | 0.992752 |
| ENST00000493841 | RACK1 | 0.005278 | 0.994722 |
| ENST00000493841 | TAF8 | 0.008949 | -0.99105 |
| ENST00000493841 | KRTAP4-7 | 0.005923 | 0.994077 |
| ENST00000493841 | CDKN3 | 4.01E-05 | -0.99996 |
| ENST00000493841 | RPUSD2 | 0.004484 | -0.99552 |
| ENST00000493841 | USP9X | 0.002577 | 0.997423 |
| ENST00000493841 | ZNF431 | 0.008521 | 0.991479 |
| ENST00000440729 | CATG00000021869.1 | 0.008601 | 0.991399 |
| ENST00000440729 | MAPKBP1 | 0.007823 | -0.99218 |
| ENST00000440729 | TREML2 | 0.001193 | -0.99881 |
| ENST00000440729 | AC093157.1 | 0.006439 | 0.993561 |
| ENST00000440729 | FRAT1 | 0.007475 | 0.992525 |
| ENST00000483316 | HIGD2A | 0.005679 | 0.994321 |
| ENST00000483316 | SLC2A4 | 0.00936 | -0.99064 |
| ENST00000483316 | SYNDIG1L | 3.46E-04 | 0.999654 |
| ENST00000483316 | HIRA | 0.009291 | 0.990709 |
| ENST00000483316 | CATG00000101330.1 | 0.00832 | 0.99168 |
| ENST00000483316 | AQP1 | 0.007611 | 0.992389 |
| ENST00000483316 | IL17RA | 0.003069 | -0.99693 |
| ENST00000483316 | KRTAP10-6 | 0.003508 | -0.99649 |
| ENST00000483316 | AP002990.1 | 0.003783 | 0.996217 |
| ENST00000483316 | AC092073.1 | 0.009073 | -0.99093 |
| ENST00000483316 | TGFBR3 | 0.009793 | -0.99021 |
| ENST00000483316 | PIKFYVE | 0.009443 | 0.990557 |
| ENST00000483316 | LYPD1 | 0.002436 | -0.99756 |
| ENST00000483316 | ELSPBP1 | 0.001653 | -0.99835 |
| ENST00000483316 | SLC6A5 | 1.95E-04 | -0.99981 |
| ENST00000483316 | RRBP1 | 0.009487 | 0.990513 |
| ENST00000483316 | PSME1 | 0.008501 | 0.991499 |
| ENST00000483316 | DOHH | 0.008612 | -0.99139 |
| ENST00000483316 | VPS13A | 0.004129 | -0.99587 |
| ENST00000537250 | PHF20 | 0.004341 | -0.99566 |
| ENST00000537250 | CDO1 | 0.006568 | -0.99343 |
| ENST00000537250 | PROKR1 | 0.006271 | -0.99373 |
| ENST00000537250 | TSC22D4 | 0.001797 | -0.9982 |
| ENST00000537250 | GRTP1 | 0.009852 | 0.990148 |
| ENST00000537250 | ABCA4 | 0.001248 | 0.998752 |
| ENST00000537250 | DEFB112 | 0.008833 | -0.99117 |
| ENST00000537250 | GPR153 | 0.006342 | 0.993658 |
| ENST00000537250 | CLEC4E | 0.005338 | 0.994662 |
| ENST00000537250 | KCTD18 | 0.003623 | 0.996377 |
| ENST00000537250 | ARG1 | 0.001748 | 0.998252 |
| ENST00000541320 | MMP1 | 0.005161 | -0.99484 |
| ENST00000541320 | TTC38 | 0.00437 | -0.99563 |
| ENST00000472821 | TAS2R42 | 0.005728 | -0.99427 |
| ENST00000472821 | GRINA | 0.001812 | -0.99819 |
| ENST00000472821 | HMGB2 | 0.006958 | 0.993042 |
| ENST00000472821 | PSRC1 | 0.008765 | 0.991235 |
| ENST00000472821 | PLIN4 | 2.54E-04 | -0.99975 |
| ENST00000472821 | LILRB1 | 0.002068 | 0.997932 |
| ENST00000472821 | RASL10A | 0.003818 | 0.996182 |
| ENST00000472821 | CXXC1 | 0.001637 | 0.998363 |
| ENST00000472821 | TBX3 | 0.007473 | -0.99253 |
| ENST00000472821 | KRT83 | 0.007091 | -0.99291 |
| ENST00000472821 | HLF | 0.009664 | -0.99034 |
| ENST00000472821 | TPTE | 0.009243 | -0.99076 |
| ENST00000472821 | NFX1 | 0.002891 | 0.997109 |
| ENST00000472821 | CATG00000012021.1 | 0.004199 | 0.995801 |
| ENST00000472821 | FYB2 | 0.005774 | -0.99423 |
| ENST00000472821 | PEA15 | 0.002689 | -0.99731 |
| ENST00000472821 | ESRRB | 0.001901 | -0.9981 |
| ENST00000472821 | IFT122 | 0.001103 | -0.9989 |
| ENST00000472821 | SLC12A3 | 0.007772 | 0.992228 |
| ENST00000472821 | SIGLEC7 | 0.003063 | -0.99694 |
| ENST00000472821 | WNT8B | 0.002008 | -0.99799 |
| ENST00000472821 | UNC5C | 0.002565 | 0.997435 |
| ENST00000472821 | AGO2 | 0.009129 | -0.99087 |
| ENST00000472821 | FAM174A | 0.007884 | 0.992116 |
| ENST00000472821 | CFAP410 | 0.007022 | -0.99298 |
| ENST00000472821 | HPS1 | 0.001118 | -0.99888 |
| ENST00000472821 | CCDC174 | 0.004084 | 0.995916 |
| ENST00000472821 | FAM151A | 0.001789 | -0.99821 |
| ENST00000494880 | LCK | 0.002397 | -0.9976 |
| ENST00000494880 | AGPS | 0.00369 | -0.99631 |
| ENST00000494880 | TEX13D | 0.002918 | 0.997082 |
| ENST00000494880 | IRX3 | 0.006118 | 0.993882 |
| ENST00000494880 | SNX16 | 0.002969 | -0.99703 |
| ENST00000494880 | SPATA13 | 0.007562 | 0.992438 |
| ENST00000494880 | BHLHB9 | 0.001741 | 0.998259 |
| ENST00000494880 | PLTP | 0.006682 | 0.993318 |
| ENST00000494880 | PLCH2 | 0.003594 | 0.996406 |
| ENST00000494880 | COBL | 0.009105 | 0.990895 |
| ENST00000494880 | ODF3B | 0.003979 | 0.996021 |
| ENST00000494880 | RRP9 | 0.001402 | 0.998598 |
| ENST00000494880 | CATG00000038465.1 | 0.006093 | 0.993907 |
| ENST00000494880 | SPSB2 | 0.009322 | 0.990678 |
| ENST00000494880 | SIPA1L2 | 0.009056 | 0.990944 |
| ENST00000452883 | INPP5J | 0.007765 | 0.992235 |
| ENST00000452883 | ECSIT | 0.006306 | 0.993694 |
| ENST00000452883 | SSBP2 | 0.009613 | 0.990387 |
| ENST00000452883 | CNEP1R1 | 0.007212 | -0.99279 |
| ENST00000452883 | FRG1 | 5.86E-04 | -0.99941 |
| ENST00000452883 | TGFBR3 | 0.008344 | 0.991656 |
| ENST00000452883 | SLC22A6 | 0.002957 | -0.99704 |
| ENST00000452883 | COA1 | 0.002396 | -0.9976 |
| ENST00000452883 | COA5 | 0.002989 | -0.99701 |
| ENST00000452883 | MORN3 | 0.009332 | -0.99067 |
| ENST00000452883 | BMPER | 0.006423 | 0.993577 |
| ENST00000452883 | VPS13A | 0.008979 | 0.991021 |
| ENST00000462085 | ECSIT | 0.006335 | 0.993665 |
| ENST00000462085 | SIRPG | 0.003153 | 0.996847 |
| ENST00000462085 | CHST8 | 0.007472 | 0.992528 |
| ENST00000462085 | AHSP | 0.003582 | 0.996418 |
| ENST00000462085 | AC092073.1 | 0.00186 | 0.99814 |
| ENST00000462085 | CATG00000022188.1 | 0.007591 | 0.992409 |
| ENST00000462085 | TGFBR3 | 0.001421 | 0.998579 |
| ENST00000462085 | GSG1L | 0.009849 | 0.990151 |
| ENST00000462085 | SLC22A6 | 0.002733 | -0.99727 |
| ENST00000462085 | SPATA31D1 | 0.004953 | 0.995047 |
| ENST00000462085 | CATG00000038058.1 | 0.004857 | 0.995143 |
| ENST00000462085 | CATG00000034210.1 | 0.003502 | 0.996498 |
| ENST00000462085 | COA5 | 0.002173 | -0.99783 |
| ENST00000462085 | RRBP1 | 0.001709 | -0.99829 |
| ENST00000462085 | ZC3H12D | 0.006433 | -0.99357 |
| ENST00000462085 | MORN3 | 3.61E-04 | -0.99964 |
| ENST00000462085 | VPS13A | 0.006199 | 0.993801 |
| ENST00000462085 | FGF9 | 0.001672 | -0.99833 |
| ENST00000462085 | CAPN15 | 3.69E-04 | 0.999631 |
| ENST00000508735 | NUP54 | 0.005671 | 0.994329 |
| ENST00000508735 | TDRD12 | 0.003914 | 0.996086 |
| ENST00000508735 | FTSJ1 | 0.005565 | 0.994435 |
| ENST00000508735 | ANXA3 | 0.005054 | 0.994946 |
| ENST00000508735 | CHIC2 | 0.003377 | -0.99662 |
| ENST00000508735 | RNF149 | 3.15E-04 | -0.99968 |
| ENST00000618234 | CCDC114 | 0.009057 | -0.99094 |
| ENST00000618234 | C1orf198 | 0.005357 | -0.99464 |
| ENST00000618234 | LHFPL6 | 0.004846 | 0.995154 |
| ENST00000618234 | BVES | 0.001624 | 0.998376 |
| ENST00000618234 | ARHGEF33 | 0.008973 | 0.991027 |
| ENST00000618234 | COMMD2 | 0.005545 | -0.99446 |
| ENST00000618234 | ANKS3 | 0.009442 | 0.990558 |
| ENST00000618234 | ASPRV1 | 0.007935 | -0.99206 |
| ENST00000524376 | AIFM3 | 0.006167 | 0.993833 |
| ENST00000524376 | ZNF77 | 0.00776 | 0.99224 |
| ENST00000433079 | ECSIT | 0.003217 | 0.996783 |
| ENST00000433079 | CATG00000056264.1 | 0.002723 | 0.997277 |
| ENST00000433079 | SIRPG | 0.002575 | 0.997425 |
| ENST00000433079 | CATG00000003494.1 | 0.005363 | 0.994637 |
| ENST00000433079 | YWHAB | 0.009146 | 0.990854 |
| ENST00000433079 | SSBP2 | 0.002596 | 0.997404 |
| ENST00000433079 | CACHD1 | 0.007056 | 0.992944 |
| ENST00000433079 | CHST8 | 9.98E-04 | 0.999002 |
| ENST00000433079 | CLEC4G | 0.005609 | 0.994391 |
| ENST00000433079 | TMC2 | 0.005244 | 0.994756 |
| ENST00000433079 | CATG00000022188.1 | 0.001107 | 0.998893 |
| ENST00000433079 | TPRX1 | 0.009409 | 0.990591 |
| ENST00000433079 | RBM7 | 0.003296 | 0.996704 |
| ENST00000433079 | SLC22A6 | 0.009071 | -0.99093 |
| ENST00000433079 | SNRPD2 | 0.009087 | 0.990913 |
| ENST00000433079 | SPATA31D1 | 0.003424 | 0.996576 |
| ENST00000433079 | CATG00000038058.1 | 0.001888 | 0.998112 |
| ENST00000433079 | ZC3H12D | 0.003579 | -0.99642 |
| ENST00000433079 | INO80B | 0.005486 | 0.994514 |
| ENST00000433079 | PLCL1 | 0.006264 | -0.99374 |
| ENST00000433079 | TNFRSF13B | 0.003748 | -0.99625 |
| ENST00000433079 | GABPA | 0.007999 | 0.992001 |
| ENST00000433079 | FGF9 | 0.004971 | -0.99503 |
| NR_110086 | SPANXN4 | 0.001381 | -0.99862 |
| NR_110086 | CD19 | 0.005495 | 0.994505 |
| NR_110086 | RELL1 | 5.97E-04 | 0.999403 |
| NR_110086 | ANKS3 | 0.006183 | 0.993817 |
| NR_110086 | ATXN7 | 0.004702 | 0.995298 |
| NR_110086 | ASPRV1 | 0.007178 | -0.99282 |
| ENST00000496267 | VPS41 | 0.006383 | 0.993617 |
| ENST00000496267 | ANXA3 | 0.009217 | 0.990783 |
| ENST00000496267 | CHIC2 | 9.86E-04 | -0.99901 |
| ENST00000496267 | TMEM43 | 0.003512 | -0.99649 |
| ENST00000496267 | RNF149 | 0.001459 | -0.99854 |
| ENST00000519714 | ZNF30 | 0.006407 | -0.99359 |
| ENST00000519714 | SZRD1 | 0.005512 | -0.99449 |
| ENST00000519714 | FAM84A | 0.004984 | -0.99502 |
| ENST00000519714 | CAPS | 0.008253 | -0.99175 |
| ENST00000519714 | GMPPA | 4.68E-05 | -0.99995 |
| ENST00000519714 | CATG00000053936.1 | 0.005274 | -0.99473 |
| ENST00000519714 | G6PC3 | 0.008129 | 0.991871 |
| ENST00000519714 | ARHGEF40 | 0.005258 | 0.994742 |
| ENST00000519714 | CATG00000107162.1 | 0.00209 | 0.99791 |
| ENST00000519714 | C16orf78 | 0.004058 | 0.995942 |
| ENST00000519714 | EFCAB8 | 0.003912 | 0.996088 |
| ENST00000519714 | MSI1 | 0.004868 | 0.995132 |
| NR_026563 | TAS2R42 | 0.005019 | 0.994981 |
| NR_026563 | B3GNT3 | 1.45E-04 | -0.99986 |
| NR_026563 | PPP3CC | 0.002597 | -0.9974 |
| NR_026563 | PSRC1 | 0.002704 | -0.9973 |
| NR_026563 | FOXO6 | 0.003994 | 0.996006 |
| NR_026563 | DGAT2L6 | 0.007494 | 0.992506 |
| NR_026563 | CATG00000053512.1 | 8.66E-04 | -0.99913 |
| NR_026563 | HLF | 9.18E-05 | 0.999908 |
| NR_026563 | SRRD | 0.008317 | 0.991683 |
| NR_026563 | CSTL1 | 0.006823 | 0.993177 |
| NR_026563 | FYB2 | 0.00584 | 0.99416 |
| NR_026563 | SLC12A3 | 0.001227 | -0.99877 |
| NR_026563 | PPP5D1 | 0.002765 | 0.997235 |
| NR_026563 | WNT8B | 0.007657 | 0.992343 |
| NR_026563 | PSMA1 | 0.007861 | -0.99214 |
| NR_026563 | UNC5C | 0.007909 | -0.99209 |
| NR_026563 | OXT | 0.002872 | 0.997128 |
| NR_026563 | FAM174A | 0.001061 | -0.99894 |
| NR_026563 | CFAP410 | 7.79E-04 | 0.999221 |
| NR_026563 | HPS1 | 0.007212 | 0.992788 |
| NR_138042 | ZFAND5 | 0.008329 | 0.991671 |
| NR_138042 | CATG00000056264.1 | 0.0074 | 0.9926 |
| NR_138042 | SSBP2 | 0.008055 | 0.991945 |
| NR_138042 | CLEC4G | 0.006898 | 0.993102 |
| NR_138042 | CD27 | 0.008534 | 0.991466 |
| NR_138042 | TMC2 | 0.003969 | 0.996031 |
| NR_138042 | NAGLU | 0.002288 | 0.997712 |
| NR_138042 | KRBA2 | 0.009674 | 0.990326 |
| NR_138042 | CLK3 | 3.52E-04 | 0.999648 |
| NR_138042 | TPRX1 | 0.003349 | 0.996651 |
| NR_138042 | AP3M2 | 0.009865 | -0.99014 |
| NR_138042 | CLCNKB | 0.00933 | 0.99067 |
| NR_138042 | PPTC7 | 0.003511 | -0.99649 |
| NR_138042 | CIB1 | 0.001844 | -0.99816 |
| ENST00000503415 | NUP54 | 0.009417 | 0.990583 |
| ENST00000503415 | MDK | 0.00261 | 0.99739 |
| ENST00000503415 | FTSJ1 | 0.005879 | 0.994121 |
| ENST00000503415 | ANXA3 | 0.00358 | 0.99642 |
| ENST00000503415 | MAP3K12 | 0.002217 | 0.997783 |
| ENST00000503415 | CLCN5 | 0.001732 | 0.998268 |
| ENST00000414039 | ACOT12 | 0.003064 | 0.996936 |
| ENST00000414039 | PALM3 | 0.0037 | -0.9963 |
| ENST00000414039 | BCAS4 | 6.52E-04 | 0.999348 |
| ENST00000414039 | PELP1 | 0.008387 | 0.991613 |
| ENST00000414039 | OR8B4 | 0.002755 | -0.99724 |
| ENST00000414039 | MYH4 | 0.006069 | 0.993931 |
| ENST00000414039 | KNCN | 0.004698 | 0.995302 |
| ENST00000414039 | FANCL | 0.002996 | 0.997004 |
| ENST00000414039 | CAMK2N2 | 0.005953 | 0.994047 |
| ENST00000525678 | PFDN6 | 5.78E-04 | -0.99942 |
| ENST00000525678 | ELOF1 | 0.001303 | 0.998697 |
| ENST00000525678 | CLEC1A | 0.006005 | -0.994 |
| ENST00000525678 | IMPA2 | 0.001942 | -0.99806 |
| ENST00000525678 | GGCT | 6.58E-04 | -0.99934 |
| ENST00000525678 | TMEM242 | 0.003655 | -0.99635 |
| ENST00000525678 | DGAT2L6 | 0.005982 | 0.994018 |
| ENST00000525678 | PRPF8 | 0.009676 | -0.99032 |
| ENST00000525678 | UNC45A | 0.004848 | 0.995152 |
| ENST00000525678 | THOC5 | 0.001957 | 0.998043 |
| ENST00000525678 | TUBGCP4 | 7.97E-04 | 0.999203 |
| ENST00000525678 | CHCHD6 | 0.005047 | 0.994953 |
| ENST00000525678 | CEP290 | 0.009615 | 0.990385 |
| ENST00000525678 | PPP5D1 | 0.007384 | 0.992616 |
| ENST00000525678 | JDP2 | 6.24E-04 | 0.999376 |
| ENST00000525678 | TAF8 | 0.003972 | -0.99603 |
| ENST00000525678 | PSMA1 | 0.003471 | -0.99653 |
| ENST00000525678 | MCM3AP | 4.14E-04 | 0.999586 |
| ENST00000443565 | NOC4L | 0.001714 | 0.998286 |
| ENST00000443565 | DNASE1L2 | 0.005551 | 0.994449 |
| ENST00000443565 | TUBB2A | 0.001742 | -0.99826 |
| ENST00000443565 | CDKL2 | 0.003332 | 0.996668 |
| ENST00000443565 | CR749689 | 0.005982 | -0.99402 |
| ENST00000580048 | EPB41L1 | 0.00441 | -0.99559 |
| ENST00000580048 | RASSF6 | 0.003965 | -0.99603 |
| ENST00000580048 | ZNF713 | 0.002425 | -0.99758 |
| ENST00000580048 | ABR | 0.006886 | -0.99311 |
| ENST00000580048 | CATG00000047316.1 | 0.003712 | -0.99629 |
| ENST00000580048 | SAMD1 | 0.006766 | 0.993234 |
| ENST00000580048 | ZMYND15 | 6.40E-04 | -0.99936 |
| ENST00000580048 | CATG00000057824.1 | 0.006111 | -0.99389 |
| ENST00000580048 | IQCF5 | 0.00635 | -0.99365 |
| ENST00000580048 | NTF3 | 0.005244 | -0.99476 |
| ENST00000580048 | DNAL4 | 0.00212 | 0.99788 |
| ENST00000580048 | FAM53B | 0.00551 | 0.99449 |
| ENST00000580048 | LDHB | 0.002795 | 0.997205 |
| ENST00000580048 | ZNF37A | 0.007345 | 0.992655 |
| ENST00000580048 | DICER1 | 0.004796 | 0.995204 |
| ENST00000412446 | DNAJC6 | 1.33E-04 | 0.999867 |
| ENST00000412446 | MFAP3L | 5.02E-04 | 0.999498 |
| ENST00000412446 | CATG00000086946.1 | 0.003552 | 0.996448 |
| ENST00000412446 | SPACA9 | 6.63E-04 | 0.999337 |
| ENST00000412446 | CMBL | 0.004063 | 0.995937 |
| ENST00000412446 | ORM1 | 0.004741 | 0.995259 |
| ENST00000431923 | INPP5J | 0.008075 | 0.991925 |
| ENST00000431923 | RTN1 | 0.003584 | -0.99642 |
| ENST00000431923 | EXOC4 | 0.006332 | 0.993668 |
| ENST00000431923 | EEF1D | 0.007622 | -0.99238 |
| ENST00000431923 | DBI | 0.003184 | -0.99682 |
| ENST00000431923 | AP3M2 | 0.003085 | -0.99692 |
| ENST00000431923 | SEC14L6 | 0.009068 | 0.990932 |
| ENST00000431923 | BMPER | 0.009796 | 0.990204 |
| ENST00000431923 | DEFA6 | 0.007169 | 0.992831 |
| ENST00000480281 | MIEN1 | 0.00628 | 0.99372 |
| ENST00000480281 | CATG00000021869.1 | 0.006627 | 0.993373 |
| ENST00000480281 | PLEKHG2 | 0.007362 | -0.99264 |
| ENST00000480281 | KCNJ11 | 0.008247 | 0.991753 |
| ENST00000480281 | TSPAN32 | 0.001927 | -0.99807 |
| ENST00000480281 | IQANK1 | 0.004631 | 0.995369 |
| ENST00000480281 | SLPI | 0.003442 | 0.996558 |
| ENST00000480281 | METTL3 | 0.008414 | 0.991586 |
| ENST00000480281 | C1QTNF9B | 0.002466 | 0.997534 |
| ENST00000480281 | KLRG2 | 0.004163 | -0.99584 |
| ENST00000480281 | UPP2 | 0.002843 | 0.997157 |
| ENST00000591809 | GFRA1 | 4.45E-04 | -0.99955 |
| ENST00000591809 | CATG00000092654.1 | 0.008729 | -0.99127 |
| ENST00000591809 | SYBU | 0.009751 | 0.990249 |
| ENST00000591809 | APBB3 | 0.008296 | 0.991704 |
| ENST00000591809 | EXOC2 | 0.004969 | -0.99503 |
| ENST00000591809 | GLIPR1L2 | 0.008281 | -0.99172 |
| ENST00000591809 | MRNIP | 0.001063 | 0.998937 |
| ENST00000591809 | ACAP3 | 0.005116 | 0.994884 |
| ENST00000591809 | ARSD | 0.002782 | -0.99722 |
| ENST00000591809 | PIKFYVE | 0.007221 | -0.99278 |
| ENST00000591809 | KIF21B | 0.006586 | 0.993414 |
| ENST00000591809 | TPRKB | 0.003097 | -0.9969 |
| ENST00000591809 | HEATR9 | 0.008245 | 0.991755 |
| ENST00000591809 | FANCE | 0.008107 | 0.991893 |
| ENST00000442326 | CXCL17 | 0.009926 | -0.99007 |
| ENST00000442326 | AL358113.1 | 0.009472 | -0.99053 |
| ENST00000442326 | SLIRP | 0.007768 | -0.99223 |
| ENST00000442326 | GYG2 | 0.002157 | -0.99784 |
| ENST00000442326 | SPEF1 | 0.004622 | -0.99538 |
| ENST00000618308 | LDLRAD4 | 0.009135 | -0.99087 |
| ENST00000618308 | DLEC1 | 0.00885 | 0.99115 |
| ENST00000618308 | CATG00000038628.1 | 0.009252 | 0.990748 |
| ENST00000414938 | GNL1 | 0.00782 | -0.99218 |
| ENST00000414938 | VRK3 | 0.008253 | 0.991747 |
| ENST00000414938 | BLCAP | 0.007854 | 0.992146 |
| ENST00000414938 | FANCD2OS | 0.00734 | 0.99266 |
| ENST00000414938 | CATG00000023328.1 | 0.001225 | -0.99877 |
| ENST00000414938 | EFEMP2 | 0.006006 | -0.99399 |
| ENST00000414938 | ACSL6 | 0.009973 | 0.990027 |
| ENST00000414938 | OR4X1 | 0.001917 | 0.998083 |
| ENST00000414938 | MAP3K5 | 3.58E-04 | 0.999642 |
| ENST00000414938 | HLA-F | 0.005088 | 0.994912 |
| ENST00000414938 | CATG00000024701.1 | 7.58E-05 | 0.999924 |
| NR_137440 | RSL1D1 | 0.004226 | -0.99577 |
| NR_137440 | SLC2A4 | 0.005925 | 0.994075 |
| NR_137440 | SYNDIG1L | 0.004374 | -0.99563 |
| NR_137440 | HIRA | 0.005386 | -0.99461 |
| NR_137440 | TMEM155 | 0.009839 | 0.990161 |
| NR_137440 | IL17RA | 9.54E-05 | 0.999905 |
| NR_137440 | PLPPR4 | 0.007835 | -0.99217 |
| NR_137440 | AHSP | 0.007147 | 0.992853 |
| NR_137440 | HLA-A | 0.009363 | -0.99064 |
| NR_137440 | AC092073.1 | 0.007073 | 0.992927 |
| NR_137440 | KCNK10 | 0.00759 | 0.99241 |
| NR_137440 | LYPD1 | 8.28E-04 | 0.999172 |
| NR_137440 | ELSPBP1 | 0.008041 | 0.991959 |
| NR_137440 | SLC6A5 | 0.002287 | 0.997713 |
| NR_137440 | RRBP1 | 0.007361 | -0.99264 |
| NR_137440 | VPS13A | 0.007188 | 0.992812 |
| ENST00000495702 | PLEKHG2 | 0.009332 | -0.99067 |
| ENST00000495702 | CATG00000061038.1 | 0.006847 | 0.993153 |
| ENST00000495702 | MAPKBP1 | 0.001999 | -0.998 |
| ENST00000495702 | AC093157.1 | 0.003615 | 0.996385 |
| ENST00000495702 | ZPR1 | 0.00888 | -0.99112 |
| ENST00000495702 | CATG00000026669.1 | 0.001175 | 0.998825 |
| ENST00000495702 | OR10R2 | 0.008687 | -0.99131 |
| ENST00000495702 | DDIT4 | 0.00954 | 0.99046 |
| ENST00000495702 | CARNS1 | 0.001542 | 0.998458 |
| ENST00000452809 | CATG00000060074.1 | 0.009834 | 0.990166 |
| ENST00000452809 | OCLN | 0.003192 | 0.996808 |
| ENST00000452809 | FAR1 | 0.006625 | 0.993375 |
| ENST00000452809 | TNFSF13B | 0.006414 | 0.993586 |
| ENST00000452809 | AGAP1 | 0.001355 | -0.99865 |
| ENST00000452809 | CATG00000051841.1 | 0.009468 | -0.99053 |
| ENST00000452809 | HILPDA | 0.00377 | -0.99623 |
| ENST00000452809 | KIF27 | 0.003983 | 0.996017 |
| ENST00000394082 | AHNAK2 | 0.005233 | 0.994767 |
| ENST00000394082 | NCCRP1 | 0.005093 | 0.994907 |
| ENST00000394082 | C20orf203 | 0.00773 | 0.99227 |
| ENST00000394082 | DUSP15 | 0.004891 | 0.995109 |
| ENST00000394082 | HOMER3 | 0.005231 | -0.99477 |
| ENST00000394082 | AASDHPPT | 0.005044 | 0.994956 |
| ENST00000394082 | ARPC4-TTLL3 | 5.90E-04 | -0.99941 |
| ENST00000394082 | MAMLD1 | 0.004056 | -0.99594 |
| ENST00000394082 | ZNF214 | 0.007426 | -0.99257 |
| ENST00000394082 | ZNF624 | 0.00592 | 0.99408 |
| ENST00000394082 | OR5D18 | 0.005753 | -0.99425 |
| ENST00000533296 | PZP | 0.001552 | 0.998448 |
| ENST00000533296 | CLEC4D | 1.43E-04 | 0.999857 |
| ENST00000533296 | CLRN2 | 0.007319 | 0.992681 |
| ENST00000533296 | SLC11A1 | 0.00864 | 0.99136 |
| NR_034015 | CATG00000060074.1 | 0.007348 | 0.992652 |
| NR_034015 | OCLN | 0.004612 | 0.995388 |
| NR_034015 | FAR1 | 0.007748 | 0.992252 |
| NR_034015 | TNFSF13B | 0.005175 | 0.994825 |
| NR_034015 | AGAP1 | 7.84E-04 | -0.99922 |
| NR_034015 | CATG00000051841.1 | 0.007049 | -0.99295 |
| NR_034015 | HILPDA | 0.005233 | -0.99477 |
| NR_034015 | KIF27 | 0.00585 | 0.99415 |
| ENST00000506463 | MINK1 | 0.005613 | -0.99439 |
| ENST00000506463 | DUSP9 | 0.003478 | -0.99652 |
| ENST00000506463 | RAB5B | 0.009384 | 0.990616 |
| ENST00000506463 | CATG00000096017.1 | 0.004015 | 0.995985 |
| ENST00000470300 | CATG00000080699.1 | 0.003684 | -0.99632 |
| ENST00000470300 | AL358113.1 | 0.004538 | -0.99546 |
| ENST00000470300 | CHST12 | 0.007962 | -0.99204 |
| ENST00000470300 | HIPK2 | 4.02E-04 | 0.999598 |
| ENST00000470300 | PCDHB8 | 0.007962 | -0.99204 |
| ENST00000564168 | TBX3 | 0.004002 | 0.995998 |
| ENST00000564168 | AHSP | 0.001265 | 0.998735 |
| ENST00000564168 | KRT83 | 0.006729 | 0.993271 |
| ENST00000564168 | AC092073.1 | 0.005254 | 0.994746 |
| ENST00000564168 | CATG00000012021.1 | 0.001886 | -0.99811 |
| ENST00000564168 | TGFBR3 | 0.009226 | 0.990774 |
| ENST00000564168 | ESRRB | 0.004174 | 0.995826 |
| ENST00000564168 | GSG1L | 0.00964 | 0.99036 |
| ENST00000564168 | IFT122 | 0.005594 | 0.994406 |
| ENST00000564168 | SIGLEC7 | 0.002166 | 0.997834 |
| ENST00000564168 | CATG00000034210.1 | 0.003896 | 0.996104 |
| ENST00000564168 | RRBP1 | 0.005122 | -0.99488 |
| ENST00000564168 | MORN3 | 0.007844 | -0.99216 |
| ENST00000564168 | CAPN15 | 0.00567 | 0.99433 |
| ENST00000564168 | CCDC174 | 0.009759 | -0.99024 |
| ENST00000564168 | FAM151A | 0.003849 | 0.996151 |
| ENST00000589723 | CATG00000080699.1 | 0.003323 | -0.99668 |
| ENST00000589723 | AL358113.1 | 0.005846 | -0.99415 |
| ENST00000589723 | ZDHHC16 | 0.005723 | 0.994277 |
| ENST00000589723 | MACROD2 | 0.009591 | -0.99041 |
| ENST00000589723 | CHST12 | 0.00435 | -0.99565 |
| ENST00000589723 | HIPK2 | 4.59E-04 | 0.999541 |
| ENST00000589723 | PCDHB8 | 0.00435 | -0.99565 |
| ENST00000607039 | GRINA | 0.007153 | 0.992847 |
| ENST00000607039 | RSL1D1 | 0.008898 | -0.9911 |
| ENST00000607039 | LILRB1 | 0.008385 | -0.99162 |
| ENST00000607039 | IL17RA | 0.009661 | 0.990339 |
| ENST00000607039 | TBX3 | 0.006042 | 0.993958 |
| ENST00000607039 | AHSP | 0.003094 | 0.996906 |
| ENST00000607039 | KRT83 | 0.008884 | 0.991116 |
| ENST00000607039 | AC092073.1 | 0.008262 | 0.991738 |
| ENST00000607039 | CATG00000012021.1 | 0.002252 | -0.99775 |
| ENST00000607039 | ESRRB | 0.003931 | 0.996069 |
| ENST00000607039 | IFT122 | 0.003556 | 0.996444 |
| ENST00000607039 | SIGLEC7 | 0.001401 | 0.998599 |
| ENST00000607039 | PTPN23 | 0.008764 | 0.991236 |
| ENST00000607039 | CATG00000034210.1 | 0.007496 | 0.992504 |
| ENST00000607039 | AGO2 | 0.006833 | 0.993167 |
| ENST00000607039 | RRBP1 | 0.008176 | -0.99182 |
| ENST00000607039 | FAM151A | 0.003351 | 0.996649 |
| ENST00000532451 | ECSIT | 0.006212 | 0.993788 |
| ENST00000532451 | SIRPG | 0.007416 | 0.992584 |
| ENST00000532451 | CHST8 | 0.009706 | 0.990294 |
| ENST00000532451 | AHSP | 0.009265 | 0.990735 |
| ENST00000532451 | AC092073.1 | 0.003828 | 0.996172 |
| ENST00000532451 | FRG1 | 0.006166 | -0.99383 |
| ENST00000532451 | TGFBR3 | 0.001368 | 0.998632 |
| ENST00000532451 | SLC22A6 | 0.001169 | -0.99883 |
| ENST00000532451 | COA1 | 0.003855 | -0.99614 |
| ENST00000532451 | COA5 | 3.15E-04 | -0.99968 |
| ENST00000532451 | RRBP1 | 0.003832 | -0.99617 |
| ENST00000532451 | MORN3 | 0.001868 | -0.99813 |
| ENST00000532451 | VPS13A | 0.002883 | 0.997117 |
| ENST00000532451 | FGF9 | 0.006796 | -0.9932 |
| ENST00000532451 | CAPN15 | 0.00513 | 0.99487 |
| NR_036499 | PLEKHA2 | 0.004205 | 0.995795 |
| NR_036499 | PLAG1 | 0.006089 | 0.993911 |
| NR_036499 | VNN3 | 0.008694 | -0.99131 |
| NR_036499 | DYNC2H1 | 0.005809 | -0.99419 |
| NR_110267 | ZC3H7B | 0.006901 | -0.9931 |
| NR_110267 | CATG00000061038.1 | 7.01E-04 | 0.999299 |
| NR_110267 | OR10R2 | 0.006328 | -0.99367 |
| NR_110267 | DDIT4 | 0.003447 | 0.996553 |
| ENST00000460115 | SIRPG | 0.006835 | 0.993165 |
| ENST00000460115 | YWHAB | 4.87E-04 | 0.999513 |
| ENST00000460115 | CACHD1 | 0.005759 | 0.994241 |
| ENST00000460115 | CHST8 | 0.009762 | 0.990238 |
| ENST00000460115 | GSTA4 | 0.004394 | 0.995606 |
| ENST00000460115 | TBX3 | 0.00877 | 0.99123 |
| ENST00000460115 | GPR161 | 0.006574 | 0.993426 |
| ENST00000460115 | EIF1AD | 0.009803 | 0.990197 |
| ENST00000460115 | KRT83 | 0.00762 | 0.99238 |
| ENST00000460115 | CATG00000022188.1 | 0.002868 | 0.997132 |
| ENST00000460115 | CAPRIN2 | 0.007224 | 0.992776 |
| ENST00000460115 | CLEC5A | 0.002828 | 0.997172 |
| ENST00000460115 | CATG00000089121.1 | 0.006425 | -0.99358 |
| ENST00000460115 | RBM7 | 4.15E-04 | 0.999585 |
| ENST00000460115 | GSG1L | 0.002367 | 0.997633 |
| ENST00000460115 | ERFE | 0.004419 | -0.99558 |
| ENST00000460115 | SNRPD2 | 7.36E-04 | 0.999264 |
| ENST00000460115 | SPATA31D1 | 0.002812 | 0.997188 |
| ENST00000460115 | CATG00000038058.1 | 0.004146 | 0.995854 |
| ENST00000460115 | CATG00000034210.1 | 0.007427 | 0.992573 |
| ENST00000460115 | ZC3H12D | 0.001803 | -0.9982 |
| ENST00000460115 | LIM2 | 0.00459 | 0.99541 |
| ENST00000460115 | PLCL1 | 0.004228 | -0.99577 |
| ENST00000460115 | TNFRSF13B | 8.06E-04 | -0.99919 |
| ENST00000460115 | FGF9 | 0.006957 | -0.99304 |
| ENST00000618092 | HMGB2 | 0.009706 | -0.99029 |
| ENST00000618092 | NR2F2 | 0.009993 | 0.990007 |
| ENST00000618092 | IGFN1 | 0.004665 | -0.99534 |
| ENST00000618092 | SLC7A4 | 0.00197 | 0.99803 |
| ENST00000618092 | GPR161 | 0.009214 | 0.990786 |
| ENST00000618092 | EIF1AD | 0.009326 | 0.990674 |
| ENST00000618092 | SMPD1 | 0.007217 | 0.992783 |
| ENST00000618092 | SRRD | 0.007968 | 0.992032 |
| ENST00000618092 | TPTE | 0.007078 | 0.992922 |
| ENST00000618092 | SAMD1 | 0.006723 | -0.99328 |
| ENST00000618092 | CPOX | 0.004396 | 0.995604 |
| ENST00000618092 | CATG00000057824.1 | 0.007586 | 0.992414 |
| ENST00000618092 | WNT2 | 0.006433 | 0.993567 |
| ENST00000618092 | PAK3 | 0.004963 | 0.995037 |
| ENST00000618092 | KLF6 | 1.12E-04 | -0.99989 |
| ENST00000618092 | LBP | 0.004058 | 0.995942 |
| ENST00000618092 | FAM53B | 0.006603 | -0.9934 |
| ENST00000618092 | LIM2 | 0.00928 | 0.99072 |
| ENST00000618092 | REG4 | 0.003881 | -0.99612 |
| NR_146896 | TMED3 | 0.008629 | 0.991371 |
| NR_146896 | ACOT12 | 0.006673 | -0.99333 |
| NR_146896 | VRK3 | 0.00741 | -0.99259 |
| NR_146896 | PALM3 | 0.006119 | 0.993881 |
| NR_146896 | BLCAP | 0.006475 | -0.99353 |
| NR_146896 | BCAS4 | 0.002875 | -0.99713 |
| NR_146896 | OR8B4 | 0.004856 | 0.995144 |
| NR_146896 | ACSL6 | 0.006374 | -0.99363 |
| NR_146896 | KNCN | 0.008255 | -0.99174 |
| NR_146896 | FANCL | 0.004832 | -0.99517 |
| NR_144312 | C16orf45 | 0.00645 | 0.99355 |
| NR_144312 | PLAGL1 | 0.006647 | 0.993353 |
| NR_144312 | MYO7A | 0.002487 | -0.99751 |
| NR_144312 | AIFM3 | 0.009172 | 0.990828 |
| NR_144312 | ABHD5 | 0.006121 | -0.99388 |
| NR_144312 | OCSTAMP | 0.003757 | -0.99624 |
| NR_144312 | SP140 | 0.004933 | 0.995067 |
| NR_144312 | IL1R2 | 7.86E-04 | -0.99921 |
| NR_144312 | CROT | 1.92E-04 | 0.999808 |
| NR_110318 | B3GNT3 | 0.003622 | -0.99638 |
| NR_110318 | HMGB2 | 0.004895 | -0.99511 |
| NR_110318 | PLIN4 | 0.008313 | 0.991687 |
| NR_110318 | FOXO6 | 6.15E-04 | 0.999385 |
| NR_110318 | CXXC1 | 0.008842 | -0.99116 |
| NR_110318 | DGAT2L6 | 0.006033 | 0.993967 |
| NR_110318 | CATG00000053512.1 | 0.009415 | -0.99058 |
| NR_110318 | HLF | 0.005023 | 0.994977 |
| NR_110318 | SRRD | 6.91E-04 | 0.999309 |
| NR_110318 | TPTE | 0.002672 | 0.997328 |
| NR_110318 | CSTL1 | 0.002482 | 0.997518 |
| NR_110318 | FYB2 | 9.82E-04 | 0.999018 |
| NR_110318 | SLC12A3 | 0.008244 | -0.99176 |
| NR_110318 | WNT2 | 0.00823 | 0.99177 |
| NR_110318 | PAK3 | 0.005193 | 0.994807 |
| NR_110318 | PPP5D1 | 0.009099 | 0.990901 |
| NR_110318 | OXT | 2.14E-04 | 0.999786 |
| NR_110318 | FAM174A | 0.001491 | -0.99851 |
| NR_110318 | REG4 | 0.009801 | -0.9902 |
| NR_110318 | CFAP410 | 0.006184 | 0.993816 |
| ENST00000504032 | MMP1 | 0.004167 | -0.99583 |
| ENST00000504032 | ACTL7B | 0.006336 | 0.993664 |
| ENST00000504032 | C15orf65 | 0.002353 | 0.997647 |
| ENST00000504032 | NTNG1 | 0.00396 | -0.99604 |
| ENST00000504032 | NDUFAF8 | 0.006982 | -0.99302 |
| ENST00000504032 | PMEPA1 | 0.004503 | 0.995497 |
| ENST00000504032 | PMIS2 | 0.008183 | 0.991817 |
| ENST00000504032 | FCRL1 | 0.004898 | 0.995102 |
| ENST00000514957 | LCK | 0.002812 | -0.99719 |
| ENST00000514957 | AGPS | 0.001071 | -0.99893 |
| ENST00000514957 | TEX13D | 1.07E-04 | 0.999893 |
| ENST00000514957 | IRX3 | 0.002683 | 0.997317 |
| ENST00000514957 | SNX16 | 4.28E-04 | -0.99957 |
| ENST00000514957 | SPATA13 | 0.005416 | 0.994584 |
| ENST00000514957 | BHLHB9 | 0.006983 | 0.993017 |
| ENST00000514957 | PLTP | 0.003239 | 0.996761 |
| ENST00000514957 | PLCH2 | 0.001591 | 0.998409 |
| ENST00000514957 | COBL | 0.003256 | 0.996744 |
| ENST00000514957 | RRP9 | 0.00621 | 0.99379 |
| ENST00000514957 | CATG00000038465.1 | 0.00335 | 0.99665 |
| ENST00000514957 | SIPA1L2 | 0.00705 | 0.99295 |
| ENST00000514957 | PIPOX | 0.003828 | -0.99617 |
| ENST00000371390 | RIPK1 | 0.006059 | -0.99394 |
| ENST00000371390 | HDHD5 | 0.008897 | -0.9911 |
| ENST00000371390 | PTGER1 | 0.006721 | -0.99328 |
| ENST00000371390 | POP1 | 0.002439 | 0.997561 |
| ENST00000371390 | PLD3 | 0.00745 | 0.99255 |
| ENST00000371390 | TMEM230 | 0.002136 | 0.997864 |
| ENST00000371390 | RAB8A | 0.003918 | 0.996082 |
| ENST00000371390 | EEF1B2 | 0.003553 | -0.99645 |
| ENST00000371390 | KIAA1211L | 0.002592 | -0.99741 |
| ENST00000371390 | TNFAIP6 | 0.001758 | 0.998242 |
| ENST00000371390 | PTPRK | 0.004364 | -0.99564 |
| ENST00000371390 | CATG00000039609.1 | 0.007321 | -0.99268 |
| ENST00000577295 | FOXJ3 | 0.002546 | 0.997454 |
| ENST00000577295 | OR10H1 | 6.69E-04 | 0.999331 |
| ENST00000413151 | CPSF3 | 0.001698 | 0.998302 |
| ENST00000413151 | C11orf71 | 0.001839 | 0.998161 |
| ENST00000413151 | MYLK3 | 0.001771 | 0.998229 |
| ENST00000413151 | SIRT6 | 0.008412 | 0.991588 |
| ENST00000413151 | TNK2 | 0.006234 | 0.993766 |
| ENST00000449466 | LCK | 0.006484 | -0.99352 |
| ENST00000449466 | CATG00000038465.1 | 0.00917 | 0.99083 |
| ENST00000449466 | CFLAR | 4.36E-04 | -0.99956 |
| ENST00000449466 | CHI3L1 | 0.00738 | 0.99262 |
| ENST00000449466 | C17orf47 | 2.36E-04 | 0.999764 |
| ENST00000440005 | STRADB | 0.009367 | 0.990633 |
| ENST00000440005 | EGF | 0.004339 | 0.995661 |
| ENST00000440005 | SHROOM3 | 0.009826 | 0.990174 |
| ENST00000440005 | CATG00000099282.1 | 0.005521 | -0.99448 |
| ENST00000440005 | RTL8B | 0.002391 | -0.99761 |
| ENST00000440005 | ASB14 | 0.001116 | -0.99888 |
| ENST00000440005 | ABCA13 | 0.009267 | 0.990733 |
| ENST00000440005 | RHD | 4.00E-04 | 0.9996 |
| ENST00000440005 | CATG00000117842.1 | 0.001352 | 0.998648 |
| ENST00000440005 | C16orf86 | 0.001635 | -0.99836 |
| ENST00000418528 | FCN2 | 1.12E-05 | 0.999989 |
| ENST00000418528 | CATG00000063531.1 | 0.007107 | 0.992893 |
| ENST00000418528 | UBE2V1 | 0.009037 | 0.990963 |
| ENST00000418528 | CEP44 | 0.009037 | 0.990963 |
| ENST00000418528 | NFE2L2 | 0.003825 | -0.99617 |
| ENST00000418528 | SIVA1 | 3.43E-04 | -0.99966 |
| ENST00000418528 | CDK11B | 0.008674 | -0.99133 |
| ENST00000418528 | TCTEX1D4 | 0.006578 | -0.99342 |
| ENST00000418528 | SMIM6 | 0.001666 | 0.998334 |
| ENST00000418528 | SH3RF3 | 0.003889 | -0.99611 |
| ENST00000418528 | ACMSD | 0.008701 | -0.9913 |
| NR_130126 | CATG00000056264.1 | 0.007149 | 0.992851 |
| NR_130126 | CATG00000003494.1 | 6.83E-04 | 0.999317 |
| NR_130126 | CACHD1 | 0.004592 | 0.995408 |
| NR_130126 | CLEC4G | 0.004417 | 0.995583 |
| NR_130126 | PCDHGA3 | 0.008775 | 0.991225 |
| NR_130126 | TPRX1 | 0.007735 | 0.992265 |
| NR_130126 | OAS1 | 0.003583 | 0.996417 |
| NR_130126 | CLCNKB | 0.003081 | 0.996919 |
| NR_130126 | DCD | 0.006377 | 0.993623 |
| NR_130126 | CSNK1A1 | 0.003963 | -0.99604 |
| NR_130126 | INO80B | 6.34E-04 | 0.999366 |
| NR_130126 | ACP6 | 0.005344 | -0.99466 |
| NR_130126 | RPL17 | 0.002573 | -0.99743 |
| NR_130126 | PLCL1 | 0.005857 | -0.99414 |
| NR_130126 | GABPA | 2.63E-04 | 0.999737 |
| ENST00000491322 | CCDC114 | 0.006926 | -0.99307 |
| ENST00000491322 | C1orf198 | 0.001963 | -0.99804 |
| ENST00000491322 | LHFPL6 | 0.005068 | 0.994932 |
| ENST00000491322 | TATDN3 | 0.007349 | -0.99265 |
| ENST00000491322 | BVES | 4.49E-04 | 0.999551 |
| ENST00000491322 | ARHGEF33 | 0.009737 | 0.990263 |
| ENST00000491322 | COMMD2 | 0.001327 | -0.99867 |
| ENST00000491322 | MRPL10 | 0.009366 | 0.990634 |
| ENST00000491322 | ANKS3 | 0.003643 | 0.996357 |
| ENST00000491322 | ATXN7 | 0.006631 | 0.993369 |
| ENST00000491322 | ASPRV1 | 0.002876 | -0.99712 |
| ENST00000451595 | ELOF1 | 0.006335 | 0.993665 |
| ENST00000451595 | GGCT | 0.003911 | -0.99609 |
| ENST00000451595 | PCP4 | 0.009357 | 0.990643 |
| ENST00000451595 | TMEM242 | 5.44E-04 | -0.99946 |
| ENST00000451595 | NLRP9 | 0.009044 | 0.990956 |
| ENST00000451595 | DHODH | 0.006472 | -0.99353 |
| ENST00000451595 | PRPF8 | 0.002581 | -0.99742 |
| ENST00000451595 | UNC45A | 0.003472 | 0.996528 |
| ENST00000451595 | CCDC149 | 0.002559 | 0.997441 |
| ENST00000451595 | THOC5 | 0.001578 | 0.998422 |
| ENST00000451595 | TUBGCP4 | 0.005086 | 0.994914 |
| ENST00000451595 | RACK1 | 0.005027 | 0.994973 |
| ENST00000451595 | CHCHD6 | 2.82E-04 | 0.999718 |
| ENST00000451595 | TMEM51 | 0.001139 | 0.998861 |
| ENST00000451595 | CEP290 | 0.004936 | 0.995064 |
| ENST00000451595 | TIGD3 | 0.009572 | -0.99043 |
| ENST00000451595 | JDP2 | 0.007945 | 0.992055 |
| ENST00000451595 | MCM3AP | 0.004047 | 0.995953 |
| ENST00000451595 | RPUSD2 | 0.003906 | -0.99609 |
| ENST00000451595 | ZNF107 | 0.004717 | -0.99528 |
| ENST00000451595 | CEP63 | 4.18E-04 | -0.99958 |
| ENST00000475255 | SLC38A10 | 0.006893 | 0.993107 |
| ENST00000475255 | ZNF30 | 0.004014 | -0.99599 |
| ENST00000475255 | SZRD1 | 0.003924 | -0.99608 |
| ENST00000475255 | FAM84A | 0.006959 | -0.99304 |
| ENST00000475255 | GMPPA | 6.09E-04 | -0.99939 |
| ENST00000475255 | CATG00000053936.1 | 0.008572 | -0.99143 |
| ENST00000475255 | ARHGEF40 | 0.008526 | 0.991474 |
| ENST00000475255 | CATG00000107162.1 | 0.004082 | 0.995918 |
| ENST00000475255 | MAP7 | 0.008235 | 0.991765 |
| ENST00000475255 | C16orf78 | 0.007279 | 0.992721 |
| ENST00000475255 | EFCAB8 | 0.00185 | 0.99815 |
| ENST00000475255 | MSI1 | 0.003373 | 0.996627 |
| ENST00000456176 | TMED3 | 0.006602 | -0.9934 |
| ENST00000456176 | GNL1 | 0.009893 | -0.99011 |
| ENST00000456176 | DEAF1 | 0.003075 | 0.996925 |
| ENST00000456176 | FNDC10 | 0.008888 | 0.991112 |
| ENST00000456176 | VRK3 | 0.001099 | 0.998901 |
| ENST00000456176 | BLCAP | 0.001528 | 0.998472 |
| ENST00000456176 | FANCD2OS | 0.003934 | 0.996066 |
| ENST00000456176 | PNPLA1 | 0.003596 | 0.996404 |
| ENST00000456176 | IDH1 | 0.005282 | -0.99472 |
| ENST00000456176 | ZNF607 | 0.007304 | 0.992696 |
| ENST00000456176 | CATG00000023328.1 | 0.007841 | -0.99216 |
| ENST00000456176 | ACSL6 | 0.001015 | 0.998985 |
| ENST00000456176 | ZNF431 | 0.00609 | 0.99391 |
| NR_104675 | CATG00000061038.1 | 0.004089 | -0.99591 |
| NR_104675 | MAPKBP1 | 0.005435 | 0.994565 |
| NR_104675 | AC093157.1 | 0.007732 | -0.99227 |
| NR_104675 | CATG00000026669.1 | 0.00195 | -0.99805 |
| NR_104675 | OR10R2 | 0.008715 | 0.991285 |
| NR_104675 | DDIT4 | 0.008099 | -0.9919 |
| NR_104675 | CARNS1 | 0.00199 | -0.99801 |
| ENST00000582251 | NAAA | 0.001871 | 0.998129 |
| ENST00000582251 | RFC5 | 0.001337 | -0.99866 |
| ENST00000582251 | CMBL | 0.003312 | -0.99669 |
| ENST00000582251 | DARS | 6.60E-04 | 0.99934 |
| ENST00000582251 | CATG00000038628.1 | 0.00951 | -0.99049 |
| ENST00000582251 | OR5T2 | 0.009326 | 0.990674 |
| ENST00000530893 | ACSBG1 | 0.008513 | 0.991487 |
| ENST00000530893 | EIF3L | 0.001426 | 0.998574 |
| ENST00000530893 | KRTAP15-1 | 0.00137 | -0.99863 |
| ENST00000530893 | CATG00000107403.1 | 0.003281 | -0.99672 |
| ENST00000425837 | EXOC4 | 0.003316 | 0.996684 |
| ENST00000425837 | TMEM132B | 0.003263 | 0.996737 |
| ENST00000425837 | EEF1D | 0.00244 | -0.99756 |
| ENST00000425837 | DBI | 0.00268 | -0.99732 |
| ENST00000425837 | SEC14L6 | 0.002041 | 0.997959 |
| ENST00000425837 | IL4I1 | 0.009908 | -0.99009 |
| ENST00000425837 | NEMP2 | 0.007096 | 0.992904 |
| ENST00000425837 | NEFM | 0.005094 | -0.99491 |
| ENST00000425837 | TBKBP1 | 0.002872 | -0.99713 |
| ENST00000425837 | SCAMP3 | 0.007902 | -0.9921 |
| ENST00000425837 | PDE3B | 0.003315 | -0.99669 |
| ENST00000425837 | DEFA6 | 0.008328 | 0.991672 |
| NR_135545 | RIPK1 | 0.008257 | 0.991743 |
| NR_135545 | NAPA | 0.003939 | -0.99606 |
| NR_135545 | PPP1CB | 1.90E-04 | -0.99981 |
| NR_135545 | P3H3 | 0.00913 | 0.99087 |
| NR_135545 | PTGER1 | 0.004677 | 0.995323 |
| NR_135545 | PGM5 | 0.005114 | 0.994886 |
| NR_135545 | CATG00000107158.1 | 0.001984 | -0.99802 |
| NR_135545 | CATG00000110054.1 | 0.00185 | -0.99815 |
| NR_135545 | YY2 | 0.004265 | 0.995735 |
| NR_135545 | RCSD1 | 0.004097 | 0.995903 |
| NR_135545 | LY6G6C | 0.009549 | 0.990451 |
| NR_135545 | KIAA1211L | 0.009666 | 0.990334 |
| NR_135545 | CHI3L1 | 0.00487 | -0.99513 |
| NR_135545 | CATG00000020281.1 | 3.76E-04 | 0.999624 |
| NR_105039 | CATG00000003494.1 | 0.004418 | -0.99558 |
| NR_105039 | CLEC4G | 0.007897 | -0.9921 |
| NR_105039 | CD27 | 0.008174 | -0.99183 |
| NR_105039 | FAM90A1 | 0.00839 | 0.99161 |
| NR_105039 | IGSF9B | 0.008959 | -0.99104 |
| NR_105039 | PCDHGA3 | 0.004138 | -0.99586 |
| NR_105039 | OAS1 | 0.005678 | -0.99432 |
| NR_105039 | CLCNKB | 0.003339 | -0.99666 |
| NR_105039 | DCD | 0.001926 | -0.99807 |
| NR_105039 | CSNK1A1 | 0.004997 | 0.995003 |
| NR_105039 | ZNF587B | 0.008849 | 0.991151 |
| NR_105039 | ELF2 | 0.005539 | 0.994461 |
| NR_105039 | INO80B | 0.004401 | -0.9956 |
| NR_105039 | ACP6 | 9.56E-04 | 0.999044 |
| NR_105039 | RPL17 | 0.004538 | 0.995462 |
| NR_105039 | GABPA | 0.003289 | -0.99671 |
| ENST00000428954 | DNAJC6 | 0.002771 | 0.997229 |
| ENST00000428954 | MFAP3L | 0.00652 | 0.99348 |
| ENST00000428954 | LGALS1 | 0.009376 | -0.99062 |
| ENST00000428954 | SPACA9 | 0.002482 | 0.997518 |
| ENST00000428954 | CMBL | 0.003656 | 0.996344 |
| ENST00000428954 | OR5T2 | 0.004856 | -0.99514 |
| NR_104046 | TBC1D29 | 0.00708 | 0.99292 |
| NR_104046 | CCDC42 | 0.003476 | 0.996524 |
| ENST00000631061 | TBKBP1 | 0.009619 | 0.990381 |
| ENST00000631061 | WT1 | 0.002862 | 0.997138 |
| ENST00000631061 | CATG00000039284.1 | 0.001679 | 0.998321 |
| ENST00000631061 | SCAMP3 | 0.00396 | 0.99604 |
| ENST00000453087 | SLC38A10 | 0.004466 | 0.995534 |
| ENST00000453087 | ZNF30 | 0.006535 | -0.99346 |
| ENST00000453087 | SZRD1 | 0.007022 | -0.99298 |
| ENST00000453087 | PLEKHD1 | 2.88E-04 | -0.99971 |
| ENST00000453087 | MLLT1 | 1.83E-04 | 0.999817 |
| ENST00000453087 | STMND1 | 0.00964 | 0.99036 |
| ENST00000453087 | DNAH6 | 5.14E-04 | 0.999486 |
| ENST00000453087 | MAP7 | 0.003572 | 0.996428 |
| ENST00000544657 | PPARD | 0.001138 | 0.998862 |
| ENST00000544657 | PTPRN | 0.003184 | 0.996816 |
| ENST00000544657 | PRKDC | 1.44E-04 | -0.99986 |
| ENST00000544657 | ACO1 | 0.002299 | 0.997701 |
| ENST00000544657 | USF2 | 0.003022 | -0.99698 |
| ENST00000544657 | SMAP2 | 0.00509 | -0.99491 |
| ENST00000567801 | PTPN6 | 0.001232 | -0.99877 |
| ENST00000567801 | DNAJA4 | 0.005494 | 0.994506 |
| ENST00000567801 | NOL9 | 0.00371 | -0.99629 |
| ENST00000567801 | PMPCA | 0.007855 | 0.992145 |
| ENST00000567801 | CAPNS1 | 0.007285 | -0.99272 |
| ENST00000567801 | SPATA13 | 0.008598 | 0.991402 |
| ENST00000567801 | SHC2 | 0.007684 | 0.992316 |
| ENST00000567801 | KRTAP4-7 | 0.004765 | 0.995235 |
| ENST00000567801 | SPSB2 | 0.006876 | 0.993124 |
| ENST00000567801 | SIPA1L2 | 0.006769 | 0.993231 |
| ENST00000354432 | EXOC4 | 0.005395 | 0.994605 |
| ENST00000354432 | TRMT11 | 0.00921 | -0.99079 |
| ENST00000354432 | ADGRE1 | 0.006374 | -0.99363 |
| ENST00000354432 | TMEM132B | 4.65E-04 | 0.999535 |
| ENST00000354432 | EEF1D | 0.006266 | -0.99373 |
| ENST00000354432 | DBI | 0.007201 | -0.9928 |
| ENST00000354432 | SEC14L6 | 0.001504 | 0.998496 |
| ENST00000354432 | IL4I1 | 0.003105 | -0.99689 |
| ENST00000354432 | NEMP2 | 0.006385 | 0.993615 |
| ENST00000354432 | NEFM | 0.00371 | -0.99629 |
| ENST00000354432 | TBKBP1 | 0.00294 | -0.99706 |
| ENST00000354432 | WT1 | 0.008648 | -0.99135 |
| ENST00000354432 | SCAMP3 | 0.006715 | -0.99328 |
| ENST00000354432 | PDE3B | 5.81E-04 | -0.99942 |
| NR_146599 | HIGD2A | 0.00955 | 0.99045 |
| NR_146599 | SYNDIG1L | 0.001985 | 0.998015 |
| NR_146599 | CATG00000101330.1 | 0.009916 | 0.990084 |
| NR_146599 | IL17RA | 0.003236 | -0.99676 |
| NR_146599 | KRTAP10-6 | 0.007193 | -0.99281 |
| NR_146599 | AHSP | 0.008289 | -0.99171 |
| NR_146599 | AP002990.1 | 0.007566 | 0.992434 |
| NR_146599 | AC092073.1 | 0.004785 | -0.99522 |
| NR_146599 | TGFBR3 | 0.005299 | -0.9947 |
| NR_146599 | LYPD1 | 0.003672 | -0.99633 |
| NR_146599 | ELSPBP1 | 0.004102 | -0.9959 |
| NR_146599 | SLC6A5 | 1.65E-04 | -0.99983 |
| NR_146599 | RRBP1 | 0.005088 | 0.994912 |
| NR_146599 | MORN3 | 0.008032 | 0.991968 |
| NR_146599 | VPS13A | 0.001691 | -0.99831 |
| ENST00000503522 | WDR90 | 0.005488 | -0.99451 |
| ENST00000503522 | SORCS2 | 0.003176 | -0.99682 |
| ENST00000503522 | PPP1R16B | 0.009975 | -0.99003 |
| ENST00000457746 | ANKRD13D | 0.001176 | -0.99882 |
| ENST00000457746 | LCK | 0.006947 | -0.99305 |
| ENST00000457746 | CATG00000087963.1 | 0.006811 | 0.993189 |
| ENST00000457746 | AGPS | 0.004426 | -0.99557 |
| ENST00000457746 | TEX13D | 0.008024 | 0.991976 |
| ENST00000457746 | EPHB6 | 0.007847 | 0.992153 |
| ENST00000457746 | SNX16 | 0.005962 | -0.99404 |
| ENST00000457746 | DNAJB13 | 0.008227 | 0.991773 |
| ENST00000457746 | PLTP | 0.001864 | 0.998136 |
| ENST00000457746 | PLCH2 | 0.004215 | 0.995785 |
| ENST00000457746 | CATG00000038465.1 | 0.002226 | 0.997774 |
| ENST00000457746 | TSFM | 0.005144 | -0.99486 |
| NR_126580 | LGALS1 | 0.007164 | -0.99284 |
| NR_126580 | TCF4 | 0.007501 | 0.992499 |
| NR_126580 | DUSP2 | 0.001967 | -0.99803 |
| ENST00000419286 | SLC13A4 | 0.002665 | -0.99734 |
| ENST00000419286 | TFF2 | 0.007202 | -0.9928 |
| ENST00000419286 | CATG00000039284.1 | 0.003317 | 0.996683 |
| NR_034091 | MYH7 | 0.00169 | 0.99831 |
| NR_034091 | NABP2 | 0.001832 | -0.99817 |
| NR_034091 | TCF23 | 0.003361 | 0.996639 |
| NR_034091 | FOXR1 | 0.004758 | 0.995242 |
| NR_040080 | GATA4 | 0.008861 | -0.99114 |
| NR_040080 | CYP21A2 | 0.003989 | -0.99601 |
| NR_040080 | HSDL2 | 2.23E-04 | 0.999777 |
| NR_040080 | ZNRD1 | 0.003627 | -0.99637 |
| NR_040080 | SYNGR3 | 0.006799 | -0.9932 |
| NR_040080 | EDDM3A | 0.00988 | -0.99012 |
| NR_027065 | RFC5 | 0.009051 | -0.99095 |
| NR_027065 | GATA4 | 0.006417 | -0.99358 |
| NR_027065 | CYP21A2 | 0.008185 | -0.99182 |
| NR_027065 | HSDL2 | 6.60E-04 | 0.99934 |
| NR_027065 | ZNRD1 | 0.007686 | -0.99231 |
| ENST00000432703 | SLC38A10 | 0.008175 | 0.991825 |
| ENST00000432703 | PLEKHD1 | 0.006246 | -0.99375 |
| ENST00000432703 | MLLT1 | 0.005551 | 0.994449 |
| ENST00000432703 | GPCPD1 | 0.002881 | -0.99712 |
| ENST00000432703 | STMND1 | 0.001909 | 0.998091 |
| ENST00000432703 | LYPD8 | 0.004361 | 0.995639 |
| ENST00000432703 | DRC1 | 0.008486 | 0.991514 |
| ENST00000432703 | ZFYVE26 | 0.009142 | 0.990858 |
| NR_040059 | RHEB | 1.97E-04 | -0.9998 |
| NR_040059 | CATG00000060074.1 | 0.009287 | -0.99071 |
| NR_040059 | VWA5A | 0.009262 | -0.99074 |
| NR_040059 | UFC1 | 6.61E-04 | -0.99934 |
| NR_040059 | JCAD | 0.005804 | 0.994196 |
| NR_040059 | TREM1 | 0.00789 | 0.99211 |
| NR_040059 | PLA1A | 0.003696 | 0.996304 |
| NR_040059 | ANKS4B | 0.004212 | 0.995788 |
| NR_130134 | RECK | 0.002514 | -0.99749 |
| NR_130134 | EDC3 | 4.91E-04 | -0.99951 |
| NR_130134 | CATG00000035925.1 | 0.007307 | -0.99269 |
| NR_130134 | MYL3 | 0.001368 | -0.99863 |
| NR_130134 | PCP4 | 0.004664 | -0.99534 |
| NR_130134 | NFE2L2 | 0.009752 | 0.990248 |
| NR_130134 | NPDC1 | 1.09E-04 | -0.99989 |
| NR_130134 | CCDC149 | 0.009268 | -0.99073 |
| NR_130134 | DNAJB13 | 0.007459 | -0.99254 |
| NR_130134 | OSBPL5 | 3.36E-04 | -0.99966 |
| NR_130134 | PCNX3 | 0.005738 | 0.994262 |
| NR_130134 | CORO7-PAM16 | 0.00922 | 0.99078 |
| NR_130134 | BTG1 | 0.00331 | 0.99669 |
| NR_130134 | ZNF107 | 0.008847 | 0.991153 |
| NR_130134 | BICRA | 0.001033 | -0.99897 |
| NR_024055 | AHNAK2 | 0.007179 | 0.992821 |
| NR_024055 | C16orf45 | 0.002324 | 0.997676 |
| NR_024055 | NCCRP1 | 3.51E-04 | 0.999649 |
| NR_024055 | HOMER3 | 0.006874 | -0.99313 |
| NR_024055 | MYO7A | 0.005253 | -0.99475 |
| NR_024055 | ARPC4-TTLL3 | 0.002382 | -0.99762 |
| NR_024055 | MAMLD1 | 8.94E-05 | -0.99991 |
| NR_024055 | ABHD5 | 0.004599 | -0.9954 |
| NR_024055 | ZNF214 | 0.002958 | -0.99704 |
| NR_024055 | OCSTAMP | 0.004865 | -0.99514 |
| NR_024055 | SP140 | 0.004704 | 0.995296 |
| ENST00000583490 | CATG00000038628.1 | 0.006166 | 0.993834 |
| ENST00000583490 | RAB5B | 0.006684 | -0.99332 |
| ENST00000533740 | INPP5J | 9.61E-05 | 0.999904 |
| ENST00000533740 | RTN1 | 0.001224 | -0.99878 |
| ENST00000533740 | CNEP1R1 | 0.006625 | -0.99337 |
| ENST00000533740 | NAGLU | 0.005514 | 0.994486 |
| ENST00000533740 | FRG1 | 0.005585 | -0.99442 |
| ENST00000533740 | AP3M2 | 0.003952 | -0.99605 |
| ENST00000533740 | BMPER | 4.03E-04 | 0.999597 |
| ENST00000596587 | SCAMP2 | 0.004657 | 0.995343 |
| ENST00000596587 | RASSF6 | 0.00447 | 0.99553 |
| ENST00000596587 | PCBP4 | 0.005291 | -0.99471 |
| ENST00000596587 | PEX14 | 0.002464 | 0.997536 |
| ENST00000596587 | XYLT2 | 0.003019 | 0.996981 |
| ENST00000596587 | ZNF713 | 0.003329 | 0.996671 |
| ENST00000596587 | ABR | 0.003868 | 0.996132 |
| ENST00000596587 | KCTD1 | 0.00472 | 0.99528 |
| ENST00000596587 | CATG00000047316.1 | 0.009459 | 0.990541 |
| ENST00000596587 | NTF3 | 0.005399 | 0.994601 |
| ENST00000596587 | RTL8B | 0.004591 | -0.99541 |
| ENST00000596587 | ASB14 | 0.00846 | -0.99154 |
| ENST00000596587 | ERVW-1 | 0.006 | -0.994 |
| ENST00000596587 | CATG00000117842.1 | 0.005706 | 0.994294 |
| ENST00000596587 | DICER1 | 0.006697 | -0.9933 |
| ENST00000596587 | C16orf86 | 0.006201 | -0.9938 |
| ENST00000500662 | CATG00000101330.1 | 0.003314 | -0.99669 |
| ENST00000500662 | CNEP1R1 | 0.001507 | -0.99849 |
| ENST00000500662 | FRG1 | 0.007322 | -0.99268 |
| ENST00000500662 | ARHGEF26 | 0.004525 | 0.995475 |
| ENST00000500662 | PHKA1 | 0.001076 | 0.998924 |
| ENST00000500662 | CATG00000026557.1 | 0.005283 | 0.994717 |
| ENST00000500662 | COA1 | 0.001107 | -0.99889 |
| ENST00000500662 | PSME1 | 0.006295 | -0.9937 |
| ENST00000500662 | DOCK1 | 0.006061 | 0.993939 |
| ENST00000500662 | BMPER | 0.008725 | 0.991275 |
| ENST00000500662 | VPS13A | 0.007757 | 0.992243 |
| ENST00000625350 | SYBU | 0.002299 | -0.9977 |
| ENST00000625350 | EXOC2 | 0.001294 | 0.998706 |
| ENST00000625350 | CHSY3 | 0.004435 | 0.995565 |
| ENST00000625350 | GLIPR1L2 | 0.001574 | 0.998426 |
| ENST00000625350 | IKBKE | 0.001097 | 0.998903 |
| ENST00000625350 | KIF21B | 0.0016 | -0.9984 |
| ENST00000625350 | CATG00000113928.1 | 0.009798 | -0.9902 |
| ENST00000625350 | KCNK16 | 0.001287 | -0.99871 |
| ENST00000625350 | MMP24 | 0.009541 | -0.99046 |
| ENST00000625350 | FANCE | 7.53E-04 | -0.99925 |
| ENST00000625350 | CLRN1 | 0.005328 | -0.99467 |
| ENST00000476980 | NAAA | 0.007093 | 0.992907 |
| ENST00000476980 | RFC5 | 0.00739 | -0.99261 |
| ENST00000476980 | CMBL | 0.007388 | -0.99261 |
| ENST00000476980 | DARS | 0.001732 | 0.998268 |
| ENST00000476980 | CATG00000038628.1 | 0.00113 | -0.99887 |
| ENST00000476980 | OR5T2 | 0.001606 | 0.998394 |
| ENST00000446543 | LDLRAD4 | 0.004991 | -0.99501 |
| ENST00000446543 | SHANK2 | 0.008235 | 0.991765 |
| ENST00000446543 | GSTM5 | 0.007757 | 0.992243 |
| ENST00000446543 | IFT80 | 0.007454 | -0.99255 |
| ENST00000446543 | KHDRBS3 | 0.002407 | -0.99759 |
| ENST00000446543 | PGAM5 | 0.007973 | 0.992027 |
| NR_109846 | CATG00000063531.1 | 0.008803 | 0.991197 |
| NR_109846 | SCAMP2 | 0.005809 | 0.994191 |
| NR_109846 | UBE2V1 | 0.009979 | 0.990021 |
| NR_109846 | RASSF6 | 0.004755 | 0.995245 |
| NR_109846 | PCBP4 | 0.004214 | -0.99579 |
| NR_109846 | PEX14 | 0.003473 | 0.996527 |
| NR_109846 | XYLT2 | 0.00404 | 0.99596 |
| NR_109846 | ZNF713 | 0.00426 | 0.99574 |
| NR_109846 | ABR | 0.003764 | 0.996236 |
| NR_109846 | KCTD1 | 0.00604 | 0.99396 |
| NR_109846 | NTF3 | 0.005471 | 0.994529 |
| NR_109846 | RTL8B | 0.003426 | -0.99657 |
| NR_109846 | ASB14 | 0.007584 | -0.99242 |
| NR_109846 | ERVW-1 | 0.004734 | -0.99527 |
| NR_109846 | CATG00000117842.1 | 0.004478 | 0.995522 |
| NR_109846 | DICER1 | 0.008414 | -0.99159 |
| NR_109846 | C16orf86 | 0.004847 | -0.99515 |
| ENST00000369123 | RPS15 | 6.31E-05 | -0.99994 |
| ENST00000369123 | ARHGAP8 | 0.005612 | -0.99439 |
| ENST00000369123 | IL31RA | 0.008259 | -0.99174 |
| ENST00000369123 | SHISA6 | 0.005915 | 0.994085 |
| ENST00000369123 | CTNNBIP1 | 0.008451 | -0.99155 |
| ENST00000520300 | TMEM132B | 0.001165 | 0.998835 |
| ENST00000520300 | EEF1D | 0.00843 | -0.99157 |
| ENST00000520300 | SEC14L6 | 0.007381 | 0.992619 |
| ENST00000520300 | IL4I1 | 7.63E-05 | -0.99992 |
| ENST00000520300 | TBKBP1 | 0.003282 | -0.99672 |
| ENST00000520300 | WT1 | 0.003416 | -0.99658 |
| ENST00000520300 | SCAMP3 | 0.003862 | -0.99614 |
| ENST00000520300 | PDE3B | 0.001127 | -0.99887 |
| ENST00000579945 | PFDN6 | 0.004702 | -0.9953 |
| ENST00000579945 | ELOF1 | 0.001074 | 0.998926 |
| ENST00000579945 | RPL23A | 0.00958 | -0.99042 |
| ENST00000579945 | IMPA2 | 0.007509 | -0.99249 |
| ENST00000579945 | GGCT | 3.65E-04 | -0.99963 |
| ENST00000579945 | TMEM242 | 7.76E-04 | -0.99922 |
| ENST00000579945 | DGAT2L6 | 0.00783 | 0.99217 |
| ENST00000579945 | DHODH | 0.004545 | -0.99546 |
| ENST00000579945 | PRPF8 | 0.002959 | -0.99704 |
| ENST00000579945 | UNC45A | 0.004559 | 0.995441 |
| ENST00000579945 | CCDC149 | 0.005485 | 0.994515 |
| ENST00000579945 | THOC5 | 2.53E-04 | 0.999747 |
| ENST00000579945 | TUBGCP4 | 0.002318 | 0.997682 |
| ENST00000579945 | CHCHD6 | 9.56E-04 | 0.999044 |
| ENST00000579945 | TMEM51 | 0.003988 | 0.996012 |
| ENST00000579945 | CEP290 | 0.003404 | 0.996596 |
| ENST00000579945 | TIGD3 | 0.009335 | -0.99067 |
| ENST00000579945 | JDP2 | 0.001845 | 0.998155 |
| ENST00000579945 | MCM3AP | 6.66E-04 | 0.999334 |
| ENST00000579945 | RPUSD2 | 0.008966 | -0.99103 |
| ENST00000579945 | ZNF107 | 0.007038 | -0.99296 |
| ENST00000579945 | CEP63 | 0.004246 | -0.99575 |
| ENST00000426428 | LRMDA | 1.58E-04 | 0.999842 |
| ENST00000426428 | RETSAT | 0.007594 | 0.992406 |
| ENST00000426428 | PHF13 | 0.003323 | -0.99668 |
| ENST00000426428 | YES1 | 0.001468 | -0.99853 |
| ENST00000426428 | CTSL | 0.001132 | 0.998868 |
| ENST00000426428 | CATG00000051841.1 | 0.008629 | -0.99137 |
| ENST00000426428 | VMP1 | 0.003615 | -0.99638 |
| ENST00000426428 | GDF2 | 0.001394 | -0.99861 |
| ENST00000418995 | ZNF227 | 0.001381 | -0.99862 |
| ENST00000418995 | CATG00000086563.1 | 0.009955 | -0.99004 |
| ENST00000418995 | DKK4 | 0.007758 | -0.99224 |
| ENST00000418995 | NAT8 | 0.007955 | -0.99204 |
| ENST00000418995 | PLA2G4F | 7.84E-04 | -0.99922 |
| ENST00000558309 | DNAJC6 | 0.008826 | 0.991174 |
| ENST00000558309 | MFAP3L | 0.004335 | 0.995665 |
| ENST00000558309 | CATG00000086946.1 | 0.004856 | 0.995144 |
| ENST00000558309 | CATG00000039284.1 | 0.009046 | -0.99095 |
| ENST00000558309 | ORM1 | 6.22E-04 | 0.999378 |
| ENST00000585659 | MRPL24 | 0.001037 | -0.99896 |
| ENST00000585659 | FAM198B | 0.00883 | -0.99117 |
| ENST00000585659 | DCLRE1B | 0.004393 | -0.99561 |
| ENST00000585659 | EXOC3L2 | 0.00866 | -0.99134 |
| ENST00000585659 | HMOX2 | 0.007291 | 0.992709 |
| ENST00000585659 | SLC11A1 | 0.002349 | 0.997651 |
| ENST00000422831 | ZNF77 | 0.008113 | 0.991887 |
| ENST00000422831 | HILPDA | 0.008331 | -0.99167 |
| ENST00000382045 | LDLRAD4 | 7.10E-05 | -0.99993 |
| ENST00000382045 | IFT80 | 0.004904 | -0.9951 |
| ENST00000382045 | DLEC1 | 0.005906 | 0.994094 |
| ENST00000475185 | DMAC2 | 0.001276 | 0.998724 |
| ENST00000475185 | GORASP1 | 0.003967 | -0.99603 |
| ENST00000475185 | ABCB8 | 0.006786 | 0.993214 |
| ENST00000475185 | HERPUD2 | 0.004569 | -0.99543 |
| ENST00000475185 | CPLX1 | 0.007227 | -0.99277 |
| ENST00000475185 | SLC22A12 | 0.006978 | 0.993022 |
| ENST00000475185 | FCHSD2 | 0.004405 | -0.99559 |
| ENST00000475185 | ZNF579 | 0.001216 | 0.998784 |
| ENST00000475185 | OR13A1 | 0.004792 | -0.99521 |
| ENST00000475185 | PTPN23 | 0.008771 | 0.991229 |
| ENST00000475185 | ANKHD1 | 0.009612 | -0.99039 |
| ENST00000475185 | SMUG1 | 0.008781 | -0.99122 |
| ENST00000475185 | TRIM47 | 0.004695 | 0.995305 |
| ENST00000475185 | DHDH | 0.005405 | 0.994595 |
| ENST00000475185 | DCUN1D1 | 0.002605 | -0.99739 |
| ENST00000475185 | C3orf84 | 0.008223 | 0.991777 |
| ENST00000475185 | AC109583.1 | 0.008984 | 0.991016 |
| ENST00000490412 | JCAD | 0.003534 | 0.996466 |
| ENST00000490412 | TREM1 | 0.001511 | 0.998489 |
| ENST00000490412 | ANKS4B | 0.009285 | 0.990715 |
| ENST00000490412 | CTNNBIP1 | 0.003584 | -0.99642 |
| NR_147088 | RRAS2 | 7.98E-04 | 0.999202 |
| ENST00000457336 | PARP6 | 0.002525 | 0.997475 |
| ENST00000457336 | DEFB112 | 0.006419 | -0.99358 |
| ENST00000457336 | GPR153 | 0.009636 | 0.990364 |
| ENST00000457336 | PLAG1 | 4.83E-04 | -0.99952 |
| ENST00000457336 | VNN3 | 0.005616 | 0.994384 |
| ENST00000426161 | PZP | 0.004456 | -0.99554 |
| ENST00000426161 | CFAP77 | 0.008008 | -0.99199 |
| ENST00000426161 | CLEC4D | 0.003046 | -0.99695 |
| ENST00000426161 | CLRN2 | 0.002727 | -0.99727 |
| ENST00000419679 | INPP5J | 0.002518 | 0.997482 |
| ENST00000419679 | RTN1 | 0.00255 | -0.99745 |
| ENST00000419679 | NAGLU | 0.001394 | 0.998606 |
| ENST00000419679 | FRG1 | 0.003989 | -0.99601 |
| ENST00000419679 | CLK3 | 0.003794 | 0.996206 |
| ENST00000419679 | AP3M2 | 0.004312 | -0.99569 |
| ENST00000419679 | BMPER | 0.002966 | 0.997034 |
| ENST00000420850 | NR2F2 | 0.007699 | 0.992301 |
| ENST00000420850 | FCN2 | 0.006609 | 0.993391 |
| ENST00000420850 | GBA | 0.002668 | 0.997332 |
| ENST00000420850 | SMPD1 | 0.005933 | 0.994067 |
| ENST00000420850 | SIVA1 | 0.006495 | -0.9935 |
| ENST00000420850 | CATG00000057824.1 | 0.009424 | 0.990576 |
| ENST00000420850 | PCNX3 | 0.005055 | -0.99494 |
| ENST00000420850 | CDK11B | 0.001256 | -0.99874 |
| ENST00000420850 | TCTEX1D4 | 9.66E-05 | -0.9999 |
| ENST00000420850 | TIGD3 | 0.006791 | -0.99321 |
| ENST00000420850 | MAP1LC3A | 0.002965 | 0.997035 |
| ENST00000420850 | LBP | 0.009832 | 0.990168 |
| ENST00000420850 | SH3RF3 | 0.001502 | -0.9985 |
| ENST00000420850 | ACMSD | 5.67E-04 | -0.99943 |
| ENST00000578800 | INTS3 | 0.007847 | -0.99215 |
| ENST00000578800 | AL358113.1 | 0.006164 | -0.99384 |
| ENST00000578800 | VPS41 | 0.009852 | -0.99015 |
| ENST00000578800 | TMEM43 | 0.005453 | 0.994547 |
| ENST00000448513 | RIPK1 | 6.28E-04 | 0.999372 |
| ENST00000448513 | NAPA | 9.28E-05 | -0.99991 |
| ENST00000448513 | PPP1CB | 0.005952 | -0.99405 |
| ENST00000448513 | P3H3 | 0.004432 | 0.995568 |
| ENST00000448513 | HDHD5 | 0.00264 | 0.99736 |
| ENST00000448513 | PTGER1 | 8.13E-04 | 0.999187 |
| ENST00000448513 | POP1 | 0.008377 | -0.99162 |
| ENST00000448513 | CATG00000110054.1 | 7.16E-04 | -0.99928 |
| ENST00000448513 | TMEM230 | 0.009934 | -0.99007 |
| ENST00000448513 | YY2 | 6.06E-04 | 0.999394 |
| ENST00000448513 | KIAA1211L | 0.002655 | 0.997345 |
| ENST00000448513 | UBN2 | 0.004157 | 0.995843 |
| ENST00000448513 | CATG00000020284.1 | 0.003769 | 0.996231 |
| ENST00000448513 | CATG00000020281.1 | 0.002474 | 0.997526 |
| ENST00000573042 | CFAP77 | 0.002963 | 0.997037 |
| ENST00000573042 | ACER1 | 0.009417 | -0.99058 |
| ENST00000463021 | ZFAND5 | 0.003966 | -0.99603 |
| ENST00000463021 | RTN1 | 0.006626 | 0.993374 |
| ENST00000463021 | NAGLU | 0.003931 | -0.99607 |
| ENST00000463021 | TP53I13 | 0.007277 | -0.99272 |
| ENST00000463021 | KRBA2 | 2.56E-04 | -0.99974 |
| ENST00000463021 | AP3M2 | 0.003116 | 0.996884 |
| ENST00000463021 | CREBRF | 0.0092 | 0.9908 |
| ENST00000521965 | NR2F2 | 0.002508 | 0.997492 |
| ENST00000521965 | GBA | 0.006606 | 0.993394 |
| ENST00000521965 | RPL23A | 0.006653 | -0.99335 |
| ENST00000521965 | SMPD1 | 0.003881 | 0.996119 |
| ENST00000521965 | DHODH | 0.003258 | -0.99674 |
| ENST00000521965 | PRPF8 | 0.005833 | -0.99417 |
| ENST00000521965 | CCDC149 | 0.006661 | 0.993339 |
| ENST00000521965 | TMEM51 | 0.009346 | 0.990654 |
| ENST00000521965 | CEP290 | 0.004289 | 0.995711 |
| ENST00000521965 | PCNX3 | 0.002113 | -0.99789 |
| ENST00000521965 | WNT2 | 0.009287 | 0.990713 |
| ENST00000521965 | CDK11B | 0.006243 | -0.99376 |
| ENST00000521965 | TCTEX1D4 | 0.002756 | -0.99724 |
| ENST00000521965 | TIGD3 | 8.12E-04 | -0.99919 |
| ENST00000521965 | MAP1LC3A | 0.005981 | 0.994019 |
| ENST00000521965 | LBP | 0.006695 | 0.993305 |
| ENST00000521965 | SH3RF3 | 0.008804 | -0.9912 |
| ENST00000521965 | ZNF107 | 0.003862 | -0.99614 |
| ENST00000521965 | REG4 | 0.009172 | -0.99083 |
| ENST00000521965 | ACMSD | 0.001496 | -0.9985 |
| NR_145493 | CATG00000060074.1 | 0.001898 | -0.9981 |
| NR_145493 | UFC1 | 0.005928 | -0.99407 |
| NR_145493 | JCAD | 0.008955 | 0.991045 |
| NR_145493 | TREM1 | 0.006668 | 0.993332 |
| NR_145493 | ANKS4B | 9.78E-04 | 0.999022 |
| ENST00000521257 | GNL1 | 0.007585 | -0.99242 |
| ENST00000521257 | POLR2F | 0.00763 | -0.99237 |
| ENST00000521257 | GPR25 | 0.003541 | 0.996459 |
| ENST00000521257 | SHC2 | 0.009508 | 0.990492 |
| ENST00000521257 | EFEMP2 | 0.001886 | -0.99811 |
| ENST00000521257 | HIST1H4E | 0.004875 | 0.995125 |
| ENST00000521257 | OR4X1 | 0.005604 | 0.994396 |
| ENST00000521257 | HLA-F | 0.002451 | 0.997549 |
| ENST00000521257 | OR10H2 | 0.003305 | 0.996695 |
| ENST00000521257 | CATG00000024701.1 | 0.009882 | 0.990118 |
| NR_110899 | TMEM176B | 0.004631 | 0.995369 |
| NR_110899 | MINK1 | 7.50E-04 | -0.99925 |
| NR_110899 | ST3GAL2 | 0.005659 | -0.99434 |
| NR_110899 | KMT5C | 0.003478 | -0.99652 |
| NR_110899 | CATG00000022091.1 | 0.001755 | -0.99825 |
| NR_110899 | GSC | 0.00788 | 0.99212 |
| NR_110899 | CATG00000096017.1 | 8.71E-04 | 0.999129 |
| ENST00000530802 | NOC4L | 0.004351 | -0.99565 |
| ENST00000530802 | SLC24A5 | 0.002996 | 0.997004 |
| ENST00000530802 | TCOF1 | 0.001794 | -0.99821 |
| ENST00000530802 | TMC5 | 0.007361 | -0.99264 |
| ENST00000530802 | SAMD13 | 0.002941 | -0.99706 |
| ENST00000530802 | ZDHHC16 | 0.003511 | 0.996489 |
| ENST00000530802 | MACROD2 | 0.001373 | -0.99863 |
| ENST00000530802 | PURG | 0.003753 | -0.99625 |
| ENST00000530802 | CACNG6 | 0.005039 | -0.99496 |
| ENST00000530802 | CHST12 | 0.005003 | -0.995 |
| ENST00000530802 | RAMP1 | 0.00894 | 0.99106 |
| ENST00000530802 | PARVG | 0.008087 | -0.99191 |
| ENST00000530802 | PCDHB8 | 0.005003 | -0.995 |
| ENST00000456273 | DNASE1L2 | 0.009541 | 0.990459 |
| ENST00000456273 | PIK3R6 | 0.005856 | 0.994144 |
| ENST00000456273 | CATG00000002970.1 | 0.006916 | 0.993084 |
| ENST00000456273 | CDKL2 | 0.006741 | 0.993259 |
| ENST00000456273 | CR749689 | 9.90E-04 | -0.99901 |
| ENST00000456273 | CRYM | 0.008288 | -0.99171 |
| ENST00000521946 | MYT1L | 0.004997 | 0.995003 |
| ENST00000521946 | TREML2 | 6.97E-04 | 0.999303 |
| ENST00000521946 | AC093157.1 | 0.008426 | -0.99157 |
| ENST00000521946 | ZPR1 | 0.008129 | 0.991871 |
| ENST00000521946 | FRAT1 | 0.001007 | -0.99899 |
| NR_146473 | LDLRAD4 | 0.006962 | 0.993038 |
| NR_146473 | GSTM5 | 5.23E-04 | -0.99948 |
| NR_146473 | ASB11 | 0.005573 | 0.994427 |
| NR_146473 | ZNRD1 | 0.008428 | -0.99157 |
| NR_146473 | PGAM5 | 0.007246 | -0.99275 |
| ENST00000417262 | PFDN6 | 0.001084 | -0.99892 |
| ENST00000417262 | ELOF1 | 0.007845 | 0.992155 |
| ENST00000417262 | CLEC1A | 0.001164 | -0.99884 |
| ENST00000417262 | IMPA2 | 0.001094 | -0.99891 |
| ENST00000417262 | GGCT | 0.00673 | -0.99327 |
| ENST00000417262 | FNDC10 | 0.002174 | 0.997826 |
| ENST00000417262 | UNC45A | 0.009364 | 0.990636 |
| ENST00000417262 | THOC5 | 0.009371 | 0.990629 |
| ENST00000417262 | PNPLA1 | 0.007175 | 0.992825 |
| ENST00000417262 | IDH1 | 0.004537 | -0.99546 |
| ENST00000417262 | TUBGCP4 | 0.003935 | 0.996065 |
| ENST00000417262 | PPP5D1 | 0.00547 | 0.99453 |
| ENST00000417262 | JDP2 | 0.005306 | 0.994694 |
| ENST00000417262 | TAF8 | 3.27E-04 | -0.99967 |
| ENST00000417262 | OR6F1 | 0.009316 | 0.990684 |
| ENST00000417262 | PSMA1 | 0.001443 | -0.99856 |
| ENST00000417262 | MCM3AP | 0.00563 | 0.99437 |
| ENST00000417262 | USP9X | 0.007548 | 0.992452 |
| ENST00000445551 | IL31RA | 0.008283 | -0.99172 |
| ENST00000445551 | NHEJ1 | 0.008957 | -0.99104 |
| ENST00000445551 | EHD4 | 0.009368 | 0.990632 |
| ENST00000445551 | PRKDC | 0.007931 | 0.992069 |
| ENST00000445551 | USF2 | 0.006413 | 0.993587 |
| ENST00000445551 | SMAP2 | 5.38E-05 | 0.999946 |
| ENST00000445551 | ISG15 | 0.00337 | -0.99663 |
| ENST00000607166 | MYD88 | 0.00683 | 0.99317 |
| ENST00000607166 | FSCB | 0.003023 | -0.99698 |
| ENST00000607166 | PPP5C | 0.008383 | -0.99162 |
| ENST00000607166 | IGSF9B | 0.008194 | -0.99181 |
| ENST00000607166 | IKZF4 | 0.008256 | -0.99174 |
| ENST00000607166 | MYH3 | 0.002406 | 0.997594 |
| ENST00000607166 | PCDHGA3 | 0.003646 | -0.99635 |
| ENST00000607166 | DCD | 0.00614 | -0.99386 |
| ENST00000607166 | PAN3 | 9.81E-04 | 0.999019 |
| ENST00000607166 | ZNF587B | 0.008151 | 0.991849 |
| ENST00000607166 | INKA2 | 0.007651 | -0.99235 |
| ENST00000607166 | YPEL5 | 0.0026 | -0.9974 |
| ENST00000424933 | INPP5J | 0.004527 | -0.99547 |
| ENST00000424933 | RFC2 | 0.004769 | 0.995231 |
| ENST00000424933 | RTN1 | 0.008092 | 0.991908 |
| ENST00000424933 | EXOC4 | 0.008128 | -0.99187 |
| ENST00000424933 | CNEP1R1 | 0.004245 | 0.995755 |
| ENST00000424933 | DBI | 0.006264 | 0.993736 |
| ENST00000424933 | TP73 | 0.002384 | -0.99762 |
| ENST00000424933 | CATG00000026557.1 | 0.009669 | -0.99033 |
| ENST00000424933 | DOCK1 | 0.005781 | -0.99422 |
| ENST00000424933 | BMPER | 0.004415 | -0.99558 |
| ENST00000424933 | DEFA6 | 0.002854 | -0.99715 |
| ENST00000424933 | CATG00000027020.1 | 0.008366 | -0.99163 |
| NR_034104 | AHNAK2 | 1.80E-04 | -0.99982 |
| NR_034104 | C16orf45 | 0.007709 | -0.99229 |
| NR_034104 | NCCRP1 | 0.005122 | -0.99488 |
| NR_034104 | HOMER3 | 2.16E-04 | 0.999784 |
| NR_034104 | TNK2 | 0.009502 | -0.9905 |
| NR_034104 | ARPC4-TTLL3 | 0.002512 | 0.997488 |
| NR_034104 | MAMLD1 | 0.005551 | 0.994449 |
| NR_034104 | ZNF214 | 0.003205 | 0.996795 |
| NR_034104 | ZNF624 | 0.009852 | -0.99015 |
| ENST00000425470 | DNAJC6 | 0.007595 | 0.992405 |
| ENST00000425470 | MFAP3L | 0.005575 | 0.994425 |
| ENST00000425470 | SPACA9 | 0.007938 | 0.992062 |
| ENST00000425470 | ORM1 | 8.86E-04 | 0.999114 |
| ENST00000529392 | RIPK1 | 0.004812 | -0.99519 |
| ENST00000529392 | NAPA | 0.004804 | 0.995196 |
| ENST00000529392 | PPP1CB | 0.003808 | 0.996192 |
| ENST00000529392 | PTGER1 | 0.001597 | -0.9984 |
| ENST00000529392 | CATG00000107158.1 | 0.007114 | 0.992886 |
| ENST00000529392 | CATG00000110054.1 | 0.003839 | 0.996161 |
| ENST00000529392 | TMEM230 | 0.003515 | 0.996485 |
| ENST00000529392 | YY2 | 0.00708 | -0.99292 |
| ENST00000529392 | RCSD1 | 0.004575 | -0.99542 |
| ENST00000529392 | KIAA1211L | 0.002585 | -0.99741 |
| ENST00000529392 | CATG00000020281.1 | 0.003373 | -0.99663 |
| ENST00000502809 | RETSAT | 0.00334 | 0.99666 |
| ENST00000502809 | C20orf203 | 0.004154 | 0.995846 |
| ENST00000502809 | MYO7A | 0.007643 | -0.99236 |
| ENST00000502809 | MAMLD1 | 0.009059 | -0.99094 |
| ENST00000502809 | ABHD5 | 0.003499 | -0.9965 |
| ENST00000502809 | FAM110C | 0.004664 | -0.99534 |
| ENST00000502809 | SP140 | 0.004513 | 0.995487 |
| ENST00000630421 | NFATC2 | 0.006588 | 0.993412 |
| ENST00000630421 | MINK1 | 0.008206 | -0.99179 |
| ENST00000630421 | CFAP77 | 0.002878 | 0.997122 |
| ENST00000630421 | ACER1 | 0.002995 | -0.997 |
| ENST00000412084 | CMBL | 0.00879 | -0.99121 |
| ENST00000412084 | DARS | 0.004357 | 0.995643 |
| ENST00000412084 | CATG00000038628.1 | 7.29E-04 | -0.99927 |
| ENST00000412084 | OR5T2 | 2.18E-04 | 0.999782 |
| ENST00000530902 | TAS2R42 | 0.004041 | 0.995959 |
| ENST00000530902 | B3GNT3 | 0.007092 | -0.99291 |
| ENST00000530902 | GRINA | 0.003243 | 0.996757 |
| ENST00000530902 | HMGB2 | 0.005939 | -0.99406 |
| ENST00000530902 | PSRC1 | 0.00564 | -0.99436 |
| ENST00000530902 | PLIN4 | 5.55E-04 | 0.999445 |
| ENST00000530902 | LILRB1 | 0.003168 | -0.99683 |
| ENST00000530902 | RASL10A | 0.004841 | -0.99516 |
| ENST00000530902 | FOXO6 | 0.009692 | 0.990308 |
| ENST00000530902 | CXXC1 | 0.002405 | -0.9976 |
| ENST00000530902 | CATG00000053512.1 | 0.008756 | -0.99124 |
| ENST00000530902 | HLF | 0.005144 | 0.994856 |
| ENST00000530902 | SRRD | 0.006708 | 0.993292 |
| ENST00000530902 | TPTE | 0.006929 | 0.993071 |
| ENST00000530902 | NFX1 | 0.003442 | -0.99656 |
| ENST00000530902 | CATG00000012021.1 | 0.008303 | -0.9917 |
| ENST00000530902 | FYB2 | 0.003022 | 0.996978 |
| ENST00000530902 | PEA15 | 0.003287 | 0.996713 |
| ENST00000530902 | ESRRB | 0.004755 | 0.995245 |
| ENST00000530902 | IFT122 | 0.003609 | 0.996391 |
| ENST00000530902 | SLC12A3 | 0.004294 | -0.99571 |
| ENST00000530902 | SIGLEC7 | 0.006945 | 0.993055 |
| ENST00000530902 | WNT8B | 0.001791 | 0.998209 |
| ENST00000530902 | UNC5C | 0.002343 | -0.99766 |
| ENST00000530902 | OXT | 0.006096 | 0.993904 |
| ENST00000530902 | FAM174A | 0.003706 | -0.99629 |
| ENST00000530902 | CFAP410 | 0.003474 | 0.996526 |
| ENST00000530902 | HPS1 | 8.55E-04 | 0.999145 |
| ENST00000530902 | CCDC174 | 0.005691 | -0.99431 |
| ENST00000530902 | FAM151A | 0.004751 | 0.995249 |
| ENST00000597160 | ZFAND5 | 0.003703 | 0.996297 |
| ENST00000597160 | RTN1 | 0.006686 | -0.99331 |
| ENST00000597160 | NAGLU | 0.003682 | 0.996318 |
| ENST00000597160 | TP53I13 | 0.007198 | 0.992802 |
| ENST00000597160 | KRBA2 | 2.85E-04 | 0.999715 |
| ENST00000597160 | AP3M2 | 0.003222 | -0.99678 |
| ENST00000597160 | CREBRF | 0.00879 | -0.99121 |
| ENST00000597160 | ZNF664 | 0.009813 | -0.99019 |
| ENST00000597160 | CIB1 | 0.009585 | -0.99041 |
| ENST00000447982 | VPS41 | 0.007248 | -0.99275 |
| ENST00000504969 | BARHL1 | 0.007662 | -0.99234 |
| ENST00000504969 | B4GALNT1 | 5.06E-04 | -0.99949 |
| ENST00000504969 | S1PR5 | 0.001251 | -0.99875 |
| ENST00000504969 | RP1L1 | 0.003228 | -0.99677 |
| ENST00000504969 | DEFA1 | 0.003888 | 0.996112 |
| ENST00000504969 | PLEKHG3 | 0.001405 | -0.99859 |
| ENST00000504969 | DEFA4 | 0.004298 | 0.995702 |
| ENST00000461249 | DNAJC6 | 0.003733 | 0.996267 |
| ENST00000461249 | MFAP3L | 0.002333 | 0.997667 |
| ENST00000461249 | CATG00000086946.1 | 0.006211 | 0.993789 |
| ENST00000461249 | SPACA9 | 0.004203 | 0.995797 |
| ENST00000461249 | ORM1 | 4.76E-04 | 0.999524 |
| ENST00000629293 | WNT16 | 0.001271 | 0.998729 |
| ENST00000629293 | PCP4 | 0.00309 | 0.99691 |
| ENST00000629293 | NLRP9 | 0.00192 | 0.99808 |
| ENST00000629293 | IRX3 | 0.008489 | 0.991511 |
| ENST00000629293 | CD180 | 0.005278 | -0.99472 |
| ENST00000629293 | DNAJB13 | 0.004737 | 0.995263 |
| ENST00000629293 | COBL | 0.003948 | 0.996052 |
| ENST00000629293 | BTG1 | 0.007677 | -0.99232 |
| ENST00000629293 | BICRA | 0.006664 | 0.993336 |
| ENST00000629293 | PIPOX | 0.002246 | -0.99775 |
| ENST00000419746 | PLEKHA2 | 0.007406 | 0.992594 |
| ENST00000419746 | PARP6 | 0.008265 | -0.99173 |
| ENST00000419746 | PLAG1 | 0.002596 | 0.997404 |
| ENST00000419746 | VNN3 | 0.007537 | -0.99246 |
| ENST00000419746 | DYNC2H1 | 0.009452 | -0.99055 |
| ENST00000414074 | GABRG1 | 0.001257 | 0.998743 |
| ENST00000414074 | CEBPD | 0.00419 | 0.99581 |
| ENST00000436340 | SCAMP2 | 0.001379 | 0.998621 |
| ENST00000436340 | EPB41L1 | 0.005343 | 0.994657 |
| ENST00000436340 | RASSF6 | 0.007122 | 0.992878 |
| ENST00000436340 | PEX14 | 2.41E-04 | 0.999759 |
| ENST00000436340 | XYLT2 | 6.09E-04 | 0.999391 |
| ENST00000436340 | ZNF713 | 0.002249 | 0.997751 |
| ENST00000436340 | ABR | 0.00852 | 0.99148 |
| ENST00000436340 | IKZF4 | 0.007462 | 0.992538 |
| ENST00000436340 | MYH3 | 0.009191 | -0.99081 |
| ENST00000436340 | KCTD1 | 6.99E-04 | 0.999301 |
| ENST00000436340 | CATG00000047316.1 | 0.002585 | 0.997415 |
| ENST00000436340 | ZMYND15 | 0.007793 | 0.992207 |
| ENST00000436340 | NTF3 | 0.009264 | 0.990736 |
| ENST00000436340 | DNAL4 | 0.008129 | -0.99187 |
| ENST00000436340 | INKA2 | 0.003413 | 0.996587 |
| ENST00000436340 | ZNF37A | 0.003326 | -0.99667 |
| ENST00000436340 | DICER1 | 0.001084 | -0.99892 |
| ENST00000477677 | PDE4C | 0.004746 | -0.99525 |
| ENST00000477677 | RUSC2 | 0.006806 | 0.993194 |
| ENST00000477677 | CDA | 0.0083 | 0.9917 |
| ENST00000430592 | TMEM255B | 0.005999 | -0.994 |
| ENST00000430592 | POMT2 | 6.09E-04 | 0.999391 |
| ENST00000430592 | INHBA | 0.0072 | 0.9928 |
| ENST00000430592 | EIF2AK2 | 0.008097 | -0.9919 |
| ENST00000430592 | PPP3R2 | 0.001045 | 0.998955 |
| ENST00000449169 | CATG00000092654.1 | 0.001159 | -0.99884 |
| ENST00000449169 | APBB3 | 0.001513 | 0.998487 |
| ENST00000449169 | CHSY3 | 0.009027 | -0.99097 |
| ENST00000449169 | GLIPR1L2 | 0.006502 | -0.9935 |
| ENST00000449169 | IKBKE | 0.008564 | -0.99144 |
| ENST00000449169 | CATG00000113928.1 | 0.008129 | 0.991871 |
| ENST00000449169 | RTN4 | 0.003422 | 0.996578 |
| ENST00000449169 | KCNK16 | 0.007521 | 0.992479 |
| ENST00000449169 | MMP24 | 0.009307 | 0.990693 |
| ENST00000449169 | FANCE | 0.008708 | 0.991292 |
| ENST00000509934 | RETSAT | 0.006015 | -0.99398 |
| ENST00000509934 | FAM84A | 0.007653 | -0.99235 |
| ENST00000509934 | CAPS | 6.49E-04 | -0.99935 |
| ENST00000509934 | C20orf203 | 0.009346 | -0.99065 |
| ENST00000509934 | GMPPA | 0.006286 | -0.99371 |
| ENST00000509934 | CATG00000053936.1 | 6.74E-04 | -0.99933 |
| ENST00000509934 | G6PC3 | 0.00999 | 0.99001 |
| ENST00000509934 | ARHGEF40 | 7.40E-04 | 0.99926 |
| ENST00000509934 | CATG00000107162.1 | 0.002434 | 0.997566 |
| ENST00000509934 | FAM110C | 0.003966 | 0.996034 |
| ENST00000509934 | VMP1 | 0.007019 | 0.992981 |
| ENST00000509934 | C16orf78 | 3.36E-04 | 0.999664 |
| ENST00000509934 | GDF2 | 0.007726 | 0.992274 |
| ENST00000475393 | CEP170 | 0.009245 | -0.99076 |
| ENST00000475393 | EXOC4 | 0.002888 | 0.997112 |
| ENST00000475393 | TRMT11 | 0.007488 | -0.99251 |
| ENST00000475393 | ADGRE1 | 0.004074 | -0.99593 |
| ENST00000475393 | TMEM132B | 0.001933 | 0.998067 |
| ENST00000475393 | EEF1D | 0.008299 | -0.9917 |
| ENST00000475393 | DBI | 0.004782 | -0.99522 |
| ENST00000475393 | SEC14L6 | 3.76E-04 | 0.999624 |
| ENST00000475393 | IL4I1 | 0.005916 | -0.99408 |
| ENST00000475393 | NEMP2 | 0.003252 | 0.996748 |
| ENST00000475393 | NEFM | 0.001445 | -0.99856 |
| ENST00000475393 | TBKBP1 | 0.005245 | -0.99476 |
| ENST00000475393 | PDE3B | 0.002156 | -0.99784 |
| ENST00000475393 | DEFA6 | 0.008666 | 0.991334 |
| ENST00000475393 | CATG00000027020.1 | 0.007374 | 0.992626 |
| ENST00000416742 | TMEM255B | 0.003871 | -0.99613 |
| ENST00000416742 | ASAP1 | 0.00192 | 0.99808 |
| ENST00000416742 | SUPV3L1 | 0.007036 | -0.99296 |
| ENST00000416742 | GPR156 | 0.002938 | 0.997062 |
| ENST00000416742 | FTCD | 0.001221 | 0.998779 |
| ENST00000416742 | ZNF77 | 0.006365 | -0.99364 |
| ENST00000416742 | INHBA | 0.004467 | 0.995533 |
| ENST00000596412 | HEXIM2 | 0.00526 | -0.99474 |
| ENST00000596412 | FAR1 | 0.008682 | -0.99132 |
| ENST00000596412 | TRIM73 | 0.008853 | -0.99115 |
| ENST00000596412 | MAPK12 | 9.70E-04 | 0.99903 |
| ENST00000596412 | SLCO2A1 | 0.008067 | 0.991933 |
| ENST00000596412 | CAMP | 0.001894 | 0.998106 |
| ENST00000596412 | TCN1 | 1.80E-04 | 0.99982 |
| ENST00000596412 | DDX58 | 0.006999 | -0.993 |
| ENST00000504184 | ELOF1 | 0.007506 | 0.992494 |
| ENST00000504184 | GGCT | 0.004962 | -0.99504 |
| ENST00000504184 | PCP4 | 0.007854 | 0.992146 |
| ENST00000504184 | TMEM242 | 0.001002 | -0.999 |
| ENST00000504184 | NLRP9 | 0.007872 | 0.992128 |
| ENST00000504184 | DHODH | 0.006571 | -0.99343 |
| ENST00000504184 | PRPF8 | 0.002512 | -0.99749 |
| ENST00000504184 | UNC45A | 0.004219 | 0.995781 |
| ENST00000504184 | CCDC149 | 0.002078 | 0.997922 |
| ENST00000504184 | THOC5 | 0.002295 | 0.997705 |
| ENST00000504184 | TUBGCP4 | 0.006328 | 0.993672 |
| ENST00000504184 | RACK1 | 0.004969 | 0.995031 |
| ENST00000504184 | CHCHD6 | 5.48E-04 | 0.999452 |
| ENST00000504184 | TMEM51 | 8.19E-04 | 0.999181 |
| ENST00000504184 | CEP290 | 0.00506 | 0.99494 |
| ENST00000504184 | TIGD3 | 0.009177 | -0.99082 |
| ENST00000504184 | JDP2 | 0.009365 | 0.990635 |
| ENST00000504184 | MCM3AP | 0.00516 | 0.99484 |
| ENST00000504184 | RPUSD2 | 0.004022 | -0.99598 |
| ENST00000504184 | ZNF107 | 0.004162 | -0.99584 |
| ENST00000504184 | CEP63 | 1.46E-04 | -0.99985 |
| NR_028038 | EEF1D | 0.00418 | 0.99582 |
| NR_028038 | TBKBP1 | 0.005394 | 0.994606 |
| NR_028038 | WT1 | 0.004997 | 0.995003 |
| NR_028038 | CATG00000039284.1 | 0.001374 | 0.998626 |
| NR_028038 | SCAMP3 | 0.002864 | 0.997136 |
| ENST00000584090 | PHF20 | 0.00606 | 0.99394 |
| ENST00000584090 | MMP1 | 0.004159 | 0.995841 |
| ENST00000584090 | C15orf65 | 0.004112 | -0.99589 |
| ENST00000584090 | NTNG1 | 0.00168 | 0.99832 |
| ENST00000584090 | MROH9 | 0.005859 | -0.99414 |
| ENST00000584090 | PMEPA1 | 0.007845 | -0.99216 |
| ENST00000584090 | PMIS2 | 8.32E-04 | -0.99917 |
| ENST00000584090 | KCTD18 | 0.006455 | -0.99355 |
| ENST00000584090 | NDUFC1 | 0.008062 | 0.991938 |
| ENST00000584090 | FCRL1 | 0.004665 | -0.99534 |
| ENST00000455701 | HEXIM2 | 0.009344 | -0.99066 |
| ENST00000455701 | OCLN | 4.71E-04 | -0.99953 |
| ENST00000455701 | FAR1 | 0.001783 | -0.99822 |
| ENST00000455701 | AGAP1 | 0.005759 | 0.994241 |
| ENST00000455701 | HILPDA | 0.006628 | 0.993372 |
| ENST00000455701 | KIF27 | 0.001402 | -0.9986 |
| ENST00000419168 | TAS2R42 | 0.001388 | 0.998612 |
| ENST00000419168 | GRINA | 0.00695 | 0.99305 |
| ENST00000419168 | PPP3CC | 0.004575 | -0.99543 |
| ENST00000419168 | PSRC1 | 0.001722 | -0.99828 |
| ENST00000419168 | ABCB8 | 0.005763 | 0.994237 |
| ENST00000419168 | LILRB1 | 0.005818 | -0.99418 |
| ENST00000419168 | RASL10A | 0.00418 | -0.99582 |
| ENST00000419168 | LTA4H | 2.65E-04 | -0.99974 |
| ENST00000419168 | TUT4 | 0.002571 | -0.99743 |
| ENST00000419168 | WDR33 | 0.004883 | 0.995117 |
| ENST00000419168 | CATG00000053512.1 | 0.005341 | -0.99466 |
| ENST00000419168 | HLF | 0.007229 | 0.992771 |
| ENST00000419168 | NFX1 | 0.004016 | -0.99598 |
| ENST00000419168 | PEA15 | 0.004261 | 0.995739 |
| ENST00000419168 | SLC12A3 | 0.003622 | -0.99638 |
| ENST00000419168 | WNT8B | 0.004259 | 0.995741 |
| ENST00000419168 | UNC5C | 0.003574 | -0.99643 |
| ENST00000419168 | AGO2 | 0.00987 | 0.99013 |
| ENST00000419168 | TRIM47 | 0.007952 | 0.992048 |
| ENST00000419168 | CATG00000087047.1 | 4.29E-04 | 0.999571 |
| ENST00000419168 | AC109583.1 | 0.00958 | 0.99042 |
| ENST00000419168 | CFAP410 | 0.00515 | 0.99485 |
| ENST00000419168 | HPS1 | 0.005922 | 0.994078 |
| ENST00000419168 | RAD51AP2 | 0.001857 | -0.99814 |
| ENST00000511705 | SYNDIG1L | 0.009797 | -0.9902 |
| ENST00000511705 | CATG00000101330.1 | 0.005139 | -0.99486 |
| ENST00000511705 | CNEP1R1 | 0.00554 | -0.99446 |
| ENST00000511705 | AC092073.1 | 0.009344 | 0.990656 |
| ENST00000511705 | FRG1 | 0.007196 | -0.9928 |
| ENST00000511705 | TGFBR3 | 0.00597 | 0.99403 |
| ENST00000511705 | SLC22A6 | 0.008156 | -0.99184 |
| ENST00000511705 | ARHGEF26 | 0.008211 | 0.991789 |
| ENST00000511705 | PHKA1 | 0.004141 | 0.995859 |
| ENST00000511705 | COA1 | 5.27E-04 | -0.99947 |
| ENST00000511705 | COA5 | 0.005925 | -0.99407 |
| ENST00000511705 | SLC6A5 | 0.007449 | 0.992551 |
| ENST00000511705 | RRBP1 | 0.009604 | -0.9904 |
| ENST00000511705 | PSME1 | 0.008411 | -0.99159 |
| ENST00000511705 | MORN3 | 0.008761 | -0.99124 |
| ENST00000511705 | VPS13A | 0.002526 | 0.997474 |
| ENST00000463884 | DNAJC22 | 8.97E-04 | -0.9991 |
| ENST00000463884 | CATG00000074949.1 | 0.004629 | -0.99537 |
| ENST00000463884 | DKK4 | 0.004096 | -0.9959 |
| ENST00000463884 | OR8G1 | 0.003811 | -0.99619 |
| ENST00000463884 | TATDN3 | 0.007231 | -0.99277 |
| ENST00000463884 | KCNE4 | 0.005553 | 0.994447 |
| ENST00000464721 | NR2F2 | 0.001059 | 0.998941 |
| ENST00000464721 | GBA | 0.004922 | 0.995078 |
| ENST00000464721 | RPL23A | 0.004814 | -0.99519 |
| ENST00000464721 | SMPD1 | 0.001803 | 0.998197 |
| ENST00000464721 | DHODH | 0.003768 | -0.99623 |
| ENST00000464721 | PRPF8 | 0.007528 | -0.99247 |
| ENST00000464721 | CCDC149 | 0.009361 | 0.990639 |
| ENST00000464721 | CEP290 | 0.005073 | 0.994927 |
| ENST00000464721 | PCNX3 | 0.004364 | -0.99564 |
| ENST00000464721 | WNT2 | 0.00648 | 0.99352 |
| ENST00000464721 | CDK11B | 0.00536 | -0.99464 |
| ENST00000464721 | TCTEX1D4 | 0.003496 | -0.9965 |
| ENST00000464721 | TIGD3 | 0.001694 | -0.99831 |
| ENST00000464721 | MAP1LC3A | 0.004165 | 0.995835 |
| ENST00000464721 | LBP | 0.003811 | 0.996189 |
| ENST00000464721 | SH3RF3 | 0.008851 | -0.99115 |
| ENST00000464721 | ZNF107 | 0.006094 | -0.99391 |
| ENST00000464721 | REG4 | 0.005988 | -0.99401 |
| ENST00000464721 | ACMSD | 0.002436 | -0.99756 |
| ENST00000495094 | ACSBG1 | 0.004472 | -0.99553 |
| ENST00000495094 | PNPLA2 | 0.00686 | 0.99314 |
| ENST00000495094 | PDCD2L | 8.22E-04 | -0.99918 |
| ENST00000495094 | CATG00000108269.1 | 0.0051 | 0.9949 |
| ENST00000495094 | FOXJ3 | 0.002468 | 0.997532 |
| ENST00000495094 | CYP27C1 | 0.002746 | 0.997254 |
| ENST00000431144 | ZFAND5 | 0.008863 | -0.99114 |
| ENST00000431144 | CATG00000056264.1 | 0.006969 | -0.99303 |
| ENST00000431144 | SSBP2 | 0.007531 | -0.99247 |
| ENST00000431144 | CLEC4G | 0.006593 | -0.99341 |
| ENST00000431144 | CD27 | 0.008741 | -0.99126 |
| ENST00000431144 | TMC2 | 0.003645 | -0.99636 |
| ENST00000431144 | NAGLU | 0.002411 | -0.99759 |
| ENST00000431144 | CLK3 | 2.52E-04 | -0.99975 |
| ENST00000431144 | TPRX1 | 0.003188 | -0.99681 |
| ENST00000431144 | CLCNKB | 0.009189 | -0.99081 |
| ENST00000431144 | PPTC7 | 0.003609 | 0.996391 |
| ENST00000431144 | CIB1 | 0.001946 | 0.998054 |
| ENST00000457348 | TMEM255B | 0.007953 | 0.992047 |
| ENST00000457348 | POMT2 | 2.96E-04 | -0.9997 |
| ENST00000457348 | INHBA | 0.008549 | -0.99145 |
| ENST00000457348 | EIF2AK2 | 0.005322 | 0.994678 |
| ENST00000457348 | PPP3R2 | 2.15E-04 | -0.99978 |
| ENST00000457348 | CATG00000068640.1 | 0.009717 | -0.99028 |
| ENST00000416951 | MINK1 | 0.004773 | 0.995227 |
| ENST00000416951 | DUSP9 | 0.001077 | 0.998923 |
| ENST00000416951 | ALS2CR12 | 0.003805 | 0.996195 |
| ENST00000416951 | CATG00000096017.1 | 0.00487 | -0.99513 |
| ENST00000616469 | CATG00000038628.1 | 0.005022 | 0.994978 |
| ENST00000616469 | OR5T2 | 0.00404 | -0.99596 |
| ENST00000616469 | RAB5B | 0.005395 | -0.99461 |
| NR_149012 | TOB1 | 0.005535 | -0.99447 |
| NR_149012 | MED20 | 0.002402 | 0.997598 |
| NR_149012 | EPHB6 | 0.005498 | 0.994502 |
| NR_149012 | TSPAN32 | 0.005148 | 0.994852 |
| NR_149012 | CATG00000021838.1 | 0.00344 | -0.99656 |
| NR_149012 | METTL3 | 0.008015 | -0.99198 |
| NR_149012 | C1QTNF9B | 0.006468 | -0.99353 |
| NR_149012 | TSFM | 0.004457 | -0.99554 |
| NR_149012 | UPP2 | 0.005053 | -0.99495 |
| ENST00000555350 | CEP170 | 0.008941 | 0.991059 |
| ENST00000555350 | TRMT11 | 0.002077 | 0.997923 |
| ENST00000555350 | ADGRE1 | 0.005543 | 0.994457 |
| ENST00000555350 | MYLK3 | 0.002621 | 0.997379 |
| ENST00000555350 | SIRT6 | 0.00348 | 0.99652 |
| ENST00000555350 | SLC39A12 | 0.005287 | -0.99471 |
| ENST00000462559 | TMEM132B | 0.008294 | 0.991706 |
| ENST00000462559 | GATA4 | 0.003778 | 0.996222 |
| ENST00000462559 | IL4I1 | 0.002401 | -0.9976 |
| ENST00000462559 | WT1 | 0.006554 | -0.99345 |
| ENST00000462559 | SLC39A12 | 0.006662 | 0.993338 |
| ENST00000462559 | SYNGR3 | 0.008815 | 0.991185 |
| ENST00000462559 | PDE3B | 0.00822 | -0.99178 |
| ENST00000409912 | HMGB2 | 0.005735 | -0.99427 |
| ENST00000409912 | IGFN1 | 0.001822 | -0.99818 |
| ENST00000409912 | YWHAB | 0.001905 | 0.998095 |
| ENST00000409912 | GSTA4 | 0.001492 | 0.998508 |
| ENST00000409912 | CXXC1 | 0.00979 | -0.99021 |
| ENST00000409912 | TBX3 | 0.00814 | 0.99186 |
| ENST00000409912 | GPR161 | 0.002737 | 0.997263 |
| ENST00000409912 | EIF1AD | 0.005535 | 0.994465 |
| ENST00000409912 | KRT83 | 0.005377 | 0.994623 |
| ENST00000409912 | TPTE | 0.008073 | 0.991927 |
| ENST00000409912 | CAPRIN2 | 0.004028 | 0.995972 |
| ENST00000409912 | CLEC5A | 1.03E-04 | 0.999897 |
| ENST00000409912 | CATG00000089121.1 | 0.005572 | -0.99443 |
| ENST00000409912 | RBM7 | 0.006413 | 0.993587 |
| ENST00000409912 | CPOX | 0.004685 | 0.995315 |
| ENST00000409912 | GSG1L | 0.004223 | 0.995777 |
| ENST00000409912 | ERFE | 0.001211 | -0.99879 |
| ENST00000409912 | PAK3 | 0.005058 | 0.994942 |
| ENST00000409912 | SNRPD2 | 0.002739 | 0.997261 |
| ENST00000409912 | ZC3H12D | 0.00899 | -0.99101 |
| ENST00000409912 | LIM2 | 2.80E-04 | 0.99972 |
| ENST00000409912 | AL627171.2 | 0.009034 | -0.99097 |
| ENST00000409912 | TNFRSF13B | 0.006587 | -0.99341 |
| ENST00000409912 | CCDC174 | 0.005852 | -0.99415 |
| ENST00000527910 | GNL1 | 1.74E-04 | -0.99983 |
| ENST00000527910 | DEAF1 | 0.001862 | 0.998138 |
| ENST00000527910 | GPR25 | 0.003658 | 0.996342 |
| ENST00000527910 | VRK3 | 0.009588 | 0.990412 |
| ENST00000527910 | FANCD2OS | 0.001197 | 0.998803 |
| ENST00000527910 | CATG00000023328.1 | 0.00983 | -0.99017 |
| ENST00000527910 | EFEMP2 | 0.003227 | -0.99677 |
| ENST00000527910 | OR4X1 | 0.003378 | 0.996622 |
| ENST00000527910 | HLA-F | 0.002865 | 0.997135 |
| ENST00000527910 | CATG00000024701.1 | 0.008406 | 0.991594 |
| ENST00000527910 | ZNF431 | 0.004326 | 0.995674 |
| ENST00000465249 | SHANK2 | 0.00544 | -0.99456 |
| ENST00000465249 | B4GALNT1 | 0.004498 | 0.995502 |
| ENST00000465249 | RP1L1 | 0.001043 | 0.998957 |
| ENST00000465249 | MECP2 | 0.00792 | -0.99208 |
| ENST00000465249 | PLEKHG3 | 0.001618 | 0.998382 |
| ENST00000612281 | HEXIM2 | 0.004624 | 0.995376 |
| ENST00000612281 | OCLN | 0.008175 | 0.991825 |
| ENST00000612281 | TRIM73 | 0.002619 | 0.997381 |
| ENST00000612281 | CATG00000063086.1 | 0.003061 | -0.99694 |
| ENST00000612281 | LTF | 5.12E-05 | -0.99995 |
| ENST00000612281 | WFDC1 | 0.005694 | -0.99431 |
| ENST00000612281 | CATG00000068640.1 | 0.005678 | -0.99432 |
| ENST00000612281 | KIF27 | 0.005795 | 0.994205 |
| ENST00000445589 | WDR90 | 0.008235 | -0.99177 |
| ENST00000445589 | SORCS2 | 0.006385 | -0.99361 |
| ENST00000445589 | PPP1R16B | 9.74E-05 | -0.9999 |
| ENST00000445589 | KIF20A | 0.008305 | -0.9917 |
| ENST00000445589 | FOXR1 | 0.00928 | 0.99072 |
| NR_027076 | PLA2G15 | 0.006593 | 0.993407 |
| NR_027076 | ZNF227 | 0.007524 | 0.992476 |
| NR_027076 | RIOX2 | 0.001071 | 0.998929 |
| NR_027076 | ASGR2 | 0.001196 | 0.998804 |
| NR_027076 | OR8G1 | 0.008631 | 0.991369 |
| NR_027076 | NAT8 | 7.53E-04 | 0.999247 |
| NR_027076 | PLA1A | 0.003839 | -0.99616 |
| ENST00000554628 | RIPK1 | 0.002244 | 0.997756 |
| ENST00000554628 | NAPA | 0.006686 | -0.99331 |
| ENST00000554628 | BTBD2 | 0.00494 | -0.99506 |
| ENST00000554628 | HDHD5 | 0.002352 | 0.997648 |
| ENST00000554628 | PTGER1 | 0.004722 | 0.995278 |
| ENST00000554628 | POP1 | 3.83E-04 | -0.99962 |
| ENST00000554628 | CATG00000110054.1 | 0.009538 | -0.99046 |
| ENST00000554628 | TMOD1 | 0.009668 | -0.99033 |
| ENST00000554628 | TMEM230 | 0.006606 | -0.99339 |
| ENST00000554628 | YY2 | 0.008616 | 0.991384 |
| ENST00000554628 | KIAA1211L | 0.002133 | 0.997867 |
| ENST00000554628 | CCNK | 0.007179 | -0.99282 |
| ENST00000554628 | TNFAIP6 | 0.008014 | -0.99199 |
| ENST00000554628 | CATG00000039609.1 | 0.007174 | 0.992826 |
| ENST00000554628 | CATG00000020284.1 | 0.003487 | 0.996513 |
| ENST00000360737 | WNT16 | 0.005775 | -0.99423 |
| ENST00000360737 | PCP4 | 0.002222 | -0.99778 |
| ENST00000360737 | TMEM242 | 0.005341 | 0.994659 |
| ENST00000360737 | NLRP9 | 0.003214 | -0.99679 |
| ENST00000360737 | PRPF8 | 0.005099 | 0.994901 |
| ENST00000360737 | UNC45A | 0.009183 | -0.99082 |
| ENST00000360737 | NPDC1 | 0.008144 | -0.99186 |
| ENST00000360737 | CCDC149 | 0.002458 | -0.99754 |
| ENST00000360737 | THOC5 | 0.008003 | -0.992 |
| ENST00000360737 | RACK1 | 0.005546 | -0.99445 |
| ENST00000360737 | CHCHD6 | 0.004118 | -0.99588 |
| ENST00000360737 | TMEM51 | 0.00175 | -0.99825 |
| ENST00000360737 | CEP290 | 0.008916 | -0.99108 |
| ENST00000360737 | PCNX3 | 0.009406 | 0.990594 |
| ENST00000360737 | RPUSD2 | 0.005587 | 0.994413 |
| ENST00000360737 | ZNF107 | 0.004507 | 0.995493 |
| ENST00000360737 | BICRA | 0.008144 | -0.99186 |
| ENST00000360737 | CEP63 | 8.95E-04 | 0.999105 |
| ENST00000446321 | CATG00000061038.1 | 0.005663 | -0.99434 |
| ENST00000446321 | MAPKBP1 | 0.002894 | 0.997106 |
| ENST00000446321 | CD19 | 0.005727 | -0.99427 |
| ENST00000446321 | AC093157.1 | 0.002535 | -0.99747 |
| ENST00000446321 | ZPR1 | 9.78E-04 | 0.999022 |
| ENST00000446321 | OR10R2 | 0.001338 | 0.998662 |
| ENST00000446321 | DDIT4 | 0.003184 | -0.99682 |
| NR_146891 | ZFAND5 | 1.06E-04 | 0.999894 |
| NR_146891 | LYL1 | 0.008263 | 0.991737 |
| NR_146891 | CD27 | 0.007652 | 0.992348 |
| NR_146891 | NAGLU | 0.007952 | 0.992048 |
| NR_146891 | TP53I13 | 0.002462 | 0.997538 |
| NR_146891 | FAM90A1 | 0.00815 | -0.99185 |
| NR_146891 | KRBA2 | 0.001974 | 0.998026 |
| NR_146891 | HIC1 | 0.005483 | 0.994517 |
| NR_146891 | CREBRF | 0.001864 | -0.99814 |
| NR_146891 | CATG00000054083.1 | 0.002754 | -0.99725 |
| NR_146891 | ZNF664 | 0.002844 | -0.99716 |
| NR_146891 | LCE2A | 0.003098 | 0.996902 |
| NR_146891 | PPTC7 | 0.006683 | -0.99332 |
| NR_146891 | CIB1 | 0.00546 | -0.99454 |
| ENST00000436724 | INPP5J | 5.41E-04 | 0.999459 |
| ENST00000436724 | RTN1 | 0.004459 | -0.99554 |
| ENST00000436724 | CNEP1R1 | 0.002484 | -0.99752 |
| ENST00000436724 | FRG1 | 0.003995 | -0.99601 |
| ENST00000436724 | AP3M2 | 0.008955 | -0.99105 |
| ENST00000436724 | PHKA1 | 0.009715 | 0.990285 |
| ENST00000436724 | COA1 | 0.007191 | -0.99281 |
| ENST00000436724 | BMPER | 2.14E-04 | 0.999786 |
| ENST00000589203 | FAM84A | 0.001855 | -0.99814 |
| ENST00000589203 | CAPS | 0.008794 | -0.99121 |
| ENST00000589203 | C20orf203 | 0.002454 | -0.99755 |
| ENST00000589203 | DUSP15 | 0.008083 | -0.99192 |
| ENST00000589203 | GMPPA | 0.009535 | -0.99047 |
| ENST00000589203 | AASDHPPT | 0.00352 | -0.99648 |
| ENST00000589203 | CATG00000053936.1 | 0.007982 | -0.99202 |
| ENST00000589203 | G6PC3 | 0.00179 | 0.99821 |
| ENST00000589203 | ARHGEF40 | 0.008188 | 0.991812 |
| ENST00000589203 | CATG00000107162.1 | 0.00909 | 0.99091 |
| ENST00000589203 | FAM110C | 0.001469 | 0.998531 |
| ENST00000589203 | C16orf78 | 0.004471 | 0.995529 |
| ENST00000589203 | OR5D18 | 0.005033 | 0.994967 |
| ENST00000489425 | TMEM132B | 0.001456 | 0.998544 |
| ENST00000489425 | EEF1D | 0.002627 | -0.99737 |
| ENST00000489425 | SEC14L6 | 0.008191 | 0.991809 |
| ENST00000489425 | IL4I1 | 0.002488 | -0.99751 |
| ENST00000489425 | TBKBP1 | 3.84E-04 | -0.99962 |
| ENST00000489425 | WT1 | 0.001639 | -0.99836 |
| ENST00000489425 | CATG00000039284.1 | 0.009734 | -0.99027 |
| ENST00000489425 | SCAMP3 | 5.11E-04 | -0.99949 |
| ENST00000489425 | PDE3B | 0.001265 | -0.99873 |
| ENST00000623342 | ZNF490 | 0.009547 | -0.99045 |
| ENST00000623342 | PNCK | 0.005076 | -0.99492 |
| ENST00000623342 | E4F1 | 0.006067 | -0.99393 |
| ENST00000623342 | MROH9 | 0.006414 | 0.993586 |
| ENST00000623342 | RNASEH2A | 0.007231 | -0.99277 |
| ENST00000623342 | NDUFC1 | 0.004063 | -0.99594 |
| ENST00000555105 | EXOC3L2 | 0.006 | -0.994 |
| ENST00000555105 | PZP | 0.003785 | 0.996215 |
| ENST00000555105 | CLEC4D | 0.001813 | 0.998187 |
| ENST00000555105 | WSCD2 | 0.009235 | -0.99076 |
| ENST00000533576 | ANKRD13D | 0.009974 | 0.990026 |
| ENST00000533576 | WNT16 | 0.00674 | -0.99326 |
| ENST00000533576 | LCK | 0.008361 | 0.991639 |
| ENST00000533576 | AGPS | 0.003127 | 0.996873 |
| ENST00000533576 | TEX13D | 0.001647 | -0.99835 |
| ENST00000533576 | NLRP9 | 0.009648 | -0.99035 |
| ENST00000533576 | IRX3 | 0.002931 | -0.99707 |
| ENST00000533576 | SNX16 | 0.002438 | 0.997562 |
| ENST00000533576 | CD180 | 0.00789 | 0.99211 |
| ENST00000533576 | DNAJB13 | 0.008044 | -0.99196 |
| ENST00000533576 | SPATA13 | 0.0068 | -0.9932 |
| ENST00000533576 | PLTP | 0.004628 | -0.99537 |
| ENST00000533576 | PLCH2 | 0.004413 | -0.99559 |
| ENST00000533576 | COBL | 9.80E-04 | -0.99902 |
| ENST00000533576 | CATG00000038465.1 | 0.005541 | -0.99446 |
| ENST00000533576 | SIPA1L2 | 0.008466 | -0.99153 |
| ENST00000533576 | PIPOX | 2.95E-04 | 0.999705 |
| ENST00000424852 | HIGD2A | 0.005914 | -0.99409 |
| ENST00000424852 | SYBU | 0.003452 | 0.996548 |
| ENST00000424852 | RFC2 | 0.007892 | -0.99211 |
| ENST00000424852 | EXOC2 | 0.006661 | -0.99334 |
| ENST00000424852 | CATG00000101330.1 | 0.004691 | -0.99531 |
| ENST00000424852 | ACAP3 | 0.006785 | 0.993215 |
| ENST00000424852 | SH3D21 | 0.00395 | 0.99605 |
| ENST00000424852 | PIKFYVE | 0.005672 | -0.99433 |
| ENST00000424852 | KIF21B | 0.004569 | 0.995431 |
| ENST00000424852 | TPRKB | 0.007935 | -0.99207 |
| ENST00000424852 | ARHGEF26 | 0.002385 | 0.997615 |
| ENST00000424852 | PHKA1 | 0.006875 | 0.993125 |
| ENST00000424852 | CATG00000026557.1 | 0.002887 | 0.997113 |
| ENST00000424852 | PSME1 | 0.002935 | -0.99707 |
| ENST00000424852 | CLRN1 | 0.003175 | 0.996825 |
| ENST00000424852 | DOCK1 | 0.005347 | 0.994653 |
| ENST00000424852 | DOHH | 0.002755 | 0.997245 |
| ENST00000426539 | TOB1 | 0.009838 | 0.990162 |
| ENST00000426539 | PPARD | 2.92E-04 | -0.99971 |
| ENST00000426539 | PTPRN | 0.004106 | -0.99589 |
| ENST00000426539 | PRKDC | 2.13E-04 | 0.999787 |
| ENST00000426539 | METTL3 | 0.009464 | 0.990536 |
| ENST00000426539 | ACO1 | 0.001292 | -0.99871 |
| ENST00000426539 | USF2 | 0.003249 | 0.996751 |
| ENST00000426539 | SMAP2 | 0.008563 | 0.991437 |
| ENST00000427950 | FILIP1 | 1.82E-05 | -0.99998 |
| ENST00000427950 | WNT16 | 0.00537 | 0.99463 |
| ENST00000427950 | HMGXB4 | 0.008163 | -0.99184 |
| ENST00000427950 | NLRP9 | 0.006036 | 0.993964 |
| ENST00000427950 | PMPCA | 0.007652 | 0.992348 |
| ENST00000427950 | UNC45A | 0.007088 | 0.992912 |
| ENST00000427950 | CD180 | 0.002248 | -0.99775 |
| ENST00000427950 | CAPNS1 | 0.003674 | -0.99633 |
| ENST00000427950 | RACK1 | 0.001416 | 0.998584 |
| ENST00000427950 | KRTAP4-7 | 0.005491 | 0.994509 |
| ENST00000427950 | CDKN3 | 0.00553 | -0.99447 |
| ENST00000427950 | RPUSD2 | 0.002308 | -0.99769 |
| NR_144516 | ZNF30 | 0.008525 | -0.99148 |
| NR_144516 | SZRD1 | 0.004998 | -0.995 |
| NR_144516 | FAM84A | 9.28E-04 | -0.99907 |
| NR_144516 | DUSP15 | 0.008081 | -0.99192 |
| NR_144516 | GMPPA | 0.00892 | -0.99108 |
| NR_144516 | AASDHPPT | 0.007624 | -0.99238 |
| NR_144516 | G6PC3 | 6.73E-04 | 0.999327 |
| NR_144516 | RTN4 | 0.006812 | 0.993188 |
| NR_144516 | MAP7 | 0.005554 | 0.994446 |
| NR_144516 | EFCAB8 | 0.009696 | 0.990304 |
| NR_144516 | OR5D18 | 0.006175 | 0.993825 |
| ENST00000445280 | CATG00000003494.1 | 0.002703 | -0.9973 |
| ENST00000445280 | CACHD1 | 0.003356 | -0.99664 |
| ENST00000445280 | INSC | 0.00828 | -0.99172 |
| ENST00000445280 | CLEC4G | 0.009835 | -0.99016 |
| ENST00000445280 | PCDHGA3 | 0.006026 | -0.99397 |
| ENST00000445280 | OAS1 | 7.13E-04 | -0.99929 |
| ENST00000445280 | CLCNKB | 0.007673 | -0.99233 |
| ENST00000445280 | DCD | 0.005254 | -0.99475 |
| ENST00000445280 | CSNK1A1 | 8.87E-04 | 0.999113 |
| ENST00000445280 | ZNF587B | 0.004758 | 0.995242 |
| ENST00000445280 | INO80B | 0.002448 | -0.99755 |
| ENST00000445280 | ACP6 | 0.006507 | 0.993493 |
| ENST00000445280 | RPL17 | 3.06E-04 | 0.999694 |
| ENST00000445280 | WAPL | 0.008873 | 0.991127 |
| ENST00000445280 | PLCL1 | 0.004719 | 0.995281 |
| ENST00000445280 | GABPA | 9.54E-04 | -0.99905 |
| ENST00000368047 | E4F1 | 0.00887 | -0.99113 |
| ENST00000368047 | BECN1 | 0.005732 | -0.99427 |
| ENST00000368047 | ZNF736 | 0.001237 | -0.99876 |
| ENST00000368047 | CCDC42 | 0.006289 | -0.99371 |
| ENST00000435738 | LRMDA | 0.006056 | 0.993944 |
| ENST00000435738 | PHF13 | 4.12E-04 | -0.99959 |
| ENST00000435738 | CTSL | 0.007055 | 0.992945 |
| ENST00000435738 | VMP1 | 0.00448 | -0.99552 |
| ENST00000435738 | GDF2 | 0.006868 | -0.99313 |
| ENST00000435738 | OR10H1 | 0.007806 | -0.99219 |
| ENST00000461248 | HMGB2 | 0.005507 | -0.99449 |
| ENST00000461248 | IGFN1 | 0.003042 | -0.99696 |
| ENST00000461248 | YWHAB | 0.001986 | 0.998014 |
| ENST00000461248 | GSTA4 | 4.53E-04 | 0.999547 |
| ENST00000461248 | CXXC1 | 0.007725 | -0.99227 |
| ENST00000461248 | TBX3 | 0.005135 | 0.994865 |
| ENST00000461248 | GPR161 | 0.004908 | 0.995092 |
| ENST00000461248 | EIF1AD | 0.00851 | 0.99149 |
| ENST00000461248 | KRT83 | 0.003082 | 0.996918 |
| ENST00000461248 | TPTE | 0.008491 | 0.991509 |
| ENST00000461248 | CAPRIN2 | 0.006487 | 0.993513 |
| ENST00000461248 | CLEC5A | 1.04E-04 | 0.999896 |
| ENST00000461248 | CATG00000089121.1 | 0.007965 | -0.99204 |
| ENST00000461248 | RBM7 | 0.005596 | 0.994404 |
| ENST00000461248 | CPOX | 0.007635 | 0.992365 |
| ENST00000461248 | GSG1L | 0.002242 | 0.997758 |
| ENST00000461248 | ERFE | 0.002694 | -0.99731 |
| ENST00000461248 | PAK3 | 0.005891 | 0.994109 |
| ENST00000461248 | SNRPD2 | 0.001514 | 0.998486 |
| ENST00000461248 | SPATA31D1 | 0.008423 | 0.991577 |
| ENST00000461248 | CATG00000034210.1 | 0.008655 | 0.991345 |
| ENST00000461248 | ZC3H12D | 0.006816 | -0.99318 |
| ENST00000461248 | LIM2 | 0.001271 | 0.998729 |
| ENST00000461248 | TNFRSF13B | 0.006388 | -0.99361 |
| ENST00000461248 | CCDC174 | 0.004008 | -0.99599 |
| ENST00000421505 | PLAGL1 | 0.002494 | -0.99751 |
| ENST00000421505 | RHBDD3 | 0.004853 | -0.99515 |
| ENST00000421505 | UBE2V1 | 0.009085 | -0.99091 |
| ENST00000421505 | AIFM3 | 0.005374 | -0.99463 |
| ENST00000421505 | VGLL3 | 9.20E-04 | 0.99908 |
| ENST00000421505 | MMP2 | 0.004161 | 0.995839 |
| ENST00000421505 | FAM186A | 0.00307 | 0.99693 |
| ENST00000360656 | MMP1 | 4.08E-04 | -0.99959 |
| ENST00000360656 | PARP6 | 0.007978 | 0.992022 |
| ENST00000360656 | C15orf65 | 0.009436 | 0.990564 |
| ENST00000360656 | NTNG1 | 0.008964 | -0.99104 |
| ENST00000360656 | PMIS2 | 0.004667 | 0.995333 |
| ENST00000519828 | PFDN6 | 0.002723 | -0.99728 |
| ENST00000519828 | CLEC1A | 8.99E-05 | -0.99991 |
| ENST00000519828 | IMPA2 | 0.004056 | -0.99594 |
| ENST00000519828 | HMGXB4 | 0.007401 | -0.9926 |
| ENST00000519828 | GGCT | 0.008448 | -0.99155 |
| ENST00000519828 | FNDC10 | 0.002184 | 0.997816 |
| ENST00000519828 | RPL11 | 0.005211 | -0.99479 |
| ENST00000519828 | UNC45A | 0.0067 | 0.9933 |
| ENST00000519828 | THOC5 | 0.009884 | 0.990116 |
| ENST00000519828 | PNPLA1 | 0.004519 | 0.995481 |
| ENST00000519828 | IDH1 | 0.003308 | -0.99669 |
| ENST00000519828 | TUBGCP4 | 0.003763 | 0.996237 |
| ENST00000519828 | JDP2 | 0.008481 | 0.991519 |
| ENST00000519828 | TAF8 | 2.20E-04 | -0.99978 |
| ENST00000519828 | PSMA1 | 0.004997 | -0.995 |
| ENST00000519828 | MCM3AP | 0.006845 | 0.993155 |
| ENST00000519828 | CDKN3 | 0.006043 | -0.99396 |
| ENST00000519828 | USP9X | 0.002941 | 0.997059 |
| ENST00000519828 | ZNF431 | 0.007602 | 0.992398 |
| ENST00000563763 | INTS2 | 0.004439 | -0.99556 |
| ENST00000563763 | RFC2 | 2.52E-04 | -0.99975 |
| ENST00000563763 | CNEP1R1 | 0.005214 | -0.99479 |
| ENST00000563763 | SH3D21 | 0.003049 | 0.996951 |
| ENST00000563763 | TP73 | 3.87E-04 | 0.999613 |
| ENST00000563763 | ARHGEF26 | 0.0078 | 0.9922 |
| ENST00000563763 | PHKA1 | 0.00651 | 0.99349 |
| ENST00000563763 | CATG00000074344.1 | 0.00683 | 0.99317 |
| ENST00000563763 | CATG00000026557.1 | 0.003236 | 0.996764 |
| ENST00000563763 | CLRN1 | 0.008185 | 0.991815 |
| ENST00000563763 | DOCK1 | 0.001077 | 0.998923 |
| ENST00000563763 | DEFA6 | 0.005314 | 0.994686 |
| ENST00000563763 | CATG00000027020.1 | 0.007231 | 0.992769 |
| ENST00000465257 | ZFY | 1.05E-04 | -0.9999 |
| ENST00000465257 | CCT8L2 | 0.007013 | -0.99299 |
| ENST00000465257 | PAK5 | 0.003412 | -0.99659 |
| ENST00000465257 | SSX1 | 5.06E-04 | 0.999494 |
| ENST00000465257 | WSCD2 | 0.006933 | 0.993067 |
| ENST00000465257 | GRIN3B | 0.005878 | -0.99412 |
| ENST00000465257 | PFKFB4 | 0.004909 | -0.99509 |
| ENST00000465257 | ALS2CR12 | 0.009376 | 0.990624 |
| ENST00000465257 | CATG00000068089.1 | 0.002878 | 0.997122 |
| ENST00000465257 | DEFA4 | 0.005052 | 0.994948 |
| ENST00000437753 | PTPRN | 0.005859 | 0.994141 |
| ENST00000437753 | EHD4 | 0.001521 | -0.99848 |
| ENST00000437753 | PRKDC | 0.008314 | -0.99169 |
| ENST00000437753 | SMAP2 | 0.003806 | -0.99619 |
| ENST00000525012 | ZFAND5 | 0.003069 | -0.99693 |
| ENST00000525012 | LYL1 | 0.003715 | -0.99628 |
| ENST00000525012 | SLC13A4 | 0.004576 | -0.99542 |
| ENST00000525012 | TP53I13 | 7.99E-04 | -0.9992 |
| ENST00000525012 | KRBA2 | 0.002949 | -0.99705 |
| ENST00000525012 | HIC1 | 0.002112 | -0.99789 |
| ENST00000525012 | TFF2 | 0.004584 | -0.99542 |
| ENST00000525012 | ORC3 | 0.008867 | -0.99113 |
| ENST00000525012 | CREBRF | 0.005359 | 0.994641 |
| ENST00000525012 | CATG00000054083.1 | 0.0062 | 0.9938 |
| ENST00000525012 | ZNF664 | 0.002924 | 0.997076 |
| ENST00000525012 | LCE2A | 0.008648 | -0.99135 |
| uc010fjw.1 | ACSBG1 | 0.002365 | -0.99763 |
| uc010fjw.1 | EIF3L | 0.003611 | -0.99639 |
| uc010fjw.1 | USP28 | 0.00199 | -0.99801 |
| uc010fjw.1 | PRR15L | 0.005482 | 0.994518 |
| uc010fjw.1 | KRTAP15-1 | 0.002135 | 0.997865 |
| ENST00000592918 | PZP | 0.009284 | -0.99072 |
| ENST00000592918 | CLEC4D | 0.003587 | -0.99641 |
| ENST00000592918 | CLRN2 | 5.33E-04 | -0.99947 |
| ENST00000592918 | SLC11A1 | 0.003908 | -0.99609 |
| ENST00000505158 | FCN2 | 1.71E-04 | 0.999829 |
| ENST00000505158 | CATG00000063531.1 | 0.005109 | 0.994891 |
| ENST00000505158 | UBE2V1 | 0.006703 | 0.993297 |
| ENST00000505158 | CEP44 | 0.006692 | 0.993308 |
| ENST00000505158 | PCBP4 | 0.007881 | -0.99212 |
| ENST00000505158 | NFE2L2 | 0.004276 | -0.99572 |
| ENST00000505158 | SIVA1 | 8.78E-04 | -0.99912 |
| ENST00000505158 | TCTEX1D4 | 0.008664 | -0.99134 |
| ENST00000505158 | SMIM6 | 0.001716 | 0.998284 |
| ENST00000505158 | SH3RF3 | 0.004868 | -0.99513 |
| ENST00000505158 | ERVW-1 | 0.008469 | -0.99153 |
| NR_120422 | EGF | 0.002591 | -0.99741 |
| NR_120422 | CATG00000099282.1 | 0.002173 | 0.997827 |
| NR_120422 | CATG00000026669.1 | 0.008386 | 0.991614 |
| NR_120422 | CARNS1 | 0.007335 | 0.992665 |
| ENST00000592195 | TMEM132B | 0.004767 | 0.995233 |
| ENST00000592195 | GATA4 | 0.005218 | 0.994782 |
| ENST00000592195 | IL4I1 | 7.08E-04 | -0.99929 |
| ENST00000592195 | TBKBP1 | 0.007819 | -0.99218 |
| ENST00000592195 | WT1 | 0.004248 | -0.99575 |
| ENST00000592195 | SLC39A12 | 0.007668 | 0.992332 |
| ENST00000592195 | SCAMP3 | 0.006671 | -0.99333 |
| ENST00000592195 | PDE3B | 0.004694 | -0.99531 |
| NR_149099 | NR2F2 | 0.003862 | 0.996138 |
| NR_149099 | GBA | 0.005227 | 0.994773 |
| NR_149099 | RPL23A | 0.009435 | -0.99057 |
| NR_149099 | SMPD1 | 0.004415 | 0.995585 |
| NR_149099 | DHODH | 0.00547 | -0.99453 |
| NR_149099 | PRPF8 | 0.008211 | -0.99179 |
| NR_149099 | CCDC149 | 0.008456 | 0.991544 |
| NR_149099 | CEP290 | 0.006707 | 0.993293 |
| NR_149099 | PCNX3 | 0.00197 | -0.99803 |
| NR_149099 | CDK11B | 0.004368 | -0.99563 |
| NR_149099 | TCTEX1D4 | 0.001234 | -0.99877 |
| NR_149099 | TIGD3 | 0.001895 | -0.9981 |
| NR_149099 | MAP1LC3A | 0.004912 | 0.995088 |
| NR_149099 | LBP | 0.007738 | 0.992262 |
| NR_149099 | SH3RF3 | 0.00602 | -0.99398 |
| NR_149099 | ZNF107 | 0.005218 | -0.99478 |
| NR_149099 | ACMSD | 4.59E-04 | -0.99954 |
| ENST00000482511 | ANKRD13D | 0.001187 | -0.99881 |
| ENST00000482511 | TRMT6 | 0.008387 | 0.991613 |
| ENST00000482511 | RECK | 0.00751 | 0.99249 |
| ENST00000482511 | EDC3 | 0.008231 | 0.991769 |
| ENST00000482511 | CATG00000035925.1 | 0.006174 | 0.993826 |
| ENST00000482511 | MYL3 | 0.006403 | 0.993597 |
| ENST00000482511 | CATG00000087963.1 | 0.001219 | 0.998781 |
| ENST00000482511 | EPHB6 | 0.003954 | 0.996046 |
| ENST00000482511 | DNAJB13 | 0.001399 | 0.998601 |
| ENST00000482511 | OSBPL5 | 0.008992 | 0.991008 |
| ENST00000482511 | PLTP | 0.009651 | 0.990349 |
| ENST00000482511 | CORO7-PAM16 | 0.008207 | -0.99179 |
| ENST00000482511 | BTG1 | 0.003059 | -0.99694 |
| ENST00000482511 | CATG00000021838.1 | 0.007937 | -0.99206 |
| ENST00000482511 | BICRA | 0.007566 | 0.992434 |
| ENST00000482511 | TSFM | 0.007754 | -0.99225 |
| ENST00000418106 | CATG00000003494.1 | 0.005187 | 0.994813 |
| ENST00000418106 | YWHAB | 0.002621 | 0.997379 |
| ENST00000418106 | CACHD1 | 6.86E-04 | 0.999314 |
| ENST00000418106 | GPR161 | 0.00679 | 0.99321 |
| ENST00000418106 | EIF1AD | 0.008144 | 0.991856 |
| ENST00000418106 | CATG00000022188.1 | 0.00467 | 0.99533 |
| ENST00000418106 | CAPRIN2 | 0.006204 | 0.993796 |
| ENST00000418106 | CLEC5A | 0.008122 | 0.991878 |
| ENST00000418106 | CATG00000089121.1 | 0.003914 | -0.99609 |
| ENST00000418106 | OAS1 | 0.006955 | 0.993045 |
| ENST00000418106 | RBM7 | 0.001706 | 0.998294 |
| ENST00000418106 | GSG1L | 0.009746 | 0.990254 |
| ENST00000418106 | ERFE | 0.006051 | -0.99395 |
| ENST00000418106 | SNRPD2 | 0.005973 | 0.994027 |
| ENST00000418106 | SPATA31D1 | 0.007212 | 0.992788 |
| ENST00000418106 | CSNK1A1 | 0.009217 | -0.99078 |
| ENST00000418106 | CATG00000038058.1 | 0.007313 | 0.992687 |
| ENST00000418106 | ZC3H12D | 0.005904 | -0.9941 |
| ENST00000418106 | LIM2 | 0.008333 | 0.991667 |
| ENST00000418106 | INO80B | 0.004896 | 0.995104 |
| ENST00000418106 | RPL17 | 0.006739 | -0.99326 |
| ENST00000418106 | PLCL1 | 2.46E-04 | -0.99975 |
| ENST00000418106 | AL627171.2 | 0.009727 | -0.99027 |
| ENST00000418106 | TNFRSF13B | 5.84E-04 | -0.99942 |
| ENST00000418106 | GABPA | 0.004985 | 0.995015 |
| ENST00000452599 | CATG00000038628.1 | 0.00509 | 0.99491 |
| ENST00000452599 | OR5T2 | 0.004531 | -0.99547 |
| ENST00000452599 | RAB5B | 0.004502 | -0.9955 |
| ENST00000443055 | SCAMP2 | 0.004756 | -0.99524 |
| ENST00000443055 | RASSF6 | 0.002861 | -0.99714 |
| ENST00000443055 | PCBP4 | 0.006011 | 0.993989 |
| ENST00000443055 | PEX14 | 0.002144 | -0.99786 |
| ENST00000443055 | XYLT2 | 0.002943 | -0.99706 |
| ENST00000443055 | ZNF713 | 0.001576 | -0.99842 |
| ENST00000443055 | ABR | 0.002875 | -0.99712 |
| ENST00000443055 | KCTD1 | 0.004297 | -0.9957 |
| ENST00000443055 | CATG00000047316.1 | 0.006804 | -0.9932 |
| ENST00000443055 | NTF3 | 0.003873 | -0.99613 |
| ENST00000443055 | RTL8B | 0.007131 | 0.992869 |
| ENST00000443055 | ERVW-1 | 0.007121 | 0.992879 |
| ENST00000443055 | ZNF37A | 0.009545 | 0.990455 |
| ENST00000443055 | CATG00000117842.1 | 0.008628 | -0.99137 |
| ENST00000443055 | DICER1 | 0.004692 | 0.995308 |
| ENST00000443055 | C16orf86 | 0.009131 | 0.990869 |
| ENST00000573802 | IGFN1 | 0.006775 | -0.99323 |
| ENST00000573802 | SLC7A4 | 3.27E-04 | 0.999673 |
| ENST00000573802 | GPR161 | 0.008233 | 0.991767 |
| ENST00000573802 | EIF1AD | 0.007237 | 0.992763 |
| ENST00000573802 | SMPD1 | 0.006828 | 0.993172 |
| ENST00000573802 | SAMD1 | 0.003283 | -0.99672 |
| ENST00000573802 | CAPRIN2 | 0.008897 | 0.991103 |
| ENST00000573802 | CPOX | 0.003445 | 0.996555 |
| ENST00000573802 | CATG00000057824.1 | 0.004185 | 0.995815 |
| ENST00000573802 | WNT2 | 0.009499 | 0.990501 |
| ENST00000573802 | PAK3 | 0.008695 | 0.991305 |
| ENST00000573802 | IQCF5 | 0.006002 | 0.993998 |
| ENST00000573802 | KLF6 | 8.79E-04 | -0.99912 |
| ENST00000573802 | MAP1LC3A | 0.008892 | 0.991108 |
| ENST00000573802 | LBP | 0.004292 | 0.995708 |
| ENST00000573802 | FAM53B | 0.003007 | -0.99699 |
| ENST00000573802 | LDHB | 0.006609 | -0.99339 |
| ENST00000573802 | REG4 | 0.005931 | -0.99407 |
| ENST00000573802 | AL627171.2 | 0.007767 | -0.99223 |
| ENST00000316853 | RIPK1 | 0.004121 | -0.99588 |
| ENST00000316853 | NAPA | 0.001792 | 0.998208 |
| ENST00000316853 | PPP1CB | 0.00108 | 0.99892 |
| ENST00000316853 | P3H3 | 0.008343 | -0.99166 |
| ENST00000316853 | HDHD5 | 0.009369 | -0.99063 |
| ENST00000316853 | PTGER1 | 0.001551 | -0.99845 |
| ENST00000316853 | PGM5 | 0.009579 | -0.99042 |
| ENST00000316853 | CATG00000107158.1 | 0.004372 | 0.995628 |
| ENST00000316853 | CATG00000110054.1 | 6.44E-04 | 0.999356 |
| ENST00000316853 | TMEM230 | 0.00986 | 0.99014 |
| ENST00000316853 | YY2 | 0.002646 | -0.99735 |
| ENST00000316853 | RCSD1 | 0.005076 | -0.99492 |
| ENST00000316853 | KIAA1211L | 0.004785 | -0.99521 |
| ENST00000316853 | CHI3L1 | 0.009764 | 0.990236 |
| ENST00000316853 | CATG00000020281.1 | 2.11E-04 | -0.99979 |
| ENST00000437488 | TRMT11 | 0.005151 | -0.99485 |
| ENST00000437488 | ADGRE1 | 0.006429 | -0.99357 |
| ENST00000437488 | TMEM132B | 0.004085 | 0.995915 |
| ENST00000437488 | SEC14L6 | 0.008104 | 0.991896 |
| ENST00000437488 | IL4I1 | 0.002076 | -0.99792 |
| ENST00000437488 | NEFM | 0.009601 | -0.9904 |
| ENST00000437488 | TBKBP1 | 0.009495 | -0.9905 |
| ENST00000437488 | SLC39A12 | 0.003229 | 0.996771 |
| ENST00000437488 | PDE3B | 0.00425 | -0.99575 |
| ENST00000563724 | CATG00000003494.1 | 0.004092 | -0.99591 |
| ENST00000563724 | YWHAB | 0.009622 | -0.99038 |
| ENST00000563724 | CACHD1 | 8.53E-04 | -0.99915 |
| ENST00000563724 | INSC | 0.005972 | -0.99403 |
| ENST00000563724 | GPR161 | 0.009676 | -0.99032 |
| ENST00000563724 | EIF1AD | 0.008637 | -0.99136 |
| ENST00000563724 | CAPRIN2 | 0.007836 | -0.99216 |
| ENST00000563724 | CATG00000089121.1 | 0.004583 | 0.995417 |
| ENST00000563724 | OAS1 | 8.05E-04 | -0.9992 |
| ENST00000563724 | RBM7 | 0.009283 | -0.99072 |
| ENST00000563724 | CSNK1A1 | 0.001689 | 0.998311 |
| ENST00000563724 | ZNF587B | 0.006097 | 0.993903 |
| ENST00000563724 | INO80B | 0.003706 | -0.99629 |
| ENST00000563724 | RPL17 | 8.17E-04 | 0.999183 |
| ENST00000563724 | WAPL | 0.007835 | 0.992165 |
| ENST00000563724 | PLCL1 | 0.001558 | 0.998442 |
| ENST00000563724 | AL627171.2 | 0.007938 | 0.992062 |
| ENST00000563724 | TNFRSF13B | 0.006284 | 0.993716 |
| ENST00000563724 | GABPA | 0.002198 | -0.9978 |
| NR_027254 | B4GALNT1 | 0.004748 | 0.995252 |
| NR_027254 | ZFY | 0.003383 | 0.996617 |
| NR_027254 | S1PR5 | 0.004999 | 0.995001 |
| NR_027254 | RAB8A | 0.007919 | 0.992081 |
| NR_027254 | SSX1 | 0.003445 | -0.99655 |
| NR_027254 | WSCD2 | 0.007529 | -0.99247 |
| NR_027254 | GRIN3B | 0.001827 | 0.998173 |
| NR_027254 | PTPRK | 0.007162 | -0.99284 |
| NR_027254 | PLEKHG3 | 0.009622 | 0.990378 |
| NR_027254 | DEFA4 | 3.03E-05 | -0.99997 |
| NR_003579 | SHANK2 | 0.005089 | -0.99491 |
| NR_003579 | B4GALNT1 | 0.004417 | 0.995583 |
| NR_003579 | S1PR5 | 0.006544 | 0.993456 |
| NR_003579 | RP1L1 | 4.24E-04 | 0.999576 |
| NR_003579 | DEFA1 | 0.004871 | -0.99513 |
| NR_003579 | PLEKHG3 | 0.001828 | 0.998172 |
| ENST00000636047 | ECSIT | 0.003638 | 0.996362 |
| ENST00000636047 | SIRPG | 0.008533 | 0.991467 |
| ENST00000636047 | SSBP2 | 0.00663 | 0.99337 |
| ENST00000636047 | CHST8 | 0.007296 | 0.992704 |
| ENST00000636047 | FRG1 | 0.001324 | -0.99868 |
| ENST00000636047 | TGFBR3 | 0.006991 | 0.993009 |
| ENST00000636047 | SLC22A6 | 0.001353 | -0.99865 |
| ENST00000636047 | COA1 | 0.004048 | -0.99595 |
| ENST00000636047 | COA5 | 0.001708 | -0.99829 |
| ENST00000636047 | MORN3 | 0.007274 | -0.99273 |
| ENST00000636047 | BMPER | 0.009218 | 0.990782 |
| ENST00000636047 | VPS13A | 0.009134 | 0.990866 |
| ENST00000636047 | FGF9 | 0.009941 | -0.99006 |
| ENST00000413185 | DMAC2 | 0.008108 | 0.991892 |
| ENST00000413185 | RPS7 | 0.006672 | 0.993328 |
| ENST00000413185 | 11-Mar | 0.002882 | -0.99712 |
| ENST00000413185 | CPLX1 | 0.001245 | -0.99875 |
| ENST00000413185 | PELP1 | 0.007728 | 0.992272 |
| ENST00000413185 | GMEB2 | 0.001295 | -0.99871 |
| ENST00000413185 | OR13A1 | 9.45E-04 | -0.99905 |
| ENST00000413185 | ANKHD1 | 0.002137 | -0.99786 |
| ENST00000413185 | SMUG1 | 4.99E-05 | -0.99995 |
| ENST00000413185 | DCUN1D1 | 0.004342 | -0.99566 |
| ENST00000413185 | C3orf84 | 7.34E-04 | 0.999266 |
| ENST00000452342 | GNL1 | 0.001534 | -0.99847 |
| ENST00000452342 | DEAF1 | 0.008238 | 0.991762 |
| ENST00000452342 | DNAJA4 | 0.007805 | 0.992195 |
| ENST00000452342 | GPR25 | 6.57E-04 | 0.999343 |
| ENST00000452342 | FANCD2OS | 0.005632 | 0.994368 |
| ENST00000452342 | SHC2 | 0.008043 | 0.991957 |
| ENST00000452342 | EFEMP2 | 3.90E-04 | -0.99961 |
| ENST00000452342 | OR4X1 | 0.002885 | 0.997115 |
| ENST00000452342 | HLA-F | 5.81E-04 | 0.999419 |
| ENST00000452342 | CATG00000024701.1 | 0.008215 | 0.991785 |
| ENST00000584542 | ECSIT | 0.001513 | 0.998487 |
| ENST00000584542 | CATG00000056264.1 | 7.83E-04 | 0.999217 |
| ENST00000584542 | SIRPG | 0.004313 | 0.995687 |
| ENST00000584542 | CATG00000003494.1 | 0.00665 | 0.99335 |
| ENST00000584542 | SSBP2 | 2.04E-04 | 0.999796 |
| ENST00000584542 | CHST8 | 8.07E-04 | 0.999193 |
| ENST00000584542 | CLEC4G | 0.003232 | 0.996768 |
| ENST00000584542 | TMC2 | 0.001519 | 0.998481 |
| ENST00000584542 | CATG00000022188.1 | 0.004421 | 0.995579 |
| ENST00000584542 | CLK3 | 0.006779 | 0.993221 |
| ENST00000584542 | TPRX1 | 0.004922 | 0.995078 |
| ENST00000584542 | RBM7 | 0.009124 | 0.990876 |
| ENST00000584542 | CLCNKB | 0.009283 | 0.990717 |
| ENST00000584542 | SLC22A6 | 0.006974 | -0.99303 |
| ENST00000584542 | SPATA31D1 | 0.007649 | 0.992351 |
| ENST00000584542 | CATG00000038058.1 | 0.004759 | 0.995241 |
| ENST00000584542 | ZC3H12D | 0.008459 | -0.99154 |
| ENST00000584542 | INO80B | 0.007 | 0.993 |
| ENST00000584542 | TNFRSF13B | 0.009801 | -0.9902 |
| ENST00000584542 | FGF9 | 0.007627 | -0.99237 |
| ENST00000420981 | ZFAND5 | 0.00178 | 0.99822 |
| ENST00000420981 | CD27 | 0.001563 | 0.998437 |
| ENST00000420981 | TP53I13 | 0.006272 | 0.993728 |
| ENST00000420981 | FAM90A1 | 0.001919 | -0.99808 |
| ENST00000420981 | KRBA2 | 0.008128 | 0.991872 |
| ENST00000420981 | HIC1 | 0.009167 | 0.990833 |
| ENST00000420981 | CLK3 | 0.008977 | 0.991023 |
| ENST00000420981 | TPRX1 | 0.007144 | 0.992856 |
| ENST00000420981 | CLCNKB | 0.008081 | 0.991919 |
| ENST00000420981 | CREBRF | 0.001147 | -0.99885 |
| ENST00000420981 | CATG00000054083.1 | 0.001483 | -0.99852 |
| ENST00000420981 | ZNF664 | 0.004201 | -0.9958 |
| ENST00000420981 | ELF2 | 0.006328 | -0.99367 |
| ENST00000420981 | LCE2A | 5.36E-04 | 0.999464 |
| ENST00000420981 | PPTC7 | 0.001718 | -0.99828 |
| ENST00000420981 | CIB1 | 0.001851 | -0.99815 |
| ENST00000420877 | NR2F2 | 7.25E-04 | 0.999275 |
| ENST00000420877 | GBA | 0.006596 | 0.993404 |
| ENST00000420877 | RPL23A | 0.003503 | -0.9965 |
| ENST00000420877 | SMPD1 | 0.002153 | 0.997847 |
| ENST00000420877 | DHODH | 0.002525 | -0.99748 |
| ENST00000420877 | PRPF8 | 0.00599 | -0.99401 |
| ENST00000420877 | CCDC149 | 0.008096 | 0.991904 |
| ENST00000420877 | CEP290 | 0.00364 | 0.99636 |
| ENST00000420877 | PCNX3 | 0.004568 | -0.99543 |
| ENST00000420877 | WNT2 | 0.005304 | 0.994696 |
| ENST00000420877 | CDK11B | 0.007275 | -0.99273 |
| ENST00000420877 | TCTEX1D4 | 0.004919 | -0.99508 |
| ENST00000420877 | TIGD3 | 0.001214 | -0.99879 |
| ENST00000420877 | MAP1LC3A | 0.005635 | 0.994365 |
| ENST00000420877 | LBP | 0.00391 | 0.99609 |
| ENST00000420877 | ZNF107 | 0.005216 | -0.99478 |
| ENST00000420877 | REG4 | 0.005282 | -0.99472 |
| ENST00000420877 | ACMSD | 0.00348 | -0.99652 |
| NR_046259 | SLC38A10 | 0.008383 | 0.991617 |
| NR_046259 | PLEKHD1 | 0.005669 | -0.99433 |
| NR_046259 | MLLT1 | 0.005085 | 0.994915 |
| NR_046259 | GPCPD1 | 0.003194 | -0.99681 |
| NR_046259 | STMND1 | 0.002343 | 0.997657 |
| NR_046259 | LYPD8 | 0.004032 | 0.995968 |
| NR_046259 | DRC1 | 0.00942 | 0.99058 |
| NR_046259 | ZFYVE26 | 0.009558 | 0.990442 |
| NR_104019 | GSTM5 | 0.007352 | -0.99265 |
| NR_104019 | SUPV3L1 | 0.005543 | 0.994457 |
| NR_104019 | UBE2V1 | 0.007997 | 0.992003 |
| NR_104019 | FAM186A | 0.004656 | -0.99534 |
| NR_104019 | PGAM5 | 4.82E-04 | -0.99952 |
| ENST00000424958 | PHF20 | 0.006199 | -0.9938 |
| ENST00000424958 | MMP1 | 6.12E-04 | -0.99939 |
| ENST00000424958 | CDO1 | 0.008745 | -0.99126 |
| ENST00000424958 | C15orf65 | 0.00607 | 0.99393 |
| ENST00000424958 | NTNG1 | 0.00418 | -0.99582 |
| ENST00000424958 | PMIS2 | 5.97E-04 | 0.999403 |
| ENST00000424958 | FCRL1 | 0.008389 | 0.991611 |
| NR_152808 | ZNF185 | 0.005313 | 0.994687 |
| NR_152808 | FAM169B | 0.002383 | 0.997617 |
| NR_152808 | GPR82 | 0.007258 | 0.992742 |
| ENST00000507035 | TMED3 | 0.009015 | -0.99098 |
| ENST00000507035 | PFDN6 | 0.006734 | -0.99327 |
| ENST00000507035 | PPP3CC | 0.007386 | -0.99261 |
| ENST00000507035 | CLEC1A | 0.003308 | -0.99669 |
| ENST00000507035 | IMPA2 | 0.004883 | -0.99512 |
| ENST00000507035 | FNDC10 | 6.21E-04 | 0.999379 |
| ENST00000507035 | WDR33 | 0.008636 | 0.991364 |
| ENST00000507035 | PNPLA1 | 0.004332 | 0.995668 |
| ENST00000507035 | IDH1 | 0.00189 | -0.99811 |
| ENST00000507035 | ZNF607 | 0.004455 | 0.995545 |
| ENST00000507035 | PPP5D1 | 0.006842 | 0.993158 |
| ENST00000507035 | TAF8 | 0.003085 | -0.99691 |
| ENST00000507035 | OR6F1 | 0.002306 | 0.997694 |
| ENST00000507035 | PSMA1 | 0.003881 | -0.99612 |
| ENST00000514343 | EPB41L1 | 0.003984 | 0.996016 |
| ENST00000514343 | RASSF6 | 0.002603 | 0.997397 |
| ENST00000514343 | PEX14 | 0.006491 | 0.993509 |
| ENST00000514343 | XYLT2 | 0.008171 | 0.991829 |
| ENST00000514343 | ZNF713 | 7.21E-04 | 0.999279 |
| ENST00000514343 | ABR | 0.004908 | 0.995092 |
| ENST00000514343 | KCTD1 | 0.008222 | 0.991778 |
| ENST00000514343 | CATG00000047316.1 | 0.00251 | 0.99749 |
| ENST00000514343 | ZMYND15 | 0.001373 | 0.998627 |
| ENST00000514343 | CATG00000057824.1 | 0.009672 | 0.990328 |
| ENST00000514343 | IQCF5 | 0.009769 | 0.990231 |
| ENST00000514343 | NTF3 | 0.003941 | 0.996059 |
| ENST00000514343 | DNAL4 | 0.002837 | -0.99716 |
| ENST00000514343 | FAM53B | 0.009288 | -0.99071 |
| ENST00000514343 | LDHB | 0.005303 | -0.9947 |
| ENST00000514343 | ZNF37A | 0.005683 | -0.99432 |
| ENST00000514343 | DICER1 | 0.00281 | -0.99719 |
| NR_148035 | EDC3 | 0.00577 | -0.99423 |
| NR_148035 | WNT16 | 0.004027 | -0.99597 |
| NR_148035 | MYL3 | 0.008357 | -0.99164 |
| NR_148035 | PCP4 | 2.28E-04 | -0.99977 |
| NR_148035 | NLRP9 | 0.002073 | -0.99793 |
| NR_148035 | PRPF8 | 0.009443 | 0.990557 |
| NR_148035 | NPDC1 | 0.005099 | -0.9949 |
| NR_148035 | CCDC149 | 0.005087 | -0.99491 |
| NR_148035 | DNAJB13 | 0.009281 | -0.99072 |
| NR_148035 | OSBPL5 | 0.005779 | -0.99422 |
| NR_148035 | RACK1 | 0.008569 | -0.99143 |
| NR_148035 | CHCHD6 | 0.009327 | -0.99067 |
| NR_148035 | TMEM51 | 0.004848 | -0.99515 |
| NR_148035 | BTG1 | 0.008032 | 0.991968 |
| NR_148035 | RPUSD2 | 0.009373 | 0.990627 |
| NR_148035 | ZNF107 | 0.007061 | 0.992939 |
| NR_148035 | BICRA | 0.003637 | -0.99636 |
| NR_148035 | CEP63 | 0.003906 | 0.996094 |
| ENST00000498872 | GNL1 | 0.006967 | -0.99303 |
| ENST00000498872 | VRK3 | 0.009597 | 0.990403 |
| ENST00000498872 | BLCAP | 0.009274 | 0.990726 |
| ENST00000498872 | FANCD2OS | 0.007292 | 0.992708 |
| ENST00000498872 | CATG00000023328.1 | 0.00199 | -0.99801 |
| ENST00000498872 | EFEMP2 | 0.004631 | -0.99537 |
| ENST00000498872 | OR4X1 | 0.001412 | 0.998588 |
| ENST00000498872 | MAP3K5 | 4.33E-04 | 0.999567 |
| ENST00000498872 | HLA-F | 0.00388 | 0.99612 |
| ENST00000498872 | CATG00000024701.1 | 2.11E-04 | 0.999789 |
| ENST00000578387 | SCAMP2 | 0.001048 | -0.99895 |
| ENST00000578387 | EPB41L1 | 0.008673 | -0.99133 |
| ENST00000578387 | RASSF6 | 0.007425 | -0.99258 |
| ENST00000578387 | PEX14 | 1.17E-04 | -0.99988 |
| ENST00000578387 | XYLT2 | 3.08E-04 | -0.99969 |
| ENST00000578387 | ZNF713 | 0.003163 | -0.99684 |
| ENST00000578387 | ABR | 0.008047 | -0.99195 |
| ENST00000578387 | IKZF4 | 0.007276 | -0.99272 |
| ENST00000578387 | KCTD1 | 8.98E-04 | -0.9991 |
| ENST00000578387 | CATG00000047316.1 | 0.005015 | -0.99498 |
| ENST00000578387 | NTF3 | 0.009338 | -0.99066 |
| ENST00000578387 | SHROOM3 | 0.009154 | -0.99085 |
| ENST00000578387 | INKA2 | 0.00517 | -0.99483 |
| ENST00000578387 | RTL8B | 0.009816 | 0.990184 |
| ENST00000578387 | ABCA13 | 0.008669 | -0.99133 |
| ENST00000578387 | ZNF37A | 0.005895 | 0.994105 |
| ENST00000578387 | DICER1 | 0.002807 | 0.997193 |
| NR_027293 | MMP1 | 0.008078 | -0.99192 |
| NR_027293 | ACTL7B | 0.00968 | 0.99032 |
| NR_027293 | TTC38 | 0.006194 | -0.99381 |
| NR_027293 | IGFL2 | 0.007674 | -0.99233 |
| ENST00000565162 | TNFRSF18 | 0.006098 | -0.9939 |
| ENST00000565162 | GORASP1 | 0.009343 | -0.99066 |
| ENST00000565162 | SLC2A4 | 0.008869 | 0.991131 |
| ENST00000565162 | HIRA | 0.009703 | -0.9903 |
| ENST00000565162 | TMEM155 | 0.005158 | 0.994842 |
| ENST00000565162 | SPATA33 | 0.005701 | -0.9943 |
| ENST00000565162 | KLHL35 | 0.001078 | -0.99892 |
| ENST00000565162 | SLC22A12 | 0.004112 | 0.995888 |
| ENST00000565162 | PLPPR4 | 0.006815 | -0.99318 |
| ENST00000565162 | FCHSD2 | 0.009599 | -0.9904 |
| ENST00000565162 | HLA-A | 0.006229 | -0.99377 |
| ENST00000565162 | MT1F | 5.81E-05 | -0.99994 |
| ENST00000565162 | WWP1 | 0.001059 | -0.99894 |
| ENST00000565162 | APOBR | 0.00492 | -0.99508 |
| ENST00000565162 | KCNK10 | 0.007018 | 0.992982 |
| ENST00000565162 | TNXB | 0.008316 | 0.991684 |
| ENST00000565162 | KYAT3 | 0.002069 | 0.997931 |
| ENST00000565162 | PAQR5 | 0.004203 | 0.995797 |
| ENST00000565162 | TNRC6B | 0.00972 | 0.99028 |
| ENST00000565162 | ATG4C | 2.88E-04 | 0.999712 |
| ENST00000581072 | HMGB2 | 0.007764 | -0.99224 |
| ENST00000581072 | IGFN1 | 0.003732 | -0.99627 |
| ENST00000581072 | YWHAB | 8.70E-04 | 0.99913 |
| ENST00000581072 | GSTA4 | 0.001253 | 0.998747 |
| ENST00000581072 | TBX3 | 0.006756 | 0.993244 |
| ENST00000581072 | GPR161 | 0.003658 | 0.996342 |
| ENST00000581072 | EIF1AD | 0.006799 | 0.993201 |
| ENST00000581072 | KRT83 | 0.004611 | 0.995389 |
| ENST00000581072 | CATG00000022188.1 | 0.008867 | 0.991133 |
| ENST00000581072 | CAPRIN2 | 0.004867 | 0.995133 |
| ENST00000581072 | CLEC5A | 1.14E-04 | 0.999886 |
| ENST00000581072 | CATG00000089121.1 | 0.005787 | -0.99421 |
| ENST00000581072 | RBM7 | 0.00394 | 0.99606 |
| ENST00000581072 | CPOX | 0.006797 | 0.993203 |
| ENST00000581072 | GSG1L | 0.002486 | 0.997514 |
| ENST00000581072 | ERFE | 0.001772 | -0.99823 |
| ENST00000581072 | PAK3 | 0.007659 | 0.992341 |
| ENST00000581072 | SNRPD2 | 0.001179 | 0.998821 |
| ENST00000581072 | SPATA31D1 | 0.007444 | 0.992556 |
| ENST00000581072 | CATG00000034210.1 | 0.009355 | 0.990645 |
| ENST00000581072 | ZC3H12D | 0.005823 | -0.99418 |
| ENST00000581072 | LIM2 | 9.85E-04 | 0.999015 |
| ENST00000581072 | PLCL1 | 0.008364 | -0.99164 |
| ENST00000581072 | TNFRSF13B | 0.004347 | -0.99565 |
| ENST00000581072 | CCDC174 | 0.006157 | -0.99384 |
| ENST00000585832 | MYLK3 | 0.006926 | -0.99307 |
| ENST00000585832 | FBLN2 | 0.001555 | -0.99844 |
| ENST00000585832 | CYP21A2 | 0.002519 | 0.997481 |
| ENST00000585832 | SLC39A12 | 0.005164 | 0.994836 |
| ENST00000585832 | ZNRD1 | 0.009153 | 0.990847 |
| ENST00000585832 | SYNGR3 | 0.001021 | 0.998979 |
| ENST00000585832 | EDDM3A | 0.001072 | 0.998928 |
| ENST00000429962 | TBC1D29 | 0.001852 | 0.998148 |
| ENST00000429962 | CCDC42 | 0.005199 | 0.994801 |
| ENST00000429962 | PRR15L | 0.007608 | -0.99239 |
| ENST00000446290 | EMP1 | 0.002945 | 0.997055 |
| ENST00000446290 | CATG00000029374.1 | 8.53E-04 | -0.99915 |
| ENST00000420146 | GBA | 0.006296 | 0.993704 |
| ENST00000420146 | SLC7A4 | 2.61E-04 | 0.999739 |
| ENST00000420146 | EIF1AD | 0.008482 | 0.991518 |
| ENST00000420146 | SMPD1 | 0.006677 | 0.993323 |
| ENST00000420146 | SAMD1 | 0.001853 | -0.99815 |
| ENST00000420146 | ZMYND15 | 0.008138 | 0.991862 |
| ENST00000420146 | CPOX | 0.005568 | 0.994432 |
| ENST00000420146 | CATG00000057824.1 | 0.001306 | 0.998694 |
| ENST00000420146 | IQCF5 | 0.00395 | 0.99605 |
| ENST00000420146 | KLF6 | 0.003599 | -0.9964 |
| ENST00000420146 | MAP1LC3A | 0.005699 | 0.994301 |
| ENST00000420146 | LBP | 0.005286 | 0.994714 |
| ENST00000420146 | FAM53B | 0.001134 | -0.99887 |
| ENST00000420146 | LDHB | 0.002297 | -0.9977 |
| ENST00000420146 | REG4 | 0.009183 | -0.99082 |
| ENST00000420146 | AL627171.2 | 0.007605 | -0.99239 |
| ENST00000565047 | PLEKHA2 | 0.003957 | -0.99604 |
| ENST00000565047 | PLAG1 | 0.005016 | -0.99498 |
| ENST00000565047 | DYNC2H1 | 0.005478 | 0.994522 |
| ENST00000512458 | TAS2R42 | 0.006844 | 0.993156 |
| ENST00000512458 | B3GNT3 | 8.38E-04 | -0.99916 |
| ENST00000512458 | PPP3CC | 0.00684 | -0.99316 |
| ENST00000512458 | HMGB2 | 0.008277 | -0.99172 |
| ENST00000512458 | PSRC1 | 0.005241 | -0.99476 |
| ENST00000512458 | PLIN4 | 0.006759 | 0.993241 |
| ENST00000512458 | FOXO6 | 0.001789 | 0.998211 |
| ENST00000512458 | CXXC1 | 0.009258 | -0.99074 |
| ENST00000512458 | DGAT2L6 | 0.007074 | 0.992926 |
| ENST00000512458 | CATG00000053512.1 | 0.003711 | -0.99629 |
| ENST00000512458 | HLF | 0.001136 | 0.998864 |
| ENST00000512458 | SRRD | 0.003517 | 0.996483 |
| ENST00000512458 | TPTE | 0.00635 | 0.99365 |
| ENST00000512458 | CSTL1 | 0.004648 | 0.995352 |
| ENST00000512458 | FYB2 | 0.001998 | 0.998002 |
| ENST00000512458 | SLC12A3 | 0.002866 | -0.99713 |
| ENST00000512458 | PPP5D1 | 0.005516 | 0.994484 |
| ENST00000512458 | WNT8B | 0.007599 | 0.992401 |
| ENST00000512458 | UNC5C | 0.008225 | -0.99177 |
| ENST00000512458 | OXT | 6.51E-04 | 0.999349 |
| ENST00000512458 | FAM174A | 4.78E-06 | -1 |
| ENST00000512458 | CFAP410 | 0.001724 | 0.998276 |
| ENST00000512458 | HPS1 | 0.006379 | 0.993621 |
| ENST00000488027 | DMAC2 | 0.009578 | 0.990422 |
| ENST00000488027 | RSL1D1 | 0.002341 | -0.99766 |
| ENST00000488027 | GORASP1 | 0.002619 | -0.99738 |
| ENST00000488027 | SLC2A4 | 0.008682 | 0.991318 |
| ENST00000488027 | HIRA | 0.007755 | -0.99225 |
| ENST00000488027 | ABCB8 | 0.005126 | 0.994874 |
| ENST00000488027 | RASL10A | 0.009704 | -0.9903 |
| ENST00000488027 | HERPUD2 | 3.84E-04 | -0.99962 |
| ENST00000488027 | SLC22A12 | 0.007509 | 0.992491 |
| ENST00000488027 | PLPPR4 | 0.009387 | -0.99061 |
| ENST00000488027 | FCHSD2 | 0.002535 | -0.99747 |
| ENST00000488027 | ZNF579 | 0.007169 | 0.992831 |
| ENST00000488027 | PTPN23 | 2.78E-04 | 0.999722 |
| ENST00000488027 | AGO2 | 0.002644 | 0.997356 |
| ENST00000488027 | TRIM47 | 0.004896 | 0.995104 |
| ENST00000488027 | DHDH | 4.23E-04 | 0.999577 |
| ENST00000498140 | CAPS | 0.003865 | -0.99614 |
| ENST00000498140 | GMPPA | 0.002224 | -0.99778 |
| ENST00000498140 | CATG00000053936.1 | 0.00216 | -0.99784 |
| ENST00000498140 | ARHGEF40 | 0.002067 | 0.997933 |
| ENST00000498140 | CATG00000107162.1 | 5.72E-04 | 0.999428 |
| ENST00000498140 | C16orf78 | 0.003318 | 0.996682 |
| ENST00000498140 | MSI1 | 0.004435 | 0.995565 |
| ENST00000479341 | SCAMP2 | 0.008588 | 0.991412 |
| ENST00000479341 | EPB41L1 | 4.28E-04 | 0.999572 |
| ENST00000479341 | INSC | 0.003188 | 0.996812 |
| ENST00000479341 | PEX14 | 0.007652 | 0.992348 |
| ENST00000479341 | XYLT2 | 0.008305 | 0.991695 |
| ENST00000479341 | ZNF713 | 0.007981 | 0.992019 |
| ENST00000479341 | MYH3 | 0.004898 | -0.9951 |
| ENST00000479341 | KCTD1 | 0.006221 | 0.993779 |
| ENST00000479341 | CATG00000047316.1 | 0.00115 | 0.99885 |
| ENST00000479341 | ZMYND15 | 0.004433 | 0.995567 |
| ENST00000479341 | PAN3 | 0.006138 | -0.99386 |
| ENST00000479341 | DNAL4 | 0.002488 | -0.99751 |
| ENST00000479341 | ZNF587B | 0.00425 | -0.99575 |
| ENST00000479341 | INKA2 | 0.00235 | 0.99765 |
| ENST00000479341 | ZNF37A | 3.83E-04 | -0.99962 |
| ENST00000479341 | WAPL | 0.001591 | -0.99841 |
| ENST00000479341 | DICER1 | 0.002091 | -0.99791 |
| ENST00000529902 | EXOC3L2 | 0.002298 | -0.9977 |
| ENST00000529902 | PLD3 | 0.002776 | 0.997224 |
| ENST00000529902 | CLEC4D | 0.00789 | 0.99211 |
| ENST00000529902 | WSCD2 | 0.003337 | -0.99666 |
| ENST00000529902 | CATG00000039609.1 | 0.005107 | -0.99489 |
| ENST00000602852 | CATG00000003494.1 | 0.005143 | -0.99486 |
| ENST00000602852 | YWHAB | 0.004623 | -0.99538 |
| ENST00000602852 | CACHD1 | 1.42E-04 | -0.99986 |
| ENST00000602852 | INSC | 0.008743 | -0.99126 |
| ENST00000602852 | GPR161 | 0.005967 | -0.99403 |
| ENST00000602852 | EIF1AD | 0.006086 | -0.99391 |
| ENST00000602852 | CATG00000022188.1 | 0.009193 | -0.99081 |
| ENST00000602852 | CAPRIN2 | 0.004867 | -0.99513 |
| ENST00000602852 | CATG00000089121.1 | 0.002468 | 0.997532 |
| ENST00000602852 | OAS1 | 0.00339 | -0.99661 |
| ENST00000602852 | RBM7 | 0.004702 | -0.9953 |
| ENST00000602852 | ERFE | 0.006259 | 0.993741 |
| ENST00000602852 | SNRPD2 | 0.009976 | -0.99002 |
| ENST00000602852 | CSNK1A1 | 0.00504 | 0.99496 |
| ENST00000602852 | LIM2 | 0.009416 | -0.99058 |
| ENST00000602852 | INO80B | 0.00475 | -0.99525 |
| ENST00000602852 | RPL17 | 0.003451 | 0.996549 |
| ENST00000602852 | PLCL1 | 1.89E-04 | 0.999811 |
| ENST00000602852 | AL627171.2 | 0.006557 | 0.993443 |
| ENST00000602852 | TNFRSF13B | 0.002627 | 0.997373 |
| ENST00000602852 | GABPA | 0.003857 | -0.99614 |
| ENST00000582435 | SLC24A5 | 0.004354 | 0.995646 |
| ENST00000582435 | TCOF1 | 0.005883 | -0.99412 |
| ENST00000582435 | TMC5 | 0.001055 | -0.99894 |
| ENST00000582435 | SAMD13 | 0.007361 | -0.99264 |
| ENST00000582435 | IKZF4 | 0.005513 | -0.99449 |
| ENST00000582435 | STRADB | 0.002988 | -0.99701 |
| ENST00000582435 | MACROD2 | 0.00867 | -0.99133 |
| ENST00000582435 | PURG | 0.001688 | -0.99831 |
| ENST00000582435 | CACNG6 | 4.90E-04 | -0.99951 |
| ENST00000582435 | SHROOM3 | 0.003165 | -0.99683 |
| ENST00000582435 | RAMP1 | 0.001491 | 0.998509 |
| ENST00000582435 | PARVG | 0.001165 | -0.99884 |
| ENST00000582435 | ABCA13 | 0.003587 | -0.99641 |
| ENST00000582435 | YPEL5 | 0.008329 | -0.99167 |
| NR_024497 | TMED3 | 0.007557 | -0.99244 |
| NR_024497 | TAS2R42 | 0.009926 | 0.990074 |
| NR_024497 | PPP3CC | 0.009559 | -0.99044 |
| NR_024497 | PSRC1 | 0.009342 | -0.99066 |
| NR_024497 | ABCB8 | 0.00647 | 0.99353 |
| NR_024497 | LTA4H | 0.002212 | -0.99779 |
| NR_024497 | TUT4 | 0.001764 | -0.99824 |
| NR_024497 | WDR33 | 0.002061 | 0.997939 |
| NR_024497 | PALM3 | 0.009747 | -0.99025 |
| NR_024497 | CATG00000063823.1 | 0.002248 | 0.997752 |
| NR_024497 | ZNF607 | 0.007891 | 0.992109 |
| NR_024497 | PSMD4 | 0.001635 | -0.99836 |
| NR_024497 | OR6F1 | 0.008199 | 0.991801 |
| NR_024497 | ADAM28 | 0.001594 | -0.99841 |
| NR_024497 | TRIM47 | 0.007287 | 0.992713 |
| NR_024497 | CATG00000087047.1 | 0.002877 | 0.997123 |
| NR_024497 | AC109583.1 | 0.002936 | 0.997064 |
| NR_024497 | RAD51AP2 | 6.43E-04 | -0.99936 |
| ENST00000414475 | DEAF1 | 0.009456 | 0.990544 |
| ENST00000414475 | VRK3 | 0.001902 | 0.998098 |
| ENST00000414475 | BLCAP | 0.001488 | 0.998512 |
| ENST00000414475 | FANCD2OS | 0.00653 | 0.99347 |
| ENST00000414475 | CATG00000023328.1 | 5.65E-04 | -0.99943 |
| ENST00000414475 | ACSL6 | 0.002416 | 0.997584 |
| ENST00000414475 | OR4X1 | 0.006204 | 0.993796 |
| ENST00000414475 | MAP3K5 | 0.00509 | 0.99491 |
| ENST00000414475 | CATG00000024701.1 | 0.002933 | 0.997067 |
| ENST00000457218 | ECSIT | 0.009851 | 0.990149 |
| ENST00000457218 | CATG00000056264.1 | 0.001524 | 0.998476 |
| ENST00000457218 | CATG00000003494.1 | 5.66E-04 | 0.999434 |
| ENST00000457218 | SSBP2 | 0.005217 | 0.994783 |
| ENST00000457218 | CACHD1 | 0.006037 | 0.993963 |
| ENST00000457218 | CHST8 | 0.006627 | 0.993373 |
| ENST00000457218 | CLEC4G | 8.65E-04 | 0.999135 |
| ENST00000457218 | TMC2 | 0.003286 | 0.996714 |
| ENST00000457218 | CATG00000022188.1 | 0.008622 | 0.991378 |
| ENST00000457218 | TPRX1 | 0.003237 | 0.996763 |
| ENST00000457218 | OAS1 | 0.009693 | 0.990307 |
| ENST00000457218 | CLCNKB | 0.002522 | 0.997478 |
| ENST00000457218 | INO80B | 7.12E-04 | 0.999288 |
| ENST00000457218 | RPL17 | 0.008135 | -0.99187 |
| ENST00000457218 | PPTC7 | 0.008628 | -0.99137 |
| ENST00000457218 | PLCL1 | 0.006596 | -0.9934 |
| ENST00000457218 | TNFRSF13B | 0.008733 | -0.99127 |
| ENST00000457218 | GABPA | 0.002016 | 0.997984 |
| ENST00000457218 | CIB1 | 0.009967 | -0.99003 |
| NR_138485 | TCF4 | 0.00472 | -0.99528 |
| NR_138485 | DUSP2 | 0.006339 | 0.993661 |
| NR_038379 | IGFN1 | 0.008018 | -0.99198 |
| NR_038379 | YWHAB | 0.002661 | 0.997339 |
| NR_038379 | CACHD1 | 0.003459 | 0.996541 |
| NR_038379 | INSC | 0.008822 | 0.991178 |
| NR_038379 | GPR161 | 8.94E-04 | 0.999106 |
| NR_038379 | EIF1AD | 0.001184 | 0.998816 |
| NR_038379 | SAMD1 | 0.008271 | -0.99173 |
| NR_038379 | CAPRIN2 | 5.36E-04 | 0.999464 |
| NR_038379 | CLEC5A | 0.005061 | 0.994939 |
| NR_038379 | CATG00000089121.1 | 1.02E-04 | -0.9999 |
| NR_038379 | OAS1 | 0.008302 | 0.991698 |
| NR_038379 | RBM7 | 0.00608 | 0.99392 |
| NR_038379 | CPOX | 0.003549 | 0.996451 |
| NR_038379 | ERFE | 0.001265 | -0.99874 |
| NR_038379 | SNRPD2 | 0.007657 | 0.992343 |
| NR_038379 | IQCF5 | 0.006937 | 0.993063 |
| NR_038379 | LIM2 | 0.003152 | 0.996848 |
| NR_038379 | RPL17 | 0.009116 | -0.99088 |
| NR_038379 | PLCL1 | 0.002917 | -0.99708 |
| NR_038379 | AL627171.2 | 0.002135 | -0.99786 |
| NR_038379 | TNFRSF13B | 0.004227 | -0.99577 |
| NR_110056 | FILIP1 | 0.00875 | -0.99125 |
| NR_110056 | WNT16 | 0.003641 | 0.996359 |
| NR_110056 | PCP4 | 0.00181 | 0.99819 |
| NR_110056 | TMEM242 | 0.006358 | -0.99364 |
| NR_110056 | NLRP9 | 0.001708 | 0.998292 |
| NR_110056 | PRPF8 | 0.00748 | -0.99252 |
| NR_110056 | UNC45A | 0.008834 | 0.991166 |
| NR_110056 | NPDC1 | 0.009711 | 0.990289 |
| NR_110056 | CD180 | 0.009022 | -0.99098 |
| NR_110056 | CCDC149 | 0.004325 | 0.995675 |
| NR_110056 | THOC5 | 0.009312 | 0.990688 |
| NR_110056 | RACK1 | 0.004043 | 0.995957 |
| NR_110056 | CHCHD6 | 0.005381 | 0.994619 |
| NR_110056 | TMEM51 | 0.003222 | 0.996778 |
| NR_110056 | RPUSD2 | 0.0044 | -0.9956 |
| NR_110056 | ZNF107 | 0.006969 | -0.99303 |
| NR_110056 | BICRA | 0.00838 | 0.99162 |
| NR_110056 | CEP63 | 0.001598 | -0.9984 |
| NR_146494 | INPP5J | 0.006378 | -0.99362 |
| NR_146494 | RFC2 | 0.009435 | 0.990565 |
| NR_146494 | CATG00000101330.1 | 0.00597 | 0.99403 |
| NR_146494 | CNEP1R1 | 2.86E-04 | 0.999714 |
| NR_146494 | FRG1 | 0.005438 | 0.994562 |
| NR_146494 | ARHGEF26 | 0.006567 | -0.99343 |
| NR_146494 | PHKA1 | 0.002042 | -0.99796 |
| NR_146494 | CATG00000026557.1 | 0.005861 | -0.99414 |
| NR_146494 | COA1 | 0.001817 | 0.998183 |
| NR_146494 | PSME1 | 0.009645 | 0.990355 |
| NR_146494 | DOCK1 | 0.005441 | -0.99456 |
| NR_146494 | BMPER | 0.004886 | -0.99511 |
| ENST00000380612 | STEAP3 | 0.005085 | 0.994915 |
| ENST00000380612 | SLC13A4 | 0.004375 | 0.995625 |
| ENST00000380612 | TFF2 | 0.005076 | 0.994924 |
| ENST00000380612 | ORC3 | 0.008089 | 0.991911 |
| ENST00000380612 | UGT2B28 | 0.003133 | 0.996867 |
| ENST00000509369 | SLC38A10 | 0.006112 | -0.99389 |
| ENST00000509369 | ZNF30 | 0.00776 | 0.99224 |
| ENST00000509369 | CATG00000092654.1 | 0.009295 | 0.990705 |
| ENST00000509369 | SZRD1 | 0.007438 | 0.992562 |
| ENST00000509369 | APBB3 | 0.008716 | -0.99128 |
| ENST00000509369 | PLEKHD1 | 9.23E-04 | 0.999077 |
| ENST00000509369 | MLLT1 | 0.001015 | -0.99899 |
| ENST00000509369 | DNAH6 | 1.29E-05 | -0.99999 |
| ENST00000509369 | MAP7 | 0.003501 | -0.9965 |
| NR_037631 | TAS2R42 | 0.008128 | 0.991872 |
| NR_037631 | GRINA | 0.002573 | 0.997427 |
| NR_037631 | HMGB2 | 0.006397 | -0.9936 |
| NR_037631 | PLIN4 | 4.07E-04 | 0.999593 |
| NR_037631 | LILRB1 | 0.00304 | -0.99696 |
| NR_037631 | RASL10A | 0.005142 | -0.99486 |
| NR_037631 | GSTA4 | 0.009121 | 0.990879 |
| NR_037631 | CXXC1 | 0.001076 | -0.99892 |
| NR_037631 | TBX3 | 0.005142 | 0.994858 |
| NR_037631 | KRT83 | 0.004938 | 0.995062 |
| NR_037631 | TPTE | 0.009165 | 0.990835 |
| NR_037631 | NFX1 | 0.004247 | -0.99575 |
| NR_037631 | CATG00000012021.1 | 0.002584 | -0.99742 |
| NR_037631 | FYB2 | 0.006577 | 0.993423 |
| NR_037631 | PEA15 | 0.003991 | 0.996009 |
| NR_037631 | ESRRB | 8.43E-04 | 0.999157 |
| NR_037631 | IFT122 | 8.88E-04 | 0.999112 |
| NR_037631 | SIGLEC7 | 0.001979 | 0.998021 |
| NR_037631 | WNT8B | 0.003428 | 0.996572 |
| NR_037631 | UNC5C | 0.004108 | -0.99589 |
| NR_037631 | AGO2 | 0.009838 | 0.990162 |
| NR_037631 | FAM174A | 0.009955 | -0.99004 |
| NR_037631 | CFAP410 | 0.009595 | 0.990405 |
| NR_037631 | HPS1 | 0.002295 | 0.997705 |
| NR_037631 | CCDC174 | 0.002765 | -0.99724 |
| NR_037631 | FAM151A | 8.14E-04 | 0.999186 |
| ENST00000428529 | UBE2V1 | 0.009681 | -0.99032 |
| ENST00000428529 | VGLL3 | 0.007044 | 0.992956 |
| ENST00000428529 | MMP2 | 0.00977 | 0.99023 |
| ENST00000428529 | FAM186A | 4.44E-04 | 0.999556 |
| ENST00000428529 | PGAM5 | 0.005386 | 0.994614 |
| ENST00000506340 | B3GNT3 | 0.004151 | -0.99585 |
| ENST00000506340 | HMGB2 | 0.004621 | -0.99538 |
| ENST00000506340 | PLIN4 | 0.008595 | 0.991405 |
| ENST00000506340 | FOXO6 | 6.86E-04 | 0.999314 |
| ENST00000506340 | CXXC1 | 0.008885 | -0.99111 |
| ENST00000506340 | DGAT2L6 | 0.006171 | 0.993829 |
| ENST00000506340 | HLF | 0.005666 | 0.994334 |
| ENST00000506340 | SRRD | 5.28E-04 | 0.999472 |
| ENST00000506340 | TPTE | 0.002387 | 0.997613 |
| ENST00000506340 | CSTL1 | 0.002462 | 0.997538 |
| ENST00000506340 | FYB2 | 0.001017 | 0.998983 |
| ENST00000506340 | SLC12A3 | 0.009043 | -0.99096 |
| ENST00000506340 | WNT2 | 0.007721 | 0.992279 |
| ENST00000506340 | PAK3 | 0.004717 | 0.995283 |
| ENST00000506340 | PPP5D1 | 0.009765 | 0.990235 |
| ENST00000506340 | OXT | 3.54E-04 | 0.999646 |
| ENST00000506340 | FAM174A | 0.001846 | -0.99815 |
| ENST00000506340 | REG4 | 0.009121 | -0.99088 |
| ENST00000506340 | CFAP410 | 0.006876 | 0.993124 |
| ENST00000523406 | B4GALNT1 | 0.008924 | -0.99108 |
| ENST00000523406 | ZFY | 0.004156 | -0.99584 |
| ENST00000523406 | S1PR5 | 0.00767 | -0.99233 |
| ENST00000523406 | RAB8A | 0.00403 | -0.99597 |
| ENST00000523406 | EEF1B2 | 0.006717 | 0.993283 |
| ENST00000523406 | SSX1 | 0.003378 | 0.996622 |
| ENST00000523406 | WSCD2 | 0.004349 | 0.995651 |
| ENST00000523406 | GRIN3B | 3.21E-04 | -0.99968 |
| ENST00000523406 | TNFAIP6 | 0.007075 | -0.99292 |
| ENST00000523406 | PTPRK | 0.003507 | 0.996493 |
| ENST00000523406 | DEFA4 | 9.24E-04 | 0.999076 |
| ENST00000418244 | NR2F2 | 0.002061 | 0.997939 |
| ENST00000418244 | RPL23A | 6.49E-04 | -0.99935 |
| ENST00000418244 | FOXO6 | 0.006817 | 0.993183 |
| ENST00000418244 | DGAT2L6 | 0.006012 | 0.993988 |
| ENST00000418244 | SMPD1 | 0.00375 | 0.99625 |
| ENST00000418244 | DHODH | 0.006791 | -0.99321 |
| ENST00000418244 | SRRD | 0.009004 | 0.990996 |
| ENST00000418244 | CSTL1 | 0.00386 | 0.99614 |
| ENST00000418244 | CEP290 | 0.008117 | 0.991883 |
| ENST00000418244 | WNT2 | 1.33E-05 | 0.999987 |
| ENST00000418244 | TIGD3 | 0.008944 | -0.99106 |
| ENST00000418244 | KLF6 | 0.004801 | -0.9952 |
| ENST00000418244 | LBP | 0.002351 | 0.997649 |
| ENST00000418244 | OXT | 0.009983 | 0.990017 |
| ENST00000418244 | REG4 | 3.38E-04 | -0.99966 |
| ENST00000490270 | CPSF3 | 2.61E-04 | 0.999739 |
| ENST00000490270 | C11orf71 | 5.86E-04 | 0.999414 |
| ENST00000490270 | MYLK3 | 0.002891 | 0.997109 |
| ENST00000490270 | FBLN2 | 0.008321 | 0.991679 |
| ENST00000490270 | TNK2 | 0.003991 | 0.996009 |
| ENST00000439198 | DMAC2 | 0.008276 | 0.991724 |
| ENST00000439198 | RPS7 | 0.007063 | 0.992937 |
| ENST00000439198 | 11-Mar | 0.00306 | -0.99694 |
| ENST00000439198 | CPLX1 | 0.001074 | -0.99893 |
| ENST00000439198 | PELP1 | 0.008243 | 0.991757 |
| ENST00000439198 | GMEB2 | 0.00137 | -0.99863 |
| ENST00000439198 | APOBR | 0.00966 | -0.99034 |
| ENST00000439198 | OR13A1 | 9.47E-04 | -0.99905 |
| ENST00000439198 | ANKHD1 | 0.001877 | -0.99812 |
| ENST00000439198 | SMUG1 | 3.72E-05 | -0.99996 |
| ENST00000439198 | DCUN1D1 | 0.004508 | -0.99549 |
| ENST00000439198 | C3orf84 | 5.95E-04 | 0.999405 |
| ENST00000503699 | ZC3H7B | 0.006216 | -0.99378 |
| ENST00000503699 | CATG00000061038.1 | 6.01E-04 | 0.999399 |
| ENST00000503699 | CD19 | 0.006857 | 0.993143 |
| ENST00000503699 | OR10R2 | 0.003475 | -0.99653 |
| ENST00000503699 | DDIT4 | 0.001298 | 0.998702 |
| ENST00000471277 | EXOC3L2 | 0.006042 | 0.993958 |
| ENST00000471277 | PZP | 0.00792 | -0.99208 |
| ENST00000471277 | CLEC4D | 0.001559 | -0.99844 |
| ENST00000471277 | CLRN2 | 0.0075 | -0.9925 |
| ENST00000471277 | SLC11A1 | 0.002619 | -0.99738 |
| NR_120607 | RIPK1 | 0.002018 | -0.99798 |
| NR_120607 | NAPA | 0.005954 | 0.994046 |
| NR_120607 | BTBD2 | 0.009497 | 0.990503 |
| NR_120607 | HDHD5 | 0.00418 | -0.99582 |
| NR_120607 | PTGER1 | 0.00286 | -0.99714 |
| NR_120607 | POP1 | 0.001719 | 0.998281 |
| NR_120607 | CATG00000110054.1 | 0.007879 | 0.992121 |
| NR_120607 | TMEM230 | 0.003013 | 0.996987 |
| NR_120607 | RAB8A | 0.009118 | 0.990882 |
| NR_120607 | EEF1B2 | 0.007566 | -0.99243 |
| NR_120607 | YY2 | 0.008362 | -0.99164 |
| NR_120607 | KIAA1211L | 5.75E-04 | -0.99943 |
| NR_120607 | TNFAIP6 | 0.005399 | 0.994601 |
| NR_120607 | PTPRK | 0.009799 | -0.9902 |
| NR_120607 | CATG00000039609.1 | 0.009944 | -0.99006 |
| NR_120607 | CATG00000020284.1 | 0.006012 | -0.99399 |
| ENST00000433094 | PGM5 | 0.002862 | 0.997138 |
| ENST00000433094 | CATG00000107158.1 | 0.007025 | -0.99298 |
| ENST00000433094 | DGAT2 | 0.006372 | 0.993628 |
| ENST00000433094 | SPRR1A | 9.72E-04 | -0.99903 |
| ENST00000433094 | LY6G6C | 9.63E-04 | 0.999037 |
| ENST00000433094 | AOAH | 0.001847 | -0.99815 |
| ENST00000433094 | MOK | 0.009401 | 0.990599 |
| ENST00000433094 | CHI3L1 | 0.005023 | -0.99498 |
| ENST00000433094 | CD38 | 0.003623 | 0.996377 |
| ENST00000440723 | AX748369 | 0.003975 | 0.996025 |
| ENST00000440723 | SLC38A10 | 0.006471 | 0.993529 |
| ENST00000440723 | ZNF30 | 0.009521 | -0.99048 |
| ENST00000440723 | PNPLA2 | 0.00892 | 0.99108 |
| ENST00000440723 | GPCPD1 | 0.004979 | -0.99502 |
| ENST00000440723 | STMND1 | 3.23E-04 | 0.999677 |
| ENST00000440723 | DRC1 | 0.001393 | 0.998607 |
| ENST00000440723 | FGD4 | 0.009621 | 0.990379 |
| ENST00000440723 | EFCAB8 | 0.009734 | 0.990266 |
| ENST00000412339 | RPUSD1 | 6.25E-04 | 0.999375 |
| ENST00000412339 | MYO7A | 0.009272 | -0.99073 |
| ENST00000412339 | ALB | 2.08E-04 | -0.99979 |
| ENST00000412339 | TNFSF13B | 0.006625 | 0.993375 |
| ENST00000412339 | HILPDA | 0.008235 | -0.99176 |
| ENST00000412339 | SP140 | 0.009898 | 0.990102 |
| ENST00000625268 | TNFRSF18 | 0.005575 | -0.99443 |
| ENST00000625268 | TMEM236 | 0.003906 | -0.99609 |
| ENST00000625268 | CPLX1 | 0.00417 | -0.99583 |
| ENST00000625268 | KLHL35 | 0.002226 | -0.99777 |
| ENST00000625268 | MT1F | 0.006351 | -0.99365 |
| ENST00000625268 | WWP1 | 0.001591 | -0.99841 |
| ENST00000625268 | APOBR | 4.55E-05 | -0.99995 |
| ENST00000625268 | OR13A1 | 0.009087 | -0.99091 |
| ENST00000625268 | TNXB | 0.00378 | 0.99622 |
| ENST00000625268 | ANKHD1 | 0.002399 | -0.9976 |
| ENST00000625268 | SMUG1 | 0.008198 | -0.9918 |
| ENST00000625268 | PAQR5 | 3.02E-04 | 0.999698 |
| ENST00000625268 | C3orf84 | 0.004819 | 0.995181 |
| ENST00000625268 | TNRC6B | 0.005597 | 0.994403 |
| ENST00000625268 | ATG4C | 0.00642 | 0.99358 |
| ENST00000500800 | LDLRAD4 | 0.008717 | 0.991283 |
| ENST00000500800 | NAAA | 0.008293 | 0.991707 |
| ENST00000500800 | GSTM5 | 0.008457 | -0.99154 |
| ENST00000500800 | RFC5 | 0.009024 | -0.99098 |
| ENST00000500800 | DLEC1 | 0.002146 | -0.99785 |
| ENST00000500800 | ASB11 | 0.009683 | 0.990317 |
| ENST00000500800 | HSDL2 | 0.004913 | 0.995087 |
| ENST00000500800 | ZNRD1 | 0.005887 | -0.99411 |
| ENST00000400814 | INTS2 | 0.00807 | -0.99193 |
| ENST00000400814 | CEP170 | 0.001019 | -0.99898 |
| ENST00000400814 | EXOC4 | 0.00339 | 0.99661 |
| ENST00000400814 | TRMT11 | 0.004542 | -0.99546 |
| ENST00000400814 | ADGRE1 | 0.001157 | -0.99884 |
| ENST00000400814 | CDIP1 | 0.002389 | -0.99761 |
| ENST00000400814 | DBI | 0.006635 | -0.99336 |
| ENST00000400814 | SEC14L6 | 0.003661 | 0.996339 |
| ENST00000400814 | NEMP2 | 6.90E-04 | 0.99931 |
| ENST00000400814 | NEFM | 0.001076 | -0.99892 |
| ENST00000400814 | CATG00000074344.1 | 0.009459 | 0.990541 |
| ENST00000400814 | DEFA6 | 0.005357 | 0.994643 |
| ENST00000400814 | CATG00000027020.1 | 0.001303 | 0.998697 |
| ENST00000525580 | FBLN2 | 0.009454 | -0.99055 |
| ENST00000525580 | GATA4 | 0.009843 | 0.990157 |
| ENST00000525580 | CYP21A2 | 0.004233 | 0.995767 |
| ENST00000525580 | IL4I1 | 0.009017 | -0.99098 |
| ENST00000525580 | SLC39A12 | 0.002431 | 0.997569 |
| ENST00000525580 | SYNGR3 | 0.00186 | 0.99814 |
| ENST00000525580 | EDDM3A | 0.006677 | 0.993323 |
| ENST00000529938 | TAS2R42 | 0.00158 | -0.99842 |
| ENST00000529938 | B3GNT3 | 0.005195 | 0.994805 |
| ENST00000529938 | GRINA | 0.009767 | -0.99023 |
| ENST00000529938 | PPP3CC | 0.001048 | 0.998952 |
| ENST00000529938 | PSRC1 | 3.66E-04 | 0.999634 |
| ENST00000529938 | LILRB1 | 0.008434 | 0.991566 |
| ENST00000529938 | RASL10A | 0.007363 | 0.992637 |
| ENST00000529938 | LTA4H | 0.002254 | 0.997746 |
| ENST00000529938 | TUT4 | 0.007135 | 0.992865 |
| ENST00000529938 | WDR33 | 0.004349 | -0.99565 |
| ENST00000529938 | CATG00000053512.1 | 0.001361 | 0.998639 |
| ENST00000529938 | HLF | 0.00292 | -0.99708 |
| ENST00000529938 | NFX1 | 0.006361 | 0.993639 |
| ENST00000529938 | PEA15 | 0.006585 | -0.99342 |
| ENST00000529938 | SLC12A3 | 0.001172 | 0.998828 |
| ENST00000529938 | PPP5D1 | 0.008142 | -0.99186 |
| ENST00000529938 | WNT8B | 0.005264 | -0.99474 |
| ENST00000529938 | OR6F1 | 0.007268 | -0.99273 |
| ENST00000529938 | UNC5C | 0.004821 | 0.995179 |
| ENST00000529938 | CATG00000087047.1 | 0.00323 | -0.99677 |
| ENST00000529938 | FAM174A | 0.007255 | 0.992745 |
| ENST00000529938 | CFAP410 | 0.002142 | -0.99786 |
| ENST00000529938 | HPS1 | 0.006348 | -0.99365 |
| ENST00000529938 | RAD51AP2 | 0.003213 | 0.996787 |
| ENST00000461942 | PTPN6 | 0.006434 | -0.99357 |
| ENST00000461942 | FILIP1 | 0.002557 | -0.99744 |
| ENST00000461942 | WNT16 | 0.005243 | 0.994757 |
| ENST00000461942 | NOL9 | 0.002773 | -0.99723 |
| ENST00000461942 | NLRP9 | 0.007777 | 0.992223 |
| ENST00000461942 | IRX3 | 0.003986 | 0.996014 |
| ENST00000461942 | CD180 | 6.94E-04 | -0.99931 |
| ENST00000461942 | CAPNS1 | 0.006902 | -0.9931 |
| ENST00000461942 | SPATA13 | 0.003145 | 0.996855 |
| ENST00000461942 | RACK1 | 0.007657 | 0.992343 |
| ENST00000461942 | COBL | 0.004391 | 0.995609 |
| ENST00000461942 | KRTAP4-7 | 0.00728 | 0.99272 |
| ENST00000461942 | RPUSD2 | 0.009978 | -0.99002 |
| ENST00000461942 | SIPA1L2 | 0.002799 | 0.997201 |
| ENST00000461942 | PIPOX | 0.006899 | -0.9931 |
| NR_033709 | GFRA1 | 0.009701 | 0.990299 |
| NR_033709 | CATG00000092654.1 | 0.002697 | 0.997303 |
| NR_033709 | APBB3 | 0.00222 | -0.99778 |
| NR_033709 | PLEKHD1 | 0.005333 | 0.994667 |
| NR_033709 | MLLT1 | 0.006452 | -0.99355 |
| NR_033709 | DNAH6 | 0.002638 | -0.99736 |
| NR_033709 | HEATR9 | 0.007792 | -0.99221 |
| ENST00000550541 | C16orf45 | 0.007508 | -0.99249 |
| ENST00000550541 | PLAGL1 | 0.008811 | -0.99119 |
| ENST00000550541 | MYO7A | 0.001673 | 0.998327 |
| ENST00000550541 | ABHD5 | 0.00474 | 0.99526 |
| ENST00000550541 | OCSTAMP | 0.004982 | 0.995018 |
| ENST00000550541 | SP140 | 0.003701 | -0.9963 |
| ENST00000550541 | IL1R2 | 0.001624 | 0.998376 |
| ENST00000550541 | CROT | 6.71E-04 | -0.99933 |
| ENST00000520902 | UBE2V1 | 0.005106 | 0.994894 |
| ENST00000520902 | AIFM3 | 0.00454 | 0.99546 |
| ENST00000520902 | ZNF77 | 0.006304 | 0.993696 |
| ENST00000520902 | VGLL3 | 0.006972 | -0.99303 |
| ENST00000538665 | TMC5 | 0.009427 | -0.99057 |
| ENST00000538665 | STRADB | 0.00534 | -0.99466 |
| ENST00000538665 | EGF | 0.002182 | -0.99782 |
| ENST00000538665 | SHROOM3 | 0.008194 | -0.99181 |
| ENST00000538665 | CATG00000099282.1 | 0.002798 | 0.997202 |
| ENST00000538665 | ASB14 | 0.003544 | 0.996456 |
| ENST00000538665 | ABCA13 | 0.008032 | -0.99197 |
| ENST00000538665 | RHD | 0.001939 | -0.99806 |
| ENST00000538665 | CATG00000117842.1 | 0.008944 | -0.99106 |
| ENST00000538665 | C16orf86 | 0.009971 | 0.990029 |
| ENST00000321248 | TMED3 | 0.00519 | -0.99481 |
| ENST00000321248 | TAS2R42 | 0.008404 | 0.991596 |
| ENST00000321248 | PPP3CC | 0.003075 | -0.99693 |
| ENST00000321248 | PSRC1 | 0.005726 | -0.99427 |
| ENST00000321248 | LTA4H | 0.003513 | -0.99649 |
| ENST00000321248 | TUT4 | 0.006771 | -0.99323 |
| ENST00000321248 | WDR33 | 1.19E-04 | 0.999881 |
| ENST00000321248 | CATG00000053512.1 | 0.005801 | -0.9942 |
| ENST00000321248 | CATG00000063823.1 | 0.004586 | 0.995414 |
| ENST00000321248 | ZNF607 | 0.003172 | 0.996828 |
| ENST00000321248 | PSMD4 | 0.004567 | -0.99543 |
| ENST00000321248 | SLC12A3 | 0.007967 | -0.99203 |
| ENST00000321248 | OR6F1 | 0.001858 | 0.998142 |
| ENST00000321248 | ADAM28 | 0.00478 | -0.99522 |
| ENST00000321248 | CATG00000087047.1 | 0.005276 | 0.994724 |
| ENST00000321248 | RAD51AP2 | 0.00123 | -0.99877 |
| ENST00000490098 | CCT8L2 | 0.00462 | -0.99538 |
| ENST00000490098 | PAK5 | 0.009932 | -0.99007 |
| ENST00000490098 | PFKFB4 | 0.005399 | -0.9946 |
| ENST00000490098 | MECP2 | 0.001749 | 0.998251 |
| ENST00000490139 | BARHL1 | 0.005271 | 0.994729 |
| ENST00000490139 | RPL29 | 0.001234 | -0.99877 |
| ENST00000490139 | CCDC172 | 0.007098 | 0.992902 |
| ENST00000490139 | CNOT1 | 0.00104 | -0.99896 |
| ENST00000490139 | DEFA1 | 0.005121 | -0.99488 |
| ENST00000495888 | FCN2 | 0.004195 | 0.995805 |
| ENST00000495888 | CATG00000063531.1 | 6.00E-04 | 0.9994 |
| ENST00000495888 | UBE2V1 | 9.13E-04 | 0.999087 |
| ENST00000495888 | CEP44 | 8.82E-04 | 0.999118 |
| ENST00000495888 | PCBP4 | 0.002731 | -0.99727 |
| ENST00000495888 | NFE2L2 | 0.009672 | -0.99033 |
| ENST00000495888 | ABR | 0.008253 | 0.991747 |
| ENST00000495888 | SIVA1 | 0.006433 | -0.99357 |
| ENST00000495888 | OR5M8 | 0.006417 | 0.993583 |
| ENST00000495888 | NTF3 | 0.009717 | 0.990283 |
| ENST00000495888 | SMIM6 | 0.005473 | 0.994527 |
| ENST00000495888 | ERVW-1 | 0.002483 | -0.99752 |
| ENST00000592919 | RPS15 | 0.001973 | -0.99803 |
| ENST00000592919 | ARHGAP8 | 0.006198 | -0.9938 |
| ENST00000592919 | IL31RA | 0.008839 | -0.99116 |
| ENST00000592919 | SHISA6 | 0.002594 | 0.997406 |
| ENST00000592919 | CTNNBIP1 | 0.002654 | -0.99735 |
| ENST00000412647 | MFAP3L | 0.009276 | -0.99072 |
| ENST00000412647 | CATG00000086946.1 | 0.007371 | -0.99263 |
| ENST00000412647 | CATG00000039284.1 | 0.00346 | 0.99654 |
| ENST00000412647 | ORM1 | 0.003733 | -0.99627 |
| ENST00000453010 | PLAGL1 | 0.005563 | 0.994437 |
| ENST00000453010 | TNK2 | 0.007027 | 0.992973 |
| ENST00000453010 | RHBDD3 | 4.00E-04 | 0.9996 |
| ENST00000453010 | VGLL3 | 0.008718 | -0.99128 |
| ENST00000453010 | MMP2 | 1.37E-05 | -0.99999 |
| ENST00000453010 | FAM186A | 0.008971 | -0.99103 |
| NR_135298 | OCLN | 0.001751 | -0.99825 |
| NR_135298 | FAR1 | 0.006384 | -0.99362 |
| NR_135298 | AGAP1 | 0.006165 | 0.993835 |
| NR_135298 | LTF | 0.008806 | 0.991194 |
| NR_135298 | HILPDA | 0.001688 | 0.998312 |
| NR_135298 | KIF27 | 9.84E-04 | -0.99902 |
| NR_104203 | LDLRAD4 | 0.001874 | 0.998126 |
| NR_104203 | IFT80 | 0.005351 | 0.994649 |
| NR_104203 | DLEC1 | 0.005604 | -0.9944 |
| ENST00000487343 | B3GNT3 | 0.001791 | -0.99821 |
| ENST00000487343 | PFDN6 | 0.006025 | -0.99398 |
| ENST00000487343 | PPP3CC | 0.003553 | -0.99645 |
| ENST00000487343 | PSRC1 | 0.007772 | -0.99223 |
| ENST00000487343 | ELOF1 | 0.008892 | 0.991108 |
| ENST00000487343 | IMPA2 | 0.00282 | -0.99718 |
| ENST00000487343 | FOXO6 | 0.005437 | 0.994563 |
| ENST00000487343 | DGAT2L6 | 0.003877 | 0.996123 |
| ENST00000487343 | CATG00000053512.1 | 0.002628 | -0.99737 |
| ENST00000487343 | HLF | 0.003175 | 0.996825 |
| ENST00000487343 | CSTL1 | 0.005946 | 0.994054 |
| ENST00000487343 | SLC12A3 | 0.00589 | -0.99411 |
| ENST00000487343 | PPP5D1 | 2.34E-05 | 0.999977 |
| ENST00000487343 | JDP2 | 0.006632 | 0.993368 |
| ENST00000487343 | TAF8 | 0.009352 | -0.99065 |
| ENST00000487343 | OR6F1 | 0.009775 | 0.990225 |
| ENST00000487343 | PSMA1 | 0.001714 | -0.99829 |
| ENST00000487343 | OXT | 0.006029 | 0.993971 |
| ENST00000487343 | FAM174A | 0.005048 | -0.99495 |
| ENST00000487343 | CFAP410 | 0.005452 | 0.994548 |
| NR_130736 | TMED3 | 0.003314 | -0.99669 |
| NR_130736 | PPP3CC | 0.003942 | -0.99606 |
| NR_130736 | PSRC1 | 0.008865 | -0.99114 |
| NR_130736 | LTA4H | 0.007307 | -0.99269 |
| NR_130736 | FNDC10 | 0.007495 | 0.992505 |
| NR_130736 | WDR33 | 7.73E-04 | 0.999227 |
| NR_130736 | CATG00000053512.1 | 0.00715 | -0.99285 |
| NR_130736 | IDH1 | 0.008799 | -0.9912 |
| NR_130736 | CATG00000063823.1 | 0.005315 | 0.994685 |
| NR_130736 | ZNF607 | 0.001168 | 0.998832 |
| NR_130736 | PSMD4 | 0.005767 | -0.99423 |
| NR_130736 | OR6F1 | 3.18E-04 | 0.999682 |
| NR_130736 | ADAM28 | 0.006097 | -0.9939 |
| NR_130736 | CATG00000087047.1 | 0.009776 | 0.990224 |
| NR_130736 | RAD51AP2 | 0.003662 | -0.99634 |
| NR_037910 | MYH7 | 0.008624 | 0.991376 |
| NR_037910 | C1orf198 | 0.005494 | -0.99451 |
| NR_037910 | NABP2 | 0.009916 | -0.99008 |
| NR_037910 | TATDN3 | 0.00408 | -0.99592 |
| NR_037910 | MRPL10 | 3.03E-04 | 0.999697 |
| NR_037910 | SMIM33 | 0.002336 | -0.99766 |
